# Genetic variation in the immunosuppression pathway genes and breast cancer susceptibility: a pooled analysis of 42,510 cases and 40,577 controls from the Breast Cancer Association Consortium

**DOI:** 10.1007/s00439-015-1616-8

**Published:** 2015-11-30

**Authors:** Jieping Lei, Anja Rudolph, Kirsten B. Moysich, Sabine Behrens, Ellen L. Goode, Manjeet K. Bolla, Joe Dennis, Alison M. Dunning, Douglas F. Easton, Qin Wang, Javier Benitez, John L. Hopper, Melissa C. Southey, Marjanka K. Schmidt, Annegien Broeks, Peter A. Fasching, Lothar Haeberle, Julian Peto, Isabel dos-Santos-Silva, Elinor J. Sawyer, Ian Tomlinson, Barbara Burwinkel, Frederik Marmé, Pascal Guénel, Thérèse Truong, Stig E. Bojesen, Henrik Flyger, Sune F. Nielsen, Børge G. Nordestgaard, Anna González-Neira, Primitiva Menéndez, Hoda Anton-Culver, Susan L. Neuhausen, Hermann Brenner, Volker Arndt, Alfons Meindl, Rita K. Schmutzler, Hiltrud Brauch, Ute Hamann, Heli Nevanlinna, Rainer Fagerholm, Thilo Dörk, Natalia V. Bogdanova, Arto Mannermaa, Jaana M. Hartikainen, Australian Ovarian Study Group, kConFab Investigators, Laurien Van Dijck, Ann Smeets, Dieter Flesch-Janys, Ursula Eilber, Paolo Radice, Paolo Peterlongo, Fergus J. Couch, Emily Hallberg, Graham G. Giles, Roger L. Milne, Christopher A. Haiman, Fredrick Schumacher, Jacques Simard, Mark S. Goldberg, Vessela Kristensen, Anne-Lise Borresen-Dale, Wei Zheng, Alicia Beeghly-Fadiel, Robert Winqvist, Mervi Grip, Irene L. Andrulis, Gord Glendon, Montserrat García-Closas, Jonine Figueroa, Kamila Czene, Judith S. Brand, Hatef Darabi, Mikael Eriksson, Per Hall, Jingmei Li, Angela Cox, Simon S. Cross, Paul D. P. Pharoah, Mitul Shah, Maria Kabisch, Diana Torres, Anna Jakubowska, Jan Lubinski, Foluso Ademuyiwa, Christine B. Ambrosone, Anthony Swerdlow, Michael Jones, Jenny Chang-Claude

**Affiliations:** Division of Cancer Epidemiology, German Cancer Research Center (DKFZ), Im Neuenheimer Feld 581, 69120 Heidelberg, Germany; Department of Cancer Prevention and Control, Roswell Park Cancer Institute, Buffalo, NY USA; Department of Health Sciences Research, Mayo Clinic, Rochester, MN USA; Centre for Cancer Genetic Epidemiology, Department of Public Health and Primary Care, University of Cambridge, Cambridge, UK; Centre for Cancer Genetic Epidemiology, Department of Oncology, University of Cambridge, Cambridge, UK; Human Cancer Genetics Program, Spanish National Cancer Research Centre, Madrid, Spain; Centro de Investigación en Red de Enfermedades Raras, Valencia, Spain; Centre for Epidemiology and Biostatistics, Melbourne School of Population and Global Health, The University of Melbourne, Melbourne, Australia; Department of Pathology, The University of Melbourne, Melbourne, Australia; Netherlands Cancer Institute, Antoni van Leeuwenhoek Hospital, Amsterdam, The Netherlands; Department of Gynaecology and Obstetrics, University Hospital Erlangen, Friedrich-Alexander University Erlangen-Nuremberg, Comprehensive Cancer Center Erlangen-EMN, Erlangen, Germany; David Geffen School of Medicine, Department of Medicine Division of Hematology and Oncology, University of California at Los Angeles, Los Angeles, CA USA; Department of Non-Communicable Disease Epidemiology, London School of Hygiene and Tropical Medicine, London, UK; Research Oncology, Guy’s Hospital, King’s College London, London, UK; Wellcome Trust Centre for Human Genetics and Oxford NIHR Biomedical Research Centre, University of Oxford, Oxford, UK; Department of Obstetrics and Gynecology, University of Heidelberg, Heidelberg, Germany; Molecular Epidemiology Group, German Cancer Research Center (DKFZ), Heidelberg, Germany; National Center for Tumor Diseases, University of Heidelberg, Heidelberg, Germany; Environmental Epidemiology of Cancer, Center for Research in Epidemiology and Population Health, INSERM, Villejuif, France; University Paris-Sud, Villejuif, France; Copenhagen General Population Study, Herlev Hospital, Copenhagen University Hospital, Herlev, Denmark; Department of Clinical Biochemistry, Herlev Hospital, Copenhagen University Hospital, Herlev, Denmark; Faculty of Health and Medical Sciences, University of Copenhagen, Copenhagen, Denmark; Department of Breast Surgery, Herlev Hospital, Copenhagen University Hospital, Herlev, Denmark; Servicio de Anatomía Patológica, Hospital Monte Naranco, Oviedo, Spain; Department of Epidemiology, University of California Irvine, Irvine, CA USA; Beckman Research Institute of City of Hope, Duarte, CA USA; Division of Clinical Epidemiology and Aging Research, German Cancer Research Center (DKFZ), Heidelberg, Germany; Division of Preventive Oncology, National Center for Tumor Diseases (NCT) and German Cancer Research Center (DKFZ), Heidelberg, Germany; German Cancer Consortium (DKTK), German Cancer Research Center (DKFZ), Heidelberg, Germany; Division of Gynaecology and Obstetrics, Technische Universität München, Munich, Germany; Center for Hereditary Breast and Ovarian Cancer, University Hospital of Cologne, Cologne, Germany; Center for Integrated Oncology (CIO), University Hospital of Cologne, Cologne, Germany; Center for Molecular Medicine Cologne (CMMC), University of Cologne, Cologne, Germany; Dr. Margarete Fischer-Bosch-Institute of Clinical Pharmacology Stuttgart, Stuttgart, Germany; University of Tübingen, Tübingen, Germany; Molecular Genetics of Breast Cancer, German Cancer Research Center (DKFZ), Heidelberg, Germany; Department of Obstetrics and Gynecology, Helsinki University Hospital, University of Helsinki, Helsinki, Finland; Gynaecology Research Unit, Hannover Medical School, Hannover, Germany; Department of Radiation Oncology, Hannover Medical School, Hannover, Germany; Cancer Center, Kuopio University Hospital, Kuopio, Finland; Institute of Clinical Medicine, Pathology and Forensic Medicine, University of Eastern Finland, Kuopio, Finland; Imaging Center, Department of Clinical Pathology, Kuopio University Hospital, Kuopio, Finland; Department of Genetics, QIMR Berghofer Medical Research Institute, Brisbane, QLD Australia; The Peter MacCallum Cancer Centre, Melbourne, VIC Australia; VIB Vesalius Research Center, Department of Oncology, University of Leuven, Leuven, Belgium; Multidisciplinary Breast Center, University Hospitals Leuven, University of Leuven, Leuven, Belgium; Institute for Medical Biometrics and Epidemiology, University Medical Center Hamburg-Eppendorf, Hamburg, Germany; Department of Cancer Epidemiology, Clinical Cancer Registry, University Medical Center Hamburg-Eppendorf, Hamburg, Germany; Unit of Molecular Bases of Genetic Risk and Genetic Testing, Department of Preventive and Predictive Medicine, Fondazione IRCCS (Istituto Di Ricovero e Cura a Carattere Scientifico) Istituto Nazionale dei Tumori (INT), Milan, Italy; IFOM, Fondazione Istituto FIRC (Italian Foundation of Cancer Research) di Oncologia Molecolare, Milan, Italy; Department of Laboratory Medicine and Pathology, Mayo Clinic, Rochester, MN USA; Cancer Epidemiology Centre, Cancer Council Victoria, Melbourne, Australia; Department of Preventive Medicine, Keck School of Medicine, University of Southern California, Los Angeles, CA USA; Genomics Center, Centre Hospitalier Universitaire de Québec Research Center, Laval University, Québec City, Canada; Department of Medicine, McGill University, Montreal, Canada; Division of Clinical Epidemiology, Royal Victoria Hospital, McGill University, Montreal, Canada; Department of Genetics, Institute for Cancer Research, Oslo University Hospital Radiumhospitalet, Oslo, Norway; K.G. Jebsen Center for Breast Cancer Research, Institute of Clinical Medicine, Faculty of Medicine, University of Oslo, Oslo, Norway; Department of Clinical Molecular Biology, Oslo University Hospital, University of Oslo, Oslo, Norway; Division of Epidemiology, Department of Medicine, Vanderbilt-Ingram Cancer Center, Vanderbilt University School of Medicine, Nashville, TN USA; Laboratory of Cancer Genetics and Tumor Biology, Department of Clinical Chemistry and Biocenter Oulu, University of Oulu, Oulu, Finland; Central Finland Hospital District, Jyväskylä Central Hospital, Jyväskylä, Finland; Department of Surgery, Oulu University Hospital, University of Oulu, Oulu, Finland; Lunenfeld-Tanenbaum Research Institute of Mount Sinai Hospital, Toronto, Canada; Department of Molecular Genetics, University of Toronto, Toronto, Canada; Division of Genetics and Epidemiology, The Institute of Cancer Research, London, UK; Division of Cancer Epidemiology and Genetics, National Cancer Institute, Rockville, MD USA; Department of Medical Epidemiology and Biostatistics, Karolinska Institutet, Stockholm, Sweden; Sheffield Cancer Research Centre, Department of Oncology, University of Sheffield, Sheffield, UK; Academic Unit of Pathology, Department of Neuroscience, University of Sheffield, Sheffield, UK; Institute of Human Genetics, Pontificia Universidad Javeriana, Bogota, Colombia; Department of Genetics and Pathology, Pomeranian Medical University, Szczecin, Poland; Roswell Park Cancer Institute, Buffalo, NY USA; Division of Genetics and Epidemiology, Institute of Cancer Research, London, UK; Division of Breast Cancer Research, Institute of Cancer Research, London, UK; University Cancer Center Hamburg (UCCH), University Medical Center Hamburg-Eppendorf, Hamburg, Germany

## Abstract

Immunosuppression plays a pivotal role in assisting tumors to evade immune destruction and promoting tumor development. We hypothesized that genetic variation in the immunosuppression pathway genes may be implicated in breast cancer tumorigenesis. We included 42,510 female breast cancer cases and 40,577 controls of European ancestry from 37 studies in the Breast Cancer Association Consortium (2015) with available genotype data for 3595 single nucleotide polymorphisms (SNPs) in 133 candidate genes. Associations between genotyped SNPs and overall breast cancer risk, and secondarily according to estrogen receptor (ER) status, were assessed using multiple logistic regression models. Gene-level associations were assessed based on principal component analysis. Gene expression analyses were conducted using RNA sequencing level 3 data from The Cancer Genome Atlas for 989 breast tumor samples and 113 matched normal tissue samples. SNP rs1905339 (A>G) in the *STAT3* region was associated with an increased breast cancer risk (per allele odds ratio 1.05, 95 % confidence interval 1.03–1.08; *p* value = 1.4 × 10^−6^). The association did not differ significantly by ER status. On the gene level, in addition to *TGFBR2* and *CCND1*, *IL5* and *GM*-*CSF* showed the strongest associations with overall breast cancer risk (*p* value = 1.0 × 10^−3^ and 7.0 × 10^−3^, respectively). Furthermore, *STAT3* and *IL5* but not *GM*-*CSF* were differentially expressed between breast tumor tissue and normal tissue (*p* value = 2.5 × 10^−3^, 4.5 × 10^−4^ and 0.63, respectively). Our data provide evidence that the immunosuppression pathway genes *STAT3,IL5,* and *GM*-*CSF* may be novel susceptibility loci for breast cancer in women of European ancestry.

**Electronic supplementary material:**

The online version of this article (doi:10.1007/s00439-015-1616-8) contains supplementary material, which is available to authorized users.

## Introduction

Breast cancer is the most frequent cancer among women and the second leading cause of cancer-related death after lung cancer in Europe. In addition to genetic variants with high and moderate penetrance, more than 90 common germline genetic variants contributing to breast cancer risk have been identified, comprising about 37 % of the familial relative risk of the disease (Michailidou et al. 2013, 2015). This suggests that a substantial portion of inherited variation has not yet been identified. In addition, most of the known common susceptibility variants reside in non-coding regions and result in subtle regulation of gene expression. The biological mechanisms through which genetic variants exert their functions are still not entirely understood.

The ability to evade immune destruction has been increasingly recognized as a key hallmark of tumors (Hanahan and Weinberg 2011). Tumor cells may secrete immunosuppressive factors like TGF-β which hampers infiltrating cytotoxic T lymphocytes and natural killer cells (Yang et al. 2010). Inflammatory cells like regulatory T cells (Treg cells), a subset of CD4+ T lymphocytes, as well as myeloid-derived suppressor cells (MDSCs) may be recruited into the tumor environment, which are actively immunosuppressive (Lindau et al. 2013; Reisfeld 2013). Higher prevalence of Treg cells has been found in various cancers (Chang et al. 2010; Michel et al. 2008; Watanabe et al. 2002), including breast cancer (Bates et al. 2006). There is evidence that tumor infiltrating Treg cells endowed with immunosuppressive potential are associated with tumor progression and unfavorable prognosis, especially in estrogen receptor (ER)-negative breast cancer (Bates et al. 2006; Kim et al. 2013; Liu et al. 2012a). In addition, infiltrating MDSCs were also found in murine mammary tumor models (Aliper et al. 2014; Gad et al. 2014), but their relevance for breast cancer patients also in terms of prognosis is not well-understood. Furthermore, previous association studies have identified susceptibility alleles for breast cancer in two genes, *TGFBR2* (transforming growth factor beta receptor II) (Michailidou et al. 2013) and *CCND1* (cyclin D1) (French et al. 2013), which may be involved in immune regulation in cancer patients (Gabrilovich and Nagaraj 2009; Krieg and Boyman 2009), including those with breast cancer. We hypothesized that immunosuppression pathway genes, particularly those relevant to Treg cell and MDSC functions, may harbor further susceptibility variants associated with breast cancer tumorigenesis, with a possible differential association by ER status.

In this analysis, we investigated associations between breast cancer risk and single nucleotide polymorphisms (SNPs) in 133 candidate genes in the immunosuppression pathway in individual level data from the Breast Cancer Association Consortium (BCAC). We also assessed associations with breast cancer risk at the gene and pathway levels. Furthermore, we used publicly available datasets through the UCSC Genome Browser (2015) to examine the putative genetic susceptibility loci for potential regulatory function.

## Materials and methods

### Study participants

In this analysis, participants were restricted to 83,087 women of European ancestry from 37 case–control studies participating in BCAC, including 42,510 invasive breast cancer cases with stage I–III disease and 40,577 cancer-free controls. Of all breast cancer patients, 26,094 were known to have ER-positive disease and 6870 to have ER-negative disease. Details of included studies are summarized in Online Resource 1. All studies were approved by the relevant ethics committees and all participants gave informed consent (Michailidou et al. 2013).

### Candidate gene selection

Candidate genes relevant to the Treg cell and MDSC pathways were identified through a comprehensive literature review in PubMed (DeNardo et al. 2010; DeNardo and Coussens 2007; Driessens et al. 2009; Gabrilovich and Nagaraj 2009; Krieg and Boyman 2009; Mills 2004; Ostrand-Rosenberg 2008; Poschke et al. 2011; Sakaguchi et al. 2013; Sica et al. 2008; Wilczynski and Duechler 2010; Zitvogel et al. 2006; Zou 2005), using the search terms “immunosuppression”/“immunosuppressive”, “regulatory T cells”/“Treg cells”/“FOXP3+ T cells”, “myeloid derived suppressor cells”/“MDSCs”, “immunosurveillance”, and “tumor escape”. The final candidate gene list included 133 immunosuppression-related genes (Online Resource 2). SNPs within 50 kb upstream and downstream of each gene were identified using HapMap CEU genotype data (2015) and dbSNP 126.

### SNP association analyses

For the BCAC studies, genotyping was carried out using a custom Illumina iSelect array (iCOGS) designed for the Collaborative Oncological Gene-Environment Study (COGS) project (Michailidou et al. 2013). Of the 211,155 SNPs on the array, 4246 were located within 50 kb of the selected candidate genes. Centralized quality control of genotype data led to the exclusion of 651 SNPs. The exclusion criteria included a call rate less than 95 % in all samples genotyped with iCOGS, minor allele frequency (MAF) less than 0.05 in all samples, evidence of deviation from Hardy–Weinberg equilibrium (HWE) at *p* value <10^−7^, and concordance in duplicate samples less than 98 % (Michailidou et al. 2013). A total of 3595 SNPs passed all quality controls and was analyzed.

Per-allele associations with the number of minor alleles were assessed using multiple logistic regression models, adjusted for study, age (at diagnosis for cases or at recruitment for controls) and nine principal components (PCs) derived based on genotyped variants to account for European population substructure. We assessed the associations of SNPs with overall breast cancer risk as primary analyses, and then restricted to ER-positive (26,094 cases and 40,577 controls) and ER-negative subtypes (6870 cases and 40,577 controls) as secondary analyses. Differences in the associations between ER-positive and ER-negative diseases were assessed by case-only analyses, using ER status as the dependent variable. To determine the number of “independent” SNPs for adjustment of multiple testing, we applied the option “--indep-pairwise” in PLINK (Purcell et al. 2007). SNPs were pruned by linkage disequilibrium (LD) of *r*^2^ < 0.2 for a window size of 50 SNPs and step size of 10 SNPs, yielding 689 “independent” SNPs. The significance threshold using Bonferroni correction corresponding to an alpha of 5 % was 7.3 × 10^−5^.

In order to identify more strongly associated variants, genotypes were imputed for SNPs at the locus for which strongest evidence of association was observed, via a two-stage procedure involving SHAPEIT (Howie et al. 2012) and IMPUTEv2 (Howie et al. 2009), using the 1000 Genomes Project data as the reference panel (Abecasis et al. 2012). Details of the imputation procedure are described elsewhere (Michailidou et al. 2015). Models assessing associations with imputed SNPs were adjusted for 16 PCs based on 1000 Genome imputed data to further improve adjustment for population stratification. To determine independent signals within imputed SNPs at *STAT3*, we ran a stepwise forward multiple logistic regression model including the most significant genotyped SNP rs1905339 and all imputed SNPs, adjusted for study, age and 16 PCs.

SNP association analyses and case-only analyses were all conducted using SAS 9.3 (Cary, NC, USA). All tests were two-sided.

For multiple associated SNPs located at the same gene, a Microsoft Excel SNP tool created by Chen et al. (2009) and the software HaploView 4.2 (Barrett et al. 2005) were used to examine LD structure between these SNPs. To be able to inspect LD structures and also for gene-level analyses, allele dosages of imputed SNPs had to be converted into the most probable genotypes. Therefore, we categorized the imputed allele dosage between [0, 0.5] as homozygote of the reference allele, the value between [0.5, 1.5] as heterozygote, and the value between [1.5, 2.0] as homozygote of the counted allele. The regional association plot was generated using the online tool LocusZoom (Pruim et al. 2010).

### Gene-level and pathway association analyses

Gene-level associations were determined by a subset of PCs, which were derived from a linear combination of SNPs in each gene explaining 80 % of the variation in the joint distribution of all relevant SNPs. Associations with derived PCs were assessed within a logistic regression framework (Biernacka et al. 2012), for overall breast cancer, ER-positive and ER-negative diseases, respectively. Pathway association of the immunosuppression pathway was assessed based on a global test of association by combining the gene-level *p* values via the Gamma method (Biernacka et al. 2012). For gene-level associations, associations with *p* value <3.8 × 10^−4^ (Bonferroni correction) were considered statistically significant. To gain empirical *p* values for gene-level associations of *TGFBR2* and *CCND1* as well as for the pathway association, a Monte Carlo procedure was used with up to 1,000,000 randomizations (Biernacka et al. 2012). An exact binomial test based on the results of the single SNPs association analyses was carried out to estimate enrichment of association in the immunosuppression pathway. Gene-level and pathway association analyses were carried out in R (version 3.1.1) using the package ‘GSAgm’ version 1.0.

### Haplotype analyses

To follow up the interesting gene associations observed, haplotype analyses were performed to identify potential susceptibility variants. Haplotype frequencies were determined with the use of the estimation maximization (EM) algorithm (Long et al. 1995) implemented in PROC HAPLOTYPE in SAS 9.3 (Cary, NC, USA). Haplotypes with frequency more or equal than 1 % were examined and the most common haplotype was used as the reference. Rare haplotypes with frequency less than 1 % were grouped into one category. Haplotype-specific odds ratios (ORs) and 95 % confidence intervals (CIs) were estimated within a multiple logistic regression framework, adjusted for the same covariates as in the single SNP association analyses. Global *p* values for association of haplotypes with breast cancer risk were computed using a likelihood ratio test comparing models with and without haplotypes of the gene of interest.

### Gene expression analyses

In order to examine whether potential causative genes influence RNA expression in breast tumor tissue, we downloaded RNA sequence level 3 data from The Cancer Genome Atlas (TCGA) (2015). We retrieved the RNA expression level as the form of RNA-Seq by expectation–maximization (RSEM) based on the IlluminaHiSeq_RNASeqV2 array. Gene expression differences in RNA levels between 989 invasive breast cancer tissues and 113 matched normal tissues for four genes of interest (*STAT3*, *PTRF*, *IL5*, and *GM*-*CSF*) were analyzed using a two-sided Wilcoxon–Mann–Whiney test. In addition, data from 183 breast tissues in the GTEx (V6) (2015) publically available online databases were evaluated to obtain information on whether the most interesting variants (rs1905339, rs8074296, rs146170568, chr17:40607850:I and rs77942990) were expression quantitative trait loci (eQTL) for any gene. Also, GTEx was queried to obtain information on whether the five variants were eQTL for *STAT3* or *PTRF*.

### Functional annotation

To investigate potential regulatory functions of interesting polymorphisms, we used the Encyclopedia of DNA Elements (ENCODE) database through the UCSC Genome Browser as well as Haploreg v4 (Ward and Kellis 2012).

## Results

Selected characteristics of the study population are described in Table 1. The controls and breast cancer patients included in this study had comparable mean reference ages of 54.8 and 55.9 years and also the proportion of postmenopausal women was similar (68 % in controls and 69 % in breast cancer patients). The proportion of women indicating a family history of breast cancer in first degree relatives was as expected greater in breast cancer patients (25 %) than in controls (12 %).

**Table 1:** Characteristics of breast cancer cases and controls

Characteristic	Controls		Cases	
	No.	%	No.	%
Total number	40,577		42,510	
Age (mean, SD)	54.8	12.0	55.9	11.6
Family history of breast cancer				
No	20,940	88	24,397	75
Yes	2829	12	7971	25
Unknown/missing	16,808		10,142	
Menopausal status				
Pre/perimenopausal	9174	32	9296	31
Postmenopausal	19,753	68	20,714	69
Unknown/missing	11,650		12,500	
Estrogen receptor status				
Negative			6870	21
Positive			26,094	79
Unknown/missing			9546	
Progesterone receptor status				
Negative			9299	33
Positive			19,017	67
Unknown/missing			14,194	
Triple-negative cancer				
No			13,675	84
Yes			2600	16
Unknown/missing			26,235	
Stage				
0			25	0.1
I			12,044	50
II			9711	40
III			1975	8
IV			496	2
Unknown/missing			18,259	
Grade				
Well differentiated			6125	21
Moderately differentiated			14,092	48
Poorly/un-differentiated			8937	31
Unknown/missing			13,356	

*SD* standard deviation

### Single SNP associations

Excluding the known *TGFBR2* and *CCND1* breast cancer susceptibility loci, the quantile–quantile (QQ) plot for associations with overall breast cancer risk for the genotyped SNPs of the other candidate genes indicated deviation from expected *p* values and thus evidence of further SNPs associated with breast cancer risk (Online Resource 3). Genetic associations with overall breast cancer risk for all assessed 3595 SNPs are summarized in Online Resource 4.

Four independent genotyped SNPs (LD *r*^2^ < 0.3) were significantly associated with breast cancer risk at *p* value <7.3 × 10^−5^, accounting for the multiple comparisons (Table 2). The four significant SNPs were located in or near *TGFBR2*, *STAT3* and *CCND1*. Since *TGFBR2* and *CCND1* have been identified as breast cancer susceptibility loci in previous studies (French et al. 2013; Michailidou et al. 2013; Rhie et al. 2013), we focused on the association of the SNP at *STAT3*. The variant rs1905339 (A>G) at *STAT3* was positively associated with overall breast cancer risk (per allele odds ratio (OR) 1.05, 95 % confidence interval (CI) 1.03–1.08, *p* value = 1.4 × 10^−6^). It showed similar associations with ER-positive and ER-negative cancers (Online Resource 5). We did not observe further SNPs that were significantly associated with ER-positive or ER-negative disease (data not shown).

**Table 2:** *TGFBR2*, *CCND1* and *STAT3* SNPs associated with overall breast cancer risk in women of European ancestry after Bonferroni correction (*p* value <7.3 × 10^−5^)

SNP	Chr.	Position^a^	Gene	Minor allele	MAF cases	MAF controls	Cases	Controls	OR (95 %CI)^b^	*p* value
rs1431131	3	30,675,880	*TGFBR2*	A	0.37	0.36	42,508	40,574	1.06 (1.04–1.08)	2.6 × 10^−8^
rs11924422	3	30,677,484	*TGFBR2*	C	0.40	0.41	42,491	40,572	0.95 (0.94–0.97)	6.9 × 10^−6^
rs7177	11	69,466,115	*CCND1*	C	0.46	0.47	42,411	40,496	0.96 (0.94–0.98)	2.7 × 10^−5^
rs1905339	17	40,582,296	*STAT3*	G	0.34	0.33	42,504	40,576	1.05 (1.03–1.08)	1.4 × 10^−6^

*SNP* single nucleotide polymorphism, *Chr*. chromosome, *MAF* minor allele frequency, *OR* odds ratio, *CI* confidence interval, *TGFBR2* transforming growth factor beta receptor II, *CCND1* cyclin D1, *STAT3* signal transducer and activator of transcription 3

^a^Build 37

^b^OR per minor allele, adjusted for age, study and nine European principal components

**Fig. 1:**
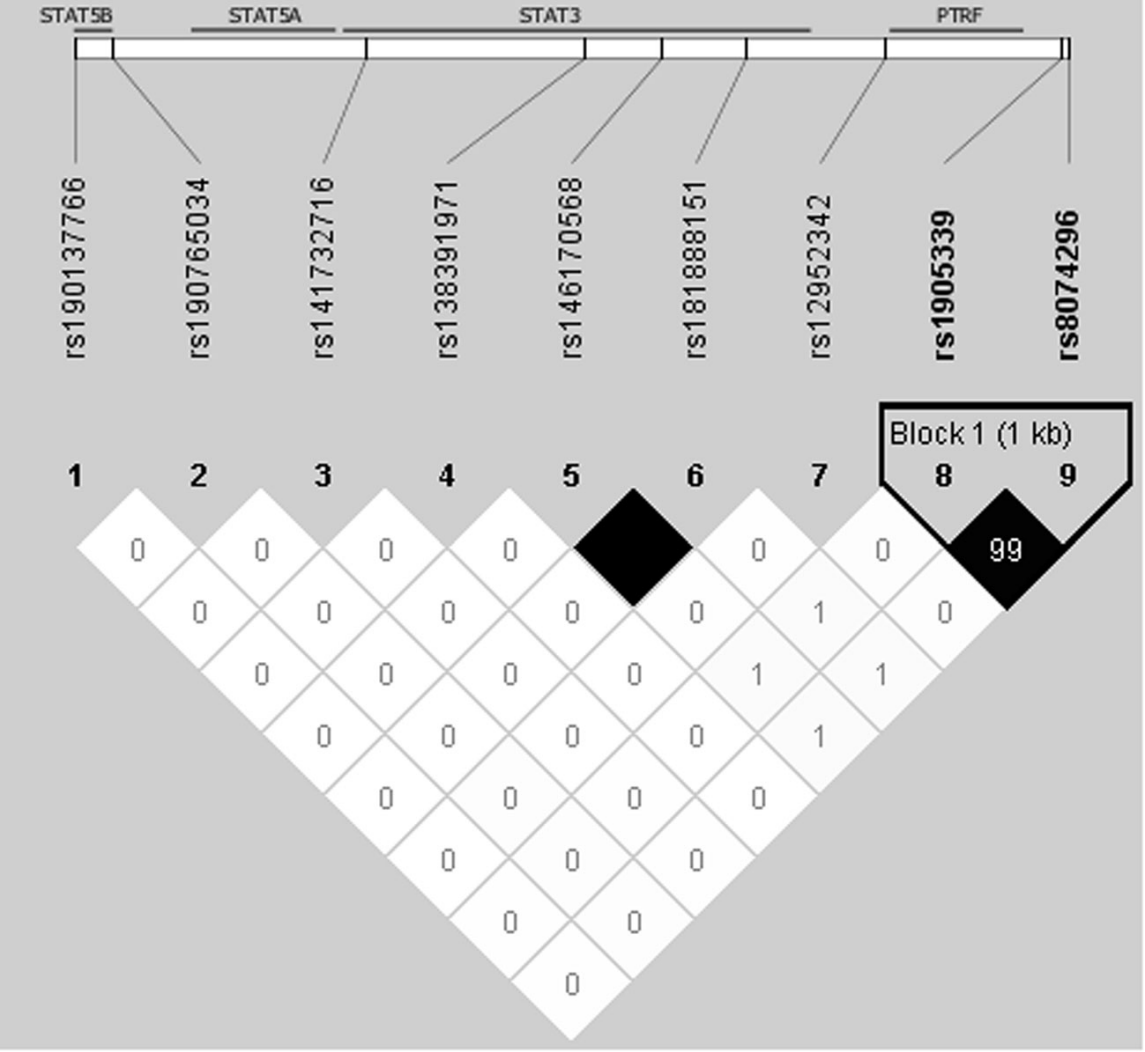
Linkage disequilibrium plot showing *r* ^2^ values and color schemes for the genotyped SNP rs1905339 and seven independent imputed SNPs as well as imputed SNP rs181888151 within ±50 kb of *STAT3.* The linkage disequilibrium (LD) plot shows that SNP rs1905339 is in strong LD with the imputed SNP rs8074296 (*r* ^2^ = 0.99), and independent of the other six imputed SNPs (*r* ^2^ ≤ 0.01) at *STAT3.* LD was estimated based on control data

**Fig. 2:**
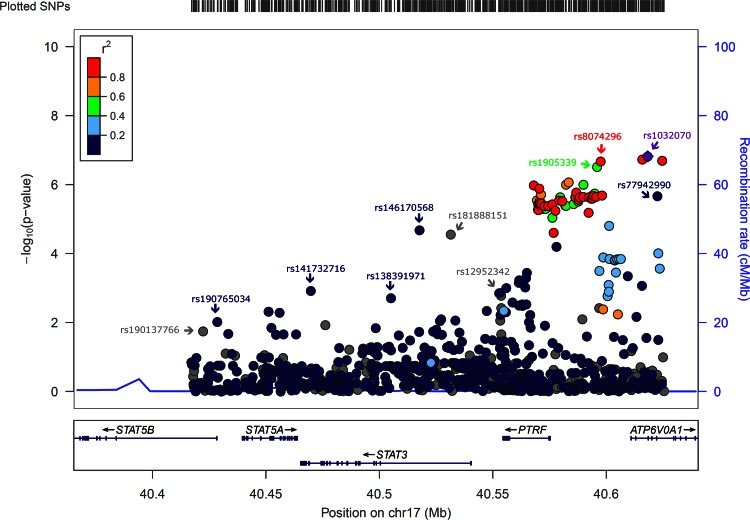
Regional association plot for the genotyped SNP rs1905339 and 885 imputed SNPs within ±50 kb of *STAT3* and *PTRF.* Each *dot* represents an SNP. The *color of each dot* reflects the extent of linkage disequilibrium (*r* ^2^) with SNP rs1032070 (in *purple diamond*). Genomic positions of SNPs were plotted based on hg19/1000 Genomes Mar 2012 European. Association is represented at the −log10 scale. *cM/Mb* centiMorgans/megabase

To identify additional susceptibility variants at *STAT3*, we further investigated 707 SNPs that were well-imputed (imputation accuracy *r*^2^ > 0.3) and with MAF >0.01 spanning a ±50 kb window around *STAT3*. Seven independent signals at *STAT3* were found through the stepwise forward selection procedure. The genotyped SNP rs1905339 was not selected. The imputed SNP rs8074296 (A>G), which was in high LD with rs1905339 (*r*^2^ = 0.99), showed a comparable OR for the association with overall breast cancer risk with a more extreme *p* value (per allele OR 1.05, 95 % CI 1.03–1.08, *p* value = 8.6 × 10^−7^, Table 3). A second imputed SNP rs146170568 (C>T), associated with a per allele OR of 1.32 (95 % CI 1.16–1.50, *p* value = 2.1 × 10^−5^), was still strongly associated at a *p* value of 3.2 × 10^−4^ after accounting for rs8074296 (Table 3). None of the independently associated imputed SNPs besides rs8074296 were correlated with rs1905339 or with each other (*r*^2^ ≤ 0.01, Fig. 1). As rs8074296 and rs1905339 are located closer to *PTRF* than to *STAT3*, we additionally analyzed data of 178 imputed variants located within ±50 kb of *PTRF*. Associations of most additional variants in the *PTRF* region with breast cancer risk were attenuated in analyses conditioning on rs8074296 (Table 4). The variants chr17:40607850:I and rs77942990 still showed a strong association with breast cancer risk (per allele OR 1.09, 95 % CI 1.04–1.15, *p* value = 0.0005; and per allele OR 1.09, 95 % CI 1.04–1.15, *p* value = 0.0007, respectively). These two variants were also not in LD with rs8074296 (*r*^2^ = 0.09 and 0.07, respectively) while all other variants in Table 4 were at least in moderate LD with rs8074296 (*r*^2^ ≥ 0.46, Online Resource 6). The LD plot (Online Resource 6) also shows that chr17:40607850:I and rs77942990 are in high LD (*r*^2^ = 0.83). A regional association plot for the genotyped SNP rs1905339 and all 885 imputed SNPs within ±50 kb of *STAT3* and *PTRF* included in this analysis is shown in Fig. 2. Associations of SNPs shown in Table 3 as well as associations of chr17:40607850:I and rs77942990 with breast cancer risk were not significantly heterogeneous between studies (all *p* values for heterogeneity >0.1); forest plots can be found in Online Resource 7 to 16.

**Table 3:** Associations with overall breast cancer risk for seven independent imputed SNPs at *STAT3* in women of European ancestry

SNP	Chr.	Position^a^	Counted allele	AF^b^	Cases	Controls	Single SNP analysis		Conditional analysis^d^	
							OR (95 % CI)^c^	*p* value	OR (95 %CI)^c^	*p* value
rs8074296	17	40,583,421	G	0.336	42,510	40,577	1.05 (1.03–1.08)	8.6 × 10^−7^	1.05 (1.03–1.07)	2.3 × 10^−5^
rs146170568	17	40,517,716	T	0.005	42,510	40,577	1.32 (1.16–1.50)	2.1 × 10^−5^	1.27 (1.11–1.44)	3.2 × 10^−4^
rs141732716	17	40,469,832	A	0.005	42,510	40,577	1.38 (1.14–1.68)	0.001	1.33 (1.09–1.62)	0.004
rs138391971	17	40,505,106	G	0.003	42,510	40,577	0.60 (0.43–0.83)	0.002	0.61 (0.44–0.85)	0.003
rs12952342	17	40,553,640	G	0.119	42,510	40,577	1.07 (1.03–1.12)	0.002	1.07 (1.02–1.11)	0.005
rs190765034	17	40,428,622	G	0.026	42,510	40,577	1.14 (1.03–1.25)	0.010	1.17 (1.06–1.29)	0.002
rs190137766	17	40,422,371	T	0.002	42,510	40,577	0.68 (0.50–0.94)	0.018	0.66 (0.48–0.90)	0.009

*SNP* single nucleotide polymorphism, *Chr*. chromosome, *OR* odds ratio, *CI* confidence interval, *STAT3* signal transducer and activator of transcription 3

^a^Build 37

^b^Allele frequency (AF) of counted allele

^c^OR per counted allele, adjusted for age, study and 16 European principal components

^d^Each SNP was tested adjusting for rs8074296, age, study and 16 European principal components. Estimate for rs8074296 is based on model including rs146170568

**Table 4:** Associations with overall breast cancer risk for 19 imputed variants near *PTRF* in women of European ancestry

SNP	Chr	Position^a^	Counted allele	AF^b^	Cases	Controls	Single SNP analysis			Conditional analysis^d^		
							OR^c^	(95 % CI)	*p* value	OR^c^	(95 % CI)	*p* value
rs8074296	17	40,583,421	G	0.336	42,510	40,577	1.05	(1.03–1.08)	8.6 × 10^−7^	1.04	(1.02–1.06)	0.0006
rs1032070	17	40,618,251	T	0.269	42,510	40,577	1.06	(1.04–1.09)	1.5 × 10^−7^	1.04	(1.00–1.09)	0.0359
rs34460267	17	40,615,865	C	0.269	42,510	40,577	1.06	(1.04.1.09)	1.9 × 10^−7^	1.04	(1.00–1.09)	0.0424
rs34807589	17	40,624,656	T	0.264	42,510	40,577	1.06	(1.04–1.09)	2.0 × 10^−7^	1.04	(1.00–1.09)	0.0423
rs36005199	17	40,597,555	G	0.268	42,510	40,577	1.06	(1.04–1.09)	2.1 × 10^−7^	1.04	(1.00–1.09)	0.0490
rs12603201	17	40,595,927	T	0.581	42,510	40,577	0.95	(0.93–0.97)	3.1 × 10^−7^	0.97	(0.93–1.00)	0.0662
chr17:40607850:I	17	40,607,850	CT	0.055	42,510	40,577	1.13	(1.07–1.18)	7.0 × 10^−7^	1.09	(1.04–1.15)	0.0005
rs4796662	17	40,594,882	C	0.576	42,510	40,577	0.95	(0.93–0.97)	1.8 × 10^−6^	0.98	(0.94–1.01)	0.2217
rs34349578	17	40,598,129	A	0.195	42,510	40,577	1.07	(1.04–1.10)	2.1 × 10^−6^	1.04	(1.00–1.08)	0.0809
rs62075801	17	40,593,921	T	0.576	42,510	40,577	0.95	(0.93–0.97)	2.1 × 10^−6^	0.98	(0.94–1.01)	0.2385
rs12951640	17	40,594,298	A	0.253	42,510	40,577	1.06	(1.03–1.08)	2.1 × 10^−6^	1.03	(0.98–1.07)	0.2269
rs77942990	17	40,622,538	A	0.046	42,510	40,577	1.13	(1.07–1.19)	2.2 × 10^−6^	1.09	(1.04–1.15)	0.0007
rs35111218	17	40,595,572	T	0.252	42,510	40,577	1.06	(1.03–1.08)	2.3 × 10^−6^	1.03	(0.98–1.07)	0.2311
rs6503704	17	40,592,253	A	0.253	42,510	40,577	1.06	(1.03–1.08)	2.3 × 10^−6^	1.03	(0.98–1.07)	0.2413
rs12943498	17	40,593,901	C	0.253	42,510	40,577	1.06	(1.03–1.08)	2.5 × 10^−6^	1.02	(0.98–1.07)	0.2529
rs12951549	17	40,593,502	T	0.253	42,510	40,577	1.06	(1.03–1.08)	2.6 × 10^−6^	1.02	(0.98–1.07)	0.2537
chr17:40593802:I	17	40,593,802	GTTTC	0.251	42,510	40,577	1.06	(1.03–1.08)	3.5 × 10^−6^	1.02	(0.98–1.07)	0.2943
rs6503703	17	40,592,207	T	0.261	42,510	40,577	1.06	(1.03–1.08)	6.5 × 10^−6^	1.02	(0.98–1.06)	0.3775
chr17:40595896:D	17	40,595,896	C	0.211	42,510	40,577	1.06	(1.03–1.09)	9.0 × 10^−6^	1.02	(0.98–1.07)	0.2373

*SNP* single nucleotide polymorphism, *Chr*. chromosome, *OR* odds ratio, *CI* confidence interval, *STAT3* signal transducer and activator of transcription 3

^a^Build 37

^b^Allele frequency (AF) of counted allele

^c^OR per counted allele, adjusted for age, study and 16 European principal components

^d^Each SNP was tested adjusting for rs8074296, age, study and 16 European principal components. Estimate for rs8074296 was based on model including chr17:40607850:I

### Gene-level and pathway associations

Gene-level associations with risks of overall breast cancer, ER-positive and ER-negative diseases, respectively, for the 133 candidate genes in the immunosuppression pathway are summarized in Online Resource 17. *TGFBR2* and *CCND1* showed significant associations with overall breast cancer risk (*p* value <10^−6^ and 3.0 × 10^−4^, respectively). In addition, *IL5* and *GM*-*CSF* may be further potential susceptibility loci of breast cancer (*p* value = 1.0 × 10^−3^ and 7.0 × 10^−3^, respectively). *STAT3* showed a less significant association with overall breast cancer risk (*p* value = 0.033). The immunosuppression pathway as a whole yielded a significant association with overall breast cancer risk (*p* value <10^−6^). Similar gene-level and pathway associations were found for ER-positive but not for ER-negative breast cancer (Online Resource 17). We found significant enrichment of association in the immunosuppression pathway based on the results of the single SNPs association analyses (313 of 3595 tests significant at *α* = 0.05, exact binomial test *p* value = 2.2 × 10^−16^).

### Haplotype analyses

Despite the evidence for a possible role of *IL5* and *GM*-*CSF* in breast cancer susceptibility from the gene-level analysis, no individual SNPs at *IL5* or *GM*-*CSF* yielded significant genetic associations. To identify potential susceptibility haplotypes, haplotype-specific associations were assessed based on seven SNPs in or near *IL5* (rs4143832, rs2079103, rs2706399, rs743562, rs739719, rs2069812 and rs2244012) and nine SNPs in or near *GM*-*CSF* (rs11575022, rs2069616, rs25881, rs25882, rs25883, rs27349, rs27438, rs40401 and rs743564). The LD structures for these SNPs at *IL5* and *GM*-*CSF* are shown in Online Resource 18 and 19, respectively. In our study sample of women of European ancestry, 11 and 7 common haplotypes with frequency >1 % were observed at *IL5* and *GM*-*CSF*, respectively. The haplotype AAAACGG in *IL5* was associated with a decreased overall breast cancer risk (OR 0.96, 95 % CI 0.93–0.99, *p* value = 5.0 × 10^−3^, Table 5). In *GM*-*CSF*, the haplotype AAGAGCGAA was also associated with a decreased overall breast cancer risk **(**OR 0.92, 95 % CI 0.87–0.96, *p* value = 2.7 × 10^−4^, Table 6). The global *p* value for haplotype association was significant for both *IL5* (*p* value = 0.005) and *GM*-*CSF* (*p* value = 0.007).

**Table 5:** Haplotype associations with overall breast cancer risk for seven SNPs at *IL5* in women of European ancestry

Haplotype	rs4143832 (C>A)	rs2079103 (C>A)	rs2706399 (A>G)	rs743562 (G>A)	rs739719 (C>A)	rs2069812 (G>A)	rs2244012 (A>G)	Frequency	OR^a^ (95 %CI)	*p* value
Reference	C	C	G	G	C	G	A	0.42	1.00	–
1	C	C	A	A	C	A	A	0.22	1.01 (0.98–1.03)	0.62
2	A	A	A	A	C	G	G	0.14	0.96 (0.93–0.99)	0.005
3	C	C	G	G	C	G	G	0.04	1.02 (0.96–1.07)	0.55
4	C	A	A	G	A	A	A	0.04	0.99 (0.94–1.05)	0.85
5	A	A	A	A	C	G	A	0.03	0.96 (0.90–1.03)	0.24
6	C	C	G	G	C	A	A	0.02	0.95 (0.88–1.02)	0.15
7	C	C	A	A	C	G	A	0.02	1.09 (1.01–1.18)	0.021
8	C	A	A	G	A	G	A	0.02	0.92 (0.85–0.99)	0.035
9	C	C	A	A	C	G	G	0.01	0.92 (0.84–1.01)	0.078
Rare	–	–	–	–	–	–	–	0.03	1.01 (0.95–1.07)	0.84
Global^b^										0.005

*OR* odds ratio, *CI* confidence interval, *IL5* interleukin 5

^a^OR adjusted for age, study and nine European principal components

^b^Global *p* value for haplotype association, likelihood ratio test with ten degrees of freedom

**Table 6:** Haplotype associations with overall breast cancer risk for nine SNPs at *GM*-*CSF* in women of European ancestry

Haplotype	rs11575022 (A>C)	rs2069616 (A>G)	rs25881 (G>A)	rs25882 (A>G)	rs25883 (G>A)	rs27349 (C>A)	rs27438 (G>A)	rs40401 (G>A)	rs743564 (A>G)	Frequency	OR (95 %CI)^a^	*p* value
Reference	A	G	G	A	G	C	G	G	G	0.38	1.00	–
1	A	A	G	A	G	C	G	G	A	0.33	0.98 (0.96–1.00)	0.11
2	A	A	A	G	A	A	A	A	A	0.11	0.99 (0.96–1.02)	0.50
3	C	A	A	G	A	A	A	A	A	0.06	0.95 (0.91–0.99)	0.025
4	A	A	G	A	G	C	G	A	A	0.05	0.92 (0.87–0.96)	2.7 × 10^−4^
5	A	G	G	G	A	C	A	G	A	0.03	0.96 (0.91–1.03)	0.24
Rare	–	–	–	–	–	–	–	–	–	0.03	0.96 (0.91–1.02)	0.23
Global^b^												0.007

*OR* odds ratio, *CI* confidence interval, *GM-CSF* granulocyte–macrophage colony stimulating factor

^a^OR adjusted for age, study and nine European principal components

^b^Global *p* value for haplotype association, likelihood ratio test with 6 degrees of freedom

### Gene expression analyses

Using TCGA RNA sequencing level 3 data, we found that RNA expression levels of *STAT3* and *IL5* were significantly higher in 113 normal tissue samples compared to 989 breast tumor samples (*p* value = 1.3 × 10^−3^ and 7.0 × 10^−4^, respectively, Online Resources 20 and 21), while overall expression of *IL5* was low in both tissues. Also expression levels of *PTRF* were significantly higher in normal tissue compared to tumor tissue samples (*p* value ≤0.0001, Online Resource 22). *GM*-*CSF* expression was very low and did not differ between breast tumor samples and normal tissue samples (*p* value = 0.49, Online Resource 23). Among 183 mammary tissues in the GTEx database, SNPs rs1905339, rs8074296 and rs77942990 were not significantly correlated with *STAT3* (*p* values = 0.36, 0.36, and 0.2, respectively; Online Resource 24 to 26) or *PTRF* expression (*p* values = 0.4, 0.4, and 0.39 Online Resource 27 to 29). The SNPs rs1905339 and rs8074296 were significant eQTL for *TUBG2* (both *p* values = 9.9 × 10^−7^, Online Resource 30 and 31). The *STAT3/PTRF* variants rs146170568 and chr17:40607850:I were not available in the GTEx database.

## Discussion

Our comprehensive examination of associations between polymorphisms in the immunosuppression pathway genes and breast cancer risk revealed that *STAT3*, *IL5,* and *GM*-*CSF* may play a role in overall breast cancer susceptibility among women of European ancestry.

**Fig. 3:**
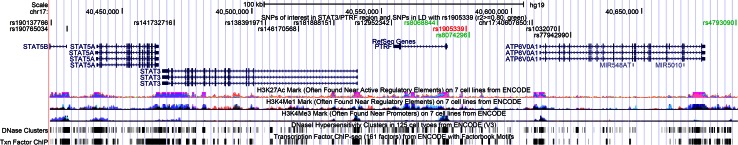
UCSC genome browser graphic for SNPs at the *STAT3/PTRF* region. The UCSC genome browser graphic shows functional annotations for the SNPs rs1905339 (*red*), correlated SNPs (*r* ^2^ > 0.80, *green*), as well as the other independent imputed SNPs (*black*) in or near the *STAT3/PTRF* region

The in silico functional analysis revealed that within a ±50 kb window of *STAT3*, several polymorphisms are located in regulatory regions that could actively affect DNA transcription (Fig. 3). The SNP rs181888151, which is in complete LD with rs146170568 (*r*^2^ = 1) but independent of rs1905339 (*r*^2^ = 0.01, Fig. 1) was significantly associated with increased risk for overall breast cancer (per allele OR 1.31, 95 % CI 1.16–1.49, *p* value = 2.8 × 10^−5^). Together with a further independently associated imputed SNP rs141732716, these polymorphisms reside in strong DNase I hypersensitivity and transcription regulatory sites (Fig. 3). This suggests that they may be functional polymorphisms, but further experimental work is required for confirmation.

*STAT3* encodes the signal transducer and activator of transcription 3, which is a member of the STAT protein family. Activated by corresponding cytokines or growth factors, STAT3 can be phosphorylated and translocate into the cell nucleus, acting as a transcription activator. In addition, STAT3 plays a key role in regulating immune response in the tumor microenvironment (Yu et al. 2009). STAT3 signaling is required for immunosuppressive and tumor-promoting functions of MDSCs (Cheng et al. 2003, 2008; Kortylewski et al. 2005, 2009; Kujawski et al. 2008; Ostrand-Rosenberg and Sinha 2009; Yu et al. 2009), as well as for Treg cell expansion (Kortylewski et al. 2005, 2009; Matsumura et al. 2007). *STAT3* has been reported in several previous genome-wide association studies (GWAS) to be associated with immune relevant diseases such as Crohn’s disease (Barrett et al. 2008; Franke et al. 2008; Yamazaki et al. 2013), inflammatory bowel disease (Jostins et al. 2012), and multiple sclerosis (Jakkula et al. 2010; Patsopoulos et al. 2011; Sawcer et al. 2011). Additionally, expression of *STAT3* was suggested to be enriched in triple-negative breast cancer, and negatively associated with lymph node involvement and breast tumor stage in a study based on an in silico network approach (Liu et al. 2012b). However, the association of rs1905339 with triple-negative breast cancer risk in our study (N triple-negative breast cancer = 2600) was similar and not stronger compared to the association observed for overall breast cancer risk (per allele OR 1.06, 95 % CI 0.99–1.14, *p* value = 0.11).

The genotyped SNP rs1905339 is also located at 7 kb 5′ of *PTRF,* which encodes the polymerase I and transcript release factor, and is not known to be directly involved in immunosuppression. In addition, two independently associated imputed SNPs rs8074296 and rs12952342 (*r*^2^ = 0.99 and 0 with rs1905339, respectively, Fig. 1) are located at 8 kb 5′ and 0.8 kb 3′ of *PTRF*, respectively (Fig. 3). PTRF is known to contribute to the formation of caveolae, small membrane caves involved in cell signaling, lipid regulation, and endocytosis (Chadda and Mayor 2008). Recently, down-regulation of *PTRF* was observed in breast cancer cell lines and breast tumor tissue, suggesting that PTRF expression might be an indicator for breast cancer progression (Bai et al. 2012). The SNPs rs1905339 and rs8074296 were also found to be eQTL for *TUBG2* (tubulin, gamma 2) in the GTEx database, the expression of *TUBG2* decreased with each variant allele (Online Resources 30 and 31, respectively). *TUBG2* encodes γ-tubulin, a protein required for the formation and polar orientation of microtubules in cells. It is currently unknown, whether *TUBG2* plays a role in breast cancer development or progression.

The other two potential susceptibility loci, *IL5* and *GM*-*CSF,* are both located in a known cytokine gene cluster at 5q31. *IL5* encodes interleukin 5, a cytokine secreted by CD4+ T helper 2 cells (Mills 2004; Parker 1993). IL5 is a growth and differentiation factor for both B cells and eosinophils, triggering eosinophil- and B cell-dependent immune response (Mills 2004; Parker 1993). *GM*-*CSF* encodes granulocyte–macrophage colony stimulating factor, a cytokine that controls differentiation and function of granulocytes and macrophages. GM-CSF is also a MDSC- inducing and activating factor in the bone marrow (Ostrand-Rosenberg and Sinha 2009; Serafini et al. 2004). In the tumor microenvironment, GM-CSF is the cytokine for dendritic cell differentiation and function, and it is often found to be underexpressed (Zou 2005). Additionally, 5q31 has been found to be a susceptibility locus for rheumatoid arthritis (Okada et al. 2012, 2014) and inflammatory bowel disease (Jostins et al. 2012).

Immunosuppression is a complex network with plenty of contributors, including transcription factors (e.g., STAT3), as well as immune mediating cytokines (e.g., IL5 and GM-CSF). Results of this analysis indicate that genetic variation in different components of the immunosuppression pathway may be susceptibility loci of breast cancer among women of European ancestry.

The main strengths of the present analysis were its large sample size, the uniform genotyping procedures and centralized quality controls used. The imputation of genotypes in the most interesting susceptibility loci provided an opportunity to identify more strongly associated variants. Assessments of gene-level associations also provided evidence for additional putative susceptibility loci. A limitation was the lack of an independent sample to replicate the observed associations; this will be feasible in the future using new studies participating in the BCAC. Further functional studies are still needed to identify causal variants and to investigate the underlying biological mechanisms.

## Conclusions

Overall, our data provide strong evidence that common variation in the immunosuppression pathway is associated with breast cancer susceptibility. The strongest candidates for mediating this association were *STAT3*, *IL5,* and *GM*-*CSF*, but we cannot exclude the possibility of multiple alleles each with effects too small to confirm.

## Electronic supplementary material


**ESM_1_Description_studies.pdf** Description of 37 Breast Cancer Association Consortium studies included in this analysis

**ESM_2_List_genes.pdf** List of 133 candidate genes relevant to the immunosuppression pathway by chromosomal position

**ESM_3_QQPlot.tif** Quantile–quantile plot for genotyped SNPs included in this analysis for associations with overall breast cancer risk (excluding SNPs located within *TGFBR2* and *CCND1*)

**ESM_4_Association_SNPs.pdf** Associations with overall breast cancer risk for 3595 SNPs in the immunosuppression pathway genes

**ESM_5_TopSNPs_ERstatus.pdf** Associations of *TGFBR2*, *CCND1* and *STAT3* SNPs with overall breast cancer risk as well as stratified by ER status

**ESM_6_LDplot_PTRF.tif** Linkage disequilibrium plot for 19 SNPs at *PTRF*

**ESM_7_ForestPlot_rs1905339.tif** Forest plot showing meta-analysis of study-wise estimates for the association of rs1905339 with breast cancer risk

**ESM_8_ForestPlot_rs8074296.tif** Forest plot showing meta-analysis of study-wise estimates for the association of rs8074296 with breast cancer risk

**ESM_9_ForestPlot_rs146170568.tif** Forest plot showing meta-analysis of study-wise estimates for the association of rs146170568 with breast cancer risk

**ESM_10_ForestPlot_rs141732716.tif** Forest plot showing meta-analysis of study-wise estimates for the association of rs141732716 with breast cancer risk

**ESM_11_ForestPlot_rs138391971.tif** Forest plot showing meta-analysis of study-wise estimates for the association of rs138391971 with breast cancer risk

**ESM_12_ForestPlot_rs12952342.tif** Forest plot showing meta-analysis of study-wise estimates for the association of rs12952342 with breast cancer risk

**ESM_13_ForestPlot_rs190765034.tif** Forest plot showing meta-analysis of study-wise estimates for the association of rs190765034 with breast cancer risk

**ESM_14_ForestPlot_rs190137766.tif** Forest plot showing meta-analysis of study-wise estimates for the association of rs190137766 with breast cancer risk

**ESM_15_ForestPlot_chr17_40607850_I.tif** Forest plot showing meta-analysis of study-wise estimates for the association of chr17:40607850:I with breast cancer risk

**ESM_16_ForestPlot_rs77942990.tif** Forest plot showing meta-analysis of study-wise estimates for the association of rs77942990 with breast cancer risk

**ESM_17_Gene_level_associations.pdf** Gene-level associations with breast cancer risk for 133 candidate genes in the immunosuppression pathway

**ESM_18_LDplot_IL5.tif** Linkage disequilibrium plot for seven SNPs at *IL5*

**ESM_19_LDplot_GM-CSF.tif** Linkage disequilibrium plot for nine SNPs at *GM*-*CSF*

**ESM_20_Boxplot_STAT3.tif** Box plot showing gene expression levels of *STAT3* in normal breast tissue as well as tumor breast tissue

**ESM_21_Boxplot_IL5.tif** Box plot showing gene expression levels of *IL5* in normal breast tissue as well as tumor breast tissue

**ESM_22_Boxplot_PTRF.tif** Box plot showing gene expression levels of *PTRF* in normal breast tissue as well as tumor breast tissue

**ESM_23_Boxplot_CSF2.tif** Box plot showing gene expression levels of *GM*-*CSF* in normal breast tissue as well as tumor breast tissue

**ESM_24_eQTL_rs1905339_STAT3.tif** Associations of rs1905339 genotypes with *STAT3* expression within 183 breast tissue samples

**ESM_25_eQTL_rs8074296_STAT3.tif** Associations of rs8074296 genotypes with *STAT3* expression within 183 breast tissue samples

**ESM_26_eQTL_rs77942990_STAT3.tif** Associations of rs8074296 genotypes with *STAT3* expression within 183 breast tissue samples

**ESM_27_eQTL_rs1905339_PTRF.tif** Associations of rs1905339 genotypes with *PTRF* expression within 183 breast tissue samples

**ESM_28_eQTL_rs8074296_PTRF.tif** Associations of rs8074296 genotypes with *PTRF* expression within 183 breast tissue samples

**ESM_29_eQTL_rs77942990_PTRF.tif** Associations of rs77942990 genotypes with *PTRF* expression within 183 breast tissue samples

**ESM_30_eQTL_rs1905339_TUBG2.tif** Associations of rs1905339 genotypes with *TUBG2* expression within 183 breast tissue samples

**ESM_31_eQTL_rs8074296_ TUBG2.tif** Associations of rs8074296 genotypes with *TUBG2* expression within 183 breast tissue samples

## Abbreviations

BCAC: Breast Cancer Association Consortium
CCND1: Cyclin D1
CI: Confidence interval
COGS: Collaborative Oncological Gene-Environment Study
DNA: Deoxyribonucleic acid
GM-CSF: Granulocyte-macrophage colony stimulating factor
EM: Estimation maximization
ENCODE: Encyclopedia of DNA elements
eQTL: Expression quantitative trait loci
ER: Estrogen receptor
GWAS: Genome-wide association study
HWE: Hardy–Weinberg equilibrium
IL5: Interleukin 5
LD: Linkage disequilibrium
MAF: Minor allele frequency
MDSCs: Myeloid-derived suppressor cells
OR: Odds ratio
PCs: Principal components
PTRF: Polymerase I and transcript release factor
QQ: Quantile–quantile
RSEM: RNA-Seq by expectation-maximization
SD: Standard deviation
SNPs: Single nucleotide polymorphisms
STAT3: Signal transducer and activator of transcription 3
TCGA: The Cancer Genome Atlas
TGFBR2: Transforming growth factor beta receptor II
Treg cells: Regulatory T cells
TUBG2: Tubulin, gamma 2
