# Supplementary material for: Genetic variation in the immunosuppression pathway genes and breast cancer susceptibility: a pooled analysis of 42,510 cases and 40,577 controls from the Breast Cancer Association Consortium
Source: Hum Genet. 2015 Nov 30;135:137–54. doi: 10.1007/s00439-015-1616-8 (PMC4698282; doi:10.1007/s00439-015-1616-8)
Supplement: Supplementary file 1 — ESM_1_Description_studies.pdf Description of 37 Breast Cancer Association Consortium studies included in this analysis [file 439_2015_1616_MOESM1_ESM.pdf]

Table S1 Description of 37 Breast Cancer Association Consortium studies included in this analysis.

Study Acronym	Study Name	Country	Study Design	N Cases	Age (mean, years)	N Controls	Age (mean, years)
ABCFS	Australian Breast Cancer Family Study	Australia	Population-based case-control study	790	39.5	551	41.9
ABCS	Amsterdam Breast Cancer Study	Netherlands	Hospital-based consecutive cases; population-based controls	1,941	42.8	1,557	47.3
BBCC	Bavarian Breast Cancer Cases and Controls	Germany	Hospital based cases; population based controls	554	60.7	458	57.1
BBCS	British Breast Cancer Study	UK	Cancer registry and National Cancer Research network (NCRN) based cases; population based controls	1,445	52.7	1,329	45.1
BIGGS	Breast Cancer in Galway Genetic Study	Ireland	Hospital based cases; population based controls	779	53.3	49	65.6
BSUCH	Breast Cancer Study of the University of Heidelberg	Germany	Hospital based cases; healthy blood donator controls	814	55.3	954	57.3
CECILE	CECILE Breast Cancer Study	France	Population-based case-control study	900	54.4	999	54.7
CGPS	Copenhagen General Population Study	Denmark	Population-based case-control study	2,811	61.8	4,534	58.2
CNIO-BCS	Spanish National Cancer Centre Breast Cancer Study	Spain	Case-control study	704	54.2	834	50.0
CTS	California Teachers Study	USA	Prospective cohort study: nested case-control	51	62.9	44	55.6
ESTHER	ESTHER Breast Cancer Study	Germany	Population-based case-control study	471	60.6	502	62.3
GC-HBOC	German Consortium for Hereditary Breast & Ovarian Cancer	Germany	Population-based familial case-control study	0	0.0	139	57.7
GENICA	Gene Environment Interaction and Breast Cancer in Germany	Germany	Population-based case-control study	465	57.0	427	57.3
HEBCS	Helsinki Breast Cancer Study	Finland	Hospital-based case-control study + additional familial cases	1,517	56.8	1,234	41.0
HMBCS	Hannover-Minsk Breast Cancer Study	Belarus	Hospital based cases; population based controls	688	49.0	130	41.9
KBCP	Kuopio Breast Cancer Project	Finland	Population-based prospective clinical cohort	411	58.9	251	52.9
LMBC	Leuven Multidisciplinary Breast Centre	Belgium	Hospital-based case-control study	2,522	56.6	1,386	44.0
MARIE	Mammary Carcinoma Risk Factor Investigation	Germany	Population-based case-control study	1,656	62.3	1,778	61.8

Study Acronym	Study Name	Country	Study Design	N Cases	Age (mean, years)	N Controls	Age (mean, years)
MBCSG	Milan Breast Cancer Study Group	Italy	Clinic-based recruitment of familial/early onset breast cancer patients (cases); population-based controls	189	42.0	400	42.3
MCBCS	Mayo Clinic Breast Cancer Study	USA	Hospital-based case-control study	1,546	57.0	1,931	56.6
MCCS	Melbourne Collaborative Cohort Study	Australia	Population-based prospective cohort study	614	60.8	511	56.3
MEC	Multi-ethnic Cohort	USA	Prospective cohort study: nested case-control study	703	66.4	741	59.8
MTLGEBCS	Montreal Gene-Environment Breast Cancer Study	Canada	Population-based case-control study	489	61.8	436	61.6
NBCS	Norwegian Breast Cancer Study	Norway	Hospital-based case-control study	828	60.1	214	51.0
NBHS	Nashville Breast Health Study	USA	Population-based case-control study	125	52.6	118	52.6
OBCS	Oulu Breast Cancer Study	Finland	Hospital-based case-control study	500	57.5	414	42.4
OFBCR	Ontario Familial Breast Cancer Registry	Canada	Population-based familial case-control study	1,157	53.0	511	51.9
PBCS	NCI Polish Breast Cancer Study	Poland	Population-based case-control study	519	56.3	424	56.3
SASBAC	Singapore and Sweden Breast Cancer Study	Sweden	Population-based case-control study	1,163	63.0	1,378	63.3
SBCS	Sheffield Breast Cancer Study	UK	Hospital-based case-control study	751	59.5	848	57.5
SEARCH	Study of Epidemiology and Risk factors in Cancer Heredity	UK	Population-based case-control study	9,095	54.4	8,064	57.8
SKKDKFZS	Städtisches Klinikum Karlsruhe Deutsches Krebsforschungszentrum Study	Germany	Hospital-based breast cancer cohort	134	58.9	29	60.7
SZBCS	IHCC-Szczecin Breast Cancer Study	Poland	Hospital based case-control study	303	55.7	315	57.4
TNBCC	Triple Negative Breast Cancer Study	USA and Greece	Hospital-based case-control study	499	52.9	152	55.4
UKBGS	UK Breakthrough Generations Study	UK	Prospective cohort study: nested case-control study of women who had had breast cancer prior to entry into the cohort	413	56.3	470	54.2
kConFab/AOCS	Kathleen Cuningham Foundation Consortium for research into Familial Breast Cancer/Australian Ovarian Cancer Study	Australia and New Zeland	Clinic-based recruitment of familial breast cancer patients (cases); population-based case-control study of ovarian cancer (controls only)	410	45.7	897	57.8
pKARMA	Karolinska Mammography Project for Risk Prediction of Breast Cancer - prevalent cases	Sweden	Case-control study	4,553	58.1	5,568	53.4
