# Supplementary material for: Genetic variation in the immunosuppression pathway genes and breast cancer susceptibility: a pooled analysis of 42,510 cases and 40,577 controls from the Breast Cancer Association Consortium
Source: Hum Genet. 2015 Nov 30;135:137–54. doi: 10.1007/s00439-015-1616-8 (PMC4698282; doi:10.1007/s00439-015-1616-8)
Supplement: Supplementary file 2 — ESM_2_List_genes.pdf List of 133 candidate genes relevant to the immunosuppression pathway by chromosomal position [file 439_2015_1616_MOESM2_ESM.pdf]

Table S2 List of 133 candidate genes relevant to the immunosuppression pathway by chromosomal position.

Gene	Entrez Id	Chr	Location	LowPoint*	HighPoint*	Description	N SNPs*
FASLG	356	1	1q23	172628185	172636013	Fas ligand	18
CD2	914	1	1p13.1	117297057	117311851	CD2 molecule	3
CSF3R	1441	1	1p35-p34.3	36931644	36948915	colony stimulating factor 3 receptor	10
EPHB2	2048	1	1p36.1-p35	23037263	23241823	ephrin(EPH) receptor B2	22
mTOR	2475	1	1p36.2	11166588	11322614	mechanistic target of rapamycin (serine/threonine kinase)	18
IL6R	3570	1	1q21	154377669	154441926	interleukin 6 receptor	35
IL10	3586	1	1q31-q32	206940948	206945839	interleukin 10	0
IL12RB2	3595	1	1p31.3-p31.2	67773047	67862583	interleukin 12 receptor, beta 2	14
COX2	5743	1	1q25.2-q25.3	186640944	186649559	also known as PTGS2: prostaglandin-endoperoxide synthase 2	15
CD45R	5788	1	1q31-q32	198608098	198726605	also known as PTPRC: protein tyrosine phosphatase, receptor type, C	14
S100A8	6279	1	1q21	153362508	153395054	S100 calcium binding protein A8	8
CD62L	6402	1	1q23-q25	169659806	169680843	CD62 ligand	10
TGFB2	7042	1	1q41	218518676	218617961	transforming growth factor, beta 2	58
TGFBR3	7049	1	1p33-p32	92145900	92371559	transforming growth factor, beta receptor III	301
TNFRSF1B	7133	1	1p36.22	12227044	12269279	tumor necrosis factor receptor superfamily, member 1B	26
TNFSF4	7292	1	1q25	173152870	173176471	tumor necrosis factor (ligand) superfamily, member 4	14
GITR	8784	1	1p36.3	1138888	1142163	also known as TNFRSF18: tumor necrosis factor receptor superfamily, member 18	9
MAPKAPK2	9261	1	1q32	206858295	206907630	mitogen-activated protein kinase-activated protein kinase 2	4
IL19	29949	1	1q32.2	206972215	207016326	interleukin 19	26
B7-H4	79679	1	1p13.1	117686209	117753582	also known as VTCN1: V-set domain containing T cell activation inhibitor 1	2
FCRL3	115352	1	1q21-q22	157646271	157670775	Fc receptor-like 3	17
IL23R	149233	1	1p31.3	67604590	67725662	interleukin 23 receptor	60
CD28	940	2	2q33	204571198	204603635	CD28 molecule	8
CTLA4	1493	2	2q33	204732511	204738683	cytotoxic T-lymphocyte-associated protein 4	12
IL1B	3553	2	2q14	113587337	113594356	interleukin 1, beta	17
CXCR1	3577	2	2q35	219027568	219031716	chemokine (C-X-C motif) receptor 1	1
IL8RA	3579	2	2q35	218990013	219001976	interleukin 8 receptor alpha	14
CD49d	3676	2	2q31.3	182321619	182402474	also known as ITGA4: integrin, alpha 4	13
PD1	5133	2	2q37.3	242792033	242801058	programmed cell death 1	4
STAT1	6772	2	2q32.2	191833762	191878976	signal transducer and activator of transcription 1	37
ADAM17	6868	2	2p25	9629392	9695917	ADAM metallopeptidase domain 17	10
CXCR4	7852	2	2q21	136871919	136875725	chemokine (C-X-C motif) receptor 4	2
IL18R1	8809	2	2q12	102979093	103015230	interleukin 18 receptor 1	6

Gene	Entrez Id	Chr	Location	LowPoint*	HighPoint*	Description	N SNPs*
PSME4	23198	2	2p16.2	54091204	54197977	proteasome (prosome, macropain) activator subunit 4	71
ICOS	29851	2	2q33	204801454	204826302	inducible T-cell co-stimulator	5
CD80	941	3	3q13.3-q21	119243140	119278481	CD80 molecule	11
CD86	942	3	3q21	121774209	121839990	CD86 molecule	14
CCR4	1233	3	3p24	32993066	32996403	chemokine (C-C motif) receptor 4	1
IL5RA	3568	3	3p26-p24	3108008	3152058	interleukin 5 receptor, alpha	62
IL12A	3592	3	3q25.33	159706623	159713806	interleukin 12A	11
MYD88	4615	3	3p22	38179969	38184513	myeloid differentiation primary response 88	2
TGFBR2	7048	3	3p22	30647994	30735634	transforming growth factor, beta receptor II	128
CCR9	10803	3	3p21.3	45927996	45944667	chemokine (C-C motif) receptor 9	19
EIF2A	83939	3	3q25.1	150264465	150303803	eukaryotic translation initiation factor 2A	11
CD38	952	4	4p15	15779921	15850706	CD38 molecule	9
IL2	3558	4	4q26-q27	123372625	123377650	interleukin 2	13
IL8	3576	4	4q13-q21	74606223	74609433	interleukin 8	4
IL15	3600	4	4q31	142557749	142655140	interleukin 15	13
CXCL10	3627	4	4q21	76942269	76944689	chemokine (C-X-C motif) ligand 10	1
GM-CSF	1437	5	5q31.1	131409485	131411863	granulocyte-macrophage colony stimulating factor	9
GZMA	3001	5	5q11-q12	54398474	54406080	granzyme A	4
HSP70	3308	5	5q31.1	132387662	132440709	heat shock 70kDa protein 4	4
IL4	3565	5	5q31.1	132009678	132018370	interleukin 4	22
IL5	3567	5	5q31.1	131877136	131892555	interleukin 5	7
IL7R	3575	5	5p13	35856977	35879705	interleukin 7 receptor	51
IL12B	3593	5	5q31.1-q33.1	158741791	158757481	interleukin 12B	28
IL13	3596	5	5q31	131993865	131996801	interleukin 13	1
RAGE	177	6	6p21.3	32148745	32152099	AGER:advanced glycosylation end product-specific receptor	16
ARG1	383	6	6q23	131894344	131905472	arginase-1	9
CCND3	896	6	6p21	41902671	42016610	cyclin D3	13
CCR6	1235	6	6q27	167525295	167554168	chemokine (C-C motif) receptor 6	10
HLA-DRA	3122	6	6p21.3	32407619	32412823	major histocompatibility complex, class II, DR alpha	23
CD73	4907	6	6q14-q21	86159302	86205509	CD73 molecule	5
VEGF	7422	6	6p12	43737946	43754224	vascular endothelial growth factor A	44
IL6	3569	7	7p21	22766766	22771621	interleukin 6	41
HDAC9	9734	7	7p21.1	18126572	19039135	histone deacetylase 9	416
IL7	3574	8	8q12-q13	79645007	79717758	interleukin 7	45

Gene	Entrez Id	Chr	Location	LowPoint*	HighPoint*	Description	N SNPs*
INDO	3620	8	8p12-p11	39771328	39786309	indoleamine 2,3-dioxygenase 1	10
MYC	4609	8	8q24.21	128748315	128753680	v-myc avian myelocytomatosis viral oncogene homolog	49
IFNA1	3439	9	9p22	21440453	21441315	interferon, alpha 1	4
TGFBR1	7046	9	9q22	101867412	101916474	transforming growth factor, beta receptor 1	42
CD274	29126	9	9p24	5450503	5470567	CD274 molecule	47
CD95	355	10	10q24.1	90750288	90775542	CD95 molecule	35
CD39	953	10	10q24	97471536	97637023	CD39 molecule	4
MAP3K8	1326	10	10p11.23	30722950	30750762	mitogen-activated protein kinase kinase kinase 8	11
IL2RA	3559	10	10p15-p14	6052657	6104333	interleukin 2 receptor, alpha	172
PRF1	5551	10	10q22	72357104	72362531	perforin 1 (pore forming protein)	3
PRKCQ	5588	10	10p15	6435558	6622254	protein kinase C, theta	170
CXCL12	6387	10	10q11.1	44865601	44880545	chemokine (C-X-C motif) ligand 12	19
CD304	8829	10	10p12	33466419	33623833	CD304 molecule	42
CCND1	595	11	11q13	69455873	69469242	cyclin D1	83
CD3D	915	11	11q23	118209789	118213459	CD3d molecule, delta	0
CD3E	916	11	11q23	118175295	118186890	CD3e molecule, epsilon (CD3-TCR complex)	4
CD3G	917	11	11q23	118215032	118224497	CD3g molecule, gamma (CD3-TCR complex)	16
GARP	2615	11	11q13.5-q14	76368568	76381990	glycoprotein A repetitions predominant	9
IL10RA	3587	11	11q23	117857106	117872198	interleukin 10 receptor, alpha	20
MMP1	4312	11	11q22.3	102660641	102668966	matrix metallopeptidase 1 (interstitial collagenase)	4
MMP3	4314	11	11q22.3	102706528	102714342	matrix metallopeptidase 3 (stromelysin 1, progelatinase)	27
MMP12	4321	11	11q22.3	102733464	102745764	matrix metallopeptidase 12 (macrophage elastase)	29
CD4	920	12	12p13.31	6898638	6929976	CD4 molecule	1
CD27	939	12	12p13	6554051	6560884	CD27 molecule	13
CD69	969	12	12p13	9905082	9913497	CD69 molecule	5
LAG3	3902	12	12p13.32	6881670	6887621	lymphocyte-activation gene 3	6
KITLG	4254	12	12q22	88886570	88974250	KIT ligand	76
STAT6	6778	12	12q13	57489187	57505196	signal transducer and activator of transcription 6	1
IL23A	51561	12	12q13.3	56732663	56734194	interleukin 23, alpha subunit p19	1
FLT3	2322	13	13q12	28577411	28682904	fms-related tyrosine kinase 3	83
TRANCE	8600	13	13q14	43136872	43182149	also known as TNFSF11:tumor necrosis factor (ligand) superfamily, member 11	22
AKT	207	14	14q32.32	105235686	105262080	also known as AKT1: v-akt murine thymoma viral oncogene homolog 1	38
GZMB	3002	14	14q11.2	25100160	25103490	granzyme B	12
HIF1a	3091	14	14q23.2	62162118	62214977	hypoxia inducible factor 1, alpha subunit	14

Gene	Entrez Id	Chr	Location	LowPoint*	HighPoint*	Description	N SNPs*
TGFB3	7043	14	14q24	76424440	76449334	transforming growth factor, beta 3	22
EIF2AK4	440275	15	15q15.1	40226331	40327797	eukaryotic translation initiation factor 2 alpha kinase 4	72
IRF8	3394	16	16q24.1	85932774	85956212	interferon regulatory factor 8	56
IL4R	3566	16	16p12.1-p11.2	27325161	27376099	interleukin 4 receptor	46
BIRC5	332	17	17q25	76210277	76221716	baculoviral IAP repeat containing 5	22
CCR7	1236	17	17q12-q21.2	38710021	38721736	chemokine (C-C motif) receptor 7	1
CSF3	1440	17	17q11.2-q12	38171614	38174066	colony stimulating factor 3	12
CD103	3682	17	17p13	3617919	3704537	CD103 molecule	7
LGALS9	3965	17	17q11.2	25958174	25976586	lectin, galactoside-binding, soluble, 9	15
iNOS	4843	17	17q11.2-q12	26083792	26127555	nitric oxide synthase 2, inducible	32
CD31	5175	17	17q23.3	62396775	62404856	CD31 molecule	7
STAT3	6774	17	17q21.31	40465342	40540586	signal transducer and activator of transcription 3	48
STAT5A	6776	17	17q11.2	40439565	40463961	signal transducer and activator of transcription 5A	0
STAT5B	6777	17	17q11.2	40351195	40428478	signal transducer and activator of transcription 5B	7
TNFRSF11A	8792	18	18q22.1	59992520	60054943	tumor necrosis factor receptor superfamily, member 11a	57
GAL10	1178	19	19q13.1	40221893	40228669	also known as CLC: Charcot-Leyden crystal galectin	8
HuR	1994	19	19p13.2	8023457	8070529	Hu antigen R	9
TGFB1	7040	19	19q13.1	41836812	41859831	transforming growth factor, beta 1	0
TNFSF14	8740	19	19p13.3	6661264	6670599	tumor necrosis factor (ligand) superfamily, member 14	14
B9D2	80776	19	19q13.2	41860322	41870078	B9 protein domain 2	8
BCL-XL	598	20	20q11.21	30252261	30311752	also known as BCL2L1: BCL2-like 1	12
CD40	958	20	20q12-q13.2	44746899	44758384	CD40 molecule	15
MMP9	4318	20	20q11.2-q13.1	44637547	44645200	matrix metallopeptidase 9 (gelatinase B, 92kDa gelatinase, 92kDa type IV collagenase)	24
IL10RB	3588	21	21q22.11	34638665	34669539	interleukin 10 receptor, beta	14
LGALS1	3956	22	22q13.1	38071613	38075809	lectin, galactoside-binding, soluble, 1	5
ERK2	5594	22	22q11.21	22113946	22221970	extracellular signal-regulated kinase 2	30
IL17RA	23765	22	22q11.1	17565849	17596584	interleukin 17 receptor A	26
CD40L	959	X	Xq26	135730281	135742549	CD40 ligand	5
NOX2	1536	X	Xp21.1	37639266	37672718	also known as CYBB: cytochrome b-245, beta polypeptide	3
IL13RA2	3598	X	Xq13.1-q28	114238538	114252207	interleukin 13 receptor, alpha 2	7
IRAK1	3654	X	Xq28	153275957	153285342	interleukin-1 receptor-associated kinase 1	4
FOXP3	50943	X	Xp11.23	49106897	49122200	forkhead box P3	4

*Based on Genome Reference Consortium Human Build 37 patch release 13 (GRCh37.p13). Number of SNPs refers to SNPs within ±50kb of each gene after quality controls.

Chr = chromosome, SNP = Single nucleotide polymorphism
