# Supplementary figures and images for: Genetic variation in the immunosuppression pathway genes and breast cancer susceptibility: a pooled analysis of 42,510 cases and 40,577 controls from the Breast Cancer Association Consortium
Source: Hum Genet. 2015 Nov 30;135:137–54. doi: 10.1007/s00439-015-1616-8 (PMC4698282; doi:10.1007/s00439-015-1616-8)

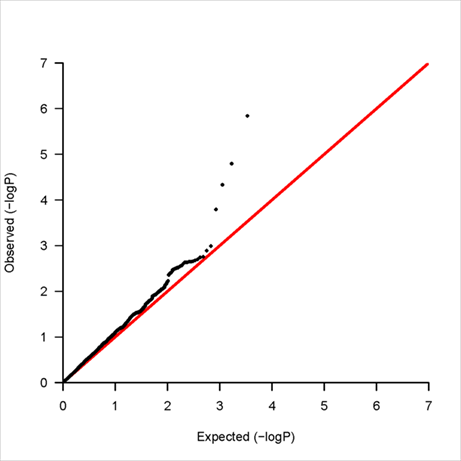

Supplement: Supplementary file 3 — ESM_3_QQPlot.tif Quantile–quantile plot for genotyped SNPs included in this analysis for associations with overall breast cancer risk (excluding SNPs located within TGFBR2 and CCND1) [file 439_2015_1616_MOESM3_ESM.tif]

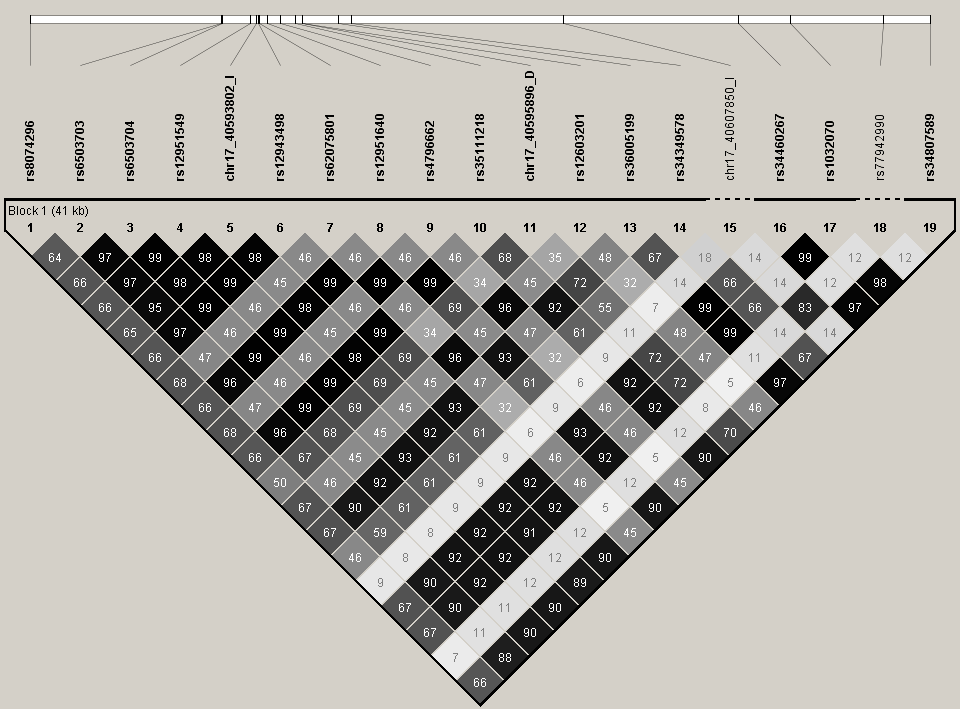

Supplement: Supplementary file 6 — ESM_6_LDplot_PTRF.tif Linkage disequilibrium plot for 19 SNPs at PTRF [file 439_2015_1616_MOESM6_ESM.tif]

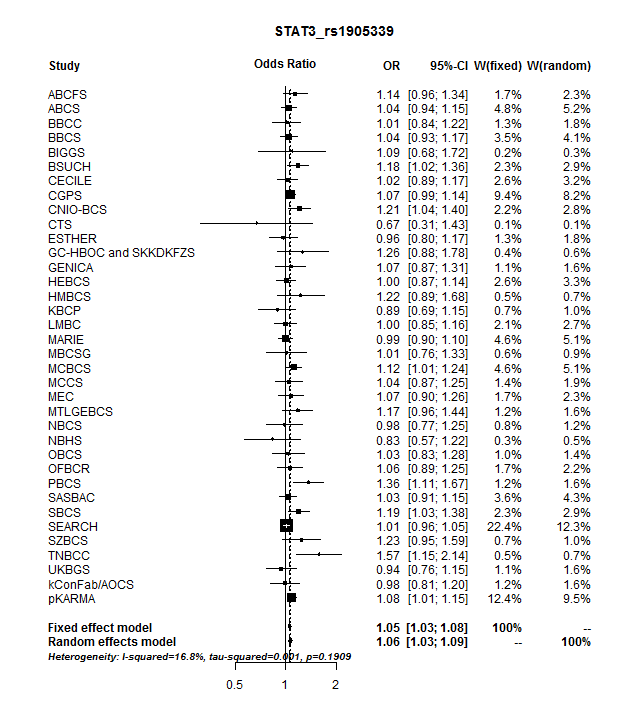

Supplement: Supplementary file 7 — ESM_7_ForestPlot_rs1905339.tif Forest plot showing meta-analysis of study-wise estimates for the association of rs1905339 with breast cancer risk [file 439_2015_1616_MOESM7_ESM.tif]

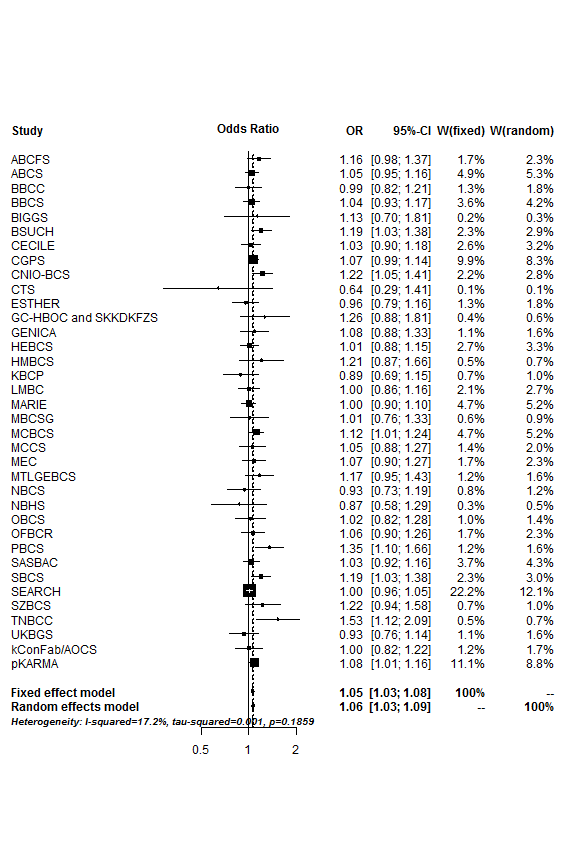

Supplement: Supplementary file 8 — ESM_8_ForestPlot_rs8074296.tif Forest plot showing meta-analysis of study-wise estimates for the association of rs8074296 with breast cancer risk [file 439_2015_1616_MOESM8_ESM.tif]

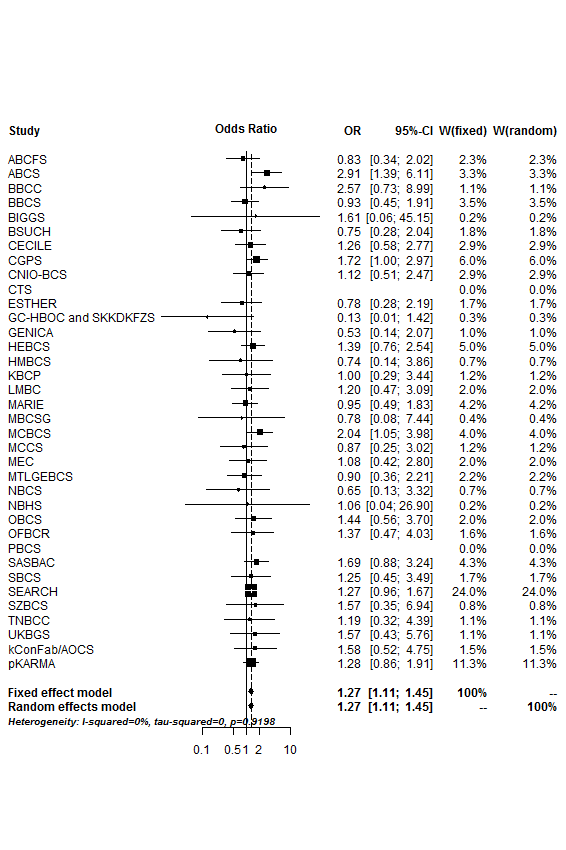

Supplement: Supplementary file 9 — ESM_9_ForestPlot_rs146170568.tif Forest plot showing meta-analysis of study-wise estimates for the association of rs146170568 with breast cancer risk [file 439_2015_1616_MOESM9_ESM.tif]

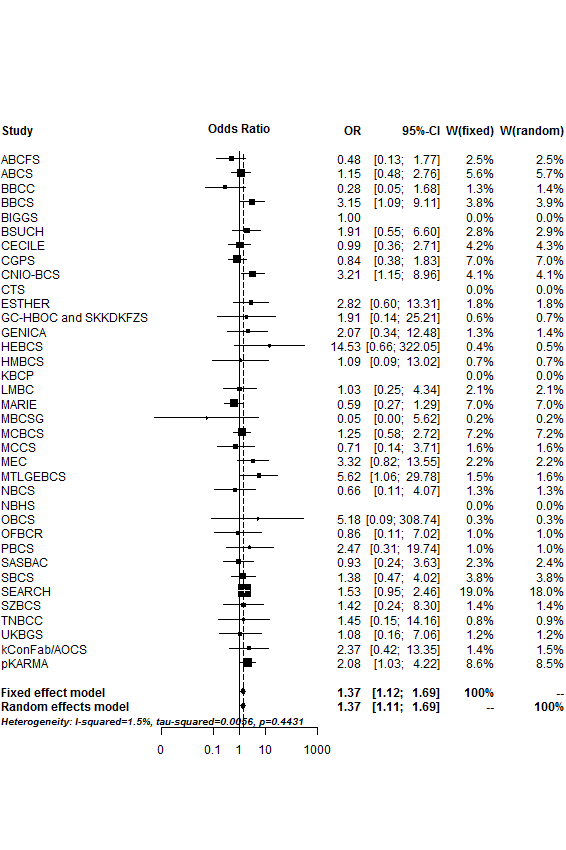

Supplement: Supplementary file 10 — ESM_10_ForestPlot_rs141732716.tif Forest plot showing meta-analysis of study-wise estimates for the association of rs141732716 with breast cancer risk [file 439_2015_1616_MOESM10_ESM.tif]

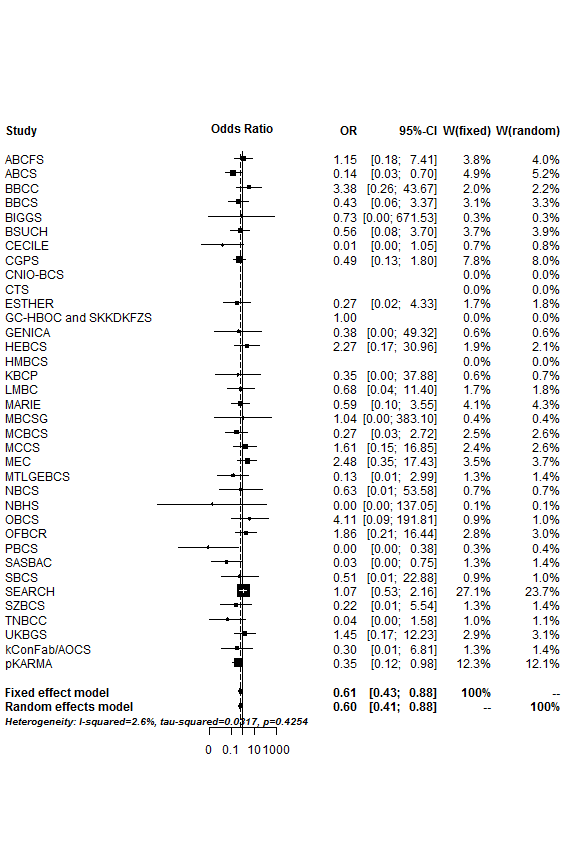

Supplement: Supplementary file 11 — ESM_11_ForestPlot_rs138391971.tif Forest plot showing meta-analysis of study-wise estimates for the association of rs138391971 with breast cancer risk [file 439_2015_1616_MOESM11_ESM.tif]

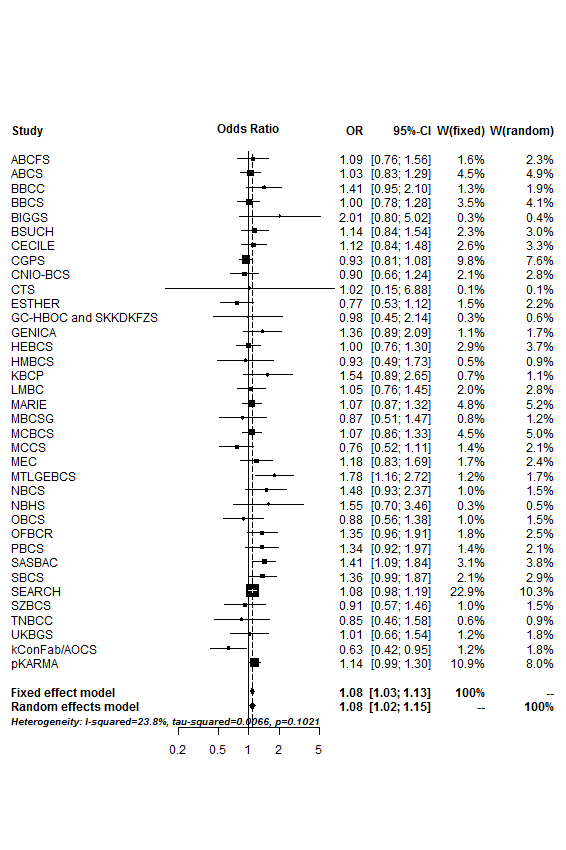

Supplement: Supplementary file 12 — ESM_12_ForestPlot_rs12952342.tif Forest plot showing meta-analysis of study-wise estimates for the association of rs12952342 with breast cancer risk [file 439_2015_1616_MOESM12_ESM.tif]

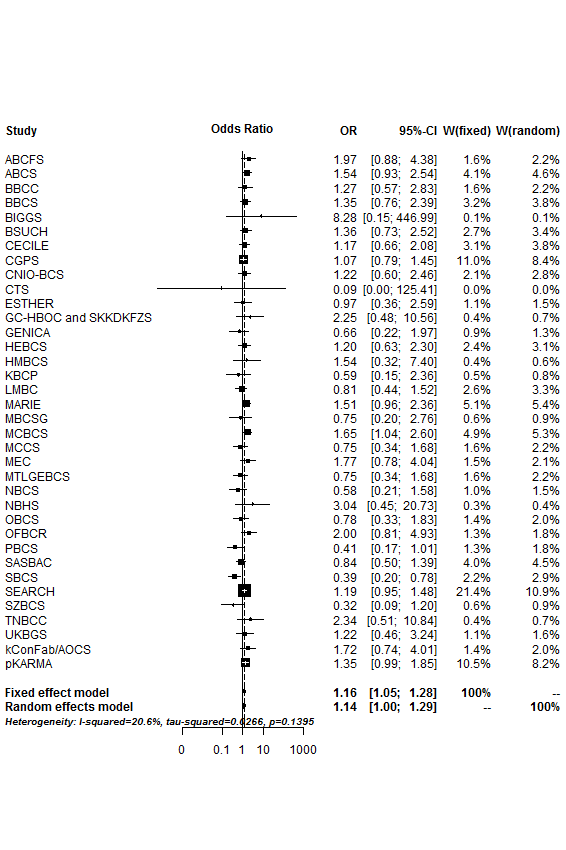

Supplement: Supplementary file 13 — ESM_13_ForestPlot_rs190765034.tif Forest plot showing meta-analysis of study-wise estimates for the association of rs190765034 with breast cancer risk [file 439_2015_1616_MOESM13_ESM.tif]

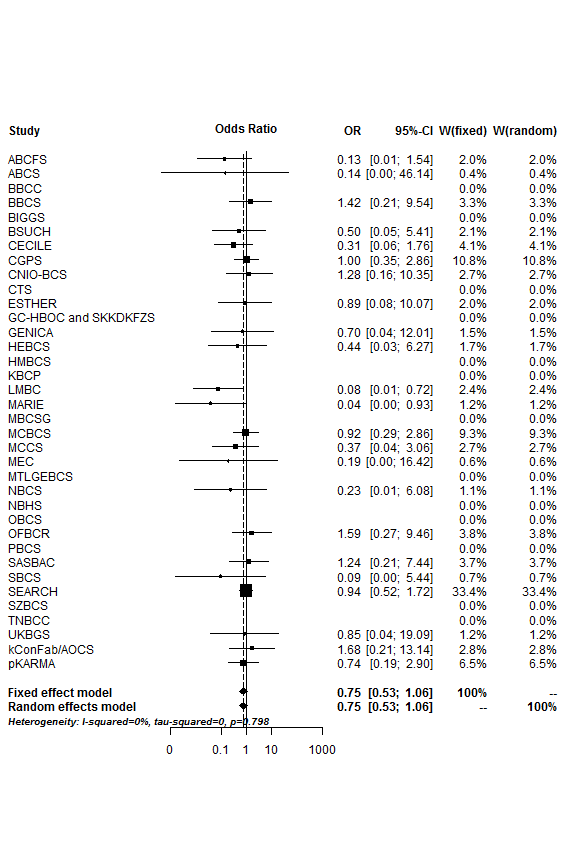

Supplement: Supplementary file 14 — ESM_14_ForestPlot_rs190137766.tif Forest plot showing meta-analysis of study-wise estimates for the association of rs190137766 with breast cancer risk [file 439_2015_1616_MOESM14_ESM.tif]

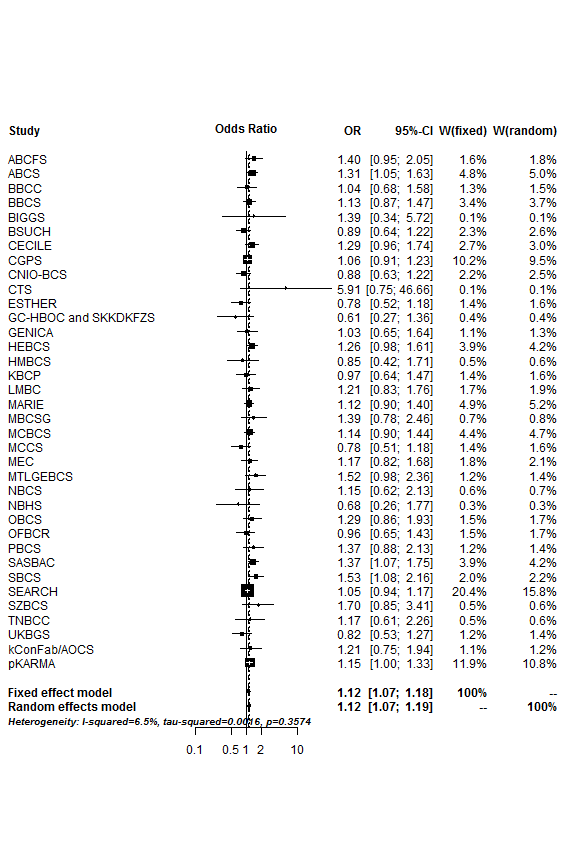

Supplement: Supplementary file 15 — ESM_15_ForestPlot_chr17_40607850_I.tif Forest plot showing meta-analysis of study-wise estimates for the association of chr17:40607850:I with breast cancer risk [file 439_2015_1616_MOESM15_ESM.tif]

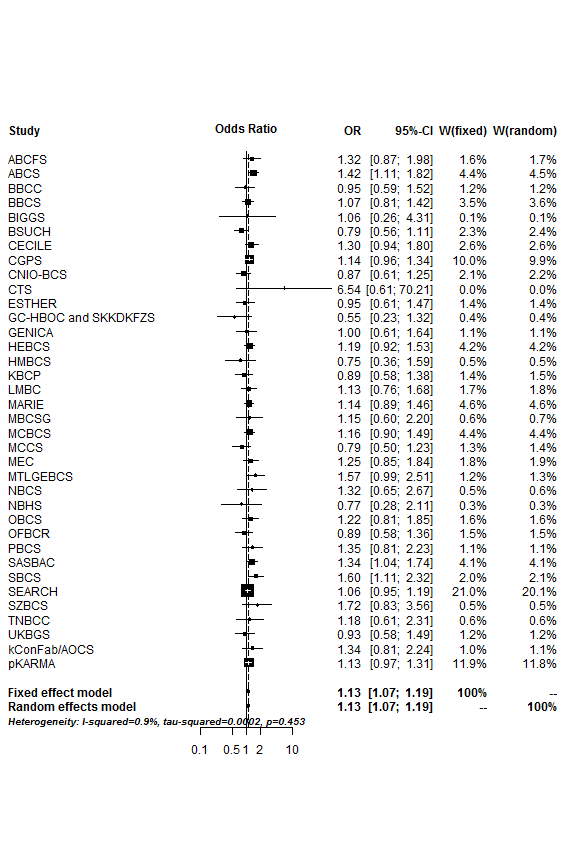

Supplement: Supplementary file 16 — ESM_16_ForestPlot_rs77942990.tif Forest plot showing meta-analysis of study-wise estimates for the association of rs77942990 with breast cancer risk [file 439_2015_1616_MOESM16_ESM.tif]

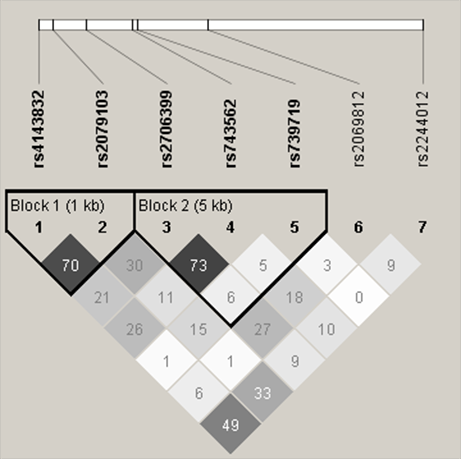

Supplement: Supplementary file 18 — ESM_18_LDplot_IL5.tif Linkage disequilibrium plot for seven SNPs at IL5 [file 439_2015_1616_MOESM18_ESM.tif]

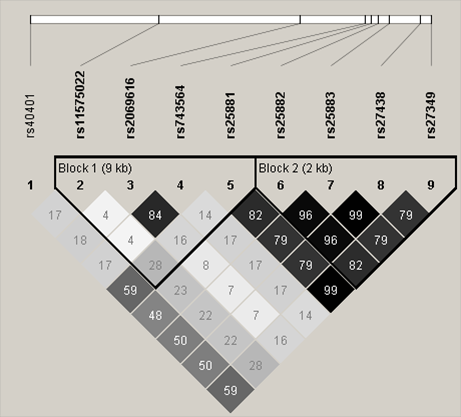

Supplement: Supplementary file 19 — ESM_19_LDplot_GM-CSF.tif Linkage disequilibrium plot for nine SNPs at GM-CSF [file 439_2015_1616_MOESM19_ESM.tif]

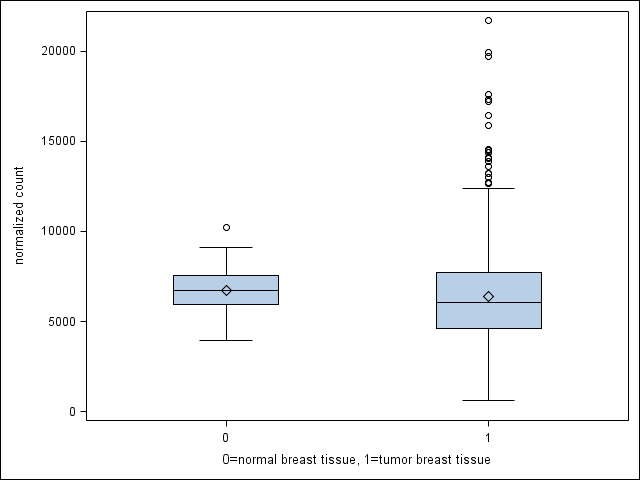

Supplement: Supplementary file 20 — ESM_20_Boxplot_STAT3.tif Box plot showing gene expression levels of STAT3 in normal breast tissue as well as tumor breast tissue [file 439_2015_1616_MOESM20_ESM.tif]

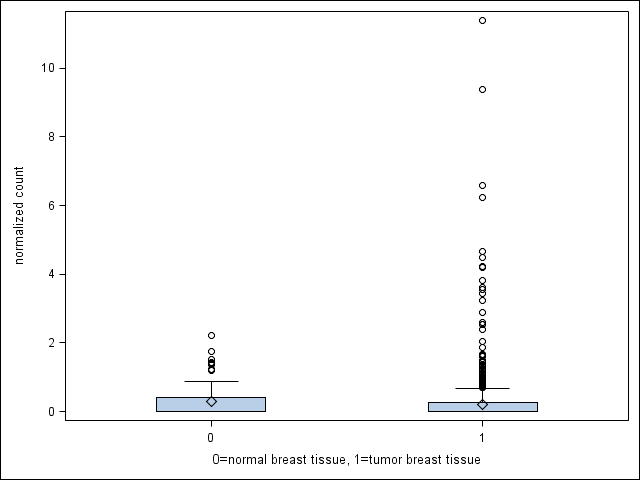

Supplement: Supplementary file 21 — ESM_21_Boxplot_IL5.tif Box plot showing gene expression levels of IL5 in normal breast tissue as well as tumor breast tissue [file 439_2015_1616_MOESM21_ESM.tif]

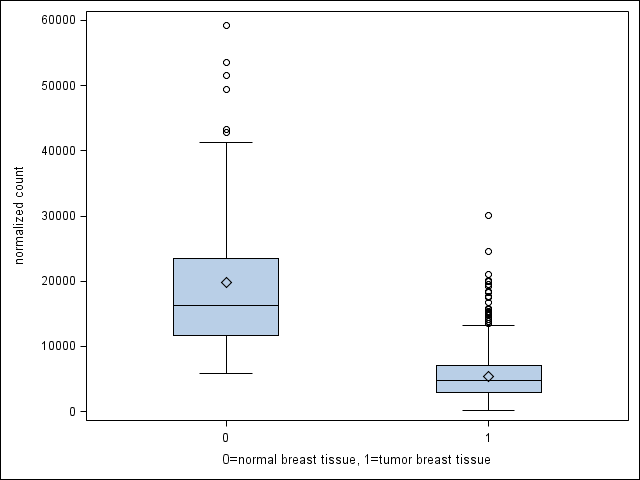

Supplement: Supplementary file 22 — ESM_22_Boxplot_PTRF.tif Box plot showing gene expression levels of PTRF in normal breast tissue as well as tumor breast tissue [file 439_2015_1616_MOESM22_ESM.tif]

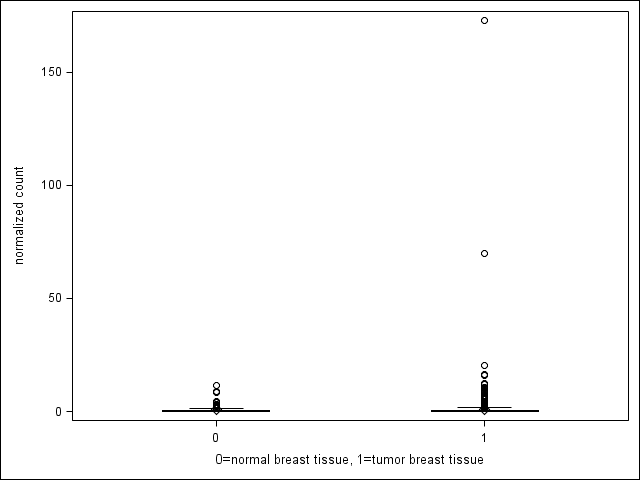

Supplement: Supplementary file 23 — ESM_23_Boxplot_CSF2.tif Box plot showing gene expression levels of GM-CSF in normal breast tissue as well as tumor breast tissue [file 439_2015_1616_MOESM23_ESM.tif]

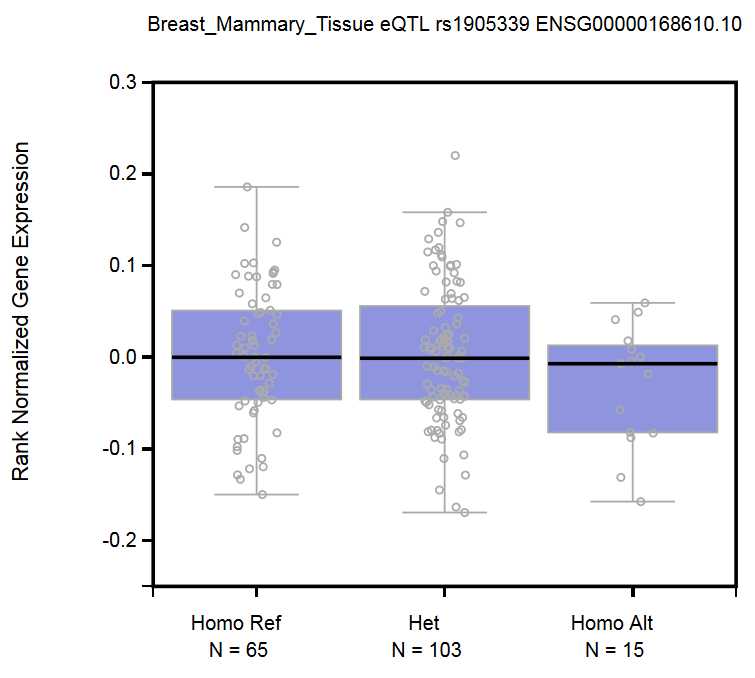

Supplement: Supplementary file 24 — ESM_24_eQTL_rs1905339_STAT3.tif Associations of rs1905339 genotypes with STAT3 expression within 183 breast tissue samples [file 439_2015_1616_MOESM24_ESM.tif]

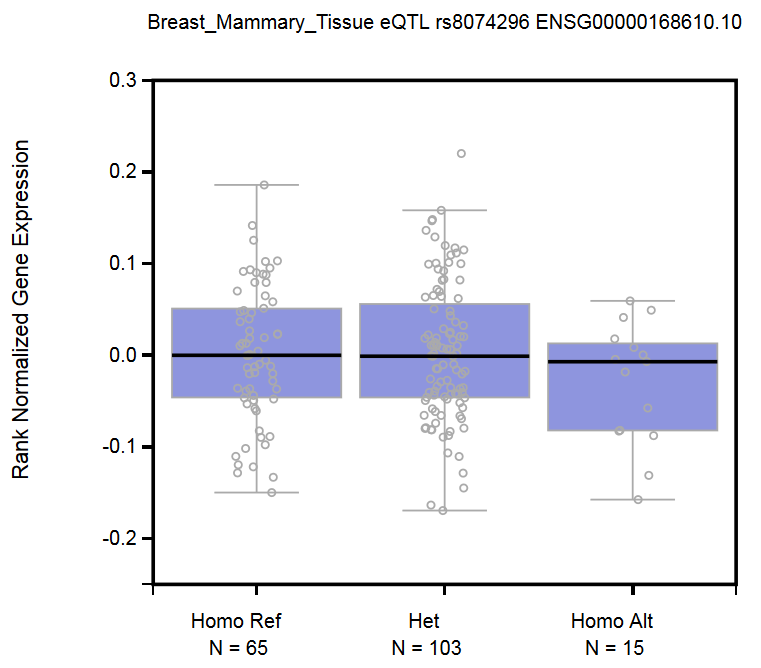

Supplement: Supplementary file 25 — ESM_25_eQTL_rs8074296_STAT3.tif Associations of rs8074296 genotypes with STAT3 expression within 183 breast tissue samples [file 439_2015_1616_MOESM25_ESM.tif]

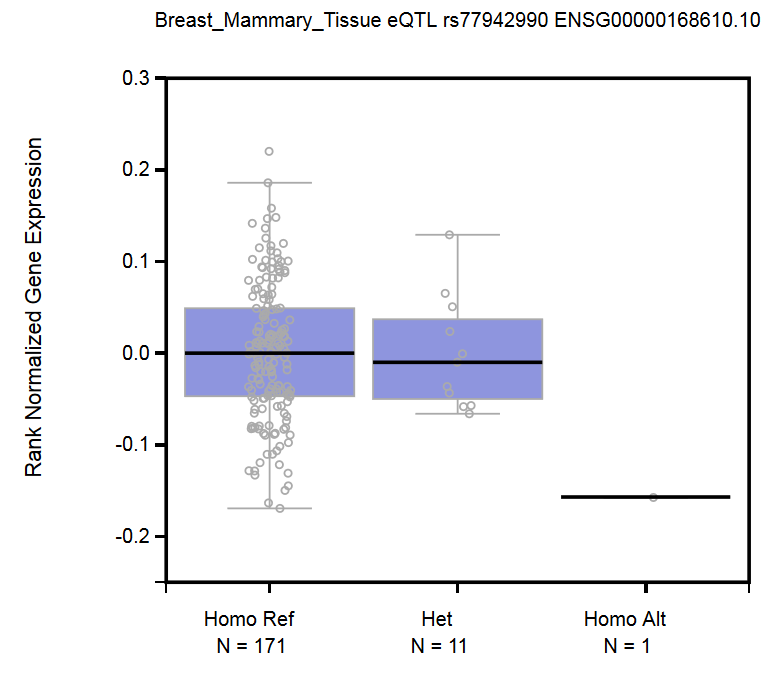

Supplement: Supplementary file 26 — ESM_26_eQTL_rs77942990_STAT3.tif Associations of rs8074296 genotypes with STAT3 expression within 183 breast tissue samples [file 439_2015_1616_MOESM26_ESM.tif]

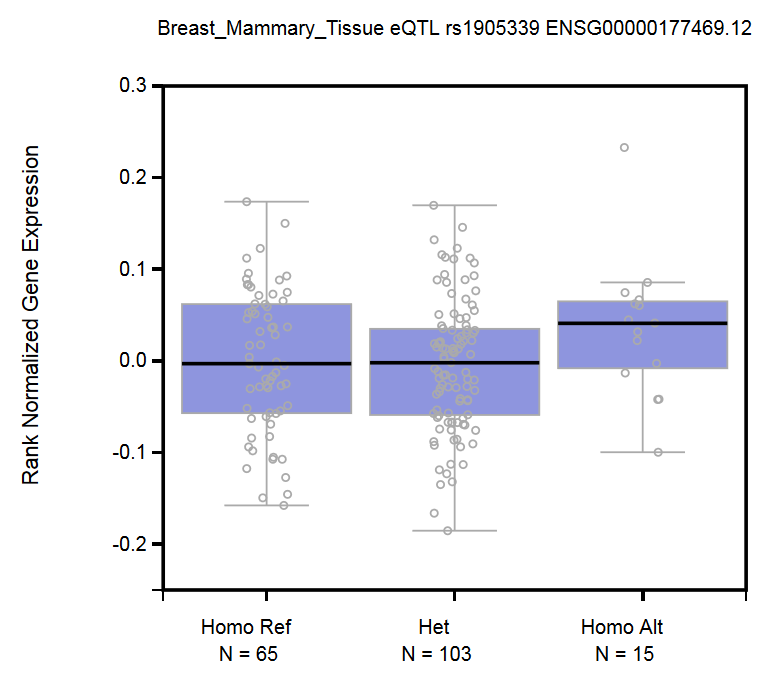

Supplement: Supplementary file 27 — ESM_27_eQTL_rs1905339_PTRF.tif Associations of rs1905339 genotypes with PTRF expression within 183 breast tissue samples [file 439_2015_1616_MOESM27_ESM.tif]

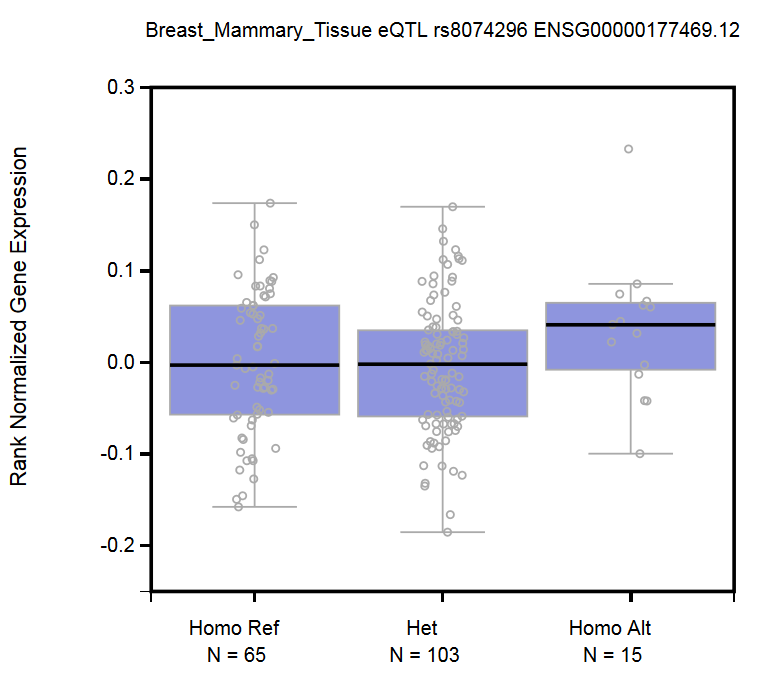

Supplement: Supplementary file 28 — ESM_28_eQTL_rs8074296_PTRF.tif Associations of rs8074296 genotypes with PTRF expression within 183 breast tissue samples [file 439_2015_1616_MOESM28_ESM.tif]

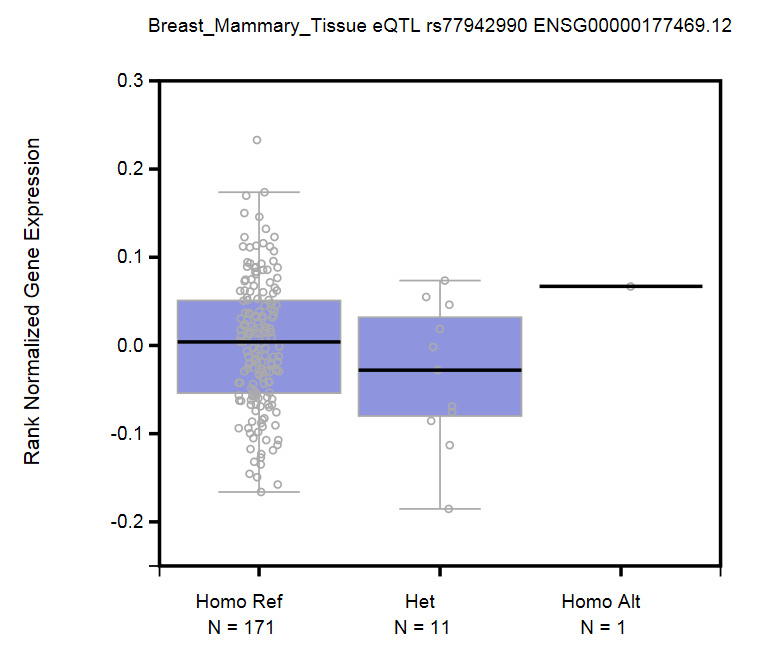

Supplement: Supplementary file 29 — ESM_29_eQTL_rs77942990_PTRF.tif Associations of rs77942990 genotypes with PTRF expression within 183 breast tissue samples [file 439_2015_1616_MOESM29_ESM.tif]

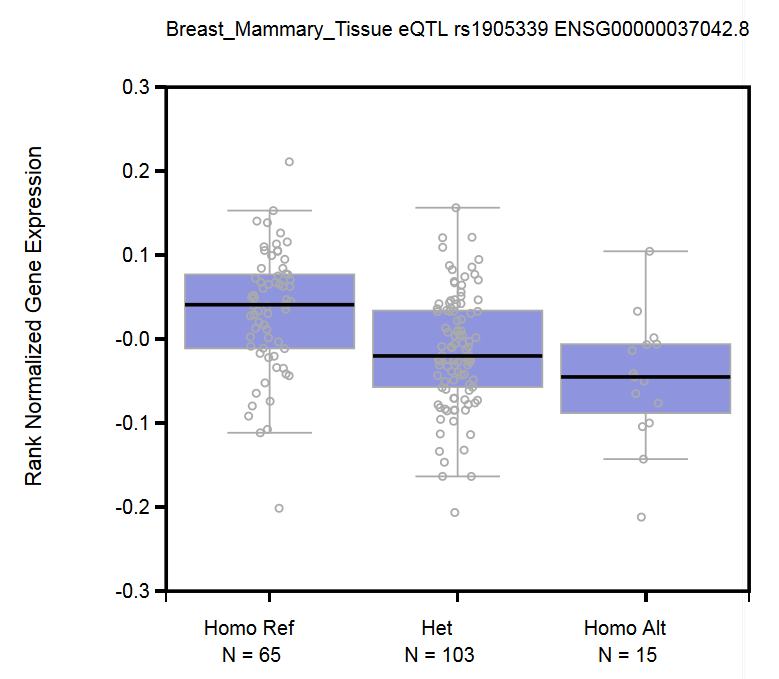

Supplement: Supplementary file 30 — ESM_30_eQTL_rs1905339_TUBG2.tif Associations of rs1905339 genotypes with TUBG2 expression within 183 breast tissue samples [file 439_2015_1616_MOESM30_ESM.tif]

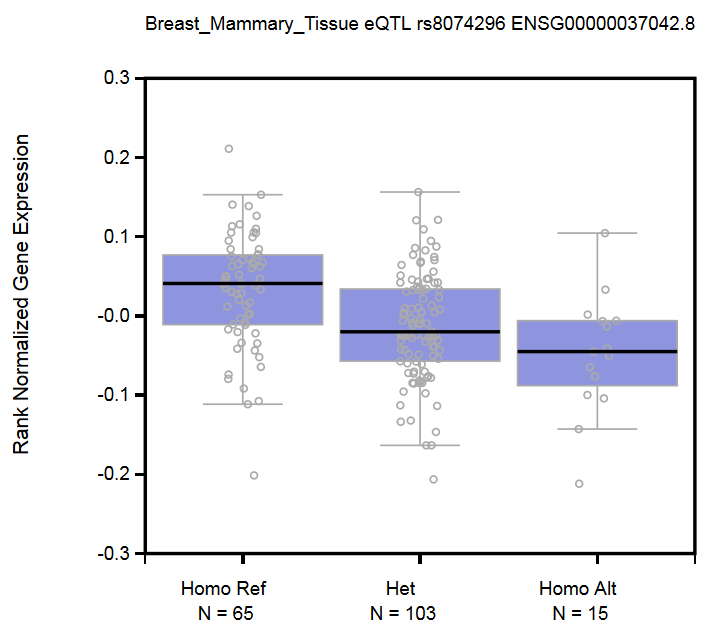

Supplement: Supplementary file 31 — ESM_31_eQTL_rs8074296_ TUBG2.tif Associations of rs8074296 genotypes with TUBG2 expression within 183 breast tissue samples [file 439_2015_1616_MOESM31_ESM.tif]
