# Supplementary material for: Genetic variation in the immunosuppression pathway genes and breast cancer susceptibility: a pooled analysis of 42,510 cases and 40,577 controls from the Breast Cancer Association Consortium
Source: Hum Genet. 2015 Nov 30;135:137–54. doi: 10.1007/s00439-015-1616-8 (PMC4698282; doi:10.1007/s00439-015-1616-8)
Supplement: Supplementary file 4 — ESM_4_Association_SNPs.pdf Associations with overall breast cancer risk for 3595 SNPs in the immunosuppression pathway genes [file 439_2015_1616_MOESM4_ESM.pdf]

Table S3 Associations with overall breast cancer risk for 3,595 SNPs in the immunosuppression pathway genes.

SNP	Chr	Position[a]	N Cases	N Controls	Allele	MAF	OR[b]	95%CI		*p* -value	Gene annotation	dbSNP functional annotation	Promoter histone marks	Enhancer histone marks	DNAse	Motifs changed
rs1431131	3	30675880	42,508	40,574	T>A	0.36	1.06	1.04	1.08	2.6E-08	*TGFBR2*	intronic		9 tissues		EWSR1-FLI1
rs12493607	3	30682939	42,494	40,572	C>G	0.34	1.06	1.04	1.08	6.2E-08	*TGFBR2*	intronic	BLD, GI	16 tissues		MZF1::1-4
rs12495646	3	30679970	42,425	40,511	C>A	0.34	1.06	1.04	1.08	1.1E-07	*TGFBR2*	intronic	BLD	7 tissues		EBF,ERalpha-a,VDR
rs13081020	3	30687969	42,501	40,574	A>G	0.34	1.06	1.04	1.08	1.4E-07	*TGFBR2*	intronic	BLD	11 tissues		5 altered motifs
rs1155705	3	30686414	42,479	40,543	A>G	0.34	1.06	1.04	1.08	1.5E-07	*TGFBR2*	intronic	BLD	13 tissues	16 tissues	
rs17838698	3	30684907	42,505	40,575	G>A	0.30	1.06	1.04	1.08	1.6E-07	*TGFBR2*	intronic	BLD	15 tissues	5 tissues	GATA,TCF11::MafG
rs12487185	3	30677269	42,506	40,573	A>G	0.31	1.06	1.03	1.08	3.4E-07	*TGFBR2*	intronic	BLD	17 tissues	7 tissues	HNF4
rs2372092	3	30687554	42,498	40,563	T>A	0.29	1.06	1.04	1.08	3.4E-07	*TGFBR2*	intronic		6 tissues	BLD	6 altered motifs
rs17025785	3	30667425	42,502	40,574	A>G	0.38	1.05	1.03	1.08	5.2E-07	*TGFBR2*	intronic	ESDR, SKIN	21 tissues	12 tissues	
rs11709624	3	30666452	42,510	40,575	G>C	0.38	1.05	1.03	1.07	7.3E-07	*TGFBR2*	intronic		11 tissues	BLD,SKIN	Nr2f2,Pbx-1
rs13086588	3	30688757	42,500	40,568	A>C	0.34	1.05	1.03	1.08	1.1E-06	*TGFBR2*	intronic	BLD	11 tissues		
rs1905339	17	40582296	42,504	40,576	A>G	0.33	1.05	1.03	1.08	1.4E-06	*ATP6V0A1, STAT3, PTRF*			10 tissues	13 tissues	Ik-2
rs11924422	3	30677484	42,491	40,572	A>C	0.41	0.95	0.94	0.97	6.9E-06	*TGFBR2*	intronic	BLD, GI	14 tissues	9 tissues	COMP1,Foxo,TCF11::MafG
rs2043138	3	30676017	42,505	40,574	G>C	0.39	0.95	0.94	0.97	1.0E-05	*TGFBR2*	intronic		10 tissues		
rs980441	3	30675326	42,498	40,558	A>G	0.40	0.96	0.94	0.98	1.4E-05	*TGFBR2*	intronic	BLD	10 tissues	BLD	
rs7224135	17	40574281	42,491	40,565	G>A	0.42	1.05	1.02	1.07	1.6E-05	*ATP6V0A1, STAT3, PTRF*	intronic	23 tissues	BLD, BRN	18 tissues	4 altered motifs
rs7177	11	69466115	42,411	40,496	A>C	0.47	0.96	0.94	0.98	2.7E-05	*CCND1, FGF19, ORAOV1, LOC100996515*	3'-UTR		SKIN, MUS, LNG	LIV	4 altered motifs
rs284147[c]	1	92278175	41,525	39,074	A>G	0.37	0.96	0.94	0.98	4.7E-05	*TGFBR3*					
rs1789167	11	69486244	42,503	40,576	G>A	0.33	0.96	0.94	0.98	1.4E-04	*CCND1, FGF19, ORAOV1, LOC100996515*	intronic		SKIN	BLD,BLD	5 altered motifs
rs41295353	10	6152220	42,491	40,565	G>C	0.14	1.06	1.03	1.09	1.6E-04	*IL2RA, PFKFB3, RBM17, RPL32P23, MIR3155A, MIR3155B*	intronic		6 tissues	BLD,BLD,HRT	4 altered motifs
rs2510467	11	69465681	42,488	40,570	G>A	0.42	0.96	0.94	0.98	1.7E-04	*CCND1, FGF19, ORAOV1, LOC100996515*	intronic		5 tissues	BLD,MUS	
rs13083813	3	30679558	42,471	40,560	T>A	0.36	0.96	0.94	0.98	1.9E-04	*TGFBR2*	intronic	BLD, GI	5 tissues	BLD	5 altered motifs
rs9823731	3	30683291	42,496	40,574	G>A	0.36	0.96	0.94	0.98	3.1E-04	*TGFBR2*	intronic	BLD, GI	19 tissues		Irx
rs12491780	3	30675054	42,295	40,309	G>A	0.27	1.04	1.02	1.06	4.2E-04	*TGFBR2*	intronic	BLD	11 tissues		PLAG1,Rad21
rs4522809	3	30668684	42,505	40,569	A>G	0.46	0.97	0.95	0.98	4.8E-04	*TGFBR2*	intronic		20 tissues	CRVX,SKIN	Hsf,KAP1
rs6802220	3	30659652	42,498	40,570	G>A	0.44	1.04	1.02	1.06	5.1E-04	*TGFBR2*	intronic	BLD	13 tissues	MUS	
rs9867701	3	30684683	42,492	40,573	A>G	0.36	0.96	0.94	0.98	5.7E-04	*TGFBR2*	intronic	FAT, BLD, MUS	17 tissues	11 tissues	BDP1,CTCFL,Myf
rs10796828	11	69490346	42,490	40,563	C>A	0.35	0.96	0.94	0.98	5.9E-04	*CCND1, FGF19, ORAOV1, LOC100996515*				51 tissues	
rs1789170	11	69509343	42,507	40,576	G>A	0.34	0.96	0.94	0.98	6.5E-04	*CCND1, FGF19, ORAOV1, LOC100129779, LOC100996515*					4 altered motifs
rs1789165	11	69481969	42,483	40,550	A>G	0.35	0.96	0.94	0.98	6.7E-04	*CCND1, FGF19, ORAOV1, LOC100996515*	3'-UTR		GI, THYM		FXR
rs7927491	11	69415560	41,574	39,748	C>G	0.27	1.04	1.02	1.06	7.4E-04	*CCND1, LOC100996515*			5 tissues	IPSC	8 altered motifs
rs654240	11	69448373	42,500	40,575	G>A	0.41	1.04	1.01	1.06	7.5E-04	*CCND1, ORAOV1, LOC100996515*					Ik-3,Nanog,TAL1
rs3212891	11	69465507	42,489	40,566	A>C	0.44	0.97	0.95	0.99	7.6E-04	*CCND1, FGF19, ORAOV1, LOC100996515*	intronic		5 tissues	LNG,SKIN	5 altered motifs
rs655089	11	69448575	42,493	40,570	A>C	0.46	0.97	0.95	0.99	9.9E-04	*CCND1, ORAOV1, LOC100996515*				IPSC	
rs2079103	5	131864506	42,500	40,567	C>A	0.25	0.96	0.94	0.98	1.0E-03	*IL5, IRF1, RAD50*		BLD	BLD, BRN, THYM		Pax-2
rs1789172	11	69494592	42,433	40,471	A>G	0.35	0.97	0.95	0.99	1.1E-03	*CCND1, FGF19, ORAOV1, LOC100996515*					
rs7109338	11	69412192	42,497	40,564	G>A	0.27	1.04	1.01	1.06	1.2E-03	*CCND1, LOC100996515*			IPSC, BLD, SPLN		
rs7112989	11	69412124	42,499	40,569	C>A	0.27	1.04	1.01	1.06	1.2E-03	*CCND1, LOC100996515*			IPSC, BLD, SPLN		CTCF,PTF1-beta
rs7109237	11	69412143	42,497	40,569	C>A	0.27	1.04	1.01	1.06	1.2E-03	*CCND1, LOC100996515*			IPSC, BLD, SPLN		Hmx,SP1
rs7109242	11	69411533	42,499	40,566	G>A	0.27	1.04	1.01	1.06	1.2E-03	*CCND1, LOC100996515*			SPLN		
rs10796826	11	69413010	42,494	40,561	A>G	0.27	1.04	1.01	1.06	1.3E-03	*CCND1, LOC100996515*				7 tissues	BRCA1,Nr2f2,Tgif1
rs678653	11	69466737	42,500	40,564	G>C	0.35	0.97	0.95	0.99	1.3E-03	*CCND1, FGF19, ORAOV1, LOC100996515*	3'-UTR		SKIN, MUS, LNG	HRT,LIV	AhR::Arnt,Arnt,Mtf1
rs7117072	11	69412881	42,498	40,574	C>A	0.27	1.04	1.01	1.06	1.3E-03	*CCND1, LOC100996515*			BLD, LNG		Gfi1,HDAC2,Nkx3
rs2498794	14	105245251	42,479	40,562	A>G	0.48	0.97	0.95	0.99	1.3E-03	*AKT1, SIVA1, ADSSL1, LINC00638, ZBTB42*	intronic		7 tissues	4 tissues	BCL
rs10737155	11	69413088	42,495	40,573	A>G	0.27	1.04	1.01	1.06	1.3E-03	*CCND1, LOC100996515*				19 tissues	TR4,Zbtb12
rs7939467	11	69410305	42,486	40,559	A>G	0.27	1.04	1.01	1.06	1.3E-03	*CCND1, LOC100996515*					TAL1
rs7948678	11	69410360	42,462	40,533	C>A	0.27	1.04	1.01	1.06	1.4E-03	*CCND1, LOC100996515*				LIV	
rs7939870	11	69414277	42,505	40,577	G>A	0.27	1.04	1.01	1.06	1.4E-03	*CCND1, LOC100996515*		BLD, SKIN, CRVX	6 tissues	5 tissues	AP-1,HES1,NRSF
rs7121742	11	69412211	42,497	40,560	A>G	0.27	1.04	1.01	1.06	1.4E-03	*CCND1, LOC100996515*			BLD, SKIN, SPLN		
rs10908194	11	69411103	42,497	40,567	G>A	0.27	1.04	1.01	1.06	1.4E-03	*CCND1, LOC100996515*					GR,Nrf-2
rs7121435	11	69413983	42,505	40,574	G>A	0.27	1.04	1.01	1.06	1.6E-03	*CCND1, LOC100996515*			4 tissues		RREB-1,Zfp740
rs7938336	11	69415367	42,462	40,525	A>G	0.27	1.04	1.01	1.06	1.6E-03	*CCND1, LOC100996515*			BLD, BRN, BONE		5 altered motifs
rs6606648	11	69408469	42,500	40,568	G>A	0.27	1.04	1.01	1.06	1.7E-03	*CCND1, LOC100996515*					6 altered motifs
rs7118790	11	69414816	42,479	40,546	T>A	0.27	1.04	1.01	1.06	1.7E-03	*CCND1, LOC100996515*			6 tissues	BRN,BRN	GATA,Maf
rs302140	7	18279936	42,503	40,577	A>G	0.26	0.96	0.94	0.99	1.8E-03	*HDAC9*	intronic				
rs6606649	11	69408850	42,499	40,565	A>G	0.27	1.04	1.01	1.06	1.8E-03	*CCND1, LOC100996515*				4 tissues	5 altered motifs
rs1991657	3	30658031	42,500	40,573	G>A	0.46	0.97	0.95	0.99	1.8E-03	*TGFBR2*	intronic	BLD	15 tissues	PLCNT,VAS	5 altered motifs
rs284156	1	92273253	42,505	40,576	G>C	0.41	1.03	1.01	1.05	1.8E-03	*TGFBR3*	intronic	FAT	20 tissues	6 tissues	5 altered motifs

SNP	Chr	Position[a]	N Cases	N Controls	Allele	MAF	OR[b]	95%CI		*p* -value	Gene annotation	dbSNP functional annotation	Promoter histone marks	Enhancer histone marks	DNAse	Motifs changed
rs654648	11	69448445	42,498	40,573	G>A	0.44	0.97	0.95	0.99	1.8E-03	*CCND1, ORAOV1, LOC100996515*					HNF4,RAR
rs1008562	2	219026972	42,501	40,572	C>G	0.48	0.97	0.95	0.99	2.0E-03	*CXCR1, CXCR2, HMGB1P9*					Pou5f1
rs4674257	2	218988774	42,505	40,576	G>A	0.48	1.03	1.01	1.05	2.1E-03	*CXCR1, CXCR2, RUFY4*			BLD		AIRE,Pax-4
rs6737563	2	218945674	42,499	40,574	G>A	0.48	1.03	1.01	1.05	2.2E-03	*CXCR2, CXCR2P1, RUFY4*	intronic	BLD	ESDR, BLD, MUS		5 altered motifs
rs9658706	10	90757620	42,502	40,575	A>G	0.11	0.95	0.92	0.98	2.2E-03	*ACTA2, FAS, FAS-AS1*	intronic	BLD	5 tissues	BRST	ATF3
rs4143832	5	131862977	42,498	40,575	C>A	0.19	0.96	0.94	0.99	2.2E-03	*IL5, IRF1, RAD50*		BLD, LIV	11 tissues	9 tissues	AIRE
rs284157	1	92271920	42,494	40,569	G>A	0.41	1.03	1.01	1.05	2.3E-03	*TGFBR3*	intronic	FAT	21 tissues	11 tissues	
rs4674259	2	218991005	42,495	40,569	A>G	0.48	1.03	1.01	1.05	2.3E-03	*CXCR1, CXCR2, RUFY4*	5'-UTR	BLD	7 tissues	ESC	5 altered motifs
rs470747	11	102661595	42,504	40,575	A>G	0.36	1.03	1.01	1.05	2.3E-03	*MMP1, MMP3, MMP10, CSNK1A1P2, WTAPP1, LOC100421658*	intronic				CEBPG,Hoxb6
rs17042509	2	113633009	42,509	40,576	G>A	0.07	0.94	0.90	0.98	2.5E-03	*IL1B, IL37, LOC100128413*			SKIN, BRST		EWSR1-FLI1
rs13104011	4	15807033	42,504	40,576	G>A	0.17	0.96	0.94	0.99	2.7E-03	*CD38*	intronic	MUS	BLD, MUS		Hic1
rs13013361	2	218947515	42,483	40,545	T>A	0.48	1.03	1.01	1.05	2.8E-03	*CXCR2, CXCR2P1, RUFY4*	intronic	LNG	4 tissues		Foxp1
rs4849127	2	113602559	42,506	40,575	G>A	0.07	0.94	0.90	0.98	2.9E-03	*IL1B, LOC100128413*					
rs9790268	3	30660680	42,495	40,567	G>A	0.45	1.03	1.01	1.05	2.9E-03	*TGFBR2*	intronic	BLD	13 tissues	BLD	8 altered motifs
rs470132	11	102666557	42,469	40,564	C>A	0.36	1.03	1.01	1.05	3.0E-03	*MMP1, MMP3, MMP10, CSNK1A1P2, WTAPP1, LOC100421658*	intronic	SKIN			13 altered motifs
rs3756242	4	15786002	42,507	40,576	C>A	0.17	0.96	0.94	0.99	3.0E-03	*CD38, LOC100288771*	intronic	BLD	6 tissues		CTCF,Spz1,TCF12
rs1571026	10	6135025	42,507	40,574	A>C	0.12	1.05	1.02	1.08	3.1E-03	*IL2RA, RBM17, RPL32P23*	intronic	BLD	5 tissues	BLD	AP-4,LBP-1
rs6682033	1	67708670	42,410	40,483	A>G	0.28	0.97	0.95	0.99	3.2E-03	*IL23R, LOC100130497*	intronic				11 altered motifs
rs6669666	1	23068465	42,509	40,574	G>A	0.21	0.96	0.94	0.99	3.2E-03	*EPHB2, MIR4684*	intronic		ESDR, VAS		Eomes,TBX5,ZEB1
rs3863057	3	30680370	42,484	40,560	G>A	0.34	0.97	0.95	0.99	3.3E-03	*TGFBR2*	intronic	BLD	12 tissues	8 tissues	RFX5,Rad21
rs10156056	7	22754088	42,506	40,573	C>G	0.12	0.96	0.93	0.98	3.3E-03	*IL6, LOC401312, LOC541472*					GR
rs9948182	18	60047489	42,501	40,570	G>A	0.34	0.97	0.95	0.99	3.4E-03	*TNFRSF11A, RPL17P44*	intronic				Mef2,PLZF,TATA
rs1872568	11	69418138	42,501	40,565	A>G	0.28	1.03	1.01	1.06	3.5E-03	*CCND1, LOC100996515*		ESC	5 tissues	IPSC,IPSC	BDP1,TBX5
rs603965	11	69462910	42,469	40,557	G>A	0.46	1.03	1.01	1.05	3.6E-03	*CCND1, ORAOV1, LOC100996515*					
rs11165720	1	92345120	42,504	40,570	G>A	0.09	1.05	1.02	1.09	3.7E-03	*TGFBR3*	intronic	FAT	12 tissues		4 altered motifs
rs1051070	10	90774772	42,509	40,575	A>T	0.12	0.95	0.92	0.99	3.9E-03	*ACTA2, FAS, FAS-AS1, MIR4679-1, MIR4679-2*	3'-UTR				BATF,HNF4,Pou2f2
rs9790292	3	30661210	42,467	40,556	G>A	0.46	1.03	1.01	1.05	3.9E-03	*TGFBR2*	intronic	BLD	16 tissues		
rs1343151	1	67719129	42,498	40,574	G>A	0.34	0.97	0.95	0.99	4.0E-03	*IL23R, LOC100130497*	intronic				
rs7143626	14	62224716	41,777	39,156	A>G	0.30	0.97	0.95	0.99	4.1E-03	*HIF1A, SNAPC1, HIF1A-AS2*			ESDR		5 altered motifs
rs5854	11	102660874	40,560	38,485	G>A	0.36	1.03	1.01	1.05	4.4E-03	*MMP1, MMP3, MMP10, CSNK1A1P2, WTAPP1, LOC100421658*	3'-UTR			SKIN	NF-AT1
rs518418	11	69406112	42,504	40,574	G>A	0.32	1.03	1.01	1.05	4.6E-03	*CCND1, LOC100996515*			LIV	GI	Mef2,TATA
rs9310938	3	30659119	42,499	40,572	G>A	0.43	1.03	1.01	1.05	5.0E-03	*TGFBR2*	intronic	BLD	11 tissues	BLD	Tel2
rs17029848	3	30661169	42,492	40,559	G>A	0.08	0.95	0.91	0.99	5.8E-03	*TGFBR2*	intronic	BLD	18 tissues		7 altered motifs
rs1143630	2	113591655	42,503	40,572	C>A	0.07	0.94	0.91	0.98	5.9E-03	*IL1A, IL1B, LOC100128413*	intronic	BLD	8 tissues	BLD,SKIN,BLD	4 altered motifs
rs743564	5	131410879	42,495	40,569	A>G	0.39	1.03	1.01	1.05	6.2E-03	*CSF2, IL3*	intronic	BLD, SKIN, BRST	12 tissues	BRST	6 altered motifs
rs17665435	18	60057341	42,454	40,541	T>A	0.33	0.97	0.95	0.99	6.6E-03	*TNFRSF11A, RPL17P44*			5 tissues		Myf,TEF-1
rs61839683	10	6131892	42,500	40,557	C>G	0.10	1.05	1.01	1.08	7.0E-03	*IL2RA, RBM17, RPL32P23*	5'-UTR	24 tissues		32 tissues	NERF1a,NRSF
rs7950206	11	69421272	42,474	40,547	G>A	0.28	1.03	1.01	1.05	7.1E-03	*CCND1, LOC100996515*			VAS	BRN,BRN	Smad4
rs10795752	10	6072354	42,501	40,576	G>A	0.40	0.97	0.95	0.99	7.2E-03	*IL2RA, RPL32P23*	intronic			BLD	
rs1036097	3	30693643	42,506	40,572	G>A	0.44	0.97	0.95	0.99	7.4E-03	*TGFBR2*	intronic			ESDR,SKIN	COMP1,SREBP
rs2082224	3	30689755	42,503	40,575	G>A	0.24	1.03	1.01	1.06	7.5E-03	*TGFBR2*	intronic	BLD	12 tissues	BLD	
rs12590163	14	105223525	42,481	40,560	A>G	0.45	1.03	1.01	1.05	7.6E-03	*AKT1, SIVA1, INF2, ADSSL1, ZBTB42, LOC100996409*	intronic		5 tissues	THYM,BLD,BLD	4 altered motifs
rs1808602	3	30690424	42,494	40,564	A>G	0.45	0.97	0.95	0.99	8.0E-03	*TGFBR2*	intronic	BLD	22 tissues	BLD	HDAC2,NF-kappaB
rs4247034	14	105208879	42,420	40,473	C>G	0.45	1.03	1.01	1.05	8.3E-03	*AKT1, SIVA1, INF2, ADSSL1, LOC100996409*	intronic		6 tissues		4 altered motifs
rs1317681	1	218575202	42,408	40,470	G>A	0.16	0.96	0.94	0.99	8.4E-03	*TGFB2*	intronic	GI	9 tissues	LNG	SRF
rs995029	12	88890521	41,939	39,740	A>G	0.09	0.95	0.92	0.99	8.6E-03	*KITLG*	3'-UTR		9 tissues	BRN,BRN	5 altered motifs
rs7142772	14	105200377	42,465	40,558	A>C	0.45	1.03	1.01	1.05	8.9E-03	*AKT1, SIVA1, INF2, ADSSL1, LOC100996409*	intronic	SKIN, CRVX	10 tissues	ESC,ESDR,MUS	Ik-2,Sin3Ak-20,Spz1
rs4983386	14	105210207	42,480	40,560	G>A	0.45	1.03	1.01	1.05	8.9E-03	*AKT1, SIVA1, INF2, ADSSL1, LOC100996409*	intronic		6 tissues	BLD	HNF4
rs7974506	12	88848006	42,509	40,577	G>A	0.07	0.95	0.91	0.99	9.0E-03	*KITLG*			6 tissues		HNF4,PPAR
rs2494734	14	105240885	42,497	40,565	C>G	0.46	1.03	1.01	1.05	9.1E-03	*AKT1, SIVA1, ADSSL1, LINC00638, ZBTB42*	intronic		5 tissues	9 tissues	4 altered motifs
rs7135958	12	88933744	41,933	39,871	A>C	0.09	0.95	0.92	0.99	9.6E-03	*KITLG*	intronic				4 altered motifs
rs10270171	7	22813879	42,481	40,517	A>C	0.22	0.97	0.95	0.99	9.7E-03	*IL6, TOMM7, RPS26P32, LOC541472*					
rs17500235	17	40356360	42,506	40,576	A>C	0.07	1.05	1.01	1.10	9.9E-03	*HCRT, RAB5C, STAT5B, KCNH4, GHDC*	intronic		KID		Foxp3
rs7034893	9	5494438	42,237	40,346	A>G	0.28	1.03	1.01	1.05	9.9E-03	*CD274, PDCD1LG2*		BLD, BRN	9 tissues	21 tissues	EWSR1-FLI1
rs11256802	10	6150501	42,509	40,574	A>C	0.08	0.95	0.92	0.99	1.0E-02	*IL2RA, PFKFB3, RBM17, RPL32P23, MIR3155A, MIR3155B*	intronic	ESC, IPSC	9 tissues	ESC,IPSC	DMRT2,Nanog
rs2745557	1	186649221	42,497	40,569	G>A	0.17	1.04	1.01	1.06	1.0E-02	*PTGS2*	intronic	23 tissues	BLD	28 tissues	
rs10815232	9	5480872	42,503	40,567	T>A	0.23	1.03	1.01	1.06	1.1E-02	*CD274, PLGRKT, PDCD1LG2*				BLD	CEBPB,Cdc5

SNP	Chr	Position[a]	N Cases	N Controls	Allele	MAF	OR[b]	95%CI		*p* -value	Gene annotation	dbSNP functional annotation	Promoter histone marks	Enhancer histone marks	DNAse	Motifs changed
rs7125320	11	102663708	42,127	40,221	A>C	0.08	0.95	0.92	0.99	1.1E-02	*MMP1, MMP3, MMP10, CSNK1A1P2, WTAPP1, LOC100421658*	intronic			GI	Pou3f3,SP1,Zfp105
rs25645	17	38173143	42,481	40,572	G>A	0.37	1.03	1.01	1.05	1.1E-02	*CSF3, PSMD3, THRA, MED24, GSDMA, LOC100505620*	synonymous	SKIN, MUS	14 tissues		Glis2
rs4948876	10	44856746	42,508	40,573	A>T	0.18	1.03	1.01	1.06	1.1E-02	*CXCL12*			LNG	LNG	Maf,TEF-1
rs4789555	17	76215273	42,507	40,572	A>G	0.08	0.95	0.92	0.99	1.2E-02	*BIRC5, TK1, SYNGR2, AFMID, TMEM235, THA1P, LOC100996291*	intronic		4 tissues		Pou2f2,Pou3f2
rs2227319	17	38170845	42,454	40,536	G>A	0.37	1.03	1.01	1.05	1.2E-02	*CSF3, PSMD3, THRA, MED24, GSDMA, LOC100505620*		MUS, LIV	18 tissues	14 tissues	9 altered motifs
rs2227321	17	38171294	42,345	40,425	C>G	0.37	1.03	1.01	1.05	1.2E-02	*CSF3, PSMD3, THRA, MED24, GSDMA, LOC100505620*		MUS, LIV	19 tissues	4 tissues	7 altered motifs
rs1797071	1	218581509	42,477	40,535	A>G	0.35	0.97	0.95	0.99	1.2E-02	*TGFB2*	intronic		MUS		
rs3017614	11	69435230	42,301	40,243	G>A	0.28	1.03	1.01	1.05	1.2E-02	*CCND1, ORAOV1, LOC100996515*					Foxp1,Msx-1
rs2745559	1	186652002	42,447	40,477	C>A	0.17	1.03	1.01	1.06	1.2E-02	*PTGS2*		STRM, BLD, SKIN	9 tissues	9 tissues	OTX
rs3024647	16	27367972	42,507	40,574	A>G	0.15	0.96	0.94	0.99	1.2E-02	*IL4R, IL21R*	intronic		BLD	MUS	BRCA1,Rad21
rs1036095	3	30662328	42,498	40,570	C>G	0.24	0.97	0.95	0.99	1.2E-02	*TGFBR2*	intronic			BLD,SKIN	
rs4742100	9	5470954	42,500	40,571	A>C	0.23	1.03	1.01	1.06	1.2E-02	*CD274, PLGRKT, PDCD1LG2*			BLD, MUS	BLD,SKIN	
rs17347096	7	18384157	42,375	40,444	G>A	0.47	1.03	1.01	1.05	1.2E-02	*HDAC9*	intronic		LIV, BRN		4 altered motifs
rs4658266	1	92240747	42,458	40,548	C>G	0.48	1.03	1.01	1.05	1.3E-02	*TGFBR3*	intronic		10 tissues		BCL,HNF4,NF-kappaB
rs6693831	1	67720867	42,473	40,546	G>A	0.25	1.03	1.01	1.05	1.3E-02	*IL23R, LOC100130497*	intronic				4 altered motifs
rs61758463	14	105258067	42,496	40,564	G>A	0.27	1.03	1.01	1.05	1.3E-02	*AKT1, SIVA1, RPS2P4, ADSSL1, LINC00638, ZBTB42, RPS26P49*	intronic		16 tissues	OVRY	Gmeb1
rs3773652	3	30718942	42,493	40,573	A>G	0.45	0.98	0.96	0.99	1.4E-02	*TGFBR2, GADL1*	intronic		8 tissues	BLD	
rs10863397	1	218585248	42,509	40,576	G>A	0.06	1.05	1.01	1.10	1.4E-02	*TGFB2*	intronic				8 altered motifs
rs470358	11	102668702	42,472	40,554	G>A	0.40	0.97	0.96	0.99	1.5E-02	*MMP1, MMP3, MMP10, CSNK1A1P2, WTAPP1, LOC100421658*	intronic	FAT, SKIN, VAS	8 tissues	5 tissues	
rs12493471	3	45951678	42,502	40,575	G>A	0.36	0.97	0.95	1.00	1.5E-02	*CXCR6, CCR9, SDHDP4, FYCO1*			BLD		16 altered motifs
rs17413169	10	33533148	42,507	40,577	A>G	0.26	0.97	0.95	0.99	1.5E-02	*NRP1*	intronic		4 tissues	MUS	Foxa,GR
rs11165496	1	92250347	42,491	40,567	G>C	0.39	1.03	1.00	1.05	1.5E-02	*TGFBR3*	intronic	FAT	18 tissues	HRT	AP-1,RREB-1,p300
rs3024613	16	27364253	42,506	40,574	G>A	0.50	1.02	1.00	1.05	1.6E-02	*IL4R, IL21R*	intronic				
rs11259432	10	6578628	42,437	40,525	A>G	0.38	1.03	1.00	1.05	1.6E-02	*PRKCQ, PRKCQ-AS1*	intronic		BLD, THYM		
rs17025795	3	30673720	42,510	40,576	G>A	0.07	1.05	1.01	1.09	1.6E-02	*TGFBR2*	intronic	BLD	16 tissues	16 tissues	BATF,Nanog,Pou5f1
rs995435	3	30700922	42,506	40,576	G>A	0.27	1.03	1.01	1.05	1.6E-02	*TGFBR2*	intronic	4 tissues	23 tissues	24 tissues	4 altered motifs
rs7552664	1	12277967	42,501	40,574	C>A	0.27	0.97	0.95	0.99	1.6E-02	*TNFRSF1B, VPS13D, LOC390998, MIR4632*			BLD		Smad,THAP1
rs10917288	1	23071845	42,506	40,574	C>G	0.19	1.03	1.01	1.06	1.6E-02	*EPHB2, MIR4684*	intronic				5 altered motifs
rs7652331	3	45962752	42,501	40,576	G>A	0.35	0.97	0.95	1.00	1.6E-02	*CXCR6, CCR9, SDHDP4, FYCO1*	3'-UTR		BLD, THYM		Pax-2,TATA,YY1
rs77290390	11	69453506	42,495	40,574	G>A	0.11	0.96	0.93	0.99	1.6E-02	*CCND1, ORAOV1, LOC100996515*		21 tissues	8 tissues	41 tissues	
rs3773636	3	30700441	42,383	40,416	G>A	0.27	1.03	1.01	1.05	1.7E-02	*TGFBR2*	intronic	12 tissues	19 tissues	29 tissues	4 altered motifs
rs3803307	14	105207134	42,499	40,569	A>G	0.46	1.02	1.00	1.05	1.7E-02	*AKT1, SIVA1, INF2, ADSSL1, LOC100996409*	intronic		4 tissues		
rs2069616	5	131408077	42,467	40,549	A>G	0.42	1.03	1.00	1.05	1.7E-02	*CSF2, IL3*			5 tissues	MUS	Evi-1,NRSF,Osf2
rs1358980	6	43764551	42,503	40,575	G>A	0.47	0.98	0.96	1.00	1.7E-02	*VEGFA*			6 tissues	MUS	FXR,Rhox11,VDR
rs1864615	3	30699782	42,507	40,576	G>A	0.27	1.03	1.00	1.05	1.7E-02	*TGFBR2*	intronic	6 tissues	20 tissues	9 tissues	Pbx3,Pou2f2
rs10905641	10	6072293	42,493	40,563	A>C	0.41	0.98	0.96	1.00	1.7E-02	*IL2RA, RPL32P23*	intronic				
rs6946864	7	22751949	42,503	40,572	A>C	0.19	0.97	0.95	0.99	1.8E-02	*IL6, LOC401312, LOC541472*			BLD		Cdx2,Hoxa9
rs6816486	4	15795482	41,400	39,455	G>A	0.16	0.97	0.94	0.99	1.8E-02	*CD38*	intronic		5 tissues	BRN	4 altered motifs
rs2250402	15	40322552	42,508	40,576	A>C	0.08	1.05	1.01	1.09	1.8E-02	*SRP14, EIF2AK4, SRP14-AS1*	intronic	HRT	6 tissues	12 tissues	ZBTB33
rs10121158	9	5483919	42,507	40,575	A>G	0.32	1.03	1.00	1.05	1.8E-02	*CD274, PLGRKT, PDCD1LG2*					
rs11764843	7	18935310	42,442	40,545	C>A	0.38	1.02	1.00	1.05	1.8E-02	*HDAC9*	intronic	7 tissues	9 tissues	16 tissues	8 altered motifs
rs10122509	9	5484203	42,499	40,570	A>G	0.32	1.03	1.00	1.05	1.9E-02	*CD274, PLGRKT, PDCD1LG2*					CTCF
rs1008563	2	219026888	42,481	40,563	G>A	0.43	0.98	0.96	1.00	1.9E-02	*CXCR1, CXCR2, HMGB1P9*					4 altered motifs
rs34900340	3	30663450	42,500	40,573	G>A	0.47	0.98	0.96	1.00	1.9E-02	*TGFBR2*	intronic	BLD	15 tissues	BLD,VAS	Irx,Pou1f1,TATA
rs7964695	12	88935629	42,488	40,563	A>C	0.09	0.96	0.93	0.99	1.9E-02	*KITLG*	intronic		STRM, SKIN	SKIN,MUS	5 altered motifs
rs12756024	1	92248440	42,505	40,575	C>A	0.46	1.02	1.00	1.04	1.9E-02	*TGFBR3*	intronic	LNG	20 tissues	IPSC	STAT
rs56226206	11	69435265	42,507	40,575	G>A	0.28	1.03	1.00	1.05	2.0E-02	*CCND1, ORAOV1, LOC100996515*					HDAC2,p300
rs1556187	9	5486384	42,498	40,566	T>A	0.33	1.03	1.00	1.05	2.1E-02	*CD274, PLGRKT, PDCD1LG2*			4 tissues		4 altered motifs
rs734186	10	33484829	42,495	40,565	G>A	0.37	1.02	1.00	1.05	2.1E-02	*NRP1*	intronic		16 tissues	9 tissues	E2F
rs2163417	3	30656573	42,506	40,575	G>A	0.07	0.96	0.92	0.99	2.2E-02	*TGFBR2*	intronic	BLD, GI	17 tissues	12 tissues	Isl2,Nkx2
rs6683840	1	92249817	42,496	40,572	A>G	0.46	1.02	1.00	1.04	2.2E-02	*TGFBR3*	intronic	FAT	18 tissues		AP-2
rs1492354	12	88947978	42,509	40,576	G>A	0.08	0.96	0.92	0.99	2.2E-02	*KITLG*	intronic	9 tissues	18 tissues	20 tissues	
rs2827	17	38173737	42,496	40,560	G>A	0.17	0.97	0.94	1.00	2.2E-02	*CSF3, PSMD3, THRA, MED24, GSDMA, LOC100505620*	3'-UTR	MUS	15 tissues	LNG,MUS	4 altered motifs
rs636800	11	69434896	42,503	40,574	G>A	0.28	1.03	1.00	1.05	2.2E-02	*CCND1, ORAOV1, LOC100996515*					6 altered motifs

SNP	Chr	Position[a]	N Cases	N Controls	Allele	MAF	OR[b]	95%CI		*p* -value	Gene annotation	dbSNP functional annotation	Promoter histone marks	Enhancer histone marks	DNAse	Motifs changed
rs7929871	11	69423221	42,504	40,569	C>A	0.27	1.03	1.00	1.05	2.2E-02	*CCND1, LOC100996515*					
rs12949918	17	40526273	42,507	40,577	A>G	0.42	1.02	1.00	1.04	2.2E-02	*STAT3, PTRF*	intronic	BLD	7 tissues	PLCNT	
rs7933453	11	69426849	42,502	40,558	A>G	0.27	1.03	1.00	1.05	2.2E-02	*CCND1, LOC100996515*			IPSC, LIV		
rs40401	5	131396478	42,502	40,573	G>A	0.23	0.97	0.95	1.00	2.2E-02	*CSF2, IL3, ACSL6*	missense		BLD		Maf,TFIIA
rs7946645	11	69425840	42,502	40,564	A>G	0.27	1.03	1.00	1.05	2.2E-02	*CCND1, LOC100996515*					BDP1,Hsf,Pax-4
rs2087016	11	69428532	42,506	40,573	C>A	0.27	1.03	1.00	1.05	2.2E-02	*CCND1, LOC100996515*					6 altered motifs
rs334804	3	3138417	42,506	40,573	G>A	0.26	0.97	0.95	1.00	2.3E-02	*IL5RA, TRNT1, CNTN4*	intronic		ESDR		
rs3024610	16	27364158	42,405	40,476	G>A	0.50	1.02	1.00	1.04	2.3E-02	*IL4R, IL21R*	intronic		GI		5 altered motifs
rs667515	11	69449076	42,485	40,572	C>G	0.37	0.98	0.96	1.00	2.3E-02	*CCND1, ORAOV1, LOC100996515*			ESDR, ADRL, MUS	IPSC,SKIN	13 altered motifs
rs545143	3	159731995	42,504	40,575	G>A	0.42	0.98	0.96	1.00	2.4E-02	*IL12A*			BLD		6 altered motifs
rs1864616	3	30665228	42,503	40,568	G>A	0.24	0.97	0.95	1.00	2.4E-02	*TGFBR2*	intronic	BLD	12 tissues	4 tissues	
rs17555072	10	6553071	42,506	40,571	A>G	0.08	1.04	1.01	1.08	2.4E-02	*PRKCQ*	synonymous		GI, MUS		Pax-1
rs3024660	16	27371158	42,454	40,529	A>G	0.15	0.97	0.94	1.00	2.4E-02	*IL4R, IL21R*	intronic		BRST	SKIN	HNF4,RORalpha1,RXRA
rs2510460	11	69450305	42,500	40,571	A>G	0.49	0.98	0.96	1.00	2.4E-02	*CCND1, ORAOV1, LOC100996515*			8 tissues	6 tissues	Bcl6b,Ik-2,Irf
rs7950525	11	69426696	42,484	40,554	A>G	0.27	1.03	1.00	1.05	2.4E-02	*CCND1, LOC100996515*			IPSC, LIV, BONE		
rs17516329	1	92319781	42,508	40,575	T>A	0.32	0.98	0.96	1.00	2.5E-02	*TGFBR3*	intronic		BRST, BLD, HRT		SRF,TATA,YY1
rs1331317	10	33517647	42,508	40,575	A>G	0.33	1.02	1.00	1.05	2.5E-02	*NRP1*	intronic		5 tissues	MUS,MUS,VAS	AIRE
rs10777125	12	88915835	42,345	40,367	A>G	0.09	0.96	0.93	0.99	2.5E-02	*KITLG*	intronic				HNF1
rs7933440	11	69426822	42,496	40,560	A>G	0.27	1.03	1.00	1.05	2.5E-02	*CCND1, LOC100996515*			IPSC, LIV	BRN	CACD,Hdx,SMC3
rs11600406	11	69415936	42,491	40,565	G>A	0.08	1.04	1.01	1.08	2.5E-02	*CCND1, LOC100996515*			LNG, SKIN, LIV	LNG	6 altered motifs
rs11263509	11	69425248	42,489	40,559	G>A	0.27	1.03	1.00	1.05	2.6E-02	*CCND1, LOC100996515*				BRN,BRN	Mrg1::Hoxa9,Mrg,TAL1
rs480134	3	159729532	42,504	40,572	G>A	0.42	0.98	0.96	1.00	2.6E-02	*IL12A*			BLD		Nanog,p300
rs1703081	12	88951387	42,509	40,577	G>A	0.08	0.96	0.92	1.00	2.6E-02	*KITLG*	intronic		BRN, SKIN	LNG,BLD	4 altered motifs
rs5005162	1	92312153	42,509	40,576	T>A	0.36	0.98	0.96	1.00	2.6E-02	*TGFBR3*	intronic	SKIN, LNG, CRVX	17 tissues	HRT	Hoxa10,RXRA
rs1770345	1	11214580	42,484	40,542	A>C	0.48	1.02	1.00	1.04	2.6E-02	*MTOR, ANGPTL7, MTOR-AS1*	intronic				Pou2f2
rs2391071	1	92312152	42,332	40,374	A>T	0.36	0.98	0.96	1.00	2.6E-02	*TGFBR3*	intronic	SKIN, LNG, CRVX	17 tissues	HRT	Foxo,Lhx3,RXRA
rs9319408	13	28570608	42,499	40,567	G>A	0.31	1.02	1.00	1.05	2.7E-02	*CDX2, FLT3, PRHOXNB, LINC00543*					GATA,Mef2
rs860416	5	35906981	42,506	40,573	G>A	0.16	0.97	0.94	1.00	2.7E-02	*IL7R, UGT3A1, CAPSL, LOC100506406*	intronic		THYM, BLD		8 altered motifs
rs1492347	12	88925973	42,487	40,542	G>A	0.09	0.96	0.93	1.00	2.7E-02	*KITLG*	intronic		BRST		EBF,HMG-IY
rs2244012	5	131901225	42,505	40,575	A>G	0.22	0.97	0.95	1.00	2.7E-02	*IL5, RAD50*	intronic				Nrf-2,Zbtb12
rs1798011	12	88956625	42,510	40,577	G>A	0.08	0.96	0.92	1.00	2.7E-02	*KITLG*	intronic	FAT, BONE	11 tissues		
rs1155506	19	40266711	42,506	40,570	A>G	0.26	1.03	1.00	1.05	2.8E-02	*CLC, DYRK1B, LEUTX*					BATF,Irf
rs2236534	10	44874309	42,505	40,574	C>A	0.21	1.03	1.00	1.05	2.8E-02	*CXCL12, RPL9P21*	intronic	STRM	6 tissues		GR,RP58
rs3024656	16	27369609	42,503	40,571	G>A	0.29	1.02	1.00	1.05	2.8E-02	*IL4R, IL21R*	intronic		LNG	OVRY	AP-2
rs2071230	11	102660959	42,421	40,516	A>G	0.08	0.96	0.92	1.00	2.8E-02	*MMP1, MMP3, MMP10, CSNK1A1P2, WTAPP1, LOC100421658*	3'-UTR			SKIN	5 altered motifs
rs2583778	8	79651141	42,481	40,540	G>A	0.18	1.03	1.00	1.06	2.8E-02	*IL7, ZC2HC1A, PRKRIRP7*	intronic	LIV, PANC	GI	PANC	7 altered motifs
rs7969188	12	88924067	42,508	40,574	G>C	0.09	0.96	0.93	1.00	2.9E-02	*KITLG*	intronic		10 tissues	SKIN	CTCF,Mef2,Rad21
rs17883307	1	92270234	42,505	40,569	G>A	0.14	0.97	0.94	1.00	2.9E-02	*TGFBR3*	intronic	4 tissues	19 tissues	16 tissues	
rs514921	11	102669230	42,507	40,571	A>G	0.28	1.02	1.00	1.05	2.9E-02	*MMP1, MMP3, MMP10, CSNK1A1P2, WTAPP1, LOC100421658*	intronic	FAT	10 tissues	4 tissues	6 altered motifs
rs1405618	7	18650086	42,506	40,569	G>A	0.32	0.98	0.96	1.00	2.9E-02	*HDAC9, LOC100419901*	intronic		VAS	13 tissues	14 altered motifs
rs11567762	5	35873201	42,508	40,576	G>A	0.14	0.97	0.94	1.00	2.9E-02	*IL7R, CAPSL*	intronic		BLD, THYM		4 altered motifs
rs17514919	1	92264005	41,605	39,616	A>G	0.14	0.97	0.94	1.00	2.9E-02	*TGFBR3*	intronic		18 tissues	10 tissues	HP1-site-factor
rs11598704	10	6035149	42,476	40,552	G>A	0.22	0.97	0.95	1.00	2.9E-02	*IL2RA, IL15RA*					Myc
rs744166	17	40514201	42,503	40,575	A>G	0.42	1.02	1.00	1.04	2.9E-02	*STAT3, PTRF*	intronic	MUS, GI	24 tissues	4 tissues	PLZF
rs4658270	1	92258782	42,507	40,573	G>A	0.32	1.02	1.00	1.05	2.9E-02	*TGFBR3*	intronic		17 tissues		MAZ
rs1423658	5	35887640	42,509	40,575	G>A	0.14	0.97	0.94	1.00	2.9E-02	*IL7R, CAPSL*			BLD, THYM	ESDR,ESDR,CRVX	Ik-3
rs893964	1	23086096	42,508	40,576	G>A	0.19	1.03	1.00	1.05	2.9E-02	*EPHB2, MIR4684*	intronic		MUS		
rs6871536	5	131969874	42,486	40,571	A>G	0.21	0.97	0.95	1.00	2.9E-02	*IL4, IL13, RAD50*	intronic				Osf2
rs12043241	1	92307058	42,505	40,571	A>G	0.36	0.98	0.96	1.00	3.0E-02	*TGFBR3*	intronic		10 tissues	SKIN	5 altered motifs
rs7555476	1	92310666	42,477	40,560	A>G	0.36	0.98	0.96	1.00	3.0E-02	*TGFBR3*	intronic	LNG, LIV, SKIN	18 tissues	24 tissues	6 altered motifs
rs2182703	1	23026489	42,488	40,565	A>G	0.31	1.02	1.00	1.05	3.0E-02	*C1QB, EPHB2, MIR4684*			ESDR, BRN, GI	GI	Mrg1::Hoxa9,p300
rs2387407	10	6547909	40,872	38,549	G>A	0.13	1.03	1.00	1.07	3.0E-02	*PRKCQ*	intronic		BLD		Smad3,Smad,p53
rs6954897	7	22750220	42,497	40,571	G>A	0.43	1.02	1.00	1.04	3.0E-02	*IL6, LOC401312, LOC541472*			BLD	SKIN	
rs6426763	1	23068761	42,493	40,571	A>G	0.19	1.03	1.00	1.06	3.0E-02	*EPHB2, MIR4684*	intronic	VAS	ESDR		AP-1
rs1881457	5	131992409	42,169	40,259	A>C	0.21	0.97	0.95	1.00	3.0E-02	*IL4, IL13, RAD50, KIF3A*		10 tissues	18 tissues	4 tissues	4 altered motifs
rs3009935	1	218663189	42,498	40,568	G>A	0.40	0.98	0.96	1.00	3.0E-02	*TGFB2*		LNG	7 tissues	LNG	5 altered motifs
rs9663421	10	6055604	42,498	40,570	G>A	0.28	0.98	0.95	1.00	3.0E-02	*IL2RA, IL15RA*	intronic			OVRY,BLD,SKIN	Myc
rs2307101	15	40322460	42,486	40,542	G>A	0.07	1.04	1.00	1.09	3.1E-02	*SRP14, EIF2AK4, SRP14-AS1*	intronic	HRT	6 tissues	6 tissues	Mrg
rs1473488	1	92241746	42,501	40,569	A>G	0.46	1.02	1.00	1.04	3.1E-02	*TGFBR3*	intronic		14 tissues		Nanog
rs10874941	1	92242760	42,495	40,570	G>A	0.46	1.02	1.00	1.04	3.1E-02	*TGFBR3*	intronic				CEBPD,HMG-IY,STAT

SNP	Chr	Position[a]	N Cases	N Controls	Allele	MAF	OR[b]	95%CI		*p* -value	Gene annotation	dbSNP functional annotation	Promoter histone marks	Enhancer histone marks	DNAse	Motifs changed
rs12123363	1	92266836	42,504	40,572	G>A	0.32	1.02	1.00	1.05	3.1E-02	*TGFBR3*	intronic		7 tissues		
rs12949932	17	25970987	41,495	39,478	A>C	0.23	1.03	1.00	1.05	3.1E-02	*LGALS9, KSR1, NOS2P1, ITM2BP1, LOC100420408*	intronic		FAT, GI, MUS		NF-Y,Nanog,TATA
rs12726500	1	92296222	42,507	40,577	A>C	0.36	0.98	0.96	1.00	3.2E-02	*TGFBR3*	intronic	8 tissues	22 tissues	8 tissues	5 altered motifs
rs6604055	1	92247465	42,506	40,568	A>C	0.46	1.02	1.00	1.04	3.2E-02	*TGFBR3*	intronic	STRM	16 tissues	6 tissues	
rs11259097	10	6521534	42,313	40,382	G>A	0.19	0.97	0.95	1.00	3.2E-02	*PRKCQ*	intronic				
rs6604054	1	92244672	42,503	40,575	G>C	0.46	1.02	1.00	1.04	3.2E-02	*TGFBR3*	intronic		17 tissues	9 tissues	7 altered motifs
rs11804539	1	92279707	42,503	40,575	G>A	0.32	1.02	1.00	1.05	3.2E-02	*TGFBR3*	intronic		14 tissues	VAS	5 altered motifs
rs943445	10	6579062	42,502	40,568	T>A	0.35	1.02	1.00	1.04	3.2E-02	*PRKCQ, PRKCQ-AS1*	intronic		BLD, THYM		
rs6602747	10	6537456	42,506	40,571	A>G	0.05	0.95	0.91	1.00	3.3E-02	*PRKCQ*	intronic	BLD	5 tissues	9 tissues	BDP1,CTCF,SMC3
rs7125062	11	102663503	42,503	40,572	A>G	0.28	0.98	0.95	1.00	3.3E-02	*MMP1, MMP3, MMP10, CSNK1A1P2, WTAPP1, LOC100421658*	intronic			4 tissues	Zfp410
rs7916294	10	90744325	42,507	40,574	C>A	0.34	0.98	0.96	1.00	3.3E-02	*ACTA2, FAS, ACTA2-AS1, FAS-AS1*	intronic		4 tissues	BLD	RORalpha1,TCF12
rs1036096	3	30695091	42,474	40,532	G>A	0.13	1.03	1.00	1.06	3.3E-02	*TGFBR2*	intronic				GR,NRSF,Rad21
rs11894425	2	191837841	42,509	40,577	A>G	0.40	1.02	1.00	1.04	3.3E-02	*GLS, STAT1, LOC100420571*	intronic		7 tissues	5 tissues	RREB-1
rs8089829	18	60031904	42,177	40,216	A>G	0.45	0.98	0.96	1.00	3.3E-02	*TNFRSF11A*	intronic				8 altered motifs
rs41294663	10	6048900	42,507	40,577	A>G	0.11	1.03	1.00	1.07	3.3E-02	*IL2RA, IL15RA*					VDR
rs12543190	8	128777482	42,290	40,261	A>C	0.13	1.03	1.00	1.06	3.4E-02	*MYC, MIR1204*			4 tissues	CRVX	
rs4075015	1	154389196	42,498	40,569	A>T	0.41	1.02	1.00	1.04	3.4E-02	*IL6R, MRPS33P1, RPSAP17, PSMD8P1*	intronic		10 tissues	MUS	4 altered motifs
rs1556192	1	92283060	42,501	40,551	A>G	0.32	1.02	1.00	1.05	3.4E-02	*TGFBR3*	intronic		16 tissues	ADRL,LNG,OVRY	8 altered motifs
rs13395505	2	191838514	42,497	40,573	G>A	0.40	1.02	1.00	1.04	3.4E-02	*GLS, STAT1, LOC100420571*	intronic		8 tissues	ESDR,PLCNT	
rs7801617	7	22758082	42,510	40,576	G>A	0.10	0.97	0.93	1.00	3.5E-02	*IL6, LOC541472*		4 tissues	14 tissues	24 tissues	
rs13075948	3	30683506	42,502	40,574	G>A	0.29	0.98	0.96	1.00	3.5E-02	*TGFBR2*	intronic	BLD, GI	18 tissues	BLD	
rs9839010	3	38206906	42,471	40,562	A>G	0.45	0.98	0.96	1.00	3.5E-02	*ACAA1, MYD88, DLEC1, OXSR1*		24 tissues		53 tissues	
rs11165546	1	92280923	42,492	40,568	C>A	0.32	1.02	1.00	1.05	3.6E-02	*TGFBR3*	intronic		8 tissues		Crx,Gsc,Pitx3
rs13234273	7	18621925	42,504	40,573	G>A	0.10	0.96	0.93	1.00	3.6E-02	*HDAC9, LOC100419901*	intronic		BRN, HRT		7 altered motifs
rs2256774	10	6097165	42,501	40,562	A>G	0.34	0.98	0.96	1.00	3.6E-02	*IL2RA, RBM17, RPL32P23*	intronic		5 tissues	BLD,BLD	4 altered motifs
rs4789559	17	76218857	42,497	40,564	G>A	0.37	0.98	0.96	1.00	3.6E-02	*BIRC5, TK1, SYNGR2, AFMID, TMEM235, THA1P, LOC100996291*	intronic		4 tissues		GATA,Zfp105
rs1539399	1	218565324	42,500	40,569	G>A	0.35	0.98	0.96	1.00	3.6E-02	*TGFB2, LOC728463*	intronic		LNG		4 altered motifs
rs11466567	1	92263429	42,492	40,551	C>A	0.25	1.02	1.00	1.05	3.6E-02	*TGFBR3*	intronic		10 tissues	PLCNT	Arid5b
rs2245675	10	6095577	42,499	40,573	G>A	0.33	0.98	0.96	1.00	3.7E-02	*IL2RA, RBM17, RPL32P23*	intronic	BLD	11 tissues	BLD	Irf,Mef2,TATA
rs416624	1	92282764	42,509	40,574	A>G	0.36	0.98	0.96	1.00	3.7E-02	*TGFBR3*	intronic		12 tissues	LNG,MUS,OVRY	
rs284159	1	92271351	42,509	40,577	G>A	0.37	0.98	0.96	1.00	3.7E-02	*TGFBR3*	intronic	GI	19 tissues	8 tissues	
rs2810884	1	92294333	42,497	40,569	G>A	0.32	1.02	1.00	1.05	3.7E-02	*TGFBR3*	intronic	7 tissues	16 tissues	ESDR,MUS,PLCNT	LBP-9,Mrg1::Hoxa9,Pbx3
rs653810	11	69448294	42,498	40,564	G>A	0.41	0.98	0.96	1.00	3.8E-02	*CCND1, ORAOV1, LOC100996515*					8 altered motifs
rs4787423	16	27367334	42,484	40,546	A>G	0.14	0.97	0.94	1.00	3.8E-02	*IL4R, IL21R*	intronic		4 tissues	4 tissues	8 altered motifs
rs1192528	1	92257080	42,503	40,572	C>G	0.37	0.98	0.96	1.00	3.8E-02	*TGFBR3*	intronic	IPSC	20 tissues	ESDR,SKIN,HRT	
rs13092349	3	30691319	42,508	40,576	G>A	0.13	1.03	1.00	1.06	3.8E-02	*TGFBR2*	intronic	BLD	15 tissues	13 tissues	Smad3,Smad
rs11575022	5	131402015	42,492	40,571	A>C	0.06	0.96	0.92	1.00	3.9E-02	*CSF2, IL3*			ESDR, BLD	BLD	9 altered motifs
rs284153	1	92274420	42,489	40,561	A>C	0.37	0.98	0.96	1.00	3.9E-02	*TGFBR3*	intronic		11 tissues	PLCNT	Irf,MZF1::1-4
rs9850060	3	30663987	42,501	40,570	A>G	0.24	0.98	0.95	1.00	3.9E-02	*TGFBR2*	intronic	BLD	15 tissues	BLD	Pou2f2,Sox
rs3773624	3	30690658	42,508	40,576	C>A	0.13	1.03	1.00	1.06	3.9E-02	*TGFBR2*	intronic	BLD	21 tissues		9 altered motifs
rs5746009	1	12249568	42,506	40,575	A>C	0.10	0.96	0.93	1.00	4.0E-02	*TNFRSF8, TNFRSF1B, VPS13D, LOC390998, MIR4632*	intronic		9 tissues		Myf,NRSF,Zfx
rs7078273	10	6128547	42,491	40,562	C>A	0.40	1.02	1.00	1.04	4.0E-02	*IL2RA, RBM17, RPL32P23*		13 tissues	20 tissues	53 tissues	10 altered motifs
rs852251	5	35922462	42,505	40,572	G>A	0.16	0.97	0.95	1.00	4.0E-02	*IL7R, UGT3A1, CAPSL, LOC100506406*	intronic		THYM, BLD		GR,THAP1,YY1
rs11165595	1	92302541	41,823	39,634	G>A	0.36	0.98	0.96	1.00	4.1E-02	*TGFBR3*	intronic		4 tissues	MUS,MUS	Evi-1,Mef2
rs2489189	1	92331736	42,507	40,577	T>A	0.06	1.04	1.00	1.09	4.1E-02	*TGFBR3*	intronic		4 tissues		7 altered motifs
rs11590254	1	92316573	42,509	40,577	A>T	0.32	0.98	0.96	1.00	4.1E-02	*TGFBR3*	intronic	FAT, GI, LNG	17 tissues	4 tissues	8 altered motifs
rs9960450	18	60016875	42,507	40,575	A>G	0.06	1.05	1.00	1.09	4.1E-02	*TNFRSF11A, KIAA1468*	intronic				4 altered motifs
rs2005061	3	30690748	42,506	40,577	G>A	0.13	1.03	1.00	1.06	4.1E-02	*TGFBR2*	intronic	BLD	21 tissues	BLD	ERalpha-a,Esr2,SF1
rs6461396	7	19001691	42,503	40,571	A>G	0.41	0.98	0.96	1.00	4.1E-02	*HDAC9, NPM1P13*	intronic		7 tissues	6 tissues	4 altered motifs
rs17296526	10	33656119	42,506	40,572	A>G	0.10	0.97	0.93	1.00	4.2E-02	*NRP1*			MUS, SKIN		ZEB1
rs947712	1	218564881	42,508	40,577	G>A	0.35	0.98	0.96	1.00	4.2E-02	*TGFB2, LOC728463*	intronic		5 tissues		8 altered motifs
rs11466500	3	30692451	42,509	40,577	G>A	0.13	1.03	1.00	1.06	4.3E-02	*TGFBR2*	intronic		BLD		
rs3117099	6	32358270	42,457	40,497	G>A	0.22	0.98	0.95	1.00	4.3E-02	*HLA-DRA, C6orf10, BTNL2, HCG23*		ESDR	BLD, SKIN	BLD	
rs3793730	10	6522698	42,379	40,479	C>G	0.15	1.03	1.00	1.06	4.3E-02	*PRKCQ*	intronic			BLD	4 altered motifs
rs564799	3	159728987	42,506	40,576	G>A	0.42	0.98	0.96	1.00	4.3E-02	*IL12A*		BLD	BLD		
rs6966799	7	18104972	42,489	40,563	G>A	0.42	1.02	1.00	1.04	4.4E-02	*HDAC9, PRPS1L1*					NF-kappaB,Pou2f2
rs6604609	1	218568482	42,508	40,577	A>T	0.05	1.05	1.00	1.09	4.4E-02	*TGFB2, LOC728463*	intronic		6 tissues		
rs16902359	8	128742851	42,497	40,573	G>A	0.12	0.97	0.94	1.00	4.4E-02	*MYC*			ESDR		Mef2,NF-AT,YY1

SNP	Chr	Position[a]	N Cases	N Controls	Allele	MAF	OR[b]	95%CI		*p* -value	Gene annotation	dbSNP functional annotation	Promoter histone marks	Enhancer histone marks	DNAse	Motifs changed
rs2082225	3	30698392	42,508	40,573	A>G	0.13	1.03	1.00	1.06	4.5E-02	*TGFBR2*	intronic		17 tissues		
rs3773627	3	30694413	42,498	40,565	C>A	0.13	1.03	1.00	1.06	4.5E-02	*TGFBR2*	intronic		BLD, VAS		TCF4
rs944722	17	26092037	41,751	39,954	A>G	0.40	1.02	1.00	1.04	4.5E-02	*NOS2, LOC645754*	intronic		LIV, MUS		RXRA,Smad
rs2704284	7	18645628	42,500	40,565	G>C	0.49	1.02	1.00	1.04	4.5E-02	*HDAC9, LOC100419901*	intronic				
rs10739778	9	101875789	42,506	40,572	A>C	0.35	1.02	1.00	1.04	4.5E-02	*COL15A1, TGFBR1*	intronic			7 tissues	15 altered motifs
rs1019855	3	30697661	42,506	40,573	A>G	0.13	1.03	1.00	1.06	4.5E-02	*TGFBR2*	intronic			MUS,SKIN	GR,Mef2,Nanog
rs6770038	3	30650327	42,507	40,575	G>A	0.18	0.97	0.95	1.00	4.5E-02	*TGFBR2*	intronic	18 tissues	7 tissues	6 tissues	Brachyury
rs10493859	1	92273644	41,964	39,977	C>A	0.32	1.02	1.00	1.04	4.6E-02	*TGFBR3*	intronic				6 altered motifs
rs12959396	18	60039309	42,506	40,575	A>C	0.47	0.98	0.96	1.00	4.6E-02	*TNFRSF11A, RPL17P44*	intronic		5 tissues	BRN	4 altered motifs
rs12490421	3	30698821	42,508	40,577	G>A	0.13	1.03	1.00	1.06	4.6E-02	*TGFBR2*	intronic	BLD	20 tissues	BLD,MUS	NF-I
rs10246722	7	18965590	42,466	40,503	A>G	0.23	1.02	1.00	1.05	4.7E-02	*HDAC9, NPM1P13*	intronic				
rs7794241	7	18913380	42,135	40,100	C>A	0.36	1.02	1.00	1.04	4.7E-02	*HDAC9*	intronic			ESC,SKIN,SKIN	35 altered motifs
rs6476982	9	5498516	42,504	40,570	C>A	0.28	1.02	1.00	1.05	4.7E-02	*CD274, PDCD1LG2*			BLD, VAS	SKIN	HNF4
rs12490466	3	30698977	42,506	40,577	G>A	0.13	1.03	1.00	1.06	4.7E-02	*TGFBR2*	intronic	BLD	20 tissues	BLD	Pax-5
rs2237060	5	131970885	42,393	40,441	A>C	0.41	1.02	1.00	1.04	4.8E-02	*IL4, IL13, RAD50*	intronic		BLD		Bbx,Duxl,Hbp1
rs1800925	5	131992809	42,497	40,570	G>A	0.20	0.98	0.95	1.00	4.8E-02	*IL4, IL13, RAD50, KIF3A*			6 tissues		4 altered motifs
rs4335431	1	218557609	42,508	40,575	A>G	0.09	0.97	0.93	1.00	4.8E-02	*TGFB2, RRP15, RPS26P17, LOC728463*	intronic		7 tissues		
rs10510636	3	30698096	42,508	40,577	G>A	0.13	1.03	1.00	1.06	4.8E-02	*TGFBR2*	intronic				4 altered motifs
rs41294605	10	6032917	42,489	40,546	G>A	0.13	1.03	1.00	1.06	4.9E-02	*IL2RA, IL15RA*					6 altered motifs
rs3782176	12	88939133	42,508	40,558	G>A	0.09	0.97	0.93	1.00	4.9E-02	*KITLG*	intronic	SKIN	10 tissues	10 tissues	Mef2
rs1323657	10	6072427	42,500	40,572	G>A	0.44	0.98	0.96	1.00	4.9E-02	*IL2RA, RPL32P23*	intronic				5 altered motifs
rs7226420	18	60060545	42,505	40,573	G>A	0.27	0.98	0.96	1.00	4.9E-02	*ACTBP9, TNFRSF11A, RPL17P44*			ESC		EBF
rs17025857	3	30681095	42,508	40,577	A>G	0.29	0.98	0.96	1.00	4.9E-02	*TGFBR2*	intronic	BLD	9 tissues		ZEB1
rs485789	3	159730148	42,509	40,577	C>A	0.42	0.98	0.96	1.00	4.9E-02	*IL12A*		BLD	4 tissues	4 tissues	4 altered motifs
rs62449495	7	22764338	42,505	40,574	G>A	0.19	1.03	1.00	1.05	4.9E-02	*IL6, RPS26P32, LOC541472*			12 tissues	BLD	
rs11129420	3	30658541	42,503	40,571	A>T	0.47	0.98	0.96	1.00	5.0E-02	*TGFBR2*	intronic	BLD	13 tissues	BLD	COMP1
rs7619345	3	3138841	42,497	40,575	G>A	0.33	1.02	1.00	1.04	5.0E-02	*IL5RA, TRNT1, CNTN4*	intronic		ESDR, BLD		ELF1,PRDM1,PU.1
rs284142	1	92256626	42,375	40,323	A>G	0.37	0.98	0.96	1.00	5.0E-02	*TGFBR3*	intronic	IPSC	17 tissues		7 altered motifs
rs11259531	10	6595558	41,849	39,682	T>A	0.15	0.97	0.94	1.00	5.0E-02	*PRKCQ, PRKCQ-AS1*	intronic		BLD		11 altered motifs
rs11597105	10	6512581	42,502	40,568	G>A	0.15	1.03	1.00	1.06	5.1E-02	*PRKCQ*	intronic		BLD		4 altered motifs
rs3730358	14	105246407	42,485	40,556	G>A	0.14	1.03	1.00	1.06	5.2E-02	*AKT1, SIVA1, ADSSL1, LINC00638, ZBTB42*	intronic		ESDR, IPSC, SKIN	BLD	
rs2156209	18	60064536	42,461	40,539	G>A	0.07	0.96	0.92	1.00	5.2E-02	*ACTBP9, TNFRSF11A, RPL17P44*					Hic1
rs2715274	3	121781723	42,478	40,547	A>G	0.19	0.98	0.95	1.00	5.2E-02	*CD86, ILDR1*	intronic		BLD		8 altered motifs
rs2297201	20	44684978	42,509	40,575	G>A	0.06	0.96	0.92	1.00	5.2E-02	*MMP9, SLC12A5, NCOA5, RPL13P2, LOC100128028*	intronic		IPSC, BRN	BRN,LIV	Myb
rs732222	17	25973203	42,079	40,062	G>A	0.24	1.02	1.00	1.05	5.2E-02	*LGALS9, KSR1, NOS2P1, ITM2BP1, LOC100420408*	intronic		4 tissues		MZF1::1-4
rs4955212	3	30669358	42,476	40,508	G>A	0.24	0.98	0.95	1.00	5.3E-02	*TGFBR2*	intronic	BLD	15 tissues	SKIN	Isl2,Myb
rs12027931	1	92260160	42,486	40,544	A>G	0.09	1.03	1.00	1.07	5.3E-02	*TGFBR3*	intronic		FAT		Pou3f2,STAT,TATA
rs1547550	2	191845725	42,503	40,572	G>C	0.34	1.02	1.00	1.04	5.3E-02	*GLS, STAT1, STAT4, LOC100420571*	intronic	VAS	14 tissues	ESDR,THYM	7 altered motifs
rs12516866	5	35851261	42,234	40,364	C>A	0.41	1.02	1.00	1.04	5.3E-02	*IL7R, SPEF2*		14 tissues	5 tissues	SKIN	6 altered motifs
rs7550856	1	92263776	42,502	40,574	T>A	0.09	1.03	1.00	1.07	5.4E-02	*TGFBR3*	intronic	FAT	14 tissues	HRT	Esr2,HNF4,Hic1
rs6752254	2	219058743	42,499	40,576	A>G	0.49	0.98	0.96	1.00	5.4E-02	*CXCR1, ARPC2, HMGB1P9*			BLD, MUS		12 altered motifs
rs9831477	3	30693522	42,494	40,567	T>A	0.42	1.02	1.00	1.04	5.5E-02	*TGFBR2*	intronic		5 tissues		Zbtb3
rs585881	10	6558269	42,507	40,575	A>G	0.19	1.03	1.00	1.05	5.5E-02	*PRKCQ*	intronic		6 tissues	ESDR,BLD	Mrg,Tgif1
rs17047804	1	218582778	42,510	40,574	A>G	0.12	0.97	0.94	1.00	5.5E-02	*TGFB2*	intronic		8 tissues		AFP1,Mef2
rs4804056	19	8027762	42,270	40,326	G>A	0.46	1.02	1.00	1.04	5.6E-02	*ELAVL1, MAP2K7, SNAPC2, TIMM44, CTXN1, TGFBR3L*	3'-UTR	LIV	ESDR, LNG, LIV	LIV	BHLHE40,p300
rs428815	1	92260920	42,509	40,575	G>A	0.09	1.03	1.00	1.07	5.6E-02	*TGFBR3*	intronic	FAT, STRM, GI	12 tissues	ADRL,MUS	9 altered motifs
rs519951	10	6558340	42,506	40,576	G>A	0.19	1.02	1.00	1.05	5.7E-02	*PRKCQ*	intronic		6 tissues	4 tissues	
rs155106	2	182351352	42,494	40,563	A>C	0.41	1.02	1.00	1.04	5.7E-02	*ITGA4*	intronic				Pax-4,Pou2f2
rs17131560	1	92254485	42,018	39,790	A>G	0.09	1.04	1.00	1.07	5.7E-02	*TGFBR3*	intronic	LNG	14 tissues	11 tissues	5 altered motifs
rs2158177	5	131984058	42,510	40,576	A>G	0.20	0.98	0.95	1.00	5.8E-02	*IL4, IL13, RAD50, KIF3A*			LIV		
rs1805034	18	60027241	42,503	40,569	A>G	0.49	1.02	1.00	1.04	5.8E-02	*TNFRSF11A*	missense		SKIN	SKIN	Osf2
rs614394	11	69419268	42,508	40,573	A>G	0.08	1.04	1.00	1.08	5.8E-02	*CCND1, LOC100996515*			ESC, IPSC, LIV	GI,PLCNT	6 altered motifs
rs284164	1	92269067	42,500	40,577	G>A	0.09	1.03	1.00	1.07	5.8E-02	*TGFBR3*	intronic	BLD	17 tissues	8 tissues	ATF4,ZID
rs6862142	5	35882296	42,510	40,577	A>G	0.42	1.02	1.00	1.04	5.8E-02	*IL7R, CAPSL*			5 tissues	SKIN,CRVX	TAL1
rs2069861	7	22771654	41,253	39,367	G>A	0.10	0.97	0.94	1.00	5.8E-02	*IL6, RPS26P32, LOC541472*				ESDR,LNG	11 altered motifs
rs4284478	12	88907563	42,315	40,352	G>A	0.19	0.98	0.95	1.00	5.9E-02	*KITLG*	intronic				8 altered motifs
rs1326266	1	198589576	42,386	40,418	G>A	0.20	1.02	1.00	1.05	5.9E-02	*PTPRC*		BLD	BLD, THYM	6 tissues	Pax-8,Pou2f2
rs12983784	19	8025491	42,485	40,564	A>G	0.25	0.98	0.96	1.00	5.9E-02	*ELAVL1, MAP2K7, SNAPC2, TIMM44, CTXN1, TGFBR3L*	3'-UTR		ESC, BLD, SKIN		INSM1

SNP	Chr	Position[a]	N Cases	N Controls	Allele	MAF	OR[b]	95%CI		*p*-value	Gene annotation	dbSNP functional annotation	Promoter histone marks	Enhancer histone marks	DNAse	Motifs changed
rs7808451	7	19002886	42,499	40,569	A>C	0.40	0.98	0.96	1.00	6.0E-02	*HDAC9, NPM1P13*	intronic				STAT
rs2243268	5	132013963	42,506	40,576	A>C	0.16	0.97	0.95	1.00	6.0E-02	*IL4, IL13, RAD50, KIF3A*	intronic		6 tissues		
rs569158	10	6562879	42,504	40,574	C>A	0.19	1.02	1.00	1.05	6.0E-02	*PRKCQ*	intronic		6 tissues	SKIN,THYM	
rs6699474	1	67859615	42,502	40,575	G>A	0.29	1.02	1.00	1.04	6.0E-02	*IL12RB2, SERBP1*	intronic	MUS	11 tissues	HRT,MUS,SKIN	5 altered motifs
rs752118	20	44746738	42,503	40,572	G>A	0.25	1.02	1.00	1.05	6.0E-02	*CD40, NCOA5, RPL13P2*		19 tissues	BRN	34 tissues	SP1
rs7072398	10	6079846	42,507	40,574	G>A	0.47	0.98	0.96	1.00	6.1E-02	*IL2RA, RPL32P23*	intronic	BLD	4 tissues	BLD,BLD	
rs541709	10	6562198	42,480	40,559	G>A	0.19	1.02	1.00	1.05	6.1E-02	*PRKCQ*	intronic		5 tissues		GATA,TATA
rs2243267	5	132013886	42,497	40,572	G>C	0.16	0.97	0.95	1.00	6.1E-02	*IL4, IL13, RAD50, KIF3A*	intronic		6 tissues		Foxc1,Mrg1::Hoxa9,Osr
rs12569180	1	92332419	42,503	40,573	G>C	0.31	0.98	0.96	1.00	6.1E-02	*TGFBR3*	intronic	SKIN, GI	13 tissues	MUS	5 altered motifs
rs7762161	6	167560272	42,505	40,576	C>G	0.31	0.98	0.96	1.00	6.1E-02	*CCR6, GPR31, TCP10L2*		5 tissues	7 tissues	BRN	Foxa,RBP-Jkappa,RREB-1
rs1835538	3	30655481	42,496	40,569	G>A	0.48	0.98	0.96	1.00	6.2E-02	*TGFBR2*	intronic	FAT, BLD	14 tissues		DMRT2,Smad
rs11799400	1	92258103	42,509	40,575	G>A	0.09	1.03	1.00	1.07	6.2E-02	*TGFBR3*	intronic	SKIN, GI	21 tissues	6 tissues	4 altered motifs
rs1939008	11	102656423	42,502	40,572	G>A	0.30	0.98	0.96	1.00	6.2E-02	*MMP1, MMP10, CSNK1A1P2, WTAPP1, LOC100421658*	intronic				HMG-IY
rs2236338	14	25100282	42,505	40,574	A>G	0.23	0.98	0.95	1.00	6.2E-02	*GZMH, GZMB*	missense	BLD	9 tissues	11 tissues	
rs4357599	10	6566975	42,506	40,575	A>G	0.42	1.02	1.00	1.04	6.2E-02	*PRKCQ*	intronic		BLD		7 altered motifs
rs2498804	14	105233095	42,499	40,571	C>A	0.32	1.02	1.00	1.04	6.2E-02	*AKT1, SIVA1, INF2, ADSSL1, ZBTB42, LOC100996409*		ESC, IPSC, LIV	ADRL	BLD,PLCNT,LIV	Nkx2,Nkx3
rs7899538	10	6059898	42,415	40,501	C>A	0.13	1.03	1.00	1.06	6.2E-02	*IL2RA, IL15RA*	intronic		4 tissues	GI,GI	5 altered motifs
rs10908195	11	69431416	42,500	40,572	G>A	0.30	1.02	1.00	1.04	6.2E-02	*CCND1, ORAOV1, LOC100996515*					RXR::LXR,RXRA,TCF4
rs6449195	4	15814814	42,505	40,570	A>G	0.11	1.03	1.00	1.07	6.2E-02	*CD38*	intronic				HNF4,PPAR
rs2634036	1	92290111	42,508	40,577	A>G	0.09	1.03	1.00	1.07	6.2E-02	*TGFBR3*	intronic		BLD, LIV		Hbp1
rs17137421	10	6556492	42,506	40,570	G>A	0.13	0.97	0.94	1.00	6.2E-02	*PRKCQ*	intronic	MUS	4 tissues	MUS	SIX5
rs2290610	3	3139957	42,505	40,574	A>G	0.34	1.02	1.00	1.04	6.2E-02	*IL5RA, TRNT1, CNTN4*	missense		BLD		
rs334353	9	101908365	42,433	40,520	A>C	0.24	1.02	1.00	1.05	6.3E-02	*TGFBR1*	intronic				Foxp1
rs2031613	10	90766924	42,502	40,567	A>G	0.30	0.98	0.96	1.00	6.3E-02	*ACTA2, FAS, FAS-AS1*	intronic		4 tissues	GI,GI,GI	Foxj2,HNF1,Pax-4
rs284161	1	92269982	42,508	40,575	C>A	0.09	1.03	1.00	1.07	6.3E-02	*TGFBR3*	intronic	4 tissues	20 tissues	8 tissues	
rs6065925	20	44734296	42,500	40,568	G>A	0.25	1.02	1.00	1.05	6.3E-02	*CD40, SLC12A5, NCOA5, RPL13P2*					Evi-1,GATA
rs17007653	4	142666472	41,093	39,108	A>C	0.09	0.97	0.93	1.00	6.3E-02	*IL15*					Nkx3,Nkx6-1
rs334348	9	101912471	42,508	40,574	A>G	0.25	1.02	1.00	1.05	6.3E-02	*TGFBR1*	3'-UTR	BLD	5 tissues		10 altered motifs
rs2520362	7	18863899	42,507	40,572	A>C	0.49	0.98	0.96	1.00	6.3E-02	*HDAC9*	intronic				AP-1,p53
rs284160	1	92270070	42,507	40,576	G>A	0.09	1.03	1.00	1.07	6.3E-02	*TGFBR3*	intronic	BLD, SKIN, GI	22 tissues	9 tissues	CEBPB
rs1352438	1	92312060	42,503	40,576	C>G	0.09	1.03	1.00	1.07	6.4E-02	*TGFBR3*	intronic	SKIN, LNG, CRVX	17 tissues	5 tissues	
rs901917	1	92292407	42,504	40,575	A>G	0.09	1.03	1.00	1.07	6.4E-02	*TGFBR3*	intronic	LIV	12 tissues	HRT	9 altered motifs
rs6131015	20	44737650	42,507	40,576	G>A	0.25	1.02	1.00	1.05	6.4E-02	*CD40, SLC12A5, NCOA5, RPL13P2*		BLD	BLD, GI, PANC	BLD,BLD	7 altered motifs
rs10789229	1	67705574	42,247	40,420	A>G	0.41	0.98	0.96	1.00	6.4E-02	*IL23R, LOC100130497*	intronic			BLD,BLD	Pou2f2,TATA
rs2681411	3	121785631	42,458	40,548	A>G	0.40	0.98	0.96	1.00	6.4E-02	*CD86, ILDR1*	intronic	BLD	ESDR, BLD	4 tissues	
rs7802442	7	22736446	42,480	40,557	A>C	0.29	1.02	1.00	1.04	6.5E-02	*IL6, LOC401312, LOC541472*		BLD, SKIN	12 tissues	19 tissues	4 altered motifs
rs10815239	9	5497346	42,443	40,518	C>A	0.41	1.02	1.00	1.04	6.5E-02	*CD274, PDCD1LG2*					5 altered motifs
rs2799547	1	92308567	42,510	40,577	G>A	0.09	1.03	1.00	1.07	6.5E-02	*TGFBR3*	intronic		9 tissues		7 altered motifs
rs734244	5	132010726	42,426	40,475	G>A	0.16	0.97	0.95	1.00	6.5E-02	*IL4, IL13, RAD50, KIF3A*	intronic	10 tissues	11 tissues	11 tissues	NRSF,Zfp691
rs17078449	3	45980673	42,509	40,576	G>A	0.21	1.02	1.00	1.05	6.5E-02	*CXCR6, CCR9, FYCO1*	intronic		18 tissues	15 tissues	FXR,Pbx-1,Zbtb12
rs2070874	5	132009710	42,507	40,575	G>A	0.16	0.97	0.95	1.00	6.5E-02	*IL4, IL13, RAD50, KIF3A*	5'-UTR		12 tissues	ESDR,BLD	15 altered motifs
rs1389832	5	35858721	42,474	40,524	G>A	0.32	0.98	0.96	1.00	6.6E-02	*IL7R, SPEF2, CAPSL*	intronic	6 tissues	9 tissues		9 altered motifs
rs9842595	3	45922691	42,507	40,575	C>A	0.08	1.03	1.00	1.07	6.6E-02	*CCR9, SDHDP4, LZTFL1, FYCO1*		BLD	9 tissues	15 tissues	
rs16970325	15	40373131	42,509	40,577	G>A	0.09	1.03	1.00	1.07	6.6E-02	*SRP14, BMF, EIF2AK4, SRP14-AS1*		10 tissues	20 tissues	15 tissues	Irf,PU.1,Pax-6
rs6959028	7	18806051	42,441	40,473	C>A	0.42	1.02	1.00	1.04	6.6E-02	*HDAC9*	intronic				4 altered motifs
rs10080	10	33467108	42,502	40,563	G>A	0.41	1.02	1.00	1.04	6.6E-02	*NRP1*	3'-UTR			9 tissues	
rs982764	10	90769998	42,502	40,576	A>G	0.31	1.02	1.00	1.04	6.6E-02	*ACTA2, FAS, FAS-AS1*	intronic				5 altered motifs
rs2129973	1	92305082	42,508	40,571	G>A	0.09	1.03	1.00	1.07	6.7E-02	*TGFBR3*	intronic		5 tissues		ATF3
rs6461392	7	18912802	41,822	39,926	G>A	0.36	1.02	1.00	1.04	6.7E-02	*HDAC9*	intronic				8 altered motifs
rs10268180	7	19019671	42,501	40,566	A>G	0.39	1.02	1.00	1.04	6.7E-02	*HDAC9, NPM1P13*	intronic				
rs12165104	18	60050641	42,502	40,568	G>A	0.27	0.98	0.96	1.00	6.8E-02	*TNFRSF11A, RPL17P44*	intronic		LIV		Ets,RFX5,TFE
rs11165679	1	92329059	42,508	40,574	G>A	0.31	0.98	0.96	1.00	6.8E-02	*TGFBR3*	intronic	FAT	9 tissues		RFX5
rs204991	6	32161366	42,503	40,570	A>G	0.19	0.98	0.95	1.00	6.8E-02	*AGER, NOTCH4, PBX2, RNF5, PPT2, AGPAT1, GPSM3, PRRT1, EGFL8, LOC100507547, PPT2-EGFL8*	intronic	4 tissues	13 tissues		
rs2254993	7	18647887	41,452	39,370	G>A	0.12	0.97	0.94	1.00	6.8E-02	*HDAC9, LOC100419901*	intronic				GR
rs2489188	1	92332489	42,498	40,565	A>G	0.34	0.98	0.96	1.00	6.8E-02	*TGFBR3*	intronic	SKIN, GI	13 tissues	MUS,MUS	Pax-8
rs1494560	5	35860637	42,497	40,560	G>A	0.32	0.98	0.96	1.00	6.8E-02	*IL7R, SPEF2, CAPSL*	intronic	BLD, GI, THYM	10 tissues	SKIN	p300
rs12364496	11	69434379	42,507	40,570	G>A	0.29	1.02	1.00	1.04	6.9E-02	*CCND1, ORAOV1, LOC100996515*				6 tissues	NF-E2,Pax-4
rs284148	1	92277843	42,508	40,575	G>A	0.09	1.03	1.00	1.07	6.9E-02	*TGFBR3*	intronic	BRST	9 tissues		PLAG1

SNP	Chr	Position[a]	N Cases	N Controls	Allele	MAF	OR[b]	95%CI		p -value	Gene annotation	dbSNP functional annotation	Promoter histone marks	Enhancer histone marks	DNAse	Motifs changed
rs10874980	1	92283899	42,506	40,568	G>A	0.09	1.03	1.00	1.07	6.9E-02	*TGFBR3*	intronic			14 tissues	E2F,Maf,TCF12
rs2243266	5	132013789	42,507	40,575	G>A	0.16	0.97	0.95	1.00	6.9E-02	*IL4, IL13, RAD50, KIF3A*	intronic		11 tissues		9 altered motifs
rs1494561	5	35857235	42,288	40,408	G>C	0.32	0.98	0.96	1.00	6.9E-02	*IL7R, SPEF2, CAPSL*	intronic	12 tissues	8 tissues	18 tissues	
rs11596355	10	6064181	42,503	40,572	A>G	0.09	0.97	0.93	1.00	6.9E-02	*IL2RA, IL15RA, RPL32P23*	intronic		BRN, PLCNT		Sin3Ak-20
rs6074024	20	44743310	42,502	40,567	A>G	0.25	1.02	1.00	1.05	6.9E-02	*CD40, NCOA5, RPL13P2*					BRCA1,Pou2f2
rs2391070	1	92292765	42,508	40,577	G>A	0.09	1.03	1.00	1.07	7.0E-02	*TGFBR3*	intronic	4 tissues	14 tissues	19 tissues	Hic1,TCF4
rs12527778	6	86254522	42,076	39,457	A>G	0.12	0.97	0.94	1.00	7.0E-02	*NT5E, SNX14*	intronic		MUS, BONE		8 altered motifs
rs2799516	1	92312764	42,507	40,577	G>A	0.09	1.03	1.00	1.07	7.0E-02	*TGFBR3*	intronic		12 tissues		Foxl1,Nkx6-2,TATA
rs12751910	1	92243307	42,508	40,574	G>A	0.30	1.02	1.00	1.04	7.0E-02	*TGFBR3*	intronic	SKIN	15 tissues	SKIN	Irf
rs78456079	11	69420993	42,416	40,510	G>A	0.07	1.04	1.00	1.08	7.1E-02	*CCND1, LOC100996515*			LIV		HDAC2
rs10490939	10	33534880	42,487	40,553	G>A	0.16	1.03	1.00	1.05	7.1E-02	*NRP1*	intronic			IPSC	
rs901913	1	92293162	42,508	40,577	C>A	0.09	1.03	1.00	1.07	7.1E-02	*TGFBR3*	intronic	5 tissues	12 tissues	7 tissues	Pou2f2
rs4234661	3	119288872	42,504	40,569	A>G	0.43	0.98	0.96	1.00	7.1E-02	*ADPRH, CD80, CSRP2P, TIMMDC1, PLA1A*		BLD	BLD, PLCNT, SPLN	BLD,BLD	Msx-1,p53
rs4658269	1	92247654	42,500	40,569	C>A	0.30	1.02	1.00	1.04	7.2E-02	*TGFBR3*	intronic	SKIN	19 tissues	9 tissues	NF-Y
rs10197010	2	204740171	42,508	40,576	A>C	0.19	1.02	1.00	1.05	7.2E-02	*CTLA4*					Myc,TCF4,TFE
rs7948546	11	69429994	42,354	40,370	A>G	0.29	1.02	1.00	1.04	7.2E-02	*CCND1, LOC100996515*			4 tissues	LIV	AP-1,Zfx,Znf143
rs1466691	12	88946871	42,509	40,573	G>A	0.09	0.97	0.94	1.00	7.2E-02	*KITLG*	intronic	FAT, LNG	12 tissues	11 tissues	Dobox4
rs1669348	3	3189398	42,502	40,563	G>A	0.34	1.02	1.00	1.04	7.2E-02	*IL5RA, TRNT1, CRBN*	intronic				
rs3093002	6	167554060	42,507	40,576	G>A	0.32	0.98	0.96	1.00	7.2E-02	*CCR6, GPR31, TCP10L2*			7 tissues		
rs172687	6	86112491	42,504	40,574	G>A	0.11	0.97	0.94	1.00	7.3E-02	*NT5E, LOC643870, DUTP5*			21 tissues		NF-kappaB,RBP-Jkappa
rs1854696	9	21435972	42,459	40,499	G>A	0.08	1.03	1.00	1.07	7.3E-02	*IFNA1, IFNA8, IFNA11P, IFNWP19, IFNA12P, IFNE, MIR31HG, IFNWP2*					GR,Irx,Zfp105
rs6604053	1	92244566	42,504	40,576	C>A	0.30	1.02	1.00	1.04	7.3E-02	*TGFBR3*	intronic		15 tissues	6 tissues	6 altered motifs
rs4849124	2	113576902	42,504	40,574	G>A	0.31	0.98	0.96	1.00	7.3E-02	*IL1A, IL1B, LOC100128413*			ESDR, LIV, PANC		
rs2799522	1	92306976	42,506	40,566	G>C	0.09	1.03	1.00	1.07	7.3E-02	*TGFBR3*	intronic		12 tissues	8 tissues	5 altered motifs
rs17368659	11	102742761	42,476	40,531	C>A	0.14	0.97	0.95	1.00	7.4E-02	*MMP3, MMP12, WTAPP1, LOC100288111*	intronic				Pou1f1,TEF
rs10752175	10	6061781	42,508	40,577	G>A	0.08	0.97	0.93	1.00	7.4E-02	*IL2RA, IL15RA*	intronic				9 altered motifs
rs4143815	9	5468257	42,499	40,569	G>C	0.28	1.02	1.00	1.04	7.4E-02	*CD274, PLGRKT, PDCD1LG2, LOC100419687*	3'-UTR			BLD	7 altered motifs
rs2799103	1	218584419	42,504	40,576	G>A	0.22	0.98	0.95	1.00	7.4E-02	*TGFB2*	intronic		5 tissues	BRN	5 altered motifs
rs10819639	9	101894361	42,501	40,570	A>C	0.24	1.02	1.00	1.05	7.4E-02	*TGFBR1*	intronic				10 altered motifs
rs10493857	1	92334510	42,507	40,575	G>A	0.31	0.98	0.96	1.00	7.4E-02	*TGFBR3*	intronic				CTCF,Rad21
rs204990	6	32161430	42,430	40,511	C>A	0.19	0.98	0.95	1.00	7.5E-02	*AGER, NOTCH4, PBX2, RNF5, PPT2, AGPAT1, GPSM3, PRRT1, EGFL8, LOC100507547, PPT2-EGFL8*	intronic	4 tissues	12 tissues	BLD	
rs13231656	7	18957681	42,500	40,568	A>G	0.22	1.02	1.00	1.05	7.5E-02	*HDAC9, NPM1P13*	intronic				HNF1,Hoxa5,Pou1f1
rs1590	9	101916165	42,502	40,570	A>C	0.25	1.02	1.00	1.04	7.5E-02	*TGFBR1*	3'-UTR		LNG		AP-2rep,RXRA,Rad21
rs1192524	1	92340684	42,503	40,574	G>A	0.32	0.98	0.96	1.00	7.6E-02	*TGFBR3*	intronic	BRST	7 tissues		BCL,NRSF
rs17443164	1	92246048	42,506	40,575	C>G	0.30	1.02	1.00	1.04	7.6E-02	*TGFBR3*	intronic		6 tissues		Hoxa13,PRDM1
rs11567737	5	35868497	42,498	40,564	A>G	0.32	0.98	0.96	1.00	7.6E-02	*IL7R, CAPSL*	intronic		BLD, THYM		Irf
rs7042852	9	101902827	42,501	40,564	T>A	0.24	1.02	1.00	1.05	7.6E-02	*TGFBR1*	intronic		10 tissues	BLD	
rs1178109	7	18744814	42,507	40,577	G>A	0.20	1.02	1.00	1.05	7.6E-02	*HDAC9*	intronic				CEBPB,STAT
rs2280235	2	191843830	42,456	40,531	A>G	0.24	1.02	1.00	1.05	7.6E-02	*GLS, STAT1, LOC100420571*	intronic		8 tissues		
rs3770120	2	182352469	42,504	40,568	A>G	0.30	1.02	1.00	1.04	7.7E-02	*ITGA4, CERKL*	intronic				5 altered motifs
rs1938901	11	102661665	42,502	40,574	G>A	0.30	0.98	0.96	1.00	7.7E-02	*MMP1, MMP3, MMP10, CSNK1A1P2, WTAPP1, LOC100421658*	intronic		GI	SKIN	CEBPG,STAT,p300
rs995030	12	88890671	42,510	40,576	G>A	0.19	0.98	0.95	1.00	7.8E-02	*KITLG*	3'-UTR		8 tissues	4 tissues	
rs3773651	3	30718532	42,508	40,575	A>G	0.06	0.96	0.92	1.00	7.8E-02	*TGFBR2, GADL1*	intronic		7 tissues		Irf,Pax-4
rs1915087	3	121838791	42,507	40,574	A>G	0.33	0.98	0.96	1.00	7.8E-02	*CD86*	3'-UTR				Evi-1,Gm397
rs10044838	5	35864590	42,460	40,539	A>G	0.32	0.98	0.96	1.00	7.8E-02	*IL7R, SPEF2, CAPSL*	intronic				Pax-5
rs275728	15	40192918	42,499	40,569	C>A	0.06	1.04	1.00	1.08	7.9E-02	*GPR176, EIF2AK4, LOC100505534*	intronic	IPSC	5 tissues	BRN	CTCF,GR
rs10482750	1	218556036	42,506	40,572	A>G	0.11	0.97	0.94	1.00	7.9E-02	*TGFB2, RRP15, RPS26P17, LOC728463*	intronic				Hdx,Mef2,STAT
rs6451226	5	35863244	42,509	40,577	G>A	0.32	0.98	0.96	1.00	8.0E-02	*IL7R, SPEF2, CAPSL*	intronic	BLD, LNG	BLD, SKIN		Foxj1,Foxq1
rs1400657	2	191833540	41,808	39,783	A>C	0.11	0.97	0.94	1.00	8.0E-02	*GLS, STAT1, LOC100420571*			STRM, PANC, MUS	MUS	6 altered motifs
rs12888409	14	62222018	42,445	40,519	T>A	0.29	1.02	1.00	1.04	8.0E-02	*HIF1A, SNAPC1, HIF1A-AS2*			9 tissues	24 tissues	AP-1,TR4
rs2796819	1	218580370	42,491	40,562	A>G	0.43	0.98	0.96	1.00	8.1E-02	*TGFB2*	intronic				Pou2f2,YY1
rs8177694	10	6003835	41,261	39,643	C>A	0.17	0.98	0.95	1.00	8.1E-02	*IL2RA, IL15RA, FBXO18*	intronic		ESDR, BRST, SPLN		RXRA
rs2494743	14	105251720	42,460	40,542	A>G	0.10	0.97	0.94	1.00	8.2E-02	*AKT1, SIVA1, ADSSL1, LINC00638, ZBTB42, RPS26P49*	intronic	BRN, GI	12 tissues	BLD	HNF4,NF-I
rs1633775	7	18651854	41,908	39,961	G>A	0.11	0.97	0.94	1.00	8.2E-02	*HDAC9, LOC100419901*	intronic				4 altered motifs
rs2634028	1	92320853	42,507	40,575	C>A	0.08	1.03	1.00	1.07	8.2E-02	*TGFBR3*	intronic		8 tissues	HRT,MUS	
rs12751814	1	67704863	42,503	40,571	A>G	0.41	0.98	0.96	1.00	8.2E-02	*IL23R, LOC100130497*	intronic				GR,Sox
rs4750517	10	6520458	42,503	40,573	A>G	0.32	1.02	1.00	1.04	8.3E-02	*PRKCQ*	intronic				

SNP	Chr	Position[a]	N Cases	N Controls	Allele	MAF	OR[b]	95%CI		*p* -value	Gene annotation	dbSNP functional annotation	Promoter histone marks	Enhancer histone marks	DNAse	Motifs changed
rs2510464	11	69434050	42,508	40,576	A>G	0.07	1.04	1.00	1.08	8.3E-02	*CCND1, ORAOV1, LOC100996515*				BLD	4 altered motifs
rs948995	11	69512089	42,504	40,577	G>A	0.07	0.97	0.93	1.00	8.3E-02	*CCND1, FGF19, ORAOV1, LOC100129779, LOC100996515*		CRVX	5 tissues	10 tissues	Maf
rs596866	10	6550199	42,467	40,498	A>G	0.19	1.02	1.00	1.05	8.3E-02	*PRKCQ*	intronic		BLD, BRN		NRSF,Zfp691
rs680753	11	102711581	42,420	40,475	C>G	0.14	0.98	0.95	1.00	8.3E-02	*MMP1, MMP3, MMP12, CSNK1A1P2, WTAPP1, LOC100288111*	intronic		GI		12 altered motifs
rs618020	10	6503450	42,509	40,574	T>A	0.11	1.03	1.00	1.06	8.3E-02	*PRKCQ*	intronic				
rs957971	17	40519925	42,502	40,570	C>G	0.36	1.02	1.00	1.04	8.3E-02	*STAT3, PTRF*	intronic	BLD, LNG	17 tissues		
rs4478839	1	198716504	42,495	40,572	A>G	0.36	1.02	1.00	1.04	8.5E-02	*PTPRC*	intronic		BLD		PTF1-beta,Pax-5
rs7217655	17	40496024	42,505	40,573	G>A	0.35	1.02	1.00	1.04	8.5E-02	*STAT3, STAT5A*	intronic		BLD	HRT	DMRT1,DMRT7,STAT
rs10733710	9	101907424	42,482	40,539	G>A	0.22	0.98	0.96	1.00	8.5E-02	*TGFBR1*	intronic			BLD	
rs10171839	2	219051314	42,497	40,570	A>G	0.50	0.98	0.96	1.00	8.5E-02	*CXCR1, CXCR2, ARPC2, HMGB1P9*				27 tissues	Pax-1,Zbtb12
rs2519889	7	18645296	42,506	40,575	C>G	0.46	1.02	1.00	1.04	8.5E-02	*HDAC9, LOC100419901*	intronic				4 altered motifs
rs309495	1	23238023	42,505	40,572	G>A	0.32	0.98	0.96	1.00	8.6E-02	*EPHB2, LACTBL1, MIR4253*	intronic				4 altered motifs
rs155110	2	182352791	42,449	40,502	G>A	0.31	1.02	1.00	1.04	8.6E-02	*ITGA4, CERKL*	intronic				Irx,Pou3f2,Sox
rs2291620	15	40328973	42,503	40,571	A>G	0.38	0.98	0.96	1.00	8.6E-02	*SRP14, EIF2AK4, SRP14-AS1*	intronic	HRT	11 tissues		
rs17368582	11	102738075	42,508	40,577	A>G	0.14	0.97	0.95	1.00	8.6E-02	*MMP3, MMP12, WTAPP1, LOC100288111*	synonymous		MUS		ERalpha-a,RXRA
rs574521	10	6556854	42,504	40,576	A>G	0.19	1.02	1.00	1.05	8.6E-02	*PRKCQ*	intronic	MUS	BLD, GI, THYM	MUS,BLD	Pbx3,RXRA,YY1
rs571715	10	6556554	42,506	40,575	A>G	0.19	1.02	1.00	1.05	8.6E-02	*PRKCQ*	intronic	MUS	4 tissues	MUS,LNG	
rs1494558	5	35861068	42,509	40,573	G>A	0.32	0.98	0.96	1.00	8.7E-02	*IL7R, SPEF2, CAPSL*	missense	GI	10 tissues	12 tissues	Mrg,Tgif1
rs2286596	12	6553055	42,496	40,568	A>G	0.31	0.98	0.96	1.00	8.8E-02	*CD27, VAMP1, MRPL51, TAPBPL, PKP2P1, RPL31P10, SRP14P1, CD27-AS1*	intronic	BLD, GI	BLD, THYM	IPSC,BLD	MIF-1,Nkx2,RFX5
rs7219739	17	40531761	42,465	40,547	C>A	0.36	1.02	1.00	1.04	8.8E-02	*STAT3, PTRF*	intronic	13 tissues	19 tissues	BLD,GI	Mef2
rs2182424	1	92107736	42,436	40,549	G>A	0.14	0.98	0.95	1.00	8.8E-02	*TGFBR3, HSP90B3P, RPL39P13*	intronic				
rs2717356	7	18975056	42,455	40,516	A>C	0.19	1.02	1.00	1.05	8.8E-02	*HDAC9, NPM1P13*	intronic				Irf
rs2227284	5	132012725	42,501	40,574	C>A	0.27	0.98	0.96	1.00	8.9E-02	*IL4, IL13, RAD50, KIF3A*	intronic	BLD	16 tissues	MUS	STAT
rs6893142	5	35863443	42,507	40,575	G>A	0.32	0.98	0.96	1.00	8.9E-02	*IL7R, SPEF2, CAPSL*	intronic	LNG	ESC, BLD, SKIN	BLD,BLD	DMRT5,Irf
rs9282801	17	26096473	42,508	40,576	C>A	0.35	0.98	0.96	1.00	8.9E-02	*NOS2, LOC645754*	intronic		MUS		GR,Gm397,Smad
rs2450926	1	92321512	42,509	40,577	A>G	0.09	1.03	1.00	1.07	8.9E-02	*TGFBR3*	intronic		BRST		GR
rs8042947	15	40325860	42,506	40,574	A>G	0.27	1.02	1.00	1.04	9.0E-02	*SRP14, EIF2AK4, SRP14-AS1*	intronic		HRT		GATA,Pax-4
rs1494556	5	35869037	42,470	40,548	A>C	0.32	0.98	0.96	1.00	9.0E-02	*IL7R, CAPSL*	intronic		BLD, THYM, BRST	GI	
rs2236337	14	25100247	42,504	40,572	A>G	0.22	0.98	0.96	1.00	9.0E-02	*GZMH, GZMB*	3'-UTR	BLD	9 tissues	17 tissues	4 altered motifs
rs3816769	17	40498273	42,506	40,571	A>G	0.36	1.02	1.00	1.04	9.0E-02	*STAT3, STAT5A*	intronic		12 tissues	ESDR	4 altered motifs
rs1476483	7	22731199	42,506	40,574	A>G	0.20	1.02	1.00	1.05	9.0E-02	*IL6, LOC401312, LOC541472*					CEBPG
rs10489628	1	67704107	42,510	40,574	G>A	0.39	0.98	0.96	1.00	9.1E-02	*IL23R, LOC100130497*	intronic				8 altered motifs
rs1805109	1	92327126	42,506	40,576	G>A	0.08	1.03	1.00	1.07	9.1E-02	*TGFBR3*	5'-UTR		BLD		
rs1907699	12	88889977	42,489	40,561	A>T	0.19	0.98	0.95	1.00	9.1E-02	*KITLG*	3'-UTR		GI, SKIN	BRN	Pou2f2
rs7711202	5	35866930	42,409	40,459	G>A	0.32	0.98	0.96	1.00	9.1E-02	*IL7R, CAPSL*	intronic	BLD	BLD		9 altered motifs
rs4842625	12	88888247	42,391	40,425	G>A	0.20	0.98	0.95	1.00	9.1E-02	*KITLG*	3'-UTR		6 tissues		9 altered motifs
rs11576557	1	92323809	42,500	40,567	G>A	0.10	1.03	1.00	1.06	9.2E-02	*TGFBR3*	intronic		BLD		AIRE,Znf143
rs1178111	7	18745723	42,507	40,576	A>C	0.21	1.02	1.00	1.05	9.2E-02	*HDAC9*	intronic				Hmbox1
rs4983384	14	105208057	42,468	40,514	G>A	0.32	1.02	1.00	1.04	9.2E-02	*AKT1, SIVA1, INF2, ADSSL1, LOC100996409*	intronic		4 tissues		5 altered motifs
rs2634025	1	92321974	42,499	40,572	G>A	0.08	1.03	0.99	1.07	9.2E-02	*TGFBR3*	intronic		BRST, BLD		4 altered motifs
rs7211777	17	40534075	42,505	40,575	A>G	0.36	1.02	1.00	1.04	9.2E-02	*STAT3, PTRF*	intronic	ESDR	11 tissues	7 tissues	Mef2
rs7647260	3	3136804	42,475	40,531	A>G	0.35	1.02	1.00	1.04	9.3E-02	*IL5RA, TRNT1, CNTN4*	intronic				5 altered motifs
rs2489186	1	92297055	42,507	40,575	G>A	0.09	1.03	1.00	1.07	9.3E-02	*TGFBR3*	intronic	GI, CRVX, LIV	13 tissues	4 tissues	AIRE,Foxq1,RREB-1
rs11746643	5	35833717	42,499	40,571	A>G	0.40	1.02	1.00	1.04	9.3E-02	*IL7R, SPEF2*		BLD	BLD, THYM		6 altered motifs
rs9891119	17	40507980	42,505	40,574	A>C	0.36	1.02	1.00	1.04	9.3E-02	*STAT3, STAT5A, PTRF*	intronic	BLD	11 tissues	BLD	GATA,Mef2,NR4A
rs7312974	12	88948872	42,457	40,485	A>G	0.20	0.98	0.95	1.00	9.4E-02	*KITLG*	intronic		8 tissues		5 altered motifs
rs10941267	5	35869818	42,509	40,575	A>G	0.32	0.98	0.96	1.00	9.5E-02	*IL7R, CAPSL*	intronic				Zfp410
rs2299007	5	132043032	42,496	40,571	A>G	0.15	0.98	0.95	1.00	9.5E-02	*IL4, IL13, KIF3A, SEPT8, CCNI2*	intronic		BRST, MUS		Mxi1,Myc
rs9292617	5	35877826	42,502	40,572	A>T	0.32	0.98	0.96	1.00	9.6E-02	*IL7R, CAPSL*			5 tissues	7 tissues	DMRT1,Pou1f1
rs754618	10	44886206	42,504	40,573	G>A	0.32	1.02	1.00	1.04	9.6E-02	*CXCL12, RPL9P21*			13 tissues	MUS,MUS,SKIN	
rs2129972	1	92305250	42,468	40,490	G>A	0.09	1.03	0.99	1.07	9.6E-02	*TGFBR3*	intronic		5 tissues	ADRL,HRT	4 altered motifs
rs17774098	8	128774649	42,481	40,511	G>A	0.15	0.98	0.95	1.00	9.6E-02	*MYC, MIR1204*			BLD, LNG		
rs1494555	5	35871190	42,498	40,572	A>G	0.32	0.98	0.96	1.00	9.6E-02	*IL7R, CAPSL*	missense		BLD, THYM		RXRA,p300
rs3773663	3	30730872	42,491	40,555	G>A	0.44	1.02	1.00	1.04	9.7E-02	*TGFBR2, GADL1*	intronic	11 tissues	16 tissues	26 tissues	BCL
rs3132947	6	32176782	42,507	40,573	C>A	0.20	0.98	0.95	1.00	9.7E-02	*AGER, NOTCH4, PBX2, RNF5, PPT2, AGPAT1, GPSM3, EGFL8, PPT2-EGFL8*	intronic		LIV		BATF,Bbx,Irf
rs626883	11	69430674	42,488	40,564	G>A	0.07	1.03	0.99	1.08	9.7E-02	*CCND1, ORAOV1, LOC100996515*		LIV	5 tissues	ESC,IPSC,LIV	4 altered motifs
rs2066804	2	191841759	42,497	40,568	G>A	0.24	1.02	1.00	1.04	9.8E-02	*GLS, STAT1, LOC100420571*	intronic				6 altered motifs
rs1473486	1	92241576	42,380	40,318	A>G	0.46	1.02	1.00	1.04	9.8E-02	*TGFBR3*	intronic		11 tissues		E2F,ERalpha-a

SNP	Chr	Position[a]	N Cases	N Controls	Allele	MAF	OR[b]	95%CI		p -value	Gene annotation	dbSNP functional annotation	Promoter histone marks	Enhancer histone marks	DNAse	Motifs changed
rs10063445	5	35870814	42,506	40,574	C>A	0.32	0.98	0.96	1.00	9.8E-02	*IL7R, CAPSL*	intronic				BDP1,MZF1::1-4
rs10486314	7	18915874	42,509	40,575	A>G	0.38	1.02	1.00	1.04	9.8E-02	*HDAC9*	intronic				5 altered motifs
rs9292616	5	35870588	42,504	40,576	G>A	0.32	0.98	0.96	1.00	9.8E-02	*IL7R, CAPSL*	intronic		4 tissues		PLZF,Pou1f1
rs10863396	1	218559032	42,500	40,571	A>G	0.31	0.98	0.96	1.00	9.8E-02	*TGFB2, RRP15, RPS26P17, LOC728463*	intronic				AIRE,ERalpha-a
rs2717351	7	19019880	42,504	40,574	A>G	0.22	0.98	0.96	1.00	9.9E-02	*HDAC9, NPM1P13*	intronic				4 altered motifs
rs3891248	8	128750139	42,382	40,470	A>T	0.14	0.98	0.95	1.00	9.9E-02	*MYC*	intronic	24 tissues		52 tissues	DMRT2,Foxo,Sox
rs1178110	7	18745001	42,503	40,576	A>G	0.20	1.02	1.00	1.05	9.9E-02	*HDAC9*	intronic				
rs646910	11	102709522	42,509	40,572	A>T	0.14	0.98	0.95	1.00	9.9E-02	*MMP1, MMP3, MMP12, CSNK1A1P2, WTAPP1, LOC100288111*	intronic		GI		Nanog
rs592412	11	69430194	42,509	40,576	G>A	0.07	1.03	0.99	1.08	9.9E-02	*CCND1, LOC100996515*			4 tissues		HP1-site-factor,NF-AT,Tgif1
rs2306581	17	40500265	42,436	40,511	C>A	0.36	1.02	1.00	1.04	9.9E-02	*STAT3, STAT5A*	intronic		18 tissues		
rs2046737	1	92326636	42,507	40,575	G>A	0.08	1.03	0.99	1.07	9.9E-02	*TGFBR3*	intronic		BLD		Bcl6b,Foxd3,Maf
rs4749894	10	6058323	42,508	40,569	A>G	0.25	0.98	0.96	1.00	1.0E-01	*IL2RA, IL15RA*	intronic		BLD, ADRL, THYM		Cdx2,GR,Gfi1
rs220488	17	3591822	42,470	40,554	A>G	0.15	1.02	1.00	1.05	1.0E-01	*CTNS, ITGAE, P2RX5, TAX1BP3, EMC6, GSG2, RPL21P125, P2RX5-TAX1BP3*	intronic		6 tissues	4 tissues	7 altered motifs
rs1178112	7	18746213	42,506	40,575	A>G	0.19	1.02	1.00	1.05	1.0E-01	*HDAC9*	intronic		BLD, HRT, GI	KID	Pax-6
rs4714696	6	43719993	42,503	40,563	A>G	0.29	0.98	0.96	1.00	1.0E-01	*VEGFA, LOC100132242*					5 altered motifs
rs3001371	14	105242831	42,442	40,520	G>A	0.31	1.02	1.00	1.04	1.0E-01	*AKT1, SIVA1, ADSSL1, LINC00638, ZBTB42*	intronic		5 tissues	MUS,BLD	RREB-1
rs605949	11	102705747	42,506	40,572	G>A	0.14	0.98	0.95	1.00	1.0E-01	*MMP1, MMP3, MMP12, CSNK1A1P2, WTAPP1, LOC100288111*	intronic		BRST, GI		7 altered motifs
rs647934	10	6534728	42,506	40,573	G>C	0.07	1.03	0.99	1.07	1.0E-01	*PRKCQ*	intronic	BLD, SKIN, BRST	BRST, BLD	BLD	4 altered motifs
rs2634019	1	92327877	42,508	40,577	T>A	0.08	1.03	0.99	1.07	1.0E-01	*TGFBR3*	intronic		FAT, BLD, CRVX		FAC1,Gfi1b,RREB-1
rs10074095	5	35868669	42,498	40,576	C>G	0.32	0.98	0.96	1.00	1.0E-01	*IL7R, CAPSL*	intronic				
rs1607595	4	142635648	42,503	40,572	G>A	0.14	1.02	1.00	1.05	1.0E-01	*IL15*	intronic	MUS	13 tissues	ESDR	
rs7914917	10	6575953	42,342	40,371	A>C	0.11	0.97	0.94	1.01	1.0E-01	*PRKCQ, PRKCQ-AS1*	intronic		BLD		5 altered motifs
rs7077367	10	6566735	42,504	40,576	A>G	0.22	1.02	1.00	1.04	1.0E-01	*PRKCQ*	intronic		BLD	THYM	10 altered motifs
rs801510	7	18665764	42,506	40,574	A>G	0.39	0.98	0.96	1.00	1.0E-01	*HDAC9, LOC100419901*	intronic		FAT		4 altered motifs
rs10777131	12	88962011	42,508	40,576	G>A	0.08	0.97	0.93	1.01	1.0E-01	*KITLG*	intronic			9 tissues	DMRT1
rs563096	11	102707366	42,501	40,571	A>T	0.14	0.98	0.95	1.00	1.0E-01	*MMP1, MMP3, MMP12, CSNK1A1P2, WTAPP1, LOC100288111*	intronic	ESC, IPSC	12 tissues	9 tissues	Foxa,STAT,p300
rs2227282	5	132013179	42,504	40,570	C>G	0.27	0.98	0.96	1.00	1.0E-01	*IL4, IL13, RAD50, KIF3A*	intronic		18 tissues	17 tissues	Myc,SREBP
rs3025000	6	43746169	42,510	40,577	G>A	0.31	0.98	0.96	1.00	1.0E-01	*VEGFA*	intronic		9 tissues		FXR
rs17322780	10	6042472	42,500	40,571	A>G	0.09	0.97	0.94	1.01	1.0E-01	*IL2RA, IL15RA*			PANC, BLD	OVRY	KAP1
rs13029532	2	191875901	42,459	40,532	A>C	0.08	0.97	0.94	1.01	1.0E-01	*GLS, STAT1, STAT4, LOC100420571*	intronic	BLD, GI, HRT	17 tissues	BLD,MUS	CDP,HNF1,Hmbox1
rs4845617	1	154377898	42,197	40,292	G>A	0.40	0.98	0.96	1.00	1.0E-01	*IL6R, MRPS33P1, RPSAP17, PSMD8P1*	5'-UTR	23 tissues	4 tissues	51 tissues	5 altered motifs
rs6972136	7	18959381	42,473	40,537	A>G	0.30	1.02	1.00	1.04	1.0E-01	*HDAC9, NPM1P13*	intronic				GATA,Irf
rs1022034	12	88918743	42,505	40,572	C>A	0.19	0.98	0.95	1.00	1.0E-01	*KITLG*	intronic				6 altered motifs
rs10889675	1	67722216	42,509	40,575	C>A	0.12	1.03	0.99	1.06	1.1E-01	*IL23R, LOC100130497*	intronic			ESDR,SKIN,VAS	8 altered motifs
rs7898890	10	6129874	42,469	40,539	A>T	0.35	1.02	1.00	1.04	1.1E-01	*IL2RA, RBM17, RPL32P23*			BLD	BLD,BLD	12 altered motifs
rs11816044	10	6074082	42,438	40,485	G>A	0.32	0.98	0.96	1.00	1.1E-01	*IL2RA, RPL32P23*	intronic				Hdx,IRC900814
rs13233322	7	18915701	42,508	40,576	C>A	0.37	1.02	1.00	1.04	1.1E-01	*HDAC9*	intronic				7 altered motifs
rs28535133	14	105242826	42,487	40,542	C>A	0.16	1.02	1.00	1.05	1.1E-01	*AKT1, SIVA1, ADSSL1, LINC00638, ZBTB42*	intronic		5 tissues	MUS,BLD	
rs72783726	10	6605559	42,509	40,577	G>A	0.09	0.97	0.94	1.01	1.1E-01	*PRKCQ, PRKCQ-AS1*	intronic	ESDR, BLD, MUS	11 tissues	ESC,LNG,BLD	CEBPB,Nanog,TCF4
rs2276109	11	102745791	42,507	40,573	A>G	0.14	0.98	0.95	1.01	1.1E-01	*MMP3, MMP12, WTAPP1, LOC100288111*			5 tissues	5 tissues	19 altered motifs
rs6060446	20	30231637	42,503	40,575	A>G	0.20	0.98	0.96	1.00	1.1E-01	*BCL2L1, ID1, COX4I2, MIR3193*	intronic			LNG	BRCA1
rs2188321	7	18669314	42,483	40,565	G>C	0.40	0.98	0.96	1.00	1.1E-01	*HDAC9*	intronic				Brachyury,Zfp187
rs10795763	10	6096199	42,461	40,537	A>C	0.41	0.98	0.96	1.00	1.1E-01	*IL2RA, RBM17, RPL32P23*	intronic			11 tissues	Zfp410
rs9610	11	117872086	42,493	40,561	G>A	0.43	1.02	1.00	1.04	1.1E-01	*IL10RA, TMPRSS4-AS1*	3'-UTR	BLD, BRN	6 tissues	BLD,BLD	CEBPB,Ik-2
rs1295687	5	131994462	42,506	40,577	C>G	0.06	0.97	0.93	1.01	1.1E-01	*IL4, IL13, RAD50, KIF3A*	intronic	LIV	4 tissues		
rs1805110	1	92327045	42,499	40,573	G>A	0.08	1.03	0.99	1.07	1.1E-01	*TGFBR3*	missense		BLD		Osr
rs6977642	7	18999729	42,497	40,566	A>G	0.34	0.98	0.96	1.00	1.1E-01	*HDAC9, NPM1P13*	intronic				Irf,Sox
rs10777129	12	88961713	42,509	40,576	G>A	0.08	0.97	0.93	1.01	1.1E-01	*KITLG*	intronic				Pou1f1
rs6793085	3	3142423	42,505	40,572	G>A	0.47	1.02	1.00	1.04	1.1E-01	*IL5RA, TRNT1, CRBN, CNTN4*	intronic		BLD		4 altered motifs
rs7917726	10	6096600	42,497	40,562	A>G	0.41	0.98	0.96	1.00	1.1E-01	*IL2RA, RBM17, RPL32P23*	intronic	LIV	8 tissues	4 tissues	Pax-8
rs12498079	3	30710765	42,401	40,466	T>A	0.13	1.02	0.99	1.05	1.1E-01	*TGFBR2*	intronic		ESC, BLD		6 altered motifs
rs16906063	8	79659801	42,499	40,562	A>G	0.14	0.98	0.95	1.01	1.1E-01	*IL7, ZC2HC1A, PRKRIRP7*	intronic				5 altered motifs
rs6503695	17	40499533	42,506	40,574	A>G	0.34	1.02	1.00	1.04	1.1E-01	*STAT3, STAT5A*	intronic		18 tissues	16 tissues	
rs7716064	5	35878249	42,507	40,577	A>G	0.32	0.98	0.96	1.00	1.1E-01	*IL7R, CAPSL*			BLD, THYM, CRVX		4 altered motifs
rs629080	11	69424606	42,508	40,576	T>A	0.07	1.03	0.99	1.08	1.1E-01	*CCND1, LOC100996515*			BLD		STAT
rs476762	11	102710707	42,507	40,575	T>A	0.14	0.98	0.95	1.01	1.1E-01	*MMP1, MMP3, MMP12, CSNK1A1P2, WTAPP1, LOC100288111*	intronic		GI		GR
rs2863212	1	67685116	42,505	40,575	A>G	0.12	1.03	0.99	1.06	1.1E-01	*IL23R*	intronic				6 altered motifs

SNP	Chr	Position[a]	N Cases	N Controls	Allele	MAF	OR[b]	95%CI		*p* -value	Gene annotation	dbSNP functional annotation	Promoter histone marks	Enhancer histone marks	DNAse	Motifs changed
rs6718902	2	191838204	41,356	39,290	G>A	0.25	1.02	1.00	1.04	1.1E-01	*GLS, STAT1, LOC100420571*	intronic		8 tissues		Nkx2,RXRA
rs10819641	9	101936231	42,510	40,576	G>A	0.06	1.03	0.99	1.08	1.1E-01	*TGFBR1, SEC61B, ALG2*				14 tissues	
rs7904213	10	6148897	42,483	40,524	G>A	0.37	0.98	0.96	1.00	1.1E-01	*IL2RA, PFKFB3, RBM17, RPL32P23, MIR3155A, MIR3155B*	intronic				5 altered motifs
rs10512263	9	101886071	42,505	40,573	A>G	0.07	0.97	0.93	1.01	1.1E-01	*TGFBR1*	intronic			BLD	Ik-1,STAT
rs2025345	10	6067688	42,502	40,568	A>G	0.37	0.98	0.96	1.00	1.1E-01	*IL2RA, IL15RA, RPL32P23*	intronic		ESDR, PANC		
rs2583759	8	79644964	40,622	38,527	A>G	0.19	1.02	1.00	1.05	1.1E-01	*IL7, ZC2HC1A, PRKRIRP7*					
rs334349	9	101914387	41,764	39,855	G>A	0.25	1.02	1.00	1.04	1.1E-01	*TGFBR1*	3'-UTR		LNG, BLD, THYM	IPSC,KID	Myb,Nanog
rs7788972	7	19034280	42,299	40,338	A>T	0.21	1.02	1.00	1.04	1.1E-01	*HDAC9, NPM1P13*	intronic		ESDR, STRM, SKIN		Pou1f1
rs6060652	20	30266845	42,502	40,565	A>G	0.30	0.98	0.96	1.00	1.1E-01	*BCL2L1, COX4I2*	intronic		24 tissues	10 tissues	
rs2297136	9	5467955	42,448	40,537	G>A	0.50	0.98	0.96	1.00	1.1E-01	*CD274, PLGRKT, PDCD1LG2, LOC100419687*	3'-UTR		BRST	SKIN	EWSR1-FLI1,Ets
rs1000788	12	88946777	42,436	40,448	A>G	0.20	0.98	0.96	1.00	1.1E-01	*KITLG*	intronic			21 tissues	HNF4,Hoxa13,RREB-1
rs2796817	1	218551008	42,504	40,574	A>C	0.14	1.02	0.99	1.05	1.1E-01	*TGFB2, RRP15, RPS26P17, LOC728463*	intronic	7 tissues	14 tissues	13 tissues	ATF3,CTCF,SRF
rs801540	7	18672384	42,499	40,570	A>C	0.41	0.98	0.96	1.00	1.1E-01	*HDAC9*	intronic				Arid5a
rs2298432	22	22123189	42,510	40,577	C>A	0.38	1.02	1.00	1.04	1.1E-01	*MAPK1, YPEL1*	intronic		BLD, THYM	SKIN	AP-1,EWSR1-FLI1,RFX5
rs1132975	14	105222037	42,496	40,569	G>A	0.33	1.02	1.00	1.04	1.1E-01	*AKT1, SIVA1, INF2, ADSSL1, ZBTB42, LOC100996409*	synonymous		11 tissues	THYM,PANC,GI	RXRA
rs662123	11	69423473	42,486	40,543	A>G	0.07	1.03	0.99	1.07	1.2E-01	*CCND1, LOC100996515*				BRN	6 altered motifs
rs3892225	1	218553297	42,507	40,576	A>G	0.19	0.98	0.95	1.01	1.2E-01	*TGFB2, RRP15, RPS26P17, LOC728463*	intronic	LNG	12 tissues		5 altered motifs
rs11259425	10	6577673	42,507	40,575	G>C	0.11	0.97	0.94	1.01	1.2E-01	*PRKCQ, PRKCQ-AS1*	intronic		BLD, SPLN	IPSC	TCF12
rs1178119	7	18753320	42,496	40,563	A>G	0.21	1.02	1.00	1.04	1.2E-01	*HDAC9*	intronic				Pou2f2,Pou3f3,RP58
rs7100767	10	6138153	42,504	40,573	T>A	0.38	0.98	0.96	1.00	1.2E-01	*IL2RA, PFKFB3, RBM17, RPL32P23*	intronic	STRM, BRN	16 tissues		7 altered motifs
rs2407206	12	88943323	42,507	40,574	A>G	0.20	0.98	0.96	1.01	1.2E-01	*KITLG*	intronic		5 tissues		Pou5f1,Sox
rs16902386	8	128757780	42,495	40,559	A>G	0.24	0.98	0.96	1.00	1.2E-01	*MYC*			6 tissues	LNG,CRVX,BRST	
rs10509561	10	90751912	42,502	40,568	T>A	0.37	0.98	0.96	1.00	1.2E-01	*ACTA2, FAS, FAS-AS1*	intronic			17 tissues	CHOP::CEBPalpha,Cphx,Hdx
rs16906164	8	79753179	42,488	40,556	G>C	0.17	0.98	0.95	1.01	1.2E-01	*IL7*		BLD	BLD, STRM, PANC	BLD,BRST	FAC1,Foxp1,Pou1f1
rs12358961	10	6066195	42,506	40,567	A>T	0.37	0.98	0.96	1.00	1.2E-01	*IL2RA, IL15RA, RPL32P23*	intronic		4 tissues	SKIN	GR
rs7945189	11	102660564	42,509	40,577	G>A	0.10	0.97	0.94	1.01	1.2E-01	*MMP1, MMP3, MMP10, CSNK1A1P2, WTAPP1, LOC100421658*	intronic			SKIN	14 altered motifs
rs2285430	7	18674040	42,501	40,570	C>A	0.44	0.98	0.96	1.00	1.2E-01	*HDAC9*	intronic				En-1,GR
rs12029576	1	218559475	42,459	40,547	A>C	0.31	0.98	0.96	1.00	1.2E-01	*TGFB2, RRP15, RPS26P17, LOC728463*	intronic		BRN, LNG, MUS		CIZ,GR,Homez
rs801763	7	18601365	42,495	40,562	G>A	0.14	0.98	0.95	1.01	1.2E-01	*HDAC9, LOC100419901*	intronic		SKIN, HRT	SKIN	HNF4
rs3773640	3	30709511	42,505	40,574	A>T	0.24	1.02	1.00	1.04	1.2E-01	*TGFBR2*	intronic	BLD	15 tissues	ESDR	9 altered motifs
rs2204113	3	150320322	42,476	40,538	G>A	0.47	1.02	1.00	1.04	1.2E-01	*SELT, EIF2A, LOC677762*		20 tissues	7 tissues	SKIN,HRT	TCF12
rs10858758	12	88944518	42,506	40,572	A>G	0.20	0.98	0.96	1.01	1.2E-01	*KITLG*	intronic				
rs4812997	20	44741498	42,499	40,572	A>G	0.30	1.02	1.00	1.04	1.2E-01	*CD40, NCOA5, RPL13P2*			ESDR, BRST, BLD	ESDR	GATA,SRF
rs7530511	1	67685387	42,510	40,577	G>A	0.13	1.02	0.99	1.05	1.2E-01	*IL23R*	missense				4 altered motifs
rs2498802	14	105234442	42,479	40,557	C>G	0.34	1.02	1.00	1.04	1.2E-01	*AKT1, SIVA1, INF2, ADSSL1, ZBTB42, LOC100996409*		SKIN	8 tissues	BLD,SKIN,MUS	5 altered motifs
rs4956410	4	142687795	42,388	40,448	G>A	0.08	0.97	0.94	1.01	1.2E-01	*IL15*					Ik-1
rs566125	11	102710471	42,441	40,504	G>A	0.14	0.98	0.95	1.01	1.2E-01	*MMP1, MMP3, MMP12, CSNK1A1P2, WTAPP1, LOC100288111*	intronic		GI		5 altered motifs
rs10887876	10	90745130	42,507	40,576	G>A	0.11	1.03	0.99	1.06	1.2E-01	*ACTA2, FAS, ACTA2-AS1, FAS-AS1*	intronic				
rs13436926	5	35825173	42,498	40,568	G>A	0.40	1.02	1.00	1.04	1.2E-01	*IL7R, SPEF2*		BLD	BLD, THYM		
rs7279064	21	34614255	42,502	40,575	A>C	0.32	1.02	1.00	1.04	1.2E-01	*IFNAR2, IL10RB, IL10RB-AS1*					
rs6573911	14	25100933	42,503	40,564	A>G	0.23	0.98	0.96	1.01	1.2E-01	*GZMH, GZMB*	intronic	BLD	BLD, GI, PANC	BLD	5 altered motifs
rs340808	3	3113831	42,482	40,555	C>G	0.49	0.98	0.96	1.00	1.2E-01	*IL5RA, CNTN4*	intronic				LRH1,NRSF,PU.1
rs17506603	8	79750813	42,508	40,573	A>G	0.14	0.98	0.95	1.01	1.2E-01	*IL7*					
rs6968777	7	19011349	42,506	40,576	A>G	0.40	1.02	1.00	1.04	1.2E-01	*HDAC9, NPM1P13*	intronic			MUS	Mef2,Sox
rs41430444	2	191878487	42,503	40,574	A>G	0.10	0.97	0.94	1.01	1.2E-01	*GLS, STAT1, STAT4, LOC100420571*	intronic	24 tissues		29 tissues	5 altered motifs
rs8192917	14	25102160	42,505	40,571	A>G	0.23	0.98	0.96	1.01	1.3E-01	*GZMH, GZMB*	missense	BLD	GI, MUS, THYM	THYM	
rs2450254	11	69449784	42,494	40,569	A>T	0.40	0.98	0.96	1.00	1.3E-01	*CCND1, ORAOV1, LOC100996515*			8 tissues	LIV	4 altered motifs
rs3213427	12	6928747	42,465	40,560	A>G	0.44	0.98	0.96	1.00	1.3E-01	*CD4, GNB3, LAG3, PTMS, TPI1, USP5, LEPREL2, GPR162, CDCA3*	3'-UTR	LIV	11 tissues	ESDR,SKIN	ERalpha-a,Esr2
rs10796145	10	6474027	42,475	40,555	A>G	0.29	0.98	0.96	1.00	1.3E-01	*PRKCQ*	intronic			4 tissues	Ets
rs12924697	16	85894429	42,498	40,569	A>G	0.19	1.02	0.99	1.05	1.3E-01	*IRF8, RPL10AP12, LOC100422319*			9 tissues		AP-1,GR
rs4474514	12	88953959	42,495	40,565	A>G	0.20	0.98	0.96	1.01	1.3E-01	*KITLG*	intronic		6 tissues	5 tissues	6 altered motifs
rs34353319	10	6029986	42,497	40,571	A>T	0.25	0.98	0.96	1.01	1.3E-01	*IL2RA, IL15RA*		BRN, BLD	7 tissues	12 tissues	Hoxb13,SIX5,TATA
rs1295683	5	131998876	42,432	40,468	G>A	0.11	0.98	0.95	1.01	1.3E-01	*IL4, IL13, RAD50, KIF3A*		BLD, LIV	13 tissues	4 tissues	Ascl2,Rad21
rs6060627	20	30262159	42,505	40,576	G>A	0.30	0.98	0.96	1.00	1.3E-01	*BCL2L1, COX4I2*	intronic	BLD	15 tissues	11 tissues	ZBRK1
rs235218	1	12264871	42,195	40,074	C>A	0.09	0.97	0.94	1.01	1.3E-01	*TNFRSF1B, VPS13D, LOC390998, MIR4632*	intronic		7 tissues		FAC1,Pax-4,Zfp105
rs10919202	1	169613794	42,503	40,569	A>G	0.16	0.98	0.95	1.01	1.3E-01	*SELL, SELP*					PPAR

SNP	Chr	Position[a]	N Cases	N Controls	Allele	MAF	OR[b]	95%CI		*p* -value	Gene annotation	dbSNP functional annotation	Promoter histone marks	Enhancer histone marks	DNAse	Motifs changed
rs3810389	19	40257116	42,502	40,576	A>G	0.14	1.02	0.99	1.05	1.3E-01	*CLC, LEUTX*			ESC, IPSC, BLD	BLD	
rs2029356	1	92209311	42,510	40,574	G>A	0.26	1.02	0.99	1.04	1.3E-01	*TGFBR3*	intronic		11 tissues		BCL
rs4714699	6	43802563	42,464	40,546	A>G	0.38	0.98	0.96	1.00	1.3E-01	*VEGFA*			7 tissues	ESDR	10 altered motifs
rs3782179	12	88953326	42,507	40,571	A>G	0.20	0.98	0.96	1.01	1.3E-01	*KITLG*	intronic		10 tissues	GI,LNG	
rs11465791	1	67649496	42,506	40,559	G>A	0.06	1.03	0.99	1.08	1.3E-01	*IL23R*	intronic				4 altered motifs
rs6518660	22	17575800	41,793	39,604	A>G	0.16	0.98	0.95	1.01	1.3E-01	*IL17RA, CECR6, CECR5, CECR7, RPL31P62, LOC100996342*	intronic		BLD		Myb,STAT
rs4645956	8	128750212	42,283	40,302	G>A	0.10	0.97	0.94	1.01	1.3E-01	*MYC*	intronic	24 tissues		51 tissues	5 altered motifs
rs10873219	14	25101706	42,508	40,576	C>A	0.20	0.98	0.96	1.01	1.3E-01	*GZMH, GZMB*	intronic			BLD,GI,THYM	VDR
rs944714	10	6475157	42,492	40,562	G>A	0.29	0.98	0.96	1.00	1.3E-01	*PRKCQ*	intronic				
rs2799523	1	92316682	42,509	40,575	G>A	0.08	1.03	0.99	1.07	1.3E-01	*TGFBR3*	intronic	15 tissues	9 tissues	5 tissues	4 altered motifs
rs1608555	7	22807473	40,655	38,522	G>A	0.36	0.98	0.96	1.00	1.3E-01	*IL6, TOMM7, RPS26P32, LOC541472*					YY1
rs12610904	19	40238776	42,507	40,577	G>A	0.14	1.02	0.99	1.05	1.3E-01	*CLC, LGALS14, LEUTX*					4 altered motifs
rs3782180	12	88953399	42,510	40,577	A>C	0.20	0.98	0.96	1.01	1.3E-01	*KITLG*	intronic		10 tissues	SKIN	21 altered motifs
rs710046	14	62119993	42,507	40,572	G>A	0.16	0.98	0.95	1.01	1.3E-01	*HIF1A, FLJ22447*	intronic	STRM	14 tissues	ESDR,VAS	4 altered motifs
rs3024630	16	27366126	42,501	40,577	A>G	0.09	0.97	0.94	1.01	1.3E-01	*IL4R, IL21R*	intronic			OVRY	
rs17349832	7	18864637	42,485	40,545	G>A	0.21	0.98	0.96	1.01	1.3E-01	*HDAC9*	intronic				
rs75745972	1	11254013	42,445	40,522	A>C	0.22	1.02	0.99	1.04	1.3E-01	*MTOR, ANGPTL7, RPL39P6, MTOR-AS1*					
rs12535092	7	19013672	42,504	40,576	G>A	0.21	1.02	0.99	1.04	1.3E-01	*HDAC9, NPM1P13*	intronic		4 tissues		Zfp410
rs10519614	4	142666982	42,509	40,575	C>A	0.09	0.97	0.94	1.01	1.3E-01	*IL15*					7 altered motifs
rs6660484	1	92172608	42,506	40,574	A>G	0.42	0.98	0.96	1.00	1.3E-01	*TGFBR3*	intronic		5 tissues		HMG-IY,Hoxa9,Sox
rs17804441	9	5457733	42,505	40,572	A>G	0.23	1.02	0.99	1.04	1.3E-01	*CD274, PLGRKT, LOC100419687*	intronic				Lmo2-complex,SZF1-1
rs3907470	12	88958350	42,497	40,570	A>G	0.20	0.98	0.96	1.01	1.3E-01	*KITLG*	intronic		10 tissues		
rs284191	1	92236048	42,375	40,404	A>G	0.38	1.02	1.00	1.04	1.3E-01	*TGFBR3*	intronic		7 tissues		4 altered motifs
rs17435702	7	19023864	42,494	40,566	G>A	0.39	1.02	1.00	1.04	1.3E-01	*HDAC9, NPM1P13*	intronic				Mef2,PLZF
rs2508450	11	117863829	42,501	40,573	G>A	0.44	0.98	0.97	1.00	1.3E-01	*IL10RA, TMPRSS4-AS1*	intronic		BLD, GI, PLCNT	GI,BLD	PPAR
rs1178099	7	18735247	42,505	40,576	A>G	0.32	1.02	1.00	1.04	1.3E-01	*HDAC9*	intronic			ADRL	ERalpha-a
rs11104952	12	88956400	42,507	40,575	C>A	0.20	0.98	0.96	1.01	1.3E-01	*KITLG*	intronic		8 tissues		AP-3,Sox
rs3814989	7	18634822	41,820	39,625	A>G	0.25	0.98	0.96	1.01	1.3E-01	*HDAC9, LOC100419901*	intronic				7 altered motifs
rs4655693	1	67692286	42,453	40,538	T>A	0.13	1.02	0.99	1.05	1.3E-01	*IL23R*	intronic		4 tissues	BLD	Hoxa13
rs2273844	14	25103414	42,505	40,569	G>A	0.23	0.98	0.96	1.01	1.3E-01	*GZMH, GZMB*	5'-UTR	BLD	BLD, THYM	7 tissues	AP-1,EWSR1-FLI1
rs231777	2	204733588	42,505	40,571	G>A	0.15	1.02	0.99	1.05	1.3E-01	*CTLA4*	intronic	BLD, GI, THYM	BLD		STAT
rs2634030	1	92317891	42,508	40,572	A>G	0.08	1.03	0.99	1.07	1.3E-01	*TGFBR3*	intronic	LNG	13 tissues	LNG	CEBPG,Hsf,STAT
rs10235067	7	18601905	42,362	40,418	A>G	0.26	0.98	0.96	1.01	1.3E-01	*HDAC9, LOC100419901*	intronic				5 altered motifs
rs10486316	7	18935459	42,495	40,562	A>G	0.24	1.02	0.99	1.04	1.3E-01	*HDAC9*	intronic			9 tissues	STAT
rs1914408	2	191839976	42,507	40,574	G>A	0.23	1.02	0.99	1.04	1.3E-01	*GLS, STAT1, LOC100420571*	intronic		7 tissues	HRT	Evi-1,HES1
rs8192921	14	25099908	42,508	40,576	T>A	0.24	0.98	0.96	1.01	1.3E-01	*GZMH, GZMB*			9 tissues	BLD,LNG	NF-kappaB
rs12874529	13	28583166	42,496	40,560	A>G	0.25	0.98	0.96	1.01	1.3E-01	*CDX2, FLT3, PRHOXNB*	intronic				
rs2286213	7	18914251	42,506	40,570	G>C	0.36	1.02	1.00	1.04	1.3E-01	*HDAC9*	intronic		FAT, SKIN		
rs1984547	17	25963317	42,177	40,142	A>G	0.24	1.02	0.99	1.04	1.3E-01	*LGALS9, KSR1, NOS2P1, ITM2BP1, LOC100420408*	intronic	BLD	BLD, GI		SIX5,Znf143,p300
rs16966365	17	25953203	42,495	40,573	G>A	0.22	1.02	0.99	1.04	1.3E-01	*LGALS9, KSR1, NOS2P1, ITM2BP1, LOC100420408*					
rs1342586	1	218597859	42,506	40,570	G>A	0.22	0.98	0.96	1.01	1.3E-01	*TGFB2*	intronic		7 tissues		
rs4794975	17	25970847	42,505	40,572	G>A	0.24	1.02	0.99	1.04	1.3E-01	*LGALS9, KSR1, NOS2P1, ITM2BP1, LOC100420408*	intronic		FAT, GI, MUS	LNG	
rs2799524	1	92317312	42,508	40,573	G>A	0.08	1.03	0.99	1.07	1.3E-01	*TGFBR3*	intronic	8 tissues	14 tissues	5 tissues	7 altered motifs
rs10482796	1	218605635	42,497	40,569	A>G	0.21	0.98	0.96	1.01	1.3E-01	*TGFB2*	intronic				6 altered motifs
rs4655530	1	67703731	42,493	40,565	A>G	0.13	1.02	0.99	1.05	1.3E-01	*IL23R, LOC100130497*	intronic				AP-1,Hoxc10,p300
rs12222869	11	69427448	42,307	40,209	A>C	0.30	1.02	0.99	1.04	1.3E-01	*CCND1, LOC100996515*					7 altered motifs
rs2046971	12	88959510	42,509	40,576	G>C	0.20	0.98	0.96	1.01	1.4E-01	*KITLG*	intronic		4 tissues		4 altered motifs
rs10125854	9	5464065	42,506	40,574	A>G	0.07	0.97	0.93	1.01	1.4E-01	*CD274, PLGRKT, PDCD1LG2, LOC100419687*	intronic				Evi-1,NF-AT1
rs10515921	2	102981018	42,509	40,574	A>C	0.13	1.02	0.99	1.05	1.4E-01	*IL18R1, IL1RL1, LOC100422339*	intronic				Eomes
rs710036	14	62132296	42,506	40,574	A>G	0.15	0.98	0.95	1.01	1.4E-01	*HIF1A, FLJ22447*			BLD		AP-3
rs17150238	7	18604337	42,050	40,027	A>G	0.24	0.98	0.96	1.01	1.4E-01	*HDAC9, LOC100419901*	intronic				Foxj2,Pou2f2,TEF
rs11594656	10	6122009	42,495	40,573	A>T	0.24	0.98	0.96	1.01	1.4E-01	*IL2RA, RBM17, RPL32P23*			BLD	IPSC	Cphx,KAP1
rs2076846	10	6063253	42,481	40,564	A>G	0.35	1.02	1.00	1.04	1.4E-01	*IL2RA, IL15RA*	intronic		BLD	BLD	
rs6977304	7	18999534	42,496	40,565	A>G	0.34	0.98	0.96	1.01	1.4E-01	*HDAC9, NPM1P13*	intronic		ESDR		CEBPB,GR,p300
rs12118310	1	92178787	42,493	40,562	A>C	0.09	0.97	0.94	1.01	1.4E-01	*TGFBR3*	intronic	8 tissues	15 tissues	16 tissues	5 altered motifs
rs883873	1	92380302	42,509	40,577	A>G	0.08	1.03	0.99	1.07	1.4E-01	*BRDT, TGFBR3*			PLCNT, CRVX	CRVX	12 altered motifs
rs7788833	7	19034191	42,269	40,291	A>G	0.21	1.02	0.99	1.04	1.4E-01	*HDAC9, NPM1P13*	intronic		4 tissues		6 altered motifs
rs17131515	1	92116019	42,509	40,574	C>A	0.08	1.03	0.99	1.07	1.4E-01	*TGFBR3, HSP90B3P, RPL39P13*		FAT	14 tissues	SKIN	Nr2f2

SNP	Chr	Position[a]	N Cases	N Controls	Allele	MAF	OR[b]	95%CI		*p* -value	Gene annotation	dbSNP functional annotation	Promoter histone marks	Enhancer histone marks	DNAse	Motifs changed
rs7808313	7	18864040	42,502	40,572	A>C	0.09	1.03	0.99	1.06	1.4E-01	*HDAC9*	intronic				7 altered motifs
rs3679	19	7978430	42,470	40,547	G>A	0.35	0.98	0.96	1.01	1.4E-01	*ELAVL1, MAP2K7, SNAPC2, TIMM44, LRRC8E, FLJ22184, EVI5L, LOC388499, CTXN1, TGFBR3L*	3'-UTR		ESDR, SKIN, BLD	MUS,LIV	5 altered motifs
rs1472899	12	88951485	42,331	40,370	A>G	0.20	0.98	0.96	1.01	1.4E-01	*KITLG*	intronic		ESDR, BRN, SKIN		
rs2294884	6	32367259	42,502	40,574	A>C	0.18	1.02	0.99	1.05	1.4E-01	*HLA-DRA, C6orf10, BTNL2, HCG23*	intronic				PLZF
rs11804284	1	67699254	42,509	40,577	G>A	0.13	1.02	0.99	1.05	1.4E-01	*IL23R, LOC100130497*	intronic				PLZF
rs6973029	7	18908850	42,507	40,575	G>A	0.35	1.02	0.99	1.04	1.4E-01	*HDAC9*	intronic				
rs2239695	11	118213632	42,482	40,566	G>A	0.15	1.02	0.99	1.05	1.4E-01	*CD3D, CD3E, CD3G, UBE4A, LOC100131626*		BLD, GI, THYM	ESC, IPSC, LIV	5 tissues	FAC1,RREB-1
rs11879130	19	40246387	42,509	40,577	G>A	0.14	1.02	0.99	1.05	1.4E-01	*CLC, LGALS14, LEUTX*			ADRL	LNG	
rs11466515	3	30714586	42,486	40,557	A>C	0.31	1.02	0.99	1.04	1.4E-01	*TGFBR2*	intronic		12 tissues	MUS,MUS,BRN	NRSF,Sin3Ak-20
rs8143115	22	22256773	42,508	40,575	G>C	0.14	1.02	0.99	1.05	1.4E-01	*MAPK1, PPM1F, LOC100286925*			PLCNT, BLD, LIV	4 tissues	5 altered motifs
rs12569077	1	92110475	42,500	40,571	A>G	0.14	0.98	0.95	1.01	1.4E-01	*TGFBR3, HSP90B3P, RPL39P13*			7 tissues		Mef2,PLZF,RXRA
rs2802211	1	11132217	42,507	40,574	A>G	0.26	1.02	0.99	1.04	1.4E-01	*MTOR, EXOSC10, SRM, MASP2, TARDBP*					
rs4656711	1	169723575	42,504	40,571	G>A	0.21	0.98	0.96	1.01	1.4E-01	*SELE, SELL, C1orf112, METTL18*					Nanog,Pou2f2
rs3181259	11	118214067	42,507	40,577	G>A	0.15	1.02	0.99	1.05	1.4E-01	*CD3D, CD3E, CD3G, UBE4A, LOC100131626*		BLD, THYM	BLD	7 tissues	NRSF
rs3819001	1	1138913	42,505	40,574	A>G	0.06	0.97	0.93	1.01	1.4E-01	*TNFRSF4, TNFRSF18, SDF4, B3GALT6, TTLL10, FAM132A, MIR200A, MIR200B, MIR429, TTLL10-AS1*	3'-UTR	BRST	6 tissues	BLD	8 altered motifs
rs2799086	1	218556202	42,508	40,574	G>A	0.14	0.98	0.95	1.01	1.4E-01	*TGFB2, RRP15, RPS26P17, LOC728463*	intronic		5 tissues		4 altered motifs
rs9267873	6	32199352	42,490	40,558	A>G	0.42	1.02	0.99	1.04	1.4E-01	*AGER, NOTCH4, PBX2, GPSM3*					
rs11080242	17	25964669	42,332	40,414	C>G	0.23	1.02	0.99	1.04	1.4E-01	*LGALS9, KSR1, NOS2P1, ITM2BP1, LOC100420408*	intronic		ESDR, GI		BDP1,LUN-1
rs66516223	11	69431628	42,510	40,576	G>A	0.07	1.03	0.99	1.07	1.4E-01	*CCND1, ORAOV1, LOC100996515*			PLCNT, LIV	PLCNT	SP1
rs917913	12	9859636	42,161	40,238	G>A	0.47	0.99	0.97	1.01	1.4E-01	*CD69, NPM1P7, CLEC2D, CLECL1, LOC374443*			BRST, BLD, THYM		4 altered motifs
rs6686126	1	92310874	42,509	40,576	G>A	0.07	1.03	0.99	1.07	1.4E-01	*TGFBR3*	intronic	LNG, LIV	19 tissues	28 tissues	5 altered motifs
rs12257092	10	90782827	42,506	40,577	A>T	0.30	0.98	0.96	1.01	1.4E-01	*ACTA2, FAS, FAS-AS1, MIR4679-1, MIR4679-2*			SKIN, BRST	4 tissues	HNF4,Hand1,NF-kappaB
rs6869411	5	158781604	42,509	40,576	A>G	0.37	0.98	0.96	1.01	1.4E-01	*IL12B, LOC285626, RNU4ATAC2P*	intronic				9 altered motifs
rs1409282	6	43796734	42,506	40,577	G>A	0.31	1.02	0.99	1.04	1.4E-01	*VEGFA*			GI, BLD	4 tissues	4 altered motifs
rs631968	10	6475296	42,503	40,569	A>G	0.29	0.98	0.96	1.01	1.4E-01	*PRKCQ*	intronic				4 altered motifs
rs7236029	18	60057061	42,509	40,577	A>G	0.07	0.97	0.93	1.01	1.4E-01	*TNFRSF11A, RPL17P44*			5 tissues	5 tissues	6 altered motifs
rs801513	7	18646433	42,106	40,077	G>A	0.12	0.98	0.95	1.01	1.4E-01	*HDAC9, LOC100419901*	intronic		IPSC		Hoxd10,Pou5f1
rs8095109	18	60057136	42,508	40,572	A>G	0.30	0.98	0.96	1.01	1.4E-01	*TNFRSF11A, RPL17P44*			5 tissues	8 tissues	GATA
rs1492351	12	88944009	42,485	40,540	G>A	0.20	0.98	0.96	1.01	1.4E-01	*KITLG*	intronic		10 tissues	4 tissues	7 altered motifs
rs9661103	1	92335906	42,509	40,571	G>A	0.32	0.98	0.96	1.01	1.4E-01	*TGFBR3*	intronic		7 tissues		5 altered motifs
rs17069898	18	60029281	42,505	40,576	A>G	0.37	1.02	0.99	1.04	1.4E-01	*TNFRSF11A*	intronic				Msx-1,RREB-1
rs6461389	7	18907030	42,490	40,565	G>A	0.36	1.02	0.99	1.04	1.5E-01	*HDAC9*	intronic		6 tissues	SKIN	CEBPB,Sp100
rs7545838	1	92342885	42,174	40,163	A>C	0.35	0.98	0.96	1.01	1.5E-01	*TGFBR3*	intronic		10 tissues	SKIN	7 altered motifs
rs1126639	14	25101548	42,377	40,452	G>A	0.23	0.98	0.96	1.01	1.5E-01	*GZMH, GZMB*	synonymous	BLD	BLD, GI, THYM		5 altered motifs
rs494379	11	102669210	42,377	40,410	A>G	0.21	0.98	0.96	1.01	1.5E-01	*MMP1, MMP3, MMP10, CSNK1A1P2, WTAPP1, LOC100421658*	intronic	FAT	10 tissues	6 tissues	AIRE,BATF
rs7089508	10	6578039	42,507	40,572	A>G	0.49	1.01	0.99	1.04	1.5E-01	*PRKCQ, PRKCQ-AS1*	intronic		BLD, THYM, SPLN	BLD	4 altered motifs
rs4090392	1	173147149	42,464	40,460	T>A	0.18	1.02	0.99	1.05	1.5E-01	*TNFSF4, GOT2P2*					8 altered motifs
rs3006475	1	153344636	42,507	40,576	A>C	0.11	0.98	0.95	1.01	1.5E-01	*S100A8, S100A9, S100A12, PGLYRP4, S100A7A, LOC645900*		BLD	BLD, GI		
rs646423	11	69426204	42,496	40,570	G>A	0.21	1.02	0.99	1.04	1.5E-01	*CCND1, LOC100996515*			LIV, BONE		4 altered motifs
rs3761549		49117345	42,506	40,577	G>A	0.13	1.02	0.99	1.05	1.5E-01	*CACNA1F, CCDC22, FOXP3, PPP1R3F, GAGE10, HSPB1P2*	intronic	BLD	THYM	THYM	GR
rs11692784	2	54188394	42,503	40,577	A>T	0.06	1.03	0.99	1.08	1.5E-01	*PSME4*	intronic		11 tissues		9 altered motifs
rs653241	6	86116532	42,466	40,533	C>A	0.10	0.98	0.94	1.01	1.5E-01	*NT5E, LOC643870, DUTP5*		LIV	15 tissues	4 tissues	4 altered motifs
rs1320565	1	1119858	42,499	40,573	G>A	0.08	0.97	0.94	1.01	1.5E-01	*TNFRSF4, TNFRSF18, SDF4, B3GALT6, LOC254099, TTLL10, MIR200A, MIR200B, MIR429, TTLL10-AS1*	intronic		5 tissues		STAT
rs12450102	17	76248280	42,483	40,565	A>G	0.16	1.02	0.99	1.05	1.5E-01	*BIRC5, AFMID, TMEM235, THA1P, LOC100996291*		4 tissues	9 tissues	9 tissues	CTCF,Rad21
rs2788570	1	11289466	42,486	40,559	G>A	0.22	1.02	0.99	1.04	1.5E-01	*MTOR, ANGPTL7, UBIAD1, RPL39P6, UBE2V2P3*	intronic		BLD	BLD	13 altered motifs
rs2796813	1	218589197	42,498	40,572	A>G	0.43	0.99	0.97	1.01	1.5E-01	*TGFB2*	intronic		MUS, LNG	BLD	
rs1253945	10	44911825	42,506	40,574	A>G	0.20	0.98	0.96	1.01	1.5E-01	*CXCL12, RPL9P21*		FAT, STRM	12 tissues	10 tissues	Nanog,Pou2f2
rs2229546	1	67861520	42,505	40,573	A>C	0.32	1.02	0.99	1.04	1.5E-01	*IL12RB2, SERBP1*	synonymous		SKIN, HRT, MUS		
rs7895774	10	6510534	42,498	40,557	G>A	0.30	1.02	0.99	1.04	1.5E-01	*PRKCQ*	intronic				

SNP	Chr	Position[a]	N Cases	N Controls	Allele	MAF	OR[b]	95%CI		*p* -value	Gene annotation	dbSNP functional annotation	Promoter histone marks	Enhancer histone marks	DNAse	Motifs changed
rs11209072	1	67908644	42,099	40,554	A>G	0.23	1.02	0.99	1.04	1.5E-01	*IL12RB2, SERBP1*			HRT	HRT	5 altered motifs
rs1411263	9	5459103	42,506	40,572	A>G	0.23	1.02	0.99	1.04	1.5E-01	*CD274, PLGRKT, LOC100419687*	intronic	BLD	7 tissues	MUS,BLD	
rs7145164	14	62228299	42,500	40,568	A>G	0.17	0.98	0.95	1.01	1.5E-01	*HIF1A, SNAPC1, HIF1A-AS2*		8 tissues	13 tissues	BLD	
rs2234895	16	27357927	42,509	40,576	G>A	0.09	0.97	0.94	1.01	1.5E-01	*IL4R*	missense	BLD	7 tissues	BLD,BLD,BLD	4 altered motifs
rs709592	17	38175553	42,417	40,526	G>A	0.39	0.99	0.97	1.01	1.5E-01	*CSF3, PSMD3, THRA, MED24, GSDMA, LOC100505620*	3'-UTR	MUS, GI	18 tissues	20 tissues	
rs2181135		135746282	42,509	40,576	G>A	0.08	0.97	0.94	1.01	1.5E-01	*CD40LG, ARHGEF6, LINC00892*		BLD	BLD		AP-1,ELF1,Ik-2
rs75653957	1	11320776	42,375	40,472	A>G	0.27	1.02	0.99	1.04	1.5E-01	*MTOR, UBIAD1, RPL39P6, UBE2V2P3*	intronic		BRST, BLD		7 altered motifs
rs2291627	15	40259848	42,510	40,576	A>C	0.08	1.03	0.99	1.06	1.5E-01	*GPR176, EIF2AK4, H3F3AP1, LOC100505534*	missense		ESC		GATA,Nr2e3
rs801521	7	18703062	42,506	40,575	C>A	0.09	0.97	0.94	1.01	1.5E-01	*HDAC9*	intronic	BLD	4 tissues	7 tissues	4 altered motifs
rs801512	7	18644762	42,509	40,575	G>A	0.11	0.98	0.95	1.01	1.6E-01	*HDAC9, LOC100419901*	intronic				4 altered motifs
rs2016515	7	18874385	42,508	40,575	A>C	0.31	1.02	0.99	1.04	1.6E-01	*HDAC9*	intronic	FAT	5 tissues	SKIN,LNG,MUS	CIZ,SIX5
rs801541	7	18672481	42,486	40,560	T>A	0.42	0.99	0.97	1.01	1.6E-01	*HDAC9*	intronic				HNF4,Nkx6-1
rs1021580	5	54439466	42,504	40,568	A>G	0.17	0.98	0.96	1.01	1.6E-01	*GZMA, CDC20B, GPX8, MIR449A, MIR449B, MIR449C*	missense				GR,ZBTB33
rs1089358	7	18641842	42,144	40,141	C>G	0.12	0.98	0.95	1.01	1.6E-01	*HDAC9, LOC100419901*	intronic				ATF3,BHLHE40,Mef2
rs6074028	20	44754858	42,494	40,567	A>G	0.14	1.02	0.99	1.05	1.6E-01	*CD40, NCOA5, CDH22, RPL13P2*	intronic		GI, BLD, LIV		20 altered motifs
rs5746016	1	12250979	42,497	40,561	G>A	0.06	0.97	0.93	1.01	1.6E-01	*TNFRSF8, TNFRSF1B, VPS13D, LOC390998, MIR4632*	intronic		ESC, BLD, PLCNT		Smad4,p53
rs6951522	7	18867953	42,508	40,575	A>G	0.36	1.02	0.99	1.04	1.6E-01	*HDAC9*	intronic		FAT, MUS	MUS	5 altered motifs
rs72931559	18	59992997	42,442	40,536	G>A	0.24	1.02	0.99	1.04	1.6E-01	*TNFRSF11A, KIAA1468*	intronic	20 tissues	ESDR, LNG, BRN	18 tissues	10 altered motifs
rs1779343	10	44908783	42,506	40,577	G>A	0.20	0.98	0.96	1.01	1.6E-01	*CXCL12, RPL9P21*			ADRL		HNF4,VDR
rs3782181	12	88953561	42,508	40,573	A>C	0.20	0.98	0.96	1.01	1.6E-01	*KITLG*	intronic		10 tissues	7 tissues	AP-4,LBP-1,TCF12
rs6700529	1	92236342	42,487	40,559	C>A	0.10	1.02	0.99	1.06	1.6E-01	*TGFBR3*	intronic		8 tissues	LNG	ZEB1
rs3761548		49118241	42,459	40,550	C>A	0.45	0.99	0.97	1.01	1.6E-01	*CACNA1F, CCDC22, FOXP3, PPP1R3F, GAGE10, HSPB1P2*	intronic		THYM		DEC,Egr-1,Myb
rs12699994	7	18895297	42,396	40,416	G>A	0.36	1.02	0.99	1.04	1.6E-01	*HDAC9*	intronic		SKIN, VAS, BONE		GR,HNF4
rs7528924	1	67689036	42,150	40,202	A>G	0.25	1.02	0.99	1.04	1.6E-01	*IL23R*	intronic				HDAC2
rs11466531	3	30733838	42,509	40,577	G>C	0.05	0.97	0.92	1.01	1.6E-01	*TGFBR2, GADL1*	3'-UTR		14 tissues		PRDM1,ZBTB33
rs741200	12	9870415	42,498	40,571	A>C	0.46	0.99	0.97	1.01	1.6E-01	*CD69, NPM1P7, CLEC2D, CLECL1*	intronic	BLD	BLD	BLD,BLD,BLD	CDP
rs72718557	8	128797655	42,507	40,575	G>C	0.10	0.98	0.94	1.01	1.6E-01	*MYC, MIR1204*			ESC, BLD	BLD	AP-3,LUN-1
rs3773645	3	30712460	42,507	40,575	C>G	0.31	1.02	0.99	1.04	1.6E-01	*TGFBR2*	intronic		4 tissues		
rs650108	11	102708787	42,507	40,576	G>A	0.25	1.02	0.99	1.04	1.6E-01	*MMP1, MMP3, MMP12, CSNK1A1P2, WTAPP1, LOC100288111*	intronic				TR4
rs6969316	7	18955687	42,499	40,571	G>A	0.29	1.02	0.99	1.04	1.6E-01	*HDAC9, NPM1P13*	intronic	SKIN	ESC, ESDR, IPSC	IPSC,IPSC	Hoxb3
rs2244305	21	34652958	42,505	40,568	G>A	0.45	0.99	0.97	1.01	1.6E-01	*IFNAR1, IFNAR2, IL10RB, IL10RB-AS1*	intronic				CTCF,TBX5,Tgif1
rs972400	7	18668239	42,502	40,567	A>T	0.19	0.98	0.96	1.01	1.6E-01	*HDAC9, LOC100419901*	intronic				
rs7910961	10	6077796	42,495	40,569	G>A	0.34	0.99	0.96	1.01	1.6E-01	*IL2RA, RPL32P23*	intronic		4 tissues		SP2,Smad
rs11258943	10	6498723	42,509	40,573	G>A	0.23	0.98	0.96	1.01	1.6E-01	*PRKCQ*	synonymous				GR
rs3024576	16	27358190	42,508	40,574	G>A	0.09	0.98	0.94	1.01	1.6E-01	*IL4R*	intronic	BLD	7 tissues	4 tissues	BDP1
rs2779248	17	26127832	41,589	39,704	A>G	0.37	0.99	0.96	1.01	1.6E-01	*NOS2, LOC645754*		GI	STRM, LIV, GI	GI,GI,GI	
rs3024998	6	43745577	42,499	40,567	G>A	0.32	0.98	0.96	1.01	1.6E-01	*VEGFA*	intronic		10 tissues	GI,BLD	SETDB1
rs17131516	1	92116687	42,510	40,576	G>A	0.08	1.03	0.99	1.07	1.6E-01	*TGFBR3, HSP90B3P*		SKIN	21 tissues	18 tissues	Arid5b,ZBRK1
rs1352947	12	88960727	42,508	40,570	A>G	0.18	0.98	0.96	1.01	1.6E-01	*KITLG*	intronic	GI	10 tissues	5 tissues	4 altered motifs
rs756853	7	18890000	42,508	40,576	A>G	0.42	1.01	0.99	1.04	1.6E-01	*HDAC9*	intronic		MUS, SKIN, BONE	IPSC,SKIN,BRN	Bcl6b,p300
rs1178118	7	18751998	42,502	40,565	A>G	0.19	1.02	0.99	1.04	1.6E-01	*HDAC9*	intronic				15 altered motifs
rs28360493	10	6057774	42,489	40,569	A>C	0.15	1.02	0.99	1.05	1.6E-01	*IL2RA, IL15RA*	intronic		BLD, ADRL		Dobox4,SP1
rs17878498	3	3152930	42,424	40,511	C>A	0.24	0.98	0.96	1.01	1.6E-01	*IL5RA, TRNT1, CRBN*		BLD	4 tissues	6 tissues	9 altered motifs
rs7555183	1	67860627	42,495	40,573	G>A	0.31	1.02	0.99	1.04	1.6E-01	*IL12RB2, SERBP1*	intronic		HRT, MUS		ERalpha-a,Ik-2,RXRA
rs9310940	3	30722218	42,504	40,576	C>A	0.45	0.99	0.97	1.01	1.6E-01	*TGFBR2, GADL1*	intronic	BRN, SKIN	17 tissues	MUS	
rs2010963	6	43738350	42,476	40,555	G>C	0.32	0.98	0.96	1.01	1.6E-01	*VEGFA*	5'-UTR	24 tissues		40 tissues	Irf,NRSF
rs3852257	7	18871692	42,507	40,573	G>A	0.35	1.01	0.99	1.04	1.6E-01	*HDAC9*	intronic		FAT, BRST, SKIN	6 tissues	Pou5f1,Sox,p300
rs3761547		49118461	42,501	40,565	A>G	0.13	1.02	0.99	1.05	1.6E-01	*CACNA1F, CCDC22, FOXP3, PPP1R3F, GAGE10, HSPB1P2*	intronic	BLD	THYM		4 altered motifs
rs10906888	10	6611326	42,498	40,569	A>G	0.35	1.01	0.99	1.04	1.6E-01	*PRKCQ, PRKCQ-AS1*	intronic			ESDR,KID	Rad21
rs340807	3	3115514	42,490	40,558	C>A	0.23	0.98	0.96	1.01	1.6E-01	*IL5RA, CNTN4*	intronic				5 altered motifs
rs17122154	11	118273019	42,500	40,572	G>A	0.26	1.02	0.99	1.04	1.6E-01	*CD3G, KMT2A, UBE4A, ATP5L, MGC13053, LOC100131626*	intronic	24 tissues		26 tissues	
rs1234313	1	173166247	42,505	40,574	G>A	0.32	0.98	0.96	1.01	1.6E-01	*TNFSF4, LOC100506023*	intronic		BLD		CEBPA,CEBPB,STAT
rs10905819	10	6153868	42,504	40,575	G>A	0.45	0.99	0.97	1.01	1.7E-01	*IL2RA, PFKFB3, RBM17, RPL32P23, MIR3155A, MIR3155B*	intronic			SKIN,BLD	PPAR,p300

SNP	Chr	Position[a]	N Cases	N Controls	Allele	MAF	OR[b]	95%CI		*p* -value	Gene annotation	dbSNP functional annotation	Promoter histone marks	Enhancer histone marks	DNAse	Motifs changed
rs12449977	17	25964779	42,424	40,469	G>A	0.24	1.02	0.99	1.04	1.7E-01	*LGALS9, KSR1, NOS2P1, ITM2BP1, LOC100420408*	intronic		ESDR, GI		
rs1892034	10	44907559	42,504	40,568	G>A	0.20	0.98	0.96	1.01	1.7E-01	*CXCL12, RPL9P21*					BCL
rs599190	11	69429136	42,504	40,572	G>A	0.21	1.02	0.99	1.04	1.7E-01	*CCND1, LOC100996515*			ESC, LIV		BDP1
rs1484994	20	30305975	42,508	40,575	A>G	0.30	0.98	0.96	1.01	1.7E-01	*BCL2L1, TPX2*	intronic	8 tissues	17 tissues	5 tissues	4 altered motifs
rs17139675	7	18733748	42,508	40,572	G>A	0.06	1.03	0.99	1.07	1.7E-01	*HDAC9*	intronic		6 tissues	KID,SKIN	Arid3a,XBP-1
rs9658702	10	90757245	42,508	40,577	G>A	0.06	0.97	0.93	1.01	1.7E-01	*ACTA2, FAS, FAS-AS1*	intronic		4 tissues	OVRY	4 altered motifs
rs4721722	7	18755646	42,255	39,820	A>G	0.10	0.98	0.94	1.01	1.7E-01	*HDAC9*	intronic		ESDR, BRN	BRN,BRN	
rs1178108	7	18743747	42,503	40,575	G>A	0.18	1.02	0.99	1.04	1.7E-01	*HDAC9*	intronic		ESDR, HRT, MUS		
rs1547980	2	54120185	42,485	40,537	G>A	0.06	0.97	0.93	1.01	1.7E-01	*GPR75, PSME4, GPR75-ASB3, MIR3682*	intronic				
rs9622682	22	38074434	42,493	40,570	G>A	0.42	0.99	0.97	1.01	1.7E-01	*LGALS1, TRIOBP, SH3BP1, GGA1, PDXP, NOL12*	intronic	BRST, BLD	16 tissues	5 tissues	4 altered motifs
rs3764383	17	76208851	42,482	40,543	A>G	0.30	1.02	0.99	1.04	1.7E-01	*BIRC5, TK1, SYNGR2, AFMID, TMEM235, THA1P, C17orf99, LOC100996291*					4 altered motifs
rs10975121	9	5446078	42,448	40,498	A>G	0.12	1.02	0.99	1.05	1.7E-01	*CD274, PLGRKT, LOC100419687*				7 tissues	10 altered motifs
rs12275686	11	102767396	42,507	40,576	G>A	0.32	0.99	0.96	1.01	1.7E-01	*MMP12, MMP13, LOC100288111*					E4BP4,Pou2f2,TFIIA
rs2227322	17	38171668	42,495	40,566	C>G	0.39	0.99	0.97	1.01	1.7E-01	*CSF3, PSMD3, THRA, MED24, GSDMA, LOC100505620*	5'-UTR	MUS, SKIN, LIV	17 tissues	5 tissues	5 altered motifs
rs1804506	1	92148013	42,506	40,575	G>A	0.16	0.98	0.95	1.01	1.7E-01	*TGFBR3, HSP90B3P*	3'-UTR		ESC, FAT, BLD		
rs822337	9	5449154	41,197	39,381	T>A	0.33	0.99	0.96	1.01	1.7E-01	*CD274, PLGRKT, LOC100419687*		BLD	7 tissues	BLD,BLD	
rs661891	10	6527344	42,491	40,567	C>A	0.48	0.99	0.97	1.01	1.7E-01	*PRKCQ*	intronic		BLD		6 altered motifs
rs2276768	3	30730239	42,507	40,572	G>A	0.11	1.02	0.99	1.06	1.7E-01	*TGFBR2, GADL1*	intronic	GI, CRVX	20 tissues		DMRT4,Myf
rs10486323	7	18963935	42,295	40,327	A>T	0.11	0.98	0.95	1.01	1.7E-01	*HDAC9, NPM1P13*	intronic				HNF1
rs3766730	1	12240676	42,502	40,576	G>A	0.15	1.02	0.99	1.05	1.7E-01	*TNFRSF8, TNFRSF1B, VPS13D, LOC390998, MIR4632*	intronic	BLD, MUS	13 tissues	6 tissues	
rs36017032	11	69430004	42,454	40,448	G>A	0.07	1.03	0.99	1.07	1.7E-01	*CCND1, LOC100996515*			4 tissues	LIV	
rs3024607	16	27363611	42,510	40,576	G>A	0.09	0.98	0.94	1.01	1.7E-01	*IL4R, IL21R*	intronic		BLD	BLD	CIZ
rs3024987	6	43740840	42,507	40,575	G>A	0.12	1.02	0.99	1.05	1.7E-01	*VEGFA*	intronic	19 tissues	7 tissues	GI,MUS	5 altered motifs
rs163551	3	3126050	42,502	40,577	A>G	0.26	1.02	0.99	1.04	1.7E-01	*IL5RA, TRNT1, CNTN4*	intronic		GI	GI	T3R
rs993082	7	19009810	42,406	40,398	G>A	0.17	0.98	0.96	1.01	1.7E-01	*HDAC9, NPM1P13*	intronic				4 altered motifs
rs656388	11	69432680	42,493	40,569	A>T	0.21	1.02	0.99	1.04	1.7E-01	*CCND1, ORAOV1, LOC100996515*			PLCNT, LIV		4 altered motifs
rs2853552	7	18980267	42,504	40,571	G>A	0.29	0.98	0.96	1.01	1.7E-01	*HDAC9, NPM1P13*	intronic	ESDR, ESC, IPSC	5 tissues	ESDR,PANC,VAS	Pou3f3
rs6656018	1	92336084	42,507	40,577	C>A	0.32	0.99	0.96	1.01	1.7E-01	*TGFBR3*	intronic		7 tissues		
rs8041785	15	40251741	42,497	40,562	G>A	0.49	1.01	0.99	1.03	1.7E-01	*GPR176, EIF2AK4, H3F3AP1, LOC100505534*	intronic		BONE	BRN	Mrg,YY1
rs12700007	7	18920523	42,302	40,388	A>T	0.28	1.02	0.99	1.04	1.7E-01	*HDAC9*	intronic				4 altered motifs
rs718798	19	40212119	42,502	40,574	G>A	0.33	1.01	0.99	1.04	1.7E-01	*CLC, LGALS14, LGALS17A, RPS29P27*			BLD		Ets,Hdx,Myc
rs6550009	3	30736551	40,985	38,755	G>A	0.32	1.02	0.99	1.04	1.7E-01	*TGFBR2, GADL1*			5 tissues		HNF1
rs11171806	12	56733531	42,510	40,577	G>A	0.07	0.97	0.93	1.01	1.7E-01	*APOF, CS, STAT2, PAN2, CNPY2, IL23A, LOC100128676, RNU7-40P, LOC100419033*	synonymous	BRST, BLD, THYM	8 tissues	SKIN,THYM	GATA
rs2798631	1	218611878	42,468	40,525	G>A	0.50	1.01	0.99	1.03	1.7E-01	*TGFB2*	intronic				Nanog,RXRA,Sox
rs7532580	1	172583299	42,504	40,565	A>G	0.23	0.98	0.96	1.01	1.7E-01	*FASLG, SUCO*					Hdx,Pou2f2,Pou5f1
rs10489629	1	67688349	42,499	40,569	A>G	0.45	0.99	0.97	1.01	1.7E-01	*IL23R*	intronic				5 altered motifs
rs11780001	8	79654653	42,457	40,465	G>A	0.07	0.97	0.94	1.01	1.7E-01	*IL7, ZC2HC1A, PRKRIRP7*	intronic				6 altered motifs
rs11256557	10	6105836	41,928	40,028	C>A	0.35	0.99	0.96	1.01	1.8E-01	*IL2RA, RBM17, RPL32P23*			BLD		13 altered motifs
rs2395184	6	32432646	42,470	40,534	G>A	0.16	0.98	0.95	1.01	1.8E-01	*HLA-DRA, HLA-DRB9*					BDP1,UF1H3BETA
rs6897932	5	35874575	42,502	40,575	G>A	0.27	1.02	0.99	1.04	1.8E-01	*IL7R, CAPSL*	missense		BLD, THYM		CDP,Maf,Pou2f2
rs11009247	10	33430409	42,508	40,574	A>G	0.10	0.98	0.95	1.01	1.8E-01	*NRP1*			5 tissues	5 tissues	SREBP
rs3213775	2	54150237	41,689	39,555	A>G	0.08	0.97	0.94	1.01	1.8E-01	*PSME4*	synonymous		BRN	SKIN	TCF4
rs11597633	10	6152289	42,509	40,577	G>A	0.10	0.98	0.95	1.01	1.8E-01	*IL2RA, PFKFB3, RBM17, RPL32P23, MIR3155A, MIR3155B*	intronic		6 tissues	BLD	
rs915894	6	32190390	42,478	40,559	A>C	0.36	0.99	0.97	1.01	1.8E-01	*AGER, NOTCH4, PBX2, RNF5, AGPAT1, GPSM3*	missense	5 tissues	10 tissues		Nr2e3,p53
rs6602820	10	6596242	42,506	40,572	A>G	0.43	1.01	0.99	1.03	1.8E-01	*PRKCQ, PRKCQ-AS1*	intronic		BLD		Ets,NRSF
rs11259434	10	6578797	42,507	40,574	A>G	0.11	0.98	0.95	1.01	1.8E-01	*PRKCQ, PRKCQ-AS1*	intronic		BLD, THYM	PLCNT	
rs11259670	10	6622094	41,769	39,664	A>G	0.33	0.99	0.96	1.01	1.8E-01	*PRKCQ, PRKCQ-AS1*	intronic	23 tissues	BRN, PLCNT, SPLN	29 tissues	
rs3025006	6	43747248	42,505	40,568	G>A	0.38	0.99	0.97	1.01	1.8E-01	*VEGFA*	intronic		12 tissues	HRT,SKIN	E2F
rs4658273	1	92274308	42,143	39,919	A>G	0.32	1.01	0.99	1.04	1.8E-01	*TGFBR3*	intronic		10 tissues	PLCNT	16 altered motifs
rs1130233	14	105239894	42,480	40,573	G>A	0.24	1.02	0.99	1.04	1.8E-01	*AKT1, SIVA1, ADSSL1, LINC00638, ZBTB42*	synonymous		5 tissues	5 tissues	NRSF,STAT
rs683878	11	102705658	42,487	40,561	G>C	0.26	1.02	0.99	1.04	1.8E-01	*MMP1, MMP3, MMP12, CSNK1A1P2, WTAPP1, LOC100288111*	intronic		BRST, GI		Ets,Pax-5,TBX5
rs4252287	11	117868638	42,506	40,575	G>A	0.11	0.98	0.95	1.01	1.8E-01	*IL10RA, TMPRSS4-AS1*	intronic	BLD	BLD	BLD,BLD,BLD	BDP1
rs1417488	1	218523730	42,499	40,562	G>A	0.26	0.98	0.96	1.01	1.8E-01	*TGFB2, RRP15, RPS26P17, LOC728463*	intronic	13 tissues	10 tissues	6 tissues	5 altered motifs

SNP	Chr	Position[a]	N Cases	N Controls	Allele	MAF	OR[b]	95%CI		*p* -value	Gene annotation	dbSNP functional annotation	Promoter histone marks	Enhancer histone marks	DNAse	Motifs changed
rs16939967	16	85949473	42,472	40,553	C>A	0.16	0.98	0.95	1.01	1.8E-01	*IRF8*	intronic		10 tissues	4 tissues	7 altered motifs
rs2280883		49109128	42,472	40,536	A>G	0.43	0.99	0.97	1.01	1.8E-01	*CACNA1F, CCDC22, FOXP3, PPP1R3F, HSPB1P2*	intronic		BLD		Rad21
rs12038654	1	172582070	42,503	40,571	A>G	0.23	0.98	0.96	1.01	1.8E-01	*FASLG, SUCO*			MUS		8 altered motifs
rs11465770	1	67633963	42,451	40,495	G>A	0.11	0.98	0.95	1.01	1.8E-01	*IL23R, C1orf141*	intronic	BLD	ADRL		Pou2f2
rs11466512	3	30713126	42,438	40,532	T>A	0.31	1.01	0.99	1.04	1.8E-01	*TGFBR2*	intronic		BLD, PANC		BRCA1,Foxd1,MZF1::1-4
rs4750565	10	6568201	42,502	40,576	G>A	0.12	1.02	0.99	1.05	1.8E-01	*PRKCQ*	intronic		BLD	BLD	Arid5b,p300
rs4656703	1	169688135	42,509	40,576	A>C	0.26	0.98	0.96	1.01	1.8E-01	*SELE, SELL*					
rs7180126	15	40279581	41,593	39,106	A>C	0.49	0.99	0.97	1.01	1.8E-01	*SRP14, EIF2AK4, H3F3AP1*	intronic		LIV	LIV	
rs6684205	1	218609702	41,741	39,338	A>G	0.29	0.99	0.96	1.01	1.8E-01	*TGFB2*	intronic		MUS		HNF1,Pax-4,ZBTB7A
rs1859308	16	27397998	42,509	40,577	G>A	0.14	1.02	0.99	1.05	1.8E-01	*IL4R, IL21R*				LIV	GCM
rs4747844	10	6074201	42,397	40,440	G>A	0.40	1.01	0.99	1.03	1.8E-01	*IL2RA, RPL32P23*	intronic				4 altered motifs
rs6691098	1	92261369	42,479	40,568	A>G	0.23	0.98	0.96	1.01	1.8E-01	*TGFBR3*	intronic	STRM, PANC, GI	14 tissues	15 tissues	
rs12127774	1	92340023	42,506	40,571	G>A	0.33	0.99	0.96	1.01	1.8E-01	*TGFBR3*	intronic		5 tissues		Hand1
rs1178101	7	18737197	42,506	40,574	C>A	0.17	1.02	0.99	1.05	1.9E-01	*HDAC9*	intronic		SKIN		BDP1,p300
rs4252279	11	117867187	42,498	40,572	G>A	0.11	0.98	0.95	1.01	1.9E-01	*IL10RA, TMPRSS4-AS1*	intronic		BLD	BLD,BLD	
rs4820294	22	38071043	42,200	40,222	G>A	0.33	0.99	0.97	1.01	1.9E-01	*LGALS1, TRIOBP, SH3BP1, GGA1, PDXP, NOL12*		22 tissues	11 tissues	52 tissues	8 altered motifs
rs2028015	7	18911061	42,452	40,508	G>A	0.30	1.01	0.99	1.04	1.9E-01	*HDAC9*	intronic				SREBP
rs1045929	17	38175426	42,488	40,567	G>A	0.39	0.99	0.97	1.01	1.9E-01	*CSF3, PSMD3, THRA, MED24, GSDMA, LOC100505620*	3'-UTR			13 tissues	11 altered motifs
rs2187639	11	117818041	42,503	40,576	C>A	0.33	0.99	0.96	1.01	1.9E-01	*IL10RA, TMPRSS13*			7 tissues	11 tissues	ERalpha-a,GR
rs2784101	1	198757361	42,498	40,574	A>G	0.43	0.99	0.97	1.01	1.9E-01	*PTPRC, LOC100131234*			4 tissues	ESDR,THYM	TEF-1
rs3006488	1	153362507	42,507	40,571	A>G	0.11	0.98	0.95	1.01	1.9E-01	*S100A8, S100A9, S100A12, PGLYRP4, S100A7P1, S100A7A, LOC645900, S100A7L2*		BLD, GI	BRST, BLD, SKIN		
rs1178120	7	18754123	42,508	40,574	G>A	0.19	1.02	0.99	1.04	1.9E-01	*HDAC9*	intronic		ESDR	BRN	Smad
rs8140233	22	22177299	42,505	40,573	T>A	0.13	0.98	0.95	1.01	1.9E-01	*MAPK1*	intronic		4 tissues		Foxl1,Foxm1
rs11466521	3	30715977	42,456	40,532	G>A	0.22	0.98	0.96	1.01	1.9E-01	*TGFBR2*	intronic		7 tissues		6 altered motifs
rs111651834	18	59997015	42,507	40,571	G>A	0.06	0.97	0.93	1.01	1.9E-01	*TNFRSF11A, KIAA1468*	intronic	GI, LNG, LIV	18 tissues	37 tissues	4 altered motifs
rs667254	10	6564775	42,500	40,572	G>A	0.27	0.99	0.96	1.01	1.9E-01	*PRKCQ*	intronic		BLD		Ik-1,Ik-2,Lhx3
rs2519890	7	18679534	42,507	40,576	G>A	0.16	0.98	0.95	1.01	1.9E-01	*HDAC9*	intronic		9 tissues	ADRL,BRN,BLD	AP-1,Egr-1
rs16872155	7	19017504	42,502	40,569	A>G	0.34	0.99	0.97	1.01	1.9E-01	*HDAC9, NPM1P13*	intronic	6 tissues	9 tissues	15 tissues	Pou5f1
rs17593222	17	40512990	42,506	40,573	C>G	0.08	1.03	0.99	1.06	1.9E-01	*STAT3, STAT5A, PTRF*	intronic		15 tissues		
rs6602391	10	6078032	42,507	40,573	G>A	0.06	0.97	0.93	1.01	1.9E-01	*IL2RA, RPL32P23*	intronic		5 tissues	THYM	4 altered motifs
rs2116138	3	30602630	42,502	40,568	C>A	0.24	0.98	0.96	1.01	1.9E-01	*TGFBR2*					PPAR
rs948992	11	69513338	42,500	40,574	A>G	0.30	1.01	0.99	1.04	1.9E-01	*CCND1, FGF19, ORAOV1, LOC100129779, LOC100996515*	3'-UTR		ESDR, IPSC, LIV	SKIN	5 altered motifs
rs11256497	10	6087794	42,509	40,577	G>A	0.37	0.99	0.97	1.01	1.9E-01	*IL2RA, RBM17, RPL32P23*	intronic	BLD	BLD, GI, THYM	BLD,BLD	Foxj1,Foxp1,Nkx3
rs4987285	1	169678024	42,506	40,575	A>G	0.26	0.98	0.96	1.01	1.9E-01	*SELE, SELL*	intronic	BLD	BLD	9 tissues	LBP-9,Smad3,Smad4
rs61758464	14	105257802	42,141	40,232	G>A	0.16	1.02	0.99	1.05	1.9E-01	*AKT1, SIVA1, RPS2P4, ADSSL1, LINC00638, ZBTB42, RPS26P49*	intronic		16 tissues	LNG	4 altered motifs
rs8177664	10	6013305	42,508	40,574	A>T	0.07	1.03	0.99	1.07	1.9E-01	*IL2RA, IL15RA, FBXO18*	intronic	4 tissues	9 tissues	20 tissues	Homez
rs2784115	1	198751401	42,506	40,574	G>A	0.28	1.01	0.99	1.04	1.9E-01	*PTPRC, LOC100131234*					UF1H3BETA
rs4672871	2	218945922	42,508	40,573	C>G	0.08	0.98	0.94	1.01	1.9E-01	*CXCR2, CXCR2P1, RUFY4*	intronic	BLD	4 tissues	BLD,BLD	AP-4,Irf
rs9646629	18	60051199	42,492	40,562	G>C	0.36	1.01	0.99	1.04	1.9E-01	*TNFRSF11A, RPL17P44*	intronic			BLD,SKIN	Egr-1,RXRA,Znf143
rs11466491	3	30675151	42,498	40,576	G>A	0.23	0.98	0.96	1.01	1.9E-01	*TGFBR2*	intronic	BLD	11 tissues		ERalpha-a
rs4795856	17	25990033	42,509	40,576	C>A	0.25	1.02	0.99	1.04	1.9E-01	*LGALS9, KSR1, NOS2P1, ITM2BP1, LOC100420408*					Myf,TAL1
rs10214237	5	35883734	42,504	40,575	A>G	0.28	1.01	0.99	1.04	1.9E-01	*IL7R, CAPSL*				SKIN	CTCF,DMRT2,Rad21
rs6550005	3	30650064	42,505	40,570	G>A	0.19	0.98	0.96	1.01	1.9E-01	*TGFBR2*	intronic	21 tissues	5 tissues	13 tissues	AIRE,NF-AT1
rs2276424	11	118209960	42,476	40,566	C>A	0.28	0.99	0.96	1.01	1.9E-01	*CD3D, CD3E, CD3G, UBE4A, LOC100131626*	intronic	BLD, GI	5 tissues	5 tissues	9 altered motifs
rs10482724	1	218520843	42,480	40,554	G>A	0.16	0.98	0.96	1.01	1.9E-01	*TGFB2, RRP15, RPS26P17, LOC728463*	intronic			28 tissues	5 altered motifs
rs833069	6	43742579	42,475	40,535	A>G	0.32	0.99	0.96	1.01	1.9E-01	*VEGFA*	intronic	15 tissues	15 tissues	43 tissues	11 altered motifs
rs11259272	10	6546071	42,464	40,530	G>A	0.46	1.01	0.99	1.03	1.9E-01	*PRKCQ*	intronic		BRST, BLD, SKIN		
rs34431893	22	22194752	42,502	40,530	G>A	0.05	0.97	0.93	1.02	1.9E-01	*MAPK1*	intronic		BLD, SKIN		4 altered motifs
rs10489270	1	173157764	42,491	40,546	G>A	0.12	0.98	0.95	1.01	1.9E-01	*TNFSF4, GOT2P2, LOC100506023*	intronic				Pax-4,Sox
rs2293361	2	54114864	42,508	40,577	A>G	0.06	0.97	0.93	1.01	1.9E-01	*GPR75, PSME4, GPR75-ASB3, MIR3682*	intronic				6 altered motifs
rs7717955	5	35862841	42,488	40,566	G>A	0.27	1.01	0.99	1.04	1.9E-01	*IL7R, SPEF2, CAPSL*	intronic	BLD	BLD, SKIN		AIRE
rs72783721	10	6601916	42,508	40,576	A>G	0.13	1.02	0.99	1.05	1.9E-01	*PRKCQ, PRKCQ-AS1*	intronic		5 tissues	BLD	Foxl1,Foxp1
rs2890658	9	5465130	42,504	40,572	C>A	0.08	1.02	0.99	1.06	1.9E-01	*CD274, PLGRKT, PDCD1LG2, LOC100419687*	intronic		BLD	BLD,BLD	4 altered motifs
rs3737463	11	69512978	42,497	40,566	G>A	0.23	1.02	0.99	1.04	1.9E-01	*CCND1, FGF19, ORAOV1, LOC100129779, LOC100996515*			CRVX		TCF12,TEF-1

SNP	Chr	Position[a]	N Cases	N Controls	Allele	MAF	OR[b]	95%CI		*p* -value	Gene annotation	dbSNP functional annotation	Promoter histone marks	Enhancer histone marks	DNAse	Motifs changed
rs9905566	17	25968300	42,347	40,472	G>A	0.24	1.02	0.99	1.04	1.9E-01	*LGALS9, KSR1, NOS2P1, ITM2BP1, LOC100420408*	intronic		GI, BLD	BLD	Hbp1
rs17433871	1	23016103	42,491	40,562	A>G	0.28	0.99	0.96	1.01	1.9E-01	*C1QA, C1QB, C1QC, EPHB2, MIR4684*		ESDR	11 tissues	24 tissues	19 altered motifs
rs2526614	7	19064020	42,503	40,574	G>A	0.39	0.99	0.97	1.01	1.9E-01	*HDAC9*			8 tissues	11 tissues	HMG-IY
rs17140258	7	18956831	41,759	39,733	G>A	0.20	1.02	0.99	1.04	2.0E-01	*HDAC9, NPM1P13*	intronic		SKIN	SKIN	Nkx3
rs3181148	10	6008030	42,504	40,576	G>A	0.09	1.02	0.99	1.06	2.0E-01	*IL2RA, IL15RA, FBXO18*	intronic		4 tissues	PANC	5 altered motifs
rs2306580	17	40491680	42,508	40,577	C>G	0.08	1.02	0.99	1.06	2.0E-01	*STAT3, STAT5A*	intronic		BLD	BLD	
rs4749882	10	6036144	42,476	40,543	G>A	0.32	1.01	0.99	1.04	2.0E-01	*IL2RA, IL15RA*					TATA
rs3773662	3	30730689	42,508	40,576	A>G	0.05	0.97	0.93	1.02	2.0E-01	*TGFBR2, GADL1*	intronic	7 tissues	18 tissues	22 tissues	Nrf1
rs3014878	1	153343178	42,348	40,474	G>A	0.10	0.98	0.95	1.01	2.0E-01	*S100A8, S100A9, S100A12, PGLYRP4, S100A7A, LOC645900*			4 tissues		Hoxa5
rs4721726	7	18943326	42,501	40,574	G>A	0.23	1.02	0.99	1.04	2.0E-01	*HDAC9, NPM1P13*	intronic		5 tissues	ESDR,MUS,MUS	Pou2f2,Pou5f1
rs6436025	2	218942848	42,509	40,577	G>A	0.08	0.98	0.94	1.01	2.0E-01	*CXCR2, CXCR2P1, RUFY4*	intronic				Egr-1
rs72642637	1	12271183	42,486	40,533	G>A	0.07	0.97	0.94	1.01	2.0E-01	*TNFRSF1B, VPS13D, LOC390998, MIR4632*		BLD	BLD, THYM, SPLN	BLD	YY1,Zbtb3
rs9369324	6	41993007	42,466	40,535	G>A	0.36	1.01	0.99	1.03	2.0E-01	*CCND3, TAF8*	intronic	BLD	8 tissues	BLD	SRF
rs12693993	2	204595597	42,506	40,576	G>A	0.44	0.99	0.97	1.01	2.0E-01	*CD28, KRT18P39, NPM1P33*	intronic		7 tissues	SKIN,PLCNT	HEY1,TATA,TFE
rs661985	10	6436034	42,509	40,576	A>G	0.12	1.02	0.99	1.05	2.0E-01	*PRKCQ, DKFZp667F0711*			4 tissues	GI,BRST	15 altered motifs
rs749794	3	30708432	42,508	40,577	A>G	0.31	1.01	0.99	1.04	2.0E-01	*TGFBR2*	intronic	BLD	18 tissues	7 tissues	4 altered motifs
rs7084816	10	6546018	42,386	40,461	G>A	0.19	1.02	0.99	1.04	2.0E-01	*PRKCQ*	intronic		BRST, BLD, SKIN		Cart1
rs1770372	1	207035647	42,508	40,575	A>G	0.09	0.98	0.94	1.01	2.0E-01	*FAIM3, IL24, IL19, IL20, RPL13AP8*					6 altered motifs
rs7328699	13	28615701	42,507	40,577	G>A	0.12	0.98	0.95	1.01	2.0E-01	*FLT3, LOC100420919*	intronic	BLD	6 tissues	4 tissues	4 altered motifs
rs1545228	8	79644437	42,504	40,574	A>G	0.26	1.01	0.99	1.04	2.0E-01	*IL7, ZC2HC1A, PRKRIRP7*					CDP,Cart1
rs6935031	6	167556667	42,504	40,572	G>A	0.34	0.99	0.97	1.01	2.0E-01	*CCR6, GPR31, TCP10L2*			BLD	PANC	9 altered motifs
rs2799085	1	218552455	42,492	40,562	C>A	0.39	0.99	0.97	1.01	2.0E-01	*TGFB2, RRP15, RPS26P17, LOC728463*	intronic		BRN, SKIN, LNG		EWSR1-FLI1,GR,HDAC2
rs1618130	18	60096200	42,382	40,443	C>A	0.05	1.03	0.98	1.07	2.0E-01	*ACTBP9, TNFRSF11A, RPL17P44*					7 altered motifs
rs4252249	11	117859209	42,508	40,574	G>A	0.11	0.98	0.95	1.01	2.0E-01	*IL10RA, TMPRSS4-AS1*	synonymous	11 tissues	4 tissues	7 tissues	Nkx2
rs1042658	17	38173902	42,487	40,547	G>A	0.39	0.99	0.97	1.01	2.0E-01	*CSF3, PSMD3, THRA, MED24, GSDMA, LOC100505620*	3'-UTR			9 tissues	
rs1411276	1	92257971	42,503	40,575	G>A	0.23	0.98	0.96	1.01	2.0E-01	*TGFBR3*	intronic	SKIN, GI	22 tissues	6 tissues	PLZF,Sp4,Zfp691
rs4721728	7	18970568	42,123	40,115	C>G	0.23	1.02	0.99	1.04	2.0E-01	*HDAC9, NPM1P13*	intronic		SKIN		4 altered motifs
rs1057079	1	11205058	42,497	40,567	A>G	0.26	1.01	0.99	1.04	2.0E-01	*MTOR, EXOSC10, ANGPTL7, MTOR-AS1*	synonymous			BRN,BRN	AFP1,HNF1,LUN-1
rs9579142	13	28589495	42,503	40,573	G>C	0.43	0.99	0.97	1.01	2.0E-01	*CDX2, FLT3, PRHOXNB*	intronic		ESC	IPSC	
rs2201841	1	67694202	41,660	39,296	A>G	0.30	0.99	0.96	1.01	2.0E-01	*IL23R, LOC100130497*	intronic		BLD		Cphx,HEY1,Maf
rs2024022	7	18926103	42,505	40,562	C>G	0.27	1.01	0.99	1.04	2.0E-01	*HDAC9*	intronic				Evi-1
rs4379363	7	18953143	42,503	40,572	A>G	0.21	0.98	0.96	1.01	2.0E-01	*HDAC9, NPM1P13*	intronic		SKIN	MUS,MUS,SKIN	7 altered motifs
rs3793727	10	6508377	42,495	40,568	A>C	0.35	1.01	0.99	1.04	2.1E-01	*PRKCQ*	intronic	KID	ESC, BLD, GI		8 altered motifs
rs3829382	13	28577688	41,933	39,902	A>C	0.49	1.01	0.99	1.03	2.1E-01	*CDX2, FLT3, PRHOXNB, LINC00543*	3'-UTR			KID	Hsf,INSM1,ZBTB7A
rs12232476	17	76169771	42,510	40,573	G>A	0.08	1.02	0.99	1.06	2.1E-01	*BIRC5, EIF5AP2, TK1, SYNGR2, TMC6, AFMID, TMC8, C17orf99*		4 tissues	19 tissues	12 tissues	Mef2
rs4141042	7	19013256	42,505	40,577	A>G	0.14	0.98	0.95	1.01	2.1E-01	*HDAC9, NPM1P13*	intronic		BRST, SKIN		Foxp1
rs2765887	1	92158447	42,499	40,569	A>G	0.39	1.01	0.99	1.03	2.1E-01	*TGFBR3, HSP90B3P*	intronic		6 tissues		8 altered motifs
rs4795816	17	25943895	42,508	40,577	G>A	0.09	1.02	0.99	1.06	2.1E-01	*LGALS9, KSR1, NOS2P1, ITM2BP1*	intronic		BLD, THYM, GI	IPSC	YY1
rs2296126	10	6441755	42,492	40,568	A>G	0.47	0.99	0.97	1.01	2.1E-01	*PRKCQ, DKFZp667F0711*		BLD, LNG	14 tissues	10 tissues	HP1-site-factor,Pax-5
rs2746640	1	11292952	42,493	40,547	A>T	0.27	1.01	0.99	1.04	2.1E-01	*MTOR, ANGPTL7, UBIAD1, RPL39P6, UBE2V2P3*	intronic		LIV		9 altered motifs
rs3024498	1	206941529	42,489	40,564	A>G	0.27	1.01	0.99	1.04	2.1E-01	*IL10, MAPKAPK2, IL19*	3'-UTR	BLD	BLD, THYM	9 tissues	DMRT7
rs11761877	7	18909223	42,487	40,553	G>A	0.30	1.01	0.99	1.04	2.1E-01	*HDAC9*	intronic				BAF155
rs7359586	17	62354992	42,509	40,575	A>G	0.25	1.01	0.99	1.04	2.1E-01	*PECAM1, TEX2, RPL31P57*		MUS	12 tissues	HRT,MUS,MUS	5 altered motifs
rs16822952	1	36888520	42,489	40,548	A>G	0.09	1.02	0.99	1.06	2.1E-01	*CSF3R, MRPS15, STK40, LSM10, OSCP1*	intronic				Pbx3,Pou2f2
rs6441929	3	45924791	42,506	40,575	A>G	0.17	1.02	0.99	1.04	2.1E-01	*CCR9, SDHDP4, LZTFL1, FYCO1*			BLD		Cart1,Irx,Sox
rs2297142	1	92263345	42,509	40,574	A>C	0.23	0.98	0.96	1.01	2.1E-01	*TGFBR3*	intronic		7 tissues	SKIN	5 altered motifs
rs12888392	14	62221983	42,501	40,569	G>A	0.34	1.01	0.99	1.04	2.1E-01	*HIF1A, SNAPC1, HIF1A-AS2*		ESDR	11 tissues	24 tissues	DMRT4,HMG-IY
rs11762171	7	18633033	42,496	40,570	A>G	0.07	1.03	0.99	1.07	2.1E-01	*HDAC9, LOC100419901*	intronic				9 altered motifs
rs9381118	6	41985436	42,508	40,575	G>A	0.26	0.99	0.96	1.01	2.1E-01	*CCND3, TAF8*	intronic		12 tissues	SKIN,BLD	ERalpha-a,Pou2f2,SREBP
rs1461082	3	30701606	42,500	40,567	A>G	0.46	0.99	0.97	1.01	2.1E-01	*TGFBR2*	intronic	BLD	15 tissues		9 altered motifs
rs3826923	19	40188968	42,499	40,574	C>A	0.33	1.01	0.99	1.04	2.1E-01	*CLC, LGALS14, LGALS16, LGALS17A, RPS29P27*			BLD		Mrg,PU.1,Tgif1
rs10874976	1	92267272	42,498	40,568	G>A	0.23	0.98	0.96	1.01	2.1E-01	*TGFBR3*	intronic			LNG	Ik-1,STAT
rs1801157	10	44868257	42,432	40,478	G>A	0.20	0.98	0.96	1.01	2.1E-01	*CXCL12, RPL9P21*	3'-UTR		ESDR, ESC, IPSC		
rs848	5	131996500	42,446	40,515	C>A	0.21	0.98	0.96	1.01	2.1E-01	*IL4, IL13, RAD50, KIF3A*	3'-UTR		5 tissues		5 altered motifs
rs12982225	19	8027754	41,299	39,394	C>A	0.07	0.97	0.94	1.01	2.1E-01	*ELAVL1, MAP2K7, SNAPC2, TIMM44, CTXN1, TGFBR3L*	3'-UTR	LIV	ESDR, LNG, LIV	KID,LIV	6 altered motifs

SNP	Chr	Position[a]	N Cases	N Controls	Allele	MAF	OR[b]	95%CI		*p* -value	Gene annotation	dbSNP functional annotation	Promoter histone marks	Enhancer histone marks	DNAse	Motifs changed
rs6060812	20	30292803	42,506	40,574	G>A	0.22	0.98	0.96	1.01	2.1E-01	*BCL2L1, TPX2*	intronic	LIV, LNG, BRN	18 tissues	15 tissues	Foxp1
rs582052	10	6469155	42,482	40,566	A>C	0.46	1.01	0.99	1.03	2.1E-01	*PRKCQ*	3'-UTR		MUS		4 altered motifs
rs2071171	6	167550042	42,505	40,576	A>G	0.38	0.99	0.97	1.01	2.1E-01	*CCR6, GPR31, TCP10L2*	synonymous	BLD	BLD, STRM, PLCNT	BLD	Pax-8,Pbx3
rs6060633	20	30263317	42,502	40,577	C>A	0.22	0.98	0.96	1.01	2.1E-01	*BCL2L1, COX4I2*	intronic	9 tissues	20 tissues	34 tissues	Ets,PEBP
rs4749955	10	6118966	42,484	40,562	A>G	0.45	1.01	0.99	1.03	2.1E-01	*IL2RA, RBM17, RPL32P23*				BLD	ZNF219
rs9886098	7	19016831	42,445	40,504	G>C	0.41	1.01	0.99	1.03	2.1E-01	*HDAC9, NPM1P13*	intronic		10 tissues	4 tissues	Pax-4
rs2274894	17	26099171	42,501	40,575	C>A	0.40	1.01	0.99	1.03	2.1E-01	*NOS2, LOC645754*	intronic		ESC, IPSC, STRM		4 altered motifs
rs597457	9	101917790	42,502	40,564	C>A	0.19	1.02	0.99	1.04	2.1E-01	*TGFBR1*					AP-2rep,GR,Sin3Ak-20
rs284198	1	92238167	42,508	40,575	C>A	0.36	1.01	0.99	1.03	2.1E-01	*TGFBR3*	intronic		BRST		HNF1
rs12897418	14	105242374	41,366	39,527	G>A	0.12	1.02	0.99	1.05	2.1E-01	*AKT1, SIVA1, ADSSL1, LINC00638, ZBTB42*	intronic		4 tissues	6 tissues	5 altered motifs
rs791588	10	6089342	42,342	40,392	C>A	0.49	0.99	0.97	1.01	2.1E-01	*IL2RA, RBM17, RPL32P23*	intronic		BLD, THYM		4 altered motifs
rs6891095	5	35866322	42,425	40,502	A>G	0.14	1.02	0.99	1.05	2.1E-01	*IL7R, CAPSL*	intronic	BLD	BLD		15 altered motifs
rs1178106	7	18742949	41,637	39,770	A>G	0.30	1.01	0.99	1.04	2.1E-01	*HDAC9*	intronic				4 altered motifs
rs11567694	5	35857704	42,438	40,509	A>G	0.27	1.01	0.99	1.04	2.1E-01	*IL7R, SPEF2, CAPSL*	intronic	12 tissues	10 tissues	CRVX	Otx2
rs1907702	12	88955469	42,510	40,575	A>G	0.20	0.98	0.96	1.01	2.1E-01	*KITLG*	intronic		5 tissues		
rs10988706	9	101870302	42,504	40,572	A>G	0.19	1.02	0.99	1.04	2.1E-01	*COL15A1, TGFBR1*	intronic			BLD,BLD,SKIN	EBF
rs11466511	3	30712827	42,508	40,571	A>C	0.22	0.98	0.96	1.01	2.1E-01	*TGFBR2*	intronic		BLD	BLD	Mxi1,RFX5
rs2049229	3	150307873	42,493	40,574	A>C	0.31	1.01	0.99	1.04	2.1E-01	*SERP1, SELT, EIF2A, LOC677762*			7 tissues	HRT	HP1-site-factor,SETDB1
rs4369774	18	60010448	42,491	40,566	C>A	0.45	1.01	0.99	1.03	2.1E-01	*TNFRSF11A, KIAA1468*	intronic	PANC	ESC, IPSC, KID	BRN	Pax-2,Pax-3,Pax-5
rs12251307	10	6123495	42,506	40,577	G>A	0.12	1.02	0.99	1.05	2.1E-01	*IL2RA, RBM17, RPL32P23*			BLD, HRT		NF-kappaB
rs284170	1	92214628	42,508	40,577	G>A	0.15	0.98	0.96	1.01	2.1E-01	*TGFBR3*	intronic		10 tissues	SKIN,SKIN,SKIN	
rs3858254	10	6518505	42,284	40,334	A>G	0.34	0.99	0.97	1.01	2.1E-01	*PRKCQ*	intronic		BLD, SKIN		Pax-6
rs10988716	9	101886816	42,419	40,501	A>G	0.19	1.02	0.99	1.04	2.2E-01	*TGFBR1*	intronic				11 altered motifs
rs6567282	18	60094992	42,506	40,574	G>A	0.40	1.01	0.99	1.03	2.2E-01	*ACTBP9, TNFRSF11A, RPL17P44*					GR,Pbx-1
rs1295686	5	131995843	42,496	40,570	G>A	0.21	0.98	0.96	1.01	2.2E-01	*IL4, IL13, RAD50, KIF3A*	intronic		4 tissues	BLD	4 altered motifs
rs7100817	10	6474384	42,497	40,564	G>A	0.46	1.01	0.99	1.03	2.2E-01	*PRKCQ*	intronic		GI	LNG	4 altered motifs
rs9863120	3	150292766	42,502	40,568	A>G	0.33	0.99	0.97	1.01	2.2E-01	*SERP1, SELT, EIF2A, LOC677762*	intronic		BLD		DMRT1
rs284200	1	92239336	42,508	40,573	G>A	0.36	1.01	0.99	1.03	2.2E-01	*TGFBR3*	intronic		4 tissues		Foxj1,Nkx2,Nkx3
rs3135369	6	32387221	42,502	40,569	G>A	0.26	0.99	0.96	1.01	2.2E-01	*HLA-DRA, HLA-DRB9, C6orf10, BTNL2, HCG23*			BLD		AP-1,DMRT1
rs10906557	10	6475698	41,686	39,820	A>C	0.46	1.01	0.99	1.03	2.2E-01	*PRKCQ*	intronic				
rs10875001	1	92302252	42,423	40,481	G>A	0.23	0.99	0.96	1.01	2.2E-01	*TGFBR3*	intronic				HNF1,Hoxa10,Hoxd10
rs2512148	11	117873048	42,505	40,576	C>A	0.33	0.99	0.97	1.01	2.2E-01	*IL10RA, TMPRSS4-AS1*		BLD	12 tissues	10 tissues	BDP1
rs7802855	7	19005077	42,502	40,575	A>G	0.40	0.99	0.97	1.01	2.2E-01	*HDAC9, NPM1P13*	intronic				
rs2296139	10	6008172	42,504	40,568	G>A	0.13	1.02	0.99	1.05	2.2E-01	*IL2RA, IL15RA, FBXO18*	synonymous		4 tissues		AhR::Arnt
rs12623380	2	54136714	42,463	40,452	A>G	0.08	0.98	0.94	1.01	2.2E-01	*GPR75, PSME4, GPR75-ASB3*	intronic				6 altered motifs
rs12090296	1	92242568	42,508	40,576	A>G	0.09	1.02	0.99	1.06	2.2E-01	*TGFBR3*	intronic		12 tissues		E2A,RREB-1
rs2104286	10	6099045	42,480	40,556	A>G	0.26	1.01	0.99	1.04	2.2E-01	*IL2RA, RBM17, RPL32P23*	intronic		7 tissues	BLD,BLD	CEBPB
rs74850636	8	128743093	42,506	40,574	A>C	0.08	1.02	0.99	1.06	2.2E-01	*MYC*			ESDR		Nanog
rs3751093	17	25958304	41,953	39,992	G>A	0.21	1.02	0.99	1.04	2.2E-01	*LGALS9, KSR1, NOS2P1, ITM2BP1, LOC100420408*	missense	15 tissues	11 tissues	16 tissues	NF-kappaB,RFX5,Znf143
rs1010447	1	11269796	42,504	40,574	G>A	0.27	1.01	0.99	1.04	2.2E-01	*MTOR, ANGPTL7, RPL39P6*	intronic				
rs1178102	7	18737866	42,504	40,577	G>A	0.17	1.02	0.99	1.04	2.2E-01	*HDAC9*	intronic		GI	LNG,CRVX	BATF,Mef2
rs3773649	3	30716602	42,506	40,574	G>A	0.29	1.01	0.99	1.04	2.2E-01	*TGFBR2*	intronic		6 tissues		4 altered motifs
rs5030772	1	172633350	42,510	40,577	A>G	0.15	0.98	0.95	1.01	2.2E-01	*FASLG*	intronic	BLD	BLD		4 altered motifs
rs12047833	1	153376986	42,484	40,564	A>G	0.11	0.98	0.95	1.01	2.2E-01	*S100A8, S100A9, S100A12, S100A7P1, S100A7A, S100A7P2, LOC645900, S100A7L2*		BLD	IPSC, BLD	4 tissues	NF-kappaB
rs3134942	6	32168771	42,287	40,343	C>A	0.13	0.98	0.95	1.01	2.2E-01	*AGER, NOTCH4, PBX2, RNF5, PPT2, AGPAT1, GPSM3, PRRT1, EGFL8, LOC100507547, PPT2-EGFL8*	synonymous	SKIN	ESDR		5 altered motifs
rs334354	9	101908915	42,505	40,572	G>A	0.19	1.02	0.99	1.04	2.2E-01	*TGFBR1*	intronic		BLD, VAS	BLD	RXRA,UF1H3BETA
rs6950598	7	18971523	42,385	40,446	G>C	0.21	1.02	0.99	1.04	2.2E-01	*HDAC9, NPM1P13*	intronic				HNF6,Nkx6-1
rs10237149	7	18915426	42,509	40,569	G>A	0.39	1.01	0.99	1.03	2.2E-01	*HDAC9*	intronic				4 altered motifs
rs3024548	16	27354531	42,494	40,562	G>C	0.45	0.99	0.97	1.01	2.2E-01	*IL4R*	intronic			BLD	VDR
rs57582212	10	6079035	42,498	40,553	A>C	0.07	0.98	0.94	1.01	2.2E-01	*IL2RA, RPL32P23*	intronic	BLD	7 tissues	5 tissues	5 altered motifs
rs2240279	7	19018009	42,407	40,453	A>G	0.34	0.99	0.97	1.01	2.2E-01	*HDAC9, NPM1P13*	intronic	STRM	12 tissues	20 tissues	Homez,STAT
rs1059703		153278829	42,204	40,316	A>G	0.14	0.98	0.95	1.01	2.2E-01	*HCFC1, IRAK1, MECP2, TMEM187, MIR718, MIR3202-2, MIR3202-1*	missense			PLCNT,LIV	5 altered motifs
rs4072227	1	206957558	42,504	40,574	A>G	0.07	0.98	0.94	1.01	2.2E-01	*IL10, MAPKAPK2, IL19*		BLD	BLD, THYM, GI		Pax-6,Pbx-1,Pou1f1
rs284172	1	92214851	42,508	40,574	T>A	0.15	0.98	0.96	1.01	2.2E-01	*TGFBR3*	intronic		14 tissues		NF-kappaB,Pax-4
rs4940552	18	60056948	42,506	40,574	A>C	0.12	1.02	0.99	1.05	2.2E-01	*TNFRSF11A, RPL17P44*			6 tissues		Mrg1::Hoxa9
rs10214273	5	35883986	42,485	40,552	A>C	0.28	1.01	0.99	1.04	2.2E-01	*IL7R, CAPSL*				CRVX	5 altered motifs

SNP	Chr	Position[a]	N Cases	N Controls	Allele	MAF	OR[b]	95%CI		*p* -value	Gene annotation	dbSNP functional annotation	Promoter histone marks	Enhancer histone marks	DNAse	Motifs changed
rs1571589	9	101883749	42,502	40,575	G>A	0.20	1.02	0.99	1.04	2.3E-01	*TGFBR1*	intronic		7 tissues	BLD	T3R,XBP-1
rs6478972	9	101869278	42,504	40,570	G>A	0.20	1.02	0.99	1.04	2.3E-01	*COL15A1, TGFBR1*	intronic	15 tissues	18 tissues	6 tissues	
rs1047444	3	45960079	42,505	40,568	A>C	0.24	0.99	0.96	1.01	2.3E-01	*CXCR6, CCR9, SDHDP4, FYCO1*	3'-UTR				9 altered motifs
rs721930	22	17585808	42,493	40,563	C>G	0.19	0.98	0.96	1.01	2.3E-01	*IL17RA, CECR6, CECR5, CECR7, RPL31P62, LOC100996342*	intronic		5 tissues		Irf,Pax-2
rs17664969	15	40260570	42,508	40,575	C>A	0.11	0.98	0.95	1.01	2.3E-01	*GPR176, EIF2AK4, H3F3AP1, LOC100505534*	intronic		ESC, HRT, BLD		4 altered motifs
rs4771203	13	28579477	42,495	40,569	A>G	0.22	1.02	0.99	1.04	2.3E-01	*CDX2, FLT3, PRHOXNB, LINC00543*	intronic				6 altered motifs
rs1488373	3	45957689	42,501	40,570	A>G	0.24	0.99	0.96	1.01	2.3E-01	*CXCR6, CCR9, SDHDP4, FYCO1*		5 tissues	15 tissues	14 tissues	
rs2391068	1	92250485	42,509	40,576	G>A	0.09	1.02	0.99	1.06	2.3E-01	*TGFBR3*	intronic	FAT	16 tissues	ADRL,HRT,SKIN	7 altered motifs
rs6441286	3	159728878	42,401	40,498	A>C	0.39	1.01	0.99	1.03	2.3E-01	*IL12A*		BLD	BLD		5 altered motifs
rs3824120	8	128747953	42,506	40,576	C>A	0.12	1.02	0.99	1.05	2.3E-01	*MYC*		24 tissues		51 tissues	
rs1889001	10	6472247	42,504	40,571	C>A	0.46	1.01	0.99	1.03	2.3E-01	*PRKCQ*	intronic				
rs11777205	8	79652134	42,502	40,569	G>A	0.07	0.98	0.94	1.01	2.3E-01	*IL7, ZC2HC1A, PRKRIRP7*	intronic	LIV			5 altered motifs
rs13384671	2	182311594	42,495	40,565	A>G	0.31	1.01	0.99	1.04	2.3E-01	*ITGA4*			BLD, THYM		Bcl6b,Evi-1
rs5353	1	169702974	42,509	40,576	A>G	0.26	0.99	0.96	1.01	2.3E-01	*SELE, SELL*	intronic				5 altered motifs
rs17720964	15	40253886	42,450	40,519	G>A	0.11	0.98	0.95	1.01	2.3E-01	*GPR176, EIF2AK4, H3F3AP1, LOC100505534*	intronic		SKIN		NF-kappaB,TEF-1
rs6969674	7	18963338	42,251	40,233	C>A	0.21	1.02	0.99	1.04	2.3E-01	*HDAC9, NPM1P13*	intronic				AP-1
rs66941654	10	6483433	42,503	40,574	A>G	0.23	0.99	0.96	1.01	2.3E-01	*PRKCQ*	intronic				7 altered motifs
rs3897942	18	60088551	42,492	40,564	C>G	0.47	0.99	0.97	1.01	2.3E-01	*ACTBP9, TNFRSF11A, RPL17P44*		4 tissues	11 tissues	9 tissues	SPIB
rs3927821	8	128774971	42,504	40,565	A>T	0.12	0.98	0.95	1.01	2.3E-01	*MYC, MIR1204*			BLD	5 tissues	Bcl6b,Foxl1
rs2817909	1	23106213	42,495	40,567	A>G	0.07	1.02	0.98	1.07	2.3E-01	*EPHB2*	intronic		6 tissues		PU.1,STAT
rs6686835	1	11305316	42,508	40,570	A>G	0.27	1.01	0.99	1.04	2.3E-01	*MTOR, ANGPTL7, UBIAD1, RPL39P6, UBE2V2P3*	intronic		BLD		9 altered motifs
rs805446	2	54175905	41,194	38,976	G>C	0.27	0.99	0.96	1.01	2.3E-01	*PSME4*	intronic		BLD		Hoxd10,Pou2f2,ZBRK1
rs75570422	10	6478771	42,480	40,555	C>A	0.46	1.01	0.99	1.03	2.3E-01	*PRKCQ*					
rs11780679	8	79658576	42,507	40,577	A>G	0.07	0.98	0.94	1.02	2.3E-01	*IL7, ZC2HC1A, PRKRIRP7*	intronic		FAT	MUS,SKIN	EBF,Ik-1,SREBP
rs7874221	9	101869068	42,478	40,552	A>G	0.19	1.02	0.99	1.04	2.3E-01	*COL15A1, TGFBR1*	intronic	17 tissues	17 tissues	BRST,SKIN	RXRA
rs7920305	10	90760225	42,507	40,571	A>G	0.11	1.02	0.99	1.05	2.3E-01	*ACTA2, FAS, FAS-AS1*	intronic	8 tissues	7 tissues	OVRY	4 altered motifs
rs7893324	10	6111075	42,507	40,575	A>G	0.14	1.02	0.99	1.05	2.3E-01	*IL2RA, RBM17, RPL32P23*		BLD, THYM	8 tissues	BLD,BLD,THYM	6 altered motifs
rs10874981	1	92285875	42,447	40,504	G>A	0.45	0.99	0.97	1.01	2.3E-01	*TGFBR3*	intronic			LNG,GI,CRVX	4 altered motifs
rs4963511	12	6943370	42,506	40,570	G>A	0.14	1.02	0.99	1.05	2.3E-01	*CD4, GNB3, TPI1, USP5, LEPREL2, GPR162, CDCA3, SPSB2, RPL13P5*	intronic	HRT	4 tissues	BLD,BLD	Pax-6
rs11256457	10	6080794	42,499	40,551	G>C	0.39	0.99	0.97	1.01	2.3E-01	*IL2RA, RPL32P23*	intronic		BLD	BLD	5 altered motifs
rs4750528	10	6531552	42,486	40,561	A>G	0.18	1.02	0.99	1.04	2.3E-01	*PRKCQ*	intronic		BLD		Hsf,SP1
rs3024530	16	27350687	42,506	40,575	A>G	0.45	0.99	0.97	1.01	2.3E-01	*IL4R, FLJ21408*	intronic		BLD, GI		
rs17721366	15	40262464	42,497	40,565	A>G	0.11	0.98	0.95	1.01	2.3E-01	*GPR176, EIF2AK4, H3F3AP1, LOC100505534*	intronic		12 tissues	4 tissues	4 altered motifs
rs7805828	7	22758562	42,491	40,565	G>A	0.40	1.01	0.99	1.03	2.3E-01	*IL6, LOC541472*			15 tissues	MUS,PANC	
rs6461386	7	18883690	42,378	40,431	G>A	0.37	1.01	0.99	1.03	2.3E-01	*HDAC9*	intronic		HRT, SKIN		Gfi1,SIX5,p300
rs2717327	7	19064300	42,500	40,571	C>G	0.39	0.99	0.97	1.01	2.3E-01	*HDAC9*		STRM, BRN, BONE	6 tissues	6 tissues	
rs4787956	16	27378249	42,503	40,570	A>G	0.35	0.99	0.97	1.01	2.3E-01	*IL4R, IL21R*		BLD	11 tissues	9 tissues	Egr-1,Mrg1::Hoxa9
rs6967021	7	18958533	42,487	40,560	A>G	0.47	0.99	0.97	1.01	2.3E-01	*HDAC9, NPM1P13*	intronic				AP-1
rs10874940	1	92242186	42,508	40,576	G>A	0.09	1.02	0.99	1.06	2.3E-01	*TGFBR3*	intronic			PANC	HNF1,NF-I
rs17456501	1	12188614	42,509	40,577	G>A	0.06	1.03	0.98	1.07	2.3E-01	*TNFRSF8, TNFRSF1B*	intronic		BLD	PLCNT	CEBPD,Pou3f2
rs11009254	10	33435992	42,500	40,577	G>A	0.11	0.98	0.95	1.01	2.3E-01	*NRP1*			BRN	BRN,BRN	
rs9892152	17	62401965	42,505	40,574	G>A	0.47	1.01	0.99	1.03	2.4E-01	*PECAM1, RPL31P57*	intronic		4 tissues		
rs10213865	5	35857850	41,885	39,608	A>C	0.28	1.01	0.99	1.04	2.4E-01	*IL7R, SPEF2, CAPSL*	intronic			6 tissues	AIRE,GATA,NRSF
rs284874	1	92170644	42,504	40,574	G>A	0.48	1.01	0.99	1.03	2.4E-01	*TGFBR3*	intronic		5 tissues	HRT,OVRY	7 altered motifs
rs12912442	15	40280539	42,496	40,567	G>A	0.11	0.98	0.95	1.01	2.4E-01	*SRP14, EIF2AK4, H3F3AP1*	intronic				4 altered motifs
rs3828069	1	67839573	42,507	40,573	A>G	0.18	1.02	0.99	1.04	2.4E-01	*IL12RB2, SERBP1*	intronic		FAT		AIRE,Hoxa13,Hoxc10
rs1019856	3	30696816	42,507	40,576	G>A	0.14	1.02	0.99	1.05	2.4E-01	*TGFBR2*	intronic			10 tissues	Maf,Pou2f2
rs7536947	1	92248695	42,510	40,577	A>G	0.09	1.02	0.99	1.06	2.4E-01	*TGFBR3*	intronic	GI	19 tissues	VAS	NRSF,Sin3Ak-20
rs2508446	11	117873372	42,495	40,564	G>A	0.33	0.99	0.97	1.01	2.4E-01	*IL10RA, TMPRSS4-AS1*		BLD	12 tissues	23 tissues	NRSF,Roaz
rs10888560	1	153406561	42,510	40,577	G>A	0.07	0.98	0.94	1.02	2.4E-01	*S100A7, S100A8, S100A7P1, S100A7A, S100A7P2, S100A7L2*					RREB-1
rs11009251	10	33434232	42,509	40,576	C>A	0.11	0.98	0.95	1.01	2.4E-01	*NRP1*			7 tissues	BLD	Myf
rs12409415	1	206989608	42,507	40,571	G>A	0.07	0.98	0.94	1.02	2.4E-01	*IL10, IL19, IL20*	intronic		FAT, BLD		Pou1f1,Pou2f2
rs11984041	7	19031935	42,443	40,469	G>A	0.09	1.02	0.99	1.06	2.4E-01	*HDAC9, NPM1P13*	intronic		BRN		
rs16970049	15	40251595	42,510	40,576	A>G	0.05	1.03	0.98	1.07	2.4E-01	*GPR176, EIF2AK4, H3F3AP1, LOC100505534*	intronic		BONE		EWSR1-FLI1,Gfi1,HDAC2
rs2191031	3	45910870	42,496	40,562	G>A	0.19	0.98	0.96	1.01	2.4E-01	*CCR9, SDHDP4, LZTFL1, FYCO1*			BLD, GI, THYM		4 altered motifs
rs10489268	1	173162218	42,477	40,558	A>G	0.20	1.02	0.99	1.04	2.4E-01	*TNFSF4, LOC100506023*	intronic				
rs3024505	1	206939904	42,507	40,576	G>A	0.16	0.98	0.96	1.01	2.4E-01	*IL10, MAPKAPK2, IL19*		BLD, BONE	15 tissues	34 tissues	MZF1::1-4,Pax-4
rs10493858	1	92287937	42,507	40,575	G>C	0.23	0.99	0.96	1.01	2.4E-01	*TGFBR3*	intronic				Hoxa7,Zbtb12

SNP	Chr	Position[a]	N Cases	N Controls	Allele	MAF	OR[b]	95%CI		*p* -value	Gene annotation	dbSNP functional annotation	Promoter histone marks	Enhancer histone marks	DNAse	Motifs changed
rs6972422	7	18834375	42,483	40,554	A>G	0.25	1.01	0.99	1.04	2.4E-01	*HDAC9*	intronic				10 altered motifs
rs1182736	7	18738319	42,475	40,550	G>A	0.17	1.02	0.99	1.04	2.4E-01	*HDAC9*	intronic		GI		DMRT7
rs1108591	17	62405369	42,477	40,558	A>G	0.37	1.01	0.99	1.03	2.4E-01	*PECAM1, RPL31P57*	intronic	CRVX	15 tissues	6 tissues	
rs2027566	1	218558327	42,503	40,573	A>C	0.35	0.99	0.97	1.01	2.4E-01	*TGFB2, RRP15, RPS26P17, LOC728463*	intronic	LNG	11 tissues	7 tissues	5 altered motifs
rs805317	2	54135004	42,509	40,571	T>A	0.45	0.99	0.97	1.01	2.4E-01	*GPR75, PSME4, GPR75-ASB3*	intronic		GI	ESDR	
rs7521065	1	92248978	42,510	40,577	G>A	0.09	1.02	0.99	1.06	2.4E-01	*TGFBR3*	intronic	STRM, GI	17 tissues		
rs10248565	7	18974723	42,423	40,519	A>C	0.13	1.02	0.99	1.05	2.4E-01	*HDAC9, NPM1P13*	intronic				4 altered motifs
rs17759796	22	22190163	42,509	40,577	C>A	0.14	0.98	0.96	1.01	2.4E-01	*MAPK1*	intronic		15 tissues	5 tissues	GATA,Pbx3
rs6777502	3	121869746	42,477	40,534	A>T	0.42	1.01	0.99	1.03	2.4E-01	*CASR, CD86*					4 altered motifs
rs4749926	10	6085312	42,496	40,565	G>A	0.38	0.99	0.97	1.01	2.4E-01	*IL2RA, RBM17, RPL32P23*	intronic		BLD		8 altered motifs
rs1320645	11	76370088	42,480	40,566	C>A	0.49	0.99	0.97	1.01	2.4E-01	*LRRC32, GUCY2EP*	3'-UTR		7 tissues		AP-1,Pou2f2
rs2302519	22	17585441	42,446	40,460	G>A	0.44	1.01	0.99	1.03	2.4E-01	*IL17RA, CECR6, CECR5, CECR7, RPL31P62, LOC100996342*	intronic		IPSC, BLD, SKIN	PANC	BDP1,CAC-binding-protein,Rad21
rs582537	3	159710098	42,494	40,574	C>A	0.43	1.01	0.99	1.03	2.4E-01	*IL12A*	intronic	BLD	4 tissues		7 altered motifs
rs1926262	1	92162784	42,505	40,565	A>C	0.07	0.98	0.94	1.02	2.4E-01	*TGFBR3*	intronic		14 tissues		
rs10760672	9	101873407	42,505	40,568	G>A	0.19	1.02	0.99	1.04	2.4E-01	*COL15A1, TGFBR1*	intronic			SKIN	GR,NF-E2
rs9610487	22	22213969	42,507	40,577	A>G	0.24	1.01	0.99	1.04	2.4E-01	*MAPK1*	intronic	BLD	13 tissues	4 tissues	4 altered motifs
rs6430600	2	136836365	42,506	40,575	G>A	0.29	0.99	0.97	1.01	2.4E-01	*CXCR4*					4 altered motifs
rs3212870	11	69461182	42,506	40,574	G>A	0.11	0.98	0.95	1.01	2.4E-01	*CCND1, ORAOV1, LOC100996515*	intronic		12 tissues	ESDR	PPAR,THAP1
rs10874996	1	92300084	42,504	40,572	G>A	0.23	0.99	0.96	1.01	2.4E-01	*TGFBR3*	intronic			MUS	Nanog,Pou5f1
rs10905599	10	6041923	42,508	40,575	A>G	0.27	1.01	0.99	1.04	2.4E-01	*IL2RA, IL15RA*				ESDR	SIX5
rs10237366	7	18915559	42,501	40,566	G>C	0.39	1.01	0.99	1.03	2.4E-01	*HDAC9*	intronic				GR,NF-AT1
rs11721321	3	121873433	42,508	40,577	G>A	0.42	1.01	0.99	1.03	2.4E-01	*CASR, CD86*					4 altered motifs
rs12929551	16	85953010	42,499	40,576	G>A	0.16	0.98	0.96	1.01	2.4E-01	*IRF8*	intronic		ESDR, BLD, SKIN	BLD,HRT,BLD	NRSF
rs4750351	10	6419765	42,500	40,568	C>A	0.26	1.01	0.99	1.04	2.4E-01	*PRKCQ, LOC399715, DKFZp667F0711, DPPA5P3*					5 altered motifs
rs3773655	3	30722507	42,458	40,372	A>G	0.36	0.99	0.97	1.01	2.4E-01	*TGFBR2, GADL1*	intronic	4 tissues	20 tissues	20 tissues	GR,Maf
rs7176881	15	40266836	42,506	40,573	G>A	0.11	0.98	0.95	1.01	2.4E-01	*EIF2AK4, H3F3AP1, LOC100505534*	intronic		SKIN, HRT, LIV		Cdc5,DMRT2,Pou3f2
rs10112382	8	128784397	42,503	40,571	G>A	0.37	1.01	0.99	1.03	2.5E-01	*MYC, MIR1204*				PLCNT	
rs10223990	7	18979516	42,477	40,530	A>G	0.41	1.01	0.99	1.03	2.5E-01	*HDAC9, NPM1P13*	intronic			LNG,LNG,SKIN	HNF4
rs2391069	1	92250585	42,508	40,577	G>A	0.09	1.02	0.99	1.06	2.5E-01	*TGFBR3*	intronic	FAT	16 tissues	SKIN,ADRL,SKIN	DMRT1,Evi-1,GATA
rs6599263	3	38144875	42,510	40,576	C>A	0.11	1.02	0.99	1.05	2.5E-01	*ACAA1, MYD88, DLEC1*	intronic		BLD	PLCNT	CHOP::CEBPalpha,Nkx3,Pou2f2
rs9607272	22	22136398	42,502	40,571	A>C	0.23	1.01	0.99	1.04	2.5E-01	*MAPK1, YPEL1*	intronic		ESDR, BLD	BLD,BLD,BLD	7 altered motifs
rs2239347	16	27359021	42,497	40,564	A>C	0.46	0.99	0.97	1.01	2.5E-01	*IL4R*	intronic		7 tissues		
rs706779	10	6098824	42,489	40,562	A>G	0.46	0.99	0.97	1.01	2.5E-01	*IL2RA, RBM17, RPL32P23*	intronic		10 tissues		AP-2,MZF1::1-4
rs9355616	6	167557522	42,499	40,571	A>G	0.37	0.99	0.97	1.01	2.5E-01	*CCR6, GPR31, TCP10L2*					4 altered motifs
rs2073964	7	18878150	42,506	40,573	G>A	0.39	1.01	0.99	1.03	2.5E-01	*HDAC9*	intronic				13 altered motifs
rs2116142	3	30717661	42,472	40,570	A>G	0.29	1.01	0.99	1.04	2.5E-01	*TGFBR2*	intronic		7 tissues		CTCFL
rs8177666	10	6011987	42,503	40,572	A>G	0.10	1.02	0.99	1.05	2.5E-01	*IL2RA, IL15RA, FBXO18*	intronic		4 tissues		Arid5b,TCF11::MafG
rs689466	1	186650751	42,507	40,571	A>G	0.19	0.99	0.96	1.01	2.5E-01	*PTGS2*		17 tissues	IPSC, BLD, KID	4 tissues	8 altered motifs
rs1178117	7	18750392	42,189	40,162	C>A	0.28	1.01	0.99	1.04	2.5E-01	*HDAC9*	intronic				Arid3a
rs4658265	1	92240685	42,507	40,571	G>A	0.31	1.01	0.99	1.03	2.5E-01	*TGFBR3*	intronic		10 tissues		Irf,RXRA
rs2270241	22	17566206	42,384	40,487	A>C	0.18	1.02	0.99	1.04	2.5E-01	*IL17RA, CECR6, CECR7, RPL31P62, LOC100996342*	intronic	23 tissues		22 tissues	Pax-5
rs6060763	20	30282223	42,485	40,561	A>G	0.22	0.99	0.96	1.01	2.5E-01	*BCL2L1, TPX2, COX4I2*	intronic		19 tissues	9 tissues	STAT
rs16970052	15	40251932	42,500	40,559	A>G	0.05	1.03	0.98	1.07	2.5E-01	*GPR176, EIF2AK4, H3F3AP1, LOC100505534*	intronic		BONE		DMRT5,RFX5,TEF
rs3771300	2	191835596	42,504	40,575	C>A	0.50	0.99	0.97	1.01	2.5E-01	*GLS, STAT1, LOC100420571*	intronic		4 tissues	VAS	4 altered motifs
rs1042542	17	76221428	42,503	40,571	G>A	0.36	1.01	0.99	1.03	2.5E-01	*BIRC5, TK1, AFMID, TMEM235, THA1P, LOC100996291*	3'-UTR				CHOP::CEBPalpha,Nkx3
rs2706399	5	131867702	42,482	40,525	A>G	0.48	1.01	0.99	1.03	2.5E-01	*IL5, IRF1, RAD50*			BLD, GI		GR,p300
rs894221	8	79649057	42,459	40,508	G>C	0.26	1.01	0.99	1.04	2.5E-01	*IL7, ZC2HC1A, PRKRIRP7*	intronic				GATA
rs11258747	10	6472891	42,507	40,574	C>A	0.23	0.99	0.96	1.01	2.5E-01	*PRKCQ*	synonymous		IPSC	IPSC,IPSC,KID	
rs4658112	1	92208666	42,502	40,573	G>A	0.35	1.01	0.99	1.03	2.5E-01	*TGFBR3*	intronic	GI	17 tissues	15 tissues	4 altered motifs
rs17772583	5	131953510	42,502	40,576	A>G	0.24	1.01	0.99	1.04	2.5E-01	*IL13, RAD50*	intronic				SREBP
rs1117533	7	18947955	42,502	40,564	A>G	0.35	1.01	0.99	1.03	2.5E-01	*HDAC9, NPM1P13*	intronic				5 altered motifs
rs527611	10	6533625	42,471	40,554	A>T	0.40	1.01	0.99	1.03	2.5E-01	*PRKCQ*	intronic	SKIN	4 tissues		Zfp281
rs11580467	1	153377503	42,408	40,504	G>A	0.11	0.98	0.95	1.01	2.5E-01	*S100A8, S100A9, S100A12, S100A7P1, S100A7A, S100A7P2, LOC645900, S100A7L2*		BLD	BLD	ESC,BLD,BLD	GR
rs7534318	1	92248798	42,501	40,567	A>G	0.09	1.02	0.99	1.06	2.5E-01	*TGFBR3*	intronic	GI	19 tissues		
rs3801986	7	18664580	42,452	40,498	A>G	0.49	1.01	0.99	1.03	2.5E-01	*HDAC9, LOC100419901*	intronic				Ets
rs2227324	17	38172192	42,476	40,524	A>G	0.07	1.02	0.98	1.06	2.5E-01	*CSF3, PSMD3, THRA, MED24, GSDMA, LOC100505620*	intronic	6 tissues	15 tissues	5 tissues	PRDM1

SNP	Chr	Position[a]	N Cases	N Controls	Allele	MAF	OR[b]	95%CI		*p* -value	Gene annotation	dbSNP functional annotation	Promoter histone marks	Enhancer histone marks	DNAse	Motifs changed
rs2074633	7	19035920	42,509	40,573	A>G	0.21	1.01	0.99	1.04	2.5E-01	*HDAC9, NPM1P13*	3'-UTR	STRM	9 tissues	11 tissues	PLZF,Pax-5
rs1937339	10	90815975	42,484	40,549	C>A	0.12	0.98	0.95	1.01	2.5E-01	*FAS, MIR4679-1, MIR4679-2*				BRN	5 altered motifs
rs10514611	16	85955242	42,484	40,539	G>A	0.24	1.01	0.99	1.04	2.5E-01	*IRF8*	3'-UTR				TCF4
rs4583693	3	30704619	42,506	40,574	A>G	0.19	0.99	0.96	1.01	2.6E-01	*TGFBR2*	intronic		10 tissues	SKIN	5 altered motifs
rs117748025	8	128747011	42,425	40,504	C>A	0.09	1.02	0.99	1.06	2.6E-01	*MYC*		23 tissues	ESDR	42 tissues	21 altered motifs
rs1041226	10	6643851	42,480	40,547	G>A	0.18	0.98	0.96	1.01	2.6E-01	*PRKCQ, PRKCQ-AS1*				BLD	Cphx,Evi-1,GATA
rs12249398	10	6599396	42,504	40,577	G>A	0.10	0.98	0.95	1.01	2.6E-01	*PRKCQ, PRKCQ-AS1*	intronic		BLD, THYM		TCF11::MafG
rs11256464	10	6082558	42,010	39,963	G>A	0.10	0.98	0.95	1.01	2.6E-01	*IL2RA, RBM17, RPL32P23*	intronic		BLD		Brachyury,TATA
rs500766	10	6550590	42,485	40,552	G>A	0.28	0.99	0.97	1.01	2.6E-01	*PRKCQ*	intronic		BLD, BRN	IPSC	Sox,Zfp187
rs3856847	3	3144246	42,504	40,572	A>T	0.24	0.99	0.96	1.01	2.6E-01	*IL5RA, TRNT1, CRBN, CNTN4*	intronic	BLD	BLD		5 altered motifs
rs11259163	10	6531083	42,493	40,571	T>A	0.24	1.01	0.99	1.04	2.6E-01	*PRKCQ*	intronic				4 altered motifs
rs4326353	8	128790616	42,502	40,573	A>G	0.37	1.01	0.99	1.03	2.6E-01	*MYC, MIR1204*			ADRL, LNG		Evi-1,Osf2,PEBP
rs2765881	1	92141006	42,398	40,450	C>A	0.40	1.01	0.99	1.03	2.6E-01	*TGFBR3, HSP90B3P*			FAT		Foxp3,Myc,Pou5f1
rs10775357	17	76238523	42,461	40,543	C>G	0.37	1.01	0.99	1.03	2.6E-01	*BIRC5, AFMID, TMEM235, THA1P, LOC100996291*				16 tissues	
rs17434924	7	18929062	42,504	40,574	A>G	0.24	1.01	0.99	1.04	2.6E-01	*HDAC9*	intronic		FAT, HRT, VAS		6 altered motifs
rs17720724	15	40250008	42,508	40,577	A>G	0.11	0.98	0.95	1.01	2.6E-01	*GPR176, EIF2AK4, H3F3AP1, LOC100505534*	intronic				AIRE
rs658230	10	6508563	42,507	40,574	G>A	0.49	0.99	0.97	1.01	2.6E-01	*PRKCQ*	intronic		4 tissues		Ets,HNF4,Pax-4
rs10988719	9	101895482	42,509	40,577	A>T	0.09	0.98	0.95	1.01	2.6E-01	*TGFBR1*	intronic			SKIN	10 altered motifs
rs1464517	3	150342895	42,495	40,566	G>A	0.32	1.01	0.99	1.03	2.6E-01	*SELT, EIF2A, FAM194A, LOC677762*	intronic			ESDR	RXRA
rs36234022	2	191879139	42,501	40,570	A>G	0.14	1.02	0.99	1.05	2.6E-01	*GLS, STAT1, STAT4, LOC100420571*		23 tissues	ESDR	53 tissues	10 altered motifs
rs4252246	11	117858185	42,505	40,575	C>A	0.11	0.98	0.95	1.01	2.6E-01	*IL10RA, TMPRSS4-AS1*	intronic	10 tissues	4 tissues	9 tissues	4 altered motifs
rs13245206	7	18891259	42,493	40,561	G>A	0.39	1.01	0.99	1.03	2.6E-01	*HDAC9*	intronic		ESC		4 altered motifs
rs6670134	1	67592114	42,509	40,573	G>A	0.24	0.99	0.96	1.01	2.6E-01	*IL23R, C1orf141*	intronic	BLD	5 tissues	4 tissues	7 altered motifs
rs1017956	1	92314691	42,506	40,575	T>A	0.45	0.99	0.97	1.01	2.6E-01	*TGFBR3*	intronic			MUS	4 altered motifs
rs7211218	17	76236954	42,509	40,575	C>A	0.08	1.02	0.98	1.06	2.6E-01	*BIRC5, AFMID, TMEM235, THA1P, LOC100996291*	3'-UTR		BRN, THYM, LIV	CRVX	ATF2,Ik-2
rs2282284	1	157648543	42,222	39,876	A>G	0.06	0.98	0.93	1.02	2.6E-01	*FCRL3, SONP1, VDAC1P9*	missense		BRST		
rs801532	7	18661156	41,792	39,823	G>A	0.11	0.98	0.95	1.01	2.6E-01	*HDAC9, LOC100419901*	intronic				6 altered motifs
rs4934434	10	90747169	42,507	40,573	C>A	0.40	1.01	0.99	1.03	2.6E-01	*ACTA2, FAS, ACTA2-AS1, FAS-AS1*	intronic	BLD	8 tissues	7 tissues	GCNF,HNF4
rs2227338	17	38172942	42,501	40,574	G>A	0.07	1.02	0.98	1.06	2.6E-01	*CSF3, PSMD3, THRA, MED24, GSDMA, LOC100505620*	intronic	SKIN, LNG, MUS	15 tissues	MUS	4 altered motifs
rs10760670	9	101872163	42,473	40,542	G>A	0.20	1.01	0.99	1.04	2.6E-01	*COL15A1, TGFBR1*	intronic			5 tissues	BCL,Cdx2,STAT
rs1852211	3	119274749	42,490	40,537	G>A	0.21	0.99	0.96	1.01	2.6E-01	*ADPRH, CD80, CSRP2P, TIMMDC1, PLA1A*	intronic	BLD	8 tissues		Smad
rs11571317	2	204732008	42,499	40,559	G>A	0.08	1.02	0.98	1.06	2.6E-01	*CTLA4*		BLD	BLD, SKIN, THYM		Irf,Sp100
rs6550004	3	30649907	42,501	40,568	A>C	0.19	0.99	0.96	1.01	2.6E-01	*TGFBR2*	intronic	22 tissues	BRN, SKIN, HRT	19 tissues	E4BP4,Gmeb1,Pax-4
rs11603541	11	69472373	42,486	40,571	G>C	0.11	0.98	0.95	1.01	2.6E-01	*CCND1, FGF19, ORAOV1, LOC100996515*			13 tissues	ESDR,LIV	5 altered motifs
rs611003	11	69445284	42,494	40,565	A>C	0.46	0.99	0.97	1.01	2.6E-01	*CCND1, ORAOV1, LOC100996515*			GI	GI	
rs6473118	8	79701683	42,477	40,548	G>A	0.07	0.98	0.94	1.02	2.6E-01	*IL7, PRKRIRP7*	intronic		4 tissues		4 altered motifs
rs17435661	7	19017368	42,510	40,576	A>G	0.07	0.98	0.94	1.02	2.6E-01	*HDAC9, NPM1P13*	intronic	7 tissues	10 tissues	10 tissues	Foxp1,Gfi1,Sox
rs8086340	18	60006978	42,498	40,569	C>G	0.45	1.01	0.99	1.03	2.6E-01	*TNFRSF11A, KIAA1468*	intronic	6 tissues	14 tissues	21 tissues	Foxm1
rs2071370	17	38172452	42,508	40,574	G>A	0.07	1.02	0.98	1.06	2.6E-01	*CSF3, PSMD3, THRA, MED24, GSDMA, LOC100505620*	intronic	4 tissues	15 tissues	IPSC,MUS	
rs2069840	7	22768572	42,504	40,571	G>C	0.33	1.01	0.99	1.03	2.6E-01	*IL6, RPS26P32, LOC541472*	intronic	4 tissues	6 tissues		GCNF,RXRA
rs2000220	1	218587636	42,492	40,565	A>G	0.41	0.99	0.97	1.01	2.6E-01	*TGFB2*	intronic	MUS	4 tissues	6 tissues	AP-2,CEBPA,Sp4
rs12899649	15	40281684	42,508	40,577	A>G	0.11	0.98	0.95	1.01	2.7E-01	*SRP14, EIF2AK4, H3F3AP1, SRP14-AS1*	intronic				Gfi1,Hdx,Spz1
rs10819638	9	101874314	42,505	40,574	G>A	0.19	1.01	0.99	1.04	2.7E-01	*COL15A1, TGFBR1*	intronic			ESDR,BLD	
rs17160155	11	76322550	42,505	40,576	A>G	0.15	0.98	0.96	1.01	2.7E-01	*LRRC32*			13 tissues	4 tissues	CCNT2
rs960326	1	207014776	42,504	40,564	A>G	0.09	0.98	0.95	1.02	2.7E-01	*IL19, IL20*	intronic		SKIN, BLD		Myc
rs10733709	9	101902346	42,506	40,574	G>A	0.19	1.01	0.99	1.04	2.7E-01	*TGFBR1*	intronic			BLD	Ets
rs4938506	11	118205702	42,495	40,564	G>A	0.25	0.99	0.96	1.01	2.7E-01	*CD3D, CD3E, CD3G, UBE4A, LOC100131626*		BLD	BLD, THYM	5 tissues	10 altered motifs
rs2073962	7	18833355	42,507	40,575	A>T	0.16	1.02	0.99	1.04	2.7E-01	*HDAC9*	intronic	STRM, VAS, GI	6 tissues		Mef2,ZEB1
rs10905669	10	6092093	42,500	40,568	G>A	0.23	0.99	0.96	1.01	2.7E-01	*IL2RA, RBM17, RPL32P23*	intronic			4 tissues	5 altered motifs
rs4983540	14	105208567	42,393	40,458	G>A	0.19	1.01	0.99	1.04	2.7E-01	*AKT1, SIVA1, INF2, ADSSL1, LOC100996409*	intronic		4 tissues		Gm397,Mtf1,Nrf1
rs4714695	6	43711459	42,504	40,570	G>A	0.19	1.01	0.99	1.04	2.7E-01	*VEGFA, LOC100132242*			10 tissues	MUS,MUS	AP-2rep,NF-kappaB,Rad21
rs2182423	1	92165626	42,419	40,446	G>A	0.40	1.01	0.99	1.03	2.7E-01	*TGFBR3*	intronic	FAT, SKIN, BONE	12 tissues	SKIN,MUS,SKIN	9 altered motifs
rs4710190	6	167559486	42,500	40,572	A>G	0.37	0.99	0.97	1.01	2.7E-01	*CCR6, GPR31, TCP10L2*			4 tissues		5 altered motifs
rs1178122	7	18763279	42,475	40,547	A>G	0.21	1.01	0.99	1.04	2.7E-01	*HDAC9*	intronic				CEBPB,DBP,Foxp3
rs2106506	7	18630523	42,505	40,573	A>C	0.16	0.98	0.96	1.01	2.7E-01	*HDAC9, LOC100419901*	intronic		SKIN	34 tissues	
rs10912551	1	173119967	42,498	40,572	C>A	0.22	0.99	0.96	1.01	2.7E-01	*TNFSF4, GOT2P2*					PU.1
rs11775538	8	79703113	42,098	40,109	A>G	0.06	0.98	0.94	1.02	2.7E-01	*IL7, PRKRIRP7*	intronic		FAT, MUS		GR,IRC900814
rs739719	5	131872865	42,510	40,577	C>A	0.07	0.98	0.94	1.02	2.7E-01	*IL5, IRF1, RAD50*					5 altered motifs

SNP	Chr	Position[a]	N Cases	N Controls	Allele	MAF	OR[b]	95%CI		*p* -value	Gene annotation	dbSNP functional annotation	Promoter histone marks	Enhancer histone marks	DNAse	Motifs changed
rs7798677	7	18822963	42,424	40,507	G>A	0.16	1.02	0.99	1.04	2.7E-01	*HDAC9*	intronic		BRST, SKIN		CEBPB,Ncx,Sox
rs3806680	3	3152384	42,503	40,577	A>G	0.25	0.99	0.96	1.01	2.7E-01	*IL5RA, TRNT1, CRBN*		6 tissues	12 tissues	BLD,BLD	
rs1800682	10	90749963	42,500	40,570	A>G	0.46	0.99	0.97	1.01	2.7E-01	*ACTA2, FAS, FAS-AS1*	intronic	22 tissues	8 tissues	37 tissues	Hsf,STAT,ZBRK1
rs6503691	17	40394090	42,508	40,575	G>A	0.10	0.98	0.95	1.01	2.7E-01	*STAT5A, STAT5B, GHDC*	intronic		7 tissues	BLD,BRN,PLCNT	Hoxc9,Pdx1
rs2071369	17	38172305	42,507	40,574	G>A	0.07	1.02	0.98	1.06	2.7E-01	*CSF3, PSMD3, THRA, MED24, GSDMA, LOC100505620*	intronic	5 tissues	16 tissues	7 tissues	Pou2f2
rs1011446	7	18811610	42,504	40,572	A>G	0.18	1.01	0.99	1.04	2.7E-01	*HDAC9*	intronic			4 tissues	Pou2f2
rs13397		153248248	42,498	40,569	G>A	0.13	0.98	0.95	1.01	2.7E-01	*HCFC1, IRAK1, MECP2, RENBP, NAA10, TMEM187, MIR718, MIR3202-2, MIR3202-1*	synonymous	BLD	6 tissues	BLD,BLD	CEBPB
rs1463513	3	30694135	42,510	40,574	A>T	0.14	1.02	0.99	1.05	2.7E-01	*TGFBR2*	intronic		BLD, VAS	BLD	Nkx2
rs10899252	11	76400134	42,480	40,558	G>A	0.15	0.98	0.96	1.01	2.7E-01	*LRRC32, GUCY2EP*				BLD	4 altered motifs
rs929039	22	38071511	42,476	40,538	A>G	0.33	0.99	0.97	1.01	2.7E-01	*LGALS1, TRIOBP, SH3BP1, GGA1, PDXP, NOL12*		23 tissues	10 tissues	51 tissues	
rs6808378	3	3142943	42,502	40,568	G>A	0.24	0.99	0.96	1.01	2.7E-01	*IL5RA, TRNT1, CRBN, CNTN4*	intronic		BLD		BCL
rs4364968	10	6503731	42,504	40,575	C>A	0.39	1.01	0.99	1.03	2.7E-01	*PRKCQ*	intronic				Pou2f2,STAT
rs10760671	9	101872674	42,499	40,572	G>A	0.19	1.01	0.99	1.04	2.7E-01	*COL15A1, TGFBR1*	intronic			SKIN	Cart1,Pou6f1
rs1178103	7	18741367	42,480	40,550	A>C	0.19	1.01	0.99	1.04	2.7E-01	*HDAC9*	intronic			ESC	GR,Irx
rs72931569	18	59996720	42,510	40,577	G>A	0.07	1.02	0.98	1.06	2.7E-01	*TNFRSF11A, KIAA1468*	intronic	GI, LIV	17 tissues	4 tissues	
rs10257046	7	18720135	42,507	40,576	G>A	0.08	0.98	0.95	1.02	2.7E-01	*HDAC9*	intronic			SKIN	YY1
rs17139842	7	18800697	42,507	40,573	G>C	0.17	1.01	0.99	1.04	2.8E-01	*HDAC9*	intronic		ESDR, STRM, BRN		E2A,LBP-1,ZEB1
rs13431828	2	102954653	42,507	40,574	G>A	0.14	0.98	0.96	1.01	2.8E-01	*IL18R1, IL1RL1*	5'-UTR	HRT, VAS	6 tissues	6 tissues	LXR
rs12164905	15	40300691	42,481	40,565	C>A	0.18	1.01	0.99	1.04	2.8E-01	*SRP14, EIF2AK4, SRP14-AS1*	intronic		6 tissues	MUS	HNF4,LXR,RXRA
rs12359875	10	6051107	42,507	40,572	G>A	0.24	0.99	0.96	1.01	2.8E-01	*IL2RA, IL15RA*		7 tissues	4 tissues	LNG,LNG	SREBP
rs3767576	1	92166931	42,503	40,575	G>A	0.19	0.99	0.96	1.01	2.8E-01	*TGFBR3*	intronic	FAT, STRM	7 tissues	9 tissues	4 altered motifs
rs805341	2	54192157	42,448	40,458	A>G	0.48	0.99	0.97	1.01	2.8E-01	*PSME4*	intronic	BLD, SKIN	16 tissues	BRST,BLD,BRST	4 altered motifs
rs2508445	11	117872544	42,505	40,576	C>A	0.33	0.99	0.97	1.01	2.8E-01	*IL10RA, TMPRSS4-AS1*		BLD	9 tissues	14 tissues	Rhox11
rs11256335	10	6055222	42,509	40,577	T>A	0.24	0.99	0.96	1.01	2.8E-01	*IL2RA, IL15RA*	intronic			BLD,THYM	STAT
rs1017957	1	92314131	42,496	40,564	A>G	0.45	0.99	0.97	1.01	2.8E-01	*TGFBR3*	intronic				GR,NF-E2,Nrf-2
rs7779024	7	18822722	42,487	40,566	T>A	0.16	1.02	0.99	1.04	2.8E-01	*HDAC9*	intronic		BRST, SKIN		
rs2634031	1	92313638	42,500	40,572	G>A	0.45	0.99	0.97	1.01	2.8E-01	*TGFBR3*	intronic		13 tissues	IPSC,PLCNT	4 altered motifs
rs6683473	1	206967152	42,501	40,576	G>A	0.23	1.01	0.99	1.04	2.8E-01	*IL10, IL19*			4 tissues	BLD,LNG	Brachyury,TBX5
rs1278150	3	3165907	42,502	40,570	A>G	0.50	0.99	0.97	1.01	2.8E-01	*IL5RA, TRNT1, CRBN*					HNF4
rs519806	11	102675489	42,508	40,573	A>G	0.41	0.99	0.97	1.01	2.8E-01	*MMP1, MMP3, MMP10, CSNK1A1P2, WTAPP1, LOC100421658*	intronic				Pax-4,Pax-6,TATA
rs1077667	19	6668972	42,502	40,572	G>A	0.21	0.99	0.96	1.01	2.8E-01	*C3, TNFSF14*	intronic			5 tissues	p53
rs17433319	7	18747178	42,508	40,577	G>A	0.11	1.02	0.99	1.05	2.8E-01	*HDAC9*	intronic		BLD, HRT		Pou2f2
rs9554252	13	28670039	42,509	40,574	G>A	0.25	0.99	0.96	1.01	2.8E-01	*FLT3, PAN3, CHCHD2P8, PAN3-AS1, LOC100420919*	intronic	BLD	BLD		DMRT2,HNF6
rs6667860	1	36958213	42,485	40,559	A>G	0.46	0.99	0.97	1.01	2.8E-01	*CSF3R, MRPS15, OSCP1*		BLD	4 tissues	4 tissues	5 altered motifs
rs3762760	3	3188016	42,502	40,573	A>G	0.18	1.01	0.99	1.04	2.8E-01	*IL5RA, TRNT1, CRBN*	intronic		FAT, STRM		4 altered motifs
rs8177751	10	6029759	42,502	40,568	T>A	0.16	0.99	0.96	1.01	2.8E-01	*IL2RA, IL15RA*		BRN, BLD	4 tissues	4 tissues	4 altered motifs
rs1508595	12	88986016	42,504	40,566	G>A	0.16	0.99	0.96	1.01	2.8E-01	*KITLG*					6 altered motifs
rs10905668	10	6092055	42,507	40,573	G>A	0.23	0.99	0.96	1.01	2.8E-01	*IL2RA, RBM17, RPL32P23*	intronic			7 tissues	AP-4,BATF
rs2476491	10	6095410	42,483	40,550	A>T	0.29	0.99	0.97	1.01	2.8E-01	*IL2RA, RBM17, RPL32P23*	intronic	BLD	11 tissues	4 tissues	Sox,TCF4
rs917864	22	17565932	42,506	40,571	G>A	0.18	1.01	0.99	1.04	2.8E-01	*IL17RA, CECR6, CECR7, RPL31P62, LOC100996342*	5'-UTR	24 tissues		49 tissues	MIZF
rs2505741	10	44823136	42,497	40,572	A>G	0.45	1.01	0.99	1.03	2.8E-01	*CXCL12, LOC100130539*			PANC	KID	DMRT1,ERalpha-a
rs4601580	1	154394417	42,320	40,388	T>A	0.45	1.01	0.99	1.03	2.8E-01	*IL6R, MRPS33P1, RPSAP17, PSMD8P1*	intronic		14 tissues		25 altered motifs
rs4103200	17	40507065	42,506	40,569	G>C	0.28	1.01	0.99	1.04	2.8E-01	*STAT3, STAT5A, PTRF*	intronic	BLD	11 tissues	13 tissues	
rs7043624	9	101883028	42,472	40,545	C>G	0.21	1.01	0.99	1.04	2.8E-01	*COL15A1, TGFBR1*	intronic		8 tissues	BLD	
rs913059	1	92149836	42,504	40,575	A>C	0.26	1.01	0.99	1.04	2.8E-01	*TGFBR3, HSP90B3P*	intronic		14 tissues	MUS,OVRY,SKIN	7 altered motifs
rs420549	9	101914873	42,506	40,572	C>G	0.19	1.01	0.99	1.04	2.8E-01	*TGFBR1*	3'-UTR		LNG		
rs10264621	7	18957353	42,495	40,567	C>A	0.20	1.01	0.99	1.04	2.8E-01	*HDAC9, NPM1P13*	intronic				GATA
rs1467198	2	191880470	42,509	40,577	A>G	0.33	0.99	0.97	1.01	2.8E-01	*STAT1, STAT4, LOC100420571*		BLD	BLD, THYM	BLD,BLD,BLD	Foxp1,Mef2,RREB-1
rs957960	7	18877408	42,491	40,551	C>A	0.38	1.01	0.99	1.03	2.8E-01	*HDAC9*	intronic				10 altered motifs
rs6461387	7	18898305	42,503	40,573	G>A	0.40	1.01	0.99	1.03	2.8E-01	*HDAC9*	intronic		SKIN		Lhx4,NF-Y,OTX
rs1178169	7	18792940	42,500	40,568	A>G	0.18	1.01	0.99	1.04	2.8E-01	*HDAC9*	intronic		5 tissues	4 tissues	E2A
rs13515	22	22115886	42,465	40,535	G>A	0.19	0.99	0.96	1.01	2.8E-01	*MAPK1, YPEL1*	3'-UTR		6 tissues	BLD	AP-1,GR
rs2297518	17	26096597	42,495	40,565	G>A	0.19	0.99	0.96	1.01	2.8E-01	*NOS2, LOC645754*	missense		MUS		HMG-IY,STAT
rs8069645	17	40494902	42,506	40,574	A>G	0.28	1.01	0.99	1.03	2.8E-01	*STAT3, STAT5A*	intronic		13 tissues	18 tissues	Irf,STAT
rs1034805	7	18977653	42,504	40,572	G>A	0.33	1.01	0.99	1.03	2.8E-01	*HDAC9, NPM1P13*	intronic			IPSC	11 altered motifs

SNP	Chr	Position[a]	N Cases	N Controls	Allele	MAF	OR[b]	95%CI		*p* -value	Gene annotation	dbSNP functional annotation	Promoter histone marks	Enhancer histone marks	DNAse	Motifs changed
rs2408490	11	102672553	42,465	40,546	G>A	0.16	1.01	0.99	1.04	2.8E-01	*MMP1, MMP3, MMP10, CSNK1A1P2, WTAPP1, LOC100421658*	intronic		5 tissues		
rs10760673	9	101878622	42,506	40,575	G>A	0.19	1.01	0.99	1.04	2.8E-01	*COL15A1, TGFBR1*	intronic			17 tissues	Hsf,TFIIA
rs16966545	17	26104622	42,495	40,566	T>A	0.20	0.99	0.96	1.01	2.8E-01	*NOS2, LOC645754*	intronic		5 tissues	SKIN	AhR,Ehf,Spdef
rs4655692	1	67691665	42,500	40,573	G>A	0.25	1.01	0.99	1.04	2.8E-01	*IL23R*	intronic		5 tissues		4 altered motifs
rs12709500	17	26134974	42,495	40,568	A>G	0.28	1.01	0.99	1.03	2.8E-01	*NOS2*		BLD, GI, HRT	14 tissues	BLD	Hsf,Pax-5
rs12244380	10	6053374	42,494	40,570	A>G	0.42	0.99	0.97	1.01	2.8E-01	*IL2RA, IL15RA*	3'-UTR		LNG		FAC1,NF-I
rs3856850	3	3151346	42,505	40,572	A>G	0.26	1.01	0.99	1.04	2.8E-01	*IL5RA, TRNT1, CRBN*	intronic	4 tissues	ESC, IPSC, BLD	4 tissues	6 altered motifs
rs25882	5	131411460	42,499	40,569	A>G	0.22	0.99	0.96	1.01	2.9E-01	*CSF2, IL3*	missense	BLD	6 tissues	BLD	GATA
rs155149	2	182329647	42,485	40,508	C>A	0.22	0.99	0.96	1.01	2.9E-01	*ITGA4*	intronic	THYM, BLD	5 tissues		
rs1872978	3	30696251	42,509	40,576	G>A	0.14	1.02	0.99	1.05	2.9E-01	*TGFBR2*	intronic	BLD	15 tissues	BLD	5 altered motifs
rs868	9	101911656	42,508	40,573	A>G	0.19	1.01	0.99	1.04	2.9E-01	*TGFBR1*	3'-UTR		6 tissues		Myb,Sox
rs10283455	9	101905643	42,506	40,575	C>A	0.19	1.01	0.99	1.04	2.9E-01	*TGFBR1*	intronic			ADRL	GATA,MIF-1,Nrf-2
rs12722606	10	6053133	42,501	40,571	G>A	0.24	0.99	0.96	1.01	2.9E-01	*IL2RA, IL15RA*	3'-UTR				6 altered motifs
rs2214850	7	18959138	42,434	40,498	A>G	0.13	1.02	0.99	1.05	2.9E-01	*HDAC9, NPM1P13*	intronic				4 altered motifs
rs62626326	10	6121286	42,510	40,577	A>G	0.06	1.02	0.98	1.07	2.9E-01	*IL2RA, RBM17, RPL32P23*					GR
rs17887110	3	3152841	42,506	40,568	A>G	0.15	1.02	0.99	1.04	2.9E-01	*IL5RA, TRNT1, CRBN*		BLD	4 tissues	6 tissues	RFX5,YY1
rs522616	11	102715048	42,506	40,576	A>G	0.20	1.01	0.99	1.04	2.9E-01	*MMP1, MMP3, MMP12, CSNK1A1P2, WTAPP1, LOC100288111*			SKIN	SKIN	5 altered motifs
rs11165594	1	92302369	42,381	40,399	A>C	0.22	0.99	0.96	1.01	2.9E-01	*TGFBR3*	intronic		FAT, GI, HRT	4 tissues	4 altered motifs
rs7082071	10	6470587	42,501	40,576	G>A	0.23	0.99	0.96	1.01	2.9E-01	*PRKCQ*	intronic				Arid5a,Dobox4,Foxp1
rs9872641	3	150266026	42,491	40,561	A>G	0.36	0.99	0.97	1.01	2.9E-01	*SERP1, EIF2A*	intronic	12 tissues	13 tissues	MUS,BLD	CEBPA,CEBPB
rs12536836	7	18881075	42,488	40,551	G>A	0.40	1.01	0.99	1.03	2.9E-01	*HDAC9*	intronic				16 altered motifs
rs35602605	15	40308859	42,497	40,572	C>A	0.18	1.01	0.99	1.04	2.9E-01	*SRP14, EIF2AK4, SRP14-AS1*	missense		FAT, MUS	11 tissues	
rs1178170	7	18793813	42,508	40,574	A>C	0.18	1.01	0.99	1.04	2.9E-01	*HDAC9*	intronic	6 tissues	LNG, STRM, MUS	10 tissues	Hbp1,Myc,Nkx3
rs16906090	8	79689755	42,509	40,576	G>A	0.07	0.98	0.94	1.02	2.9E-01	*IL7, PRKRIRP7*	intronic				
rs2071214	17	76219591	42,509	40,576	A>G	0.05	1.02	0.98	1.07	2.9E-01	*BIRC5, TK1, AFMID, TMEM235, THA1P, LOC100996291*	missense		9 tissues	BLD,MUS,VAS	
rs10506957	12	88967007	42,508	40,572	A>G	0.18	0.99	0.96	1.01	2.9E-01	*KITLG*	intronic			SKIN,SKIN,LNG	Pou5f1
rs4796052	17	26103034	42,509	40,577	G>A	0.20	0.99	0.96	1.01	2.9E-01	*NOS2, LOC645754*	intronic		FAT, LIV, ADRL		Mtf1,RBP-Jkappa,Sin3Ak-20
rs27438	5	131413255	42,508	40,573	G>A	0.22	0.99	0.96	1.01	2.9E-01	*CSF2, IL3*			BLD		
rs2796822	1	218586132	42,505	40,574	A>G	0.41	0.99	0.97	1.01	2.9E-01	*TGFB2*	intronic		BRN, MUS, BONE		LUN-1
rs4819553	22	17564907	42,502	40,574	A>G	0.18	1.01	0.99	1.04	2.9E-01	*IL17RA, CECR6, CECR7, RPL31P62, LOC100996342*		7 tissues	10 tissues	6 tissues	NF-E2
rs11768780	7	18926583	42,503	40,570	G>A	0.25	1.01	0.99	1.04	2.9E-01	*HDAC9*	intronic			SKIN	7 altered motifs
rs1178356	7	18189670	42,502	40,573	G>A	0.30	1.01	0.99	1.03	2.9E-01	*HDAC9, MIR1302-6*	intronic				5 altered motifs
rs12699981	7	18823893	42,501	40,570	A>G	0.16	1.01	0.99	1.04	2.9E-01	*HDAC9*	intronic				
rs12540265	7	18830464	42,494	40,570	A>G	0.16	1.01	0.99	1.04	2.9E-01	*HDAC9*	intronic				Pbx-1
rs4054760	3	3151668	42,487	40,562	G>A	0.28	0.99	0.97	1.01	2.9E-01	*IL5RA, TRNT1, CRBN*	5'-UTR	6 tissues	8 tissues	ESDR,BLD,BLD	NRSF,Rad21,TATA
rs17203439	3	121795506	42,488	40,569	A>C	0.06	0.98	0.94	1.02	2.9E-01	*CD86*	intronic	BLD	9 tissues	5 tissues	
rs334358	9	101910613	42,426	40,488	C>A	0.19	1.01	0.99	1.04	2.9E-01	*TGFBR1*	intronic		BLD		Evi-1,HNF4
rs2387356	13	28670595	42,477	40,501	A>G	0.06	0.98	0.94	1.02	2.9E-01	*FLT3, PAN3, CHCHD2P8, PAN3-AS1, LOC100420919*	intronic	BLD	BLD	4 tissues	Foxm1,SIX5,Znf143
rs72932376	2	204579339	42,507	40,574	T>A	0.08	1.02	0.98	1.06	2.9E-01	*CD28, KRT18P39, LOC100287498*	intronic	BLD	6 tissues		GATA,RFX5
rs6470574	8	128771572	42,494	40,554	G>A	0.30	0.99	0.97	1.01	2.9E-01	*MYC, MIR1204*		BLD	9 tissues	LNG,SKIN	NRSF,Znf143
rs11165489	1	92248274	42,508	40,577	G>A	0.09	1.02	0.98	1.06	2.9E-01	*TGFBR3*	intronic		19 tissues		5 altered motifs
rs8072785	17	40451643	42,322	40,402	G>A	0.10	1.02	0.98	1.05	2.9E-01	*STAT3, STAT5A, STAT5B*	intronic		MUS	4 tissues	
rs8064189	16	85951796	42,504	40,577	A>C	0.25	1.01	0.99	1.04	2.9E-01	*IRF8*	intronic		BLD, KID	4 tissues	AFP1,GR
rs11766273	7	22775663	42,484	40,519	G>A	0.08	0.98	0.95	1.02	2.9E-01	*IL6, RPS26P32, LOC541472*				IPSC	EBF,Foxd1
rs7737000	5	35871273	42,507	40,575	G>A	0.14	1.02	0.99	1.05	2.9E-01	*IL7R, CAPSL*					
rs17350515	7	18941624	42,506	40,576	A>G	0.38	1.01	0.99	1.03	2.9E-01	*HDAC9, NPM1P13*	intronic		7 tissues	MUS,MUS,MUS	HMG-IY,HNF1
rs945128	6	43710756	42,500	40,565	A>G	0.19	1.01	0.99	1.04	2.9E-01	*VEGFA, LOC100132242*			9 tissues		CDP,Hoxa5,PEBP
rs73963407	18	60004259	42,510	40,577	A>G	0.08	0.98	0.95	1.02	2.9E-01	*TNFRSF11A, KIAA1468*	intronic		5 tissues		5 altered motifs
rs1178178	7	18799718	42,499	40,569	G>A	0.17	1.01	0.99	1.04	2.9E-01	*HDAC9*	intronic				Maf,Zec
rs2087299	1	92203803	42,501	40,575	G>A	0.46	1.01	0.99	1.03	2.9E-01	*TGFBR3*	intronic		7 tissues		Hoxa5,Zfp691
rs4147359	10	6108439	42,494	40,565	G>A	0.35	0.99	0.97	1.01	3.0E-01	*IL2RA, RBM17, RPL32P23*		BLD	9 tissues	ESDR,LNG,BLD	Cphx,DEC
rs6503697	17	40501579	42,502	40,575	T>A	0.28	1.01	0.99	1.03	3.0E-01	*STAT3, STAT5A*	intronic		11 tissues		9 altered motifs
rs11567754	5	35872190	42,501	40,575	T>A	0.14	1.02	0.99	1.05	3.0E-01	*IL7R, CAPSL*	intronic		BLD		Irf,Pou2f2,Sox
rs7709212	5	158764177	42,468	40,543	A>G	0.33	1.01	0.99	1.03	3.0E-01	*IL12B, LOC285626, RNU4ATAC2P*	intronic		10 tissues	4 tissues	ERalpha-a
rs16985442	20	44671875	42,510	40,577	C>G	0.05	1.02	0.98	1.07	3.0E-01	*MMP9, SLC12A5, NCOA5, LOC100128028*	missense			BLD	Rad21
rs1178179	7	18799782	42,507	40,574	A>G	0.22	1.01	0.99	1.04	3.0E-01	*HDAC9*	intronic			4 tissues	CDP,Cart1
rs1295685	5	131996445	42,475	40,548	G>A	0.21	0.99	0.96	1.01	3.0E-01	*IL4, IL13, RAD50, KIF3A*	3'-UTR		5 tissues		AP-1,SMC3,Znf143

SNP	Chr	Position[a]	N Cases	N Controls	Allele	MAF	OR[b]	95%CI		*p* -value	Gene annotation	dbSNP functional annotation	Promoter histone marks	Enhancer histone marks	DNAse	Motifs changed
rs2290611	3	3116438	42,502	40,568	G>A	0.24	1.01	0.99	1.04	3.0E-01	*IL5RA, CNTN4*	intronic		BLD	BLD	
rs2799537	1	92293412	42,494	40,560	A>G	0.45	0.99	0.97	1.01	3.0E-01	*TGFBR3*	intronic	5 tissues	16 tissues	6 tissues	AP-1,CTCF
rs334356	9	101909372	42,502	40,571	G>A	0.19	1.01	0.99	1.04	3.0E-01	*TGFBR1*	intronic		BLD, VAS		6 altered motifs
rs10905578	10	6031880	42,339	40,360	G>A	0.26	1.01	0.99	1.04	3.0E-01	*IL2RA, IL15RA*				LNG,MUS,SKIN	8 altered motifs
rs6458350	6	43789616	42,505	40,575	A>G	0.32	1.01	0.99	1.03	3.0E-01	*VEGFA*			GI, PLCNT		6 altered motifs
rs2029314	3	30692219	42,508	40,575	G>A	0.14	1.02	0.99	1.05	3.0E-01	*TGFBR2*	intronic		6 tissues	ADRL	CTCF,NRSF
rs2519746	7	18817369	42,490	40,561	A>G	0.25	1.01	0.99	1.04	3.0E-01	*HDAC9*	intronic				ERalpha-a,NR4A,RORalpha1
rs2229113	11	117869670	42,497	40,545	G>A	0.33	0.99	0.97	1.01	3.0E-01	*IL10RA, TMPRSS4-AS1*	missense	ESDR	4 tissues		4 altered motifs
rs2440598	7	18843093	42,504	40,569	C>A	0.21	1.01	0.99	1.04	3.0E-01	*HDAC9*	intronic				Bcl6b,CIZ,NF-kappaB
rs6963748	7	18666217	42,507	40,574	G>A	0.25	0.99	0.97	1.01	3.0E-01	*HDAC9, LOC100419901*	intronic	FAT	STRM, FAT		CDP
rs6074032	20	44763320	42,485	40,564	C>A	0.40	1.01	0.99	1.03	3.0E-01	*CD40, NCOA5, CDH22, RPL13P2*			BRN		Klf7,UF1H3BETA
rs12722496	10	6096667	42,504	40,574	A>G	0.10	1.02	0.98	1.05	3.0E-01	*IL2RA, RBM17, RPL32P23*	intronic	LIV	7 tissues		Znf143
rs1129055	3	121838319	42,508	40,577	G>A	0.28	0.99	0.97	1.01	3.0E-01	*CD86*	missense				HNF4,Myc,p300
rs2872753	17	26095562	42,182	40,237	A>G	0.42	1.01	0.99	1.03	3.0E-01	*NOS2, LOC645754*	intronic				Pax-2,Pbx3
rs17131553	1	92226887	42,510	40,572	G>A	0.08	1.02	0.98	1.06	3.0E-01	*TGFBR3*	intronic		14 tissues	10 tissues	Maf,SETDB1,STAT
rs334357	9	101909753	42,505	40,575	T>A	0.19	1.01	0.99	1.04	3.0E-01	*TGFBR1*	intronic		BLD		4 altered motifs
rs284873	1	92170401	42,510	40,576	A>G	0.08	1.02	0.98	1.06	3.0E-01	*TGFBR3*	intronic		5 tissues		7 altered motifs
rs17293761	11	102659233	42,470	40,556	G>A	0.08	0.98	0.95	1.02	3.0E-01	*MMP1, MMP3, MMP10, CSNK1A1P2, WTAPP1, LOC100421658*	intronic		SKIN, GI	7 tissues	
rs2129975	1	92282080	42,504	40,573	C>A	0.45	0.99	0.97	1.01	3.0E-01	*TGFBR3*	intronic		12 tissues	OVRY	DMRT1
rs6790706	3	30691473	42,483	40,549	A>G	0.14	1.02	0.99	1.05	3.0E-01	*TGFBR2*	intronic	BLD	13 tissues	ESDR,BLD,LNG	4 altered motifs
rs4650272	1	92168649	42,508	40,571	A>T	0.20	0.99	0.96	1.01	3.0E-01	*TGFBR3*	intronic		FAT, SKIN	MUS	8 altered motifs
rs4658275	1	92285359	42,507	40,577	A>G	0.45	0.99	0.97	1.01	3.0E-01	*TGFBR3*	intronic	MUS	19 tissues		15 altered motifs
rs11070241	15	40284802	42,509	40,577	A>G	0.22	1.01	0.99	1.04	3.0E-01	*SRP14, EIF2AK4, H3F3AP1, SRP14-AS1*	intronic			BRN	GATA,Myc,Znf143
rs1367610	3	30697330	42,496	40,567	G>C	0.14	1.02	0.99	1.05	3.0E-01	*TGFBR2*	intronic	BLD	18 tissues	4 tissues	GR,Gfi1,HNF1
rs957958	7	18877616	42,498	40,568	A>G	0.39	1.01	0.99	1.03	3.0E-01	*HDAC9*	intronic				Zec
rs11776228	8	79686236	42,509	40,576	G>A	0.06	0.98	0.94	1.02	3.0E-01	*IL7, PRKRIRP7*	intronic				TEF-1
rs10260111	7	22816495	42,505	40,576	G>A	0.29	1.01	0.99	1.03	3.0E-01	*IL6, TOMM7, RPS26P32, LOC541472*				IPSC	4 altered motifs
rs9894367	17	76237462	42,500	40,553	A>C	0.08	1.02	0.98	1.06	3.0E-01	*BIRC5, AFMID, TMEM235, THA1P, LOC100996291*		LIV	5 tissues	4 tissues	4 altered motifs
rs1546646	2	191828596	42,227	40,229	G>A	0.38	1.01	0.99	1.03	3.0E-01	*GLS, STAT1, LOC100420571*	3'-UTR		STRM, HRT, PANC		Homez
rs1870703	3	30691078	42,500	40,560	A>G	0.14	1.02	0.99	1.05	3.0E-01	*TGFBR2*	intronic	BLD	14 tissues	ESDR,BLD,BLD	7 altered motifs
rs11119564	1	206966159	42,507	40,576	G>A	0.31	0.99	0.97	1.01	3.1E-01	*IL10, IL19*			6 tissues		Nanog,VDR
rs1178172	7	18794541	42,485	40,567	A>G	0.22	1.01	0.99	1.04	3.1E-01	*HDAC9*	intronic	6 tissues	BRST, FAT, LNG	12 tissues	CEBPG,HEY1,Pax-1
rs1468063	10	90775291	42,497	40,559	G>A	0.10	1.02	0.98	1.05	3.1E-01	*ACTA2, FAS, FAS-AS1, MIR4679-1, MIR4679-2*	3'-UTR		BLD	SKIN	Nkx2
rs11466522	3	30729246	42,469	40,491	G>A	0.11	1.02	0.99	1.05	3.1E-01	*TGFBR2, GADL1*	intronic		14 tissues		Hic1,Ik-1,ZBTB7A
rs10053847	5	35878140	42,508	40,576	G>A	0.14	1.02	0.99	1.05	3.1E-01	*IL7R, CAPSL*					Pax-4
rs2239926	7	18636561	42,483	40,555	G>C	0.27	0.99	0.97	1.01	3.1E-01	*HDAC9, LOC100419901*	intronic		ESC, HRT	IPSC	Dobox4
rs1178171	7	18794346	42,505	40,573	A>T	0.22	1.01	0.99	1.04	3.1E-01	*HDAC9*	intronic	6 tissues	LNG, BRST, FAT	6 tissues	AP-2
rs3759000	11	118135449	42,505	40,576	A>G	0.26	0.99	0.97	1.01	3.1E-01	*CD3E, MPZL2, AMICA1, MPZL3*		11 tissues	5 tissues	12 tissues	SIX5,Spz1
rs1013186	9	101884337	42,502	40,574	G>A	0.19	1.01	0.99	1.04	3.1E-01	*TGFBR1*	intronic			GI	Evi-1
rs284201	1	92240238	42,484	40,538	G>A	0.28	1.01	0.99	1.03	3.1E-01	*TGFBR3*	intronic		9 tissues	10 tissues	Pdx1
rs284870	1	92179383	42,489	40,541	C>A	0.08	1.02	0.98	1.06	3.1E-01	*TGFBR3*	intronic	4 tissues	15 tissues	8 tissues	4 altered motifs
rs6461390	7	18910232	42,464	40,532	A>G	0.39	1.01	0.99	1.03	3.1E-01	*HDAC9*	intronic				9 altered motifs
rs10988714	9	101884880	42,506	40,575	G>A	0.19	1.01	0.99	1.04	3.1E-01	*TGFBR1*	intronic				5 altered motifs
rs11878235	19	7976698	42,470	40,554	A>G	0.42	0.99	0.97	1.01	3.1E-01	*ELAVL1, MAP2K7, SNAPC2, TIMM44, LRRC8E, FLJ22184, EVI5L, LOC388499, CTXN1, TGFBR3L*	intronic		SKIN, LIV	4 tissues	
rs1178168	7	18792822	42,505	40,573	A>G	0.18	1.01	0.99	1.04	3.1E-01	*HDAC9*	intronic		5 tissues		7 altered motifs
rs1940935	11	102755015	42,505	40,576	A>G	0.33	1.01	0.99	1.03	3.1E-01	*MMP3, MMP12, WTAPP1, LOC100288111*					
rs2270501	16	85932988	42,504	40,574	G>A	0.18	0.99	0.96	1.01	3.1E-01	*IRF8*	intronic	20 tissues	4 tissues	14 tissues	NF-AT1
rs12680047	8	128758861	42,492	40,528	A>G	0.38	0.99	0.97	1.01	3.1E-01	*MYC, MIR1204*			5 tissues	OVRY,PANC,LNG	Myc,SREBP
rs592456	10	6435374	42,509	40,576	A>G	0.10	1.02	0.98	1.05	3.1E-01	*PRKCQ, DKFZp667F0711*			BLD		ATF3,Maf,Nrf-2
rs6602398	10	6082953	42,464	40,528	C>A	0.32	0.99	0.97	1.01	3.1E-01	*IL2RA, RBM17, RPL32P23*	intronic		BLD		Mrg,Myb,Tgif1
rs17139840	7	18800603	42,507	40,576	A>G	0.18	1.01	0.99	1.04	3.1E-01	*HDAC9*	intronic		ESDR, STRM, BRN	KID	Rad21,SMC3
rs2853694	5	158749088	42,506	40,577	C>A	0.49	0.99	0.97	1.01	3.1E-01	*IL12B, UBLCP1, LOC285626, RNU4ATAC2P*	intronic				HDAC2,Obox6
rs12220818	10	6517476	42,033	40,173	C>A	0.14	0.99	0.96	1.01	3.1E-01	*PRKCQ*	intronic	BLD	6 tissues		T3R
rs3792424	3	3117700	42,510	40,577	A>G	0.07	1.02	0.98	1.06	3.1E-01	*IL5RA, CNTN4*	intronic				
rs2254514	4	142640538	42,499	40,563	G>A	0.28	0.99	0.97	1.01	3.1E-01	*IL15*	5'-UTR		BLD		
rs16882	16	85935573	42,509	40,576	A>G	0.18	1.01	0.99	1.04	3.1E-01	*IRF8*	intronic	6 tissues	7 tissues	9 tissues	E2A,Myf,NF-kappaB
rs2386841	10	6057732	42,508	40,574	C>A	0.17	1.01	0.99	1.04	3.1E-01	*IL2RA, IL15RA*	intronic		BLD, ADRL		4 altered motifs

SNP	Chr	Position[a]	N Cases	N Controls	Allele	MAF	OR[b]	95%CI		*p* -value	Gene annotation	dbSNP functional annotation	Promoter histone marks	Enhancer histone marks	DNAse	Motifs changed
rs8064645	17	76253595	42,453	40,547	A>G	0.17	0.99	0.96	1.01	3.1E-01	*BIRC5, AFMID, TMEM235, THA1P, LOC100996291*		BLD	14 tissues	9 tissues	5 altered motifs
rs16991114	20	44767100	42,506	40,570	A>G	0.10	0.98	0.95	1.02	3.1E-01	*CD40, NCOA5, CDH22, RPL13P2*			5 tissues		5 altered motifs
rs2300095	1	11265717	42,502	40,573	G>A	0.29	1.01	0.99	1.03	3.1E-01	*MTOR, ANGPTL7, RPL39P6*	intronic				Ik-2
rs4246868	9	21478318	42,504	40,575	G>A	0.48	0.99	0.97	1.01	3.1E-01	*IFNA1, IFNWP19, IFNE, MIR31, MIR31HG*	intronic				4 altered motifs
rs17886680	3	3144909	42,478	40,529	G>A	0.11	1.02	0.98	1.05	3.1E-01	*IL5RA, TRNT1, CRBN, CNTN4*	intronic				
rs17505589	8	79689635	42,500	40,564	G>A	0.10	1.02	0.98	1.05	3.1E-01	*IL7, PRKRIRP7*	intronic				Ets,HNF4,MIZF
rs9268832	6	32427789	42,499	40,568	G>A	0.41	0.99	0.97	1.01	3.1E-01	*HLA-DRA, HLA-DRB9*		16 tissues	BLD, GI	LNG,BLD	
rs10226243	7	18968003	42,504	40,576	C>A	0.19	1.01	0.99	1.04	3.1E-01	*HDAC9, NPM1P13*	intronic				Foxo,Sox,p300
rs4655688	1	67657982	40,657	37,922	A>G	0.13	0.98	0.95	1.01	3.1E-01	*IL23R*	intronic	BLD	5 tissues	ADRL,BLD	Irf
rs2190278	7	18931305	42,507	40,574	A>G	0.24	1.01	0.99	1.04	3.1E-01	*HDAC9*	intronic		5 tissues		7 altered motifs
rs1977426	10	6573467	42,472	40,517	C>A	0.12	1.02	0.99	1.05	3.1E-01	*PRKCQ, PRKCQ-AS1*	intronic	BLD	ESC, BLD, THYM		
rs11009360	10	33655450	42,380	40,429	A>G	0.14	1.01	0.99	1.04	3.1E-01	*NRP1*		MUS	6 tissues	5 tissues	HMG-IY,Sox
rs9269043	6	32438598	42,490	40,565	A>G	0.27	0.99	0.97	1.01	3.1E-01	*HLA-DRA, HLA-DRB5, HLA-DRB9*					
rs982098	10	44828212	42,499	40,568	A>C	0.45	1.01	0.99	1.03	3.1E-01	*CXCL12, LOC100130539*			MUS	GI	Smad3,Smad
rs11990466	8	79700362	42,508	40,577	G>A	0.06	0.98	0.94	1.02	3.1E-01	*IL7, PRKRIRP7*	intronic				4 altered motifs
rs2268626	14	76444767	42,504	40,572	A>G	0.20	0.99	0.96	1.01	3.1E-01	*TGFB3, TTLL5, IFT43, LOC100506576*	intronic	PLCNT, MUS	18 tissues	4 tissues	5 altered motifs
rs10912561	1	173164565	42,495	40,571	G>A	0.24	1.01	0.99	1.04	3.1E-01	*TNFSF4, LOC100506023*	intronic				TCF4
rs12722563	10	6069561	42,498	40,566	G>A	0.11	1.02	0.98	1.05	3.1E-01	*IL2RA, IL15RA, RPL32P23*	intronic		BLD		15 altered motifs
rs17349622	7	18835921	42,431	40,530	A>C	0.25	1.01	0.99	1.04	3.1E-01	*HDAC9*	intronic		ESC		PLZF,SIX5
rs7622183	3	3152661	42,485	40,560	A>G	0.48	1.01	0.99	1.03	3.1E-01	*IL5RA, TRNT1, CRBN*		BLD	10 tissues	ESC,ESDR	GATA,Pou2f2
rs3806681	3	3152235	42,497	40,571	G>C	0.25	1.01	0.99	1.04	3.1E-01	*IL5RA, TRNT1, CRBN*		6 tissues	12 tissues	6 tissues	
rs3093006	6	167551452	42,509	40,573	G>A	0.12	1.02	0.99	1.05	3.1E-01	*CCR6, GPR31, TCP10L2*	3'-UTR		BLD	LNG	EWSR1-FLI1
rs11775264	8	79685194	42,497	40,566	G>A	0.06	0.98	0.94	1.02	3.1E-01	*IL7, PRKRIRP7*	intronic		CRVX	BRST	5 altered motifs
rs10136000	14	105253581	42,493	40,568	G>A	0.28	1.01	0.99	1.03	3.1E-01	*AKT1, SIVA1, RPS2P4, ADSSL1, LINC00638, ZBTB42, RPS26P49*	intronic			BLD	AP-1,NF-kappaB,TATA
rs929196	7	18824742	42,483	40,562	C>G	0.16	1.01	0.99	1.04	3.2E-01	*HDAC9*	intronic				
rs943072	6	43795968	42,509	40,572	A>C	0.09	0.98	0.95	1.02	3.2E-01	*VEGFA*			5 tissues		5 altered motifs
rs9268924	6	32432858	42,493	40,566	G>A	0.27	0.99	0.97	1.01	3.2E-01	*HLA-DRA, HLA-DRB9*					7 altered motifs
rs1178114	7	18746435	42,503	40,573	C>A	0.28	1.01	0.99	1.03	3.2E-01	*HDAC9*	intronic		BLD, HRT, GI	KID	Hsf,Irf,Nr2e3
rs527832	11	102701884	42,507	40,576	G>A	0.11	1.02	0.98	1.05	3.2E-01	*MMP1, MMP3, MMP12, CSNK1A1P2, WTAPP1, LOC100288111*	intronic		FAT, SKIN, GI	KID	4 altered motifs
rs9797244	17	26097131	42,506	40,576	A>G	0.19	0.99	0.96	1.01	3.2E-01	*NOS2, LOC645754*	intronic				Foxp1
rs1998843	1	198632356	42,497	40,561	G>A	0.34	1.01	0.99	1.03	3.2E-01	*PTPRC, PEBP1P3*	intronic	BLD	6 tissues	BLD	4 altered motifs
rs2512147	11	117872734	42,491	40,549	G>A	0.33	0.99	0.97	1.01	3.2E-01	*IL10RA, TMPRSS4-AS1*		BLD	9 tissues	8 tissues	ATF3
rs10229723	7	18968547	41,851	39,830	G>A	0.19	1.01	0.99	1.04	3.2E-01	*HDAC9, NPM1P13*	intronic				5 altered motifs
rs17045386	2	54140575	42,507	40,572	G>C	0.08	0.98	0.94	1.02	3.2E-01	*PSME4*	intronic		BLD, VAS		CAC-binding-protein,Egr-1,Pou2f2
rs1296210	1	198741944	42,506	40,575	C>G	0.46	1.01	0.99	1.03	3.2E-01	*PTPRC, LOC100131234*		BLD	4 tissues	ADRL,MUS	4 altered motifs
rs7515374	1	206908023	42,496	40,574	A>C	0.41	1.01	0.99	1.03	3.2E-01	*IL10, MAPKAPK2, RPS14P1*			8 tissues	31 tissues	EBF,TAL1
rs1178116	7	18750202	42,502	40,568	G>A	0.19	1.01	0.99	1.04	3.2E-01	*HDAC9*	intronic				SRF
rs6677319	1	67865150	42,505	40,576	A>G	0.41	1.01	0.99	1.03	3.2E-01	*IL12RB2, SERBP1*		ESC	5 tissues	ESC,IPSC	PLZF
rs17659401	3	3112865	42,503	40,576	G>A	0.24	1.01	0.99	1.04	3.2E-01	*IL5RA, CNTN4*	intronic		ESDR		YY1
rs17884684	1	92223221	42,502	40,554	C>A	0.07	1.02	0.98	1.06	3.2E-01	*TGFBR3*	intronic		7 tissues		25 altered motifs
rs11165354	1	92194322	42,503	40,572	A>C	0.38	0.99	0.97	1.01	3.2E-01	*TGFBR3*	intronic		7 tissues	MUS,MUS,BLD	Lhx8,Maf,Pou2f2
rs2236757	21	34624917	42,504	40,573	G>A	0.29	1.01	0.99	1.03	3.2E-01	*IFNAR2, IL10RB, IL10RB-AS1*	intronic		BRST, MUS		TFII-I
rs1178098	7	18734921	42,501	40,573	G>A	0.29	1.01	0.99	1.03	3.2E-01	*HDAC9*	intronic		ESDR		5 altered motifs
rs10739779	9	101882199	42,498	40,569	C>A	0.19	1.01	0.99	1.04	3.2E-01	*COL15A1, TGFBR1*	intronic			BLD	4 altered motifs
rs472317	10	6532178	42,505	40,571	G>A	0.39	1.01	0.99	1.03	3.2E-01	*PRKCQ*	intronic		BLD, THYM		Myf,STAT,WT1
rs3773643	3	30710243	42,507	40,576	A>G	0.20	0.99	0.96	1.01	3.2E-01	*TGFBR2*	intronic	HRT	4 tissues		Pou1f1,Pou3f3
rs12132114	1	92293721	42,508	40,572	G>A	0.21	0.99	0.96	1.01	3.2E-01	*TGFBR3*	intronic	5 tissues	13 tissues	6 tissues	CDP
rs9818228	3	121850589	42,500	40,574	A>C	0.45	0.99	0.97	1.01	3.2E-01	*CD86*					5 altered motifs
rs1147879	10	44850650	42,506	40,575	A>G	0.49	1.01	0.99	1.03	3.2E-01	*CXCL12*			5 tissues		4 altered motifs
rs17834593	14	62127263	42,508	40,576	A>G	0.13	0.99	0.96	1.01	3.2E-01	*HIF1A, FLJ22447*		BLD	13 tissues	7 tissues	5 altered motifs
rs1074078	1	11326788	42,499	40,563	G>A	0.33	1.01	0.99	1.03	3.2E-01	*MTOR, UBIAD1, RPL39P6, UBE2V2P3*				BLD	
rs9579165	13	28724311	42,509	40,575	A>G	0.06	1.02	0.98	1.06	3.3E-01	*FLT3, PAN3, CHCHD2P8, PAN3-AS1*	intronic		BLD		Hoxb9,VDR
rs11581605	1	218524304	42,183	40,192	A>G	0.23	0.99	0.96	1.01	3.3E-01	*TGFB2, RRP15, RPS26P17, LOC728463*	intronic	13 tissues	7 tissues	12 tissues	Mef2
rs1805117	1	92148839	42,508	40,573	A>G	0.18	0.99	0.96	1.01	3.3E-01	*TGFBR3, HSP90B3P*	3'-UTR		13 tissues		p300
rs2389941	7	18392320	42,510	40,577	G>A	0.11	0.98	0.95	1.02	3.3E-01	*HDAC9*	intronic	ESC	16 tissues	18 tissues	5 altered motifs
rs34410111	10	6151262	42,507	40,577	G>A	0.27	0.99	0.97	1.01	3.3E-01	*IL2RA, PFKFB3, RBM17, RPL32P23, MIR3155A, MIR3155B*	intronic	BLD	8 tissues	15 tissues	
rs4297265	1	67852335	42,505	40,572	A>G	0.41	1.01	0.99	1.03	3.3E-01	*IL12RB2, SERBP1*	synonymous				
rs305084	16	85934168	42,502	40,569	A>G	0.09	1.02	0.98	1.05	3.3E-01	*IRF8*	intronic	10 tissues	BLD, GI	5 tissues	DMRT5

SNP	Chr	Position[a]	N Cases	N Controls	Allele	MAF	OR[b]	95%CI		*p* -value	Gene annotation	dbSNP functional annotation	Promoter histone marks	Enhancer histone marks	DNAse	Motifs changed
rs9610417	22	22199970	42,484	40,540	G>A	0.23	1.01	0.99	1.04	3.3E-01	*MAPK1*	intronic		BLD, SKIN, MUS	4 tissues	
rs11764116	7	18800413	42,504	40,573	C>A	0.22	1.01	0.99	1.04	3.3E-01	*HDAC9*	intronic		ESDR, STRM, BRN		5 altered motifs
rs2232360	1	207040659	42,510	40,576	A>G	0.23	0.99	0.97	1.01	3.3E-01	*FAIM3, IL24, IL19, IL20, RPL13AP8*	intronic		BRST, SKIN, CRVX	SKIN	
rs3153	21	34609505	42,506	40,574	G>A	0.29	1.01	0.99	1.03	3.3E-01	*IFNAR2, IL10RB, IL10RB-AS1*	intronic	BLD	9 tissues	BLD	7 altered motifs
rs3093001	6	167554185	42,498	40,571	A>C	0.49	1.01	0.99	1.03	3.3E-01	*CCR6, GPR31, TCP10L2*			7 tissues	BLD,GI,GI	6 altered motifs
rs17350341	7	18928240	42,444	40,542	C>A	0.24	1.01	0.99	1.04	3.3E-01	*HDAC9*	intronic				4 altered motifs
rs3785898	17	40515120	42,502	40,576	C>A	0.28	1.01	0.99	1.03	3.3E-01	*STAT3, PTRF*	intronic	MUS	21 tissues	MUS	Myc,p300
rs27349	5	131413722	42,491	40,572	C>A	0.19	0.99	0.96	1.01	3.3E-01	*CSF2, IL3*			SKIN		ERalpha-a,NERF1a,THAP1
rs7178419	15	40284523	42,504	40,571	A>G	0.22	1.01	0.99	1.04	3.3E-01	*SRP14, EIF2AK4, H3F3AP1, SRP14-AS1*	intronic				4 altered motifs
rs10783003	1	92195746	42,419	40,518	A>G	0.38	0.99	0.97	1.01	3.3E-01	*TGFBR3*	intronic			4 tissues	Rad21,SP1
rs16824035	2	191837634	42,510	40,576	G>A	0.16	1.01	0.99	1.04	3.3E-01	*GLS, STAT1, LOC100420571*	intronic		5 tissues	SKIN	Zfp410
rs790815	10	6530052	42,496	40,570	A>G	0.49	1.01	0.99	1.03	3.3E-01	*PRKCQ*	intronic		BLD	BLD	
rs2028016	7	18834799	42,500	40,572	A>G	0.17	1.01	0.99	1.04	3.3E-01	*HDAC9*	intronic		GI		7 altered motifs
rs1805115	1	92149139	42,506	40,575	G>A	0.18	0.99	0.96	1.01	3.3E-01	*TGFBR3, HSP90B3P*	3'-UTR		14 tissues	ESC,SKIN	4 altered motifs
rs2270614	1	67856021	42,509	40,577	G>A	0.41	1.01	0.99	1.03	3.3E-01	*IL12RB2, SERBP1*	intronic	IPSC, BRN, BONE	10 tissues	8 tissues	4 altered motifs
rs996999	11	102667063	40,879	38,400	G>A	0.21	0.99	0.96	1.01	3.3E-01	*MMP1, MMP3, MMP10, CSNK1A1P2, WTAPP1, LOC100421658*	intronic	SKIN			9 altered motifs
rs1615309	7	18619934	42,474	40,534	G>A	0.46	0.99	0.97	1.01	3.3E-01	*HDAC9, LOC100419901*	intronic		BRN		5 altered motifs
rs9268977	6	32434939	42,508	40,575	A>G	0.27	0.99	0.97	1.01	3.3E-01	*HLA-DRA, HLA-DRB9*					
rs45531934	14	105244651	42,396	40,415	G>A	0.06	1.02	0.98	1.06	3.3E-01	*AKT1, SIVA1, ADSSL1, LINC00638, ZBTB42*	intronic		12 tissues	4 tissues	4 altered motifs
rs12722558	10	6070276	42,416	40,476	T>A	0.11	1.02	0.98	1.05	3.3E-01	*IL2RA, RPL32P23*	intronic		BLD	ESDR,MUS	GR,Rad21
rs6951144	7	18911789	42,488	40,549	C>G	0.38	1.01	0.99	1.03	3.3E-01	*HDAC9*	intronic				4 altered motifs
rs12700003	7	18905866	42,493	40,559	G>A	0.41	1.01	0.99	1.03	3.3E-01	*HDAC9*	intronic		10 tissues	HRT,SKIN	
rs13038175	20	44624097	42,460	40,490	G>A	0.13	1.02	0.98	1.05	3.3E-01	*MMP9, SLC12A5, ZNF335, PCIF1, FTLP1, LOC100128028*		LNG, CRVX	10 tissues	SKIN,CRVX	ERalpha-a
rs4721729	7	18970877	42,504	40,575	G>A	0.20	1.01	0.99	1.04	3.3E-01	*HDAC9, NPM1P13*	intronic				GR,HNF1,p300
rs77173997	20	44761541	42,507	40,574	G>A	0.11	0.98	0.95	1.02	3.3E-01	*CD40, NCOA5, CDH22, RPL13P2*					
rs4955189	3	30721939	42,438	40,491	G>A	0.39	1.01	0.99	1.03	3.3E-01	*TGFBR2, GADL1*	intronic		14 tissues		BRCA1,Nanog,Sox
rs764522	3	30646550	42,499	40,568	G>C	0.19	0.99	0.96	1.01	3.3E-01	*TGFBR2*		BLD, GI, LIV	15 tissues		AP-1,Bach2
rs1624397	7	18680661	42,485	40,559	G>C	0.25	0.99	0.97	1.01	3.3E-01	*HDAC9*	intronic		BRN		
rs11583394	1	206968893	42,509	40,577	A>G	0.23	0.99	0.97	1.01	3.3E-01	*IL10, IL19*			BLD, PANC	BLD,PANC	BDP1,TBX5
rs859652	1	172654377	42,496	40,563	G>A	0.35	1.01	0.99	1.03	3.3E-01	*FASLG*			BLD		CEBPA,Irf,PRDM1
rs17139571	7	18714254	42,466	40,527	A>C	0.06	1.02	0.98	1.07	3.3E-01	*HDAC9*	intronic				10 altered motifs
rs11778246	8	79705472	42,280	40,357	A>G	0.06	0.98	0.94	1.02	3.3E-01	*IL7, PRKRIRP7*	intronic	FAT, STRM	6 tissues	5 tissues	MZF1::1-4,Rad21
rs2191030	3	45910946	42,505	40,572	G>A	0.22	0.99	0.96	1.01	3.3E-01	*CCR9, SDHDP4, LZTFL1, FYCO1*			BLD, GI, THYM		Pax-6
rs17147986	10	6038478	42,508	40,577	C>A	0.17	1.01	0.99	1.04	3.3E-01	*IL2RA, IL15RA*			ESC, ESDR, IPSC		Eomes,Pax-5,Roaz
rs7950838	11	76363817	42,500	40,573	A>G	0.49	1.01	0.99	1.03	3.3E-01	*LRRC32, GUCY2EP*			KID, LNG, GI		Hlx1,Pax-4
rs4655707	1	67875414	42,508	40,574	G>A	0.41	1.01	0.99	1.03	3.3E-01	*IL12RB2, SERBP1*	3'-UTR		4 tissues		
rs2190274	7	18952826	42,466	40,527	C>A	0.14	1.01	0.99	1.04	3.4E-01	*HDAC9, NPM1P13*	intronic			4 tissues	CEBPB,Gfi1
rs3136614	10	6005674	42,510	40,577	A>G	0.22	0.99	0.96	1.01	3.4E-01	*IL2RA, IL15RA, FBXO18*	intronic		PANC		FXR,GR
rs4846476	1	218526228	42,495	40,567	G>C	0.23	0.99	0.97	1.01	3.4E-01	*TGFB2, RRP15, RPS26P17, LOC728463*	intronic		8 tissues		4 altered motifs
rs2661694	17	76221008	42,504	40,572	C>A	0.27	1.01	0.99	1.03	3.4E-01	*BIRC5, TK1, AFMID, TMEM235, THA1P, LOC100996291*	3'-UTR	STRM, BONE	7 tissues	4 tissues	Foxl1,Nkx3,Pou6f1
rs6602368	10	6062915	41,840	40,119	G>A	0.45	1.01	0.99	1.03	3.4E-01	*IL2RA, IL15RA*	intronic		BLD	5 tissues	4 altered motifs
rs1178156	7	18783170	42,395	40,426	G>A	0.21	1.01	0.99	1.04	3.4E-01	*HDAC9*	intronic			BRN,MUS	4 altered motifs
rs3773661	3	30728290	42,510	40,577	G>C	0.11	1.02	0.98	1.05	3.4E-01	*TGFBR2, GADL1*	intronic		9 tissues		4 altered motifs
rs17047703	1	218525588	42,495	40,575	C>A	0.23	0.99	0.97	1.01	3.4E-01	*TGFB2, RRP15, RPS26P17, LOC728463*	intronic	MUS	9 tissues		Ets,STAT,Sp100
rs6702254	1	206964952	42,508	40,577	C>A	0.31	0.99	0.97	1.01	3.4E-01	*IL10, IL19*			8 tissues	BLD,BLD	CTCF
rs6586161	10	90741259	42,502	40,570	A>T	0.14	1.01	0.99	1.04	3.4E-01	*ACTA2, FAS, ACTA2-AS1, FAS-AS1*	intronic		10 tissues	OVRY	Nkx2
rs3804798	3	3127975	42,496	40,572	T>A	0.38	1.01	0.99	1.03	3.4E-01	*IL5RA, TRNT1, CNTN4*	intronic		BLD		YY1
rs4721701	7	18236056	42,503	40,575	A>G	0.38	1.01	0.99	1.03	3.4E-01	*HDAC9*	intronic				Pou2f2,Zfp410
rs305083	16	85935878	42,433	40,546	A>G	0.18	1.01	0.99	1.04	3.4E-01	*IRF8*	intronic	6 tissues	13 tissues	6 tissues	4 altered motifs
rs56754533	11	118188276	42,494	40,559	G>A	0.31	1.01	0.99	1.03	3.4E-01	*CD3D, CD3E, CD3G, UBE4A*		BLD	7 tissues	BLD,THYM	HNF1,Znf143
rs12133463	1	92156208	42,507	40,574	G>A	0.19	0.99	0.96	1.01	3.4E-01	*TGFBR3, HSP90B3P*	intronic		5 tissues		5 altered motifs
rs11165390	1	92203668	42,475	40,555	A>G	0.35	1.01	0.99	1.03	3.4E-01	*TGFBR3*	intronic		10 tissues		5 altered motifs
rs4395860	8	128788985	42,504	40,575	G>A	0.34	1.01	0.99	1.03	3.4E-01	*MYC, MIR1204*			ADRL, VAS, BLD	SKIN,VAS,BLD	5 altered motifs
rs284876	1	92171267	42,492	40,561	G>A	0.50	1.01	0.99	1.03	3.4E-01	*TGFBR3*	intronic	BLD	5 tissues	BLD	4 altered motifs
rs11649549	16	85935797	42,504	40,568	A>G	0.18	1.01	0.99	1.04	3.4E-01	*IRF8*	intronic	6 tissues	11 tissues	9 tissues	7 altered motifs
rs2182410	10	6122669	42,501	40,566	G>A	0.40	1.01	0.99	1.03	3.4E-01	*IL2RA, RBM17, RPL32P23*			BLD		5 altered motifs
rs11571316	2	204731089	42,497	40,559	G>A	0.41	0.99	0.97	1.01	3.4E-01	*CTLA4*		BLD, GI	BLD, THYM		STAT
rs12066159	1	92204798	42,508	40,574	A>G	0.35	1.01	0.99	1.03	3.4E-01	*TGFBR3*	intronic		7 tissues		AP-1
rs56346849	22	22132985	42,508	40,577	C>A	0.08	0.98	0.95	1.02	3.4E-01	*MAPK1, YPEL1*	intronic		BLD		Zbtb3

SNP	Chr	Position[a]	N Cases	N Controls	Allele	MAF	OR[b]	95%CI		*p* -value	Gene annotation	dbSNP functional annotation	Promoter histone marks	Enhancer histone marks	DNAse	Motifs changed
rs8192920	14	25099980	42,498	40,571	C>A	0.25	0.99	0.97	1.01	3.4E-01	*GZMH, GZMB*			9 tissues	4 tissues	SP1
rs594025	11	69443823	42,496	40,561	A>G	0.45	0.99	0.97	1.01	3.4E-01	*CCND1, ORAOV1, LOC100996515*			FAT		6 altered motifs
rs805316	2	54133744	42,505	40,573	A>G	0.28	0.99	0.97	1.01	3.4E-01	*GPR75, PSME4, GPR75-ASB3*	synonymous				Foxa
rs10245874	7	18968883	42,501	40,567	A>G	0.20	1.01	0.99	1.04	3.4E-01	*HDAC9, NPM1P13*	intronic				9 altered motifs
rs12533274	7	18837143	42,506	40,573	T>A	0.17	1.01	0.99	1.04	3.5E-01	*HDAC9*	intronic				12 altered motifs
rs3024570	16	27357784	42,509	40,577	G>A	0.08	0.98	0.95	1.02	3.5E-01	*IL4R*	intronic		BLD, MUS	KID	RBP-Jkappa
rs3814993	7	18635277	42,505	40,572	A>G	0.25	0.99	0.97	1.01	3.5E-01	*HDAC9, LOC100419901*	intronic		GI		5 altered motifs
rs305071	16	85949271	42,505	40,576	G>A	0.13	1.01	0.98	1.05	3.5E-01	*IRF8*	intronic		9 tissues		STAT,YY1
rs3092946		135729603	42,502	40,570	T>A	0.11	0.98	0.95	1.02	3.5E-01	*CD40LG, ARHGEF6, LINC00892*		BLD	BLD	BLD	4 altered motifs
rs2512145	11	117863032	42,490	40,564	G>A	0.33	0.99	0.97	1.01	3.5E-01	*IL10RA, TMPRSS4-AS1*	intronic	GI, BLD	BLD, LIV, GI	BLD,BLD,BLD	HIF1::Arnt
rs2068017	2	54119571	42,488	40,541	C>A	0.13	0.99	0.96	1.02	3.5E-01	*GPR75, PSME4, GPR75-ASB3, MIR3682*	intronic				7 altered motifs
rs3773660	3	30727776	42,509	40,573	G>A	0.11	1.02	0.98	1.05	3.5E-01	*TGFBR2, GADL1*	intronic		9 tissues	BRN,LNG	4 altered motifs
rs2520343	7	18843808	42,393	40,475	C>A	0.31	1.01	0.99	1.03	3.5E-01	*HDAC9*	intronic		4 tissues		Pou2f2,STAT
rs6950979	7	18971873	42,233	40,304	G>A	0.21	1.01	0.99	1.04	3.5E-01	*HDAC9, NPM1P13*	intronic				20 altered motifs
rs6604050	1	92153491	42,498	40,564	C>G	0.39	0.99	0.97	1.01	3.5E-01	*TGFBR3, HSP90B3P*	intronic		7 tissues		21 altered motifs
rs11165532	1	92267636	42,508	40,577	G>A	0.46	0.99	0.97	1.01	3.5E-01	*TGFBR3*	intronic	FAT	6 tissues	MUS,OVRY,MUS	4 altered motifs
rs8177628	10	6019698	42,388	40,418	G>A	0.17	1.01	0.99	1.04	3.5E-01	*IL2RA, IL15RA, FBXO18*	intronic	24 tissues		44 tissues	ERalpha-a,Znf143
rs116358504	10	6502444	42,472	40,529	A>G	0.16	0.99	0.96	1.01	3.5E-01	*PRKCQ*	intronic		THYM		11 altered motifs
rs3096702	6	32192331	42,499	40,576	G>A	0.38	1.01	0.99	1.03	3.5E-01	*AGER, NOTCH4, PBX2, RNF5, AGPAT1, GPSM3*			7 tissues	BLD	TLX1::NFIC
rs2266967	22	22156789	42,494	40,570	A>C	0.50	1.01	0.99	1.03	3.5E-01	*MAPK1*	intronic		ESDR, FAT, SKIN		4 altered motifs
rs11891922	2	9649966	42,505	40,567	G>A	0.42	0.99	0.97	1.01	3.5E-01	*ADAM17, CPSF3, IAH1*	intronic		8 tissues		Fox,Myc,RREB-1
rs11906879	20	44591324	42,426	40,506	A>G	0.44	1.01	0.99	1.03	3.5E-01	*MMP9, ZNF335, PCIF1, FTLP1*	intronic		BLD, SKIN		BCL
rs2248814	17	26100321	42,501	40,574	G>A	0.40	1.01	0.99	1.03	3.5E-01	*NOS2, LOC645754*	intronic			LNG	AP-2,p300
rs334355	9	101909286	42,143	40,160	G>A	0.20	1.01	0.99	1.04	3.5E-01	*TGFBR1*	intronic		BLD, VAS		7 altered motifs
rs1841528	3	30700853	42,505	40,576	G>A	0.14	1.01	0.98	1.04	3.5E-01	*TGFBR2*	intronic	4 tissues	23 tissues	35 tissues	FAC1,Sox
rs17293823	11	102660407	42,505	40,575	G>A	0.15	0.99	0.96	1.01	3.5E-01	*MMP1, MMP3, MMP10, CSNK1A1P2, WTAPP1, LOC100421658*	intronic			SKIN,SKIN	6 altered motifs
rs3213460	11	102668882	42,508	40,576	G>A	0.15	0.99	0.96	1.01	3.5E-01	*MMP1, MMP3, MMP10, CSNK1A1P2, WTAPP1, LOC100421658*	5'-UTR	SKIN, VAS	11 tissues	19 tissues	Rad21
rs743562	5	131872383	42,502	40,574	G>A	0.44	0.99	0.97	1.01	3.5E-01	*IL5, IRF1, RAD50*					Ik-3,Irf,TCF12
rs2027567	1	218558588	42,505	40,576	A>G	0.24	0.99	0.97	1.01	3.5E-01	*TGFB2, RRP15, RPS26P17, LOC728463*	intronic		8 tissues		
rs25881	5	131411138	42,502	40,572	G>A	0.19	0.99	0.96	1.01	3.5E-01	*CSF2, IL3*	intronic	BLD, SKIN, BRST	14 tissues	BRST,MUS	8 altered motifs
rs1178164	7	18788589	42,502	40,573	A>G	0.22	1.01	0.99	1.04	3.5E-01	*HDAC9*	intronic				PLZF
rs10905656	10	6086093	42,500	40,574	C>A	0.42	0.99	0.97	1.01	3.5E-01	*IL2RA, RBM17, RPL32P23*	intronic				BRCA1,HEY1,Sox
rs3773658	3	30724436	42,508	40,575	A>G	0.11	1.01	0.98	1.05	3.5E-01	*TGFBR2, GADL1*	intronic	BRN	17 tissues	9 tissues	5 altered motifs
rs1633787	7	18674936	42,509	40,576	C>A	0.06	1.02	0.98	1.06	3.5E-01	*HDAC9*	intronic				Eomes,Pax-5,TBX5
rs2253316	1	92161515	42,498	40,566	A>T	0.11	1.01	0.98	1.05	3.5E-01	*TGFBR3*	intronic		ESDR, FAT		Bbx,Pou3f2,RAR
rs5019497	1	92265633	42,497	40,572	C>A	0.46	0.99	0.97	1.01	3.5E-01	*TGFBR3*	intronic		11 tissues	6 tissues	5 altered motifs
rs885334	1	206962416	42,506	40,577	A>G	0.31	0.99	0.97	1.01	3.5E-01	*IL10, IL19*			BLD		13 altered motifs
rs4983382	14	105189504	42,029	40,245	A>G	0.23	1.01	0.99	1.04	3.6E-01	*AKT1, SIVA1, INF2, ADSSL1, MIR4710, LOC100996409*		MUS, LIV	19 tissues	21 tissues	4 altered motifs
rs6951128	7	18728720	42,500	40,569	G>A	0.10	0.98	0.95	1.02	3.6E-01	*HDAC9*	intronic		ESDR, STRM, BLD		HNF1,Mef2
rs284182	1	92228645	42,495	40,562	A>G	0.17	0.99	0.96	1.01	3.6E-01	*TGFBR3*	intronic		13 tissues		Lhx3,Mef2,OTX
rs17516399	1	92322843	42,510	40,577	A>C	0.08	0.98	0.95	1.02	3.6E-01	*TGFBR3*	intronic		4 tissues		CEBPB,Pou1f1,p300
rs10486293	7	18635795	42,418	40,478	G>A	0.22	0.99	0.97	1.01	3.6E-01	*HDAC9, LOC100419901*	intronic				HNF1,HP1-site-factor
rs10783037	1	92281635	42,492	40,571	G>A	0.45	0.99	0.97	1.01	3.6E-01	*TGFBR3*	intronic			4 tissues	ZID
rs13410158	2	9648988	42,483	40,564	A>C	0.42	0.99	0.97	1.01	3.6E-01	*ADAM17, CPSF3, IAH1*	intronic	MUS	ESDR, STRM, HRT		
rs12167	3	33038183	42,497	40,556	A>G	0.28	1.01	0.99	1.03	3.6E-01	*CCR4, GLB1, SEC13P1*	3'-UTR		BLD, ADRL		HNF1,Sox
rs4432111	13	28590258	42,472	40,537	G>A	0.06	0.98	0.94	1.02	3.6E-01	*CDX2, FLT3, PRHOXNB*	intronic				4 altered motifs
rs4819964	22	17605824	42,194	40,176	A>G	0.15	0.99	0.96	1.02	3.6E-01	*IL17RA, CECR6, CECR5, CECR5-AS1, RPL31P62, LOC100996342*					Arid5b,Sox
rs632009	11	102738499	42,258	40,368	G>A	0.33	1.01	0.99	1.03	3.6E-01	*MMP3, MMP12, WTAPP1, LOC100288111*	intronic		MUS		AP-1,CCNT2
rs2285437	7	18681992	42,461	40,505	C>G	0.09	1.02	0.98	1.05	3.6E-01	*HDAC9*	intronic		SKIN, ADRL	MUS,MUS	Evi-1
rs6951745	7	18911957	42,490	40,571	G>A	0.37	1.01	0.99	1.03	3.6E-01	*HDAC9*	intronic				RORalpha1
rs10063294	5	35877505	42,457	40,548	A>G	0.46	0.99	0.97	1.01	3.6E-01	*IL7R, CAPSL*				CRVX	7 altered motifs
rs1178174	7	18797020	42,501	40,571	G>A	0.22	1.01	0.99	1.04	3.6E-01	*HDAC9*	intronic		5 tissues		
rs6461382	7	18718161	42,348	40,406	G>A	0.10	0.98	0.95	1.02	3.6E-01	*HDAC9*	intronic				4 altered motifs
rs17187428	8	128746344	42,479	40,565	G>A	0.06	1.02	0.98	1.06	3.6E-01	*MYC*		17 tissues	14 tissues	53 tissues	E2F
rs1178155	7	18782969	42,458	40,535	T>A	0.20	1.01	0.99	1.04	3.6E-01	*HDAC9*	intronic			BRN	4 altered motifs
rs41295105	10	6122674	42,463	40,553	A>C	0.19	1.01	0.99	1.04	3.6E-01	*IL2RA, RBM17, RPL32P23*			BLD		14 altered motifs
rs284178	1	92225773	42,483	40,506	A>G	0.17	0.99	0.96	1.01	3.6E-01	*TGFBR3*	intronic		9 tissues		p300

SNP	Chr	Position[a]	N Cases	N Controls	Allele	MAF	OR[b]	95%CI		*p* -value	Gene annotation	dbSNP functional annotation	Promoter histone marks	Enhancer histone marks	DNAse	Motifs changed
rs74655772	22	22164673	42,500	40,566	G>A	0.50	0.99	0.97	1.01	3.6E-01	*MAPK1*					
rs10912560	1	173164457	42,506	40,576	A>G	0.25	1.01	0.99	1.03	3.6E-01	*TNFSF4, LOC100506023*	intronic				DMRT1,DMRT4
rs17513917	1	92205197	42,489	40,565	G>A	0.35	1.01	0.99	1.03	3.6E-01	*TGFBR3*	intronic		BRST	SKIN	EBF
rs4427135	8	128802021	42,492	40,568	A>C	0.46	1.01	0.99	1.03	3.6E-01	*MYC, MIR1204*				IPSC	
rs8177667	10	6011623	42,501	40,570	C>A	0.19	1.01	0.99	1.04	3.6E-01	*IL2RA, IL15RA, FBXO18*	intronic		4 tissues	BLD	SP1
rs473191	15	40248716	42,481	40,504	G>A	0.41	1.01	0.99	1.03	3.6E-01	*GPR176, EIF2AK4, H3F3AP1, LOC100505534*	intronic				
rs1555890	1	92160886	42,509	40,577	A>G	0.11	1.01	0.98	1.05	3.6E-01	*TGFBR3*	intronic		FAT		HDAC2,PU.1,Pou5f1
rs6550007	3	30701801	42,497	40,575	G>A	0.14	1.01	0.98	1.04	3.6E-01	*TGFBR2*	intronic	BLD, MUS	15 tissues	KID	Foxp3,SIX5
rs12722584	10	6062937	42,510	40,576	G>A	0.07	0.98	0.94	1.02	3.6E-01	*IL2RA, IL15RA*	intronic		BLD	6 tissues	
rs6604056	1	92260586	42,485	40,564	T>A	0.46	0.99	0.97	1.01	3.7E-01	*TGFBR3*	intronic		12 tissues		4 altered motifs
rs12089918	1	92298223	42,506	40,574	C>G	0.22	0.99	0.97	1.01	3.7E-01	*TGFBR3*	intronic		4 tissues	ADRL	5 altered motifs
rs17590111	2	9627680	42,473	40,541	A>G	0.08	1.02	0.98	1.06	3.7E-01	*ADAM17, CPSF3, IAH1*	intronic				AP-1,SP2
rs6537064	4	142658406	41,050	39,211	A>G	0.49	1.01	0.99	1.03	3.7E-01	*IL15*		VAS			4 altered motifs
rs7810384	7	18844771	42,502	40,560	G>A	0.31	1.01	0.99	1.03	3.7E-01	*HDAC9*	intronic		HRT	IPSC,PANC	Irf,NF-E2,PRDM1
rs2412456	15	40290027	42,508	40,577	A>T	0.34	0.99	0.97	1.01	3.7E-01	*SRP14, EIF2AK4, H3F3AP1, SRP14-AS1*	intronic		4 tissues		BCL,Irf,TCF4
rs10106163	8	128786712	42,507	40,577	G>A	0.09	0.98	0.95	1.02	3.7E-01	*MYC, MIR1204*				IPSC,CRVX	HNF4
rs3020919	11	102713046	42,502	40,576	G>A	0.23	0.99	0.97	1.01	3.7E-01	*MMP1, MMP3, MMP12, CSNK1A1P2, WTAPP1, LOC100288111*	intronic				E4BP4
rs305082	16	85936978	42,506	40,576	A>G	0.18	1.01	0.99	1.04	3.7E-01	*IRF8*	intronic	BLD, THYM	6 tissues	6 tissues	Sox
rs876687	3	30725645	42,505	40,575	A>G	0.11	1.01	0.98	1.05	3.7E-01	*TGFBR2, GADL1*	intronic	FAT	18 tissues	PLCNT	NF-AT,Zfp691
rs212671	7	18741874	42,451	40,542	A>C	0.31	1.01	0.99	1.03	3.7E-01	*HDAC9*	intronic		ESDR	MUS	Egr-1,Pax-5
rs17026240	3	30725213	42,506	40,577	A>T	0.11	1.01	0.98	1.05	3.7E-01	*TGFBR2, GADL1*	intronic		16 tissues		Gfi1
rs11600510	11	102660097	42,506	40,571	G>A	0.15	0.99	0.96	1.02	3.7E-01	*MMP1, MMP3, MMP10, CSNK1A1P2, WTAPP1, LOC100421658*	intronic		SKIN	4 tissues	HLF
rs2512143	11	117860582	42,480	40,545	G>A	0.33	0.99	0.97	1.01	3.7E-01	*IL10RA, TMPRSS4-AS1*	intronic	6 tissues	6 tissues	14 tissues	8 altered motifs
rs2520360	7	18821750	42,495	40,570	A>G	0.17	1.01	0.99	1.04	3.7E-01	*HDAC9*	intronic		4 tissues	ADRL	Maf,Myc,Pou3f2
rs25883	5	131411932	42,456	40,529	G>A	0.22	0.99	0.97	1.01	3.7E-01	*CSF2, IL3*			BLD, LNG, LIV		14 altered motifs
rs3093007	6	167549775	42,499	40,571	A>G	0.19	1.01	0.99	1.04	3.7E-01	*CCR6, GPR31, TCP10L2*	synonymous		BLD, STRM, GI	BLD	Mef2
rs17350397	7	18931787	42,507	40,572	C>G	0.26	1.01	0.99	1.03	3.7E-01	*HDAC9*	intronic				Dobox4,Pou1f1
rs212666	7	18738911	42,505	40,575	G>A	0.28	1.01	0.99	1.03	3.7E-01	*HDAC9*	intronic		GI		4 altered motifs
rs731465	3	30736169	42,505	40,574	C>G	0.33	1.01	0.99	1.03	3.7E-01	*TGFBR2, GADL1*			6 tissues		
rs11656102	17	76243058	42,496	40,568	G>A	0.38	0.99	0.97	1.01	3.7E-01	*BIRC5, AFMID, TMEM235, THA1P, LOC100996291*			6 tissues		5 altered motifs
rs12598434	16	85973059	42,454	40,549	C>A	0.33	0.99	0.97	1.01	3.7E-01	*IRF8*			BLD, LIV, GI		
rs6017737	20	44743311	42,499	40,567	A>G	0.21	0.99	0.97	1.01	3.7E-01	*CD40, NCOA5, RPL13P2*					Pou2f2
rs17140399	7	18998604	42,494	40,570	A>C	0.37	0.99	0.97	1.01	3.7E-01	*HDAC9, NPM1P13*	intronic		FAT, MUS, SKIN	10 tissues	CDP,Zfp105
rs6990483	8	128699278	42,501	40,576	G>A	0.12	1.01	0.98	1.05	3.7E-01	*MYC*			8 tissues	4 tissues	NRSF,SMC3
rs2239680	17	76219783	42,501	40,574	A>G	0.28	1.01	0.99	1.03	3.7E-01	*BIRC5, TK1, AFMID, TMEM235, THA1P, LOC100996291*	3'-UTR		10 tissues		Evi-1,PPAR
rs3736863	10	6014203	42,505	40,575	A>T	0.22	1.01	0.99	1.04	3.7E-01	*IL2RA, IL15RA, FBXO18*	intronic		6 tissues	BLD,BLD	
rs7796078	7	18673238	42,503	40,575	A>G	0.32	0.99	0.97	1.01	3.7E-01	*HDAC9*	intronic				CEBPG,Hdx
rs12038005	1	92219364	42,462	40,541	T>A	0.39	0.99	0.97	1.01	3.7E-01	*TGFBR3*	intronic		4 tissues		HNF1,SRF
rs7816685	8	79658906	42,509	40,577	A>G	0.07	0.98	0.94	1.02	3.7E-01	*IL7, ZC2HC1A, PRKRIRP7*	intronic		FAT, SKIN		AP-1,LXR,RORalpha1
rs3024583	16	27359296	42,503	40,566	G>A	0.08	0.98	0.95	1.02	3.8E-01	*IL4R*	intronic		7 tissues	BLD	4 altered motifs
rs17659900	3	3135505	42,471	40,526	A>G	0.14	1.01	0.98	1.04	3.8E-01	*IL5RA, TRNT1, CNTN4*	intronic				Foxc1,TATA
rs12669760	7	18630293	42,504	40,568	C>G	0.23	1.01	0.99	1.04	3.8E-01	*HDAC9, LOC100419901*	intronic	SKIN	BRST, BLD, SKIN	5 tissues	Maf,NF-E2
rs8177762	10	6020191	42,500	40,563	G>A	0.06	1.02	0.98	1.06	3.8E-01	*IL2RA, IL15RA, FBXO18*		24 tissues	4 tissues	30 tissues	25 altered motifs
rs3006423	1	153401664	42,399	40,529	A>G	0.08	0.98	0.95	1.02	3.8E-01	*S100A7, S100A8, S100A7P1, S100A7A, S100A7P2, LOC645900, S100A7L2*			SKIN		E2F,TATA
rs374450	1	12305286	42,510	40,577	G>A	0.14	0.99	0.96	1.02	3.8E-01	*TNFRSF1B, VPS13D, LOC390998*	intronic		4 tissues		IRC900814,Pou1f1,Pou2f2
rs1926263	1	92162679	42,506	40,576	A>T	0.11	1.01	0.98	1.05	3.8E-01	*TGFBR3*	intronic		14 tissues		
rs9368726	6	32438542	42,421	40,510	A>G	0.31	0.99	0.97	1.01	3.8E-01	*HLA-DRA, HLA-DRB5, HLA-DRB9*					Evi-1,PLZF
rs1003199	5	158755566	42,495	40,569	A>G	0.49	0.99	0.97	1.01	3.8E-01	*IL12B, UBLCP1, LOC285626, RNU4ATAC2P*	intronic				Lmo2-complex,Sox
rs8832	16	27375787	42,506	40,572	G>A	0.44	0.99	0.97	1.01	3.8E-01	*IL4R, IL21R*	3'-UTR	LNG	9 tissues	7 tissues	HMG-IY
rs2276338	2	9645789	42,507	40,573	A>G	0.42	0.99	0.97	1.01	3.8E-01	*ADAM17, CPSF3, IAH1*	intronic		ADRL		5 altered motifs
rs72823022	17	40513732	42,510	40,577	A>G	0.08	1.02	0.98	1.06	3.8E-01	*STAT3, STAT5A, PTRF*	intronic		19 tissues		
rs10157808	1	23076916	42,494	40,568	G>C	0.07	1.02	0.98	1.06	3.8E-01	*EPHB2, MIR4684*	intronic			32 tissues	
rs20544	20	44645010	41,686	39,915	A>G	0.43	0.99	0.97	1.01	3.8E-01	*MMP9, SLC12A5, NCOA5, ZNF335, FTLP1, LOC100128028*	3'-UTR		LIV		SETDB1,Znf143
rs2021840	1	172643220	42,509	40,574	A>G	0.25	0.99	0.97	1.01	3.8E-01	*FASLG*			BLD, PLCNT, BONE		Maf
rs928344	6	167571430	42,502	40,570	T>A	0.36	0.99	0.97	1.01	3.8E-01	*CCR6, GPR31, TCP10L2*			ESDR, BRST, BLD	BLD,BLD,BLD	Evi-1,SIX5,p53
rs1326280	1	198652769	42,510	40,576	G>A	0.05	0.98	0.94	1.03	3.8E-01	*PTPRC, PEBP1P3*	intronic	BLD, THYM	5 tissues	BLD	BHLHE40,Gm397,Pax-4

SNP	Chr	Position[a]	N Cases	N Controls	Allele	MAF	OR[b]	95%CI		*p* -value	Gene annotation	dbSNP functional annotation	Promoter histone marks	Enhancer histone marks	DNAse	Motifs changed
rs12700014	7	18964076	42,479	40,540	A>G	0.47	0.99	0.97	1.01	3.8E-01	*HDAC9, NPM1P13*	intronic			CRVX	AP-1,Maf,NF-E2
rs11878563	19	6670910	42,496	40,563	G>A	0.43	1.01	0.99	1.03	3.8E-01	*C3, TNFSF14*		BLD, GI	5 tissues	BLD,BLD,LIV	Myc
rs1029489	16	27376217	42,504	40,571	G>A	0.40	0.99	0.97	1.01	3.8E-01	*IL4R, IL21R*				13 tissues	4 altered motifs
rs155128	2	182305254	42,506	40,567	A>G	0.36	1.01	0.99	1.03	3.8E-01	*ITGA4*			5 tissues	THYM	
rs1470453	17	62404390	42,508	40,574	G>A	0.23	1.01	0.99	1.03	3.8E-01	*PECAM1, RPL31P57*	intronic	BLD, CRVX	19 tissues	12 tissues	5 altered motifs
rs10494878	1	206915809	42,505	40,571	A>G	0.24	1.01	0.99	1.03	3.8E-01	*IL10, MAPKAPK2, RPS14P1*					CEBPG
rs6972607	7	18834534	42,452	40,508	G>A	0.18	1.01	0.99	1.04	3.8E-01	*HDAC9*	intronic		GI		HMG-IY,Sox,p300
rs1629017	7	18620165	42,388	40,464	G>A	0.46	0.99	0.97	1.01	3.8E-01	*HDAC9, LOC100419901*	intronic		BRN	BRN,MUS	BATF
rs11216860	11	118240332	42,505	40,574	A>G	0.26	1.01	0.99	1.03	3.8E-01	*CD3D, CD3G, UBE4A, ATP5L, LOC100131626*	intronic				PU.1
rs9405108	6	32438648	42,500	40,561	G>A	0.31	0.99	0.97	1.01	3.8E-01	*HLA-DRA, HLA-DRB5, HLA-DRB9*					Pdx1
rs2588597	7	18817610	42,498	40,568	A>G	0.24	1.01	0.99	1.03	3.8E-01	*HDAC9*	intronic		4 tissues		5 altered motifs
rs4659190	1	117737486	42,507	40,575	G>A	0.23	1.01	0.99	1.03	3.8E-01	*VTCN1*	intronic				11 altered motifs
rs1982774	11	69442963	42,500	40,566	G>A	0.44	1.01	0.99	1.03	3.8E-01	*CCND1, ORAOV1, LOC100996515*					GATA,SREBP,Sox
rs7904311	10	6424490	42,439	40,507	G>A	0.14	1.01	0.98	1.04	3.8E-01	*PRKCQ, LOC399715, DKFZp667F0711*		5 tissues	13 tissues	8 tissues	Myb
rs1627122	15	40255964	42,509	40,574	G>A	0.45	0.99	0.97	1.01	3.8E-01	*GPR176, EIF2AK4, H3F3AP1, LOC100505534*	intronic		SKIN		Sox
rs9843143	3	30729229	42,502	40,566	C>G	0.49	0.99	0.97	1.01	3.8E-01	*TGFBR2, GADL1*	intronic		14 tissues		COMP1,Ets,GR
rs3740286	10	90751340	42,354	40,436	A>G	0.36	1.01	0.99	1.03	3.8E-01	*ACTA2, FAS, FAS-AS1*	intronic	24 tissues		40 tissues	6 altered motifs
rs7031302	9	101873532	42,314	40,257	A>G	0.20	1.01	0.99	1.04	3.8E-01	*COL15A1, TGFBR1*	intronic		BLD, SKIN	SKIN	HDAC2
rs12722605	10	6053163	42,506	40,571	A>T	0.15	0.99	0.96	1.02	3.8E-01	*IL2RA, IL15RA*	3'-UTR				5 altered motifs
rs284197	1	92237547	42,503	40,567	G>A	0.18	0.99	0.96	1.01	3.8E-01	*TGFBR3*	intronic		BRST, BLD		9 altered motifs
rs4233207	1	36977565	42,501	40,570	A>G	0.40	1.01	0.99	1.03	3.8E-01	*CSF3R, MRPS15*			4 tissues	9 tissues	GATA,LBP-1,LBP-9
rs2022090	7	18724179	42,468	40,534	A>G	0.11	0.99	0.96	1.02	3.8E-01	*HDAC9*	intronic				
rs16906062	8	79659182	42,501	40,571	A>T	0.06	0.98	0.94	1.02	3.8E-01	*IL7, ZC2HC1A, PRKRIRP7*	intronic				9 altered motifs
rs9658691	10	90756163	42,305	40,322	A>G	0.12	1.01	0.98	1.04	3.8E-01	*ACTA2, FAS, FAS-AS1*	intronic	BLD	6 tissues	BLD,BLD,OVRY	Hoxa9,Hoxb9
rs768410	16	85945728	42,501	40,569	A>C	0.42	1.01	0.99	1.03	3.8E-01	*IRF8*	intronic	FAT	16 tissues	4 tissues	AFP1,CTCF,HDAC2
rs6958814	7	18689386	42,508	40,576	G>A	0.08	0.98	0.95	1.02	3.8E-01	*HDAC9*	intronic		4 tissues	CRVX	YY1
rs17598983	10	72316973	42,482	40,556	G>A	0.19	1.01	0.99	1.04	3.8E-01	*PRF1, PALD1*	intronic		ESDR, HRT		Ets,SMC3,TR4
rs3792421	3	3149791	42,474	40,552	A>G	0.23	1.01	0.99	1.03	3.8E-01	*IL5RA, TRNT1, CRBN*	intronic		BLD		Nkx3
rs744751	3	30735937	42,506	40,575	G>A	0.33	1.01	0.99	1.03	3.8E-01	*TGFBR2, GADL1*			9 tissues	BRN	RFX5
rs1015407	7	18713259	42,414	40,479	A>G	0.19	1.01	0.99	1.04	3.8E-01	*HDAC9*	intronic				15 altered motifs
rs17139560	7	18712720	42,510	40,577	G>A	0.08	0.98	0.95	1.02	3.8E-01	*HDAC9*	intronic				E4F1
rs12123436	1	92215089	42,501	40,565	G>A	0.40	0.99	0.97	1.01	3.8E-01	*TGFBR3*	intronic	CRVX	14 tissues	CRVX	VDR
rs17069904	18	60032949	42,509	40,573	G>A	0.10	0.99	0.95	1.02	3.8E-01	*TNFRSF11A, RPL17P44*	intronic		BLD	BLD,BLD	DMRT4
rs9818482	3	121850554	42,371	40,481	A>C	0.45	0.99	0.97	1.01	3.8E-01	*CD86*					Nanog,Pou3f1
rs10280633	7	18818806	42,501	40,569	G>A	0.17	1.01	0.99	1.04	3.9E-01	*HDAC9*	intronic			BRN,LNG	Sox
rs1089359	7	18639829	42,504	40,572	A>T	0.12	0.99	0.96	1.02	3.9E-01	*HDAC9, LOC100419901*	intronic				4 altered motifs
rs2243154	3	159716242	42,509	40,577	G>A	0.09	1.02	0.98	1.05	3.9E-01	*IL12A*					
rs8058904	16	85951682	42,498	40,572	A>G	0.18	1.01	0.99	1.04	3.9E-01	*IRF8*	intronic		BLD, KID	6 tissues	Cdx2,Pou3f2,STAT
rs8177688	10	6007549	42,507	40,575	T>A	0.09	1.02	0.98	1.05	3.9E-01	*IL2RA, IL15RA, FBXO18*	intronic		5 tissues	GI,PANC	
rs7090504	10	6091017	40,142	38,444	A>T	0.21	0.99	0.96	1.01	3.9E-01	*IL2RA, RBM17, RPL32P23*	intronic		BLD, GI		7 altered motifs
rs1418555	1	218523650	42,508	40,575	G>A	0.23	0.99	0.97	1.01	3.9E-01	*TGFB2, RRP15, RPS26P17, LOC728463*	intronic	13 tissues	10 tissues	6 tissues	10 altered motifs
rs34739845	18	60024435	42,507	40,573	G>A	0.11	0.99	0.95	1.02	3.9E-01	*TNFRSF11A*	intronic		BLD	BLD,BLD	Gfi1
rs175741		135691879	42,509	40,575	C>A	0.08	0.98	0.95	1.02	3.9E-01	*CD40LG, LINC00892*		BLD	FAT, BLD		Maf
rs12533489	7	19016933	42,506	40,576	G>C	0.16	0.99	0.96	1.02	3.9E-01	*HDAC9, NPM1P13*	intronic		10 tissues	6 tissues	6 altered motifs
rs11165294	1	92174737	42,507	40,575	G>A	0.11	0.99	0.96	1.02	3.9E-01	*TGFBR3*	intronic		4 tissues		
rs8044444	16	27328543	42,508	40,576	G>A	0.15	0.99	0.96	1.02	3.9E-01	*IL4R, NSMCE1, FLJ21408*	intronic	BLD, SKIN, GI	18 tissues	5 tissues	Gfi1,RFX5,Sox
rs424971	16	85946450	42,501	40,565	A>G	0.43	1.01	0.99	1.03	3.9E-01	*IRF8*	intronic		14 tissues	9 tissues	4 altered motifs
rs6060563	20	30248803	42,494	40,574	G>A	0.32	0.99	0.97	1.01	3.9E-01	*BCL2L1, COX4I2*			7 tissues		13 altered motifs
rs987106	5	35875593	42,499	40,569	A>T	0.46	0.99	0.97	1.01	3.9E-01	*IL7R, CAPSL*	intronic		BLD, THYM		Pbx-1,p300
rs12486781	3	121725087	42,508	40,576	G>A	0.10	0.99	0.95	1.02	3.9E-01	*CD86, ILDR1*	intronic		ESDR, ESC, BLD		Ik-2,NF-AT
rs1053004	17	40466092	42,479	40,545	A>G	0.37	1.01	0.99	1.03	3.9E-01	*STAT3, STAT5A, STAT5B*	3'-UTR			23 tissues	CTCF,Pbx3,Smad4
rs1427363	2	204837629	42,420	40,508	G>A	0.24	1.01	0.99	1.03	3.9E-01	*ICOS*					Pax-4
rs805385	2	54097112	42,502	40,572	G>A	0.27	0.99	0.97	1.01	3.9E-01	*GPR75, PSME4, GPR75-ASB3, MIR3682*	intronic				RXRA
rs2839690	10	44875166	42,499	40,576	A>G	0.21	0.99	0.97	1.01	3.9E-01	*CXCL12, RPL9P21*	intronic		4 tissues		Pax-6
rs1409876	10	6543063	42,506	40,573	A>G	0.37	0.99	0.97	1.01	3.9E-01	*PRKCQ*	intronic		BRST, SKIN, MUS		Elf3,SETDB1
rs805352	2	54158957	42,487	40,558	G>A	0.31	0.99	0.97	1.01	3.9E-01	*PSME4*	intronic				PRDM1
rs9606615	22	17580813	42,500	40,569	A>G	0.46	1.01	0.99	1.03	3.9E-01	*IL17RA, CECR6, CECR5, CECR7, RPL31P62, LOC100996342*	intronic		BLD, THYM		Nkx2,STAT
rs2395185	6	32433167	42,503	40,571	C>A	0.31	0.99	0.97	1.01	3.9E-01	*HLA-DRA, HLA-DRB9*					4 altered motifs
rs2588599	7	18819266	42,504	40,570	G>A	0.17	1.01	0.99	1.04	3.9E-01	*HDAC9*	intronic		GI	GI	CEBPG,HDAC2
rs4941129	18	60000477	42,474	40,544	A>G	0.30	0.99	0.97	1.01	3.9E-01	*TNFRSF11A, KIAA1468*	intronic		5 tissues		
rs1726614	7	18628779	42,481	40,546	G>A	0.06	1.02	0.98	1.06	4.0E-01	*HDAC9, LOC100419901*	intronic				Gm397,Mtf1

SNP	Chr	Position[a]	N Cases	N Controls	Allele	MAF	OR[b]	95%CI		*p* -value	Gene annotation	dbSNP functional annotation	Promoter histone marks	Enhancer histone marks	DNAse	Motifs changed
rs17807076	22	17582776	42,374	40,454	G>A	0.28	1.01	0.99	1.03	4.0E-01	*IL17RA, CECR6, CECR5, CECR7, RPL31P62, LOC100996342*	intronic		6 tissues	KID,THYM	5 altered motifs
rs9803750	1	198644124	42,498	40,562	G>A	0.30	1.01	0.99	1.03	4.0E-01	*PTPRC, PEBP1P3*	intronic	BLD	BLD		4 altered motifs
rs2810888	1	92156749	42,506	40,568	A>G	0.11	1.01	0.98	1.05	4.0E-01	*TGFBR3, HSP90B3P*	intronic		FAT		Hdx,Sox,Zfp105
rs147968	16	85945839	42,492	40,566	A>G	0.42	1.01	0.99	1.03	4.0E-01	*IRF8*	intronic		17 tissues	6 tissues	GR
rs805351	2	54157904	42,508	40,577	G>A	0.31	0.99	0.97	1.01	4.0E-01	*PSME4*	intronic			LNG	Maf
rs1805016	16	27374927	42,287	40,368	A>C	0.05	1.02	0.97	1.07	4.0E-01	*IL4R, IL21R*	missense		8 tissues		4 altered motifs
rs284183	1	92231746	42,496	40,566	A>C	0.32	0.99	0.97	1.01	4.0E-01	*TGFBR3*	intronic		7 tissues	PLCNT	13 altered motifs
rs3116536	2	204834613	42,505	40,576	A>G	0.24	1.01	0.99	1.03	4.0E-01	*ICOS*					Ehf,Elf3
rs11669203	19	7979801	42,469	40,563	C>G	0.23	1.01	0.99	1.03	4.0E-01	*ELAVL1, MAP2K7, SNAPC2, TIMM44, LRRC8E, FLJ22184, EVI5L, LOC388499, CTXN1, TGFBR3L*		SKIN	8 tissues	SKIN,MUS,SKIN	9 altered motifs
rs2588604	7	18820271	42,495	40,562	A>G	0.17	1.01	0.99	1.04	4.0E-01	*HDAC9*	intronic		IPSC, SKIN	SKIN	SRF
rs4899056	14	62189531	41,912	39,983	G>A	0.09	1.02	0.98	1.05	4.0E-01	*HIF1A, SNAPC1, HIF1A-AS2*	intronic		4 tissues	ESDR	15 altered motifs
rs17139658	7	18730268	42,227	40,264	A>G	0.07	1.02	0.98	1.06	4.0E-01	*HDAC9*	intronic				4 altered motifs
rs16902407	8	128765361	42,491	40,569	G>A	0.22	0.99	0.97	1.01	4.0E-01	*MYC, MIR1204*			ADRL		STAT
rs4750491	10	6499228	42,502	40,572	A>G	0.32	0.99	0.97	1.01	4.0E-01	*PRKCQ*	intronic		THYM		Pou2f2
rs17129664	1	67583298	42,508	40,576	G>A	0.08	0.98	0.95	1.02	4.0E-01	*IL23R, C1orf141*	intronic				STAT,ZNF263
rs11117415	16	85950686	42,507	40,577	A>G	0.05	1.02	0.97	1.07	4.0E-01	*IRF8*	intronic	GI	14 tissues	MUS,GI	5 altered motifs
rs10492096	12	6580582	40,206	37,979	A>G	0.21	1.01	0.99	1.04	4.0E-01	*CD27, VAMP1, NCAPD2, MRPL51, TAPBPL, PKP2P1, SRP14P1, CD27-AS1, SCARNA10*				13 tissues	
rs41295071	10	6118111	42,471	40,544	G>A	0.09	1.01	0.98	1.05	4.0E-01	*IL2RA, RBM17, RPL32P23*					6 altered motifs
rs806399	10	6468605	42,504	40,577	G>A	0.25	0.99	0.97	1.01	4.0E-01	*PRKCQ*			MUS		YY1
rs3024633	16	27366499	42,509	40,577	A>G	0.08	0.98	0.95	1.02	4.0E-01	*IL4R, IL21R*	3'-UTR				DEC,ERalpha-a,Hand1
rs3024544	16	27353357	42,508	40,576	G>A	0.14	0.99	0.96	1.02	4.0E-01	*IL4R*	intronic		BRST, BLD		FXR
rs212672	7	18797190	42,508	40,573	T>A	0.25	1.01	0.99	1.03	4.0E-01	*HDAC9*	intronic		5 tissues		CIZ
rs1488371	3	45938089	42,505	40,576	A>C	0.39	1.01	0.99	1.03	4.0E-01	*CXCR6, CCR9, SDHDP4, FYCO1*	intronic		13 tissues		4 altered motifs
rs17432448	7	18548419	42,507	40,572	A>G	0.36	0.99	0.97	1.01	4.0E-01	*HDAC9*	intronic	12 tissues	12 tissues	17 tissues	Pou1f1
rs17350431	7	18933110	42,461	40,548	G>A	0.24	1.01	0.99	1.03	4.0E-01	*HDAC9*	intronic		BRST, SKIN	BLD,SKIN,SKIN	BHLHE40,Mef2
rs2276767	3	30732821	42,499	40,570	C>A	0.33	1.01	0.99	1.03	4.0E-01	*TGFBR2, GADL1*	intronic		17 tissues	LNG,VAS	Pax-5,RXRA
rs303438	10	30733493	42,502	40,570	G>A	0.35	0.99	0.97	1.01	4.0E-01	*CCND3P, MAP3K8*	intronic		5 tissues		
rs17879478	1	92173722	42,507	40,575	A>C	0.11	0.99	0.96	1.02	4.0E-01	*TGFBR3*	intronic		5 tissues	HRT	
rs16949	17	26124699	42,505	40,572	A>G	0.24	0.99	0.97	1.01	4.0E-01	*NOS2, LOC645754*	intronic	GI	GI, LIV	IPSC,GI,GI	
rs2588596	7	18816536	42,496	40,567	A>C	0.17	1.01	0.99	1.04	4.0E-01	*HDAC9*	intronic				Cdx
rs12153168	5	158785885	42,505	40,574	A>G	0.32	0.99	0.97	1.01	4.0E-01	*IL12B, LOC285626, RNU4ATAC2P*	intronic		BLD		4 altered motifs
rs10486329	7	18994192	42,508	40,573	T>A	0.46	1.01	0.99	1.03	4.0E-01	*HDAC9, NPM1P13*	intronic				Ik-1,LUN-1,Pou2f2
rs4749924	10	6082396	42,505	40,574	A>C	0.32	0.99	0.97	1.01	4.0E-01	*IL2RA, RBM17, RPL32P23*	intronic	BLD	ESDR, BLD		Hdx,NF-I
rs6678564	1	92257650	42,501	40,571	C>G	0.08	1.02	0.98	1.05	4.0E-01	*TGFBR3*	intronic	SKIN, GI	22 tissues	7 tissues	Mxi1,Myf,RP58
rs2023937	7	19037051	42,235	40,261	A>T	0.10	1.01	0.98	1.05	4.0E-01	*HDAC9, NPM1P13*			FAT, SKIN		4 altered motifs
rs77149222	11	69437784	42,502	40,563	A>T	0.44	1.01	0.99	1.03	4.0E-01	*CCND1, ORAOV1, LOC100996515*					22 altered motifs
rs2704289	7	18579254	42,508	40,576	G>A	0.26	1.01	0.99	1.03	4.0E-01	*HDAC9, LOC100419901*	intronic		HRT, KID, SKIN		HP1-site-factor,Pax-4
rs805449	2	54178132	42,506	40,575	A>C	0.27	0.99	0.97	1.01	4.0E-01	*PSME4*	intronic		BONE		8 altered motifs
rs7976678	12	6537544	42,507	40,576	G>A	0.37	0.99	0.97	1.01	4.0E-01	*CD27, LTBR, VAMP1, TAPBPL, PKP2P1, RPL31P10, SRP14P1, CD27-AS1*		BLD	BLD	BLD,OVRY,VAS	DMRT2,DMRT7,Nr2f2
rs10493860	1	92212703	42,437	40,505	G>A	0.17	1.01	0.98	1.04	4.0E-01	*TGFBR3*	intronic			BLD	E2F
rs266093	10	44866208	42,506	40,566	G>C	0.37	0.99	0.97	1.01	4.0E-01	*CXCL12, RPL9P21*	3'-UTR	ESC, IPSC	7 tissues	8 tissues	ELF1,PU.1
rs4934433	10	90740953	42,505	40,572	A>C	0.27	1.01	0.99	1.03	4.0E-01	*ACTA2, FAS, ACTA2-AS1, FAS-AS1*	intronic	VAS, GI, LNG	13 tissues		Mtf1
rs2028012	7	18820482	42,504	40,573	G>A	0.17	1.01	0.98	1.04	4.0E-01	*HDAC9*	intronic		SKIN		5 altered motifs
rs41295061	10	6114660	42,510	40,576	C>A	0.09	1.02	0.98	1.05	4.0E-01	*IL2RA, RBM17, RPL32P23*		BLD, FAT, GI	4 tissues	12 tissues	
rs163549	3	3127730	42,295	40,334	A>C	0.39	0.99	0.97	1.01	4.1E-01	*IL5RA, TRNT1, CNTN4*	intronic		ESDR, BLD	BLD	Irf,SRF
rs866066	9	5450953	42,482	40,551	G>A	0.48	0.99	0.97	1.01	4.1E-01	*CD274, PLGRKT, LOC100419687*	intronic	24 tissues		38 tissues	15 altered motifs
rs10807289	6	43709785	42,504	40,569	A>G	0.49	0.99	0.97	1.01	4.1E-01	*VEGFA, LOC100132242*					
rs1178100	7	18735515	42,508	40,573	G>A	0.29	1.01	0.99	1.03	4.1E-01	*HDAC9*	intronic				8 altered motifs
rs17140387	7	18995719	42,488	40,558	C>G	0.37	0.99	0.97	1.01	4.1E-01	*HDAC9, NPM1P13*	intronic				Duxl,HNF6
rs9325604	10	90775756	42,510	40,577	A>G	0.16	1.01	0.98	1.04	4.1E-01	*ACTA2, FAS, FAS-AS1, MIR4679-1, MIR4679-2*			BLD	IPSC	Irf
rs2301113	14	62206548	42,506	40,572	A>C	0.22	0.99	0.97	1.01	4.1E-01	*HIF1A, SNAPC1, HIF1A-AS2*	intronic		HRT	4 tissues	PEBP
rs17749316	2	191840709	42,509	40,576	C>G	0.07	0.98	0.95	1.02	4.1E-01	*GLS, STAT1, LOC100420571*	intronic		HRT		Tgif1
rs3024536	16	27352713	42,506	40,574	G>A	0.14	0.99	0.96	1.02	4.1E-01	*IL4R*	intronic		6 tissues	SKIN	4 altered motifs
rs7647903	3	3133791	42,504	40,576	G>A	0.22	0.99	0.97	1.01	4.1E-01	*IL5RA, TRNT1, CNTN4*	3'-UTR				12 altered motifs
rs650652	10	6540723	42,496	40,569	G>A	0.34	1.01	0.99	1.03	4.1E-01	*PRKCQ*	intronic	BLD	6 tissues	6 tissues	Mef2,THAP1
rs2588623	7	18821685	42,493	40,564	A>G	0.17	1.01	0.98	1.04	4.1E-01	*HDAC9*	intronic		4 tissues	ADRL	Hsf

SNP	Chr	Position[a]	N Cases	N Controls	Allele	MAF	OR[b]	95%CI		*p* -value	Gene annotation	dbSNP functional annotation	Promoter histone marks	Enhancer histone marks	DNAse	Motifs changed
rs498186	11	102669645	42,506	40,574	A>C	0.45	0.99	0.97	1.01	4.1E-01	*MMP1, MMP3, MMP10, CSNK1A1P2, WTAPP1, LOC100421658*	intronic		5 tissues		EBF
rs2520345	7	18820355	42,500	40,570	A>C	0.17	1.01	0.98	1.04	4.1E-01	*HDAC9*	intronic		IPSC, SKIN		Foxo,GR,Sox
rs12535294	7	18818397	42,495	40,567	A>G	0.24	1.01	0.99	1.03	4.1E-01	*HDAC9*	intronic		7 tissues	5 tissues	CTCF,TR4
rs743409	22	22129215	42,507	40,574	G>A	0.49	1.01	0.99	1.03	4.1E-01	*MAPK1, YPEL1*	intronic		8 tissues	33 tissues	Pou2f2,Sox
rs3024537	16	27352819	42,507	40,576	G>A	0.14	0.99	0.96	1.02	4.1E-01	*IL4R*	intronic		6 tissues	BLD,BLD	9 altered motifs
rs9268923	6	32432835	42,500	40,569	G>A	0.31	0.99	0.97	1.01	4.1E-01	*HLA-DRA, HLA-DRB9*					5 altered motifs
rs17348617	7	18727970	42,488	40,558	C>A	0.10	0.99	0.95	1.02	4.1E-01	*HDAC9*	intronic				AP-3
rs6974011	7	18838463	42,504	40,573	A>C	0.38	1.01	0.99	1.03	4.1E-01	*HDAC9*	intronic		5 tissues	7 tissues	Mef2
rs7231380	18	60095095	42,509	40,576	A>G	0.22	0.99	0.97	1.01	4.1E-01	*ACTBP9, TNFRSF11A, RPL17P44*					Mef2,Sox
rs2588603	7	18820126	42,504	40,573	G>A	0.17	1.01	0.98	1.04	4.1E-01	*HDAC9*	intronic		GI, SKIN	5 tissues	Nrf-2,Zfp105
rs1143627	2	113594387	42,493	40,570	A>G	0.34	0.99	0.97	1.01	4.1E-01	*IL1B, LOC100128413*		9 tissues	11 tissues	26 tissues	5 altered motifs
rs9607340	22	22222320	41,508	39,772	A>C	0.49	0.99	0.97	1.01	4.1E-01	*MAPK1*		24 tissues		47 tissues	BCL
rs991694	3	30702140	42,509	40,577	G>A	0.14	1.01	0.98	1.04	4.1E-01	*TGFBR2*	intronic	BLD	15 tissues		EBF
rs12404952	1	92215725	42,506	40,572	G>A	0.06	1.02	0.98	1.06	4.1E-01	*TGFBR3*	intronic	CRVX	18 tissues	CRVX,SKIN	4 altered motifs
rs3863318	11	117811167	42,491	40,562	C>A	0.44	0.99	0.97	1.01	4.1E-01	*IL10RA, TMPRSS13*					Pou2f2
rs10508307	10	6497307	42,502	40,575	G>A	0.16	1.01	0.98	1.04	4.1E-01	*PRKCQ*	intronic				
rs2025818	10	6564577	42,502	40,567	A>G	0.50	0.99	0.97	1.01	4.1E-01	*PRKCQ*	intronic		BLD		5 altered motifs
rs78352136	2	113537600	42,502	40,568	T>A	0.30	1.01	0.99	1.03	4.1E-01	*IL1A, IL1B, CKAP2L*	intronic		BRST, SKIN		6 altered motifs
rs10266741	7	18827914	42,500	40,563	C>G	0.17	1.01	0.98	1.04	4.1E-01	*HDAC9*	intronic			4 tissues	GATA
rs7811991	7	18918559	42,505	40,575	A>G	0.28	1.01	0.99	1.03	4.1E-01	*HDAC9*	intronic			BRN	
rs10434	6	43753212	42,492	40,567	G>A	0.47	1.01	0.99	1.03	4.1E-01	*VEGFA*	3'-UTR			PANC,SKIN	Mxi1,Nanog,SREBP
rs2069763	4	123377482	42,448	40,508	C>A	0.36	1.01	0.99	1.03	4.1E-01	*IL2, ADAD1*	synonymous	BLD, GI	BLD	THYM	Hand1,Msx-1
rs11259212	10	6537425	42,506	40,575	A>G	0.20	1.01	0.99	1.04	4.1E-01	*PRKCQ*	intronic	BLD	5 tissues	9 tissues	Mrg,Pax-5,Pbx3
rs4721725	7	18812501	42,487	40,566	G>A	0.17	1.01	0.98	1.04	4.1E-01	*HDAC9*	intronic		ESDR		Hoxd10,Ik-3
rs801520	7	18702222	42,493	40,572	C>A	0.11	0.99	0.96	1.02	4.2E-01	*HDAC9*	intronic		BLD		4 altered motifs
rs1926196	10	90753748	42,478	40,535	G>A	0.50	1.01	0.99	1.03	4.2E-01	*ACTA2, FAS, FAS-AS1*	intronic	13 tissues	12 tissues	8 tissues	NF-I,RREB-1
rs3118956	10	30798293	42,506	40,572	A>C	0.18	1.01	0.98	1.04	4.2E-01	*MAP3K8, HNRNPA1P32*			5 tissues		4 altered motifs
rs744120	17	76207524	42,339	40,435	G>C	0.26	0.99	0.97	1.01	4.2E-01	*BIRC5, TK1, SYNGR2, AFMID, TMEM235, THA1P, C17orf99, LOC100996291*		ESDR, MUS	6 tissues	MUS,MUS,BRN	CACD,GLI,NRSF
rs34256674	18	60029217	42,504	40,574	A>G	0.48	0.99	0.97	1.01	4.2E-01	*TNFRSF11A*	intronic			KID	Pax-4,STAT
rs2069812	5	131879916	42,493	40,575	G>A	0.30	1.01	0.99	1.03	4.2E-01	*IL5, RAD50*			BLD		
rs9394859	6	42040026	42,492	40,544	A>G	0.18	1.01	0.99	1.04	4.2E-01	*CCND3, TAF8, C6orf132*	intronic		FAT, HRT		4 altered motifs
rs284878	1	92174260	42,510	40,577	G>A	0.06	1.02	0.98	1.06	4.2E-01	*TGFBR3*	synonymous		5 tissues	HRT	Crx,SP1
rs1805011	16	27373872	42,098	40,290	A>C	0.11	0.99	0.96	1.02	4.2E-01	*IL4R, IL21R*	missense		5 tissues		12 altered motifs
rs2241049	22	17587680	42,493	40,569	A>G	0.36	0.99	0.97	1.01	4.2E-01	*IL17RA, CECR6, CECR5, CECR7, RPL31P62, LOC100996342*	intronic	BLD	BLD, THYM, SPLN	4 tissues	GATA
rs1110470	16	27336427	42,405	40,527	G>A	0.48	1.01	0.99	1.03	4.2E-01	*IL4R, FLJ21408*	intronic	BLD, GI	14 tissues	9 tissues	BRCA1
rs163550	3	3126727	42,226	40,327	C>G	0.25	1.01	0.99	1.03	4.2E-01	*IL5RA, TRNT1, CNTN4*	intronic		ESDR, BLD, GI	ESDR	SRF
rs2287848	19	6696342	42,495	40,563	G>A	0.39	1.01	0.99	1.03	4.2E-01	*C3, TNFSF14, TRIP10, GPR108*	intronic			BLD	
rs503830	15	40247689	42,393	40,437	G>A	0.41	1.01	0.99	1.03	4.2E-01	*GPR176, EIF2AK4, H3F3AP1, LOC100505534*	intronic				6 altered motifs
rs4250	1	173120636	42,474	40,554	A>G	0.06	0.98	0.94	1.03	4.2E-01	*TNFSF4, GOT2P2*					
rs17878995	3	3118142	42,506	40,575	C>A	0.26	1.01	0.99	1.03	4.2E-01	*IL5RA, CNTN4*	intronic				BDP1,Ets
rs25676	3	119305379	42,479	40,560	A>T	0.28	0.99	0.97	1.01	4.2E-01	*ADPRH, CD80, PLA1A, RPL10P7*	synonymous	MUS, BLD	7 tissues		
rs12129174	1	92222850	42,507	40,569	G>A	0.17	1.01	0.98	1.04	4.2E-01	*TGFBR3*	intronic		4 tissues		5 altered motifs
rs62515814	8	128764965	42,502	40,565	A>G	0.37	0.99	0.97	1.01	4.2E-01	*MYC, MIR1204*			ADRL		20 altered motifs
rs2695028	7	18627486	42,395	40,435	A>G	0.32	1.01	0.99	1.03	4.2E-01	*HDAC9, LOC100419901*	intronic				20 altered motifs
rs621685	10	6542667	41,861	39,872	G>A	0.25	0.99	0.97	1.01	4.2E-01	*PRKCQ*	intronic		BLD, MUS, BRST		10 altered motifs
rs11887698	2	191854874	42,505	40,574	A>G	0.15	1.01	0.98	1.04	4.2E-01	*GLS, STAT1, STAT4, LOC100420571*	intronic		ESDR, FAT, MUS		AhR,SZF1-1,YY1
rs3024554	16	27355285	42,504	40,575	C>A	0.14	0.99	0.96	1.02	4.2E-01	*IL4R*	intronic			LIV	Rad21
rs2031541	13	43221530	42,507	40,576	A>G	0.32	1.01	0.99	1.03	4.2E-01	*TNFSF11*					AP-1,GATA
rs12404261	1	92213835	42,510	40,576	G>A	0.06	1.02	0.97	1.06	4.2E-01	*TGFBR3*	intronic		7 tissues		4 altered motifs
rs10798176	1	172675525	42,497	40,563	A>G	0.18	0.99	0.96	1.02	4.2E-01	*FASLG, SLC25A38P1*					Ncx,Zfp410
rs982097	10	44828234	42,488	40,565	A>G	0.43	1.01	0.99	1.03	4.2E-01	*CXCL12, LOC100130539*			MUS		4 altered motifs
rs727851	7	18786817	42,503	40,576	C>A	0.21	1.01	0.99	1.04	4.2E-01	*HDAC9*	intronic			LNG,IPSC	TATA
rs2071286	6	32179896	42,509	40,576	G>A	0.22	1.01	0.99	1.03	4.2E-01	*AGER, NOTCH4, PBX2, RNF5, PPT2, AGPAT1, GPSM3, EGFL8, PPT2-EGFL8*	intronic		BRST, SPLN	BRST	AP-1,FAC1
rs9803978	1	198645649	42,509	40,577	A>G	0.10	1.01	0.98	1.05	4.2E-01	*PTPRC, PEBP1P3*	intronic	BLD	6 tissues	12 tissues	Bbx
rs41294573	10	6025235	42,234	40,319	G>A	0.06	1.02	0.97	1.06	4.2E-01	*IL2RA, IL15RA, FBXO18*					MIF-1
rs36234015	2	191880573	42,510	40,577	G>A	0.07	1.02	0.98	1.06	4.2E-01	*STAT1, STAT4, LOC100420571*		BLD	BLD, THYM	BLD	
rs2914117	3	159744022	42,478	40,545	A>C	0.19	1.01	0.99	1.04	4.2E-01	*IL12A*			LNG, BLD		9 altered motifs
rs2520361	7	18861140	42,499	40,568	A>G	0.20	0.99	0.97	1.01	4.3E-01	*HDAC9*	intronic			BRN	Pbx3,Pou2f2,YY1

SNP	Chr	Position[a]	N Cases	N Controls	Allele	MAF	OR[b]	95%CI		*p* -value	Gene annotation	dbSNP functional annotation	Promoter histone marks	Enhancer histone marks	DNAse	Motifs changed
rs2980971	18	60074064	42,510	40,577	G>A	0.09	1.01	0.98	1.05	4.3E-01	*ACTBP9, TNFRSF11A, RPL17P44*					GR,STAT
rs2274755	20	44639692	42,497	40,559	C>A	0.14	1.01	0.98	1.04	4.3E-01	*MMP9, SLC12A5, NCOA5, ZNF335, FTLP1, LOC100128028*	intronic	BLD, SKIN, GI	13 tissues	7 tissues	
rs1059702		153284192	42,501	40,564	G>A	0.13	0.99	0.96	1.02	4.3E-01	*HCFC1, IRAK1, MECP2, TMEM187, MIR718, MIR3202-2, MIR3202-1*	missense			MUS,BLD	11 altered motifs
rs56176742	11	69430656	42,493	40,559	G>A	0.09	0.99	0.95	1.02	4.3E-01	*CCND1, ORAOV1, LOC100996515*		LIV	5 tissues	IPSC,GI,LIV	Foxm1
rs1859664	7	18993028	42,252	40,427	G>A	0.23	0.99	0.97	1.01	4.3E-01	*HDAC9, NPM1P13*	intronic		ESDR, HRT	IPSC	Zfx
rs17045435	2	54174258	42,506	40,576	A>G	0.08	0.98	0.95	1.02	4.3E-01	*PSME4*	intronic		BLD, VAS		11 altered motifs
rs3136534	4	123369776	42,500	40,575	A>C	0.36	1.01	0.99	1.03	4.3E-01	*IL2, ADAD1*					DMRT2,Pou2f2,TCF4
rs12073224	1	92200963	42,505	40,571	G>A	0.46	1.01	0.99	1.03	4.3E-01	*TGFBR3*	intronic		14 tissues	GI,GI,SKIN	
rs805319	2	54135429	42,474	40,540	G>C	0.31	0.99	0.97	1.01	4.3E-01	*GPR75, PSME4, GPR75-ASB3*	intronic				13 altered motifs
rs2066793	2	191839464	42,502	40,572	G>A	0.13	1.01	0.98	1.04	4.3E-01	*GLS, STAT1, LOC100420571*	intronic		10 tissues	HRT,LIV	6 altered motifs
rs4484585	7	18718573	42,509	40,574	C>A	0.06	1.02	0.97	1.06	4.3E-01	*HDAC9*	intronic				6 altered motifs
rs3096745	2	204835807	42,506	40,570	T>A	0.24	1.01	0.99	1.03	4.3E-01	*ICOS*			FAT, VAS, GI		5 altered motifs
rs12133753	1	92222089	42,505	40,571	G>A	0.17	1.01	0.98	1.04	4.3E-01	*TGFBR3*	intronic		5 tissues		
rs3773656	3	30722653	42,501	40,572	G>A	0.23	1.01	0.99	1.03	4.3E-01	*TGFBR2, GADL1*	intronic	4 tissues	19 tissues	5 tissues	7 altered motifs
rs6606651	11	69498292	42,436	40,491	A>G	0.09	0.99	0.95	1.02	4.3E-01	*CCND1, FGF19, ORAOV1, LOC100996515*			LIV		4 altered motifs
rs10010469	4	142617431	42,502	40,573	C>A	0.21	0.99	0.97	1.01	4.3E-01	*IL15*	intronic				
rs6696224	1	92193662	42,503	40,571	A>G	0.12	0.99	0.96	1.02	4.3E-01	*TGFBR3*	intronic	BLD	10 tissues	6 tissues	Foxd1,Foxj1
rs177080	7	18741007	42,473	40,526	G>A	0.28	1.01	0.99	1.03	4.3E-01	*HDAC9*	intronic				BRCA1
rs6947529	7	18958942	42,498	40,567	C>A	0.38	0.99	0.97	1.01	4.3E-01	*HDAC9, NPM1P13*	intronic			BRN	DMRT7,Hmbox1,PLZF
rs4739109	8	79749907	42,495	40,559	A>C	0.11	0.99	0.96	1.02	4.3E-01	*IL7*					CEBPG,Dobox4,SIX5
rs9887957	1	92223589	42,494	40,572	G>A	0.16	1.01	0.98	1.04	4.3E-01	*TGFBR3*	intronic		11 tissues	13 tissues	Nkx2,Pou2f2
rs2071232	11	102665669	42,498	40,570	A>G	0.20	0.99	0.97	1.01	4.3E-01	*MMP1, MMP3, MMP10, CSNK1A1P2, WTAPP1, LOC100421658*	intronic	SKIN	GI		NR4A
rs4721732	7	19006961	42,505	40,576	G>C	0.21	0.99	0.97	1.01	4.3E-01	*HDAC9, NPM1P13*	intronic				11 altered motifs
rs6747070	2	9620730	42,508	40,576	G>A	0.08	1.02	0.98	1.05	4.3E-01	*ADAM17, CPSF3, IAH1*	intronic		4 tissues	ESDR	5 altered motifs
rs10281362	7	18843527	42,398	40,436	A>T	0.07	0.98	0.95	1.02	4.3E-01	*HDAC9*	intronic				16 altered motifs
rs7536195	1	23142496	42,505	40,577	G>A	0.10	1.01	0.98	1.05	4.3E-01	*EPHB2, MIR4253*	intronic		8 tissues	MUS	5 altered motifs
rs10459953	17	26127518	42,492	40,567	G>C	0.36	1.01	0.99	1.03	4.3E-01	*NOS2, LOC645754*	5'-UTR			GI,GI,MUS	4 altered motifs
rs4948873	10	44828001	42,502	40,574	A>G	0.43	1.01	0.99	1.03	4.3E-01	*CXCL12, LOC100130539*			ESDR, MUS		Ets,STAT,Znf143
rs11165293	1	92174652	42,508	40,577	G>A	0.11	0.99	0.96	1.02	4.3E-01	*TGFBR3*	intronic		4 tissues		CTCF,Rad21
rs11571291	2	204721132	42,133	40,211	A>G	0.41	0.99	0.97	1.01	4.3E-01	*CTLA4*		BLD	BLD		
rs17881940	17	40476667	42,510	40,575	G>C	0.09	0.99	0.95	1.02	4.3E-01	*STAT3, STAT5A, STAT5B*	intronic		BRST, MUS, LNG	OVRY	NRSF
rs8177656	10	6014984	42,509	40,576	G>A	0.09	1.01	0.98	1.05	4.3E-01	*IL2RA, IL15RA, FBXO18*	intronic		5 tissues	BLD,BLD,SKIN	5 altered motifs
rs1535507	6	43711981	42,507	40,574	G>A	0.16	1.01	0.98	1.04	4.3E-01	*VEGFA, LOC100132242*			10 tissues		NF-kappaB,ZBTB7A
rs284202	1	92241033	42,485	40,559	A>G	0.35	1.01	0.99	1.03	4.3E-01	*TGFBR3*	intronic		12 tissues		
rs648731	10	6468731	42,500	40,568	G>A	0.25	0.99	0.97	1.01	4.3E-01	*PRKCQ*			MUS		AP-1,Arid5a
rs62235148	22	22191458	42,505	40,568	A>G	0.20	1.01	0.99	1.04	4.4E-01	*MAPK1*	intronic	5 tissues	16 tissues	8 tissues	HDAC2,TATA
rs17882046	1	92225054	42,510	40,576	G>A	0.05	1.02	0.97	1.06	4.4E-01	*TGFBR3*	intronic		10 tissues	SKIN	10 altered motifs
rs2209619	1	92127272	42,499	40,565	G>C	0.16	0.99	0.96	1.02	4.4E-01	*TGFBR3, HSP90B3P*					TCF12
rs12127644	1	92219068	42,497	40,546	G>A	0.17	1.01	0.98	1.04	4.4E-01	*TGFBR3*	intronic		4 tissues		
rs10791595	11	102635501	42,478	40,566	A>G	0.36	0.99	0.97	1.01	4.4E-01	*MMP1, MMP8, MMP10, CSNK1A1P2, WTAPP1, LOC100421658*					7 altered motifs
rs519349	10	6564910	42,354	40,395	A>G	0.31	0.99	0.97	1.01	4.4E-01	*PRKCQ*	intronic		BLD		STAT
rs2026432	10	6507603	42,508	40,574	G>A	0.39	0.99	0.97	1.01	4.4E-01	*PRKCQ*	intronic		ESC, KID, BLD	KID,BLD	Pbx-1,TCF4
rs1861089	12	9863968	42,509	40,574	A>G	0.07	1.02	0.98	1.06	4.4E-01	*CD69, NPM1P7, CLEC2D, CLECL1*			7 tissues	BLD	5 altered motifs
rs3212227	5	158742950	42,507	40,577	A>C	0.19	1.01	0.98	1.04	4.4E-01	*IL12B, UBLCP1, LOC285626, RNU4ATAC2P*	3'-UTR				Pou3f2
rs10912295	1	172679935	42,498	40,560	G>C	0.18	0.99	0.96	1.02	4.4E-01	*FASLG, SLC25A38P1*					AFP1,Mef2,Pou3f2
rs2957146	18	60077294	42,505	40,575	A>G	0.09	1.01	0.98	1.05	4.4E-01	*ACTBP9, TNFRSF11A, RPL17P44*				ESDR,IPSC	Pax-5,Zec
rs6604061	1	92328613	42,509	40,576	T>A	0.40	0.99	0.97	1.01	4.4E-01	*TGFBR3*	intronic		6 tissues		5 altered motifs
rs7631671	3	150292771	42,469	40,517	G>A	0.15	1.01	0.98	1.04	4.4E-01	*SERP1, SELT, EIF2A, LOC677762*	intronic		BLD		DMRT1
rs10458360	1	172633975	42,499	40,569	C>G	0.44	0.99	0.97	1.01	4.4E-01	*FASLG*	intronic				
rs6950376	7	18728485	42,466	40,553	A>G	0.10	0.99	0.96	1.02	4.4E-01	*HDAC9*	intronic		ESDR	SKIN	
rs1178163	7	18787563	42,503	40,564	C>A	0.17	1.01	0.98	1.04	4.4E-01	*HDAC9*	intronic				9 altered motifs
rs805338	2	54186536	42,504	40,575	G>A	0.27	0.99	0.97	1.01	4.4E-01	*PSME4*	intronic		BLD, SKIN		4 altered motifs
rs3181096	2	204570092	42,488	40,570	G>A	0.36	1.01	0.99	1.03	4.4E-01	*CD28, LOC100287498*		BLD, THYM	4 tissues	4 tissues	CEBPA,CEBPB,HNF1
rs8052064	16	85945231	42,508	40,577	G>A	0.14	1.01	0.98	1.04	4.4E-01	*IRF8*	synonymous		13 tissues		VDR
rs4845374	1	154426947	42,257	40,242	A>T	0.16	1.01	0.98	1.04	4.4E-01	*IL6R, TDRD10, SHE, PSMD8P1*	intronic		6 tissues		5 altered motifs
rs7614342	3	45940817	42,501	40,572	A>T	0.17	1.01	0.98	1.04	4.4E-01	*CXCR6, CCR9, SDHDP4, FYCO1*	intronic		THYM, BLD		
rs2190273	7	18959046	42,484	40,553	G>A	0.13	1.01	0.98	1.04	4.4E-01	*HDAC9, NPM1P13*	intronic			KID	GR
rs7529421	1	92221425	42,501	40,575	A>C	0.17	1.01	0.98	1.04	4.4E-01	*TGFBR3*	intronic		9 tissues	LNG,MUS	E2F,Smad4,TCF4

SNP	Chr	Position[a]	N Cases	N Controls	Allele	MAF	OR[b]	95%CI		*p* -value	Gene annotation	dbSNP functional annotation	Promoter histone marks	Enhancer histone marks	DNAse	Motifs changed
rs12567680	1	92176044	42,507	40,574	G>A	0.32	0.99	0.97	1.01	4.4E-01	*TGFBR3*	intronic	HRT, BLD	6 tissues	ADRL,BLD,SKIN	AP-1,GR
rs11868709	17	76228571	42,490	40,557	C>A	0.34	1.01	0.99	1.03	4.4E-01	*BIRC5, TK1, AFMID, TMEM235, THA1P, LOC100996291*	intronic	6 tissues	11 tissues	ESC,IPSC	Ets,RREB-1
rs12121039	1	92212347	42,503	40,576	C>G	0.17	1.01	0.98	1.04	4.4E-01	*TGFBR3*	intronic		5 tissues		Irf
rs12808148	11	102733163	42,505	40,574	A>G	0.16	0.99	0.96	1.02	4.4E-01	*MMP3, MMP12, WTAPP1, LOC100288111*					Fox
rs1178123	7	18763844	42,494	40,564	G>A	0.19	1.01	0.98	1.04	4.4E-01	*HDAC9*	intronic				
rs61763396	22	22140647	42,347	40,372	A>C	0.08	0.99	0.95	1.02	4.4E-01	*MAPK1*	intronic			BLD	GR
rs7090512	10	6110829	42,507	40,576	A>G	0.31	0.99	0.97	1.01	4.4E-01	*IL2RA, RBM17, RPL32P23*		BLD, THYM	8 tissues	BLD,BLD,BLD	CCNT2,Mtf1
rs448260	19	6697150	42,495	40,568	C>A	0.39	1.01	0.99	1.03	4.4E-01	*C3, TNFSF14, TRIP10, GPR108*	intronic		LIV		INSM1,ZBTB7A
rs521153	10	6565099	42,508	40,575	A>G	0.31	0.99	0.97	1.01	4.5E-01	*PRKCQ*	intronic		BLD		4 altered motifs
rs6669044	1	92244248	42,502	40,570	A>G	0.16	1.01	0.98	1.04	4.5E-01	*TGFBR3*	intronic		10 tissues		
rs9912773	17	40510534	42,508	40,576	C>G	0.26	1.01	0.99	1.03	4.5E-01	*STAT3, STAT5A, PTRF*	intronic	BLD	13 tissues	BLD	
rs1192929	11	69512674	42,315	40,242	A>G	0.23	1.01	0.99	1.03	4.5E-01	*CCND1, FGF19, ORAOV1, LOC100129779, LOC100996515*			ESDR, ESC, CRVX		SRF,UF1H3BETA
rs16867426	2	182273419	42,496	40,564	A>G	0.25	0.99	0.97	1.01	4.5E-01	*ITGA4*			BLD		
rs7514724	1	92173847	42,506	40,576	G>A	0.11	0.99	0.96	1.02	4.5E-01	*TGFBR3*	intronic		4 tissues		PLZF
rs1178124	7	18764286	42,507	40,575	A>G	0.20	1.01	0.98	1.04	4.5E-01	*HDAC9*	intronic				NF-kappaB,TR4
rs2090916	3	150307263	42,506	40,566	G>A	0.15	1.01	0.98	1.04	4.5E-01	*SERP1, SELT, EIF2A, LOC677762*			THYM		
rs9610216	22	22098687	42,509	40,577	A>G	0.28	1.01	0.99	1.03	4.5E-01	*MAPK1, PPIL2, YPEL1*			4 tissues	10 tissues	5 altered motifs
rs17571088	1	92146890	42,482	40,553	A>G	0.19	0.99	0.96	1.02	4.5E-01	*TGFBR3, HSP90B3P*	3'-UTR				Pax-3,STAT
rs9858487	3	30677559	42,510	40,577	C>G	0.06	0.98	0.94	1.03	4.5E-01	*TGFBR2*	intronic	BLD, GI	14 tissues	8 tissues	FAC1,Foxp1,RREB-1
rs17235675	1	11166073	42,500	40,568	G>A	0.16	1.01	0.98	1.04	4.5E-01	*MTOR, EXOSC10, SRM, MTOR-AS1*					4 altered motifs
rs3024685	16	27376910	42,509	40,573	A>G	0.40	0.99	0.97	1.01	4.5E-01	*IL4R, IL21R*		BLD, LNG	16 tissues	17 tissues	AP-1,NF-I,PU.1
rs1148458	1	12233061	42,501	40,571	G>A	0.07	0.99	0.95	1.02	4.5E-01	*TNFRSF8, TNFRSF1B, LOC390998, MIR4632*	intronic	BLD, FAT, GI	12 tissues	4 tissues	PLAG1,Znf143
rs2306886	1	92200111	42,503	40,564	G>A	0.46	1.01	0.99	1.03	4.5E-01	*TGFBR3*	intronic		10 tissues		
rs2717536	8	79701876	42,267	40,340	G>A	0.09	0.99	0.95	1.02	4.5E-01	*IL7, PRKRIRP7*	intronic		FAT, MUS, SKIN		8 altered motifs
rs2296621	1	92163786	42,508	40,576	C>A	0.20	0.99	0.97	1.02	4.5E-01	*TGFBR3*	intronic	BLD	12 tissues	OVRY,CRVX	9 altered motifs
rs4733550	8	128763162	42,503	40,570	T>A	0.50	1.01	0.99	1.03	4.5E-01	*MYC, MIR1204*			BLD	BLD	
rs2282055	9	5455732	42,498	40,571	A>C	0.26	0.99	0.97	1.01	4.5E-01	*CD274, PLGRKT, LOC100419687*	intronic	5 tissues	9 tissues	PLCNT,BLD	
rs2588621	7	18842091	42,477	40,541	T>A	0.31	1.01	0.99	1.03	4.5E-01	*HDAC9*	intronic				Mef2
rs11466579	1	92199920	42,510	40,577	G>A	0.10	1.01	0.98	1.05	4.5E-01	*TGFBR3*	intronic		9 tissues	HRT	Pou5f1
rs17720604	15	40245926	42,510	40,577	G>A	0.11	0.99	0.96	1.02	4.5E-01	*GPR176, EIF2AK4, H3F3AP1, LOC100505534*	intronic		SKIN, PLCNT, BRST	7 tissues	10 altered motifs
rs10905644	10	6073929	42,295	40,367	A>G	0.05	0.98	0.94	1.03	4.5E-01	*IL2RA, RPL32P23*	intronic				Pou2f2,STAT,Sox
rs859665	1	172664442	42,356	40,443	A>G	0.34	1.01	0.99	1.03	4.5E-01	*FASLG*			BLD		
rs12030948	1	67701765	42,501	40,567	C>A	0.35	1.01	0.99	1.03	4.5E-01	*IL23R, LOC100130497*	intronic		GI		
rs10114060	9	5461729	42,441	40,477	G>A	0.26	0.99	0.97	1.01	4.5E-01	*CD274, PLGRKT, PDCD1LG2, LOC100419687*	intronic				GZF1,Gm397
rs1409082	13	43228324	42,507	40,575	G>A	0.11	0.99	0.96	1.02	4.6E-01	*TNFSF11*			4 tissues		AP-2,NF-kappaB
rs2254859	7	18648536	42,504	40,576	G>A	0.22	0.99	0.97	1.01	4.6E-01	*HDAC9, LOC100419901*	intronic				4 altered motifs
rs11117425	16	85972271	42,503	40,577	G>A	0.33	0.99	0.97	1.01	4.6E-01	*IRF8*			BLD, SPLN	BLD	NRSF
rs4764590	12	6532350	42,501	40,574	A>C	0.37	0.99	0.97	1.01	4.6E-01	*CD27, LTBR, SCNN1A, VAMP1, TAPBPL, RPL31P10, SRP14P1, CD27-AS1*			SKIN	BLD	Hoxa9,Mef2,TFII-I
rs3018352	18	60074865	42,507	40,575	C>A	0.09	1.01	0.98	1.05	4.6E-01	*ACTBP9, TNFRSF11A, RPL17P44*			BLD		7 altered motifs
rs12121065	1	92212467	42,504	40,573	C>A	0.17	1.01	0.98	1.04	4.6E-01	*TGFBR3*	intronic		6 tissues	5 tissues	GATA,Mef2
rs2280233	2	191850566	42,498	40,572	A>G	0.48	0.99	0.97	1.01	4.6E-01	*GLS, STAT1, STAT4, LOC100420571*	intronic		4 tissues	HRT	
rs11582136	1	92199184	42,510	40,577	A>G	0.10	1.01	0.98	1.05	4.6E-01	*TGFBR3*	intronic		13 tissues	KID,MUS,MUS	
rs525891	1	12249643	42,502	40,575	T>A	0.24	0.99	0.97	1.01	4.6E-01	*TNFRSF8, TNFRSF1B, VPS13D, LOC390998, MIR4632*	intronic		5 tissues		6 altered motifs
rs66488311	1	11281053	42,158	40,068	T>A	0.29	1.01	0.99	1.03	4.6E-01	*MTOR, ANGPTL7, RPL39P6*	intronic				10 altered motifs
rs3212263	11	118216423	42,499	40,574	A>G	0.30	1.01	0.99	1.03	4.6E-01	*CD3D, CD3E, CD3G, UBE4A, LOC100131626*	intronic	BLD, GI, THYM	BLD	BLD,THYM	4 altered motifs
rs17432462	7	18548613	42,506	40,566	A>G	0.37	0.99	0.97	1.01	4.6E-01	*HDAC9*	intronic	13 tissues	11 tissues	33 tissues	DMRT3,DMRT7
rs7647948	3	30760602	42,503	40,575	G>A	0.36	1.01	0.99	1.03	4.6E-01	*TGFBR2, GADL1*			BLD, LIV		ERalpha-a,Zfx
rs624016	10	6486611	42,451	40,537	T>A	0.33	1.01	0.99	1.03	4.6E-01	*PRKCQ*	intronic				5 altered motifs
rs3917148	14	76446521	42,505	40,576	A>C	0.07	0.99	0.95	1.02	4.6E-01	*TGFB3, TTLL5, IFT43, LOC100506576*	intronic	16 tissues	8 tissues	11 tissues	PU.1,SRF
rs4938499	11	118162496	42,502	40,575	A>G	0.33	1.01	0.99	1.03	4.6E-01	*CD3D, CD3E, MPZL2, MPZL3*		FAT, BLD, CRVX	BRST, SKIN, GI	8 tissues	Pax-4
rs11165441	1	92224347	42,504	40,573	G>A	0.16	1.01	0.98	1.04	4.6E-01	*TGFBR3*	intronic		12 tissues		Bcl6b
rs9610375	22	22187093	42,494	40,569	C>A	0.46	0.99	0.97	1.01	4.6E-01	*MAPK1*	intronic	FAT	19 tissues	21 tissues	RREB-1,Zbtb3
rs12041613	1	172639582	42,505	40,571	A>G	0.46	1.01	0.99	1.03	4.6E-01	*FASLG*		BLD	5 tissues	BLD,BLD	Pou3f2,Pou5f1
rs7103514	11	117820561	42,507	40,576	A>G	0.08	1.01	0.98	1.05	4.6E-01	*IL10RA, TMPRSS13*		BLD	BLD, THYM	BLD	11 altered motifs
rs17347159	7	18394098	42,505	40,574	C>A	0.18	0.99	0.97	1.02	4.6E-01	*HDAC9*	intronic		5 tissues	BLD,GI	Gm397,Maf
rs7042084	9	5458035	42,495	40,563	C>A	0.48	0.99	0.97	1.01	4.6E-01	*CD274, PLGRKT, LOC100419687*	intronic		BLD, VAS	BLD	AIRE,GATA
rs2583764	8	79663800	42,040	40,047	A>G	0.16	0.99	0.96	1.02	4.6E-01	*IL7, ZC2HC1A, PRKRIRP7*	intronic				GR
rs3729912	22	22160384	42,510	40,577	A>G	0.09	0.99	0.95	1.02	4.6E-01	*MAPK1*	intronic			HRT,BLD	5 altered motifs

SNP	Chr	Position[a]	N Cases	N Controls	Allele	MAF	OR[b]	95%CI		*p* -value	Gene annotation	dbSNP functional annotation	Promoter histone marks	Enhancer histone marks	DNAse	Motifs changed
rs489508	15	40245779	42,501	40,570	G>A	0.41	1.01	0.99	1.03	4.6E-01	*GPR176, EIF2AK4, H3F3AP1, LOC100505534*	intronic		4 tissues	SKIN,PLCNT	5 altered motifs
rs548580	1	12249770	42,498	40,574	G>A	0.24	0.99	0.97	1.01	4.6E-01	*TNFRSF8, TNFRSF1B, VPS13D, LOC390998, MIR4632*	intronic		5 tissues		ATF3,p53
rs3213094	5	158750769	42,509	40,575	G>A	0.19	1.01	0.98	1.04	4.6E-01	*IL12B, UBLCP1, LOC285626, RNU4ATAC2P*	intronic				Sox,Zec
rs62626314	10	6109449	42,509	40,576	C>A	0.06	1.02	0.97	1.06	4.6E-01	*IL2RA, RBM17, RPL32P23*			6 tissues	BLD	5 altered motifs
rs2588627	7	18829392	42,505	40,572	A>C	0.17	1.01	0.98	1.04	4.6E-01	*HDAC9*	intronic				MZF1::1-4,p300
rs2853697	5	158743403	42,510	40,577	A>C	0.20	1.01	0.98	1.04	4.6E-01	*IL12B, UBLCP1, LOC285626, RNU4ATAC2P*	intronic		IPSC, SKIN		THAP1
rs3024547	16	27354361	42,508	40,575	G>A	0.14	0.99	0.96	1.02	4.6E-01	*IL4R*	intronic			BLD	
rs976881	1	12233754	42,477	40,544	G>A	0.31	1.01	0.99	1.03	4.6E-01	*TNFRSF8, TNFRSF1B, LOC390998, MIR4632*	intronic	4 tissues	13 tissues	13 tissues	Ncx,Nkx1-1
rs3024543	16	27353230	42,507	40,576	G>A	0.14	0.99	0.96	1.02	4.6E-01	*IL4R*	intronic		BRST, BLD	BLD	7 altered motifs
rs10905743	10	6133365	42,508	40,574	C>A	0.08	0.99	0.95	1.02	4.7E-01	*IL2RA, RBM17, RPL32P23*	intronic			LIV	11 altered motifs
rs1052238	1	198634625	42,450	40,516	G>A	0.48	0.99	0.97	1.01	4.7E-01	*PTPRC, PEBP1P3*	intronic			BLD,BLD,PLCNT	GR,SIX5
rs6993386	8	79654145	42,500	40,574	A>G	0.32	1.01	0.99	1.03	4.7E-01	*IL7, ZC2HC1A, PRKRIRP7*	intronic				4 altered motifs
rs17576	20	44640225	42,478	40,543	A>G	0.36	1.01	0.99	1.03	4.7E-01	*MMP9, SLC12A5, NCOA5, ZNF335, FTLP1, LOC100128028*	missense	5 tissues	12 tissues	ESC	Pax-4
rs3850347	2	54166321	42,505	40,566	G>A	0.07	0.99	0.95	1.02	4.7E-01	*PSME4*	intronic		BLD, MUS	MUS	CEBPA,CEBPB
rs880364	16	85959165	42,482	40,562	A>G	0.25	1.01	0.99	1.03	4.7E-01	*IRF8*			4 tissues	PLCNT	
rs3767574	1	92169004	42,504	40,574	G>A	0.23	0.99	0.97	1.02	4.7E-01	*TGFBR3*	intronic		FAT, STRM, SKIN	MUS	AP-1,YY1
rs2980968	18	60068125	42,095	40,074	G>A	0.08	1.01	0.98	1.05	4.7E-01	*ACTBP9, TNFRSF11A, RPL17P44*			BLD, LIV		Pax-6
rs4654828	1	23283076	42,506	40,573	G>A	0.31	0.99	0.97	1.01	4.7E-01	*EPHB2, LACTBL1*					4 altered motifs
rs12722497	10	6095928	42,505	40,577	C>A	0.09	0.99	0.95	1.02	4.7E-01	*IL2RA, RBM17, RPL32P23*	intronic	BLD, GI, LIV	12 tissues	10 tissues	BDP1,En-1,Maf
rs9906835	17	26089374	42,479	40,549	A>G	0.40	0.99	0.97	1.01	4.7E-01	*NOS2, LOC645754*	intronic				CDP,NF-kappaB
rs529650	3	119295128	42,186	40,188	G>A	0.39	0.99	0.97	1.01	4.7E-01	*ADPRH, CD80, CSRP2P, PLA1A*					8 altered motifs
rs3024632	16	27366296	42,487	40,549	A>G	0.09	0.99	0.95	1.02	4.7E-01	*IL4R, IL21R*	intronic				CCNT2,GATA,TAL1
rs6540512	1	206813737	42,509	40,575	G>A	0.16	1.01	0.98	1.04	4.7E-01	*EIF2D, DYRK3, MAPKAPK2*	intronic				Myc,Pou5f1,SREBP
rs587325	7	18335683	42,501	40,570	G>A	0.16	0.99	0.96	1.02	4.7E-01	*HDAC9*	intronic	BLD	BLD	BLD,BLD,HRT	CEBPG,Foxj1,Isl2
rs805426	2	54130532	42,504	40,573	G>A	0.30	0.99	0.97	1.01	4.7E-01	*GPR75, PSME4, GPR75-ASB3*	intronic		BRN		STAT
rs4845980	1	11243138	42,367	40,374	A>G	0.05	0.98	0.94	1.03	4.7E-01	*MTOR, ANGPTL7, RPL39P6, MTOR-AS1*	intronic		ESDR	BRN,BRN	AP-1,SMC3
rs688391	10	6489652	42,497	40,574	A>G	0.33	0.99	0.97	1.01	4.7E-01	*PRKCQ*	intronic		THYM, MUS	THYM	GR,NF-I,Roaz
rs6684753	1	92330016	42,506	40,573	C>G	0.40	0.99	0.97	1.01	4.7E-01	*TGFBR3*	intronic	8 tissues	17 tissues	13 tissues	AP-1,PU.1,STAT
rs13246896	7	18843694	42,404	40,454	A>T	0.25	1.01	0.99	1.03	4.7E-01	*HDAC9*	intronic		ESDR, BRST, HRT		4 altered motifs
rs3918249	20	44638136	42,491	40,561	A>G	0.36	1.01	0.99	1.03	4.7E-01	*MMP9, SLC12A5, ZNF335, FTLP1, LOC100128028*	intronic	BLD, SKIN	6 tissues	BLD,BLD	4 altered motifs
rs10123377	9	5461394	42,490	40,564	A>G	0.50	0.99	0.97	1.01	4.7E-01	*CD274, PLGRKT, PDCD1LG2, LOC100419687*	intronic				Egr-1,Pou3f3
rs1805012	16	27373964	42,505	40,564	A>G	0.11	0.99	0.96	1.02	4.7E-01	*IL4R, IL21R*	missense		5 tissues	GI	Bach2,Pax-2,RXRA
rs9340	22	22115353	42,495	40,569	G>A	0.41	0.99	0.97	1.01	4.7E-01	*MAPK1, YPEL1*	3'-UTR	LNG	7 tissues	4 tissues	4 altered motifs
rs12141338	1	92161991	42,509	40,576	A>C	0.20	0.99	0.97	1.02	4.7E-01	*TGFBR3*	intronic		4 tissues	5 tissues	
rs12903784	15	40304407	42,505	40,570	A>C	0.36	0.99	0.97	1.01	4.7E-01	*SRP14, EIF2AK4, SRP14-AS1*	intronic		BRN		HNF4
rs12722561	10	6069893	42,508	40,577	G>A	0.16	1.01	0.98	1.04	4.8E-01	*IL2RA, IL15RA, RPL32P23*	intronic		BLD		
rs2038931	1	92174415	42,505	40,567	G>A	0.11	0.99	0.96	1.02	4.8E-01	*TGFBR3*	intronic		ADRL, HRT, BLD	BLD	8 altered motifs
rs2056576	7	22761202	42,502	40,575	G>A	0.31	1.01	0.99	1.03	4.8E-01	*IL6, LOC541472*			10 tissues	ESDR,MUS,PLCNT	CTCF
rs11725823	4	123425785	42,491	40,569	G>C	0.36	1.01	0.99	1.03	4.8E-01	*IL2*			BLD		Nkx3,Pou2f2
rs3917211	14	76426723	42,508	40,575	A>G	0.23	0.99	0.97	1.02	4.8E-01	*TGFB3, TTLL5, IFT43*	intronic	BLD	BLD, SKIN, MUS	9 tissues	4 altered motifs
rs2949810	2	54153341	42,508	40,574	A>G	0.28	0.99	0.97	1.01	4.8E-01	*PSME4*	intronic				GR
rs2372214	3	30766438	42,486	40,558	A>G	0.36	1.01	0.99	1.03	4.8E-01	*TGFBR2, GADL1*					Arid5b,Ik-2,Pou2f2
rs2009112	1	218553529	42,496	40,560	G>A	0.41	1.01	0.99	1.03	4.8E-01	*TGFB2, RRP15, RPS26P17, LOC728463*	intronic	BRN, LNG, SKIN	19 tissues	4 tissues	HDAC2,Hbp1
rs10272709	7	18934838	42,502	40,575	G>A	0.13	0.99	0.96	1.02	4.8E-01	*HDAC9*	intronic			THYM	BDP1,YY1
rs6470563	8	128708570	42,500	40,574	A>C	0.11	0.99	0.96	1.02	4.8E-01	*MYC*			BLD		MZF1::1-4,Pou5f1,ZNF263
rs805447	2	54177130	42,467	40,510	C>A	0.27	0.99	0.97	1.01	4.8E-01	*PSME4*	intronic		10 tissues		TCF12
rs13003194	2	204848818	42,508	40,574	C>A	0.09	0.99	0.95	1.02	4.8E-01	*ICOS*				BLD	5 altered motifs
rs3754021	1	92158751	42,509	40,574	A>G	0.20	0.99	0.97	1.02	4.8E-01	*TGFBR3, HSP90B3P*	intronic		6 tissues		Hoxb9,Hoxd10,TATA
rs6809408	3	3122827	42,502	40,575	C>A	0.36	0.99	0.97	1.01	4.8E-01	*IL5RA, TRNT1, CNTN4*	intronic		ESC, IPSC, BLD		
rs3809758	17	40471980	42,504	40,574	G>A	0.19	1.01	0.98	1.03	4.8E-01	*STAT3, STAT5A, STAT5B*	intronic	BLD, SKIN, VAS	14 tissues	25 tissues	Esr2
rs2695027	7	18631680	42,504	40,573	G>A	0.39	1.01	0.99	1.03	4.8E-01	*HDAC9, LOC100419901*	intronic				6 altered motifs
rs3917210	14	76427780	42,504	40,571	A>T	0.23	0.99	0.97	1.02	4.8E-01	*TGFB3, TTLL5, IFT43*	intronic		BLD, SKIN		8 altered motifs
rs2249817	7	18896011	42,500	40,568	A>G	0.45	1.01	0.99	1.03	4.8E-01	*HDAC9*	intronic		5 tissues		Arid5b,Mef2
rs12042254	1	92114531	42,510	40,573	G>A	0.08	1.01	0.98	1.05	4.8E-01	*TGFBR3, HSP90B3P, RPL39P13*			FAT		6 altered motifs
rs756790	7	18714381	42,476	40,524	G>A	0.19	1.01	0.98	1.03	4.8E-01	*HDAC9*	intronic				5 altered motifs
rs12572136	10	6077191	42,503	40,573	C>G	0.08	0.99	0.95	1.02	4.8E-01	*IL2RA, RPL32P23*	intronic		6 tissues		
rs17339927	12	88929348	41,235	39,165	A>C	0.11	0.99	0.96	1.02	4.8E-01	*KITLG*	intronic				
rs12913170	15	40376208	42,508	40,574	G>A	0.17	1.01	0.98	1.04	4.8E-01	*SRP14, BMF, EIF2AK4, SRP14-AS1*			9 tissues	4 tissues	AP-1

SNP	Chr	Position[a]	N Cases	N Controls	Allele	MAF	OR[b]	95%CI		*p* -value	Gene annotation	dbSNP functional annotation	Promoter histone marks	Enhancer histone marks	DNAse	Motifs changed
rs4810482	20	44634550	42,507	40,576	A>G	0.37	1.01	0.99	1.03	4.8E-01	*MMP9, SLC12A5, ZNF335, FTLP1, LOC100128028*		BLD	9 tissues	ESC,BLD	AP-1
rs2158495	7	19021592	42,507	40,576	G>A	0.17	0.99	0.96	1.02	4.8E-01	*HDAC9, NPM1P13*	intronic				5 altered motifs
rs6766008	3	30721628	42,386	40,466	C>A	0.23	0.99	0.97	1.02	4.8E-01	*TGFBR2, GADL1*	intronic		11 tissues		18 altered motifs
rs10250414	7	18979194	42,486	40,565	G>A	0.41	1.01	0.99	1.03	4.8E-01	*HDAC9, NPM1P13*	intronic				9 altered motifs
rs913060	1	92150058	42,510	40,576	G>A	0.38	1.01	0.99	1.03	4.8E-01	*TGFBR3, HSP90B3P*	intronic		20 tissues	MUS,MUS,SKIN	CTCF,Nanog
rs2506145	10	33471130	42,503	40,571	A>G	0.12	1.01	0.98	1.04	4.8E-01	*NRP1*	intronic		SKIN, LNG		CTCF,Zbtb12
rs3024560	16	27356667	42,493	40,556	A>C	0.35	0.99	0.97	1.01	4.9E-01	*IL4R*	intronic		BLD, MUS	BLD,PLCNT	4 altered motifs
rs403038	16	85949071	42,489	40,571	G>A	0.15	1.01	0.98	1.04	4.9E-01	*IRF8*	intronic		7 tissues		4 altered motifs
rs2066960	5	131994435	42,502	40,577	C>A	0.11	1.01	0.98	1.04	4.9E-01	*IL4, IL13, RAD50, KIF3A*	intronic	LIV	4 tissues		SRF
rs2240419	7	18975189	42,484	40,551	G>A	0.25	0.99	0.97	1.01	4.9E-01	*HDAC9, NPM1P13*	intronic				GATA,NF-I,Pax-5
rs9512989	13	28606269	42,499	40,565	A>C	0.21	1.01	0.98	1.03	4.9E-01	*FLT3, PRHOXNB, LOC100420919*	intronic	BLD	BLD, MUS	5 tissues	7 altered motifs
rs12601611	17	40497828	41,772	39,945	G>A	0.27	1.01	0.99	1.03	4.9E-01	*STAT3, STAT5A*	intronic		8 tissues	7 tissues	
rs13010343	2	191843445	42,509	40,576	G>A	0.13	1.01	0.98	1.04	4.9E-01	*GLS, STAT1, LOC100420571*	intronic		11 tissues	4 tissues	Evi-1
rs7421861	2	242795350	42,476	40,556	A>G	0.36	0.99	0.97	1.01	4.9E-01	*PDCD1, NEU4, CXXC11, LOC285095*	intronic		IPSC, SKIN, MUS		9 altered motifs
rs2834179	21	34679731	42,507	40,577	G>A	0.35	0.99	0.97	1.01	4.9E-01	*IFNAR1, IFNAR2, IL10RB, USF1P1, IL10RB-AS1*			4 tissues		
rs306587	10	30722908	41,487	39,517	C>G	0.40	0.99	0.97	1.01	4.9E-01	*CCND3P, MAP3K8*		24 tissues		52 tissues	17 altered motifs
rs2859228	1	172613241	42,503	40,573	A>G	0.48	0.99	0.97	1.01	4.9E-01	*FASLG, SUCO*		BLD	10 tissues	7 tissues	CDP,TR4
rs2976230	17	3631241	42,505	40,575	A>G	0.21	0.99	0.97	1.02	4.9E-01	*ITGAE, P2RX5, GSG2, P2RX5-TAX1BP3*	missense		BLD, THYM	BLD	RXRA
rs12985234	19	8026549	42,486	40,564	A>G	0.29	0.99	0.97	1.01	4.9E-01	*ELAVL1, MAP2K7, SNAPC2, TIMM44, CTXN1, TGFBR3L*	3'-UTR		5 tissues	4 tissues	
rs2236416	20	44640575	42,493	40,564	A>G	0.14	1.01	0.98	1.04	4.9E-01	*MMP9, SLC12A5, NCOA5, ZNF335, FTLP1, LOC100128028*	intronic	BLD, SKIN, GI	14 tissues	33 tissues	NF-Y
rs10204137	2	102968212	42,492	40,553	A>G	0.38	1.01	0.99	1.03	4.9E-01	*IL18R1, IL1RL1*	missense			THYM	WT1
rs8192282	1	154401679	42,509	40,575	G>A	0.16	1.01	0.98	1.04	4.9E-01	*IL6R, PSMD8P1*					
rs3808847	9	5509091	42,509	40,577	G>A	0.06	1.01	0.97	1.06	4.9E-01	*CD274, PDCD1LG2*		MUS	14 tissues	5 tissues	NRSF,Nkx3
rs41295087	10	6119384	42,446	40,494	G>A	0.11	0.99	0.96	1.02	4.9E-01	*IL2RA, RBM17, RPL32P23*					9 altered motifs
rs3918261	20	44643592	42,507	40,568	A>G	0.14	1.01	0.98	1.04	4.9E-01	*MMP9, SLC12A5, NCOA5, ZNF335, FTLP1, LOC100128028*	intronic		9 tissues	BLD,BRST	Foxo,GR
rs12523963	6	43771002	42,434	40,495	G>A	0.30	1.01	0.99	1.03	4.9E-01	*VEGFA*			15 tissues	11 tissues	AP-1
rs3181225	5	158740623	42,504	40,573	G>A	0.20	1.01	0.98	1.03	4.9E-01	*IL12B, UBLCP1, LOC285626, RNU4ATAC2P*					RAR,RORalpha1,RXRA
rs17140423	7	19007884	42,505	40,574	A>G	0.30	0.99	0.97	1.01	4.9E-01	*HDAC9, NPM1P13*	intronic				Arid5a,RP58,TAL1
rs9289186	3	121740304	42,507	40,576	G>A	0.10	0.99	0.96	1.02	4.9E-01	*CD86, ILDR1*	intronic	6 tissues	5 tissues	GI,BLD	E2A,Myf
rs805408	2	54120025	42,497	40,570	T>A	0.30	0.99	0.97	1.01	4.9E-01	*GPR75, PSME4, GPR75-ASB3, MIR3682*	missense				GR,TCF4,TFE
rs12075255	1	206961628	42,510	40,577	G>A	0.15	0.99	0.96	1.02	4.9E-01	*IL10, IL19*			BLD		INSM1,p53
rs2474737	10	33464928	42,495	40,569	G>A	0.15	0.99	0.96	1.02	4.9E-01	*NRP1*			4 tissues	SKIN	Cphx,Evi-1,GATA
rs8074524	17	40469598	42,503	40,576	G>A	0.19	1.01	0.98	1.03	4.9E-01	*STAT3, STAT5A, STAT5B*	intronic		10 tissues	11 tissues	Pax-5
rs8047999	16	85947527	42,428	40,520	C>G	0.45	1.01	0.99	1.03	4.9E-01	*IRF8*	intronic		11 tissues	6 tissues	
rs470221	11	102665270	42,500	40,571	G>A	0.18	0.99	0.97	1.02	4.9E-01	*MMP1, MMP3, MMP10, CSNK1A1P2, WTAPP1, LOC100421658*	intronic	SKIN	4 tissues	24 tissues	CTCF,Rad21
rs11505418	7	18993249	42,444	40,530	A>G	0.46	1.01	0.99	1.03	4.9E-01	*HDAC9, NPM1P13*	intronic		ESDR, HRT		HNF4
rs2157847	7	18642019	42,475	40,544	G>A	0.06	1.01	0.97	1.06	4.9E-01	*HDAC9, LOC100419901*	intronic				Foxa,TCF4
rs305088	16	85981601	42,483	40,563	G>A	0.32	0.99	0.97	1.01	4.9E-01	*IRF8*		7 tissues	6 tissues	12 tissues	4 altered motifs
rs6948023	7	18841289	42,425	40,485	G>A	0.31	1.01	0.99	1.03	4.9E-01	*HDAC9*	intronic	FAT	FAT		4 altered motifs
rs9282743	4	142654518	42,497	40,560	C>A	0.06	0.99	0.95	1.03	4.9E-01	*IL15*	3'-UTR				6 altered motifs
rs12817967	12	6545994	42,493	40,569	A>C	0.43	0.99	0.97	1.01	4.9E-01	*CD27, LTBR, VAMP1, TAPBPL, PKP2P1, RPL31P10, SRP14P1, CD27-AS1*					9 altered motifs
rs805365	2	54170035	42,504	40,577	C>A	0.31	0.99	0.97	1.01	4.9E-01	*PSME4*	intronic		BLD, STRM, GI		Irf,Pax-5,RXRA
rs16944	2	113594867	42,498	40,564	G>A	0.34	0.99	0.97	1.01	4.9E-01	*IL1B, LOC100128413*		4 tissues	13 tissues	SKIN	Maf
rs10876966	12	57543572	42,475	40,556	G>A	0.23	1.01	0.98	1.03	4.9E-01	*LRP1, STAT6, MIR1228*	intronic			13 tissues	NF-I,p300
rs7812296	7	19036738	42,505	40,572	A>G	0.45	1.01	0.99	1.03	5.0E-01	*HDAC9, NPM1P13*	3'-UTR		4 tissues		21 altered motifs
rs805425	2	54129482	42,492	40,561	A>G	0.31	0.99	0.97	1.01	5.0E-01	*GPR75, PSME4, GPR75-ASB3*	intronic				Evi-1,PEBP
rs6783320	3	159751822	42,507	40,575	G>A	0.18	0.99	0.97	1.02	5.0E-01	*IL12A*			BLD		5 altered motifs
rs6567280	18	60063622	42,492	40,572	A>G	0.41	0.99	0.97	1.01	5.0E-01	*ACTBP9, TNFRSF11A, RPL17P44*					5 altered motifs
rs3824448	9	5508986	42,509	40,575	G>C	0.06	1.01	0.97	1.06	5.0E-01	*CD274, PDCD1LG2*		MUS	14 tissues	SKIN,SKIN,BLD	NRSF,Pou2f2,Sin3Ak-20
rs7796939	7	18954983	42,507	40,576	A>G	0.47	0.99	0.97	1.01	5.0E-01	*HDAC9, NPM1P13*	intronic		SKIN		Msx-1
rs17610618	1	198609908	40,245	38,352	G>A	0.22	0.99	0.97	1.02	5.0E-01	*PTPRC, PEBP1P3*	intronic	6 tissues		BLD	5 altered motifs
rs9268969	6	32434349	42,405	40,402	G>A	0.31	0.99	0.97	1.01	5.0E-01	*HLA-DRA, HLA-DRB9*					Hsf,STAT
rs4625363	10	6072504	42,508	40,572	A>G	0.16	1.01	0.98	1.04	5.0E-01	*IL2RA, RPL32P23*	intronic				Rad21
rs3804793	3	3148245	42,505	40,565	A>C	0.13	1.01	0.98	1.04	5.0E-01	*IL5RA, TRNT1, CRBN, CNTN4*	intronic		ESDR		12 altered motifs
rs2810891	1	92154088	42,499	40,565	G>A	0.11	1.01	0.98	1.04	5.0E-01	*TGFBR3, HSP90B3P*	intronic		10 tissues		Nkx3,SREBP

SNP	Chr	Position[a]	N Cases	N Controls	Allele	MAF	OR[b]	95%CI		*p* -value	Gene annotation	dbSNP functional annotation	Promoter histone marks	Enhancer histone marks	DNAse	Motifs changed
rs11713419	3	3150267	42,500	40,571	A>G	0.16	0.99	0.96	1.02	5.0E-01	*IL5RA, TRNT1, CRBN*	5'-UTR	GI	4 tissues		Zfp691
rs7078614	10	6075831	42,484	40,534	C>A	0.40	1.01	0.99	1.03	5.0E-01	*IL2RA, RPL32P23*	intronic	STRM	6 tissues	4 tissues	Foxp3,GATA
rs4001107	7	18849893	42,491	40,568	A>C	0.28	1.01	0.99	1.03	5.0E-01	*HDAC9*	intronic				BHLHE40,Pou2f2,TFE
rs6809777	3	30672362	42,503	40,560	G>A	0.28	0.99	0.97	1.01	5.0E-01	*TGFBR2*	intronic	BLD	18 tissues	7 tissues	TCF11::MafG
rs3087243	2	204738919	42,503	40,568	G>A	0.43	0.99	0.97	1.01	5.0E-01	*CTLA4*			4 tissues	5 tissues	
rs11165377	1	92200634	42,501	40,571	G>A	0.27	1.01	0.99	1.03	5.0E-01	*TGFBR3*	intronic		12 tissues	BLD,ADRL,GI	5 altered motifs
rs1071676	2	113587433	42,503	40,571	G>C	0.25	0.99	0.97	1.02	5.0E-01	*IL1A, IL1B, LOC100128413*	3'-UTR			BLD	14 altered motifs
rs875890	3	45945287	42,468	40,555	T>A	0.27	0.99	0.97	1.01	5.0E-01	*CXCR6, CCR9, SDHDP4, FYCO1*			THYM, BLD		NRSF,Zbtb3
rs7087802	10	6551732	42,508	40,576	A>G	0.50	0.99	0.97	1.01	5.0E-01	*PRKCQ*	intronic				
rs17131540	1	92191378	42,473	40,558	G>A	0.12	0.99	0.96	1.02	5.0E-01	*TGFBR3*	intronic	BONE	15 tissues	12 tissues	KAP1,Smad
rs12069176	1	92200782	42,499	40,572	A>G	0.31	0.99	0.97	1.01	5.0E-01	*TGFBR3*	intronic		12 tissues	BLD,GI	Cdc5
rs12208813	6	42025318	42,503	40,574	G>A	0.11	1.01	0.98	1.04	5.0E-01	*CCND3, TAF8, C6orf132*	intronic				7 altered motifs
rs11256416	10	6075359	42,480	40,514	G>A	0.20	1.01	0.98	1.03	5.0E-01	*IL2RA, RPL32P23*	intronic		ESDR		Bcl6b,Mef2,RXRA
rs2223286	1	169665632	42,507	40,575	A>G	0.30	0.99	0.97	1.01	5.0E-01	*SELE, SELL*	intronic	BLD	4 tissues	9 tissues	8 altered motifs
rs10518261	19	40272816	42,510	40,577	A>G	0.19	0.99	0.97	1.02	5.0E-01	*CLC, DYRK1B, LEUTX*	intronic				
rs698856	2	54122956	41,712	39,777	G>A	0.36	1.01	0.99	1.03	5.0E-01	*GPR75, PSME4, GPR75-ASB3, MIR3682*	intronic				Gcm1,RFX5
rs1126159	19	40174119	42,498	40,570	G>A	0.33	0.99	0.97	1.01	5.0E-01	*CLC, LGALS14, LGALS16, LGALS17A, LOC100129935, RPS29P27*	intronic				
rs681071	10	6471343	42,505	40,567	G>A	0.06	0.99	0.94	1.03	5.0E-01	*PRKCQ*	intronic			THYM,MUS	16 altered motifs
rs805427	2	54131014	42,500	40,552	A>T	0.30	0.99	0.97	1.01	5.0E-01	*GPR75, PSME4, GPR75-ASB3*	intronic				10 altered motifs
rs2359994	14	76429369	42,498	40,570	A>C	0.16	0.99	0.96	1.02	5.0E-01	*TGFB3, TTLL5, IFT43*	intronic				
rs3027935		153304468	42,502	40,571	G>A	0.07	1.01	0.97	1.05	5.0E-01	*IRAK1, MECP2, MIR718*	intronic		8 tissues	SKIN,MUS,SKIN	BDP1,NRSF
rs3861950	1	173156292	42,485	40,561	A>G	0.32	1.01	0.99	1.03	5.0E-01	*TNFSF4, GOT2P2, LOC100506023*	intronic		BRST, SKIN		
rs2243935	11	117824960	42,487	40,543	G>A	0.29	0.99	0.97	1.01	5.0E-01	*IL10RA, TMPRSS13*			BLD, THYM	BLD,BLD	DBP,SRF
rs3918270	20	44645339	42,499	40,548	G>A	0.14	1.01	0.98	1.04	5.0E-01	*MMP9, SLC12A5, NCOA5, ZNF335, FTLP1, LOC100128028*	intronic		LIV		5 altered motifs
rs8179181	19	41838206	42,487	40,561	G>A	0.25	0.99	0.97	1.02	5.1E-01	*TGFB1, HNRNPUL1, B9D2, CCDC97, TMEM91*	intronic		4 tissues	BLD	
rs1078985	3	30690911	42,471	40,527	A>G	0.29	1.01	0.99	1.03	5.1E-01	*TGFBR2*	intronic			6 tissues	Maf,Myb
rs604663	10	6504126	42,484	40,568	A>G	0.45	1.01	0.99	1.03	5.1E-01	*PRKCQ*	intronic		KID	5 tissues	
rs1178162	7	18786967	42,459	40,540	C>A	0.21	1.01	0.98	1.03	5.1E-01	*HDAC9*	intronic				10 altered motifs
rs10199181	2	191873553	42,482	40,541	T>A	0.37	1.01	0.99	1.03	5.1E-01	*GLS, STAT1, STAT4, LOC100420571*	intronic				4 altered motifs
rs4842633	12	88941011	41,702	39,665	A>G	0.10	0.99	0.96	1.02	5.1E-01	*KITLG*	intronic		7 tissues	LNG,MUS,SKIN	14 altered motifs
rs6680740	1	92184088	42,497	40,572	G>A	0.27	1.01	0.99	1.03	5.1E-01	*TGFBR3*	intronic		BRST		4 altered motifs
rs10192157	2	102968356	42,500	40,566	G>A	0.38	1.01	0.99	1.03	5.1E-01	*IL18R1, IL1RL1*	missense				PRDM1,Pax-5,Roaz
rs534757	15	40226877	42,503	40,572	A>G	0.26	0.99	0.97	1.02	5.1E-01	*GPR176, EIF2AK4, H3F3AP1, LOC100505534*	intronic	24 tissues		33 tissues	
rs2301108	14	62197464	42,506	40,564	G>A	0.08	1.01	0.98	1.05	5.1E-01	*HIF1A, SNAPC1, HIF1A-AS2*	intronic		5 tissues	8 tissues	4 altered motifs
rs1320534	13	28641534	42,506	40,572	A>G	0.20	1.01	0.98	1.03	5.1E-01	*FLT3, CHCHD2P8, LOC100420919*	intronic		MUS	MUS	4 altered motifs
rs2287846	19	6696557	42,488	40,562	C>G	0.39	1.01	0.99	1.03	5.1E-01	*C3, TNFSF14, TRIP10, GPR108*	intronic		LIV, SPLN		E2F,Glis2
rs6461666	7	22796330	42,506	40,575	G>A	0.23	0.99	0.97	1.02	5.1E-01	*IL6, RPS26P32, LOC541472*				IPSC	AP-1
rs1887326	10	6608502	42,477	40,551	A>C	0.08	1.01	0.98	1.05	5.1E-01	*PRKCQ, PRKCQ-AS1*	intronic		6 tissues		7 altered motifs
rs9320007	18	59950871	42,502	40,569	A>C	0.34	1.01	0.99	1.03	5.1E-01	*TNFRSF11A, KIAA1468*	intronic		FAT, HRT, MUS		ERalpha-a,RAR,RORalpha1
rs7178	11	69469030	42,493	40,569	A>G	0.08	0.99	0.95	1.02	5.1E-01	*CCND1, FGF19, ORAOV1, LOC100996515*	3'-UTR		7 tissues	6 tissues	AhR,Ets,Pax-3
rs1178128	7	18767714	42,508	40,577	A>G	0.21	1.01	0.98	1.03	5.1E-01	*HDAC9*	intronic				AP-1,HMG-IY,PU.1
rs1178167	7	18791281	42,071	40,135	C>A	0.17	1.01	0.98	1.04	5.1E-01	*HDAC9*	intronic				8 altered motifs
rs8051462	16	85947327	42,504	40,572	A>G	0.45	1.01	0.99	1.03	5.1E-01	*IRF8*	intronic		11 tissues	6 tissues	E2F
rs6681271	1	157666644	42,501	40,574	A>G	0.47	0.99	0.97	1.01	5.1E-01	*FCRL2, FCRL3, SONP1, VDAC1P9*	intronic	BLD	BLD		GR
rs706087	7	18713515	42,290	40,399	A>G	0.20	1.01	0.98	1.03	5.1E-01	*HDAC9*	intronic				Irf,STAT
rs56227348	22	22145732	42,507	40,575	T>A	0.41	0.99	0.97	1.01	5.1E-01	*MAPK1*	intronic	BLD	7 tissues	BLD	13 altered motifs
rs579044	15	40249222	42,457	40,521	G>A	0.41	1.01	0.99	1.03	5.1E-01	*GPR176, EIF2AK4, H3F3AP1, LOC100505534*	intronic				Evi-1,SPIB,SRF
rs12677655	8	79712326	42,497	40,563	A>G	0.16	0.99	0.96	1.02	5.1E-01	*IL7, PRKRIRP7*	intronic				
rs4787948	16	27341059	42,500	40,562	A>G	0.30	0.99	0.97	1.01	5.1E-01	*IL4R, FLJ21408*	intronic		14 tissues	4 tissues	
rs9937847	16	85917551	42,507	40,575	A>G	0.07	0.99	0.95	1.03	5.1E-01	*IRF8*		ESDR			SRF
rs2474729	10	33473660	42,503	40,572	G>A	0.12	1.01	0.98	1.04	5.1E-01	*NRP1*					
rs2886086	12	6526672	41,578	39,550	G>C	0.41	0.99	0.97	1.01	5.1E-01	*CD27, LTBR, SCNN1A, VAMP1, TAPBPL, RPL31P10, SRP14P1, CD27-AS1*		BLD	BLD	IPSC,IPSC,BLD	AP-2,BDP1
rs2695026	7	18635619	42,129	40,179	G>A	0.24	0.99	0.97	1.02	5.2E-01	*HDAC9, LOC100419901*	intronic				DMRT3
rs631272	1	12206755	42,493	40,561	A>G	0.48	0.99	0.97	1.01	5.2E-01	*TNFRSF8, TNFRSF1B, MIR4632*			19 tissues	BLD	Hsf
rs12686783	9	101861767	42,502	40,563	G>A	0.11	1.01	0.98	1.04	5.2E-01	*COL15A1, TGFBR1*			BLD		
rs13022389	2	136868073	42,502	40,575	G>C	0.10	0.99	0.96	1.02	5.2E-01	*CXCR4*		BLD	BLD, BRN, ADRL	BLD	Nanog
rs7526590	1	92221454	42,491	40,575	A>T	0.17	1.01	0.98	1.04	5.2E-01	*TGFBR3*	intronic		9 tissues		9 altered motifs
rs35610689	7	22773820	42,507	40,571	A>G	0.26	1.01	0.98	1.03	5.2E-01	*IL6, RPS26P32, LOC541472*			BLD		6 altered motifs
rs3773634	3	30697936	42,503	40,575	A>G	0.29	1.01	0.99	1.03	5.2E-01	*TGFBR2*	intronic		15 tissues	VAS	HP1-site-factor

SNP	Chr	Position[a]	N Cases	N Controls	Allele	MAF	OR[b]	95%CI		*p* -value	Gene annotation	dbSNP functional annotation	Promoter histone marks	Enhancer histone marks	DNAse	Motifs changed
rs8052	3	150259942	42,507	40,576	G>A	0.12	1.01	0.98	1.04	5.2E-01	*SERP1, EIF2A*	3'-UTR	BLD	BLD, BRN, GI		PEBP
rs7100984	10	6078539	42,504	40,576	G>A	0.31	0.99	0.97	1.01	5.2E-01	*IL2RA, RPL32P23*	intronic		4 tissues		7 altered motifs
rs9512990	13	28606415	42,501	40,566	G>A	0.21	1.01	0.98	1.03	5.2E-01	*FLT3, PRHOXNB, LOC100420919*	intronic	BLD	BLD		TATA
rs7913599	10	6022562	42,502	40,565	G>A	0.35	0.99	0.97	1.01	5.2E-01	*IL2RA, IL15RA, FBXO18*			4 tissues		AFP1,Mef2,Zfp105
rs306588	10	30723593	42,045	40,092	A>G	0.31	0.99	0.97	1.01	5.2E-01	*CCND3P, MAP3K8*	intronic	22 tissues		51 tissues	E2F,ELF1,Nrf1
rs2583762	8	79697483	42,380	40,414	A>T	0.16	0.99	0.96	1.02	5.2E-01	*IL7, PRKRIRP7*	intronic				7 altered motifs
rs2160543	7	18720602	42,506	40,574	A>G	0.21	0.99	0.97	1.02	5.2E-01	*HDAC9*	intronic		SKIN, BRN	SKIN	
rs2520458	7	18622929	42,506	40,573	A>C	0.38	1.01	0.99	1.03	5.2E-01	*HDAC9, LOC100419901*	intronic		6 tissues	SKIN,ADRL,HRT	11 altered motifs
rs3207297	15	40313141	42,506	40,574	G>A	0.36	0.99	0.97	1.01	5.2E-01	*SRP14, EIF2AK4, SRP14-AS1*	synonymous				NF-I,TAL1
rs805321	2	54139017	42,493	40,551	A>G	0.31	0.99	0.97	1.01	5.2E-01	*PSME4*	intronic				Pou3f1
rs7023227	9	5456550	42,501	40,571	G>A	0.42	0.99	0.97	1.01	5.2E-01	*CD274, PLGRKT, LOC100419687*	intronic	BLD	BLD, MUS, VAS	BLD,BLD	
rs2158768	7	18970150	42,500	40,573	C>A	0.46	0.99	0.97	1.01	5.2E-01	*HDAC9, NPM1P13*	intronic				6 altered motifs
rs1343152	1	67704332	42,479	40,561	A>C	0.35	1.01	0.99	1.03	5.2E-01	*IL23R, LOC100130497*	intronic				4 altered motifs
rs12591842	15	40317608	42,508	40,575	G>A	0.30	0.99	0.97	1.01	5.2E-01	*SRP14, EIF2AK4, SRP14-AS1*	intronic				5 altered motifs
rs294218	1	23033257	42,452	40,539	A>C	0.38	1.01	0.99	1.03	5.2E-01	*C1QB, EPHB2, MIR4684*					Foxd1,Zfp410
rs5755694	22	22200530	42,501	40,573	G>A	0.48	1.01	0.99	1.03	5.2E-01	*MAPK1*	intronic		13 tissues	5 tissues	
rs5746014	1	12250888	42,484	40,569	A>G	0.10	1.01	0.98	1.05	5.2E-01	*TNFRSF8, TNFRSF1B, VPS13D, LOC390998, MIR4632*	intronic		ESC, BLD, PLCNT		Irf
rs2234768	10	90749943	42,507	40,572	A>G	0.12	1.01	0.98	1.04	5.2E-01	*ACTA2, FAS, FAS-AS1*	intronic	22 tissues	8 tissues	38 tissues	Pou2f2
rs7802277	7	22782613	42,504	40,572	G>A	0.15	0.99	0.96	1.02	5.2E-01	*IL6, RPS26P32, LOC541472*					5 altered motifs
rs1307968	11	69515423	42,495	40,573	A>G	0.14	0.99	0.96	1.02	5.2E-01	*CCND1, FGF19, ORAOV1, LOC100129779, LOC100996515*	intronic	6 tissues	21 tissues	28 tissues	Hsf
rs10122089	9	5463780	42,496	40,565	G>A	0.49	0.99	0.97	1.01	5.2E-01	*CD274, PLGRKT, PDCD1LG2, LOC100419687*	intronic				GR,HNF4,Nr2e3
rs6764672	3	150255152	42,505	40,572	A>C	0.12	1.01	0.98	1.04	5.2E-01	*SERP1, EIF2A*	intronic				
rs305064	16	85974142	42,488	40,556	G>A	0.34	1.01	0.99	1.03	5.2E-01	*IRF8*		GI, BLD	4 tissues	8 tissues	Dbx2,Maf,Pou5f1
rs12722527	10	6077328	42,495	40,563	G>A	0.16	1.01	0.98	1.04	5.2E-01	*IL2RA, RPL32P23*	intronic		5 tissues	OVRY	6 altered motifs
rs3213097	5	158748679	42,447	40,525	A>T	0.19	1.01	0.98	1.03	5.2E-01	*IL12B, UBLCP1, LOC285626, RNU4ATAC2P*	intronic				DEC,Nkx2
rs4763813	12	9862336	42,492	40,547	G>A	0.07	1.01	0.97	1.05	5.3E-01	*CD69, NPM1P7, CLEC2D, CLECL1*			BLD	BLD,BLD	Zfp410
rs78244689	11	69516025	42,506	40,570	G>A	0.15	1.01	0.98	1.04	5.3E-01	*CCND1, FGF19, ORAOV1, LOC100129779, LOC100996515*					
rs2031229	10	6066568	42,507	40,571	G>A	0.24	1.01	0.98	1.03	5.3E-01	*IL2RA, IL15RA, RPL32P23*	intronic		SKIN, BRN	SKIN	XBP-1
rs12076549	1	92201699	42,504	40,573	C>A	0.42	0.99	0.97	1.01	5.3E-01	*TGFBR3*	intronic		15 tissues		29 altered motifs
rs17131522	1	92151273	42,507	40,565	G>A	0.20	1.01	0.98	1.03	5.3E-01	*TGFBR3, HSP90B3P*	intronic		14 tissues		4 altered motifs
rs2526630	7	19036578	42,505	40,575	G>A	0.45	1.01	0.99	1.03	5.3E-01	*HDAC9, NPM1P13*	3'-UTR		5 tissues	LNG,MUS	4 altered motifs
rs929087	1	172632057	42,506	40,573	G>A	0.46	1.01	0.99	1.03	5.3E-01	*FASLG*	intronic	BLD, FAT, GI	12 tissues	26 tissues	Rad21
rs8083014	18	60030587	42,505	40,572	C>A	0.24	0.99	0.97	1.02	5.3E-01	*TNFRSF11A*	intronic				Zfp691
rs2569254	5	158751249	42,496	40,575	G>A	0.20	1.01	0.98	1.03	5.3E-01	*IL12B, UBLCP1, LOC285626, RNU4ATAC2P*	intronic		5 tissues	SKIN,LNG	Pax-6
rs7986482	13	43210254	42,466	40,559	G>A	0.40	1.01	0.99	1.03	5.3E-01	*TNFSF11*					
rs805437	2	54054978	42,508	40,575	G>A	0.32	1.01	0.99	1.03	5.3E-01	*GPR75, PSME4, ERLEC1, ASB3, GPR75-ASB3, MIR3682*	intronic	BRN, LIV	15 tissues		GR,MZF1::1-4
rs11209018	1	67667291	42,502	40,571	G>A	0.46	0.99	0.97	1.01	5.3E-01	*IL23R*	intronic				CEBPA,CEBPB
rs6432018	2	9721896	42,500	40,576	A>C	0.45	0.99	0.97	1.01	5.3E-01	*ADAM17, YWHAQ*			MUS	GI	AP-1,HNF4,PPAR
rs340812	3	3125337	42,509	40,576	A>G	0.18	1.01	0.98	1.03	5.3E-01	*IL5RA, TRNT1, CNTN4*	intronic		GI, BLD		INSM1,Ik-2,SRF
rs17257083	14	25074711	42,506	40,569	A>G	0.16	0.99	0.96	1.02	5.3E-01	*CTSG, GZMH, GZMB*			BLD, SKIN	13 tissues	Mef2,Ncx
rs11466414	14	76447850	42,507	40,576	G>A	0.07	0.99	0.95	1.03	5.3E-01	*TGFB3, TTLL5, IFT43, LOC100506576*	5'-UTR	23 tissues	4 tissues	38 tissues	GR,LXR
rs3024994	6	43743507	42,509	40,576	G>A	0.06	1.01	0.97	1.06	5.3E-01	*VEGFA*	intronic	FAT	20 tissues	BLD,BLD	
rs706086	7	18707842	42,484	40,546	G>A	0.09	0.99	0.96	1.02	5.3E-01	*HDAC9*	3'-UTR		MUS, VAS, SKIN		4 altered motifs
rs2286003	7	19000257	42,507	40,575	G>A	0.19	1.01	0.98	1.03	5.3E-01	*HDAC9, NPM1P13*	intronic				5 altered motifs
rs2302879	2	54152927	42,478	40,532	A>G	0.31	0.99	0.97	1.01	5.3E-01	*PSME4*	intronic				7 altered motifs
rs10282136	7	18991904	42,488	40,562	G>A	0.46	1.01	0.99	1.03	5.3E-01	*HDAC9, NPM1P13*	intronic				BAF155
rs10493856	1	92334749	42,501	40,572	A>G	0.40	0.99	0.97	1.01	5.3E-01	*TGFBR3*	intronic			BLD	TEF-1,Zbtb12
rs3138060	2	219031500	42,499	40,568	C>G	0.05	0.99	0.94	1.03	5.3E-01	*CXCR1, CXCR2, HMGB1P9*	intronic	BLD	5 tissues	BLD	En-1,Zic
rs9319410	13	28591318	42,388	40,481	A>G	0.21	1.01	0.98	1.03	5.3E-01	*CDX2, FLT3, PRHOXNB*	intronic				4 altered motifs
rs6420908	14	76384993	42,499	40,562	A>G	0.07	0.99	0.95	1.03	5.4E-01	*TGFB3, TTLL5*	intronic		BLD		7 altered motifs
rs723157	7	18622368	41,891	40,049	G>A	0.47	0.99	0.97	1.01	5.4E-01	*HDAC9, LOC100419901*	intronic		ESDR, HRT	BLD	
rs12124897	1	92331900	42,505	40,574	T>A	0.40	0.99	0.97	1.01	5.4E-01	*TGFBR3*	intronic		7 tissues		9 altered motifs
rs12146362	10	6503966	42,509	40,568	A>T	0.16	0.99	0.96	1.02	5.4E-01	*PRKCQ*	intronic		KID		GATA
rs10923135	1	117255312	42,471	40,540	A>G	0.19	0.99	0.97	1.02	5.4E-01	*CD2, IGSF3, NEFHP1, GAPDHP64, MIR320B1*				4 tissues	RXRA
rs1053005	17	40465910	42,505	40,575	A>G	0.19	1.01	0.98	1.03	5.4E-01	*STAT3, STAT5A, STAT5B*	3'-UTR			13 tissues	
rs12248888	10	6496186	41,949	40,071	C>G	0.33	1.01	0.99	1.03	5.4E-01	*PRKCQ*	intronic				HNF1
rs10215908	7	18845072	42,507	40,576	A>G	0.07	0.99	0.95	1.03	5.4E-01	*HDAC9*	intronic			HRT	
rs2704282	7	18644378	42,494	40,572	G>A	0.23	0.99	0.97	1.02	5.4E-01	*HDAC9, LOC100419901*	intronic			BRN	BCL,Irf

SNP	Chr	Position[a]	N Cases	N Controls	Allele	MAF	OR[b]	95%CI		*p* -value	Gene annotation	dbSNP functional annotation	Promoter histone marks	Enhancer histone marks	DNAse	Motifs changed
rs9554228	13	28637838	42,504	40,571	G>A	0.39	1.01	0.99	1.03	5.4E-01	*FLT3, CHCHD2P8, LOC100420919*	intronic				Pou2f2
rs805429	2	54131696	42,502	40,568	A>G	0.31	0.99	0.97	1.01	5.4E-01	*GPR75, PSME4, GPR75-ASB3*	intronic				7 altered motifs
rs41511150	2	191854639	42,509	40,569	C>A	0.14	1.01	0.98	1.04	5.4E-01	*GLS, STAT1, STAT4, LOC100420571*	intronic		ESDR, BLD, FAT		6 altered motifs
rs16906115	8	79712998	42,507	40,565	G>A	0.09	0.99	0.95	1.02	5.4E-01	*IL7, PRKRIRP7*	intronic				ZNF263
rs451698	1	92241053	42,500	40,572	A>G	0.27	1.01	0.98	1.03	5.4E-01	*TGFBR3*	intronic		12 tissues		4 altered motifs
rs1801275	16	27374400	42,483	40,535	A>G	0.21	0.99	0.97	1.02	5.4E-01	*IL4R, IL21R*	missense		5 tissues	ESDR,OVRY	CDP,Pax-5
rs7028894	9	5460910	42,463	40,518	G>A	0.49	0.99	0.97	1.01	5.5E-01	*CD274, PLGRKT, PDCD1LG2, LOC100419687*	intronic		BLD		GR,HMG-IY,YY1
rs11864884	16	85901039	42,498	40,569	G>A	0.27	1.01	0.98	1.03	5.5E-01	*IRF8*			BLD	5 tissues	NRSF
rs17348528	7	18706703	42,505	40,573	A>G	0.12	0.99	0.96	1.02	5.5E-01	*HDAC9*	3'-UTR		8 tissues	PANC	4 altered motifs
rs1779384	10	44894443	42,503	40,573	C>A	0.21	1.01	0.98	1.03	5.5E-01	*CXCL12, RPL9P21*			ESC, ESDR, IPSC	5 tissues	ZBTB33
rs2494732	14	105239192	42,500	40,575	A>G	0.43	1.01	0.99	1.03	5.5E-01	*AKT1, SIVA1, ADSSL1, LINC00638, ZBTB42*	intronic		SPLN	LIV	AP-4,LBP-1,Zfp410
rs10271724	7	18971647	42,505	40,575	A>G	0.47	0.99	0.97	1.01	5.5E-01	*HDAC9, NPM1P13*	intronic				7 altered motifs
rs805445	2	54175440	42,508	40,571	A>G	0.14	0.99	0.96	1.02	5.5E-01	*PSME4*	intronic		BLD		ZEB1
rs12722489	10	6102012	42,509	40,575	G>A	0.16	1.01	0.98	1.04	5.5E-01	*IL2RA, RBM17, RPL32P23*	intronic	BLD, GI	BLD, HRT, MUS	BLD	ERalpha-a
rs2071374	2	113537352	42,498	40,564	A>C	0.27	1.01	0.98	1.03	5.5E-01	*IL1A, IL1B, CKAP2L*	intronic		BRST, SKIN	SKIN,SKIN	Ik-3,Pou2f2
rs340833	3	3111458	42,502	40,574	A>G	0.49	0.99	0.97	1.01	5.5E-01	*IL5RA, CNTN4*	3'-UTR				6 altered motifs
rs1113283	17	26107398	42,508	40,576	G>A	0.24	0.99	0.97	1.02	5.5E-01	*NOS2, LOC645754*	intronic		STRM, SKIN	SKIN	Nkx2,ZBRK1
rs2246012	6	131898208	42,506	40,575	A>G	0.17	0.99	0.97	1.02	5.5E-01	*ARG1, MED23*	intronic		4 tissues		
rs1467142	2	219026091	42,509	40,576	A>G	0.05	0.99	0.94	1.03	5.5E-01	*CXCR1, CXCR2, HMGB1P9*		BLD			4 altered motifs
rs2498788	14	105253009	42,467	40,526	G>A	0.07	0.99	0.95	1.03	5.5E-01	*AKT1, SIVA1, RPS2P4, ADSSL1, LINC00638, ZBTB42, RPS26P49*	intronic	BLD, MUS	16 tissues		YY1
rs12925861	16	27342596	42,504	40,571	A>T	0.30	0.99	0.97	1.02	5.5E-01	*IL4R, FLJ21408*	intronic	FAT, STRM, GI	19 tissues	BLD	HNF4
rs1945764	11	118182801	42,504	40,577	A>G	0.34	0.99	0.97	1.01	5.5E-01	*CD3D, CD3E, CD3G, UBE4A, MPZL2*	intronic		4 tissues	BLD	PLZF
rs117154698	10	6031260	42,501	40,565	T>A	0.35	0.99	0.97	1.01	5.5E-01	*IL2RA, IL15RA*					
rs284173	1	92215714	42,510	40,577	G>C	0.16	1.01	0.98	1.04	5.5E-01	*TGFBR3*	intronic	CRVX	18 tissues	CRVX,SKIN	
rs4970362	1	1094738	41,886	40,014	G>A	0.37	0.99	0.97	1.01	5.5E-01	*TNFRSF18, C1orf159, LOC254099, TTLL10, MIR200A, MIR200B, MIR429, TTLL10-AS1*		SKIN, GI	10 tissues	GI	GR
rs822340	9	5453260	42,459	40,531	G>A	0.25	0.99	0.97	1.02	5.5E-01	*CD274, PLGRKT, LOC100419687*	intronic	4 tissues	6 tissues	6 tissues	
rs10505504	8	128700344	42,505	40,573	G>A	0.36	0.99	0.97	1.01	5.5E-01	*MYC*				4 tissues	GATA
rs11465804	1	67702526	42,508	40,575	A>C	0.06	1.01	0.97	1.06	5.5E-01	*IL23R, LOC100130497*	intronic				6 altered motifs
rs10248070	7	18835449	42,506	40,576	A>G	0.06	0.99	0.95	1.03	5.5E-01	*HDAC9*	intronic				SIX5,XBP-1,Znf143
rs9513011	13	28630691	42,504	40,568	C>G	0.21	1.01	0.98	1.03	5.5E-01	*FLT3, LOC100420919*	intronic		5 tissues	IPSC,MUS,BLD	
rs10094872	8	128719884	42,503	40,577	A>T	0.39	1.01	0.99	1.03	5.5E-01	*MYC*				7 tissues	11 altered motifs
rs1144396	11	102679052	42,504	40,576	A>C	0.50	0.99	0.97	1.01	5.5E-01	*MMP1, MMP3, MMP10, CSNK1A1P2, WTAPP1, LOC100421658*	intronic	ESC, IPSC, LNG	ESC	5 tissues	NRSF,Sin3Ak-20
rs2306887	1	92200218	42,473	40,541	A>G	0.31	0.99	0.97	1.02	5.5E-01	*TGFBR3*	intronic		9 tissues		4 altered motifs
rs2953474	8	79670387	42,483	40,560	C>A	0.16	0.99	0.97	1.02	5.5E-01	*IL7, ZC2HC1A, PRKRIRP7*	intronic			BLD	
rs12506479	4	74592161	42,505	40,573	A>G	0.26	1.01	0.98	1.03	5.5E-01	*IL8*		STRM, LNG	17 tissues	8 tissues	4 altered motifs
rs8177582	10	6028440	42,505	40,575	C>G	0.15	1.01	0.98	1.04	5.5E-01	*IL2RA, IL15RA, FBXO18*			5 tissues	SKIN,MUS,SKIN	Sox
rs6973918	7	18963677	42,505	40,573	G>A	0.12	1.01	0.98	1.04	5.5E-01	*HDAC9, NPM1P13*	intronic				GR
rs10206753	2	102968362	42,467	40,537	A>G	0.38	1.01	0.99	1.03	5.5E-01	*IL18R1, IL1RL1*	missense				Pax-5,Pou2f2,Roaz
rs10276724	7	18926662	42,510	40,576	A>G	0.26	1.01	0.98	1.03	5.5E-01	*HDAC9*	intronic				
rs805315	2	54132540	42,508	40,576	A>G	0.31	0.99	0.97	1.02	5.5E-01	*GPR75, PSME4, GPR75-ASB3*	intronic				CCNT2,TAL1
rs12044500	1	92231325	42,508	40,573	A>G	0.22	0.99	0.97	1.02	5.5E-01	*TGFBR3*	intronic		7 tissues	PLCNT,CRVX	GATA
rs6415794	9	5458702	42,474	40,526	T>A	0.49	0.99	0.97	1.01	5.5E-01	*CD274, PLGRKT, LOC100419687*	intronic		4 tissues		NRSF
rs7652589	3	121889088	42,498	40,569	G>A	0.40	0.99	0.97	1.01	5.5E-01	*CASR, CD86*			SKIN, BRST	SKIN,BRST,SKIN	GCNF,Hmx,Nanog
rs2269923	7	18997811	42,386	40,476	C>A	0.24	0.99	0.97	1.02	5.5E-01	*HDAC9, NPM1P13*	intronic		SKIN, MUS		Gfi1,Hdx,Nanog
rs6678420	1	92184253	42,499	40,569	G>C	0.50	0.99	0.97	1.01	5.6E-01	*TGFBR3*	intronic		BRST, LIV		HMG-IY
rs41284471	10	6626214	42,508	40,577	G>A	0.19	1.01	0.98	1.03	5.6E-01	*PRKCQ, PRKCQ-AS1*		BLD	BLD		Pou3f1,STAT
rs630516	10	6506947	42,468	40,566	G>C	0.47	0.99	0.97	1.01	5.6E-01	*PRKCQ*	intronic		BLD	ESDR,BLD	Hic1,PRDM1,VDR
rs266105	10	44855663	42,496	40,568	G>A	0.12	0.99	0.96	1.02	5.6E-01	*CXCL12*					BATF,PU.1
rs2859242	1	172624864	42,506	40,576	G>A	0.44	0.99	0.97	1.01	5.6E-01	*FASLG, SUCO*			BRST		5 altered motifs
rs12926854	16	85951258	42,487	40,568	A>G	0.26	0.99	0.97	1.02	5.6E-01	*IRF8*	intronic		BLD, GI	BLD,BLD,GI	5 altered motifs
rs2241043	22	17567807	42,497	40,569	A>G	0.37	1.01	0.99	1.03	5.6E-01	*IL17RA, CECR6, CECR7, RPL31P62, LOC100996342*	intronic	8 tissues	15 tissues	6 tissues	
rs7517044	1	92198162	42,500	40,566	A>G	0.31	0.99	0.97	1.02	5.6E-01	*TGFBR3*	intronic	5 tissues	22 tissues	23 tissues	6 altered motifs
rs2868945	3	150322418	42,507	40,576	A>G	0.12	1.01	0.98	1.04	5.6E-01	*SELT, EIF2A, LOC677762*	intronic	19 tissues	11 tissues	BLD,BLD	5 altered motifs
rs4771207	13	28621473	42,504	40,566	A>C	0.21	1.01	0.98	1.03	5.6E-01	*FLT3, LOC100420919*	intronic		ESDR, LNG, BLD	ESDR,LNG	GATA,Mef2,Mrg
rs10110519	8	79688026	42,504	40,573	C>A	0.09	0.99	0.95	1.03	5.6E-01	*IL7, PRKRIRP7*	intronic				4 altered motifs
rs2506140	10	33467539	42,507	40,574	A>G	0.15	0.99	0.96	1.02	5.6E-01	*NRP1*	3'-UTR		11 tissues	11 tissues	
rs2073974	7	18624800	42,502	40,567	G>A	0.47	0.99	0.97	1.01	5.6E-01	*HDAC9, LOC100419901*	intronic				IRC900814
rs2284792	14	76443579	42,491	40,560	A>G	0.25	0.99	0.97	1.02	5.6E-01	*TGFB3, TTLL5, IFT43, LOC100506576*	intronic		12 tissues	ESC,CRVX	CEBPB

SNP	Chr	Position[a]	N Cases	N Controls	Allele	MAF	OR[b]	95%CI		*p* -value	Gene annotation	dbSNP functional annotation	Promoter histone marks	Enhancer histone marks	DNAse	Motifs changed
rs11256258	10	6033415	42,501	40,563	A>G	0.10	1.01	0.98	1.04	5.6E-01	*IL2RA, IL15RA*					TAL1
rs884205	18	60054857	42,507	40,574	C>A	0.26	1.01	0.98	1.03	5.6E-01	*TNFRSF11A, RPL17P44*			GI	GI	10 altered motifs
rs10779329	1	218573741	42,508	40,574	A>G	0.23	0.99	0.97	1.02	5.6E-01	*TGFB2*	intronic				
rs10278449	7	18931219	42,507	40,577	A>G	0.15	1.01	0.98	1.04	5.6E-01	*HDAC9*	intronic			ESDR	Znf143
rs10874911	1	92194523	42,507	40,570	A>G	0.48	1.01	0.99	1.03	5.6E-01	*TGFBR3*	intronic				NRSF,Roaz
rs1053023	17	40465616	42,506	40,576	A>G	0.19	1.01	0.98	1.03	5.6E-01	*STAT3, STAT5A, STAT5B*	3'-UTR			10 tissues	CEBPG,Pdx1
rs11165372	1	92199295	42,506	40,571	G>A	0.31	0.99	0.97	1.02	5.6E-01	*TGFBR3*	intronic		8 tissues		9 altered motifs
rs3763959	17	25957334	42,465	40,548	G>A	0.44	0.99	0.97	1.01	5.6E-01	*LGALS9, KSR1, NOS2P1, ITM2BP1, LOC100420408*		GI	4 tissues	BLD	ZBTB33,Zfp161
rs12699982	7	18846124	42,507	40,576	A>G	0.28	1.01	0.98	1.03	5.6E-01	*HDAC9*	intronic		ESDR, IPSC		TATA,YY1
rs518770	15	40230272	42,507	40,573	A>G	0.26	0.99	0.97	1.02	5.6E-01	*GPR176, EIF2AK4, H3F3AP1, LOC100505534*	intronic	BLD, BONE	15 tissues	SKIN	DBP
rs10795791	10	6108340	42,508	40,576	A>G	0.42	0.99	0.97	1.01	5.6E-01	*IL2RA, RBM17, RPL32P23*				BLD,BLD	GATA,Mrg,TAL1
rs1323651	10	6033301	42,506	40,574	C>A	0.39	0.99	0.97	1.01	5.6E-01	*IL2RA, IL15RA*					Cdx2,Foxl1,Pax-6
rs3757718	7	18568627	42,505	40,576	G>A	0.28	1.01	0.98	1.03	5.6E-01	*HDAC9, LOC100419901*	intronic	VAS	5 tissues	MUS	NF-I
rs3917192	14	76431674	42,509	40,577	G>A	0.16	0.99	0.97	1.02	5.6E-01	*TGFB3, TTLL5, IFT43, LOC100506576*	intronic		BLD	BLD	YY1,p53
rs9512979	13	28595203	42,504	40,574	A>G	0.21	1.01	0.98	1.03	5.6E-01	*FLT3, PRHOXNB, LOC100420919*	intronic				Smad3
rs3901892	7	18819605	42,328	40,332	A>G	0.25	1.01	0.98	1.03	5.6E-01	*HDAC9*	intronic		GI, SKIN		Egr-1,Hand1
rs2919935	8	79683948	42,490	40,559	T>A	0.08	0.99	0.95	1.03	5.6E-01	*IL7, PRKRIRP7*	intronic				Foxp1,Irx,TCF4
rs284185	1	92233820	42,487	40,548	T>A	0.41	0.99	0.97	1.01	5.6E-01	*TGFBR3*	intronic		PLCNT, LIV		6 altered motifs
rs1143634	2	113590390	42,496	40,567	G>A	0.25	0.99	0.97	1.02	5.6E-01	*IL1A, IL1B, LOC100128413*	synonymous	BLD	7 tissues	BLD,SKIN,BLD	
rs4843860	16	85950921	42,491	40,568	A>G	0.26	0.99	0.97	1.02	5.6E-01	*IRF8*	intronic		9 tissues	BLD	15 altered motifs
rs805349	2	54155240	42,498	40,570	A>G	0.31	0.99	0.97	1.02	5.6E-01	*PSME4*	intronic				Foxp1
rs3917410	1	169701108	42,510	40,577	A>G	0.11	0.99	0.96	1.02	5.6E-01	*SELE, SELL*	intronic				ZNF263
rs7517847	1	67681669	42,493	40,567	A>C	0.44	0.99	0.97	1.01	5.6E-01	*IL23R*	intronic				4 altered motifs
rs7845292	8	128702676	42,503	40,571	A>G	0.43	0.99	0.97	1.01	5.6E-01	*MYC*					Foxp3,NRSF
rs34997637	2	191858830	42,507	40,574	G>A	0.24	1.01	0.98	1.03	5.7E-01	*GLS, STAT1, STAT4, LOC100420571*	intronic				DMRT2,DMRT3
rs41380244	11	102713447	42,428	40,480	G>C	0.09	1.01	0.98	1.05	5.7E-01	*MMP1, MMP3, MMP12, CSNK1A1P2, WTAPP1, LOC100288111*	synonymous		BRST, SKIN		5 altered motifs
rs5361	1	169701060	42,506	40,573	A>C	0.11	0.99	0.96	1.02	5.7E-01	*SELE, SELL*	missense				4 altered motifs
rs8054065	16	85951354	42,494	40,573	A>G	0.49	1.01	0.99	1.03	5.7E-01	*IRF8*	intronic		BLD, GI	BLD	Ik-1,Pou1f1
rs580836	15	40249020	42,503	40,576	A>G	0.24	0.99	0.97	1.02	5.7E-01	*GPR176, EIF2AK4, H3F3AP1, LOC100505534*	intronic				4 altered motifs
rs1836981	10	44827011	42,494	40,552	G>A	0.43	1.01	0.99	1.03	5.7E-01	*CXCL12, LOC100130539*					Bcl6b,EBF
rs6498012	16	27331974	42,478	40,545	G>C	0.38	0.99	0.97	1.01	5.7E-01	*IL4R, FLJ21408*	intronic	GI	12 tissues		Hdx,NF-I,p300
rs3773644	3	30712344	42,500	40,564	G>A	0.42	0.99	0.97	1.01	5.7E-01	*TGFBR2*	intronic		BLD, VAS, MUS		6 altered motifs
rs1178129	7	18768334	42,508	40,577	A>C	0.21	1.01	0.98	1.03	5.7E-01	*HDAC9*	intronic				6 altered motifs
rs2717543	8	79682613	42,486	40,557	G>A	0.16	0.99	0.97	1.02	5.7E-01	*IL7, PRKRIRP7*	intronic				Dlx2,ERalpha-a,HNF1
rs3788268	22	17626665	42,447	40,542	G>A	0.15	0.99	0.96	1.02	5.7E-01	*RPL32P5, IL17RA, CECR6, CECR5, CECR1, CECR5-AS1, LOC100996342*	intronic			MUS	Pbx3,p300
rs12979435	19	6641342	42,119	40,262	C>A	0.33	0.99	0.97	1.02	5.7E-01	*C3, TNFSF14, RPL7P50*		BLD	BLD, GI, THYM	BLD	11 altered motifs
rs11646550	16	85938835	42,509	40,576	G>A	0.15	1.01	0.98	1.04	5.7E-01	*IRF8*	intronic		4 tissues	BLD	Hoxa7,NF-I
rs3731973	2	54159999	42,506	40,572	G>A	0.14	0.99	0.96	1.02	5.7E-01	*PSME4*	intronic				Foxq1,HNF1,Mef2
rs10807291	6	43781918	42,508	40,575	G>A	0.15	0.99	0.96	1.02	5.7E-01	*VEGFA*					SRF
rs12135884	1	172638500	42,506	40,572	A>G	0.31	0.99	0.97	1.02	5.7E-01	*FASLG*			BLD		CEBPB
rs2506143	10	33468169	42,509	40,570	A>G	0.15	0.99	0.96	1.02	5.7E-01	*NRP1*	3'-UTR	STRM	15 tissues	6 tissues	5 altered motifs
rs11465817	1	67721097	42,465	40,533	C>A	0.28	1.01	0.98	1.03	5.7E-01	*IL23R, LOC100130497*	intronic				
rs13122023	4	123331316	42,498	40,562	A>C	0.27	0.99	0.97	1.02	5.7E-01	*IL2, KIAA1109, ADAD1*	intronic				5 altered motifs
rs2810886	1	92163330	42,498	40,571	A>G	0.25	0.99	0.97	1.02	5.7E-01	*TGFBR3*	intronic	FAT, STRM, BLD	14 tissues	7 tissues	GR
rs2494738	14	105246686	42,509	40,575	G>A	0.07	0.99	0.95	1.03	5.7E-01	*AKT1, SIVA1, ADSSL1, LINC00638, ZBTB42*	intronic		ESDR, IPSC, SKIN	BLD,HRT,THYM	4 altered motifs
rs2153000	13	28622356	42,495	40,567	A>G	0.21	1.01	0.98	1.03	5.7E-01	*FLT3, LOC100420919*	intronic	BLD	ESC, IPSC, BLD	BLD,BLD,BLD	
rs7235803	18	60000379	42,479	40,544	A>G	0.34	0.99	0.97	1.02	5.7E-01	*TNFRSF11A, KIAA1468*	intronic	BLD	6 tissues	HRT,GI	4 altered motifs
rs587198	10	6531149	42,494	40,571	A>G	0.45	0.99	0.97	1.01	5.7E-01	*PRKCQ*	intronic			IPSC	SIX5,TBX5,Tgif1
rs10234468	7	18835725	42,508	40,577	A>G	0.06	0.99	0.95	1.03	5.7E-01	*HDAC9*	intronic				TCF11::MafG
rs1492907	1	172642118	42,508	40,577	G>A	0.30	0.99	0.97	1.02	5.7E-01	*FASLG*			ESDR, BLD, SKIN		Bach1
rs7089504	10	6516327	42,507	40,571	A>G	0.35	0.99	0.97	1.02	5.7E-01	*PRKCQ*	intronic	BLD, THYM, GI	6 tissues	6 tissues	4 altered motifs
rs3888020	8	79716536	42,335	40,430	C>A	0.37	1.01	0.99	1.03	5.7E-01	*IL7, PRKRIRP7*	intronic	23 tissues	4 tissues	5 tissues	9 altered motifs
rs7524066	1	92184814	42,507	40,572	C>A	0.28	0.99	0.97	1.02	5.7E-01	*TGFBR3*	intronic		4 tissues	LIV	
rs2241042	22	17567699	42,494	40,568	C>A	0.38	1.01	0.99	1.03	5.7E-01	*IL17RA, CECR6, CECR7, RPL31P62, LOC100996342*	intronic	10 tissues	14 tissues	BLD,MUS	5 altered motifs
rs805312	2	54149385	42,500	40,572	A>C	0.31	0.99	0.97	1.02	5.7E-01	*PSME4*	intronic				HMG-IY,Ik-1,Pax-4
rs1800587	2	113542960	42,508	40,574	G>A	0.31	0.99	0.97	1.02	5.7E-01	*IL1A, IL1B, CKAP2L*	5'-UTR	SKIN	8 tissues		Bbx,RFX5
rs11801868	1	92208326	42,263	40,444	G>A	0.38	1.01	0.99	1.03	5.7E-01	*TGFBR3*	intronic	GI	16 tissues	MUS,VAS	HNF4,Mtf1
rs3092949		135727335	42,506	40,571	G>A	0.34	0.99	0.97	1.02	5.7E-01	*CD40LG, ARHGEF6, LINC00892*			BLD		5 altered motifs

SNP	Chr	Position[a]	N Cases	N Controls	Allele	MAF	OR[b]	95%CI		*p* -value	Gene annotation	dbSNP functional annotation	Promoter histone marks	Enhancer histone marks	DNAse	Motifs changed
rs805415	2	54124791	42,479	40,565	A>G	0.31	0.99	0.97	1.02	5.7E-01	*GPR75, PSME4, GPR75-ASB3, MIR3682*	intronic		9 tissues		15 altered motifs
rs1476482	7	22730709	42,507	40,576	A>G	0.05	1.01	0.97	1.06	5.7E-01	*IL6, LOC401312, LOC541472*					Sox
rs502720	15	40255053	42,510	40,575	G>A	0.41	1.01	0.99	1.03	5.7E-01	*GPR176, EIF2AK4, H3F3AP1, LOC100505534*	intronic		SKIN		CAC-binding-protein,ERalpha-a,RXRA
rs10167514	2	191860529	42,504	40,569	G>A	0.24	1.01	0.98	1.03	5.7E-01	*GLS, STAT1, STAT4, LOC100420571*	intronic				TCF4
rs11898680	2	113556867	42,506	40,573	G>A	0.33	0.99	0.97	1.02	5.7E-01	*IL1A, IL1B, CKAP2L*			BLD	4 tissues	6 altered motifs
rs12083537	1	154381103	42,502	40,568	A>G	0.21	0.99	0.97	1.02	5.7E-01	*IL6R, MRPS33P1, RPSAP17, PSMD8P1*	intronic	7 tissues	19 tissues	9 tissues	11 altered motifs
rs12759271	1	67906853	42,507	40,575	A>C	0.39	1.01	0.99	1.03	5.7E-01	*IL12RB2, SERBP1*		BLD	19 tissues	BLD,HRT	Mef2,XBP-1
rs2096766	11	102791236	42,479	40,563	T>A	0.37	0.99	0.97	1.01	5.7E-01	*MMP12, MMP13, LOC100288111*		BLD	BLD, FAT	4 tissues	
rs4655107	1	23094454	42,503	40,572	G>A	0.24	0.99	0.97	1.02	5.7E-01	*EPHB2, MIR4684*	intronic		ESDR, BRN		NRSF,Pax-2,Pax-5
rs3794766	17	26121921	42,330	40,324	G>A	0.24	0.99	0.97	1.02	5.7E-01	*NOS2, LOC645754*	intronic		GI, LIV	4 tissues	CHOP::CEBPalpha,NF-kappaB
rs9472114	6	43695675	42,476	40,553	A>G	0.26	1.01	0.98	1.03	5.7E-01	*VEGFA, MRPS18A, LOC100132242*			SKIN		
rs10261390	7	18927022	42,502	40,573	G>A	0.26	1.01	0.98	1.03	5.7E-01	*HDAC9*	intronic				CDP,Pbx-1
rs2189741	7	19015263	42,506	40,575	C>A	0.23	0.99	0.97	1.02	5.8E-01	*HDAC9, NPM1P13*	intronic		5 tissues		Arid5a,Bcl6b,Mef2
rs2853556	7	18969241	42,484	40,526	G>C	0.46	0.99	0.97	1.01	5.8E-01	*HDAC9, NPM1P13*	intronic				Evi-1,Hltf,Mef2
rs9512977	13	28591773	42,473	40,520	A>G	0.21	1.01	0.98	1.03	5.8E-01	*CDX2, FLT3, PRHOXNB, LOC100420919*	intronic				5 altered motifs
rs2781667	6	131895144	42,505	40,576	G>A	0.31	1.01	0.98	1.03	5.8E-01	*ARG1, MED23*	3'-UTR	BLD, LIV	STRM, PANC, BLD		Hsf,NF-kappaB
rs17350355	7	18928503	42,505	40,576	G>A	0.38	1.01	0.99	1.03	5.8E-01	*HDAC9*	intronic				7 altered motifs
rs614448	1	117273577	42,312	40,188	A>C	0.28	0.99	0.97	1.02	5.8E-01	*CD2, NEFHP1, GAPDHP64, FTH1P22*			BLD		
rs6691738	1	173152036	42,507	40,577	A>C	0.30	1.01	0.98	1.03	5.8E-01	*TNFSF4, GOT2P2*					7 altered motifs
rs3212217	5	158755130	42,507	40,572	C>G	0.19	1.01	0.98	1.03	5.8E-01	*IL12B, UBLCP1, LOC285626, RNU4ATAC2P*	intronic				FXR,HDAC2
rs9512987	13	28604376	42,493	40,559	G>A	0.21	1.01	0.98	1.03	5.8E-01	*FLT3, PRHOXNB, LOC100420919*	intronic				Ets
rs336082	5	54455439	42,508	40,575	G>A	0.19	0.99	0.97	1.02	5.8E-01	*GZMA, CDC20B, GPX8, MIR449A, MIR449B, MIR449C*	intronic	11 tissues	8 tissues		Irf
rs2781668	6	131897278	42,505	40,577	G>A	0.17	0.99	0.97	1.02	5.8E-01	*ARG1, MED23*	intronic	BLD	4 tissues		Klf4,Pbx3,STAT
rs16923193	9	5512459	42,502	40,567	A>G	0.12	1.01	0.98	1.04	5.8E-01	*CD274, PDCD1LG2*	intronic	11 tissues	9 tissues	8 tissues	
rs4721718	7	18726997	41,994	40,016	C>A	0.17	0.99	0.97	1.02	5.8E-01	*HDAC9*	intronic				Hmbox1,Mef2
rs3744483	17	40466438	42,493	40,554	A>G	0.19	1.01	0.98	1.03	5.8E-01	*STAT3, STAT5A, STAT5B*	3'-UTR		11 tissues	10 tissues	Foxa,p300
rs2506144	10	33468456	42,501	40,575	G>A	0.15	0.99	0.96	1.02	5.8E-01	*NRP1*	3'-UTR	STRM	14 tissues	PLCNT,OVRY	
rs2717548	8	79671067	42,494	40,558	G>A	0.16	0.99	0.97	1.02	5.8E-01	*IL7, ZC2HC1A, PRKRIRP7*	intronic				CEBPB,Foxp1,Nkx3
rs8034362	15	40283797	42,503	40,574	A>C	0.37	0.99	0.97	1.01	5.8E-01	*SRP14, EIF2AK4, H3F3AP1, SRP14-AS1*	intronic				TEF-1
rs9900213	17	40375881	42,485	40,573	C>A	0.16	0.99	0.97	1.02	5.8E-01	*HCRT, STAT5B, KCNH4, GHDC*	intronic				ERalpha-a,HNF4
rs6701726	1	11126941	41,151	38,890	A>G	0.27	1.01	0.98	1.03	5.8E-01	*MTOR, EXOSC10, SRM, MASP2, TARDBP*	intronic		BRST	PLCNT	GR,HES1
rs2282283	1	157648371	42,407	40,480	A>C	0.22	1.01	0.98	1.03	5.8E-01	*FCRL3, SONP1, VDAC1P9*	3'-UTR		BRST		
rs4848304	2	113552737	42,437	40,485	A>G	0.31	0.99	0.97	1.02	5.8E-01	*IL1A, IL1B, CKAP2L*		CRVX, BLD, SKIN	15 tissues	9 tissues	TCF12
rs11913721	22	22210135	42,015	40,002	A>C	0.41	0.99	0.97	1.01	5.8E-01	*MAPK1*	intronic	BLD	17 tissues	5 tissues	
rs10108662	8	39779989	42,502	40,568	C>A	0.33	0.99	0.97	1.02	5.8E-01	*IDO1, IDO2, LOC100420480*	intronic				4 altered motifs
rs73707295	8	128757836	42,507	40,575	A>G	0.11	1.01	0.98	1.04	5.8E-01	*MYC*			7 tissues	5 tissues	5 altered motifs
rs2494751	14	105262961	42,386	40,457	A>G	0.07	0.99	0.95	1.03	5.8E-01	*AKT1, SIVA1, RPS2P4, ADSSL1, LINC00638, ZBTB42, RPS26P49*		7 tissues	18 tissues	25 tissues	4 altered motifs
rs7522061	1	157668390	42,494	40,572	A>G	0.47	0.99	0.97	1.01	5.8E-01	*FCRL2, FCRL3, SONP1, VDAC1P9*	missense	BLD	ESDR, BLD, SPLN		YY1,Znf143
rs1726595	7	18625532	42,478	40,513	G>A	0.47	0.99	0.97	1.01	5.8E-01	*HDAC9, LOC100419901*	intronic				5 altered motifs
rs9507983	13	28620036	42,495	40,554	A>G	0.39	1.01	0.99	1.03	5.8E-01	*FLT3, LOC100420919*	intronic		ESC, BLD		
rs3136558	2	113591275	42,501	40,573	A>G	0.24	0.99	0.97	1.02	5.8E-01	*IL1A, IL1B, LOC100128413*	intronic	BLD	7 tissues	4 tissues	5 altered motifs
rs1633982	7	18613702	42,486	40,525	G>A	0.48	0.99	0.97	1.01	5.8E-01	*HDAC9, LOC100419901*	intronic		6 tissues	BLD,KID	7 altered motifs
rs2776928	10	33574405	42,508	40,577	A>G	0.09	1.01	0.97	1.05	5.8E-01	*NRP1*	intronic		8 tissues		4 altered motifs
rs11879199	19	7975464	42,382	40,512	A>C	0.33	0.99	0.97	1.02	5.8E-01	*ELAVL1, MAP2K7, SNAPC2, TIMM44, LRRC8E, FLJ22184, EVI5L, LOC388499, CTXN1, TGFBR3L*	intronic		IPSC, SPLN	MUS,BLD	E2F,NRSF,Pax-5
rs2804455	10	33669666	42,508	40,571	G>A	0.10	1.01	0.98	1.04	5.9E-01	*NRP1*			4 tissues		6 altered motifs
rs9512985	13	28601621	42,509	40,576	G>A	0.21	1.01	0.98	1.03	5.9E-01	*FLT3, PRHOXNB, LOC100420919*	intronic		ESC, BLD, BRN	THYM,BLD	
rs943451	10	6621773	42,502	40,559	G>A	0.31	0.99	0.97	1.02	5.9E-01	*PRKCQ, PRKCQ-AS1*	intronic	16 tissues	9 tissues	9 tissues	Ets
rs1319013	10	33543929	42,292	40,374	C>A	0.46	1.01	0.99	1.03	5.9E-01	*NRP1*	intronic		11 tissues	4 tissues	STAT
rs7076103	10	6054951	42,507	40,577	G>A	0.15	1.01	0.98	1.04	5.9E-01	*IL2RA, IL15RA*	intronic			OVRY	Smad3,Smad,TCF12
rs11263903	1	36968736	42,470	40,535	A>T	0.28	0.99	0.97	1.02	5.9E-01	*CSF3R, MRPS15*			LIV		
rs7048841	9	5460801	42,397	40,508	A>G	0.49	0.99	0.97	1.01	5.9E-01	*CD274, PLGRKT, PDCD1LG2, LOC100419687*	intronic		BLD		7 altered motifs
rs2491241	13	28613279	42,479	40,550	A>G	0.43	1.01	0.99	1.03	5.9E-01	*FLT3, LOC100420919*	intronic				4 altered motifs
rs4748152	10	6606286	42,506	40,576	A>G	0.12	0.99	0.96	1.02	5.9E-01	*PRKCQ, PRKCQ-AS1*	intronic	BLD	9 tissues	THYM	4 altered motifs
rs11955690	5	35920686	42,500	40,571	G>A	0.43	0.99	0.97	1.01	5.9E-01	*IL7R, UGT3A1, CAPSL, LOC100506406*	intronic		6 tissues		
rs822338	9	5451557	42,492	40,563	A>G	0.27	0.99	0.97	1.02	5.9E-01	*CD274, PLGRKT, LOC100419687*	intronic	13 tissues	6 tissues	6 tissues	Sox
rs2246031	10	6092210	42,503	40,574	G>A	0.17	1.01	0.98	1.04	5.9E-01	*IL2RA, RBM17, RPL32P23*	intronic	BLD, GI	11 tissues	11 tissues	TATA
rs931273	13	43178583	42,504	40,573	G>A	0.13	1.01	0.98	1.04	5.9E-01	*TNFSF11*	intronic				NRSF
rs7214643	17	62398530	42,506	40,575	G>A	0.31	1.01	0.98	1.03	5.9E-01	*PECAM1, RPL31P57*	3'-UTR	LIV	14 tissues	44 tissues	

SNP	Chr	Position[a]	N Cases	N Controls	Allele	MAF	OR[b]	95%CI		*p* -value	Gene annotation	dbSNP functional annotation	Promoter histone marks	Enhancer histone marks	DNAse	Motifs changed
rs10261942	7	18927303	42,509	40,574	C>G	0.29	1.01	0.98	1.03	5.9E-01	*HDAC9*	intronic				SIX5,TATA
rs17461328	4	142573714	42,509	40,577	G>A	0.08	0.99	0.95	1.03	5.9E-01	*IL15*	intronic		11 tissues	15 tissues	Ik-2
rs2854386	2	219027502	42,509	40,576	G>C	0.05	0.99	0.95	1.03	5.9E-01	*CXCR1, CXCR2, HMGB1P9*		BLD	BRST, SKIN		4 altered motifs
rs3917201	14	76429555	42,510	40,575	A>G	0.24	0.99	0.97	1.02	5.9E-01	*TGFB3, TTLL5, IFT43*	intronic				
rs2919921	8	79662336	42,504	40,573	A>T	0.16	0.99	0.97	1.02	5.9E-01	*IL7, ZC2HC1A, PRKRIRP7*	intronic		BLD		EBF,Pax-5
rs10173099	2	191867332	42,490	40,553	G>A	0.32	1.01	0.98	1.03	5.9E-01	*GLS, STAT1, STAT4, LOC100420571*	intronic				
rs4029774	17	40428961	41,984	39,940	A>G	0.30	0.99	0.97	1.02	5.9E-01	*STAT3, STAT5A, STAT5B*		24 tissues		30 tissues	Irf,Pax-5,RXRA
rs72933640	18	60054077	42,509	40,576	G>C	0.10	0.99	0.96	1.02	5.9E-01	*TNFRSF11A, RPL17P44*			5 tissues		NRSF
rs3752985	10	90772834	42,255	40,313	A>C	0.43	1.01	0.99	1.03	5.9E-01	*ACTA2, FAS, FAS-AS1*	intronic				
rs6956139	7	18676842	42,488	40,561	C>A	0.09	1.01	0.98	1.04	5.9E-01	*HDAC9*	intronic				AP-2rep,MZF1::1-4,SP1
rs6461384	7	18825453	42,493	40,545	C>A	0.24	1.01	0.98	1.03	5.9E-01	*HDAC9*	intronic				FXR,HNF4,RFX5
rs13029247	2	191866658	42,507	40,575	A>G	0.30	1.01	0.98	1.03	5.9E-01	*GLS, STAT1, STAT4, LOC100420571*	intronic		7 tissues		PLZF
rs9512982	13	28597839	42,505	40,569	G>A	0.21	1.01	0.98	1.03	5.9E-01	*FLT3, PRHOXNB, LOC100420919*	intronic	LIV	11 tissues	ESDR,ESDR	5 altered motifs
rs6685808	1	92195033	42,504	40,572	G>C	0.39	0.99	0.97	1.02	5.9E-01	*TGFBR3*	intronic		8 tissues	LNG	5 altered motifs
rs1726597	7	18621630	42,505	40,570	A>G	0.22	0.99	0.97	1.02	5.9E-01	*HDAC9, LOC100419901*	intronic		4 tissues		Foxd1,Foxp1
rs4795067	17	26106675	42,505	40,574	A>G	0.35	0.99	0.97	1.02	5.9E-01	*NOS2, LOC645754*	intronic		STRM, SKIN	BLD	Mef2
rs7911500	10	6037726	42,508	40,577	G>A	0.11	0.99	0.96	1.02	5.9E-01	*IL2RA, IL15RA*					
rs9658750	10	90766596	41,888	39,775	A>G	0.16	1.01	0.98	1.04	5.9E-01	*ACTA2, FAS, FAS-AS1*	intronic		BLD	GI	CEBPB,GR,Nkx6-1
rs2491242	13	28594996	42,504	40,571	A>G	0.43	1.01	0.99	1.03	5.9E-01	*FLT3, PRHOXNB, LOC100420919*	intronic				7 altered motifs
rs10858753	12	88910470	42,357	40,423	C>A	0.07	1.01	0.97	1.05	5.9E-01	*KITLG*	intronic				FXR,Gfi1,RORalpha1
rs1926261	1	92162920	42,506	40,575	A>G	0.25	0.99	0.97	1.02	5.9E-01	*TGFBR3*	intronic	BLD	13 tissues	GI,MUS,SKIN	Myc
rs17657829	6	131903131	42,267	40,328	A>G	0.12	1.01	0.98	1.04	5.9E-01	*ARG1, MED23*	intronic		BLD, LIV		5 altered motifs
rs17577980	6	32359821	42,481	40,549	G>A	0.09	0.99	0.96	1.03	5.9E-01	*HLA-DRA, C6orf10, BTNL2, HCG23*	intronic				6 altered motifs
rs340813	3	3124500	42,496	40,563	C>A	0.40	1.01	0.99	1.03	6.0E-01	*IL5RA, TRNT1, CNTN4*	intronic		4 tissues		
rs231727	2	204741550	42,500	40,574	G>A	0.35	0.99	0.97	1.02	6.0E-01	*CTLA4*			BLD		
rs2069778	4	123376135	42,506	40,573	G>A	0.16	0.99	0.97	1.02	6.0E-01	*IL2, ADAD1*	intronic	GI	BLD		Pax-2
rs866730	6	43691873	42,327	40,376	G>A	0.34	1.01	0.98	1.03	6.0E-01	*VEGFA, MRPS18A, LOC100132242*		SKIN, LIV, BLD	16 tissues	20 tissues	
rs1567868	2	219026619	42,508	40,574	A>G	0.05	0.99	0.95	1.03	6.0E-01	*CXCR1, CXCR2, HMGB1P9*		BLD	BLD, SKIN	BLD,BLD	ZBTB33
rs4239242	17	25974258	42,506	40,573	A>G	0.36	1.01	0.98	1.03	6.0E-01	*LGALS9, KSR1, NOS2P1, ITM2BP1, LOC100420408*	intronic		GI, BLD	MUS,BRN	
rs4672875	2	219021385	42,510	40,575	A>C	0.05	0.99	0.95	1.03	6.0E-01	*CXCR1, CXCR2, HMGB1P9*		BLD	BLD		4 altered motifs
rs9512995	13	28614361	42,506	40,576	A>G	0.08	0.99	0.95	1.03	6.0E-01	*FLT3, LOC100420919*	intronic			BLD	
rs17208888	6	32379506	42,509	40,577	G>A	0.09	0.99	0.96	1.03	6.0E-01	*HLA-DRA, HLA-DRB9, C6orf10, BTNL2, HCG23*		GI	GI	4 tissues	5 altered motifs
rs11767043	7	18979366	42,491	40,555	G>A	0.21	1.01	0.98	1.03	6.0E-01	*HDAC9, NPM1P13*	intronic		4 tissues		
rs475002	19	7986638	42,486	40,556	C>G	0.44	1.01	0.99	1.03	6.0E-01	*ELAVL1, MAP2K7, SNAPC2, TIMM44, LRRC8E, FLJ22184, LOC388499, CTXN1, TGFBR3L*	missense	12 tissues	19 tissues	13 tissues	BDP1
rs9512988	13	28605429	42,501	40,572	A>G	0.21	1.01	0.98	1.03	6.0E-01	*FLT3, PRHOXNB, LOC100420919*	intronic		BLD, MUS, SPLN	BLD	Ik-2,VDR,Zfp410
rs2639614	1	172625957	42,509	40,577	G>A	0.14	0.99	0.96	1.02	6.0E-01	*FASLG, SUCO*					
rs805308	2	54194934	42,508	40,575	C>G	0.50	0.99	0.98	1.01	6.0E-01	*PSME4*	intronic	11 tissues	13 tissues	6 tissues	Pax-5
rs1475961	6	32194609	42,330	40,375	A>G	0.38	0.99	0.97	1.02	6.0E-01	*AGER, NOTCH4, PBX2, RNF5, AGPAT1, GPSM3*		BLD	BLD, VAS	BLD	4 altered motifs
rs12722596	10	6056294	42,406	40,485	A>G	0.11	1.01	0.98	1.04	6.0E-01	*IL2RA, IL15RA*	intronic	GI	7 tissues	48 tissues	4 altered motifs
rs3767569	1	92173371	42,507	40,575	G>A	0.17	0.99	0.97	1.02	6.0E-01	*TGFBR3*	intronic		7 tissues	8 tissues	
rs45598737	14	105244239	42,311	40,335	G>A	0.09	0.99	0.96	1.03	6.0E-01	*AKT1, SIVA1, ADSSL1, LINC00638, ZBTB42*	intronic		12 tissues	ESC,LNG	CACD,RREB-1,Spz1
rs2494742	14	105251196	41,814	39,847	C>G	0.08	0.99	0.95	1.03	6.0E-01	*AKT1, SIVA1, ADSSL1, LINC00638, ZBTB42, RPS26P49*	intronic	GI	14 tissues	HRT,LNG	Znf143
rs2023651	7	18636045	42,434	40,472	G>A	0.21	0.99	0.97	1.02	6.0E-01	*HDAC9, LOC100419901*	intronic		ESC		6 altered motifs
rs10795726	10	6030749	42,503	40,574	A>G	0.38	1.01	0.99	1.03	6.0E-01	*IL2RA, IL15RA*					Maf
rs7940405	11	118142666	42,493	40,564	A>G	0.34	0.99	0.97	1.02	6.0E-01	*CD3E, MPZL2, AMICA1, MPZL3*			ESDR, GI, CRVX		5 altered motifs
rs11543651	7	18590659	42,198	40,390	A>G	0.31	0.99	0.97	1.02	6.0E-01	*HDAC9, LOC100419901*	intronic		ESC, BLD, BRN	BRN	7 altered motifs
rs11955657	5	35920564	42,494	40,575	C>A	0.43	0.99	0.97	1.01	6.0E-01	*IL7R, UGT3A1, CAPSL, LOC100506406*	intronic		LNG, FAT, SKIN		
rs9972424	15	40365851	42,467	40,535	A>G	0.34	1.01	0.98	1.03	6.0E-01	*SRP14, BMF, EIF2AK4, SRP14-AS1*		BLD, THYM	BLD, THYM	KID,THYM	
rs12775598	10	97509462	42,509	40,577	A>G	0.05	1.01	0.97	1.06	6.0E-01	*ENTPD1, ENTPD1-AS1*	intronic		BLD	BLD	AP-4,BDP1,Rad21
rs3729508	17	26109030	42,508	40,575	G>A	0.41	1.01	0.99	1.03	6.0E-01	*NOS2, LOC645754*	intronic				Nanog,SP1,STAT
rs1119642	8	79664483	42,506	40,576	G>A	0.16	0.99	0.97	1.02	6.1E-01	*IL7, ZC2HC1A, PRKRIRP7*	intronic				Ik-2,TFIIA,ZEB1
rs2057768	16	27322095	42,508	40,577	G>A	0.30	0.99	0.97	1.02	6.1E-01	*IL4R, NSMCE1, FLJ21408*			5 tissues	8 tissues	GR,NF-E2
rs10435744	9	5486856	42,508	40,576	A>G	0.10	1.01	0.98	1.04	6.1E-01	*CD274, PLGRKT, PDCD1LG2*					7 altered motifs
rs3763311	6	32376176	42,506	40,576	G>A	0.28	1.01	0.98	1.03	6.1E-01	*HLA-DRA, C6orf10, BTNL2, HCG23*		BLD			8 altered motifs
rs6567266	18	59994633	42,506	40,577	G>A	0.18	1.01	0.98	1.03	6.1E-01	*TNFRSF11A, KIAA1468*	intronic	4 tissues	10 tissues	GI	CEBPA,CEBPB,RXRA
rs7801662	7	18955448	42,440	40,496	C>A	0.38	0.99	0.97	1.02	6.1E-01	*HDAC9, NPM1P13*	intronic		4 tissues		12 altered motifs
rs7898286	10	6009462	42,500	40,573	C>A	0.40	0.99	0.97	1.02	6.1E-01	*IL2RA, IL15RA, FBXO18*	intronic		PANC		6 altered motifs

SNP	Chr	Position[a]	N Cases	N Controls	Allele	MAF	OR[b]	95%CI		*p* -value	Gene annotation	dbSNP functional annotation	Promoter histone marks	Enhancer histone marks	DNAse	Motifs changed
rs339286	3	3081544	42,498	40,575	G>A	0.09	0.99	0.96	1.03	6.1E-01	*IL5RA, CNTN4*	intronic		IPSC		
rs7789378	7	19002696	42,501	40,575	C>G	0.20	1.01	0.98	1.03	6.1E-01	*HDAC9, NPM1P13*	intronic				Ik-1
rs822339	9	5453172	42,496	40,564	G>A	0.25	0.99	0.97	1.02	6.1E-01	*CD274, PLGRKT, LOC100419687*	intronic	5 tissues	7 tissues	10 tissues	11 altered motifs
rs12670036	7	19032243	41,890	39,851	A>G	0.11	1.01	0.98	1.04	6.1E-01	*HDAC9, NPM1P13*	intronic				5 altered motifs
rs12607858	18	60095749	42,507	40,574	A>G	0.15	0.99	0.96	1.02	6.1E-01	*ACTBP9, TNFRSF11A, RPL17P44*					Ets
rs475007	11	102669312	42,499	40,569	T>A	0.44	0.99	0.98	1.01	6.1E-01	*MMP1, MMP3, MMP10, CSNK1A1P2, WTAPP1, LOC100421658*	intronic	FAT	10 tissues	5 tissues	Myf,ZEB1
rs7318817	13	28617708	42,501	40,569	A>G	0.39	1.01	0.98	1.03	6.1E-01	*FLT3, LOC100420919*	intronic		ESDR, BLD	BLD	HNF4
rs10815226	9	5459705	42,504	40,572	T>A	0.20	1.01	0.98	1.03	6.1E-01	*CD274, PLGRKT, LOC100419687*	intronic			BLD,BLD	SP2
rs4252330	14	76437039	42,508	40,574	A>G	0.17	0.99	0.97	1.02	6.1E-01	*TGFB3, TTLL5, IFT43, LOC100506576*	intronic		HRT, MUS	KID	Hoxa7
rs10889687	1	67909782	42,431	40,472	G>A	0.16	1.01	0.98	1.04	6.1E-01	*IL12RB2, SERBP1*					5 altered motifs
rs334786	3	3150603	42,346	40,377	G>A	0.35	0.99	0.97	1.02	6.1E-01	*IL5RA, TRNT1, CRBN*	intronic	GI	BLD		4 altered motifs
rs10231651	7	18829788	42,508	40,575	T>A	0.07	0.99	0.95	1.03	6.1E-01	*HDAC9*	intronic				CDP
rs12430881	13	28594802	42,502	40,575	A>G	0.20	1.01	0.98	1.03	6.1E-01	*FLT3, PRHOXNB, LOC100420919*	intronic				
rs7539625	1	67672765	42,508	40,575	G>A	0.28	1.01	0.98	1.03	6.1E-01	*IL23R*	intronic	BLD			Cdc5,Pou5f1,Sox
rs11642583	16	85938595	42,452	40,528	A>C	0.15	1.01	0.98	1.04	6.1E-01	*IRF8*	intronic		5 tissues	PLCNT,BLD,BLD	Sox,TCF4
rs12082710	1	92155337	42,509	40,575	A>G	0.39	0.99	0.97	1.02	6.1E-01	*TGFBR3, HSP90B3P*	intronic	GI, VAS	13 tissues	12 tissues	HDAC2
rs1325798	13	43139049	42,505	40,575	G>A	0.45	1.01	0.99	1.03	6.1E-01	*TNFSF11*	intronic				
rs3024585	16	27359844	42,503	40,570	G>A	0.45	0.99	0.97	1.02	6.1E-01	*IL4R*	intronic		BRST, BLD	BRST	
rs3212220	5	158754195	42,502	40,573	C>A	0.19	1.01	0.98	1.03	6.1E-01	*IL12B, UBLCP1, LOC285626, RNU4ATAC2P*	intronic				Zfp691
rs805401	2	54116003	42,509	40,574	G>A	0.31	0.99	0.97	1.02	6.1E-01	*GPR75, PSME4, GPR75-ASB3, MIR3682*	intronic				12 altered motifs
rs563798	10	6552852	42,419	40,511	G>A	0.14	1.01	0.98	1.04	6.2E-01	*PRKCQ*	intronic		GI, MUS	SKIN	AP-2,ERalpha-a,VDR
rs12194513	6	42037167	42,508	40,577	G>A	0.11	1.01	0.98	1.04	6.2E-01	*CCND3, TAF8, C6orf132*	intronic		HRT		OTX
rs9507984	13	28622924	42,502	40,564	A>G	0.21	1.01	0.98	1.03	6.2E-01	*FLT3, LOC100420919*	intronic	BLD	ESC, IPSC, BLD	BLD,BLD	
rs7213889	17	62398568	42,491	40,567	A>G	0.31	1.01	0.98	1.03	6.2E-01	*PECAM1, RPL31P57*	3'-UTR	LIV	14 tissues	53 tissues	Nkx2,Nkx3
rs12255506	10	6043534	42,506	40,575	G>A	0.15	1.01	0.98	1.04	6.2E-01	*IL2RA, IL15RA*			BLD		Hoxb13,Hoxd10
rs7325635	13	43145319	42,502	40,575	G>A	0.45	1.01	0.99	1.03	6.2E-01	*TNFSF11*	intronic				CACD,p300
rs2069776	4	123371976	42,501	40,574	A>G	0.27	0.99	0.97	1.02	6.2E-01	*IL2, ADAD1*		BLD	BLD	ESC	HNF4,PEBP,RXRA
rs801525	7	18710388	42,508	40,576	G>A	0.11	0.99	0.96	1.02	6.2E-01	*HDAC9*	intronic				5 altered motifs
rs2909459	2	54194431	42,508	40,571	G>A	0.29	0.99	0.97	1.02	6.2E-01	*PSME4*	intronic	ESDR, BRST, BLD	11 tissues	BLD	ATF3
rs7077067	10	6132692	42,488	40,554	G>A	0.50	1.01	0.99	1.03	6.2E-01	*IL2RA, RBM17, RPL32P23*	intronic	19 tissues	17 tissues	PLCNT,LNG	Foxa,Foxj2,HDAC2
rs3212219	5	158754461	42,506	40,576	C>A	0.19	1.01	0.98	1.03	6.2E-01	*IL12B, UBLCP1, LOC285626, RNU4ATAC2P*	intronic				
rs10259495	7	18725683	42,331	40,400	C>G	0.08	1.01	0.97	1.05	6.2E-01	*HDAC9*	intronic			5 tissues	Nanog
rs698855	2	54091688	42,504	40,569	G>A	0.31	0.99	0.97	1.02	6.2E-01	*GPR75, PSME4, ERLEC1, GPR75-ASB3, MIR3682*	3'-UTR				Myc,Nanog,SIX5
rs2887286	1	1156131	42,499	40,564	A>G	0.16	0.99	0.97	1.02	6.2E-01	*TNFRSF4, TNFRSF18, SDF4, UBE2J2, B3GALT6, TTLL10, FAM132A, TTLL10-AS1*	intronic		BLD, ADRL		Nkx2,Sox
rs2980980	18	59946568	42,500	40,565	A>G	0.40	0.99	0.97	1.02	6.2E-01	*TNFRSF11A, KIAA1468*	intronic		IPSC		Spz1
rs407743	19	6642417	42,342	40,422	G>C	0.05	1.01	0.97	1.06	6.2E-01	*C3, TNFSF14, RPL7P50*		BLD	BLD, GI, THYM	BLD,BLD	
rs1332179	9	21420677	42,497	40,560	A>G	0.11	0.99	0.96	1.02	6.2E-01	*IFNA1, IFNA2, IFNA8, IFNA11P, IFNWP19, IFNA12P, MIR31HG, IFNWP2*			LNG, FAT	BLD	STAT
rs13438248	7	18938081	42,199	40,181	A>G	0.17	1.01	0.98	1.03	6.2E-01	*HDAC9*	intronic				Pou3f2
rs10269422	7	18854601	42,490	40,552	T>A	0.33	1.01	0.98	1.03	6.2E-01	*HDAC9*	intronic				7 altered motifs
rs12722517	10	6081040	42,223	40,121	A>G	0.24	0.99	0.97	1.02	6.2E-01	*IL2RA, RBM17, RPL32P23*	intronic		BLD		Foxf1,Foxo,Foxq1
rs1234315	1	173178463	42,503	40,573	G>A	0.46	1.01	0.99	1.03	6.2E-01	*TNFSF4, LOC100506023*			4 tissues	GI,MUS	RAR
rs11264794	1	157647789	42,500	40,570	A>C	0.47	0.99	0.98	1.02	6.2E-01	*FCRL3, SONP1, VDAC1P9*					Maf,NF-AT1
rs586355	10	6558188	42,502	40,576	C>A	0.12	0.99	0.96	1.02	6.2E-01	*PRKCQ*	intronic		BLD, MUS	BLD	
rs9533165	13	43176111	42,503	40,572	G>A	0.30	1.01	0.98	1.03	6.2E-01	*TNFSF11*	intronic		ESDR, STRM, BLD	BLD	
rs4236081	6	42021892	42,502	40,573	G>A	0.44	1.01	0.99	1.03	6.2E-01	*CCND3, TAF8, C6orf132*	intronic	FAT, GI	BLD, GI	GI	
rs305065	16	85973866	42,494	40,571	G>C	0.35	1.01	0.98	1.03	6.2E-01	*IRF8*			4 tissues	BLD	EWSR1-FLI1,HDAC2
rs1726599	7	18648426	42,393	40,442	C>A	0.11	1.01	0.98	1.04	6.2E-01	*HDAC9, LOC100419901*	intronic				NRSF,Nkx2,RORalpha1
rs1411262	9	5459419	42,501	40,574	G>A	0.26	0.99	0.97	1.02	6.2E-01	*CD274, PLGRKT, LOC100419687*	intronic	BLD	7 tissues	16 tissues	9 altered motifs
rs4747826	10	6024313	42,504	40,573	G>A	0.16	1.01	0.98	1.04	6.2E-01	*IL2RA, IL15RA, FBXO18*			BLD		Arid3a,GR
rs13145929	4	123352201	42,499	40,574	A>G	0.27	0.99	0.97	1.02	6.2E-01	*IL2, ADAD1*					Evi-1,Mef2
rs11651728	17	3693400	42,505	40,576	G>C	0.49	1.01	0.99	1.03	6.2E-01	*ITGAE, C17orf85*	intronic		BLD, THYM		Elf5
rs11256448	10	6079479	42,401	40,451	A>G	0.25	0.99	0.97	1.02	6.2E-01	*IL2RA, RPL32P23*	intronic	BLD	4 tissues	4 tissues	Evi-1,GATA,Pou3f2
rs7648642	3	119261375	42,507	40,573	A>C	0.47	1.00	0.98	1.02	6.2E-01	*ADPRH, CD80, CSRP2P, TIMMDC1, POGLUT1*	intronic		BLD	BLD	
rs11992286	8	128766080	42,505	40,574	A>G	0.23	0.99	0.97	1.02	6.3E-01	*MYC, MIR1204*		SKIN, LNG, LIV	13 tissues	ADRL,MUS	NF-I,p300
rs4983559	14	105277209	42,493	40,565	A>G	0.39	1.01	0.98	1.03	6.3E-01	*AKT1, RPS2P4, LINC00638, ZBTB42, RPS26P49*				KID	GR
rs6710479	2	242798018	42,433	40,526	G>A	0.43	1.00	0.98	1.02	6.3E-01	*PDCD1, NEU4, CXXC11, LOC285095*	intronic	BLD	4 tissues		RREB-1,SP1

SNP	Chr	Position[a]	N Cases	N Controls	Allele	MAF	OR[b]	95%CI		*p* -value	Gene annotation	dbSNP functional annotation	Promoter histone marks	Enhancer histone marks	DNAse	Motifs changed
rs1888235	20	44623967	42,503	40,566	G>A	0.14	1.01	0.98	1.04	6.3E-01	*MMP9, SLC12A5, ZNF335, PCIF1, FTLP1, LOC100128028*		LNG	13 tissues	CRVX	8 altered motifs
rs7949206	11	76349080	42,504	40,575	G>A	0.19	1.01	0.98	1.03	6.3E-01	*LRRC32, GUCY2EP*		BLD	10 tissues	9 tissues	4 altered motifs
rs8068149	17	26088855	42,371	40,495	G>A	0.43	1.00	0.98	1.02	6.3E-01	*NOS2, LOC645754*	intronic				4 altered motifs
rs3025040	6	43753051	42,507	40,573	G>A	0.14	0.99	0.97	1.02	6.3E-01	*VEGFA*	3'-UTR		SKIN, HRT, GI		LBP-1,LBP-9
rs8128184	21	34677132	42,509	40,569	G>A	0.05	0.99	0.95	1.03	6.3E-01	*IFNAR1, IFNAR2, IL10RB, USF1P1, IL10RB-AS1*		BLD	BLD, LIV, THYM	7 tissues	7 altered motifs
rs11724582	4	123391464	42,506	40,577	A>G	0.27	0.99	0.97	1.02	6.3E-01	*IL2, ADAD1*					FAC1,Pou2f2
rs10482751	1	218556297	42,503	40,572	G>A	0.31	0.99	0.97	1.02	6.3E-01	*TGFB2, RRP15, RPS26P17, LOC728463*	intronic				Pax-5
rs4742097	9	5455632	42,494	40,567	A>G	0.49	1.00	0.98	1.02	6.3E-01	*CD274, PLGRKT, LOC100419687*	intronic	5 tissues	9 tissues	8 tissues	PU.1,Sox
rs2030171	2	191869163	42,506	40,573	G>A	0.32	1.01	0.98	1.03	6.3E-01	*GLS, STAT1, STAT4, LOC100420571*	intronic		BLD	BLD	NF-E2
rs17095852		114237792	42,432	40,505	A>C	0.15	1.01	0.98	1.04	6.3E-01	*IL13RA2*					COMP1,Mef2
rs1360238	9	5514372	42,489	40,560	A>C	0.32	1.01	0.98	1.03	6.3E-01	*CD274, PDCD1LG2*	intronic	BLD	8 tissues	MUS,BLD,BLD	SP2,STAT,YY1
rs2280234	2	191850099	42,506	40,575	G>A	0.37	1.01	0.98	1.03	6.3E-01	*GLS, STAT1, STAT4, LOC100420571*	intronic		9 tissues		6 altered motifs
rs12439335	15	40245114	42,440	40,507	A>G	0.34	0.99	0.97	1.02	6.3E-01	*GPR176, EIF2AK4, H3F3AP1, LOC100505534*	intronic				p300
rs2765880	1	92139867	42,509	40,576	T>A	0.43	1.00	0.98	1.02	6.3E-01	*TGFBR3, HSP90B3P*			5 tissues	SKIN,ADRL,SKIN	AP-3,Nkx3
rs688853	10	6489773	42,449	40,511	G>A	0.06	0.99	0.95	1.03	6.3E-01	*PRKCQ*	intronic		THYM, MUS	THYM	p53
rs486816	15	40225465	42,504	40,571	A>G	0.27	0.99	0.97	1.02	6.3E-01	*GPR176, EIF2AK4, H3F3AP1, LOC100505534*		12 tissues	13 tissues	6 tissues	Sox
rs62626343	10	6128374	42,505	40,575	C>G	0.11	0.99	0.96	1.02	6.3E-01	*IL2RA, RBM17, RPL32P23*		16 tissues	18 tissues	29 tissues	4 altered motifs
rs729838	20	30263528	42,510	40,575	G>A	0.07	0.99	0.95	1.03	6.3E-01	*BCL2L1, COX4I2*	intronic	9 tissues	19 tissues	15 tissues	Egr-1,TLX1::NFIC
rs2391027	1	92172097	41,693	39,359	G>A	0.41	1.01	0.98	1.03	6.3E-01	*TGFBR3*	intronic		7 tissues	HRT,LNG,BLD	Bcl6b,STAT
rs3763313	6	32376471	42,485	40,557	A>C	0.20	1.01	0.98	1.03	6.3E-01	*HLA-DRA, C6orf10, BTNL2, HCG23*					Bcl6b,STAT
rs648778	10	6534592	42,504	40,570	G>A	0.33	1.01	0.98	1.03	6.3E-01	*PRKCQ*	intronic	BLD, SKIN, BRST	BRST, BLD, THYM	BLD,SKIN,SKIN	7 altered motifs
rs11259403	10	6572225	42,506	40,574	A>G	0.34	1.01	0.98	1.03	6.3E-01	*PRKCQ*	intronic	BLD	ESC, BLD, THYM	BLD,BLD,BLD	
rs9926664	16	85938755	42,508	40,576	A>G	0.15	1.01	0.98	1.04	6.3E-01	*IRF8*	intronic		5 tissues		CCNT2,GATA,TAL1
rs480095	15	40234916	42,502	40,574	C>A	0.26	0.99	0.97	1.02	6.3E-01	*GPR176, EIF2AK4, H3F3AP1, LOC100505534*	intronic		13 tissues	10 tissues	Pax-5
rs9533154	13	43140102	42,498	40,573	G>A	0.45	1.00	0.98	1.03	6.3E-01	*TNFSF11*	intronic				4 altered motifs
rs2532083	4	15881412	42,506	40,577	A>G	0.25	1.01	0.98	1.03	6.3E-01	*CD38, LOC100130067*					4 altered motifs
rs374205	6	32196873	42,474	40,542	A>G	0.08	0.99	0.95	1.03	6.3E-01	*AGER, NOTCH4, PBX2, RNF5, GPSM3*					Zic
rs12699985	7	18854401	42,497	40,563	A>G	0.27	1.01	0.98	1.03	6.3E-01	*HDAC9*	intronic		BRN		LRH1,NF-AT
rs10889677	1	67725120	42,503	40,571	C>A	0.30	1.01	0.98	1.03	6.4E-01	*IL12RB2, IL23R, LOC100130497*	3'-UTR				5 altered motifs
rs12146477	11	102624956	42,485	40,551	A>G	0.27	0.99	0.97	1.02	6.4E-01	*MMP1, MMP8, MMP10, MMP27, WTAPP1, LOC100421658*					
rs2608897	6	131893463	42,485	40,542	G>A	0.31	1.01	0.98	1.03	6.4E-01	*ARG1, MED23*			BLD		7 altered motifs
rs733618	2	204730944	42,510	40,577	A>G	0.08	1.01	0.97	1.05	6.4E-01	*CTLA4*		BLD, GI	BLD, THYM		4 altered motifs
rs4485469	18	59999573	42,508	40,573	A>G	0.45	1.00	0.98	1.02	6.4E-01	*TNFRSF11A, KIAA1468*	intronic	BLD	BLD, GI, VAS	4 tissues	5 altered motifs
rs805404	2	54116800	42,506	40,574	A>C	0.31	0.99	0.97	1.02	6.4E-01	*GPR75, PSME4, GPR75-ASB3, MIR3682*	intronic				Gsc,Obox6
rs2915558	17	3631118	42,501	40,574	A>G	0.32	0.99	0.97	1.02	6.4E-01	*ITGAE, P2RX5, GSG2, P2RX5-TAX1BP3*	intronic		ESDR, BLD, THYM		Evi-1,HNF1
rs12765241	10	90740501	42,425	40,455	G>A	0.13	1.01	0.98	1.04	6.4E-01	*ACTA2, FAS, ACTA2-AS1, FAS-AS1*	intronic	VAS, GI	14 tissues	SKIN,HRT,MUS	SRF
rs805416	2	54125258	42,457	40,497	A>G	0.41	1.00	0.98	1.03	6.4E-01	*GPR75, PSME4, GPR75-ASB3, MIR3682*	intronic				
rs2129974	1	92256928	42,510	40,577	G>A	0.11	0.99	0.96	1.02	6.4E-01	*TGFBR3*	intronic		18 tissues	14 tissues	
rs6060913	20	30326840	42,504	40,575	A>G	0.07	0.99	0.95	1.03	6.4E-01	*BCL2L1, TPX2*		24 tissues		52 tissues	Ik-1
rs2853696	5	158744660	42,510	40,577	G>A	0.19	0.99	0.97	1.02	6.4E-01	*IL12B, UBLCP1, LOC285626, RNU4ATAC2P*	intronic				LUN-1,PU.1,Rhox11
rs4240872	1	154436195	42,500	40,552	A>G	0.25	1.01	0.98	1.03	6.4E-01	*IL6R, TDRD10, SHE, PSMD8P1*	intronic	BLD, CRVX	20 tissues	9 tissues	
rs6638	16	85956044	42,503	40,573	T>A	0.50	1.00	0.98	1.02	6.4E-01	*IRF8*	3'-UTR				Hand1
rs6060531	20	30243358	42,508	40,576	G>A	0.07	0.99	0.95	1.03	6.4E-01	*BCL2L1, ID1, COX4I2, MIR3193*				SKIN,BRST	10 altered motifs
rs591441	10	6511386	42,508	40,577	G>A	0.18	1.01	0.98	1.03	6.4E-01	*PRKCQ*	intronic			BLD	
rs11259372	10	6564883	42,190	40,212	A>C	0.07	1.01	0.97	1.05	6.4E-01	*PRKCQ*	intronic		BLD		Nanog,Pou2f2,Pou3f3
rs17147267	7	22811994	42,213	40,190	C>A	0.17	1.01	0.98	1.03	6.4E-01	*IL6, TOMM7, RPS26P32, LOC541472*			GI, LIV		13 altered motifs
rs8052962	16	27326842	42,485	40,562	C>A	0.38	1.00	0.97	1.02	6.4E-01	*IL4R, NSMCE1, FLJ21408*	intronic	21 tissues	8 tissues	24 tissues	6 altered motifs
rs284168	1	92213235	42,493	40,560	A>G	0.44	1.00	0.98	1.03	6.4E-01	*TGFBR3*	intronic		11 tissues	CRVX	RFX5,XBP-1
rs17140345	7	18977511	42,508	40,577	A>C	0.16	0.99	0.97	1.02	6.4E-01	*HDAC9, NPM1P13*	intronic		ESC, LNG		
rs2781665	6	131893247	42,495	40,574	T>A	0.31	1.01	0.98	1.03	6.4E-01	*ARG1, MED23*			BLD, PANC		Spz1
rs4833830	4	123422563	42,500	40,569	A>G	0.27	0.99	0.97	1.02	6.4E-01	*IL2*		BLD	4 tissues	BLD	Nanog,Pou2f2,TATA
rs16970137	15	40283250	42,474	40,539	G>A	0.36	1.00	0.97	1.02	6.4E-01	*SRP14, EIF2AK4, H3F3AP1, SRP14-AS1*	intronic		ESDR		LRH1,TCF4
rs1178132	7	18771108	42,504	40,575	G>C	0.21	1.01	0.98	1.03	6.4E-01	*HDAC9*	intronic				Zbtb3
rs879577	22	17589209	42,470	40,550	G>A	0.25	1.01	0.98	1.03	6.4E-01	*IL17RA, CECR6, CECR5, CECR7, RPL31P62, LOC100996342*	missense		BLD, PLCNT		4 altered motifs
rs6610650		37636514	42,510	40,575	G>A	0.16	1.01	0.98	1.03	6.4E-01	*CYBB, XK*		BLD	BLD, THYM	BLD,BLD,BLD	
rs3823968	7	18664975	42,507	40,576	A>G	0.10	1.01	0.97	1.04	6.4E-01	*HDAC9, LOC100419901*	intronic			MUS	4 altered motifs
rs6451229	5	35866218	42,480	40,557	A>G	0.40	1.00	0.98	1.03	6.4E-01	*IL7R, CAPSL*	intronic	BLD	BLD		CEBPG,CIZ,Nr2f2
rs4654814	1	23094421	42,497	40,569	G>A	0.29	0.99	0.97	1.02	6.5E-01	*EPHB2, MIR4684*	intronic		ESDR, BRN		Foxa,HDAC2

SNP	Chr	Position[a]	N Cases	N Controls	Allele	MAF	OR[b]	95%CI		*p* -value	Gene annotation	dbSNP functional annotation	Promoter histone marks	Enhancer histone marks	DNAse	Motifs changed
rs2862833	10	90775629	42,505	40,575	A>G	0.43	1.00	0.98	1.03	6.5E-01	*ACTA2, FAS, FAS-AS1, MIR4679-1, MIR4679-2*			BLD		AIRE,STAT,TCF4
rs231726	2	204740866	42,505	40,573	G>A	0.35	1.00	0.97	1.02	6.5E-01	*CTLA4*			BLD		NF-AT1
rs10036474	5	54370231	42,509	40,575	A>G	0.13	0.99	0.96	1.02	6.5E-01	*GZMA, GZMK, CDC20B*					
rs2421047	5	158746307	42,344	40,476	G>A	0.19	1.01	0.98	1.03	6.5E-01	*IL12B, UBLCP1, LOC285626, RNU4ATAC2P*	intronic		BRN		4 altered motifs
rs10811537	9	21408824	41,424	39,037	A>G	0.20	0.99	0.97	1.02	6.5E-01	*IFNA1, IFNA2, IFNA8, IFNA13, IFNA11P, IFNWP19, IFNA12P, MIR31HG, IFNWP2*					5 altered motifs
rs12085107	1	172644222	42,509	40,575	A>T	0.05	1.01	0.97	1.06	6.5E-01	*FASLG*		BLD	9 tissues	ESDR,BLD,BLD	
rs3801983	7	18683672	42,506	40,576	A>G	0.22	0.99	0.97	1.02	6.5E-01	*HDAC9*	intronic		STRM, BRN	SKIN	6 altered motifs
rs11259366	10	6561475	42,503	40,572	G>C	0.49	1.00	0.98	1.02	6.5E-01	*PRKCQ*	intronic		5 tissues		
rs1800794	2	113543273	42,504	40,571	G>A	0.30	0.99	0.97	1.02	6.5E-01	*IL1A, IL1B, CKAP2L*			6 tissues		EWSR1-FLI1,Foxo,STAT
rs1268331	10	6639986	42,506	40,571	A>G	0.36	1.00	0.98	1.03	6.5E-01	*PRKCQ, PRKCQ-AS1*		BLD	BLD	BLD	6 altered motifs
rs6094238	20	44653107	42,486	40,553	G>C	0.23	1.01	0.98	1.03	6.5E-01	*MMP9, SLC12A5, NCOA5, FTLP1, LOC100128028*	intronic			BLD,BLD,LNG	6 altered motifs
rs17086213	13	28584744	42,503	40,570	A>G	0.28	0.99	0.97	1.02	6.5E-01	*CDX2, FLT3, PRHOXNB*	intronic		ESC		Arid5b,NRSF,Sin3Ak-20
rs3816900	15	40302243	42,501	40,571	A>G	0.44	1.00	0.98	1.02	6.5E-01	*SRP14, EIF2AK4, SRP14-AS1*	intronic		5 tissues		FXR,HNF1,RORalpha1
rs62237471	22	22224518	42,503	40,566	T>A	0.19	1.01	0.98	1.03	6.5E-01	*MAPK1, PPM1F*		BLD		BLD	
rs7808621	7	19003032	42,320	40,371	G>A	0.19	1.01	0.98	1.03	6.5E-01	*HDAC9, NPM1P13*	intronic				AIRE,Pitx2
rs4986956	21	34614250	42,508	40,573	A>G	0.08	1.01	0.97	1.05	6.5E-01	*IFNAR2, IL10RB, IL10RB-AS1*					
rs2066795	2	191851897	42,509	40,575	G>A	0.12	1.01	0.98	1.04	6.5E-01	*GLS, STAT1, STAT4, LOC100420571*	intronic		BLD, LIV		12 altered motifs
rs1891467	1	218579985	42,509	40,577	A>G	0.22	0.99	0.97	1.02	6.5E-01	*TGFB2*	intronic				Mrg1::Hoxa9
rs753173	10	30778738	42,503	40,566	A>G	0.17	1.01	0.98	1.03	6.5E-01	*MAP3K8, HNRNPA1P32*		BRN	8 tissues	SKIN,BLD	EWSR1-FLI1,HDAC2,TFII-I
rs1004819	1	67670213	42,494	40,570	G>A	0.28	1.01	0.98	1.03	6.5E-01	*IL23R*	intronic				AIRE,Gfi1
rs9472113	6	43695564	42,391	40,486	C>A	0.26	1.01	0.98	1.03	6.5E-01	*VEGFA, MRPS18A, LOC100132242*			SKIN		MIF-1
rs2494744	14	105255814	42,508	40,576	G>A	0.08	0.99	0.95	1.03	6.5E-01	*AKT1, SIVA1, RPS2P4, ADSSL1, LINC00638, ZBTB42, RPS26P49*	intronic		13 tissues	GI	HES1,NF-I
rs10783002	1	92195601	42,506	40,574	G>A	0.39	1.00	0.98	1.03	6.5E-01	*TGFBR3*	intronic			BLD,BLD	5 altered motifs
rs2314812	17	26110814	42,509	40,575	G>A	0.18	1.01	0.98	1.03	6.5E-01	*NOS2, LOC645754*	intronic		10 tissues		4 altered motifs
rs6058391	20	30279908	42,509	40,577	G>A	0.07	0.99	0.95	1.03	6.5E-01	*BCL2L1, TPX2, COX4I2*	intronic	5 tissues	21 tissues	11 tissues	
rs4537545	1	154418879	42,504	40,572	G>A	0.40	1.00	0.98	1.02	6.5E-01	*IL6R, SHE, PSMD8P1*	intronic	SKIN	14 tissues	18 tissues	
rs4073	4	74606024	42,449	40,512	A>T	0.46	1.00	0.98	1.02	6.5E-01	*IL8*		BLD, SKIN, VAS	12 tissues	CRVX,VAS	5 altered motifs
rs11689629	2	204633075	42,264	40,268	G>A	0.25	0.99	0.97	1.02	6.5E-01	*CD28, KRT18P39, NPM1P33*			BLD		NF-AT,NF-AT1,Sox
rs2268625	14	76439634	42,508	40,575	A>G	0.17	0.99	0.97	1.02	6.5E-01	*TGFB3, TTLL5, IFT43, LOC100506576*	intronic		9 tissues	13 tissues	Osr,Pou2f2
rs7144366	14	25103662	42,496	40,568	G>A	0.40	1.00	0.98	1.02	6.5E-01	*GZMH, GZMB*		BLD	BLD	9 tissues	4 altered motifs
rs12040948	1	206975221	42,508	40,576	G>A	0.36	1.00	0.97	1.02	6.6E-01	*IL10, IL19*	intronic	BLD	FAT, BLD, THYM		GATA
rs4509570	1	154436384	42,507	40,575	G>C	0.25	1.01	0.98	1.03	6.6E-01	*IL6R, TDRD10, SHE, PSMD8P1*	intronic	BLD, CRVX	20 tissues	15 tissues	CTCF,Smad4
rs9554265	13	28685062	42,505	40,563	G>A	0.08	0.99	0.96	1.03	6.6E-01	*FLT3, PAN3, CHCHD2P8, PAN3-AS1, LOC100420919*					
rs634492	10	6535432	42,500	40,571	G>A	0.33	1.00	0.98	1.03	6.6E-01	*PRKCQ*	intronic	SKIN	BRST, BLD, SKIN	6 tissues	CTCF,EBF
rs6798904	3	30658717	42,479	40,535	G>A	0.29	1.00	0.97	1.02	6.6E-01	*TGFBR2*	intronic	BLD	12 tissues	BLD,BLD	
rs17086212	13	28584648	42,497	40,567	A>G	0.28	0.99	0.97	1.02	6.6E-01	*CDX2, FLT3, PRHOXNB*	intronic		ESC		
rs25648	6	43738977	42,418	40,503	G>A	0.17	1.01	0.98	1.03	6.6E-01	*VEGFA*	synonymous	24 tissues	BRN	23 tissues	Nr2f2
rs7565639	2	242797063	42,485	40,558	G>A	0.31	1.00	0.98	1.03	6.6E-01	*PDCD1, NEU4, CXXC11, LOC285095*	intronic	BLD	12 tissues	5 tissues	
rs2279580	15	40300214	42,494	40,560	C>G	0.38	1.00	0.98	1.02	6.6E-01	*SRP14, EIF2AK4, SRP14-AS1*	intronic		4 tissues	MUS	Hand1,KAP1,Nanog
rs11875353	18	60098019	42,505	40,577	C>G	0.14	0.99	0.97	1.02	6.6E-01	*ACTBP9, TNFRSF11A, RPL17P44*					COMP1,HMG-IY
rs805379	2	54092984	42,510	40,576	G>A	0.49	1.00	0.98	1.02	6.6E-01	*GPR75, PSME4, ERLEC1, GPR75-ASB3, MIR3682*	intronic		4 tissues		BATF,Pou3f2,TCF4
rs2520355	7	18854120	42,421	40,464	G>A	0.33	1.00	0.98	1.03	6.6E-01	*HDAC9*	intronic				
rs1076161	7	18715271	42,499	40,571	A>G	0.18	1.01	0.98	1.03	6.6E-01	*HDAC9*	intronic				NF-kappaB
rs4141402	7	18681740	42,507	40,576	G>A	0.09	1.01	0.97	1.04	6.6E-01	*HDAC9*	intronic		ADRL		Mxi1,Pou5f1,SREBP
rs2520354	7	18854050	42,470	40,531	G>A	0.33	1.00	0.98	1.03	6.6E-01	*HDAC9*	intronic				4 altered motifs
rs2965026	7	19012564	42,399	40,442	A>G	0.26	1.01	0.98	1.03	6.6E-01	*HDAC9, NPM1P13*	intronic		BRST, STRM, SKIN	4 tissues	6 altered motifs
rs1034969	12	6573856	42,499	40,573	C>A	0.31	1.00	0.97	1.02	6.6E-01	*CD27, VAMP1, NCAPD2, MRPL51, TAPBPL, PKP2P1, SRP14P1, CD27-AS1, SCARNA10*	3'-UTR			ESDR,THYM,MUS	YY1
rs7884397		37619114	42,506	40,572	G>A	0.16	1.01	0.98	1.03	6.6E-01	*CYBB, XK*			BLD		Pou3f1
rs1887027	10	6113782	42,503	40,569	G>A	0.22	0.99	0.97	1.02	6.6E-01	*IL2RA, RBM17, RPL32P23*		BLD	11 tissues	PLCNT,THYM,BLD	Mrg1::Hoxa9,Pou2f2
rs1010601	5	35908204	42,509	40,573	A>G	0.43	1.00	0.98	1.02	6.6E-01	*IL7R, UGT3A1, CAPSL, LOC100506406*	intronic				11 altered motifs
rs284169	1	92213442	42,506	40,573	G>A	0.44	1.00	0.98	1.02	6.6E-01	*TGFBR3*	intronic		9 tissues		MIZF
rs2268623	14	76439014	42,504	40,576	C>G	0.17	0.99	0.97	1.02	6.6E-01	*TGFB3, TTLL5, IFT43, LOC100506576*	intronic		12 tissues	ESC,ESDR,IPSC	
rs791590	10	6090322	42,498	40,563	T>A	0.17	1.01	0.98	1.03	6.6E-01	*IL2RA, RBM17, RPL32P23*	intronic		BLD, THYM	BLD	Bbx,CEBPB,Hbp1
rs3766186	1	1162435	42,469	40,556	C>A	0.09	1.01	0.97	1.04	6.6E-01	*TNFRSF4, TNFRSF18, SDF4, UBE2J2, B3GALT6, TTLL10, FAM132A, TTLL10-AS1*	intronic		6 tissues		5 altered motifs

SNP	Chr	Position[a]	N Cases	N Controls	Allele	MAF	OR[b]	95%CI		*p* -value	Gene annotation	dbSNP functional annotation	Promoter histone marks	Enhancer histone marks	DNAse	Motifs changed
rs2895332	22	17591089	42,502	40,564	A>G	0.32	1.00	0.98	1.03	6.6E-01	*IL17RA, CECR6, CECR5, CECR5-AS1, RPL31P62, LOC100996342*	3'-UTR		BLD		GR,T3R
rs13210960	6	43768422	42,498	40,576	A>T	0.31	1.00	0.98	1.03	6.6E-01	*VEGFA*			9 tissues	MUS	9 altered motifs
rs56382813	10	6124257	42,504	40,576	G>A	0.22	0.99	0.97	1.02	6.6E-01	*IL2RA, RBM17, RPL32P23*			BLD, THYM		6 altered motifs
rs2834156	21	34616157	42,510	40,574	A>G	0.10	0.99	0.96	1.03	6.6E-01	*IFNAR2, IL10RB, IL10RB-AS1*	intronic		BLD, LIV, THYM		
rs8192914	5	54402970	42,501	40,561	G>A	0.13	0.99	0.96	1.02	6.6E-01	*GZMA, CDC20B*	intronic		BLD		CTCF,Evi-1
rs1144393	11	102669409	42,507	40,576	A>G	0.39	1.00	0.98	1.03	6.6E-01	*MMP1, MMP3, MMP10, CSNK1A1P2, WTAPP1, LOC100421658*	intronic		6 tissues	GI,GI	
rs3218097	6	41905275	42,405	40,465	G>A	0.25	0.99	0.97	1.02	6.6E-01	*BYSL, CCND3, MED20, USP49*	intronic	BLD	13 tissues	BLD	Hic1
rs4024109	5	35919618	42,500	40,564	A>G	0.43	1.00	0.98	1.02	6.6E-01	*IL7R, UGT3A1, CAPSL, LOC100506406*	intronic		8 tissues	9 tissues	
rs2494736	14	105243565	42,484	40,567	A>G	0.06	1.01	0.97	1.05	6.7E-01	*AKT1, SIVA1, ADSSL1, LINC00638, ZBTB42*	intronic		8 tissues		6 altered motifs
rs4347211	1	206978340	42,507	40,575	A>G	0.30	1.00	0.98	1.03	6.7E-01	*IL10, IL19*	intronic	BLD	BLD, GI, CRVX		Zfp691
rs10508300	10	6055124	42,508	40,575	C>G	0.07	0.99	0.95	1.03	6.7E-01	*IL2RA, IL15RA*	intronic			IPSC,MUS	5 altered motifs
rs2498795	14	105243435	41,553	39,364	C>A	0.05	0.99	0.95	1.04	6.7E-01	*AKT1, SIVA1, ADSSL1, LINC00638, ZBTB42*	intronic		8 tissues	PANC	Znf143
rs7239667	18	60029238	42,500	40,564	C>G	0.31	1.00	0.98	1.03	6.7E-01	*TNFRSF11A*	intronic			KID	Irf,Pax-5,RXRA
rs2071277	6	32171683	42,435	40,538	A>G	0.50	1.00	0.98	1.02	6.7E-01	*AGER, NOTCH4, PBX2, RNF5, PPT2, AGPAT1, GPSM3, EGFL8, LOC100507547, PPT2-EGFL8*	intronic		5 tissues		5 altered motifs
rs4366576	13	28678488	42,508	40,573	C>A	0.08	0.99	0.96	1.03	6.7E-01	*FLT3, PAN3, CHCHD2P8, PAN3-AS1, LOC100420919*					5 altered motifs
rs7099537	10	33462578	42,510	40,576	A>G	0.07	1.01	0.97	1.05	6.7E-01	*NRP1*					AIRE,Hmbox1
rs9579159	13	28671724	42,479	40,535	A>G	0.19	0.99	0.97	1.02	6.7E-01	*FLT3, PAN3, CHCHD2P8, PAN3-AS1, LOC100420919*	intronic	BLD	BLD		6 altered motifs
rs3804791	3	3149568	42,479	40,558	A>G	0.35	1.00	0.97	1.02	6.7E-01	*IL5RA, TRNT1, CRBN, CNTN4*	intronic		BLD		
rs11696804	20	44628380	42,423	40,520	A>G	0.41	1.00	0.98	1.03	6.7E-01	*MMP9, SLC12A5, ZNF335, FTLP1, LOC100128028*			5 tissues	BRST,BLD	HNF4
rs2210911	1	157643867	41,877	40,008	A>G	0.50	1.00	0.98	1.02	6.7E-01	*FCRL3, SONP1*					Pou1f1,Sox
rs6973548	7	18697782	42,505	40,574	G>A	0.09	1.01	0.97	1.04	6.7E-01	*HDAC9*	intronic		ESC, IPSC, VAS	IPSC	8 altered motifs
rs6427213	1	169713146	42,507	40,574	A>G	0.14	1.01	0.98	1.04	6.7E-01	*SELE, SELL, METTL18*					Pou3f1
rs2412464	15	40303842	42,506	40,570	A>G	0.44	1.00	0.98	1.02	6.7E-01	*SRP14, EIF2AK4, SRP14-AS1*	intronic	BRN	BRST, MUS, BRN		Sin3Ak-20
rs12170331	22	17598409	42,503	40,572	G>A	0.26	1.00	0.98	1.03	6.7E-01	*IL17RA, CECR6, CECR5, CECR5-AS1, RPL31P62, LOC100996342*	3'-UTR	BLD, BRN	14 tissues	MUS	5 altered motifs
rs1178127	7	18767343	42,502	40,576	A>G	0.25	1.01	0.98	1.03	6.7E-01	*HDAC9*	synonymous		ESDR, MUS	MUS,MUS	Pax-8
rs13280865	8	128781333	41,103	39,296	A>G	0.43	1.00	0.98	1.02	6.7E-01	*MYC, MIR1204*					Ets,Msx-1,Ncx
rs6849238	4	123402770	42,489	40,567	G>A	0.27	1.00	0.97	1.02	6.7E-01	*IL2*			4 tissues	BLD	Cart1,Dlx3,Mef2
rs805328	2	54145761	42,478	40,539	A>G	0.31	1.00	0.97	1.02	6.7E-01	*PSME4*	intronic				AP-1,Evi-1,PLZF
rs6959501	7	18915133	42,509	40,576	A>G	0.11	1.01	0.98	1.04	6.7E-01	*HDAC9*	intronic		FAT		6 altered motifs
rs8177655	10	6015119	42,506	40,573	G>A	0.33	1.00	0.97	1.02	6.7E-01	*IL2RA, IL15RA, FBXO18*	intronic	BLD	4 tissues	BLD	Dobox4
rs10505505	8	128739771	42,510	40,577	A>C	0.06	0.99	0.95	1.03	6.7E-01	*MYC*				7 tissues	Fox,Foxa
rs620736	10	6536308	42,500	40,572	G>A	0.33	1.00	0.98	1.03	6.7E-01	*PRKCQ*	intronic	BLD	6 tissues	IPSC,BLD,BLD	6 altered motifs
rs6984633	8	128707095	42,508	40,577	G>A	0.06	0.99	0.95	1.03	6.7E-01	*MYC*		BLD	ESDR, BLD, GI	BLD	5 altered motifs
rs12778662	10	6149037	42,426	40,511	G>A	0.09	0.99	0.96	1.03	6.7E-01	*IL2RA, PFKFB3, RBM17, RPL32P23, MIR3155A, MIR3155B*	intronic				Foxp3,ZEB1
rs6656929	1	67661041	42,443	40,481	T>A	0.37	1.00	0.98	1.02	6.7E-01	*IL23R*	intronic		ESDR, BLD, THYM		5 altered motifs
rs4845618	1	154400015	42,505	40,576	A>C	0.44	1.00	0.98	1.02	6.7E-01	*IL6R, MRPS33P1, RPSAP17, PSMD8P1*	intronic	GI	19 tissues	5 tissues	HDAC2,Irf,STAT
rs2450252	11	69434272	42,251	40,181	G>A	0.07	1.01	0.97	1.05	6.7E-01	*CCND1, ORAOV1, LOC100996515*					Mrg1::Hoxa9,PLZF
rs10795733	10	6043478	42,504	40,573	A>C	0.49	1.00	0.98	1.02	6.7E-01	*IL2RA, IL15RA*					BCL,EBF,Mef2
rs2291477	1	92178335	42,509	40,573	C>G	0.21	0.99	0.97	1.02	6.7E-01	*TGFBR3*	intronic	4 tissues	11 tissues	4 tissues	Zfp187
rs4553185	1	154410955	42,502	40,576	A>G	0.43	1.00	0.98	1.02	6.7E-01	*IL6R, SHE, PSMD8P1*	intronic		6 tissues		Pax-6,Pou3f4,RORalpha1
rs5917471		37652518	42,504	40,574	G>A	0.42	1.00	0.98	1.02	6.8E-01	*CYBB, DYNLT3*	intronic		BLD		
rs3917187	14	76432136	42,505	40,568	G>A	0.24	0.99	0.97	1.02	6.8E-01	*TGFB3, TTLL5, IFT43, LOC100506576*	intronic		BLD		EBF
rs3739319	8	39785321	42,506	40,567	G>A	0.39	1.00	0.98	1.03	6.8E-01	*IDO1, IDO2, LOC100420480*	intronic		MUS	ESDR	7 altered motifs
rs795346	3	30740289	42,507	40,575	A>G	0.36	1.00	0.98	1.02	6.8E-01	*TGFBR2, GADL1*					NRSF
rs428377	1	92241052	42,473	40,551	G>A	0.12	1.01	0.98	1.04	6.8E-01	*TGFBR3*	intronic		12 tissues		4 altered motifs
rs62215621	20	44761854	42,507	40,575	G>A	0.15	0.99	0.97	1.02	6.8E-01	*CD40, NCOA5, CDH22, RPL13P2*			6 tissues	BLD,MUS,BLD	BATF,CEBPB,Pou5f1
rs13090169	3	3152489	42,506	40,576	G>A	0.12	1.01	0.98	1.04	6.8E-01	*IL5RA, TRNT1, CRBN*		HRT, BLD	12 tissues	ESC,BLD	Nkx2
rs11209026	1	67705958	42,510	40,577	G>A	0.06	1.01	0.97	1.05	6.8E-01	*IL23R, LOC100130497*	missense				GR
rs678815	11	102713777	42,496	40,571	C>G	0.49	1.00	0.98	1.02	6.8E-01	*MMP1, MMP3, MMP12, CSNK1A1P2, WTAPP1, LOC100288111*	intronic		BRST, SKIN	LNG,SKIN,SKIN	Hoxd10,Pou3f2
rs5987839		114241615	42,490	40,555	G>A	0.15	1.01	0.98	1.03	6.8E-01	*IL13RA2*	intronic				Hltf,Hoxa10
rs391525	16	85944439	42,502	40,572	A>G	0.33	1.00	0.98	1.03	6.8E-01	*IRF8*	intronic		7 tissues	BLD,BLD,THYM	Foxa,Pax-5
rs645419	11	102716321	42,502	40,571	A>G	0.49	1.00	0.98	1.02	6.8E-01	*MMP1, MMP3, MMP12, CSNK1A1P2, WTAPP1, LOC100288111*					Hsf,STAT

SNP	Chr	Position[a]	N Cases	N Controls	Allele	MAF	OR[b]	95%CI		*p*-value	Gene annotation	dbSNP functional annotation	Promoter histone marks	Enhancer histone marks	DNAse	Motifs changed
rs3773642	3	30710137	42,505	40,562	G>A	0.18	1.01	0.98	1.03	6.8E-01	*TGFBR2*	intronic	HRT	8 tissues		PPAR,RORalpha1
rs11574790	5	158743846	42,510	40,576	G>A	0.10	0.99	0.96	1.03	6.8E-01	*IL12B, UBLCP1, LOC285626, RNU4ATAC2P*	intronic		IPSC	ESC	
rs2268624	14	76439345	42,455	40,509	C>G	0.17	0.99	0.97	1.02	6.8E-01	*TGFB3, TTLL5, IFT43, LOC100506576*	intronic		11 tissues	14 tissues	CTCF,Egr-1,Rad21
rs8177653	10	6015381	42,301	40,345	A>G	0.34	1.00	0.97	1.02	6.8E-01	*IL2RA, IL15RA, FBXO18*	intronic	BLD	BLD, GI		CIZ,SIX5,TATA
rs4613984	8	39776542	42,506	40,572	G>A	0.05	0.99	0.95	1.04	6.8E-01	*IDO1, IDO2, LOC100420480*	intronic		LNG	4 tissues	ZBRK1
rs1441850	8	79657666	41,249	39,124	A>G	0.27	1.00	0.98	1.03	6.8E-01	*IL7, ZC2HC1A, PRKRIRP7*	intronic				
rs1017101	12	6574301	42,496	40,575	C>A	0.30	1.00	0.97	1.02	6.8E-01	*CD27, VAMP1, NCAPD2, MRPL51, TAPBPL, PKP2P1, SRP14P1, CD27-AS1, SCARNA10*	intronic			BLD,THYM,BLD	Irf
rs2810894	1	92143234	42,249	40,285	G>A	0.34	1.00	0.97	1.02	6.8E-01	*TGFBR3, HSP90B3P*					Zfp740
rs473509	11	102669729	42,496	40,559	G>A	0.42	1.00	0.98	1.02	6.8E-01	*MMP1, MMP3, MMP10, CSNK1A1P2, WTAPP1, LOC100421658*	intronic		5 tissues		
rs877572	3	30726432	42,505	40,574	C>G	0.44	1.00	0.98	1.02	6.8E-01	*TGFBR2, GADL1*	intronic		14 tissues		Sox
rs3918253	20	44639511	42,491	40,564	A>G	0.43	1.00	0.98	1.02	6.8E-01	*MMP9, SLC12A5, ZNF335, FTLP1, LOC100128028*	intronic	4 tissues	14 tissues	10 tissues	
rs4329505	1	154432420	42,504	40,573	A>G	0.16	1.01	0.98	1.03	6.8E-01	*IL6R, TDRD10, SHE, PSMD8P1*	intronic		4 tissues		Gfi1
rs9843942	3	30729636	42,503	40,575	G>A	0.36	1.00	0.98	1.03	6.8E-01	*TGFBR2, GADL1*	intronic		20 tissues	BLD	Brachyury,TBX5
rs10499563	7	22760488	42,029	40,188	A>G	0.22	0.99	0.97	1.02	6.8E-01	*IL6, LOC541472*					Dbx1,Sox
rs11165313	1	92180390	42,509	40,577	G>C	0.21	0.99	0.97	1.02	6.8E-01	*TGFBR3*	intronic		11 tissues	ESDR,BLD	SP2
rs7540516	1	206986545	42,503	40,575	A>C	0.30	1.00	0.98	1.03	6.8E-01	*IL10, IL19*	intronic		BLD		Osf2,RREB-1,SIX5
rs1346907	3	30723470	42,504	40,576	G>A	0.45	1.00	0.98	1.02	6.8E-01	*TGFBR2, GADL1*	intronic	6 tissues	21 tissues	24 tissues	
rs7783171	7	18828796	42,501	40,574	A>C	0.24	1.00	0.98	1.03	6.8E-01	*HDAC9*	intronic				9 altered motifs
rs803121	17	38752382	42,502	40,573	A>G	0.14	0.99	0.97	1.02	6.8E-01	*CCR7, SMARCE1*			FAT, BLD, LIV	BLD,BLD	6 altered motifs
rs17140244	7	18939294	42,148	40,205	C>A	0.27	1.00	0.98	1.03	6.8E-01	*HDAC9, NPM1P13*	intronic				DMRT5,TATA
rs5946040		114247766	42,505	40,574	A>C	0.15	1.01	0.98	1.03	6.8E-01	*IL13RA2*	intronic		FAT, BRST		4 altered motifs
rs2043136	3	30720304	42,504	40,576	A>G	0.25	1.00	0.97	1.02	6.8E-01	*TGFBR2, GADL1*	intronic		8 tissues		10 altered motifs
rs10488	11	102668022	42,505	40,566	G>A	0.06	1.01	0.97	1.05	6.8E-01	*MMP1, MMP3, MMP10, CSNK1A1P2, WTAPP1, LOC100421658*	synonymous			BLD,SKIN	
rs2296123	10	6498799	42,506	40,569	C>G	0.38	1.00	0.98	1.02	6.8E-01	*PRKCQ*	intronic				Pax-4
rs609581	11	69446719	42,507	40,575	G>A	0.44	1.00	0.98	1.02	6.8E-01	*CCND1, ORAOV1, LOC100996515*					4 altered motifs
rs10229062	7	18700490	42,504	40,574	A>G	0.09	1.01	0.97	1.04	6.8E-01	*HDAC9*	intronic			BLD,SKIN	GLI
rs1933437	13	28624294	42,506	40,574	A>G	0.39	1.00	0.98	1.03	6.8E-01	*FLT3, LOC100420919*	missense	4 tissues	7 tissues	BLD,PANC,BLD	GR,Zfp410
rs11567701	5	35859863	42,505	40,572	C>A	0.27	1.00	0.97	1.02	6.8E-01	*IL7R, SPEF2, CAPSL*	intronic	5 tissues	12 tissues	ESDR,THYM	FXR,Gfi1
rs11746138	5	158773779	42,509	40,575	G>A	0.14	1.01	0.98	1.04	6.8E-01	*IL12B, LOC285626, RNU4ATAC2P*	intronic		BLD, SKIN		
rs3804795	3	3130645	42,508	40,577	A>G	0.08	1.01	0.97	1.04	6.9E-01	*IL5RA, TRNT1, CNTN4*	intronic	BLD	BLD, PANC	BLD	AP-1,Lhx3
rs11567699	5	35859011	42,505	40,573	G>C	0.27	1.00	0.97	1.02	6.9E-01	*IL7R, SPEF2, CAPSL*	intronic	8 tissues	10 tissues	4 tissues	NRSF,RP58
rs12592831	15	40302441	42,494	40,565	A>G	0.44	1.00	0.98	1.02	6.9E-01	*SRP14, EIF2AK4, SRP14-AS1*	intronic	MUS	6 tissues	BLD	Myf
rs13072593	3	150288489	42,509	40,571	A>G	0.25	1.00	0.97	1.02	6.9E-01	*SERP1, SELT, EIF2A, LOC677762*	intronic				CCNT2,GATA
rs1800469	19	41860296	42,477	40,561	G>A	0.30	1.00	0.98	1.03	6.9E-01	*BCKDHA, TGFB1, HNRNPUL1, EXOSC5, B9D2, CCDC97, TMEM91*		22 tissues	8 tissues	11 tissues	HNF4,Nkx2
rs4934932	10	33633866	41,993	39,951	G>A	0.08	0.99	0.96	1.03	6.9E-01	*NRP1*			5 tissues	LNG	4 altered motifs
rs9907137	17	76228630	42,464	40,531	G>A	0.32	1.00	0.97	1.02	6.9E-01	*BIRC5, TK1, AFMID, TMEM235, THA1P, LOC100996291*	intronic	ESC, IPSC, BRN	12 tissues	ESC,IPSC,IPSC	6 altered motifs
rs2214090	7	18585173	42,508	40,577	A>G	0.05	1.01	0.97	1.06	6.9E-01	*HDAC9, LOC100419901*	intronic				Pou2f2
rs13040272	20	44633111	42,480	40,538	A>G	0.41	1.00	0.98	1.02	6.9E-01	*MMP9, SLC12A5, ZNF335, FTLP1, LOC100128028*		BLD	9 tissues	4 tissues	5 altered motifs
rs565660	10	6538303	42,367	40,455	C>A	0.33	1.00	0.98	1.03	6.9E-01	*PRKCQ*	intronic			BLD	4 altered motifs
rs1571011	10	90757787	42,506	40,576	A>C	0.41	1.00	0.98	1.02	6.9E-01	*ACTA2, FAS, FAS-AS1*	intronic	BLD	5 tissues	OVRY	4 altered motifs
rs6687620	1	67648460	42,508	40,576	G>A	0.13	1.01	0.98	1.04	6.9E-01	*IL23R*	intronic	BRN			4 altered motifs
rs17299670	10	6470994	42,508	40,577	A>G	0.10	1.01	0.97	1.04	6.9E-01	*PRKCQ*	intronic				
rs9610834	22	38086574	42,507	40,576	G>A	0.40	1.00	0.98	1.02	6.9E-01	*LGALS1, TRIOBP, SH3BP1, PDXP, NOL12*	intronic		BRST, SKIN, GI	4 tissues	KAP1,Pitx2
rs1053924	6	32120715	42,500	40,572	G>A	0.31	1.00	0.98	1.03	6.9E-01	*AGER, ATF6B, NOTCH4, PBX2, RNF5, TNXB, PPT2, AGPAT1, GPSM3, FKBPL, PRRT1, EGFL8, LOC100507547, PPT2-EGFL8*				SKIN,BLD	13 altered motifs
rs17086214	13	28586414	42,499	40,570	G>A	0.28	1.00	0.97	1.02	6.9E-01	*CDX2, FLT3, PRHOXNB*	intronic		LNG, BLD, SKIN		MIF-1,PU.1,RFX5
rs1362846	8	39830700	42,494	40,558	C>A	0.44	1.00	0.98	1.02	6.9E-01	*IDO1, IDO2, LOC100420480, LOC100420944*	intronic				5 altered motifs
rs12693590	2	191858636	42,403	40,439	A>C	0.07	1.01	0.97	1.05	6.9E-01	*GLS, STAT1, STAT4, LOC100420571*	intronic				6 altered motifs
rs2302371	12	6858126	42,501	40,567	G>A	0.13	0.99	0.96	1.02	6.9E-01	*CD4, LAG3, PTMS, MLF2, COPS7A, PIANP*	synonymous		SKIN		E4F1,Pbx3
rs879575	22	17589567	42,446	40,520	G>A	0.24	1.00	0.98	1.03	6.9E-01	*IL17RA, CECR6, CECR5, CECR7, RPL31P62, LOC100996342*	synonymous		BLD, PLCNT		GR
rs10887883	10	90782973	42,505	40,574	G>A	0.40	1.00	0.98	1.02	6.9E-01	*ACTA2, FAS, FAS-AS1, MIR4679-1, MIR4679-2*				SKIN	Hlx1
rs2717344	7	19030853	42,416	40,470	A>G	0.34	1.00	0.98	1.03	6.9E-01	*HDAC9, NPM1P13*	intronic				8 altered motifs
rs16966598	17	26138122	42,500	40,566	G>C	0.15	1.01	0.98	1.03	6.9E-01	*NOS2*		BLD	7 tissues	ESC	Zbtb12

SNP	Chr	Position[a]	N Cases	N Controls	Allele	MAF	OR[b]	95%CI		*p* -value	Gene annotation	dbSNP functional annotation	Promoter histone marks	Enhancer histone marks	DNAse	Motifs changed
rs10950704	7	18836179	42,453	40,527	G>A	0.24	1.00	0.98	1.03	6.9E-01	*HDAC9*	intronic				AP-1,Ncx
rs11079041	17	40414079	41,944	39,820	T>A	0.29	1.00	0.97	1.02	6.9E-01	*STAT5A, STAT5B*	intronic		BLD		
rs903202	16	85947779	42,501	40,573	A>G	0.46	1.00	0.98	1.02	6.9E-01	*IRF8*	intronic		11 tissues	6 tissues	4 altered motifs
rs4750531	10	6534384	42,508	40,567	G>A	0.46	1.00	0.98	1.02	6.9E-01	*PRKCQ*	intronic	SKIN, BRST	4 tissues	9 tissues	SIX5,TCF11::MafG,XBP-1
rs2425761	20	44797562	42,508	40,574	A>G	0.15	1.01	0.98	1.03	6.9E-01	*CD40, CDH22*					Zfp187
rs4733676	8	128706719	42,476	40,555	A>C	0.42	1.00	0.98	1.02	6.9E-01	*MYC*			4 tissues	BLD	SIX5,Znf143
rs3213615	7	18633807	42,197	40,226	A>G	0.31	1.00	0.98	1.03	6.9E-01	*HDAC9, LOC100419901*	intronic				RORalpha1
rs4233292	1	23080351	42,458	40,449	A>G	0.08	1.01	0.97	1.05	6.9E-01	*EPHB2, MIR4684*	intronic	SKIN, BRN, BONE	12 tissues	13 tissues	Pou2f2,Pou6f1
rs3025039	6	43752536	42,503	40,564	G>A	0.14	0.99	0.97	1.02	6.9E-01	*VEGFA*	3'-UTR		SKIN, HRT	BLD	RORalpha1
rs9268528	6	32383108	42,496	40,560	A>G	0.38	1.00	0.98	1.03	6.9E-01	*HLA-DRA, HLA-DRB9, C6orf10, BTNL2, HCG23*			BLD, SKIN, BRN	BLD,BLD	11 altered motifs
rs2799097	1	218524632	42,505	40,565	G>A	0.15	1.01	0.98	1.03	6.9E-01	*TGFB2, RRP15, RPS26P17, LOC728463*	intronic	15 tissues	4 tissues	14 tissues	Foxp1
rs3933239	20	44628668	42,504	40,574	C>A	0.41	1.00	0.98	1.02	6.9E-01	*MMP9, SLC12A5, ZNF335, FTLP1, LOC100128028*		BLD	9 tissues		p53
rs6673027	1	172645898	42,458	40,526	G>A	0.13	0.99	0.96	1.02	6.9E-01	*FASLG*			IPSC, BLD		Mxi1
rs486442	10	6539390	42,471	40,532	C>A	0.47	1.00	0.98	1.02	6.9E-01	*PRKCQ*	intronic		BLD, BRN		Mef2,Pou2f2
rs370703	16	85920480	42,490	40,556	G>A	0.34	1.00	0.98	1.03	7.0E-01	*IRF8*					Ets,Sp100
rs12442731	15	40280693	42,407	40,450	A>G	0.42	1.00	0.98	1.02	7.0E-01	*SRP14, EIF2AK4, H3F3AP1*	intronic				Pax-4,RXR::LXR,Zfp691
rs2111868	2	54186053	42,497	40,563	A>G	0.49	1.00	0.98	1.02	7.0E-01	*PSME4*	intronic		4 tissues		23 altered motifs
rs231775	2	204732714	42,509	40,576	A>G	0.40	1.00	0.98	1.02	7.0E-01	*CTLA4*	missense	BLD, GI, THYM	4 tissues	BLD,BLD	AP-4,Rad21
rs11574674	10	6003451	42,488	40,556	A>G	0.17	0.99	0.97	1.02	7.0E-01	*IL2RA, IL15RA, FBXO18*	intronic		LNG, BRST, SKIN	IPSC	TATA,Zfx
rs805392	2	54104731	42,498	40,566	A>T	0.47	1.00	0.98	1.02	7.0E-01	*GPR75, PSME4, GPR75-ASB3, MIR3682*	intronic		7 tissues		HNF1,Nkx3
rs305077	16	85943466	42,504	40,574	A>G	0.33	1.00	0.98	1.03	7.0E-01	*IRF8*	intronic		5 tissues		4 altered motifs
rs805423	2	54127041	42,504	40,573	G>A	0.49	1.00	0.98	1.02	7.0E-01	*GPR75, PSME4, GPR75-ASB3*	synonymous				GATA,Nanog,p300
rs231747	2	204688432	42,510	40,577	C>A	0.06	0.99	0.95	1.03	7.0E-01	*CTLA4*			BLD		Foxd1,Nkx3,TEF
rs6651524	9	5458575	42,493	40,570	T>A	0.19	1.01	0.98	1.03	7.0E-01	*CD274, PLGRKT, LOC100419687*	intronic		BLD, SKIN, VAS		CIZ,Foxp3
rs2195940	5	158744352	42,508	40,574	G>A	0.10	0.99	0.96	1.03	7.0E-01	*IL12B, UBLCP1, LOC285626, RNU4ATAC2P*	intronic				7 altered motifs
rs12503413	4	76993872	42,489	40,562	G>A	0.09	0.99	0.96	1.03	7.0E-01	*ART3, CXCL10, CXCL11, NUP54, RPL36P8*	intronic	ESC, IPSC	5 tissues	IPSC	5 altered motifs
rs3850245	6	131899504	42,506	40,577	G>A	0.12	1.01	0.98	1.04	7.0E-01	*ARG1, MED23*	intronic		BLD, LIV		39 altered motifs
rs2301950	7	18932510	42,507	40,570	A>T	0.25	1.00	0.98	1.03	7.0E-01	*HDAC9*	intronic				36 altered motifs
rs639752	11	102707339	42,493	40,569	C>A	0.49	1.00	0.98	1.02	7.0E-01	*MMP1, MMP3, MMP12, CSNK1A1P2, WTAPP1, LOC100288111*	intronic	ESC, IPSC	12 tissues	11 tissues	
rs16902328	8	128713873	42,432	40,502	A>C	0.06	0.99	0.95	1.03	7.0E-01	*MYC*				IPSC	
rs11466561	1	92314842	42,504	40,573	A>G	0.07	0.99	0.95	1.03	7.0E-01	*TGFBR3*	intronic		12 tissues		Hltf,SRF
rs6573399	14	62215725	42,506	40,575	C>A	0.13	0.99	0.97	1.02	7.0E-01	*HIF1A, SNAPC1, HIF1A-AS2*		6 tissues	6 tissues	SKIN	
rs11077350	17	76230729	42,423	40,515	A>G	0.46	1.00	0.98	1.02	7.0E-01	*BIRC5, TK1, AFMID, TMEM235, THA1P, LOC100996291*	synonymous	ESDR	11 tissues	IPSC,HRT,THYM	
rs12547643	8	128713173	42,490	40,565	G>A	0.34	1.00	0.98	1.03	7.0E-01	*MYC*					SP1,YY1
rs726116	7	18952086	42,433	40,517	A>G	0.31	1.00	0.97	1.02	7.0E-01	*HDAC9, NPM1P13*	intronic				Bbx,Pou3f3,Sox
rs433322	16	85924572	42,489	40,558	A>G	0.34	1.00	0.98	1.03	7.0E-01	*IRF8*			5 tissues		BDP1,Spz1,WT1
rs10236906	7	18739670	42,416	40,497	G>A	0.10	0.99	0.96	1.03	7.0E-01	*HDAC9*	intronic				7 altered motifs
rs1938	3	119211867	42,504	40,573	G>C	0.29	1.00	0.97	1.02	7.0E-01	*CD80, CSRP2P, TIMMDC1, TMEM39A, POGLUT1*	3'-UTR		BLD		Cdx2,Pax-2
rs7208107	17	40585877	42,510	40,576	A>G	0.09	0.99	0.96	1.03	7.0E-01	*ATP6V0A1, STAT3, PTRF*			LIV	PLCNT	EWSR1-FLI1,Ik-2,Pax-4
rs3801985	7	18670104	42,452	40,507	G>A	0.06	1.01	0.97	1.05	7.0E-01	*HDAC9*	intronic				Arid5b,STAT
rs7820268	8	39777529	42,508	40,573	G>A	0.33	1.00	0.97	1.02	7.0E-01	*IDO1, IDO2, LOC100420480*	intronic				11 altered motifs
rs7630625	3	121889205	41,381	39,527	G>A	0.38	1.00	0.98	1.02	7.1E-01	*CASR, CD86*					6 altered motifs
rs12699991	7	18860760	42,501	40,562	A>G	0.40	1.00	0.98	1.02	7.1E-01	*HDAC9*	intronic			HRT	CEBPB,E2F,GLI
rs67583154	14	105266092	42,500	40,566	G>A	0.14	0.99	0.97	1.02	7.1E-01	*AKT1, SIVA1, RPS2P4, LINC00638, ZBTB42, RPS26P49*		15 tissues	14 tissues	34 tissues	4 altered motifs
rs11805303	1	67675516	42,508	40,576	G>A	0.28	1.00	0.98	1.03	7.1E-01	*IL23R*	intronic	BLD	PANC		Bcl6b,ELF1
rs2073362	21	34620801	42,507	40,573	A>G	0.08	1.01	0.97	1.05	7.1E-01	*IFNAR2, IL10RB, IL10RB-AS1*	intronic		BLD, MUS		6 altered motifs
rs2051920	7	18989232	42,489	40,556	G>A	0.25	1.00	0.97	1.02	7.1E-01	*HDAC9, NPM1P13*	intronic				
rs8030980	15	40278192	42,510	40,574	G>A	0.31	1.00	0.98	1.03	7.1E-01	*SRP14, EIF2AK4, H3F3AP1*	intronic		HRT		TLX1::NFIC
rs7095794	10	6618822	42,250	40,248	A>G	0.32	1.00	0.98	1.03	7.1E-01	*PRKCQ, PRKCQ-AS1*	intronic	BLD, THYM	BLD, THYM	BLD	PLAG1
rs2387346	13	28616629	42,439	40,572	A>T	0.47	1.00	0.98	1.02	7.1E-01	*FLT3, LOC100420919*	intronic		ESC, BLD		5 altered motifs
rs2474710	10	33451379	42,499	40,567	A>G	0.27	1.00	0.97	1.02	7.1E-01	*NRP1*			SKIN, LIV, GI	GI,GI	6 altered motifs
rs3118470	10	6101713	42,488	40,564	A>G	0.33	1.00	0.98	1.03	7.1E-01	*IL2RA, RBM17, RPL32P23*	intronic		BLD	BLD	Pbx-1
rs801539	7	18671212	42,482	40,564	G>C	0.10	1.01	0.97	1.04	7.1E-01	*HDAC9*	intronic		ESC, IPSC		5 altered motifs
rs10252945	7	18924850	42,472	40,553	C>A	0.25	1.00	0.98	1.03	7.1E-01	*HDAC9*	intronic				Ik-3,TCF12
rs2301333	12	6880996	42,505	40,567	G>A	0.06	0.99	0.95	1.04	7.1E-01	*CD4, LAG3, PTMS, MLF2, GPR162, COPS7A*			16 tissues	SKIN,LNG	Eomes,GATA
rs8004244	14	76489451	42,510	40,577	C>A	0.14	1.01	0.98	1.04	7.1E-01	*TGFB3, IFT43, LOC100506576*	intronic		7 tissues	5 tissues	VDR

SNP	Chr	Position[a]	N Cases	N Controls	Allele	MAF	OR[b]	95%CI		*p* -value	Gene annotation	dbSNP functional annotation	Promoter histone marks	Enhancer histone marks	DNAse	Motifs changed
rs2526639	7	19016165	42,497	40,570	A>G	0.26	1.00	0.97	1.02	7.1E-01	*HDAC9, NPM1P13*	intronic		BRST, MUS, SKIN	IPSC	Evi-1,GR,Ik-2
rs7532589	1	92192679	42,509	40,576	C>A	0.28	1.00	0.97	1.02	7.1E-01	*TGFBR3*	intronic		13 tissues	LNG,OVRY	DMRT2,Eomes,Sox
rs2073963	7	18877874	42,487	40,556	A>C	0.40	1.00	0.98	1.02	7.1E-01	*HDAC9*	intronic				
rs17095925		114249548	42,501	40,569	A>G	0.15	1.01	0.98	1.03	7.1E-01	*IL13RA2, LOC100419790*	intronic		FAT		Foxp1,HMG-IY
rs4655108	1	23104384	42,507	40,574	G>A	0.14	0.99	0.97	1.02	7.1E-01	*EPHB2*	intronic		6 tissues	MUS,MUS	Irf
rs2898973	15	40298358	41,922	39,927	G>A	0.30	1.00	0.97	1.02	7.1E-01	*SRP14, EIF2AK4, SRP14-AS1*	intronic		8 tissues	7 tissues	Fox,p300
rs566792	15	40226495	42,475	40,565	G>A	0.14	1.01	0.98	1.03	7.1E-01	*GPR176, EIF2AK4, H3F3AP1, LOC100505534*		24 tissues		48 tissues	4 altered motifs
rs3025036	6	43751669	42,481	40,532	C>G	0.34	1.00	0.98	1.03	7.1E-01	*VEGFA*	intronic		4 tissues	BLD	AP-2rep,CACD,SP1
rs9658727	10	90761865	42,505	40,573	A>G	0.15	1.01	0.98	1.03	7.1E-01	*ACTA2, FAS, FAS-AS1*	intronic	BLD, OVRY	15 tissues	4 tissues	4 altered motifs
rs10159236	1	154431405	42,505	40,573	C>A	0.16	1.01	0.98	1.03	7.1E-01	*IL6R, TDRD10, SHE, PSMD8P1*	intronic				HDAC2
rs11121887	1	12273402	42,490	40,571	G>A	0.38	1.00	0.98	1.02	7.1E-01	*TNFRSF1B, VPS13D, LOC390998, MIR4632*			BLD		YY1
rs334807	3	3129349	42,504	40,570	C>A	0.36	1.00	0.98	1.02	7.1E-01	*IL5RA, TRNT1, CNTN4*	intronic		BLD		
rs9472115	6	43697187	42,505	40,571	A>G	0.18	1.00	0.98	1.03	7.1E-01	*VEGFA, MRPS18A, LOC100132242*			SKIN, GI		Homez
rs489424	15	40233918	41,691	39,553	A>C	0.26	1.00	0.97	1.02	7.1E-01	*GPR176, EIF2AK4, H3F3AP1, LOC100505534*	intronic		9 tissues		4 altered motifs
rs17086226	13	28592546	42,501	40,571	A>G	0.21	1.00	0.97	1.02	7.1E-01	*CDX2, FLT3, PRHOXNB, LOC100420919*	intronic			IPSC	5 altered motifs
rs2491244	13	28599914	42,493	40,565	A>G	0.43	1.00	0.98	1.02	7.1E-01	*FLT3, PRHOXNB, LOC100420919*	intronic		IPSC		6 altered motifs
rs1892847	22	22186538	42,196	40,253	G>A	0.05	0.99	0.95	1.04	7.2E-01	*MAPK1*	intronic		9 tissues	BLD,KID,THYM	HNF4
rs13005843	2	191854726	42,498	40,558	G>A	0.07	1.01	0.97	1.05	7.2E-01	*GLS, STAT1, STAT4, LOC100420571*	intronic		ESDR, BLD, FAT		9 altered motifs
rs2541318	7	18702508	42,499	40,572	G>A	0.11	0.99	0.96	1.03	7.2E-01	*HDAC9*	intronic		4 tissues		13 altered motifs
rs334782	3	3148458	42,444	40,515	A>G	0.42	1.00	0.98	1.02	7.2E-01	*IL5RA, TRNT1, CRBN, CNTN4*	intronic		ESDR		5 altered motifs
rs4627473	18	60061163	42,484	40,554	A>C	0.06	1.01	0.97	1.05	7.2E-01	*ACTBP9, TNFRSF11A, RPL17P44*			GI	ESC,IPSC	HDAC2
rs2917836	15	40239477	40,314	38,460	G>A	0.47	1.00	0.98	1.02	7.2E-01	*GPR176, EIF2AK4, H3F3AP1, LOC100505534*	intronic		6 tissues	ESDR	Pax-5,YY1
rs681803	10	6496922	42,263	40,300	T>A	0.29	1.00	0.98	1.03	7.2E-01	*PRKCQ*	intronic			IPSC	EWSR1-FLI1
rs7549250	1	154404336	42,488	40,567	A>G	0.43	1.00	0.98	1.02	7.2E-01	*IL6R, SHE, PSMD8P1*	intronic		19 tissues	24 tissues	5 altered motifs
rs6814718	4	123423316	42,499	40,574	G>A	0.27	1.00	0.97	1.02	7.2E-01	*IL2*					Gm397,Pou2f2,SRF
rs2046736	1	92175008	42,282	40,370	G>A	0.24	1.00	0.98	1.03	7.2E-01	*TGFBR3*	intronic		4 tissues		CEBPB,Foxa,Pou1f1
rs4474240	1	154457855	42,509	40,577	C>A	0.17	1.00	0.98	1.03	7.2E-01	*IL6R, TDRD10, SHE*	intronic	BLD	11 tissues	13 tissues	
rs34954158	13	43186205	42,508	40,575	A>G	0.12	0.99	0.96	1.03	7.2E-01	*TNFSF11*					
rs2241046	22	17586471	42,503	40,570	A>G	0.22	1.00	0.97	1.02	7.2E-01	*IL17RA, CECR6, CECR5, CECR7, RPL31P62, LOC100996342*	intronic		7 tissues		5 altered motifs
rs486055	11	102650424	42,493	40,563	G>A	0.16	1.00	0.98	1.03	7.2E-01	*MMP1, MMP10, CSNK1A1P2, WTAPP1, LOC100421658*	missense		ESC, ESDR, IPSC	PLCNT	GR,HDAC2
rs6602745	10	6536552	42,508	40,575	G>A	0.13	0.99	0.97	1.02	7.2E-01	*PRKCQ*	intronic	BLD	6 tissues	BLD,BLD	GR,RREB-1
rs2293155	17	40460989	42,507	40,574	A>G	0.17	1.00	0.98	1.03	7.2E-01	*STAT3, STAT5A, STAT5B*	intronic		9 tissues	4 tissues	4 altered motifs
rs12249263	10	6577366	42,464	40,518	G>C	0.22	1.00	0.97	1.02	7.2E-01	*PRKCQ, PRKCQ-AS1*	intronic		BLD	BLD	RORalpha1
rs4845371	1	154408340	42,437	40,488	G>A	0.42	1.00	0.98	1.02	7.2E-01	*IL6R, SHE, PSMD8P1*	intronic	GI	8 tissues	MUS	4 altered motifs
rs11165262	1	92153974	42,503	40,573	A>G	0.20	1.00	0.98	1.03	7.2E-01	*TGFBR3, HSP90B3P*	intronic		9 tissues	BLD,GI	BDP1,HNF4
rs591058	11	102711338	42,501	40,572	A>G	0.49	1.00	0.98	1.02	7.2E-01	*MMP1, MMP3, MMP12, CSNK1A1P2, WTAPP1, LOC100288111*	intronic		GI		4 altered motifs
rs4582902	10	97529318	42,508	40,575	G>A	0.50	1.00	0.98	1.02	7.3E-01	*ENTPD1, ENTPD1-AS1*	intronic		BLD	BLD	6 altered motifs
rs6667434	1	154409100	42,494	40,573	G>A	0.42	1.00	0.98	1.02	7.3E-01	*IL6R, SHE, PSMD8P1*	intronic	GI	8 tissues	4 tissues	BCL,NRSF
rs9906989	17	40455846	42,508	40,574	C>A	0.17	1.00	0.98	1.03	7.3E-01	*STAT3, STAT5A, STAT5B*	intronic	LIV	BLD	BLD	STAT
rs2588637	7	18854186	42,371	40,398	G>A	0.33	1.00	0.98	1.03	7.3E-01	*HDAC9*	intronic				Hoxa10,Maf,PLZF
rs9696950	9	5462245	42,405	40,445	A>T	0.09	0.99	0.96	1.03	7.3E-01	*CD274, PLGRKT, PDCD1LG2, LOC100419687*	intronic				6 altered motifs
rs7947185	11	118214726	42,501	40,571	A>G	0.34	1.00	0.98	1.03	7.3E-01	*CD3D, CD3E, CD3G, UBE4A, LOC100131626*		BLD, GI, THYM	BLD	BLD,THYM,BLD	6 altered motifs
rs116955241	22	22190066	42,504	40,568	A>C	0.05	0.99	0.95	1.04	7.3E-01	*MAPK1*					
rs16845585	1	173164811	42,510	40,576	A>C	0.07	0.99	0.96	1.03	7.3E-01	*TNFSF4, LOC100506023*	intronic	BLD	7 tissues	4 tissues	RXR::LXR,RXRA
rs3918256	20	44640959	42,501	40,574	A>G	0.43	1.00	0.98	1.02	7.3E-01	*MMP9, SLC12A5, NCOA5, ZNF335, FTLP1, LOC100128028*	intronic	GI	10 tissues	ESC,LIV	Klf7
rs3116498	2	204538714	42,510	40,575	T>A	0.07	0.99	0.96	1.03	7.3E-01	*CD28, LOC729532, LOC100287498*			8 tissues	6 tissues	Evi-1,Foxd3,Zfp105
rs3181216	5	158752978	42,501	40,569	T>A	0.32	1.00	0.98	1.02	7.3E-01	*IL12B, UBLCP1, LOC285626, RNU4ATAC2P*	intronic				6 altered motifs
rs11104940	12	88932724	42,410	40,377	A>G	0.10	0.99	0.96	1.03	7.3E-01	*KITLG*	intronic		BRST, GI		HNF1,Pax-2,Pbx-1
rs17094998		114239621	42,503	40,573	A>G	0.15	1.00	0.98	1.03	7.3E-01	*IL13RA2*	intronic				4 altered motifs
rs6432017	2	9693875	42,507	40,572	A>G	0.34	1.00	0.98	1.02	7.3E-01	*ADAM17, YWHAQ*	intronic	6 tissues	17 tissues		E2A,Myf,TAL1
rs2293152	17	40481529	42,492	40,564	C>G	0.39	1.00	0.98	1.02	7.3E-01	*STAT3, STAT5A*	intronic	7 tissues	16 tissues	26 tissues	4 altered motifs
rs4655689	1	67659421	42,463	40,519	A>G	0.13	1.01	0.98	1.04	7.3E-01	*IL23R*	intronic		ESDR, BLD		
rs2269752	7	18699285	42,506	40,577	C>A	0.07	1.01	0.97	1.05	7.3E-01	*HDAC9*	intronic		5 tissues	GI	GR,Sox,TCF12
rs2026430	10	6556041	42,494	40,570	G>A	0.49	1.00	0.98	1.02	7.3E-01	*PRKCQ*	intronic	MUS	BLD, GI, THYM	ESDR,THYM	HDAC2,SETDB1
rs510745	10	6545104	42,510	40,577	G>A	0.11	0.99	0.96	1.03	7.3E-01	*PRKCQ*	intronic	SKIN	4 tissues	BLD	RBP-Jkappa,RFX5,SREBP
rs12632952	3	3137313	42,494	40,562	A>T	0.17	1.00	0.98	1.03	7.3E-01	*IL5RA, TRNT1, CNTN4*	intronic		BLD		Evi-1,Nkx2
rs791587	10	6088699	42,504	40,577	G>A	0.47	1.00	0.98	1.02	7.3E-01	*IL2RA, RBM17, RPL32P23*	intronic	BLD	BLD, THYM	BLD	
rs3135392	6	32409242	42,502	40,574	C>A	0.40	1.00	0.98	1.02	7.3E-01	*HLA-DRA, HLA-DRB9, BTNL2, HCG23*	intronic	6 tissues	BLD, SPLN	BLD	

SNP	Chr	Position[a]	N Cases	N Controls	Allele	MAF	OR[b]	95%CI		*p* -value	Gene annotation	dbSNP functional annotation	Promoter histone marks	Enhancer histone marks	DNAse	Motifs changed
rs73014299	11	118189965	42,509	40,576	G>A	0.11	0.99	0.96	1.03	7.3E-01	*CD3D, CD3E, CD3G, UBE4A*		BLD, SPLN	7 tissues	THYM,BLD	NERF1a,ZBRK1,p300
rs6478974	9	101874403	42,471	40,545	A>T	0.48	1.00	0.98	1.02	7.3E-01	*COL15A1, TGFBR1*	intronic	BLD, GI	15 tissues	SKIN	AP-1,ATF3,ZEB1
rs4842631	12	88926787	42,399	40,510	G>A	0.10	0.99	0.96	1.03	7.3E-01	*KITLG*	intronic				AP-3,Gm397,Pou2f2
rs12723359	1	23137761	42,494	40,561	A>G	0.30	1.00	0.98	1.03	7.3E-01	*EPHB2*	intronic		BLD		4 altered motifs
rs4721719	7	18740090	42,508	40,575	G>A	0.11	0.99	0.96	1.03	7.3E-01	*HDAC9*	intronic			IPSC	SP1,TFII-I,UF1H3BETA
rs11070245	15	40317792	42,507	40,569	C>A	0.47	1.00	0.98	1.02	7.3E-01	*SRP14, EIF2AK4, SRP14-AS1*	intronic				TAL1
rs7802307	7	22766433	42,171	40,158	T>A	0.43	1.00	0.98	1.02	7.3E-01	*IL6, RPS26P32, LOC541472*	intronic	18 tissues	14 tissues	24 tissues	4 altered motifs
rs11607917	11	76418482	42,500	40,571	A>G	0.20	1.00	0.97	1.02	7.3E-01	*LRRC32, GUCY2EP*	intronic		PLCNT		
rs12360225	10	44838832	42,504	40,576	A>G	0.11	0.99	0.96	1.03	7.3E-01	*CXCL12, LOC100130539*					4 altered motifs
rs6592657	11	76377819	42,507	40,577	G>A	0.44	1.00	0.98	1.02	7.3E-01	*LRRC32, GUCY2EP*	intronic	10 tissues	14 tissues	7 tissues	Elf5
rs11258959	10	6500785	42,504	40,576	T>A	0.27	1.00	0.97	1.02	7.3E-01	*PRKCQ*	intronic		BLD, THYM	THYM	4 altered motifs
rs6961943	7	18936474	42,506	40,573	G>A	0.12	0.99	0.97	1.03	7.3E-01	*HDAC9*	intronic		10 tissues	4 tissues	DMRT5
rs1107345	10	6087295	42,509	40,571	C>A	0.23	1.00	0.98	1.03	7.3E-01	*IL2RA, RBM17, RPL32P23*	intronic	BLD	4 tissues	6 tissues	7 altered motifs
rs12576767	11	117915055	42,510	40,577	G>A	0.07	0.99	0.96	1.03	7.3E-01	*IL10RA, TMPRSS4, TMPRSS4-AS1*	intronic		BRST, BLD, THYM		5 altered motifs
rs10489630	1	67662622	42,493	40,562	A>C	0.41	1.00	0.98	1.02	7.3E-01	*IL23R*	intronic				GATA,Tgif1
rs10752169	10	6039371	42,508	40,577	A>G	0.15	1.00	0.98	1.03	7.3E-01	*IL2RA, IL15RA*					26 altered motifs
rs17599586	6	131904719	42,453	40,538	G>A	0.12	1.01	0.97	1.04	7.4E-01	*ARG1, MED23*	intronic				AP-4,LBP-1,Tgif1
rs17184300	6	131905717	42,508	40,573	A>G	0.12	1.01	0.97	1.04	7.4E-01	*ARG1, MED23*	intronic				CEBPA,PTF1-beta,STAT
rs3736290	15	40321351	42,479	40,547	A>C	0.47	1.00	0.98	1.02	7.4E-01	*SRP14, EIF2AK4, SRP14-AS1*	intronic	BRN, VAS	5 tissues	SKIN,OVRY,VAS	Eomes,HNF4,Pax-6
rs9423814	10	6088699	42,508	40,577	G>A	0.47	1.00	0.98	1.02	7.4E-01	*IL2RA, RBM17, RPL32P23*					
rs12124746	1	92210961	42,501	40,575	A>G	0.24	1.00	0.97	1.02	7.4E-01	*TGFBR3*	intronic	FAT, SKIN, LIV	14 tissues	8 tissues	5 altered motifs
rs266094	10	44864300	42,508	40,573	G>A	0.13	0.99	0.97	1.02	7.4E-01	*CXCL12, RPL9P21*			4 tissues	4 tissues	BDP1
rs3917924	1	36945653	42,501	40,569	G>A	0.39	1.00	0.98	1.02	7.4E-01	*CSF3R, MRPS15, OSCP1*	intronic	BLD	4 tissues		BCL,NRSF
rs4934583	10	33543702	42,500	40,574	A>G	0.36	1.00	0.98	1.02	7.4E-01	*NRP1*	intronic		13 tissues	4 tissues	4 altered motifs
rs9554238	13	28643608	41,868	39,979	A>G	0.18	1.00	0.98	1.03	7.4E-01	*FLT3, CHCHD2P8, LOC100420919*	intronic				E4F1,Egr-1
rs2280232	2	191850766	42,497	40,573	A>C	0.25	1.00	0.97	1.02	7.4E-01	*GLS, STAT1, STAT4, LOC100420571*	intronic		5 tissues		11 altered motifs
rs4849123	2	113569039	42,507	40,576	A>G	0.31	1.00	0.98	1.03	7.4E-01	*IL1A, IL1B, CKAP2L, LOC100128413*		BLD	18 tissues		Hand1,Smad3
rs3780395	9	5464552	42,012	40,219	G>A	0.41	1.00	0.98	1.02	7.4E-01	*CD274, PLGRKT, PDCD1LG2, LOC100419687*	intronic		BLD	BLD	TAL1
rs876688	3	30725776	42,497	40,562	G>A	0.38	1.00	0.98	1.02	7.4E-01	*TGFBR2, GADL1*	intronic	FAT	18 tissues		PU.1
rs6963568	7	18836701	42,509	40,566	G>A	0.24	1.00	0.98	1.03	7.4E-01	*HDAC9*	intronic		ESC, IPSC		BDP1,CTCF
rs10833	4	142654547	41,971	40,025	G>A	0.36	1.00	0.98	1.02	7.4E-01	*IL15*	3'-UTR				TATA
rs11610915	12	88902041	42,510	40,576	G>A	0.07	0.99	0.95	1.03	7.4E-01	*KITLG*	intronic		SKIN		BATF,Irf
rs6670338	1	92230305	42,506	40,575	G>A	0.22	1.00	0.98	1.03	7.4E-01	*TGFBR3*	intronic		21 tissues	ESC,HRT,PLCNT	SIX5,Znf143
rs11579207	1	92203190	42,510	40,576	A>G	0.11	1.01	0.97	1.04	7.4E-01	*TGFBR3*	intronic	5 tissues	21 tissues	CRVX	4 altered motifs
rs2277289	11	118182660	42,496	40,556	A>G	0.32	1.00	0.98	1.02	7.4E-01	*CD3D, CD3E, CD3G, UBE4A, MPZL2*	intronic		BLD, THYM, GI	BLD,BLD	Brachyury,PPAR
rs305090	16	85979346	42,461	40,551	G>A	0.37	1.00	0.98	1.02	7.4E-01	*IRF8*		IPSC, BLD, GI	6 tissues	BLD,BLD,BLD	
rs9288066	2	182285695	42,493	40,567	G>C	0.24	1.00	0.97	1.02	7.4E-01	*ITGA4*			ESDR, BLD, THYM	BLD,BLD	TATA
rs2275477	1	36886117	42,500	40,571	G>A	0.21	1.00	0.97	1.02	7.4E-01	*CSF3R, MRPS15, STK40, LSM10, OSCP1*	missense	BLD	16 tissues	12 tissues	Sin3Ak-20,TLX1::NFIC
rs2520456	7	18614519	42,441	40,509	C>A	0.32	1.00	0.98	1.03	7.4E-01	*HDAC9, LOC100419901*	intronic		5 tissues	MUS	
rs1137933	17	26105932	41,881	39,832	G>A	0.22	1.00	0.97	1.02	7.4E-01	*NOS2, LOC645754*	synonymous		7 tissues		ATF3
rs41367851	10	6532004	42,508	40,576	G>A	0.07	1.01	0.97	1.05	7.4E-01	*PRKCQ*	intronic		BLD, THYM		4 altered motifs
rs9838539	3	30728925	42,508	40,576	G>A	0.15	1.00	0.97	1.02	7.4E-01	*TGFBR2, GADL1*	intronic		8 tissues		
rs11104913	12	88911022	42,411	40,469	G>C	0.10	0.99	0.96	1.03	7.4E-01	*KITLG*	intronic		SKIN		Cdx,Mef2,p53
rs8110090	19	41845872	42,506	40,576	A>G	0.05	1.01	0.96	1.05	7.4E-01	*TGFB1, HNRNPUL1, EXOSC5, B9D2, CCDC97, TMEM91*	intronic		5 tissues	MUS,MUS	
rs75727317	10	6499921	42,491	40,559	G>A	0.38	1.00	0.98	1.02	7.4E-01	*PRKCQ*					
rs9884645	4	142686873	42,496	40,547	C>A	0.07	0.99	0.96	1.03	7.5E-01	*IL15*					9 altered motifs
rs2146323	6	43745095	42,441	40,522	C>A	0.35	1.00	0.98	1.02	7.5E-01	*VEGFA*	intronic		12 tissues	MUS,BLD	p53
rs6734469	2	9735900	42,493	40,560	A>G	0.46	1.00	0.98	1.02	7.5E-01	*ADAM17, YWHAQ*	intronic		14 tissues	BLD	GATA
rs4322988	3	3120571	42,509	40,576	C>A	0.12	0.99	0.96	1.03	7.5E-01	*IL5RA, TRNT1, CNTN4*	intronic				PRDM1
rs2236938	3	45938939	42,506	40,573	G>A	0.10	1.01	0.97	1.04	7.5E-01	*CXCR6, CCR9, SDHDP4, FYCO1*	intronic		12 tissues	CRVX,MUS,BRN	9 altered motifs
rs7796691	7	22802407	42,506	40,573	A>G	0.19	1.00	0.97	1.02	7.5E-01	*IL6, TOMM7, RPS26P32, LOC541472*			LNG		13 altered motifs
rs78459945	18	60054757	42,489	40,543	A>G	0.10	1.01	0.97	1.04	7.5E-01	*TNFRSF11A, RPL17P44*			GI	GI,GI	SIX5
rs2272087	17	40459562	42,499	40,570	A>G	0.17	1.00	0.98	1.03	7.5E-01	*STAT3, STAT5A, STAT5B*	intronic	BLD	BLD, SKIN, THYM	OVRY,MUS,BLD	GR,Pbx3
rs2817872	1	23139126	42,505	40,576	G>A	0.30	1.00	0.98	1.03	7.5E-01	*EPHB2*	intronic				
rs10274006	7	18930016	42,502	40,574	G>A	0.26	1.00	0.98	1.03	7.5E-01	*HDAC9*	intronic			15 tissues	18 altered motifs
rs1110258	4	15742716	42,502	40,575	A>G	0.26	1.00	0.97	1.02	7.5E-01	*BST1, CD38, RPL10AP7, LOC100288771*		BLD	LIV		
rs3819250	11	118185346	42,499	40,574	G>A	0.32	1.00	0.98	1.02	7.5E-01	*CD3D, CD3E, CD3G, UBE4A*	intronic		7 tissues	THYM	
rs10892201	11	117848292	42,471	40,550	G>A	0.12	0.99	0.96	1.03	7.5E-01	*IL10RA, TMPRSS13, TMPRSS4-AS1*				THYM	
rs1319462	14	62219225	42,474	40,547	A>G	0.15	1.00	0.98	1.03	7.5E-01	*HIF1A, SNAPC1, HIF1A-AS2*		FAT, BLD	10 tissues	HRT,BLD,SKIN	Mef2,Myb
rs12945695	17	25956348	42,510	40,576	A>C	0.09	1.01	0.97	1.04	7.5E-01	*LGALS9, KSR1, NOS2P1, ITM2BP1, LOC100420408*			4 tissues	SKIN,SKIN	ATF3,BRCA1,Znf143

SNP	Chr	Position[a]	N Cases	N Controls	Allele	MAF	OR[b]	95%CI		p -value	Gene annotation	dbSNP functional annotation	Promoter histone marks	Enhancer histone marks	DNAse	Motifs changed
rs643610	10	6521147	42,508	40,574	G>A	0.19	1.00	0.97	1.02	7.5E-01	*PRKCQ*	intronic				EWSR1-FLI1,Foxp3,HDAC2
rs2292980	16	85945076	42,504	40,572	A>G	0.33	1.00	0.98	1.02	7.5E-01	*IRF8*	intronic		9 tissues	BLD,MUS,BLD	Foxa,RXRA
rs7918373	10	6485722	42,510	40,576	G>A	0.12	1.01	0.97	1.04	7.5E-01	*PRKCQ*	intronic		VAS		Hbp1,VDR
rs2010128	8	79759499	42,500	40,571	G>A	0.41	1.00	0.98	1.02	7.5E-01	*IL7*			BLD		6 altered motifs
rs7975360	12	6605155	42,504	40,575	A>G	0.19	1.00	0.98	1.03	7.5E-01	*CD27, GAPDH, VAMP1, NCAPD2, IFFO1, MRPL51, TAPBPL, PKP2P1, CD27-AS1, SCARNA10, LOC100996356*	intronic	BLD	13 tissues		GR
rs11080344	17	26104511	42,495	40,566	A>G	0.45	1.00	0.98	1.02	7.5E-01	*NOS2, LOC645754*	intronic		9 tissues	4 tissues	Foxo
rs3115968	2	204537170	42,508	40,572	A>G	0.07	0.99	0.96	1.03	7.5E-01	*CD28, LOC729532, LOC100287498*			5 tissues		7 altered motifs
rs16970907	17	76180515	42,504	40,574	G>C	0.06	1.01	0.97	1.05	7.5E-01	*BIRC5, EIF5AP2, TK1, SYNGR2, AFMID, TMC8, TMEM235, C17orf99*	intronic	GI, CRVX	15 tissues	4 tissues	HEN1,Sp4
rs6662887	1	92174914	42,506	40,577	G>A	0.33	1.00	0.98	1.03	7.5E-01	*TGFBR3*	intronic		4 tissues		Cdc5,DMRT3,Irx
rs6427658	1	154400799	42,488	40,562	G>A	0.43	1.00	0.98	1.02	7.5E-01	*IL6R, RPSAP17, PSMD8P1*	intronic	GI	19 tissues	GI,GI	
rs520540	11	102709425	42,047	40,075	A>G	0.49	1.00	0.98	1.02	7.5E-01	*MMP1, MMP3, MMP12, CSNK1A1P2, WTAPP1, LOC100288111*	synonymous		GI		
rs7329160	13	28593787	42,504	40,577	A>G	0.19	1.00	0.98	1.03	7.5E-01	*FLT3, PRHOXNB, LOC100420919*	intronic	LIV	ESC	LIV	7 altered motifs
rs11009326	10	33549849	42,501	40,574	A>G	0.37	1.00	0.98	1.02	7.5E-01	*NRP1*	intronic		10 tissues	7 tissues	Evi-1,HDAC2
rs2980981	18	59947118	42,500	40,571	A>G	0.34	1.00	0.98	1.02	7.5E-01	*TNFRSF11A, KIAA1468*	intronic		ESDR, IPSC, SKIN	ESDR	Mef2
rs12601982	17	40461674	42,501	40,567	A>G	0.17	1.00	0.98	1.03	7.5E-01	*STAT3, STAT5A, STAT5B*	intronic		11 tissues	8 tissues	GCNF,HNF4,RXRA
rs2292982	16	85944823	42,500	40,575	A>C	0.33	1.00	0.98	1.02	7.5E-01	*IRF8*	intronic		8 tissues	5 tissues	
rs12434438	14	62197298	42,332	40,264	A>G	0.20	1.00	0.97	1.02	7.5E-01	*HIF1A, SNAPC1, HIF1A-AS2*	intronic		6 tissues	SKIN,CRVX	HDAC2,Irf
rs7099451	10	6501188	42,502	40,573	G>A	0.27	1.00	0.97	1.02	7.5E-01	*PRKCQ*	intronic		BLD, THYM		7 altered motifs
rs2241044	22	17581838	42,377	40,408	C>A	0.49	1.00	0.98	1.02	7.5E-01	*IL17RA, CECR6, CECR5, CECR7, RPL31P62, LOC100996342*	intronic		BLD, THYM, SPLN		CEBPB
rs3762313	1	67896959	42,484	40,559	A>C	0.39	1.00	0.98	1.02	7.5E-01	*IL12RB2, SERBP1*		24 tissues		40 tissues	
rs17564408	17	62380287	42,507	40,575	A>G	0.11	0.99	0.96	1.03	7.5E-01	*PECAM1, TEX2, RPL31P57*			8 tissues	4 tissues	HNF1,HP1-site-factor,NF-I
rs13053889	22	17580394	42,505	40,574	G>A	0.19	1.00	0.98	1.03	7.5E-01	*IL17RA, CECR6, CECR5, CECR7, RPL31P62, LOC100996342*	intronic		BLD, THYM, SPLN	PLCNT	CACD,Klf7
rs6505469	17	26111886	42,481	40,554	T>A	0.47	1.00	0.98	1.02	7.6E-01	*NOS2, LOC645754*	intronic		LNG	BLD	Rad21
rs4748127	10	6576881	42,432	40,481	C>A	0.22	1.00	0.97	1.02	7.6E-01	*PRKCQ, PRKCQ-AS1*	intronic		BLD		5 altered motifs
rs2280378	16	85952745	42,313	40,368	A>G	0.49	1.00	0.98	1.02	7.6E-01	*IRF8*	intronic		BLD		
rs11466338	19	41845801	42,510	40,576	A>G	0.07	0.99	0.95	1.03	7.6E-01	*TGFB1, HNRNPUL1, EXOSC5, B9D2, CCDC97, TMEM91*	intronic		5 tissues	MUS,MUS	5 altered motifs
rs6683039	1	67631333	42,506	40,574	A>G	0.46	1.00	0.98	1.02	7.6E-01	*IL23R, C1orf141*			BLD		HDAC2
rs7846217	8	39793318	42,505	40,570	A>G	0.13	1.00	0.97	1.03	7.6E-01	*IDO1, IDO2, LOC100420480*	intronic		ESC, IPSC	BLD	GR,SIX5
rs3024509	1	206943297	42,509	40,575	A>G	0.06	0.99	0.95	1.04	7.6E-01	*IL10, MAPKAPK2, IL19*	intronic	4 tissues	8 tissues	BLD,BLD	Brachyury,EBF
rs6691569	1	157648098	42,501	40,576	G>A	0.25	1.00	0.98	1.03	7.6E-01	*FCRL3, SONP1, VDAC1P9*	3'-UTR				9 altered motifs
rs9533173	13	43186976	42,493	40,564	G>A	0.44	1.00	0.98	1.02	7.6E-01	*TNFSF11*			THYM		ZBTB7A
rs8114050	20	44625817	42,489	40,556	G>A	0.41	1.00	0.98	1.02	7.6E-01	*MMP9, SLC12A5, ZNF335, PCIF1, FTLP1, LOC100128028*			6 tissues		Maf
rs7528684	1	157670816	42,501	40,569	A>G	0.44	1.00	0.98	1.02	7.6E-01	*FCRL2, FCRL3, SONP1, VDAC1P9*		BLD	BLD	6 tissues	BCL,NF-kappaB,STAT
rs2372212	3	30721201	42,504	40,574	G>A	0.20	1.00	0.98	1.03	7.6E-01	*TGFBR2, GADL1*	intronic		12 tissues		Hsf,LF-A1
rs7477011	10	6040594	42,510	40,577	A>G	0.49	1.00	0.98	1.02	7.6E-01	*IL2RA, IL15RA*					OTX
rs10752641	1	154432042	42,503	40,566	G>C	0.25	1.00	0.98	1.03	7.6E-01	*IL6R, TDRD10, SHE, PSMD8P1*	intronic				GZF1,Pbx3
rs12923978	16	85946324	42,493	40,550	A>G	0.19	1.00	0.97	1.02	7.6E-01	*IRF8*	intronic		15 tissues	16 tissues	Brachyury,E2A
rs340805	3	3121848	42,492	40,556	G>A	0.30	1.00	0.98	1.03	7.6E-01	*IL5RA, TRNT1, CNTN4*	intronic		ESDR, ESC		6 altered motifs
rs9507991	13	28648151	42,372	40,422	C>A	0.21	1.00	0.97	1.02	7.6E-01	*FLT3, CHCHD2P8, LOC100420919*	intronic				
rs3783515	2	113545834	42,501	40,576	A>G	0.39	1.00	0.98	1.02	7.6E-01	*IL1A, IL1B, CKAP2L*		BLD	BLD, VAS	BLD,BLD	4 altered motifs
rs1130094	2	9630016	42,503	40,571	G>A	0.50	1.00	0.98	1.02	7.6E-01	*ADAM17, CPSF3, IAH1*	3'-UTR		BLD		10 altered motifs
rs2318104	17	3598018	42,496	40,562	G>A	0.19	1.00	0.98	1.03	7.6E-01	*CTNS, ITGAE, P2RX5, TAX1BP3, EMC6, GSG2, RPL21P125, P2RX5-TAX1BP3*	intronic	10 tissues	9 tissues	9 tissues	4 altered motifs
rs6694817	1	154401972	42,510	40,574	G>A	0.43	1.00	0.98	1.02	7.6E-01	*IL6R, SHE, PSMD8P1*	intronic	SKIN	13 tissues	SKIN,MUS,SKIN	
rs3025053	6	43753325	42,432	40,526	G>A	0.12	1.00	0.97	1.04	7.6E-01	*VEGFA*	3'-UTR		HRT, GI, BLD	MUS,BLD	SETDB1,SREBP,STAT
rs4072376	18	59970583	42,507	40,574	G>A	0.15	1.00	0.98	1.03	7.6E-01	*TNFRSF11A, KIAA1468*	intronic				AFP1,Hoxa4,TEF
rs11265618	1	154430092	42,507	40,572	G>A	0.17	1.00	0.98	1.03	7.6E-01	*IL6R, TDRD10, SHE, PSMD8P1*	intronic		BLD, FAT		
rs12413938	10	30794642	42,509	40,571	G>A	0.18	1.00	0.97	1.02	7.6E-01	*MAP3K8, HNRNPA1P32*		BLD	4 tissues	14 tissues	Ets,NF-AT1,TATA
rs12370494	12	88948703	41,954	40,078	A>G	0.10	0.99	0.96	1.03	7.6E-01	*KITLG*	intronic		12 tissues	4 tissues	5 altered motifs
rs9648246	7	18933411	42,502	40,575	G>A	0.39	1.00	0.98	1.02	7.6E-01	*HDAC9*	intronic		HRT, BRST, SKIN		Mef2
rs1386821	1	154382049	42,501	40,573	A>C	0.19	1.00	0.97	1.02	7.6E-01	*IL6R, MRPS33P1, RPSAP17, PSMD8P1*	intronic	BLD, LIV	10 tissues		Nkx6-2
rs6518661	22	17587975	42,496	40,568	G>A	0.35	1.00	0.98	1.02	7.6E-01	*IL17RA, CECR6, CECR5, CECR7, RPL31P62, LOC100996342*	intronic	BLD	BLD, THYM, SPLN	12 tissues	

SNP	Chr	Position[a]	N Cases	N Controls	Allele	MAF	OR[b]	95%CI		*p* -value	Gene annotation	dbSNP functional annotation	Promoter histone marks	Enhancer histone marks	DNAse	Motifs changed
rs5031036	11	102666164	42,505	40,576	A>G	0.06	1.01	0.97	1.05	7.6E-01	*MMP1, MMP3, MMP10, CSNK1A1P2, WTAPP1, LOC100421658*	intronic	SKIN	GI		
rs10127763	1	67651733	42,510	40,577	G>A	0.13	1.00	0.98	1.04	7.6E-01	*IL23R*	intronic				
rs6588247	1	67649344	42,479	40,534	C>A	0.13	1.00	0.97	1.04	7.6E-01	*IL23R*	intronic				4 altered motifs
rs9660710	1	1099342	42,507	40,575	C>A	0.07	1.01	0.97	1.05	7.6E-01	*TNFRSF4, TNFRSF18, C1orf159, LOC254099, TTLL10, MIR200A, MIR200B, MIR429, TTLL10-AS1*		8 tissues	4 tissues		BRCA1,CHD2
rs2869511	1	23140700	42,503	40,574	A>G	0.30	1.00	0.98	1.03	7.6E-01	*EPHB2, MIR4253*	intronic		SKIN, BRN, GI		4 altered motifs
rs7562024	2	191855521	42,504	40,573	G>A	0.40	1.00	0.98	1.02	7.6E-01	*GLS, STAT1, STAT4, LOC100420571*	intronic		MUS		
rs5746062	1	12266612	42,494	40,556	A>G	0.34	1.00	0.98	1.02	7.6E-01	*TNFRSF1B, VPS13D, LOC390998, MIR4632*	intronic		IPSC	BLD	Rad21
rs10482792	1	218605461	42,502	40,574	G>A	0.27	1.00	0.97	1.02	7.6E-01	*TGFB2*	intronic			SKIN,PLCNT	18 altered motifs
rs1561624	1	23101688	42,506	40,577	C>A	0.13	1.00	0.97	1.03	7.6E-01	*EPHB2*	intronic		10 tissues		Pbx-1
rs7782319	7	18923575	41,703	39,396	A>G	0.42	1.00	0.98	1.02	7.7E-01	*HDAC9*	intronic				9 altered motifs
rs384247	6	32184574	42,508	40,575	G>A	0.16	1.00	0.97	1.02	7.7E-01	*AGER, NOTCH4, PBX2, RNF5, AGPAT1, GPSM3, EGFL8, PPT2-EGFL8*	intronic		BLD		4 altered motifs
rs3176886	10	97604406	42,507	40,575	A>G	0.14	1.00	0.97	1.02	7.7E-01	*ENTPD1, ENTPD1-AS1*	intronic				
rs6969258	7	22782528	42,504	40,573	A>G	0.43	1.00	0.98	1.02	7.7E-01	*IL6, RPS26P32, LOC541472*					Pou3f1
rs16970130	15	40281538	42,503	40,576	A>G	0.17	1.00	0.97	1.02	7.7E-01	*SRP14, EIF2AK4, H3F3AP1, SRP14-AS1*	intronic				4 altered motifs
rs987107	5	35875227	42,507	40,574	G>A	0.27	1.00	0.97	1.02	7.7E-01	*IL7R, CAPSL*	intronic		BLD, THYM		Tgif1
rs2210913	1	157668993	42,506	40,575	G>A	0.44	1.00	0.98	1.02	7.7E-01	*FCRL2, FCRL3, SONP1, VDAC1P9*	intronic	BLD	BLD		BCL
rs3793729	10	6522506	42,481	40,562	A>G	0.27	1.00	0.97	1.02	7.7E-01	*PRKCQ*	intronic				CEBPG,Hoxa5
rs12562433	1	92190103	42,506	40,576	C>G	0.44	1.00	0.98	1.02	7.7E-01	*TGFBR3*	intronic		8 tissues		CEBPB,Cdc5,OTX
rs8057456	16	85953196	42,279	40,399	G>A	0.40	1.00	0.98	1.02	7.7E-01	*IRF8*	intronic		ESDR, BLD, SKIN	BLD	
rs6682925	1	67631262	42,493	40,565	A>G	0.46	1.00	0.98	1.02	7.7E-01	*IL23R, C1orf141*			BLD		Hoxa5,Nkx2,Nkx3
rs1886050	10	6523125	42,502	40,576	G>A	0.24	1.00	0.97	1.02	7.7E-01	*PRKCQ*	intronic				MZF1::1-4
rs17257110	14	25107523	42,506	40,575	G>A	0.12	1.00	0.96	1.03	7.7E-01	*GZMH, GZMB*					
rs4742095	9	5445990	41,733	39,712	C>A	0.10	1.01	0.97	1.04	7.7E-01	*CD274, PLGRKT, LOC100419687*			BLD, SKIN, BRST	MUS,BLD,BLD	TCF4,p53
rs755004	3	159700325	42,507	40,576	G>A	0.15	1.00	0.97	1.02	7.7E-01	*IL12A*			ESDR, BRN		SP1
rs1021189	13	43116211	42,502	40,574	A>G	0.47	1.00	0.98	1.02	7.7E-01	*TNFSF11*					4 altered motifs
rs404860	6	32184345	42,494	40,563	A>G	0.16	1.00	0.97	1.02	7.7E-01	*AGER, NOTCH4, PBX2, RNF5, AGPAT1, GPSM3, EGFL8, PPT2-EGFL8*	intronic		BLD		
rs1488369	3	45885360	42,501	40,574	A>G	0.45	1.00	0.98	1.02	7.7E-01	*CCR9, SDHDP4, LZTFL1, SLC6A20*			THYM		5 altered motifs
rs7794273	7	18848534	42,490	40,566	A>G	0.35	1.00	0.98	1.02	7.7E-01	*HDAC9*	intronic				Cphx,YY1,ZID
rs4722166	7	22738762	42,503	40,575	A>C	0.37	1.00	0.98	1.02	7.7E-01	*IL6, LOC401312, LOC541472*			7 tissues	7 tissues	HEY1
rs12146799	12	88962681	42,510	40,577	A>C	0.10	1.00	0.96	1.03	7.7E-01	*KITLG*	intronic		5 tissues		
rs11465597	2	102987213	42,508	40,577	A>G	0.11	1.00	0.96	1.03	7.7E-01	*IL18RAP, IL18R1, IL1RL1, LOC100422339*	intronic				AP-1,Ik-2,NF-AT
rs11104948	12	88943793	42,473	40,540	A>G	0.10	1.00	0.96	1.03	7.7E-01	*KITLG*	intronic		9 tissues	ESDR,MUS,SKIN	CEBPA,CEBPB
rs2228017	3	119263680	42,506	40,576	G>A	0.26	1.00	0.97	1.02	7.7E-01	*ADPRH, CD80, CSRP2P, TIMMDC1*	synonymous		BLD	LNG,BLD	
rs7850895	9	101916076	42,499	40,569	A>G	0.09	1.01	0.97	1.04	7.7E-01	*TGFBR1*	3'-UTR		LNG		Arid5a
rs8177600	10	6025063	42,503	40,575	A>G	0.41	1.00	0.98	1.02	7.7E-01	*IL2RA, IL15RA, FBXO18*					Sox
rs1319107	13	28636312	42,506	40,569	G>A	0.19	1.00	0.97	1.02	7.7E-01	*FLT3, CHCHD2P8, LOC100420919*	intronic				4 altered motifs
rs2200287	13	43168660	42,413	40,473	G>A	0.37	1.00	0.98	1.02	7.7E-01	*TNFSF11*	intronic		11 tissues	9 tissues	Maf,Sp4
rs12403389	1	92208764	42,494	40,569	G>C	0.34	1.00	0.98	1.02	7.7E-01	*TGFBR3*	intronic	GI	17 tissues	20 tissues	DMRT1,DMRT7
rs6461667	7	22796743	42,503	40,574	G>A	0.43	1.00	0.98	1.02	7.7E-01	*IL6, RPS26P32, LOC541472*					BRCA1
rs2297516	17	26095730	42,501	40,571	A>C	0.39	1.00	0.98	1.02	7.8E-01	*NOS2, LOC645754*	intronic				
rs6967723	7	18724797	42,483	40,557	G>A	0.15	1.00	0.97	1.02	7.8E-01	*HDAC9*	intronic		STRM, BLD	LNG	Myf
rs1537947	1	157654663	42,243	40,213	C>G	0.22	1.00	0.98	1.03	7.8E-01	*FCRL3, SONP1, VDAC1P9*	intronic				
rs11466536	3	30735156	42,508	40,575	G>A	0.07	0.99	0.96	1.03	7.8E-01	*TGFBR2, GADL1*	3'-UTR		16 tissues	49 tissues	PRDM1
rs11256433	10	6077845	42,501	40,562	A>C	0.22	1.00	0.98	1.03	7.8E-01	*IL2RA, RPL32P23*	intronic		4 tissues	BLD	
rs17140374	7	18992118	42,475	40,556	A>G	0.17	1.00	0.98	1.03	7.8E-01	*HDAC9, NPM1P13*	intronic				4 altered motifs
rs3822733	5	35875134	42,489	40,567	A>G	0.27	1.00	0.97	1.02	7.8E-01	*IL7R, CAPSL*	intronic		BLD, THYM		BCL,Hoxa3,SRF
rs10827243	10	33633418	42,459	40,544	G>A	0.08	0.99	0.96	1.03	7.8E-01	*NRP1*				ADRL	
rs11152343	18	60086260	42,505	40,575	C>A	0.23	1.00	0.97	1.02	7.8E-01	*ACTBP9, TNFRSF11A, RPL17P44*			BLD	BRST,BLD	Smad
rs79348039	8	128764961	42,109	39,978	A>G	0.22	1.00	0.97	1.02	7.8E-01	*MYC, MIR1204*			ADRL		7 altered motifs
rs432	7	19038332	42,504	40,575	A>G	0.35	1.00	0.98	1.02	7.8E-01	*HDAC9, NPM1P13*			ESC, IPSC, SKIN		
rs3194051	5	35876274	42,493	40,559	A>G	0.27	1.00	0.97	1.02	7.8E-01	*IL7R, CAPSL*	missense		4 tissues	SKIN,SKIN	
rs805391	2	54104318	42,510	40,576	G>A	0.48	1.00	0.98	1.02	7.8E-01	*GPR75, PSME4, GPR75-ASB3, MIR3682*	intronic		ADRL		6 altered motifs
rs4845626	1	154423485	42,508	40,576	C>A	0.17	1.00	0.98	1.03	7.8E-01	*IL6R, SHE, PSMD8P1*	intronic				
rs5277	1	186648197	42,470	40,538	C>G	0.16	1.00	0.98	1.03	7.8E-01	*PTGS2*	synonymous	8 tissues	BRST, BLD	LNG,VAS,SKIN	
rs1321157	1	67654110	42,503	40,575	G>A	0.47	1.00	0.98	1.02	7.8E-01	*IL23R*	intronic		BLD, LIV	GI	Smad
rs677844	1	12244318	42,346	40,423	A>G	0.25	1.00	0.97	1.02	7.8E-01	*TNFRSF8, TNFRSF1B, VPS13D, LOC390998, MIR4632*	intronic	4 tissues	18 tissues	31 tissues	NF-kappaB,p300

SNP	Chr	Position[a]	N Cases	N Controls	Allele	MAF	OR[b]	95%CI		p -value	Gene annotation	dbSNP functional annotation	Promoter histone marks	Enhancer histone marks	DNAse	Motifs changed
rs10089084	8	39769949	42,509	40,575	C>G	0.34	1.00	0.98	1.02	7.8E-01	*IDO1, IDO2, LOC100420480*				CRVX	DMRT2,DMRT3
rs7793526	7	22805608	42,493	40,562	G>A	0.34	1.00	0.98	1.02	7.8E-01	*IL6, TOMM7, RPS26P32, LOC541472*			BLD	BLD,BLD	Pax-4
rs1890995	1	218604678	42,487	40,557	G>A	0.27	1.00	0.97	1.02	7.8E-01	*TGFB2*	intronic		5 tissues	ESDR,OVRY	6 altered motifs
rs7530435	1	92178538	42,496	40,572	G>A	0.45	1.00	0.98	1.02	7.8E-01	*TGFBR3*	intronic	4 tissues	12 tissues	8 tissues	E2F
rs17139630	7	18724512	42,344	40,400	A>C	0.07	1.01	0.97	1.05	7.8E-01	*HDAC9*	intronic		STRM		Foxo,SIX5,Zfp105
rs4750457	10	6482279	42,501	40,571	G>C	0.27	1.00	0.97	1.02	7.8E-01	*PRKCQ*	intronic				Nrf-2
rs3917158	14	76444469	42,507	40,576	G>A	0.17	1.00	0.97	1.02	7.8E-01	*TGFB3, TTLL5, IFT43, LOC100506576*	intronic	PLCNT, CRVX	18 tissues	BLD,THYM,PANC	Irf,Pou2f2,TCF4
rs4737171	8	39807281	42,508	40,575	G>A	0.07	0.99	0.96	1.03	7.8E-01	*IDO1, IDO2, LOC100420480, LOC100420944*	intronic	BLD, LIV	ESC, IPSC, BLD	4 tissues	6 altered motifs
rs4748153	10	6606541	42,508	40,576	G>A	0.11	1.00	0.96	1.03	7.8E-01	*PRKCQ, PRKCQ-AS1*	intronic	BLD	9 tissues	6 tissues	
rs3917925	1	36945559	42,492	40,556	G>A	0.39	1.00	0.98	1.02	7.8E-01	*CSF3R, MRPS15, OSCP1*	intronic	BLD, CRVX	ESDR, BLD, PLCNT	4 tissues	6 altered motifs
rs10506953	12	88900246	42,490	40,549	C>A	0.10	1.00	0.96	1.03	7.8E-01	*KITLG*	intronic				5 altered motifs
rs334809	3	3130221	42,446	40,507	T>A	0.34	1.00	0.98	1.02	7.8E-01	*IL5RA, TRNT1, CNTN4*	intronic		BLD		ZID
rs17189589	2	54209345	42,507	40,575	A>G	0.15	1.00	0.98	1.03	7.8E-01	*PSME4, RPL21P30*	intronic				7 altered motifs
rs17186926	8	128718443	42,503	40,576	A>G	0.21	1.00	0.97	1.02	7.8E-01	*MYC*			GI		CDP
rs45508093	11	118224059	42,505	40,575	A>G	0.18	1.00	0.97	1.02	7.8E-01	*CD3D, CD3E, CD3G, UBE4A, ATP5L, LOC100131626*	3'-UTR		BLD, BRN	34 tissues	9 altered motifs
rs2799083	1	218581617	42,499	40,566	A>G	0.27	1.00	0.97	1.02	7.8E-01	*TGFB2*	intronic		MUS		NF-kappaB
rs2796821	1	218585821	42,506	40,574	G>A	0.27	1.00	0.97	1.02	7.8E-01	*TGFB2*	intronic		9 tissues	6 tissues	12 altered motifs
rs2939421	18	59959991	42,507	40,575	A>G	0.33	1.00	0.98	1.02	7.9E-01	*TNFRSF11A, KIAA1468*	intronic				7 altered motifs
rs6960494	7	18743305	42,506	40,576	A>G	0.12	1.00	0.97	1.03	7.9E-01	*HDAC9*	intronic		HRT		Sox
rs5946039		114244622	42,209	40,322	T>A	0.15	1.00	0.98	1.03	7.9E-01	*IL13RA2*	intronic				9 altered motifs
rs20417	1	186650321	42,502	40,570	C>G	0.15	1.00	0.97	1.02	7.9E-01	*PTGS2*		23 tissues	BLD	10 tissues	10 altered motifs
rs6684961	1	92226827	42,507	40,572	G>A	0.15	1.00	0.97	1.02	7.9E-01	*TGFBR3*	intronic		14 tissues	8 tissues	
rs73684321	7	22774590	42,318	40,264	A>G	0.16	1.00	0.97	1.02	7.9E-01	*IL6, RPS26P32, LOC541472*					7 altered motifs
rs2227306	4	74607055	42,493	40,564	G>A	0.42	1.00	0.98	1.02	7.9E-01	*IL8*	intronic	8 tissues	8 tissues	6 tissues	STAT
rs4934597	10	33614721	42,508	40,577	G>A	0.09	1.00	0.96	1.03	7.9E-01	*NRP1*	intronic	FAT	13 tissues	5 tissues	TCF4
rs4842627	12	88918440	42,499	40,573	C>G	0.10	1.00	0.96	1.03	7.9E-01	*KITLG*	intronic				Hoxd8,RXRA
rs805309	2	54196014	42,505	40,574	G>A	0.50	1.00	0.98	1.02	7.9E-01	*PSME4*	intronic	22 tissues	6 tissues	BRST,VAS	5 altered motifs
rs11104934	12	88927315	42,505	40,573	A>G	0.10	1.00	0.96	1.03	7.9E-01	*KITLG*	intronic				9 altered motifs
rs3812138	6	86199233	42,500	40,569	G>A	0.34	1.00	0.98	1.02	7.9E-01	*NT5E, SNX14*					
rs6667450	1	36957831	42,507	40,573	A>G	0.28	1.00	0.98	1.02	7.9E-01	*CSF3R, MRPS15, OSCP1*		BLD	ESDR, BLD, LIV		TBX5
rs4406737	10	90759724	42,398	40,405	G>A	0.44	1.00	0.98	1.02	7.9E-01	*ACTA2, FAS, FAS-AS1*	intronic	9 tissues	7 tissues	6 tissues	4 altered motifs
rs5746051	1	12261972	42,305	40,479	A>G	0.18	1.00	0.97	1.02	7.9E-01	*TNFRSF1B, VPS13D, LOC390998, MIR4632*	intronic		5 tissues	BLD,BLD,BLD	5 altered motifs
rs2583763	8	79663408	42,507	40,573	G>A	0.10	1.00	0.97	1.04	7.9E-01	*IL7, ZC2HC1A, PRKRIRP7*	intronic				Irx,Pou2f2
rs2029355	1	92181678	42,506	40,572	A>C	0.21	1.00	0.97	1.02	7.9E-01	*TGFBR3*	intronic		5 tissues	LNG	Foxa
rs17131552	1	92224815	42,507	40,568	A>G	0.15	1.00	0.97	1.02	7.9E-01	*TGFBR3*	intronic		10 tissues	4 tissues	BDP1,KAP1
rs3794764	17	26111428	42,508	40,568	G>A	0.21	1.00	0.97	1.02	7.9E-01	*NOS2, LOC645754*	intronic		ESDR, LNG		4 altered motifs
rs7521418	1	92178437	42,501	40,571	G>A	0.45	1.00	0.98	1.02	7.9E-01	*TGFBR3*	intronic	4 tissues	12 tissues	4 tissues	17 altered motifs
rs3136611	10	6021547	42,475	40,520	A>G	0.42	1.00	0.98	1.02	7.9E-01	*IL2RA, IL15RA, FBXO18*					Pou2f2
rs17350236	7	18924289	42,500	40,572	C>A	0.18	1.00	0.98	1.03	7.9E-01	*HDAC9*	intronic			IPSC	
rs2291557	12	88910307	42,489	40,551	G>A	0.10	1.00	0.96	1.03	7.9E-01	*KITLG*	intronic				
rs882643	22	17591823	42,398	40,460	G>C	0.16	1.00	0.98	1.03	7.9E-01	*IL17RA, CECR6, CECR5, CECR5-AS1, RPL31P62, LOC100996342*	3'-UTR		IPSC, BLD		Pax-5
rs12609318	19	6663916	42,506	40,577	G>A	0.25	1.00	0.97	1.02	7.9E-01	*C3, TNFSF14*	3'-UTR	GI	FAT, BLD, LNG	13 tissues	PEBP
rs1358748	1	67636900	42,509	40,576	G>A	0.13	1.00	0.97	1.03	7.9E-01	*IL23R, C1orf141*	intronic	BLD	6 tissues	BLD,BLD	5 altered motifs
rs1492356	12	88921961	42,505	40,572	G>A	0.10	1.00	0.96	1.03	7.9E-01	*KITLG*	intronic				STAT,YY1
rs9729550	1	1135242	42,443	40,528	A>C	0.26	1.00	0.97	1.02	7.9E-01	*TNFRSF4, TNFRSF18, SDF4, B3GALT6, TTLL10, FAM132A, MIR200A, MIR200B, MIR429, TTLL10-AS1*		BLD	8 tissues		Ets,NRSF
rs6492925	15	40304170	42,509	40,576	C>A	0.08	1.00	0.97	1.04	7.9E-01	*SRP14, EIF2AK4, SRP14-AS1*	intronic		BRN		
rs1126647	4	74609045	41,136	39,202	A>T	0.42	1.00	0.98	1.02	7.9E-01	*IL8*	3'-UTR	BRST			11 altered motifs
rs12661819	6	41898564	42,501	40,570	G>A	0.24	1.00	0.98	1.03	7.9E-01	*BYSL, CCND3, MED20, USP49*	intronic		BLD		Pbx3,Zfp410
rs2519891	7	18632130	42,369	40,408	T>A	0.24	1.00	0.98	1.03	8.0E-01	*HDAC9, LOC100419901*	intronic				Cdx2,SRF
rs11567751	5	35871684	42,506	40,574	G>A	0.27	1.00	0.97	1.02	8.0E-01	*IL7R, CAPSL*	intronic		BLD, THYM		DMRT7,En-1
rs6694899	1	23138844	42,478	40,562	A>G	0.30	1.00	0.98	1.02	8.0E-01	*EPHB2*	intronic				Nkx3
rs1178121	7	18762652	42,398	40,498	C>A	0.22	1.00	0.98	1.03	8.0E-01	*HDAC9*	intronic				6 altered motifs
rs11104907	12	88893259	42,510	40,577	G>A	0.10	1.00	0.96	1.03	8.0E-01	*KITLG*	intronic		ADRL	ADRL	HNF4
rs8176984	10	30731660	42,510	40,576	C>A	0.09	1.00	0.96	1.03	8.0E-01	*CCND3P, MAP3K8*	intronic		5 tissues	BLD	6 altered motifs
rs6684439	1	154395839	42,505	40,573	G>A	0.40	1.00	0.98	1.02	8.0E-01	*IL6R, MRPS33P1, RPSAP17, PSMD8P1*	intronic	MUS, SKIN	18 tissues	MUS,BLD	5 altered motifs
rs7532161	1	67642223	42,482	40,553	G>A	0.43	1.00	0.98	1.02	8.0E-01	*IL23R, C1orf141*	intronic				4 altered motifs
rs7650774	3	119205050	42,510	40,571	A>G	0.18	1.00	0.97	1.02	8.0E-01	*CD80, CSRP2P, TIMMDC1, TMEM39A, POGLUT1*	intronic				Zbtb12

SNP	Chr	Position[a]	N Cases	N Controls	Allele	MAF	OR[b]	95%CI		*p* -value	Gene annotation	dbSNP functional annotation	Promoter histone marks	Enhancer histone marks	DNAse	Motifs changed
rs12030415	1	117731972	42,504	40,571	G>A	0.28	1.00	0.98	1.02	8.0E-01	*VTCN1, LOC100506478*	intronic		4 tissues		En-1,Hoxa3,Nkx2
rs11104941	12	88933341	42,509	40,570	G>A	0.10	1.00	0.96	1.03	8.0E-01	*KITLG*	intronic				4 altered motifs
rs2106362	7	18725261	42,493	40,559	C>G	0.18	1.00	0.98	1.03	8.0E-01	*HDAC9*	intronic		STRM	BRN	
rs11998154	8	79713245	42,508	40,576	G>A	0.10	1.00	0.96	1.03	8.0E-01	*IL7, PRKRIRP7*	intronic				CTCF
rs679620	11	102713620	42,395	40,455	A>G	0.49	1.00	0.98	1.02	8.0E-01	*MMP1, MMP3, MMP12, CSNK1A1P2, WTAPP1, LOC100288111*	missense		BRST, SKIN		p300
rs7998462	13	28674026	42,506	40,571	A>G	0.19	1.00	0.97	1.02	8.0E-01	*FLT3, PAN3, CHCHD2P8, PAN3-AS1, LOC100420919*	intronic	12 tissues	11 tissues	ESC,BLD	Foxd1,RXRA
rs10491434	5	35877914	42,504	40,573	A>G	0.27	1.00	0.97	1.02	8.0E-01	*IL7R, CAPSL*				5 tissues	ERalpha-a,HNF1,RXRA
rs11807271	1	92155615	42,494	40,552	A>G	0.20	1.00	0.98	1.03	8.0E-01	*TGFBR3, HSP90B3P*	intronic	4 tissues	14 tissues	5 tissues	Myb,Pax-6,Smad
rs2279627	19	6663594	42,508	40,575	C>G	0.25	1.00	0.97	1.02	8.0E-01	*C3, TNFSF14*	3'-UTR		BLD, FAT, GI	7 tissues	TBX5
rs4404254	2	204825286	42,503	40,568	A>G	0.24	1.00	0.97	1.02	8.0E-01	*ICOS*	3'-UTR		STRM, VAS, MUS		
rs11104921	12	88919483	42,502	40,572	A>G	0.10	1.00	0.96	1.03	8.0E-01	*KITLG*	intronic				BCL,NRSF,Sin3Ak-20
rs17131554	1	92228208	42,509	40,576	A>G	0.15	1.00	0.97	1.02	8.0E-01	*TGFBR3*	intronic		11 tissues	PLCNT	5 altered motifs
rs41294657	10	6048060	41,740	40,010	A>C	0.08	1.00	0.97	1.04	8.0E-01	*IL2RA, IL15RA*			6 tissues	ESC,IPSC	5 altered motifs
rs11104906	12	88891776	42,509	40,574	T>A	0.10	1.00	0.96	1.03	8.0E-01	*KITLG*	intronic		BRST, SKIN		TCF4
rs11569361	12	6553806	42,507	40,576	G>A	0.25	1.00	0.97	1.02	8.0E-01	*CD27, VAMP1, NCAPD2, MRPL51, TAPBPL, PKP2P1, RPL31P10, SRP14P1, CD27-AS1*	intronic	BLD, GI, THYM	BLD, THYM	BLD	
rs9551427	13	28614740	42,497	40,575	G>A	0.29	1.00	0.98	1.03	8.0E-01	*FLT3, LOC100420919*	intronic		HRT, SPLN		SIX5
rs2412459	15	40295959	42,489	40,558	A>G	0.08	1.00	0.97	1.04	8.0E-01	*SRP14, EIF2AK4, SRP14-AS1*	intronic	LIV	9 tissues	ESDR,IPSC	EWSR1-FLI1
rs4794976	17	25974545	42,072	40,110	A>C	0.30	1.00	0.98	1.02	8.0E-01	*LGALS9, KSR1, NOS2P1, ITM2BP1, LOC100420408*	intronic		GI, BLD		
rs4129267	1	154426264	42,504	40,574	G>A	0.39	1.00	0.98	1.02	8.0E-01	*IL6R, TDRD10, SHE, PSMD8P1*	intronic	BLD, SKIN	17 tissues	24 tissues	EBF,Elf3,Pou1f1
rs4722178	7	22798650	42,503	40,571	A>G	0.49	1.00	0.98	1.02	8.0E-01	*IL6, RPS26P32, LOC541472*			SKIN	SKIN	Myc,Myf
rs8030687	15	40265588	42,509	40,576	G>A	0.29	1.00	0.98	1.03	8.0E-01	*EIF2AK4, H3F3AP1, LOC100505534*	intronic		LIV		5 altered motifs
rs11104932	12	88925437	42,507	40,575	A>G	0.10	1.00	0.96	1.03	8.0E-01	*KITLG*	intronic		BRST, SKIN		E2F,PLZF,Pbx-1
rs17639305	13	43146187	42,500	40,571	A>G	0.17	1.00	0.97	1.02	8.1E-01	*TNFSF11*	intronic				7 altered motifs
rs17414706	8	79715374	42,507	40,576	G>A	0.10	1.00	0.96	1.03	8.1E-01	*IL7, PRKRIRP7*	intronic	GI, BLD	4 tissues	BLD	Nkx3,TATA
rs1554606	7	22768707	42,503	40,572	C>A	0.47	1.00	0.98	1.02	8.1E-01	*IL6, RPS26P32, LOC541472*	intronic	LNG	ESDR, MUS, SKIN	BLD	SETDB1,SIX5
rs11260542	1	1110019	42,473	40,542	A>G	0.09	1.00	0.96	1.03	8.1E-01	*TNFRSF4, TNFRSF18, SDF4, LOC254099, TTLL10, MIR200A, MIR200B, MIR429, TTLL10-AS1*	3'-UTR		7 tissues	ESC,IPSC,BLD	XBP-1
rs3181224	5	158740850	42,506	40,574	A>G	0.10	1.00	0.96	1.03	8.1E-01	*IL12B, UBLCP1, LOC285626, RNU4ATAC2P*			SKIN		
rs4750495	10	6501445	42,501	40,572	T>A	0.27	1.00	0.98	1.02	8.1E-01	*PRKCQ*	intronic		BLD, THYM		En-1,p53
rs11064337	12	6845793	42,446	40,446	G>C	0.11	1.00	0.97	1.04	8.1E-01	*LAG3, PTMS, MLF2, COPS7A, ZNF384, PIANP*			FAT, BRST, SKIN	BLD	5 altered motifs
rs1201157	1	12249031	42,492	40,573	G>A	0.43	1.00	0.98	1.02	8.1E-01	*TNFRSF8, TNFRSF1B, VPS13D, LOC390998, MIR4632*	intronic		15 tissues	IPSC,GI	
rs730691	5	158756227	42,501	40,570	G>A	0.37	1.00	0.98	1.02	8.1E-01	*IL12B, UBLCP1, LOC285626, RNU4ATAC2P*	intronic				6 altered motifs
rs5742909	2	204732347	42,510	40,575	G>A	0.09	1.00	0.97	1.04	8.1E-01	*CTLA4*		BLD	BLD, SKIN, THYM	BLD,THYM	
rs13075797	3	3122695	42,311	40,401	A>C	0.13	1.00	0.97	1.03	8.1E-01	*IL5RA, TRNT1, CNTN4*	intronic		4 tissues		4 altered motifs
rs3807907	7	18629590	42,505	40,573	A>G	0.48	1.00	0.98	1.02	8.1E-01	*HDAC9, LOC100419901*	intronic		BLD		Dbx1,Pou3f2
rs900	1	218614905	42,481	40,553	A>T	0.27	1.00	0.98	1.02	8.1E-01	*TGFB2*	3'-UTR				Maf
rs7100152	10	6607058	42,503	40,569	A>G	0.11	1.00	0.96	1.03	8.1E-01	*PRKCQ, PRKCQ-AS1*	intronic	BLD	ESDR, BLD, THYM		Cdx,Foxj2,Pou2f2
rs62235145	22	22178104	42,312	40,306	A>G	0.10	1.00	0.96	1.03	8.1E-01	*MAPK1*	intronic		15 tissues	IPSC,BLD	4 altered motifs
rs1494554	5	35873872	42,498	40,570	A>C	0.27	1.00	0.98	1.02	8.1E-01	*IL7R, CAPSL*	intronic		BLD, THYM		
rs1805113	1	92177938	42,501	40,572	A>G	0.45	1.00	0.98	1.02	8.1E-01	*TGFBR3*	synonymous	HRT, MUS, BLD	10 tissues	BLD	GATA,PU.1,STAT
rs4844553	1	206934363	42,398	40,486	G>A	0.06	0.99	0.95	1.04	8.1E-01	*IL10, MAPKAPK2, IL19*			BLD, KID, SPLN		YY1
rs1234317	1	173187775	42,510	40,573	G>A	0.26	1.00	0.97	1.02	8.1E-01	*TNFSF4, LOC100506023*					Cdc5,Hltf,Pax-4
rs17045337	2	54104615	42,507	40,570	A>G	0.11	1.00	0.97	1.04	8.1E-01	*GPR75, PSME4, GPR75-ASB3, MIR3682*	intronic		7 tissues	ADRL	4 altered motifs
rs10782997	1	92177394	42,507	40,576	T>A	0.45	1.00	0.98	1.02	8.1E-01	*TGFBR3*	intronic				AP-1,Hltf
rs11104930	12	88925072	42,452	40,545	A>C	0.10	1.00	0.96	1.03	8.1E-01	*KITLG*	intronic	SKIN	6 tissues	4 tissues	7 altered motifs
rs11256442	10	6079344	42,508	40,567	G>A	0.29	1.00	0.98	1.02	8.1E-01	*IL2RA, RPL32P23*	intronic		4 tissues	4 tissues	GATA,Pax-5
rs2359952	1	198656407	42,469	40,484	A>G	0.31	1.00	0.98	1.02	8.1E-01	*PTPRC, PEBP1P3*	intronic	BLD	BLD, THYM		E2A,Lmo2-complex,TCF12
rs17887218	1	92179918	42,510	40,572	G>A	0.07	1.00	0.97	1.05	8.1E-01	*TGFBR3*	intronic		8 tissues		4 altered motifs
rs11104924	12	88921216	42,507	40,577	A>G	0.10	1.00	0.96	1.03	8.1E-01	*KITLG*	intronic			BLD	MAZR,MZF1::1-4,PU.1
rs12517292	5	132355147	42,508	40,576	G>A	0.18	1.00	0.98	1.03	8.1E-01	*HSPA4, ZCCHC10, LOC402230*	intronic		LNG		p300
rs656715	10	6520549	42,493	40,554	A>G	0.19	1.00	0.97	1.02	8.1E-01	*PRKCQ*	intronic				LRH1
rs2243263	5	132013299	42,499	40,566	C>G	0.11	1.00	0.97	1.04	8.1E-01	*IL4, IL13, RAD50, KIF3A*	intronic		15 tissues		Mef2,Pou1f1,TCF4
rs2069762	4	123377980	42,488	40,557	A>C	0.29	1.00	0.98	1.02	8.1E-01	*IL2, ADAD1*		BLD, GI	BLD, PANC	BLD,GI	5 altered motifs
rs6894567	5	158756968	42,505	40,577	A>G	0.19	1.00	0.98	1.03	8.1E-01	*IL12B, UBLCP1, LOC285626, RNU4ATAC2P*	intronic		MUS		Foxp1,THAP1
rs2035207	2	182289461	42,510	40,575	G>A	0.26	1.00	0.97	1.02	8.1E-01	*ITGA4*			BLD		11 altered motifs
rs305080	16	85941774	42,507	40,570	G>A	0.33	1.00	0.98	1.02	8.1E-01	*IRF8*	intronic	BLD	BLD, FAT, THYM	BLD	GATA,Hdx

SNP	Chr	Position[a]	N Cases	N Controls	Allele	MAF	OR[b]	95%CI		*p* -value	Gene annotation	dbSNP functional annotation	Promoter histone marks	Enhancer histone marks	DNAse	Motifs changed
rs942201	10	6086292	42,503	40,569	C>A	0.23	1.00	0.98	1.03	8.1E-01	*IL2RA, RBM17, RPL32P23*	intronic		BLD, THYM	THYM	6 altered motifs
rs4842476	12	88922974	42,497	40,565	A>T	0.10	1.00	0.96	1.03	8.1E-01	*KITLG*	intronic				Arid5a,Foxa,HP1-site-factor
rs12146312	10	6503894	42,505	40,575	C>A	0.27	1.00	0.98	1.02	8.2E-01	*PRKCQ*	intronic		KID		STAT
rs9869655	3	3121857	42,490	40,546	G>A	0.11	1.00	0.97	1.03	8.2E-01	*IL5RA, TRNT1, CNTN4*	intronic		ESDR, ESC		6 altered motifs
rs8078731	17	40480381	42,505	40,574	A>T	0.18	1.00	0.98	1.03	8.2E-01	*STAT3, STAT5A*	intronic		ESC, IPSC, BRST		Maf
rs12105941	2	182448410	42,504	40,565	G>A	0.28	1.00	0.98	1.03	8.2E-01	*ITGA4, CERKL*	intronic				TCF4
rs11104931	12	88925213	42,506	40,570	A>G	0.10	1.00	0.96	1.03	8.2E-01	*KITLG*	intronic		BRST, SKIN, GI		5 altered motifs
rs2520341	7	18841917	42,087	39,925	A>C	0.31	1.00	0.98	1.02	8.2E-01	*HDAC9*	intronic				5 altered motifs
rs3782174	12	88938877	42,510	40,576	T>A	0.10	1.00	0.96	1.03	8.2E-01	*KITLG*	intronic	SKIN	10 tissues	9 tissues	
rs822336	9	5448690	42,501	40,573	C>G	0.46	1.00	0.98	1.02	8.2E-01	*CD274, PLGRKT, LOC100419687*		BLD	6 tissues	BLD,BLD	Gfi1,NRSF
rs4266612	8	128707406	42,387	40,511	A>C	0.35	1.00	0.98	1.02	8.2E-01	*MYC*		BLD	ESDR, BLD	18 tissues	
rs12401885	1	186682875	41,851	39,820	A>G	0.13	1.00	0.97	1.03	8.2E-01	*PTGS2*			CRVX		12 altered motifs
rs11633416	15	40250506	42,503	40,570	A>G	0.35	1.00	0.98	1.02	8.2E-01	*GPR176, EIF2AK4, H3F3AP1, LOC100505534*	intronic				
rs11165307	1	92179256	42,500	40,573	A>G	0.45	1.00	0.98	1.02	8.2E-01	*TGFBR3*	intronic	4 tissues	15 tissues	22 tissues	5 altered motifs
rs11104911	12	88904764	42,510	40,577	G>A	0.10	1.00	0.96	1.03	8.2E-01	*KITLG*	intronic		FAT, SKIN, LNG		Evi-1
rs7073906	10	6012544	42,503	40,569	A>C	0.35	1.00	0.98	1.02	8.2E-01	*IL2RA, IL15RA, FBXO18*	intronic		5 tissues	BLD	
rs17885088	3	3111047	42,510	40,575	G>A	0.10	1.00	0.96	1.03	8.2E-01	*IL5RA, CNTN4*	3'-UTR				6 altered motifs
rs2279581	15	40300092	42,508	40,575	G>A	0.08	1.00	0.97	1.04	8.2E-01	*SRP14, EIF2AK4, SRP14-AS1*	intronic		4 tissues		ERalpha-a,PPAR
rs919766	5	158747564	42,126	40,258	A>C	0.10	1.00	0.96	1.03	8.2E-01	*IL12B, UBLCP1, LOC285626, RNU4ATAC2P*	intronic				Brachyury,EBF,HNF1
rs3218108	6	41902655	42,500	40,566	G>A	0.25	1.00	0.97	1.02	8.2E-01	*BYSL, CCND3, MED20, USP49*			13 tissues	MUS	PLZF,SP2
rs2390042	7	19032534	42,501	40,572	A>G	0.40	1.00	0.98	1.02	8.2E-01	*HDAC9, NPM1P13*	intronic				HNF1,HNF4
rs6997891	8	79718005	42,507	40,577	C>A	0.10	1.00	0.96	1.03	8.2E-01	*IL7, PRKRIRP7*		19 tissues	5 tissues	5 tissues	5 altered motifs
rs10899245	11	76354115	42,480	40,536	A>G	0.35	1.00	0.98	1.02	8.2E-01	*LRRC32, GUCY2EP*					BATF,PRDM1,TCF12
rs928180	9	101897732	42,507	40,574	A>G	0.09	1.00	0.97	1.04	8.2E-01	*TGFBR1*	intronic	BLD, SKIN	11 tissues	BLD,THYM	5 altered motifs
rs17476066	4	15852104	42,479	40,544	A>G	0.30	1.00	0.98	1.02	8.2E-01	*CD38, LOC100130067*			BLD		4 altered motifs
rs1887327	10	6608733	42,506	40,576	A>G	0.15	1.00	0.98	1.03	8.2E-01	*PRKCQ, PRKCQ-AS1*	intronic		5 tissues	ESDR	BRCA1
rs10911898	1	186612593	42,494	40,571	C>A	0.26	1.00	0.98	1.03	8.2E-01	*PTGS2*				CRVX	
rs879574	22	17588305	42,495	40,570	T>A	0.12	1.00	0.97	1.04	8.2E-01	*IL17RA, CECR6, CECR5, CECR7, RPL31P62, LOC100996342*	intronic	BLD	4 tissues	BLD	4 altered motifs
rs7077401	10	6025878	42,504	40,574	A>G	0.42	1.00	0.98	1.02	8.2E-01	*IL2RA, IL15RA, FBXO18*			4 tissues	7 tissues	
rs10276613	7	18729827	42,500	40,571	G>A	0.17	1.00	0.97	1.02	8.2E-01	*HDAC9*	intronic				Cdx
rs6658473	1	218600419	42,461	40,540	A>G	0.27	1.00	0.98	1.02	8.2E-01	*TGFB2*	intronic		ESDR, FAT, BRST		TBX5
rs11256456	10	6080712	42,463	40,540	A>G	0.21	1.00	0.98	1.03	8.2E-01	*IL2RA, RPL32P23*	intronic		BLD	BLD,BLD,THYM	12 altered motifs
rs3764384	17	76207728	42,502	40,570	G>A	0.32	1.00	0.98	1.02	8.2E-01	*BIRC5, TK1, SYNGR2, AFMID, TMEM235, THA1P, C17orf99, LOC100996291*		MUS	4 tissues	OVRY,MUS	CTCFL
rs2236379	10	6527143	42,503	40,569	G>A	0.24	1.00	0.97	1.02	8.2E-01	*PRKCQ*	missense		BLD		5 altered motifs
rs72783703	10	6566026	42,498	40,567	G>A	0.08	1.00	0.97	1.04	8.2E-01	*PRKCQ*	intronic		BLD		COMP1
rs12366295	12	88920019	42,508	40,577	G>A	0.10	1.00	0.96	1.03	8.2E-01	*KITLG*	intronic				SETDB1
rs7546005	1	92186303	42,356	40,475	A>T	0.38	1.00	0.98	1.02	8.2E-01	*TGFBR3*	intronic		LIV		6 altered motifs
rs9282641	3	121796768	42,506	40,573	G>A	0.09	1.00	0.96	1.03	8.2E-01	*CD86*	5'-UTR	BLD, GI	8 tissues	4 tissues	
rs10518676	15	40250677	42,502	40,568	A>T	0.35	1.00	0.98	1.02	8.2E-01	*GPR176, EIF2AK4, H3F3AP1, LOC100505534*	intronic				Ik-1,NF-kappaB,RBP-Jkappa
rs11777564	8	79719034	42,504	40,575	A>G	0.10	1.00	0.96	1.03	8.2E-01	*IL7, PRKRIRP7*		9 tissues	7 tissues	4 tissues	
rs3136615	10	6005510	42,510	40,575	G>C	0.19	1.00	0.97	1.02	8.2E-01	*IL2RA, IL15RA, FBXO18*	intronic		PANC		6 altered motifs
rs1960859	4	123323926	42,414	40,546	A>G	0.27	1.00	0.98	1.02	8.2E-01	*IL2, KIAA1109, ADAD1*	intronic				RXRA
rs284174	1	92221608	42,497	40,563	A>C	0.39	1.00	0.98	1.02	8.2E-01	*TGFBR3*	intronic		9 tissues	6 tissues	AP-1
rs7762931	6	43773768	42,505	40,572	C>A	0.29	1.00	0.98	1.02	8.3E-01	*VEGFA*		LIV, GI	7 tissues	GI,GI,LIV	
rs8178561	21	34668797	42,509	40,576	G>A	0.05	1.00	0.95	1.04	8.3E-01	*IFNAR1, IFNAR2, IL10RB, USF1P1, IL10RB-AS1*	3'-UTR		BLD	4 tissues	BDP1,HNF4
rs1474348	7	22767908	42,482	40,552	C>G	0.44	1.00	0.98	1.02	8.3E-01	*IL6, RPS26P32, LOC541472*	intronic	14 tissues	10 tissues	7 tissues	6 altered motifs
rs4252328	14	76437128	42,508	40,577	G>A	0.22	1.00	0.97	1.02	8.3E-01	*TGFB3, TTLL5, IFT43, LOC100506576*	intronic		HRT, MUS		
rs7090530	10	6110875	42,496	40,572	A>C	0.39	1.00	0.98	1.02	8.3E-01	*IL2RA, RBM17, RPL32P23*		BLD, THYM	8 tissues	4 tissues	Foxc1
rs4435098	13	28587839	42,476	40,547	C>A	0.27	1.00	0.98	1.02	8.3E-01	*CDX2, FLT3, PRHOXNB*	intronic		BLD		Hoxa4
rs2143417	1	186653787	42,505	40,577	C>A	0.15	1.00	0.97	1.03	8.3E-01	*PTGS2*					Hoxd10,Sox
rs10858757	12	88932804	42,504	40,571	G>C	0.10	1.00	0.96	1.03	8.3E-01	*KITLG*	intronic			MUS	
rs12573280	10	6487774	42,492	40,572	C>A	0.32	1.00	0.98	1.02	8.3E-01	*PRKCQ*	intronic		5 tissues	20 tissues	Hoxb13,Hoxd10
rs2296620	1	92174563	41,414	39,588	G>A	0.21	1.00	0.97	1.02	8.3E-01	*TGFBR3*	intronic		ADRL, HRT, BLD		Evi-1,Pou2f2
rs1726610	7	18630208	42,503	40,565	C>A	0.36	1.00	0.98	1.02	8.3E-01	*HDAC9, LOC100419901*	intronic	SKIN	BRST, BLD, SKIN	ESDR,ESC,LNG	E4F1
rs7614952	3	45900621	42,485	40,568	G>A	0.29	1.00	0.98	1.02	8.3E-01	*CCR9, SDHDP4, LZTFL1*			4 tissues	5 tissues	FAC1,Irx,p300
rs6984323	8	128706908	42,484	40,557	A>G	0.50	1.00	0.98	1.02	8.3E-01	*MYC*			ESDR, BLD, GI	BLD	5 altered motifs
rs7602690	2	182299180	42,497	40,571	G>C	0.14	1.00	0.97	1.03	8.3E-01	*ITGA4*			BLD		Homez,Irx,p300
rs3744678	17	3635768	42,498	40,571	A>C	0.22	1.00	0.98	1.03	8.3E-01	*ITGAE, P2RX5, GSG2, P2RX5-TAX1BP3*	intronic	4 tissues	12 tissues	16 tissues	Rhox11
rs2188320	7	18666901	42,492	40,546	A>C	0.34	1.00	0.98	1.02	8.3E-01	*HDAC9, LOC100419901*	intronic		FAT		

SNP	Chr	Position[a]	N Cases	N Controls	Allele	MAF	OR[b]	95%CI		*p* -value	Gene annotation	dbSNP functional annotation	Promoter histone marks	Enhancer histone marks	DNAse	Motifs changed
rs2796823	1	218586214	42,282	40,320	A>T	0.27	1.00	0.98	1.02	8.3E-01	*TGFB2*	intronic		MUS		5 altered motifs
rs1044873	16	85955671	42,507	40,575	G>A	0.39	1.00	0.98	1.02	8.3E-01	*IRF8*	3'-UTR			MUS	4 altered motifs
rs6602360	10	6029726	42,496	40,566	A>G	0.35	1.00	0.98	1.02	8.3E-01	*IL2RA, IL15RA*		BRN, BLD	4 tissues	LNG,MUS,BLD	PRDM1
rs7521726	1	92178753	42,497	40,576	G>A	0.45	1.00	0.98	1.02	8.3E-01	*TGFBR3*	intronic	8 tissues	15 tissues	13 tissues	VDR
rs11258976	10	6502442	42,501	40,563	G>A	0.38	1.00	0.98	1.02	8.3E-01	*PRKCQ*	intronic		THYM		9 altered motifs
rs6952102	7	18753386	42,310	40,390	G>A	0.10	1.00	0.97	1.04	8.3E-01	*HDAC9*	intronic				FXR,Hoxb4,Nkx2
rs11258960	10	6500858	42,508	40,573	A>G	0.27	1.00	0.98	1.02	8.3E-01	*PRKCQ*	intronic		BLD, THYM	5 tissues	Ets
rs2526632	7	19032809	42,276	40,211	A>C	0.39	1.00	0.98	1.02	8.3E-01	*HDAC9, NPM1P13*	intronic				
rs11104895	12	88865006	42,495	40,564	G>A	0.10	1.00	0.96	1.03	8.3E-01	*KITLG*			6 tissues		GATA
rs76094862	8	128713665	42,499	40,572	G>A	0.31	1.00	0.98	1.02	8.3E-01	*MYC*				IPSC	5 altered motifs
rs16923195	9	5512522	42,503	40,572	A>C	0.26	1.00	0.98	1.03	8.4E-01	*CD274, PDCD1LG2*	intronic	11 tissues	9 tissues	4 tissues	PU.1
rs284877	1	92173796	42,506	40,573	G>A	0.27	1.00	0.98	1.03	8.4E-01	*TGFBR3*	intronic		5 tissues		4 altered motifs
rs1323653	10	6039058	42,504	40,577	A>G	0.10	1.00	0.97	1.04	8.4E-01	*IL2RA, IL15RA*			ESC, ESDR, IPSC		5 altered motifs
rs8083511	18	60028655	42,489	40,542	A>C	0.19	1.00	0.97	1.02	8.4E-01	*TNFRSF11A*	intronic	ESDR	BLD, GI, HRT	IPSC,OVRY,GI	GR
rs6588250	1	67669516	42,507	40,577	C>A	0.25	1.00	0.98	1.03	8.4E-01	*IL23R*	intronic			LNG,SKIN,LNG	4 altered motifs
rs3134883	10	6100725	42,498	40,564	G>A	0.31	1.00	0.98	1.02	8.4E-01	*IL2RA, RBM17, RPL32P23*	intronic	BLD	FAT, BLD	BLD,BLD	Foxj1,Mrg1::Hoxa9
rs284177	1	92224698	42,506	40,573	A>G	0.44	1.00	0.98	1.02	8.4E-01	*TGFBR3*	intronic		12 tissues	SKIN	6 altered motifs
rs7072496	10	6608313	42,472	40,554	A>G	0.15	1.00	0.98	1.03	8.4E-01	*PRKCQ, PRKCQ-AS1*	intronic	BLD	6 tissues		
rs6792117	3	30704007	42,499	40,558	G>A	0.48	1.00	0.98	1.02	8.4E-01	*TGFBR2*	intronic		11 tissues		Bcl6b
rs17884458	3	3147196	42,508	40,576	A>G	0.20	1.00	0.98	1.03	8.4E-01	*IL5RA, TRNT1, CRBN, CNTN4*	5'-UTR		BLD		4 altered motifs
rs17377783	10	90791823	42,498	40,575	G>A	0.35	1.00	0.98	1.02	8.4E-01	*ACTA2, FAS, FAS-AS1, MIR4679-1, MIR4679-2*		5 tissues	18 tissues	8 tissues	
rs2876981	22	22205611	42,483	40,554	A>C	0.37	1.00	0.98	1.02	8.4E-01	*MAPK1*	intronic		4 tissues	BLD	AP-1,Evi-1,PLZF
rs2506135	10	33465037	42,492	40,562	A>G	0.28	1.00	0.98	1.02	8.4E-01	*NRP1*			5 tissues	SKIN	Sox
rs284180	1	92227848	42,482	40,569	A>C	0.44	1.00	0.98	1.02	8.4E-01	*TGFBR3*	intronic		13 tissues		
rs2498786	14	105262368	42,458	40,547	C>G	0.38	1.00	0.98	1.02	8.4E-01	*AKT1, SIVA1, RPS2P4, ADSSL1, LINC00638, ZBTB42, RPS26P49*		23 tissues	BRN, LNG	17 tissues	4 altered motifs
rs3129881	6	32409484	42,491	40,531	G>A	0.30	1.00	0.98	1.02	8.4E-01	*HLA-DRA, HLA-DRB9, BTNL2, HCG23*	intronic	5 tissues	BLD, SPLN		Nkx2,Nkx3,PU.1
rs2244677	10	30678440	42,509	40,573	A>G	0.48	1.00	0.98	1.02	8.4E-01	*CCND3P, MAP3K8, MTPAP, MKI67IPP1, LOC729668, DNM1P17, GOLGA2P6*			ADRL, LNG		11 altered motifs
rs587372	10	6524500	42,466	40,542	C>G	0.36	1.00	0.98	1.02	8.4E-01	*PRKCQ*	intronic		BLD	BLD	4 altered motifs
rs790633	1	67678993	42,508	40,576	G>A	0.30	1.00	0.98	1.02	8.4E-01	*IL23R*	intronic		BLD	13 tissues	Hoxa3,Sox
rs7548373	1	206933490	42,463	40,508	C>A	0.06	1.00	0.96	1.04	8.4E-01	*IL10, MAPKAPK2, IL19*			BLD		Pax-8
rs8192284	1	154426970	42,493	40,565	A>C	0.39	1.00	0.98	1.02	8.4E-01	*IL6R, TDRD10, SHE, PSMD8P1*					
rs17047682	1	218523025	42,509	40,576	A>G	0.06	1.00	0.96	1.05	8.4E-01	*TGFB2, RRP15, RPS26P17, LOC728463*	intronic	12 tissues	7 tissues	6 tissues	Hoxb13
rs62215622	20	44763201	42,509	40,577	C>A	0.07	1.00	0.96	1.04	8.4E-01	*CD40, NCOA5, CDH22, RPL13P2*			BRN	IPSC,BLD	Nkx3,TCF12
rs2283563	16	27346354	42,490	40,569	G>A	0.32	1.00	0.98	1.02	8.4E-01	*IL4R, FLJ21466*	intronic	GI	11 tissues	6 tissues	4 altered motifs
rs11258759	10	6474308	42,509	40,575	G>A	0.25	1.00	0.98	1.03	8.4E-01	*PRKCQ*	intronic		GI	LNG	6 altered motifs
rs730690	5	158756166	42,509	40,577	G>A	0.19	1.00	0.98	1.03	8.4E-01	*IL12B, UBLCP1, LOC285626, RNU4ATAC2P*	intronic				5 altered motifs
rs7841229	8	128726314	42,151	40,319	T>A	0.49	1.00	0.98	1.02	8.4E-01	*MYC*					11 altered motifs
rs17512269	1	92177062	42,502	40,576	G>A	0.24	1.00	0.98	1.03	8.4E-01	*TGFBR3*	intronic	HRT	5 tissues		BATF,Hdx,Hsf
rs10506956	12	88932035	42,510	40,577	C>A	0.10	1.00	0.96	1.03	8.4E-01	*KITLG*	intronic				SIX5,STAT
rs3804803	3	3124182	42,510	40,577	A>G	0.13	1.00	0.97	1.03	8.4E-01	*IL5RA, TRNT1, CNTN4*	intronic	BLD	ESC, BLD	BLD	
rs9658761	10	90769886	42,510	40,576	C>A	0.11	1.00	0.97	1.04	8.4E-01	*ACTA2, FAS, FAS-AS1*	intronic				7 altered motifs
rs4500848	18	60014463	42,509	40,576	G>A	0.06	1.00	0.96	1.05	8.4E-01	*TNFRSF11A, KIAA1468*	intronic	IPSC, BLD, VAS	9 tissues	8 tissues	Mtf1
rs4846480	1	218598469	42,499	40,569	T>A	0.27	1.00	0.98	1.02	8.4E-01	*TGFB2*	intronic		8 tissues		Hoxa5,PRDM1
rs1800871	1	206946634	42,507	40,572	G>A	0.24	1.00	0.98	1.03	8.4E-01	*IL10, MAPKAPK2, IL19*		BLD	4 tissues	BLD	5 altered motifs
rs4845623	1	154415777	42,501	40,571	A>G	0.41	1.00	0.98	1.02	8.5E-01	*IL6R, SHE, PSMD8P1*	intronic		11 tissues		
rs655828	7	18392543	42,505	40,573	A>G	0.17	1.00	0.97	1.02	8.5E-01	*HDAC9*	intronic	ESC	14 tissues	BLD,MUS,CRVX	ERalpha-a,NRSF
rs4711748	6	43694598	42,504	40,571	G>A	0.23	1.00	0.98	1.03	8.5E-01	*VEGFA, MRPS18A, LOC100132242*			SKIN		CTCF,RBP-Jkappa
rs944712	10	6474841	42,476	40,543	A>G	0.25	1.00	0.98	1.03	8.5E-01	*PRKCQ*	intronic				AP-1,RXRA,Smad
rs4842632	12	88931532	42,497	40,571	A>G	0.10	1.00	0.96	1.03	8.5E-01	*KITLG*	intronic				9 altered motifs
rs7828417	8	79716435	42,508	40,577	A>G	0.10	1.00	0.96	1.03	8.5E-01	*IL7, PRKRIRP7*	intronic	23 tissues	4 tissues	9 tissues	Foxj2,Hand1
rs8177636	10	6018594	42,494	40,573	A>G	0.41	1.00	0.98	1.02	8.5E-01	*IL2RA, IL15RA, FBXO18*	intronic	22 tissues	6 tissues	23 tissues	10 altered motifs
rs863716	9	5445702	42,139	40,218	G>A	0.47	1.00	0.98	1.02	8.5E-01	*CD274, PLGRKT, LOC100419687*			BLD		DMRT5
rs1569922	1	67664963	42,503	40,576	G>A	0.41	1.00	0.98	1.02	8.5E-01	*IL23R*	intronic				4 altered motifs
rs2717350	7	19021073	42,495	40,550	G>A	0.22	1.00	0.97	1.02	8.5E-01	*HDAC9, NPM1P13*	intronic				Pbx3
rs6949149	7	22749157	42,507	40,577	C>A	0.07	1.00	0.96	1.04	8.5E-01	*IL6, LOC401312, LOC541472*		ESDR, BLD	4 tissues	8 tissues	4 altered motifs
rs2072324	17	26116896	42,509	40,575	C>A	0.20	1.00	0.98	1.03	8.5E-01	*NOS2, LOC645754*	intronic		ESDR		7 altered motifs
rs4142559	13	28661648	42,504	40,573	T>A	0.39	1.00	0.98	1.02	8.5E-01	*FLT3, CHCHD2P8, PAN3-AS1, LOC100420919*	intronic				AP-1,Foxp1
rs3816899	15	40314526	42,509	40,575	A>G	0.08	1.00	0.97	1.04	8.5E-01	*SRP14, EIF2AK4, SRP14-AS1*	intronic		BLD	BLD,BLD	4 altered motifs
rs4073630	13	28589267	42,487	40,563	A>G	0.48	1.00	0.98	1.02	8.5E-01	*CDX2, FLT3, PRHOXNB*	intronic				Hdx,Pbx-1
rs6605258	2	242796660	42,118	40,203	G>A	0.40	1.00	0.98	1.02	8.5E-01	*PDCD1, NEU4, CXXC11, LOC285095*	intronic		10 tissues		8 altered motifs

SNP	Chr	Position[a]	N Cases	N Controls	Allele	MAF	OR[b]	95%CI		*p* -value	Gene annotation	dbSNP functional annotation	Promoter histone marks	Enhancer histone marks	DNAse	Motifs changed
rs6596104	5	132480458	42,506	40,570	G>A	0.10	1.00	0.96	1.03	8.5E-01	*HSPA4, RPL6P15, LOC100506102*					7 altered motifs
rs17140251	7	18943707	42,510	40,577	C>A	0.06	1.00	0.96	1.05	8.5E-01	*HDAC9, NPM1P13*	intronic	FAT	4 tissues	MUS,MUS,SKIN	Foxp1
rs4803455	19	41851509	42,504	40,576	A>C	0.50	1.00	0.98	1.02	8.5E-01	*TGFB1, HNRNPUL1, EXOSC5, B9D2, CCDC97, TMEM91*	intronic		BLD		Hoxb7,STAT
rs6074047	20	44787478	42,470	40,544	G>A	0.13	1.00	0.97	1.03	8.5E-01	*CD40, CDH22*			ESDR, ADRL, GI		Klf7,Pax-4
rs11104939	12	88932364	42,402	40,500	A>G	0.10	1.00	0.96	1.03	8.5E-01	*KITLG*	intronic				BATF,GATA
rs10272295	7	18934478	42,412	40,492	A>C	0.13	1.00	0.97	1.03	8.5E-01	*HDAC9*	intronic				Pitx2
rs2396081	6	43770512	42,506	40,575	A>G	0.39	1.00	0.98	1.02	8.5E-01	*VEGFA*			11 tissues	GI,GI,BLD	6 altered motifs
rs7514452	1	154438084	42,506	40,573	A>G	0.20	1.00	0.98	1.03	8.5E-01	*IL6R, TDRD10, SHE*	3'-UTR	BLD	12 tissues		ERalpha-a,RXRA,SP1
rs7900744	10	6065611	41,606	39,795	A>G	0.22	1.00	0.98	1.03	8.5E-01	*IL2RA, IL15RA, RPL32P23*	intronic		4 tissues	5 tissues	Mrg1::Hoxa9
rs116985019	10	6003387	42,450	40,467	A>G	0.41	1.00	0.98	1.02	8.5E-01	*IL2RA, IL15RA, FBXO18*	intronic		LNG, BRST		7 altered motifs
rs4721715	7	18694434	42,509	40,574	G>A	0.16	1.00	0.97	1.03	8.5E-01	*HDAC9*	intronic		ESC, BLD, GI	MUS	
rs6699304	1	92193716	42,503	40,569	G>A	0.22	1.00	0.97	1.02	8.5E-01	*TGFBR3*	intronic	BLD	10 tissues	MUS	NRSF
rs4658261	1	92184744	42,507	40,573	G>A	0.22	1.00	0.97	1.02	8.5E-01	*TGFBR3*	intronic		BRST, LIV	PANC,LIV	HNF4
rs17810546	3	159665050	42,507	40,575	A>G	0.12	1.00	0.97	1.03	8.5E-01	*IL12A, SCHIP1, IQCJ-SCHIP1*			8 tissues	10 tissues	4 altered motifs
rs2887501	8	79718580	42,502	40,577	A>C	0.10	1.00	0.96	1.03	8.5E-01	*IL7, PRKRIRP7*		17 tissues	5 tissues	7 tissues	
rs944715	10	6479156	42,509	40,576	G>A	0.25	1.00	0.98	1.03	8.6E-01	*PRKCQ*	intronic				RP58,p300
rs17461808	4	142590988	42,510	40,577	G>C	0.08	1.00	0.97	1.04	8.6E-01	*IL15*	intronic				IRC900814,Mef2
rs2412463	15	40302973	42,504	40,575	G>C	0.29	1.00	0.98	1.02	8.6E-01	*SRP14, EIF2AK4, SRP14-AS1*	intronic	BRN, MUS	7 tissues	14 tissues	7 altered motifs
rs10882680	10	97640542	42,503	40,568	A>C	0.14	1.00	0.97	1.03	8.6E-01	*ENTPD1, ENTPD1-AS1, C10orf131*	intronic				Sox,TEF
rs937213	15	40322124	42,494	40,561	A>G	0.42	1.00	0.98	1.02	8.6E-01	*SRP14, EIF2AK4, SRP14-AS1*	intronic	HRT	4 tissues	BLD,HRT	7 altered motifs
rs6073983	20	44629891	42,501	40,571	T>A	0.22	1.00	0.98	1.03	8.6E-01	*MMP9, SLC12A5, ZNF335, FTLP1, LOC100128028*		FAT, BLD	11 tissues	BLD,BRN	Arid5b
rs8030640	15	40265503	42,477	40,556	C>A	0.29	1.00	0.98	1.02	8.6E-01	*EIF2AK4, H3F3AP1, LOC100505534*	intronic		LIV		
rs3118471	10	6102757	42,484	40,558	A>G	0.31	1.00	0.98	1.02	8.6E-01	*IL2RA, RBM17, RPL32P23*	intronic	BLD, GI	BLD	BLD,BLD	17 altered motifs
rs11165347	1	92191269	42,484	40,553	G>C	0.28	1.00	0.98	1.02	8.6E-01	*TGFBR3*	intronic	BONE	15 tissues	5 tissues	
rs2541323	7	18750861	42,507	40,577	A>C	0.10	1.00	0.96	1.03	8.6E-01	*HDAC9*	intronic				HDAC2
rs1474347	7	22768124	42,507	40,576	A>C	0.44	1.00	0.98	1.02	8.6E-01	*IL6, RPS26P32, LOC541472*	intronic	11 tissues	9 tissues	18 tissues	6 altered motifs
rs1557696	7	18620049	42,491	40,559	A>G	0.47	1.00	0.98	1.02	8.6E-01	*HDAC9, LOC100419901*	intronic		BRN		Maf,NF-kappaB
rs1005230	6	43736496	42,503	40,571	G>A	0.50	1.00	0.98	1.02	8.6E-01	*VEGFA*				6 tissues	STAT,TCF12
rs17558745	1	218548521	42,502	40,568	G>A	0.31	1.00	0.98	1.02	8.6E-01	*TGFB2, RRP15, RPS26P17, LOC728463*	intronic	LNG	14 tissues	15 tissues	TAL1
rs8143081	22	22262369	42,505	40,574	A>G	0.29	1.00	0.98	1.02	8.6E-01	*MAPK1, TOP3B, PPM1F, LOC100286925*				BLD	PPAR
rs7185022	16	85952929	42,433	40,530	C>A	0.50	1.00	0.98	1.02	8.6E-01	*IRF8*	intronic		ESDR, BLD	BLD	
rs16843518	1	198595042	42,507	40,577	A>G	0.05	1.00	0.95	1.04	8.6E-01	*PTPRC*			BLD, THYM	BLD	Arid3a,Hoxa10,SIX5
rs12442713	15	40280766	42,492	40,558	A>C	0.37	1.00	0.98	1.02	8.6E-01	*SRP14, EIF2AK4, H3F3AP1*	intronic			PLCNT	Ik-2,Rhox11
rs11104928	12	88921417	42,464	40,491	A>G	0.10	1.00	0.97	1.04	8.6E-01	*KITLG*	intronic				SRF
rs17268130	2	54172496	42,500	40,564	C>G	0.15	1.00	0.97	1.03	8.6E-01	*PSME4*	intronic	FAT, BLD	8 tissues		AP-1,RXRA
rs8064496	17	40474864	42,388	40,517	A>G	0.17	1.00	0.98	1.03	8.6E-01	*STAT3, STAT5A, STAT5B*	intronic		14 tissues	47 tissues	Gfi1b,Spz1
rs3730013	17	26125918	42,506	40,577	G>A	0.33	1.00	0.98	1.02	8.6E-01	*NOS2, LOC645754*	intronic	GI	GI		EWSR1-FLI1,HDAC2
rs11165300	1	92177663	42,499	40,573	A>C	0.24	1.00	0.98	1.03	8.6E-01	*TGFBR3*	intronic	HRT, BLD	4 tissues	BLD	Foxa,GR
rs1488691	1	92332865	42,500	40,573	A>C	0.48	1.00	0.98	1.02	8.6E-01	*TGFBR3*	intronic	SKIN, GI	12 tissues	ADRL	TAL1,VDR
rs2279455	1	92185185	42,489	40,560	G>A	0.40	1.00	0.98	1.02	8.6E-01	*TGFBR3*	intronic		5 tissues		AP-2,RREB-1
rs1049894	2	54093956	42,509	40,577	G>A	0.06	1.00	0.96	1.05	8.6E-01	*GPR75, PSME4, ERLEC1, GPR75-ASB3, MIR3682*	synonymous				
rs7010461	8	39781444	42,499	40,565	G>A	0.32	1.00	0.98	1.02	8.6E-01	*IDO1, IDO2, LOC100420480*	intronic		9 tissues	SKIN,BLD	PU.1
rs10827248	10	33650374	42,510	40,574	G>A	0.08	1.00	0.97	1.04	8.6E-01	*NRP1*					p300
rs13241157	7	18860014	42,467	40,539	G>A	0.29	1.00	0.98	1.02	8.6E-01	*HDAC9*	intronic				AP-1
rs4698119	4	15738795	42,505	40,577	G>A	0.31	1.00	0.98	1.02	8.6E-01	*BST1, CD38, FAM200B, RPL10AP7, LOC100288771*			BLD, THYM		RORalpha1
rs7549755	1	92157808	42,508	40,568	A>G	0.21	1.00	0.97	1.02	8.6E-01	*TGFBR3, HSP90B3P*	intronic		FAT		4 altered motifs
rs12428172	13	28645757	42,504	40,577	G>A	0.47	1.00	0.98	1.02	8.6E-01	*FLT3, CHCHD2P8, LOC100420919*	intronic			THYM	
rs9533147	13	43123925	42,498	40,575	G>A	0.47	1.00	0.98	1.02	8.6E-01	*TNFSF11*		BLD	8 tissues	5 tissues	SIX5
rs12565552	1	92224631	42,498	40,572	G>A	0.15	1.00	0.97	1.03	8.6E-01	*TGFBR3*	intronic		12 tissues	PLCNT,OVRY	PRDM1
rs906639	12	88891453	42,485	40,538	G>A	0.10	1.00	0.96	1.03	8.7E-01	*KITLG*	intronic		BRST, SKIN, BONE		ATF3,Pbx-1,TCF4
rs2008134	12	6588445	42,455	40,524	A>G	0.32	1.00	0.98	1.02	8.7E-01	*CD27, VAMP1, NCAPD2, MRPL51, TAPBPL, PKP2P1, SRP14P1, CD27-AS1, SCARNA10*			ESDR, SPLN, SKIN	4 tissues	BCL,NRSF,Sin3Ak-20
rs3807918	7	18615101	42,073	40,497	G>C	0.14	1.00	0.97	1.03	8.7E-01	*HDAC9, LOC100419901*	intronic			LNG	Ik-2
rs3738441	1	92224067	42,499	40,574	G>A	0.26	1.00	0.98	1.02	8.7E-01	*TGFBR3*	intronic		15 tissues	BLD,SKIN,MUS	HNF4,HP1-site-factor
rs4721721	7	18740164	42,506	40,575	A>G	0.12	1.00	0.97	1.03	8.7E-01	*HDAC9*	intronic				9 altered motifs
rs10486327	7	18981897	42,405	40,462	G>A	0.09	1.00	0.96	1.03	8.7E-01	*HDAC9, NPM1P13*	intronic				Zfp187
rs17095086		114251054	42,346	40,330	G>C	0.15	1.00	0.97	1.03	8.7E-01	*IL13RA2, LOC100419790*	intronic		FAT		4 altered motifs
rs1882924	17	3659516	42,485	40,567	C>G	0.20	1.00	0.98	1.03	8.7E-01	*ITGAE, GSG2*	intronic		4 tissues		6 altered motifs

SNP	Chr	Position[a]	N Cases	N Controls	Allele	MAF	OR[b]	95%CI		*p* -value	Gene annotation	dbSNP functional annotation	Promoter histone marks	Enhancer histone marks	DNAse	Motifs changed
rs12324733	15	40262691	42,508	40,573	C>G	0.35	1.00	0.98	1.02	8.7E-01	*GPR176, EIF2AK4, H3F3AP1, LOC100505534*	intronic		9 tissues		6 altered motifs
rs3770111	2	182382316	42,483	40,561	G>A	0.11	1.00	0.97	1.03	8.7E-01	*ITGA4, CERKL*	intronic		5 tissues	SKIN	
rs6665312	1	92180903	42,508	40,576	G>A	0.09	1.00	0.97	1.04	8.7E-01	*TGFBR3*	intronic		5 tissues	BLD	STAT
rs2796814	1	218561581	42,502	40,571	C>G	0.23	1.00	0.97	1.02	8.7E-01	*TGFB2, RPS26P17, LOC728463*	intronic		BRN, LNG		Cdx,Smad3
rs4616886	5	132423764	42,501	40,573	A>G	0.18	1.00	0.98	1.03	8.7E-01	*HSPA4, LOC100506102*	intronic				AhR,Foxa,Pax-4
rs16923189	9	5510644	42,501	40,571	A>G	0.29	1.00	0.98	1.02	8.7E-01	*CD274, PDCD1LG2*	5'-UTR	17 tissues	11 tissues	23 tissues	LBP-1,Nanog
rs10827239	10	33630848	42,507	40,575	A>G	0.08	1.00	0.96	1.03	8.7E-01	*NRP1*				8 tissues	12 altered motifs
rs305089	16	85981030	42,504	40,570	G>A	0.33	1.00	0.98	1.02	8.7E-01	*IRF8*		BLD, GI	5 tissues	6 tissues	4 altered motifs
rs502919	10	6517167	42,509	40,575	A>G	0.19	1.00	0.97	1.02	8.7E-01	*PRKCQ*	intronic	BLD	6 tissues	KID	
rs9579141	13	28583657	42,503	40,574	G>A	0.28	1.00	0.98	1.02	8.7E-01	*CDX2, FLT3, PRHOXNB*	intronic		ESC, BLD, LIV		TCF4
rs17150245	7	18698060	42,507	40,576	T>A	0.07	1.00	0.97	1.04	8.7E-01	*HDAC9*	intronic				Nanog,Pou5f1,Sox
rs2206593	1	186642429	42,501	40,568	G>A	0.06	1.00	0.96	1.04	8.7E-01	*PTGS2*	3'-UTR				Foxa,Homez,Pou2f2
rs12427992	13	28598825	42,502	40,573	A>G	0.18	1.00	0.97	1.02	8.7E-01	*FLT3, PRHOXNB, LOC100420919*	intronic				8 altered motifs
rs2528406	7	18986970	42,429	40,477	G>A	0.16	1.00	0.97	1.03	8.7E-01	*HDAC9, NPM1P13*	intronic				Arid3a,Pou2f2
rs4771218	13	28655311	42,462	40,515	A>G	0.39	1.00	0.98	1.02	8.7E-01	*FLT3, CHCHD2P8, LOC100420919*	intronic				Foxa,NF-kappaB,STAT
rs2241715	19	41856886	41,852	40,026	C>A	0.30	1.00	0.98	1.02	8.7E-01	*BCKDHA, TGFB1, HNRNPUL1, EXOSC5, B9D2, CCDC97, TMEM91*	intronic	20 tissues	10 tissues	19 tissues	4 altered motifs
rs2229238	1	154437896	42,469	40,553	G>A	0.20	1.00	0.98	1.03	8.7E-01	*IL6R, TDRD10, SHE*	3'-UTR	BLD	12 tissues	BLD	FAC1,Nanog,Sox
rs9839494	3	3078603	42,505	40,575	A>G	0.38	1.00	0.98	1.02	8.7E-01	*IL5RA, CNTN4*	intronic		ESDR, BRN		5 altered motifs
rs10146195	14	76477246	42,506	40,575	A>C	0.15	1.00	0.98	1.03	8.7E-01	*TGFB3, IFT43, LOC100506576*	intronic				5 altered motifs
rs923378	1	92202883	42,355	40,363	G>C	0.11	1.00	0.97	1.03	8.7E-01	*TGFBR3*	intronic	8 tissues	22 tissues	26 tissues	5 altered motifs
rs1413711	6	43740678	42,408	40,538	G>A	0.50	1.00	0.98	1.02	8.7E-01	*VEGFA*	intronic	21 tissues	4 tissues	12 tissues	13 altered motifs
rs4842477	12	88926072	42,459	40,519	G>A	0.10	1.00	0.96	1.03	8.7E-01	*KITLG*	intronic				HDAC2,Pou5f1
rs8092336	18	60036083	42,508	40,576	G>A	0.06	1.00	0.96	1.05	8.7E-01	*TNFRSF11A, RPL17P44*	synonymous		BLD	BLD,BLD	4 altered motifs
rs4934599	10	33633962	42,499	40,564	G>A	0.08	1.00	0.96	1.03	8.7E-01	*NRP1*			5 tissues		12 altered motifs
rs805367	2	54171093	42,414	40,477	C>A	0.50	1.00	0.98	1.02	8.7E-01	*PSME4*	intronic				9 altered motifs
rs7080157	10	6512559	42,503	40,571	G>A	0.19	1.00	0.97	1.02	8.7E-01	*PRKCQ*	intronic		BLD	BLD	Mrg,Nkx2
rs2491223	13	28607989	42,508	40,576	C>A	0.22	1.00	0.98	1.03	8.7E-01	*FLT3, PRHOXNB, LOC100420919*	intronic		BLD		SRF
rs12358161	10	6481227	42,504	40,575	G>A	0.14	1.00	0.97	1.03	8.7E-01	*PRKCQ*	intronic				SEF-1
rs6745710	2	191878654	42,495	40,562	C>G	0.30	1.00	0.98	1.02	8.7E-01	*GLS, STAT1, STAT4, LOC100420571*	intronic	24 tissues		45 tissues	
rs7524679	1	92179448	42,509	40,576	G>A	0.09	1.00	0.97	1.04	8.8E-01	*TGFBR3*	intronic		13 tissues	5 tissues	
rs11466605	1	92177352	42,510	40,576	A>G	0.09	1.00	0.97	1.04	8.8E-01	*TGFBR3*	intronic	HRT, BLD	4 tissues		Foxd3,Foxj1
rs7406657	17	26083690	42,498	40,564	G>C	0.24	1.00	0.98	1.03	8.8E-01	*NOS2, LOC645754*				BLD	Pbx3,Rad21,SP2
rs12722588	10	6060433	42,509	40,576	G>A	0.19	1.00	0.97	1.02	8.8E-01	*IL2RA, IL15RA*	intronic		9 tissues	6 tissues	Foxj2
rs3773650	3	30718429	42,507	40,575	C>A	0.19	1.00	0.98	1.03	8.8E-01	*TGFBR2, GADL1*	intronic		7 tissues		Pax-6,Pou3f2
rs304839	3	30731282	42,504	40,575	A>T	0.18	1.00	0.98	1.03	8.8E-01	*TGFBR2, GADL1*	intronic		19 tissues	SKIN,CRVX,VAS	Cphx,Nkx6-1,Pou2f2
rs17131544	1	92205087	42,509	40,577	A>G	0.11	1.00	0.97	1.03	8.8E-01	*TGFBR3*	intronic		BRST	BRST,SKIN	LBP-1
rs12722864	1	92191534	42,506	40,575	G>A	0.09	1.00	0.96	1.03	8.8E-01	*TGFBR3*	intronic	BONE	15 tissues	14 tissues	Barhl1,Hmx,Isl2
rs2546893	5	158755960	42,508	40,575	G>A	0.50	1.00	0.98	1.02	8.8E-01	*IL12B, UBLCP1, LOC285626, RNU4ATAC2P*	intronic				ZBTB7A,Zfp691
rs11465803	1	67702141	42,506	40,576	G>A	0.12	1.00	0.97	1.03	8.8E-01	*IL23R, LOC100130497*	intronic		GI	GI	
rs2453	10	6469673	42,506	40,575	G>A	0.25	1.00	0.98	1.03	8.8E-01	*PRKCQ*	3'-UTR				NF-I,Pou1f1,TATA
rs3135334	6	32402686	42,495	40,568	A>G	0.19	1.00	0.98	1.03	8.8E-01	*HLA-DRA, HLA-DRB9, BTNL2, HCG23*			BLD		Ik-3,RREB-1
rs12070470	1	67697271	42,494	40,573	G>A	0.12	1.00	0.97	1.03	8.8E-01	*IL23R, LOC100130497*	intronic		SKIN		DEC,Myf
rs2293158	17	40447558	42,505	40,570	A>G	0.29	1.00	0.98	1.02	8.8E-01	*STAT3, STAT5A, STAT5B*	intronic		BLD		5 altered motifs
rs17140333	7	18977047	42,508	40,577	G>A	0.07	1.00	0.97	1.04	8.8E-01	*HDAC9, NPM1P13*	intronic				Sox
rs10195683	2	191879560	42,505	40,574	G>A	0.23	1.00	0.97	1.02	8.8E-01	*GLS, STAT1, STAT4, LOC100420571*				21 tissues	7 altered motifs
rs791589	10	6089571	42,506	40,577	A>G	0.13	1.00	0.97	1.03	8.8E-01	*IL2RA, RBM17, RPL32P23*	intronic		BLD		11 altered motifs
rs2491231	13	28610183	42,499	40,574	G>A	0.22	1.00	0.98	1.03	8.8E-01	*FLT3, PRHOXNB, LOC100420919*	intronic		BLD		Ik-2
rs434364	19	6640827	42,360	40,410	G>A	0.38	1.00	0.98	1.02	8.8E-01	*C3, CD70, TNFSF14, RPL7P50*			BLD		Egr-1
rs928055	10	30731146	42,507	40,577	G>A	0.25	1.00	0.98	1.02	8.8E-01	*CCND3P, MAP3K8*	intronic		12 tissues	11 tissues	4 altered motifs
rs12145973	1	206994725	42,502	40,575	G>A	0.15	1.00	0.97	1.03	8.8E-01	*IL10, IL19, IL20*	intronic		FAT, MUS		Brachyury
rs1805112	1	92185657	42,501	40,572	G>A	0.44	1.00	0.98	1.02	8.8E-01	*TGFBR3*	synonymous		5 tissues		12 altered motifs
rs944713	10	6475053	42,490	40,562	T>A	0.25	1.00	0.98	1.03	8.8E-01	*PRKCQ*	intronic				AIRE
rs7765636	6	86136661	42,495	40,561	G>A	0.34	1.00	0.98	1.02	8.8E-01	*NT5E, LOC643870, DUTP5*					IRC900814
rs3115962	2	204556706	42,507	40,564	G>A	0.08	1.00	0.96	1.04	8.8E-01	*CD28, LOC100287498*			7 tissues	MUS,MUS,VAS	15 altered motifs
rs5748863	22	17571355	42,505	40,572	A>G	0.32	1.00	0.98	1.02	8.8E-01	*IL17RA, CECR6, CECR5, CECR7, RPL31P62, LOC100996342*	intronic		BLD, THYM		E4BP4,Irf,Myc
rs3815975	10	6533877	42,493	40,570	A>G	0.41	1.00	0.98	1.02	8.8E-01	*PRKCQ*	intronic	SKIN	4 tissues	4 tissues	Foxo
rs2695029	7	18626623	42,423	40,512	G>A	0.45	1.00	0.98	1.02	8.8E-01	*HDAC9, LOC100419901*	intronic				
rs9566990	13	43164710	42,481	40,551	G>C	0.37	1.00	0.98	1.02	8.8E-01	*TNFSF11*	intronic		ESC	BLD	AP-1,Pax-4,SP1
rs2269754	7	18699475	42,501	40,576	A>G	0.22	1.00	0.97	1.02	8.8E-01	*HDAC9*	intronic		5 tissues	5 tissues	GATA,RFX5
rs2285443	7	18704889	42,508	40,573	G>A	0.15	1.00	0.97	1.03	8.8E-01	*HDAC9*	intronic			IPSC,ADRL	Nkx2,RFX5

SNP	Chr	Position[a]	N Cases	N Controls	Allele	MAF	OR[b]	95%CI		*p*-value	Gene annotation	dbSNP functional annotation	Promoter histone marks	Enhancer histone marks	DNAse	Motifs changed
rs673964	10	6488790	42,502	40,573	G>A	0.29	1.00	0.98	1.02	8.8E-01	*PRKCQ*	intronic		BLD, THYM	ESDR	Pou3f3
rs9642880	8	128718068	42,503	40,575	C>A	0.47	1.00	0.98	1.02	8.8E-01	*MYC*					COMP1,Pbx-1,Roaz
rs11567697	5	35858324	42,431	40,508	A>G	0.11	1.00	0.97	1.03	8.8E-01	*IL7R, SPEF2, CAPSL*	intronic	7 tissues	11 tissues	SKIN,SKIN,CRVX	
rs6965090	7	18743563	42,493	40,529	G>A	0.11	1.00	0.97	1.03	8.8E-01	*HDAC9*	intronic		ESDR, HRT, MUS	4 tissues	
rs10249700	7	18978611	42,509	40,574	C>A	0.09	1.00	0.96	1.03	8.8E-01	*HDAC9, NPM1P13*	intronic				Mef2
rs8178556	21	34666555	42,500	40,564	A>C	0.06	1.00	0.96	1.05	8.8E-01	*IFNAR1, IFNAR2, IL10RB, USF1P1, IL10RB-AS1*	intronic	THYM	BLD, SKIN	7 tissues	Cphx,Prrx2,Znf143
rs392751	19	6645178	42,498	40,569	A>G	0.32	1.00	0.98	1.02	8.9E-01	*C3, TNFSF14*					PEBP,Sox
rs2395175	6	32405026	42,496	40,565	G>A	0.17	1.00	0.98	1.03	8.9E-01	*HLA-DRA, HLA-DRB9, BTNL2, HCG23*		BLD	4 tissues	5 tissues	Ets,NRSF
rs10999385	10	72308254	42,499	40,569	G>A	0.23	1.00	0.97	1.02	8.9E-01	*PRF1, PALD1*	intronic			IPSC,IPSC	BCL,p300
rs3129852	6	32397784	42,503	40,572	A>G	0.19	1.00	0.98	1.03	8.9E-01	*HLA-DRA, HLA-DRB9, BTNL2, HCG23*					Sox
rs943450	10	6606435	42,508	40,573	A>G	0.16	1.00	0.97	1.03	8.9E-01	*PRKCQ, PRKCQ-AS1*	intronic	BLD	9 tissues	6 tissues	5 altered motifs
rs2425756	20	44790262	42,504	40,575	C>A	0.36	1.00	0.98	1.02	8.9E-01	*CD40, CDH22*					GATA,Hltf,TAL1
rs6602705	10	6487428	42,502	40,575	G>A	0.41	1.00	0.98	1.02	8.9E-01	*PRKCQ*	intronic		4 tissues	BLD	Foxl1,Zfp105
rs12493328	3	119218781	42,465	40,550	T>A	0.20	1.00	0.97	1.02	8.9E-01	*CD80, CSRP2P, TIMMDC1, TMEM39A, POGLUT1*	intronic	19 tissues	10 tissues		4 altered motifs
rs9978523	21	34589270	42,485	40,515	G>A	0.32	1.00	0.98	1.02	8.9E-01	*IFNAR2, IL10RB, C21orf54, IL10RB-AS1*		IPSC, BLD	10 tissues	14 tissues	Nanog,Pou5f1,Sox
rs9309251	2	54133638	42,306	40,238	G>A	0.07	1.00	0.96	1.04	8.9E-01	*GPR75, PSME4, GPR75-ASB3*	intronic				5 altered motifs
rs305061	16	85975659	42,493	40,564	A>G	0.33	1.00	0.98	1.02	8.9E-01	*IRF8*			BLD, LIV		Esr2,RXRA
rs2389962	7	18486949	42,483	40,536	G>A	0.20	1.00	0.98	1.03	8.9E-01	*HDAC9*	intronic				PEBP
rs2387585	10	6614057	42,479	40,535	A>G	0.38	1.00	0.98	1.02	8.9E-01	*PRKCQ, PRKCQ-AS1*	intronic		BLD, THYM		
rs72781746	10	6508405	42,455	40,521	G>A	0.19	1.00	0.97	1.02	8.9E-01	*PRKCQ*	intronic		4 tissues		4 altered motifs
rs11104896	12	88865241	42,443	40,524	A>G	0.10	1.00	0.97	1.03	8.9E-01	*KITLG*			12 tissues	4 tissues	Spz1
rs9610500	22	22221167	42,504	40,567	A>G	0.36	1.00	0.98	1.02	8.9E-01	*MAPK1*	intronic	24 tissues		37 tissues	ELF1
rs17815013	13	28614264	42,502	40,573	G>A	0.15	1.00	0.97	1.03	8.9E-01	*FLT3, LOC100420919*	intronic			THYM	AIRE,Pou5f1,Sox
rs1940937	11	102763604	42,508	40,576	G>A	0.18	1.00	0.98	1.03	8.9E-01	*MMP3, MMP12, LOC100288111*					Dobox4,En-1
rs2491240	13	28613167	42,501	40,573	A>G	0.18	1.00	0.97	1.02	8.9E-01	*FLT3, LOC100420919*	intronic				Rad21
rs1555887	1	92160957	42,507	40,573	A>G	0.26	1.00	0.98	1.02	8.9E-01	*TGFBR3*	intronic		FAT		Pax-2
rs6908133	6	43768330	42,486	40,562	A>C	0.39	1.00	0.98	1.02	8.9E-01	*VEGFA*			12 tissues		SP1
rs2494748	14	105258892	42,452	40,522	A>G	0.39	1.00	0.98	1.02	8.9E-01	*AKT1, SIVA1, RPS2P4, ADSSL1, LINC00638, ZBTB42, RPS26P49*	intronic	4 tissues	19 tissues	4 tissues	5 altered motifs
rs1418556	1	218616014	42,480	40,547	G>A	0.29	1.00	0.98	1.02	8.9E-01	*TGFB2*	3'-UTR				8 altered motifs
rs3787268	20	44641731	42,501	40,574	G>A	0.22	1.00	0.98	1.03	8.9E-01	*MMP9, SLC12A5, NCOA5, ZNF335, FTLP1, LOC100128028*	intronic		5 tissues	BLD,BLD	6 altered motifs
rs7327579	13	28615835	42,507	40,575	A>G	0.47	1.00	0.98	1.02	8.9E-01	*FLT3, LOC100420919*	intronic	BLD	5 tissues	4 tissues	7 altered motifs
rs11811962	1	92176726	42,507	40,577	A>G	0.09	1.00	0.97	1.04	8.9E-01	*TGFBR3*	intronic	HRT	9 tissues		HES1
rs10508781	10	33640577	42,508	40,576	A>C	0.08	1.00	0.96	1.03	8.9E-01	*NRP1*					4 altered motifs
rs3176774	12	9917106	42,503	40,573	C>A	0.31	1.00	0.98	1.02	8.9E-01	*CD69, CLECL1*		BLD, THYM	4 tissues	7 tissues	Evi-1,FAC1,PEBP
rs34129849	17	40418956	42,229	40,281	A>C	0.29	1.00	0.98	1.02	8.9E-01	*STAT3, STAT5A, STAT5B*	intronic	BLD, LNG	15 tissues	24 tissues	GATA
rs16990497	21	34680575	42,336	40,471	G>A	0.06	1.00	0.96	1.05	9.0E-01	*IFNAR1, IFNAR2, IL10RB, USF1P1, IL10RB-AS1*		BLD	BLD, MUS, THYM		Hic1,Pax-5
rs2546892	5	158755475	42,499	40,565	G>A	0.17	1.00	0.97	1.02	9.0E-01	*IL12B, UBLCP1, LOC285626, RNU4ATAC2P*	intronic				GATA,SETDB1
rs7523492	1	157637964	42,496	40,554	A>G	0.41	1.00	0.98	1.02	9.0E-01	*FCRL3, SONP1*					
rs801767	7	18567280	41,706	39,677	A>G	0.05	1.00	0.96	1.05	9.0E-01	*HDAC9*	intronic		BLD		
rs805310	2	54196238	42,509	40,576	G>A	0.35	1.00	0.98	1.02	9.0E-01	*PSME4*	intronic	22 tissues	4 tissues	BRST,VAS	
rs10075878	5	132425049	42,494	40,563	G>A	0.49	1.00	0.98	1.02	9.0E-01	*HSPA4, LOC100506102*	intronic				SRF
rs3024496	1	206941864	42,506	40,570	A>G	0.49	1.00	0.98	1.02	9.0E-01	*IL10, MAPKAPK2, IL19*	3'-UTR	BLD	BLD, SKIN, THYM		
rs2810887	1	92158571	42,507	40,575	G>A	0.26	1.00	0.98	1.02	9.0E-01	*TGFBR3, HSP90B3P*	intronic		6 tissues		14 altered motifs
rs13401719	2	54185647	42,485	40,537	G>A	0.07	1.00	0.96	1.04	9.0E-01	*PSME4*	intronic		ESC, IPSC, BLD	SKIN	Foxp1,KAP1,Pax-4
rs8177685	10	6008613	42,508	40,575	A>G	0.12	1.00	0.97	1.03	9.0E-01	*IL2RA, IL15RA, FBXO18*	intronic		FAT		Pax-4,VDR
rs10827245	10	33639488	42,508	40,574	A>C	0.08	1.00	0.96	1.03	9.0E-01	*NRP1*					
rs4658260	1	92184673	42,497	40,566	G>A	0.22	1.00	0.97	1.02	9.0E-01	*TGFBR3*	intronic		BRST, LIV	GI,PANC,LIV	4 altered motifs
rs17659894	3	3134203	42,507	40,576	C>A	0.06	1.00	0.96	1.04	9.0E-01	*IL5RA, TRNT1, CNTN4*	intronic				
rs12735851	1	92182699	42,502	40,569	C>A	0.09	1.00	0.97	1.04	9.0E-01	*TGFBR3*	intronic				CEBPB
rs7560575	2	54142030	42,510	40,577	A>G	0.06	1.00	0.96	1.04	9.0E-01	*PSME4*	intronic		BLD		4 altered motifs
rs718872	2	54173706	42,502	40,573	T>A	0.50	1.00	0.98	1.02	9.0E-01	*PSME4*	intronic		4 tissues		7 altered motifs
rs726805	7	18962688	42,493	40,564	A>T	0.38	1.00	0.98	1.02	9.0E-01	*HDAC9, NPM1P13*	intronic				AP-1,Foxa,STAT
rs801758	7	18564562	41,943	39,937	G>A	0.29	1.00	0.98	1.02	9.0E-01	*HDAC9*	intronic				4 altered motifs
rs6974299	7	18859647	41,668	39,583	A>G	0.30	1.00	0.98	1.02	9.0E-01	*HDAC9*	intronic				
rs16971010	17	76269099	42,507	40,571	A>G	0.08	1.00	0.96	1.04	9.0E-01	*BIRC5, TMEM235, THA1P, LOC100996291*		9 tissues	15 tissues	30 tissues	KAP1,PU.1
rs2474731	10	33472215	42,506	40,574	G>A	0.10	1.00	0.97	1.04	9.0E-01	*NRP1*	intronic				ATF3,Roaz,THAP1
rs72633721	1	11310456	42,509	40,574	G>A	0.09	1.00	0.96	1.03	9.0E-01	*MTOR, UBIAD1, RPL39P6, UBE2V2P3*	intronic				HNF1,Lhx3

SNP	Chr	Position[a]	N Cases	N Controls	Allele	MAF	OR[b]	95%CI		*p* -value	Gene annotation	dbSNP functional annotation	Promoter histone marks	Enhancer histone marks	DNAse	Motifs changed
rs13417051	2	54101772	42,367	40,316	G>A	0.06	1.00	0.96	1.04	9.0E-01	*GPR75, PSME4, GPR75-ASB3, MIR3682*	intronic				5 altered motifs
rs2239927	7	18689883	42,455	40,497	C>A	0.15	1.00	0.97	1.03	9.0E-01	*HDAC9*	intronic		HRT		AFP1,Foxo,Pax-4
rs7982889	13	28644885	42,503	40,570	A>G	0.18	1.00	0.98	1.03	9.0E-01	*FLT3, CHCHD2P8, LOC100420919*	intronic				Homez,Pou2f2,Pou3f2
rs7911362	10	6130226	42,499	40,568	G>A	0.40	1.00	0.98	1.02	9.0E-01	*IL2RA, RBM17, RPL32P23*		FAT	BLD, BRN	5 tissues	ERalpha-a,GR
rs12559890		135685169	42,504	40,574	C>A	0.22	1.00	0.98	1.03	9.0E-01	*CD40LG, VGLL1, LINC00892*			BLD		CEBPB,Foxj2
rs1476420	22	38041649	42,474	40,542	A>G	0.09	1.00	0.96	1.03	9.0E-01	*LGALS1, SH3BP1, GGA1, PDXP, NOL12*	intronic		BLD	OVRY	4 altered motifs
rs4970420	1	1106473	42,455	40,511	G>A	0.19	1.00	0.97	1.02	9.0E-01	*TNFRSF4, TNFRSF18, SDF4, LOC254099, TTLL10, MIR200A, MIR200B, MIR429, TTLL10-AS1*			7 tissues	BLD	Otx2
rs10506954	12	88924752	41,824	39,822	A>T	0.10	1.00	0.97	1.03	9.0E-01	*KITLG*	intronic			BRST,SKIN,BRST	4 altered motifs
rs2150686	10	33649023	42,509	40,577	A>G	0.08	1.00	0.97	1.04	9.0E-01	*NRP1*					GR,Zbtb12
rs7820850	8	128768191	42,505	40,574	G>A	0.12	1.00	0.97	1.03	9.0E-01	*MYC, MIR1204*			8 tissues	14 tissues	21 altered motifs
rs12048049	1	218597297	42,508	40,576	G>C	0.29	1.00	0.98	1.02	9.0E-01	*TGFB2*	intronic		BRN, GI, LNG		4 altered motifs
rs586457	10	6497422	42,502	40,570	A>G	0.29	1.00	0.98	1.02	9.0E-01	*PRKCQ*	intronic				Pax-6
rs2491227	13	28609825	42,501	40,577	G>A	0.22	1.00	0.98	1.03	9.0E-01	*FLT3, PRHOXNB, LOC100420919*	intronic		BLD		5 altered motifs
rs12344	12	88886963	42,417	40,487	A>G	0.10	1.00	0.97	1.03	9.0E-01	*KITLG*	3'-UTR				Mef2,Sox,TATA
rs117355523	8	128747001	42,153	40,265	G>A	0.41	1.00	0.98	1.02	9.1E-01	*MYC*					
rs7095466	10	90784755	42,490	40,571	G>A	0.39	1.00	0.98	1.02	9.1E-01	*ACTA2, FAS, FAS-AS1, MIR4679-1, MIR4679-2*			MUS		Myc,Pax-6,Pou2f2
rs17131539	1	92190482	42,507	40,574	G>A	0.22	1.00	0.97	1.02	9.1E-01	*TGFBR3*	intronic		9 tissues		AP-2rep
rs7806011	7	18939200	42,356	40,418	G>A	0.36	1.00	0.98	1.02	9.1E-01	*HDAC9, NPM1P13*	intronic				11 altered motifs
rs1418553	1	218610254	42,505	40,577	G>A	0.29	1.00	0.98	1.02	9.1E-01	*TGFB2*	intronic		FAT, BRST, BRN		4 altered motifs
rs10911905	1	186652395	42,506	40,577	A>C	0.13	1.00	0.97	1.03	9.1E-01	*PTGS2*					Irf,Nrf1,STAT
rs9907247	17	40425876	42,449	40,508	G>A	0.29	1.00	0.98	1.02	9.1E-01	*STAT3, STAT5A, STAT5B*	intronic	4 tissues	11 tissues	4 tissues	4 altered motifs
rs3024491	1	206945046	42,507	40,573	C>A	0.49	1.00	0.98	1.02	9.1E-01	*IL10, MAPKAPK2, IL19*	intronic	BLD, GI	BLD, STRM, THYM	BLD	
rs2520346	7	18847958	42,501	40,564	G>A	0.15	1.00	0.97	1.03	9.1E-01	*HDAC9*	intronic		BRST		5 altered motifs
rs2026319	10	33621502	42,507	40,569	G>C	0.08	1.00	0.96	1.03	9.1E-01	*NRP1*	intronic	22 tissues	6 tissues	21 tissues	5 altered motifs
rs2285440	7	18698661	42,460	40,525	A>C	0.07	1.00	0.96	1.04	9.1E-01	*HDAC9*	intronic				14 altered motifs
rs3790567	1	67822377	42,507	40,572	G>A	0.23	1.00	0.98	1.03	9.1E-01	*IL12RB2*	intronic	SKIN	10 tissues	SKIN	Foxl1,THAP1
rs1800795	7	22766645	42,482	40,564	C>G	0.44	1.00	0.98	1.02	9.1E-01	*IL6, RPS26P32, LOC541472*	intronic	18 tissues	14 tissues	39 tissues	
rs991967	1	218615451	42,264	40,324	A>C	0.29	1.00	0.98	1.02	9.1E-01	*TGFB2*	3'-UTR				Foxa
rs2183715	10	33648763	42,508	40,573	A>C	0.08	1.00	0.97	1.04	9.1E-01	*NRP1*					
rs7217728	17	40447401	42,502	40,573	A>G	0.29	1.00	0.98	1.02	9.1E-01	*STAT3, STAT5A, STAT5B*	intronic		BLD	MUS,BRN	8 altered motifs
rs2498791	14	105248470	42,279	40,469	C>A	0.37	1.00	0.98	1.02	9.1E-01	*AKT1, SIVA1, ADSSL1, LINC00638, ZBTB42, RPS26P49*	intronic		5 tissues		4 altered motifs
rs8177692	10	6004012	42,503	40,575	A>G	0.28	1.00	0.98	1.02	9.1E-01	*IL2RA, IL15RA, FBXO18*	intronic		BRST		DMRT1,Ets,YY1
rs17300888	10	6509035	42,503	40,569	G>A	0.19	1.00	0.97	1.02	9.1E-01	*PRKCQ*	intronic		4 tissues	BLD,THYM	AP-2rep,Nkx6-1
rs7917197	10	6009026	42,508	40,566	G>A	0.13	1.00	0.97	1.03	9.1E-01	*IL2RA, IL15RA, FBXO18*	intronic				Homez
rs2387351	13	28649457	42,463	40,508	A>C	0.21	1.00	0.97	1.02	9.1E-01	*FLT3, CHCHD2P8, LOC100420919*	intronic				CDP,Maf,Pou2f2
rs11009357	10	33648920	42,505	40,577	C>A	0.08	1.00	0.97	1.04	9.1E-01	*NRP1*					Mef2
rs833061	6	43737486	42,491	40,558	A>G	0.50	1.00	0.98	1.02	9.1E-01	*VEGFA*		24 tissues	BLD, HRT	53 tissues	BCL,Pax-5,Znf143
rs17026703	3	3140785	42,496	40,571	A>T	0.31	1.00	0.98	1.02	9.1E-01	*IL5RA, TRNT1, CNTN4*	intronic				p300
rs1805015	16	27374180	42,500	40,560	A>G	0.17	1.00	0.97	1.03	9.1E-01	*IL4R, IL21R*	missense		6 tissues	IPSC,BLD,GI	FXR,NF-E2,p300
rs4737159	8	39796413	42,456	40,542	A>G	0.13	1.00	0.97	1.03	9.1E-01	*IDO1, IDO2, LOC100420480*	intronic		ESC, BLD, GI	LNG	7 altered motifs
rs3134603	6	32126002	42,510	40,574	G>A	0.13	1.00	0.97	1.03	9.1E-01	*AGER, ATF6B, NOTCH4, PBX2, RNF5, TNXB, PPT2, AGPAT1, GPSM3, FKBPL, PRRT1, EGFL8, LOC100507547, PPT2-EGFL8*	intronic				
rs9554232	13	28641053	42,504	40,573	A>G	0.19	1.00	0.98	1.03	9.1E-01	*FLT3, CHCHD2P8, LOC100420919*	intronic				TCF12,Zbtb3
rs10096900	8	128708284	42,497	40,572	C>A	0.32	1.00	0.98	1.02	9.1E-01	*MYC*					Cdx,Maf
rs3918262	20	44643770	42,482	40,554	A>G	0.22	1.00	0.98	1.03	9.1E-01	*MMP9, SLC12A5, NCOA5, ZNF335, FTLP1, LOC100128028*	intronic		6 tissues	BLD,LIV	9 altered motifs
rs3740778	11	76372418	42,504	40,576	G>A	0.13	1.00	0.97	1.03	9.1E-01	*LRRC32, GUCY2EP*	synonymous		MUS, PLCNT		
rs6756424	2	54130835	42,510	40,577	A>C	0.06	1.00	0.96	1.04	9.1E-01	*GPR75, PSME4, GPR75-ASB3*	intronic				23 altered motifs
rs17350383	7	18930213	42,500	40,570	G>A	0.18	1.00	0.98	1.03	9.1E-01	*HDAC9*	intronic		6 tissues	LNG,BRST	Pou2f2,SRF
rs7035064	9	101931740	42,409	40,462	A>G	0.09	1.00	0.97	1.04	9.1E-01	*TGFBR1, ALG2*		ESC, IPSC	4 tissues		5 altered motifs
rs1049631	16	27375542	42,509	40,572	A>G	0.46	1.00	0.98	1.02	9.1E-01	*IL4R, IL21R*	3'-UTR			4 tissues	5 altered motifs
rs2491237	13	28612351	42,505	40,575	G>A	0.18	1.00	0.97	1.02	9.2E-01	*FLT3, PRHOXNB, LOC100420919*	intronic		BLD		GATA
rs8177607	10	6024331	42,510	40,576	A>G	0.15	1.00	0.97	1.03	9.2E-01	*IL2RA, IL15RA, FBXO18*			BLD		Pbx-1
rs3917194	14	76431533	42,508	40,573	T>A	0.07	1.00	0.96	1.04	9.2E-01	*TGFB3, TTLL5, IFT43, LOC100506576*	intronic				AP-1,Nkx6-1
rs10264011	7	18970232	42,507	40,576	T>A	0.33	1.00	0.98	1.02	9.2E-01	*HDAC9, NPM1P13*	intronic				9 altered motifs
rs805424	2	54128235	42,480	40,557	A>G	0.50	1.00	0.98	1.02	9.2E-01	*GPR75, PSME4, GPR75-ASB3*	intronic		BRN		5 altered motifs
rs6671221	1	67638291	42,272	40,338	G>A	0.47	1.00	0.98	1.02	9.2E-01	*IL23R, C1orf141*	intronic		4 tissues		5 altered motifs
rs6567273	18	60033043	42,391	40,391	A>T	0.47	1.00	0.98	1.02	9.2E-01	*TNFRSF11A, RPL17P44*	intronic		BLD		19 altered motifs

SNP	Chr	Position[a]	N Cases	N Controls	Allele	MAF	OR[b]	95%CI		*p* -value	Gene annotation	dbSNP functional annotation	Promoter histone marks	Enhancer histone marks	DNAse	Motifs changed
rs2810893	1	92144970	42,494	40,568	G>A	0.43	1.00	0.98	1.02	9.2E-01	*TGFBR3, HSP90B3P*			5 tissues	MUS,SKIN	TCF12
rs11264793	1	157647526	42,420	40,438	A>T	0.24	1.00	0.98	1.02	9.2E-01	*FCRL3, SONP1, VDAC1P9*					6 altered motifs
rs2412452	15	40240544	42,507	40,575	A>C	0.47	1.00	0.98	1.02	9.2E-01	*GPR176, EIF2AK4, H3F3AP1, LOC100505534*	intronic		STRM, GI		Pax-4
rs17772814	8	128711742	42,485	40,512	G>A	0.09	1.00	0.96	1.03	9.2E-01	*MYC*			4 tissues		HNF1,Rhox11
rs10905773	10	6142476	42,506	40,573	A>G	0.34	1.00	0.98	1.02	9.2E-01	*IL2RA, PFKFB3, RBM17, RPL32P23*	intronic			ESC,GI,BLD	GR,Zbtb12
rs558662	15	40256324	42,506	40,574	A>G	0.47	1.00	0.98	1.02	9.2E-01	*GPR176, EIF2AK4, H3F3AP1, LOC100505534*	intronic			CRVX,SKIN	9 altered motifs
rs790635	1	67683486	42,497	40,549	G>A	0.27	1.00	0.98	1.02	9.2E-01	*IL23R*	intronic		4 tissues	BRN,MUS	5 altered motifs
rs7990075	13	43185780	42,500	40,577	A>G	0.40	1.00	0.98	1.02	9.2E-01	*TNFSF11*			BRN		Egr-1,MOVO-B
rs4683147	3	45948264	42,507	40,576	G>A	0.39	1.00	0.98	1.02	9.2E-01	*CXCR6, CCR9, SDHDP4, FYCO1*		GI	12 tissues	MUS	Ets,Pax-5,p300
rs6545377	2	54220049	42,505	40,567	C>A	0.35	1.00	0.98	1.02	9.2E-01	*PSME4, RPL21P30*	intronic		BLD		6 altered motifs
rs2588606	7	18731058	42,503	40,575	C>A	0.16	1.00	0.97	1.03	9.2E-01	*HDAC9*	intronic				Maf
rs10490573	2	204583163	42,496	40,537	G>A	0.18	1.00	0.98	1.03	9.2E-01	*CD28, KRT18P39, LOC100287498*	intronic			12 tissues	
rs16967637	17	40446422	42,502	40,572	C>A	0.29	1.00	0.98	1.02	9.2E-01	*STAT3, STAT5A, STAT5B*	intronic		9 tissues	4 tissues	4 altered motifs
rs4252340	14	76430993	42,492	40,567	A>G	0.07	1.00	0.96	1.04	9.2E-01	*TGFB3, TTLL5, IFT43, LOC100506576*	intronic				
rs790634	1	67680928	42,467	40,541	A>G	0.27	1.00	0.98	1.02	9.2E-01	*IL23R*	intronic		BRST, BLD, PANC	BLD	DMRT5,DMRT7,Tgif1
rs6702784	1	36904720	42,509	40,576	A>C	0.06	1.00	0.96	1.04	9.2E-01	*CSF3R, MRPS15, LSM10, OSCP1*	intronic		ESDR, SKIN, PLCNT	ESDR	5 altered motifs
rs6602364	10	6038853	42,502	40,566	G>C	0.44	1.00	0.98	1.02	9.2E-01	*IL2RA, IL15RA*			ESC, ESDR, IPSC	6 tissues	
rs3006490	1	153369009	42,509	40,577	C>A	0.07	1.00	0.96	1.04	9.2E-01	*S100A8, S100A9, S100A12, PGLYRP4, S100A7P1, S100A7A, S100A7P2, LOC645900, S100A7L2*			BLD, GI	BLD	7 altered motifs
rs3804797	3	3129632	42,509	40,575	A>G	0.15	1.00	0.97	1.03	9.2E-01	*IL5RA, TRNT1, CNTN4*	intronic		BLD		
rs1800797	7	22766221	42,506	40,577	G>A	0.43	1.00	0.98	1.02	9.2E-01	*IL6, RPS26P32, LOC541472*	5'-UTR	16 tissues	17 tissues	49 tissues	CAC-binding-protein
rs10889665	1	67636652	42,499	40,571	A>G	0.13	1.00	0.97	1.03	9.2E-01	*IL23R, C1orf141*	intronic			4 tissues	Mef2,Sox
rs11225442	11	102739319	41,476	39,430	G>A	0.14	1.00	0.97	1.03	9.2E-01	*MMP3, MMP12, WTAPP1, LOC100288111*	intronic		8 tissues	14 tissues	
rs11064213	12	6580376	42,499	40,571	A>G	0.20	1.00	0.98	1.03	9.2E-01	*CD27, VAMP1, NCAPD2, MRPL51, TAPBPL, PKP2P1, SRP14P1, CD27-AS1, SCARNA10*		24 tissues		52 tissues	Ets,Lmo2-complex,Spdef
rs2717349	7	19023736	42,451	40,525	C>G	0.26	1.00	0.98	1.02	9.2E-01	*HDAC9, NPM1P13*	intronic				6 altered motifs
rs2474728	10	33473743	42,507	40,573	G>A	0.10	1.00	0.97	1.04	9.2E-01	*NRP1*	intronic			KID	4 altered motifs
rs4655686	1	67638004	42,497	40,560	A>T	0.31	1.00	0.98	1.02	9.2E-01	*IL23R, C1orf141*	intronic		4 tissues	BLD,BLD,SKIN	12 altered motifs
rs1884444	1	67633812	42,508	40,571	A>C	0.47	1.00	0.98	1.02	9.2E-01	*IL23R, C1orf141*	missense	BLD	ADRL		7 altered motifs
rs2670289	3	119274586	42,506	40,574	A>C	0.25	1.00	0.98	1.02	9.2E-01	*ADPRH, CD80, CSRP2P, TIMMDC1, PLA1A*	intronic	BLD	6 tissues	BLD,BLD	8 altered motifs
rs477634	1	117268631	42,473	40,550	C>G	0.47	1.00	0.98	1.02	9.2E-01	*CD2, NEFHP1, GAPDHP64, FTH1P22*			ESC, BLD	5 tissues	
rs12751148	1	92190072	42,492	40,566	G>A	0.38	1.00	0.98	1.02	9.3E-01	*TGFBR3*	intronic		8 tissues	MUS,MUS	
rs6751340	2	54187617	42,509	40,577	A>G	0.07	1.00	0.96	1.04	9.3E-01	*PSME4*	intronic		5 tissues		4 altered motifs
rs7910501	10	6531513	42,504	40,575	G>A	0.09	1.00	0.97	1.04	9.3E-01	*PRKCQ*	intronic		BLD		EWSR1-FLI1,p300
rs501878	10	6526497	42,496	40,560	A>C	0.36	1.00	0.98	1.02	9.3E-01	*PRKCQ*	intronic		BLD		CEBPG,HEY1,SIX5
rs3025033	6	43751075	42,495	40,557	A>G	0.16	1.00	0.97	1.03	9.3E-01	*VEGFA*	intronic		7 tissues	6 tissues	BCL,INSM1,SP1
rs10950917	7	22811921	42,497	40,573	G>A	0.37	1.00	0.98	1.02	9.3E-01	*IL6, TOMM7, RPS26P32, LOC541472*					Foxa,Pou3f2,VDR
rs2241718	19	41829606	42,503	40,572	G>A	0.16	1.00	0.97	1.03	9.3E-01	*TGFB1, HNRNPUL1, B9D2, CCDC97, TMEM91*	3'-UTR	5 tissues	21 tissues	36 tissues	
rs9581970	13	28640952	42,505	40,576	A>G	0.19	1.00	0.98	1.03	9.3E-01	*FLT3, CHCHD2P8, LOC100420919*	intronic				Eomes,Gbx1,Nkx1-1
rs9282638	3	119263770	42,508	40,576	A>G	0.15	1.00	0.97	1.03	9.3E-01	*ADPRH, CD80, CSRP2P, TIMMDC1*	intronic		BLD		
rs944725	17	26109571	42,505	40,577	G>A	0.39	1.00	0.98	1.02	9.3E-01	*NOS2, LOC645754*	intronic		4 tissues	BRN	Maf,YY1
rs7218653	17	40425320	42,484	40,546	A>G	0.29	1.00	0.98	1.02	9.3E-01	*STAT3, STAT5A, STAT5B*	intronic	BLD	10 tissues	BLD	CTCF
rs11259245	10	6541923	42,509	40,575	A>G	0.14	1.00	0.97	1.03	9.3E-01	*PRKCQ*	intronic	MUS	4 tissues	MUS	6 altered motifs
rs7193869	16	27423501	42,343	40,420	G>A	0.28	1.00	0.98	1.02	9.3E-01	*GTF3C1, IL4R, IL21R, IL21R-AS1*	intronic		BLD		7 altered motifs
rs12401432	1	67656931	42,508	40,574	G>C	0.31	1.00	0.98	1.02	9.3E-01	*IL23R*	intronic		9 tissues	BLD	Arid5b,EWSR1-FLI1
rs9551435	13	28653242	42,285	40,333	G>A	0.21	1.00	0.98	1.03	9.3E-01	*FLT3, CHCHD2P8, LOC100420919*	intronic				Nkx2,Nkx3
rs6688383	1	67655954	42,499	40,568	A>G	0.31	1.00	0.98	1.02	9.3E-01	*IL23R*	intronic	BLD	LNG, BLD, GI	BLD,BLD	Myc
rs4955104	3	30721703	42,471	40,547	T>A	0.20	1.00	0.97	1.02	9.3E-01	*TGFBR2, GADL1*	intronic		11 tissues	MUS	Myb,Nanog
rs1075835	12	6905417	42,501	40,576	G>A	0.22	1.00	0.98	1.02	9.3E-01	*CD4, GNB3, LAG3, PTMS, MLF2, LEPREL2, GPR162*	intronic				6 altered motifs
rs11258802	10	6479507	42,508	40,576	C>G	0.14	1.00	0.97	1.03	9.3E-01	*PRKCQ*	intronic				8 altered motifs
rs1703078	12	89018883	42,509	40,576	G>C	0.06	1.00	0.96	1.04	9.3E-01	*KITLG*			11 tissues	15 tissues	5 altered motifs
rs10229457	7	22800800	42,499	40,570	G>A	0.26	1.00	0.98	1.02	9.3E-01	*IL6, RPS26P32, LOC541472*					4 altered motifs
rs3135932	11	117864063	42,501	40,563	A>G	0.17	1.00	0.97	1.03	9.3E-01	*IL10RA, TMPRSS4-AS1*	missense		4 tissues	BLD,BLD	
rs9554231	13	28640171	42,501	40,564	G>A	0.19	1.00	0.98	1.03	9.3E-01	*FLT3, CHCHD2P8, LOC100420919*	intronic				Egr-1,GR
rs2526640	7	19014843	42,506	40,575	G>A	0.22	1.00	0.98	1.03	9.3E-01	*HDAC9, NPM1P13*	intronic		7 tissues	8 tissues	Pou2f2,TATA
rs10849451	12	6524679	42,507	40,574	G>A	0.50	1.00	0.98	1.02	9.3E-01	*CD27, LTBR, SCNN1A, VAMP1, TAPBPL, RPL31P10, SRP14P1, CD27-AS1*					6 altered motifs
rs844644	1	173209495	42,507	40,572	C>A	0.47	1.00	0.98	1.02	9.3E-01	*TNFSF4, LOC100506023*	intronic				Pou5f1,ZEB1
rs2481955	13	28583581	42,501	40,572	G>A	0.28	1.00	0.98	1.02	9.4E-01	*CDX2, FLT3, PRHOXNB*	intronic		ESC, BLD, LIV		NF-I,Zbtb3
rs3917200	14	76429868	42,508	40,577	A>G	0.07	1.00	0.96	1.04	9.4E-01	*TGFB3, TTLL5, IFT43*	intronic			THYM	

SNP	Chr	Position[a]	N Cases	N Controls	Allele	MAF	OR[b]	95%CI		*p* -value	Gene annotation	dbSNP functional annotation	Promoter histone marks	Enhancer histone marks	DNAse	Motifs changed
rs11216863	11	118257081	42,504	40,574	G>A	0.08	1.00	0.97	1.04	9.4E-01	*CD3D, CD3G, UBE4A, ATP5L, MGC13053, LOC100131626*	intronic				Arid3a,Pou2f2
rs12088448	1	218546170	42,506	40,573	A>C	0.35	1.00	0.98	1.02	9.4E-01	*TGFB2, RRP15, RPS26P17, LOC728463*	intronic		15 tissues	MUS	Ets,HNF4
rs805322	2	54139895	42,499	40,566	A>G	0.50	1.00	0.98	1.02	9.4E-01	*PSME4*	intronic		ESDR, LNG, BLD		GR
rs305092	16	85979117	42,058	40,228	G>A	0.36	1.00	0.98	1.02	9.4E-01	*IRF8*		BLD, GI	5 tissues	BLD,BLD,BLD	ZBTB33
rs7209222	17	40426489	42,473	40,560	A>G	0.29	1.00	0.98	1.02	9.4E-01	*STAT3, STAT5A, STAT5B*	intronic	14 tissues	14 tissues	BLD,BLD,CRVX	Mef2,PU.1
rs1726596	7	18616374	42,505	40,575	G>A	0.09	1.00	0.97	1.04	9.4E-01	*HDAC9, LOC100419901*	intronic		BLD, GI		Irf,p300
rs2290608	3	3151759	42,505	40,575	G>A	0.25	1.00	0.98	1.02	9.4E-01	*IL5RA, TRNT1, CRBN*	5'-UTR	6 tissues	8 tissues	9 tissues	HNF1
rs702275	9	5446388	42,498	40,568	C>A	0.46	1.00	0.98	1.02	9.4E-01	*CD274, PLGRKT, LOC100419687*			BLD, SKIN, BRST	4 tissues	
rs7776857	7	22754768	42,503	40,574	A>C	0.35	1.00	0.98	1.02	9.4E-01	*IL6, LOC401312, LOC541472*			BLD, HRT, MUS		LBP-1,YY1
rs6693438	1	92163174	42,506	40,575	A>G	0.20	1.00	0.98	1.03	9.4E-01	*TGFBR3*	intronic	4 tissues	14 tissues	19 tissues	GR,Hbp1,Pou2f2
rs7569954	2	54216979	42,508	40,576	A>C	0.42	1.00	0.98	1.02	9.4E-01	*PSME4, RPL21P30*	intronic		9 tissues	BLD	Zec
rs2934194	15	40239912	42,506	40,577	G>A	0.28	1.00	0.98	1.02	9.4E-01	*GPR176, EIF2AK4, H3F3AP1, LOC100505534*	intronic		5 tissues	4 tissues	PLZF
rs4733677	8	128711821	42,417	40,523	G>A	0.24	1.00	0.98	1.02	9.4E-01	*MYC*			5 tissues	ESDR,GI,BLD	Pou2f2
rs4714565	6	42064147	42,508	40,576	G>A	0.05	1.00	0.96	1.05	9.4E-01	*CCND3, TAF8, C6orf132*			11 tissues	9 tissues	Foxp1,Smad3
rs2243204	5	131999494	42,506	40,576	G>A	0.08	1.00	0.96	1.04	9.4E-01	*IL4, IL13, RAD50, KIF3A*		BLD, LIV	13 tissues	GI,BLD,BLD	
rs887796	22	17593685	42,507	40,575	A>G	0.18	1.00	0.97	1.03	9.4E-01	*IL17RA, CECR6, CECR5, CECR5-AS1, RPL31P62, LOC100996342*	3'-UTR		BLD, LNG, GI	GI	6 altered motifs
rs7549100	1	157657842	42,402	40,457	A>G	0.24	1.00	0.98	1.02	9.4E-01	*FCRL3, SONP1, VDAC1P9*	intronic				DMRT4,Mrg
rs9655161	7	18853305	42,507	40,576	G>C	0.07	1.00	0.96	1.04	9.4E-01	*HDAC9*	intronic			BRN	Foxo,Myc
rs11868371	17	76217059	42,502	40,572	G>C	0.18	1.00	0.98	1.03	9.4E-01	*BIRC5, TK1, SYNGR2, AFMID, TMEM235, THA1P, LOC100996291*	intronic		IPSC		ERalpha-a,Hic1,TLX1::NFIC
rs1880242	7	22759607	42,502	40,574	A>C	0.50	1.00	0.98	1.02	9.4E-01	*IL6, LOC541472*			5 tissues	ESDR	4 altered motifs
rs16967620	17	40422341	42,498	40,576	C>A	0.29	1.00	0.98	1.02	9.4E-01	*STAT3, STAT5A, STAT5B*	intronic	BLD	10 tissues	11 tissues	Cdx,NF-kappaB,Sp100
rs8082391	17	40398973	42,495	40,576	C>A	0.29	1.00	0.98	1.02	9.4E-01	*STAT5A, STAT5B*	intronic	BLD, LIV	14 tissues	6 tissues	HNF4,TAL1,TCF4
rs6657275	1	218596461	42,500	40,576	A>G	0.29	1.00	0.98	1.02	9.5E-01	*TGFB2*	intronic		BRN		GR
rs7093069	10	6063319	42,485	40,550	G>A	0.19	1.00	0.97	1.03	9.5E-01	*IL2RA, IL15RA*	intronic		BLD		
rs2187557	11	118148271	42,506	40,571	G>A	0.27	1.00	0.98	1.02	9.5E-01	*CD3E, MPZL2, MPZL3*		CRVX, SKIN	12 tissues	20 tissues	Ets,GATA
rs3218100	6	41904483	42,509	40,577	G>A	0.08	1.00	0.96	1.04	9.5E-01	*BYSL, CCND3, MED20, USP49*	intronic	BLD, MUS	19 tissues	12 tissues	GCM,Pax-4,Pax-5
rs7081639	10	30784631	42,509	40,574	A>G	0.35	1.00	0.98	1.02	9.5E-01	*MAP3K8, HNRNPA1P32*			6 tissues		KAP1
rs11789689	9	101818796	42,508	40,576	G>A	0.09	1.00	0.96	1.03	9.5E-01	*COL15A1, TGFBR1*	intronic		10 tissues		STAT
rs6662137	1	218542059	42,503	40,573	A>C	0.32	1.00	0.98	1.02	9.5E-01	*TGFB2, RRP15, RPS26P17, LOC728463*	intronic		6 tissues	4 tissues	Elf5
rs2855537	12	6899181	42,509	40,576	C>A	0.22	1.00	0.98	1.02	9.5E-01	*CD4, LAG3, PTMS, MLF2, LEPREL2, GPR162*	intronic	14 tissues	9 tissues	BLD	4 altered motifs
rs584507	10	6531750	42,506	40,575	A>G	0.31	1.00	0.98	1.02	9.5E-01	*PRKCQ*	intronic		BLD		6 altered motifs
rs2026431	10	6510454	42,378	40,417	C>A	0.30	1.00	0.98	1.02	9.5E-01	*PRKCQ*	intronic				11 altered motifs
rs6731217	2	54131093	42,507	40,571	G>A	0.06	1.00	0.96	1.04	9.5E-01	*GPR75, PSME4, GPR75-ASB3*	intronic				10 altered motifs
rs1524104	7	22795153	42,503	40,573	A>G	0.14	1.00	0.97	1.03	9.5E-01	*IL6, RPS26P32, LOC541472*				IPSC	Mef2,TATA,THAP1
rs2188495	7	18540461	42,508	40,572	A>C	0.25	1.00	0.98	1.02	9.5E-01	*HDAC9*	intronic		6 tissues		7 altered motifs
rs2491222	13	28607916	42,496	40,573	A>T	0.22	1.00	0.98	1.03	9.5E-01	*FLT3, PRHOXNB, LOC100420919*	intronic		BLD		8 altered motifs
rs9533156	13	43147671	42,496	40,567	A>G	0.47	1.00	0.98	1.02	9.5E-01	*TNFSF11*	intronic	BLD	BLD, LIV	ESC	Foxo,HNF4
rs2241719	19	41829581	42,455	40,540	A>T	0.16	1.00	0.97	1.03	9.5E-01	*TGFB1, HNRNPUL1, B9D2, CCDC97, TMEM91*	3'-UTR	6 tissues	22 tissues	39 tissues	4 altered motifs
rs670804	10	6490221	42,499	40,573	A>G	0.29	1.00	0.98	1.02	9.5E-01	*PRKCQ*	intronic		THYM		Sin3Ak-20
rs1178130	7	18768788	41,969	39,883	A>G	0.21	1.00	0.98	1.03	9.5E-01	*HDAC9*	intronic				AP-1
rs3806113	6	41876335	42,508	40,577	G>A	0.25	1.00	0.98	1.02	9.5E-01	*BYSL, CCND3, MED20, USP49*	intronic				AP-2rep,Hsf
rs688879	10	6525953	42,498	40,568	G>C	0.36	1.00	0.98	1.02	9.5E-01	*PRKCQ*	intronic		BLD, THYM		9 altered motifs
rs870457	10	72311394	42,490	40,509	G>A	0.14	1.00	0.97	1.03	9.5E-01	*PRF1, PALD1*	intronic		14 tissues	10 tissues	9 altered motifs
rs4379670	1	154439865	42,499	40,577	T>A	0.20	1.00	0.98	1.03	9.5E-01	*IL6R, TDRD10, SHE*	3'-UTR		5 tissues	5 tissues	5 altered motifs
rs535068	1	12189561	42,504	40,575	A>G	0.32	1.00	0.98	1.02	9.5E-01	*TNFRSF8, TNFRSF1B*	intronic		STRM, BLD		7 altered motifs
rs162879	3	3150202	42,503	40,573	A>G	0.41	1.00	0.98	1.02	9.5E-01	*IL5RA, TRNT1, CRBN*	intronic	GI	4 tissues		CEBPB
rs2569253	5	158750993	42,487	40,563	G>A	0.49	1.00	0.98	1.02	9.5E-01	*IL12B, UBLCP1, LOC285626, RNU4ATAC2P*	intronic		MUS		Maf,Nrf-2,Pbx3
rs3025010	6	43747577	42,493	40,557	A>G	0.36	1.00	0.98	1.02	9.5E-01	*VEGFA*	intronic		12 tissues	LNG,GI	NF-I
rs2504234	13	28612246	42,506	40,576	A>G	0.18	1.00	0.97	1.03	9.5E-01	*FLT3, PRHOXNB, LOC100420919*	intronic		BLD	4 tissues	Sox
rs17045452	2	54211184	42,509	40,577	G>A	0.35	1.00	0.98	1.02	9.5E-01	*PSME4, RPL21P30*	intronic				Maf,PEBP
rs4739111	8	79758241	42,445	40,515	A>G	0.45	1.00	0.98	1.02	9.6E-01	*IL7*					Mrg1::Hoxa9,Pou5f1
rs11758083	6	43767340	42,483	40,549	G>C	0.36	1.00	0.98	1.02	9.6E-01	*VEGFA*		BLD, MUS, CRVX	16 tissues	BLD	Ets,NRSF
rs163546	3	3123326	42,508	40,577	A>G	0.30	1.00	0.98	1.02	9.6E-01	*IL5RA, TRNT1, CNTN4*	intronic	BLD	ESC, IPSC, BLD		4 altered motifs
rs6677523	1	92202467	41,711	39,712	A>G	0.11	1.00	0.97	1.03	9.6E-01	*TGFBR3*	intronic	10 tissues	20 tissues	19 tissues	SIX5
rs2148072	13	43162063	42,502	40,576	G>A	0.37	1.00	0.98	1.02	9.6E-01	*TNFSF11*	intronic		LIV		Dbx1,Pou2f2,TATA
rs3024676	16	27373558	42,510	40,576	C>A	0.17	1.00	0.97	1.03	9.6E-01	*IL4R, IL21R*	intronic		BRST, LIV, SKIN		Irf,RXRA
rs10827249	10	33654291	42,509	40,577	G>A	0.08	1.00	0.96	1.04	9.6E-01	*NRP1*				4 tissues	Rad21
rs2243248	5	132008644	42,509	40,575	A>C	0.07	1.00	0.96	1.04	9.6E-01	*IL4, IL13, RAD50, KIF3A*			GI, THYM, BLD		Osr
rs7518199	1	154407419	42,481	40,550	A>C	0.40	1.00	0.98	1.02	9.6E-01	*IL6R, SHE, PSMD8P1*	intronic	FAT, GI	16 tissues	10 tissues	NRSF

SNP	Chr	Position[a]	N Cases	N Controls	Allele	MAF	OR[b]	95%CI		*p*-value	Gene annotation	dbSNP functional annotation	Promoter histone marks	Enhancer histone marks	DNAse	Motifs changed
rs2491218	13	28604041	42,498	40,568	G>A	0.22	1.00	0.98	1.02	9.6E-01	*FLT3, PRHOXNB, LOC100420919*	intronic		FAT	PLCNT	6 altered motifs
rs943452	10	6615454	42,506	40,574	G>A	0.38	1.00	0.98	1.02	9.6E-01	*PRKCQ, PRKCQ-AS1*	intronic		5 tissues	BLD	Irf,Irx
rs7984870	13	43146482	42,496	40,565	G>C	0.47	1.00	0.98	1.02	9.6E-01	*TNFSF11*	intronic				9 altered motifs
rs2810885	1	92164498	42,505	40,577	A>T	0.31	1.00	0.98	1.02	9.6E-01	*TGFBR3*	intronic		11 tissues		GATA,Znf143
rs10948095	6	43767703	42,376	40,426	A>G	0.39	1.00	0.98	1.02	9.6E-01	*VEGFA*				7 tissues	5 altered motifs
rs10258269	7	18855915	42,362	40,380	G>C	0.11	1.00	0.97	1.03	9.6E-01	*HDAC9*	intronic				BATF,EWSR1-FLI1,Irf
rs17520924	1	186593131	42,509	40,576	G>A	0.12	1.00	0.97	1.03	9.6E-01	*PTGS2, LOC100131939*			BLD		GR,Maf
rs4845141	1	206972072	42,506	40,576	G>A	0.46	1.00	0.98	1.02	9.6E-01	*IL10, IL19*		BLD	4 tissues		Gfi1,Gfi1b,RREB-1
rs284176	1	92223976	42,196	40,303	G>A	0.32	1.00	0.98	1.02	9.6E-01	*TGFBR3*	intronic		13 tissues	BLD,SKIN,MUS	5 altered motifs
rs2491236	13	28611738	42,503	40,573	A>G	0.18	1.00	0.97	1.03	9.6E-01	*FLT3, PRHOXNB, LOC100420919*	intronic		5 tissues	16 tissues	5 altered motifs
rs6602392	10	6078079	42,454	40,522	C>A	0.10	1.00	0.97	1.03	9.6E-01	*IL2RA, RPL32P23*	intronic		5 tissues	THYM	Pou2f2
rs6689393	1	154426097	42,507	40,574	G>A	0.43	1.00	0.98	1.02	9.6E-01	*IL6R, TDRD10, SHE, PSMD8P1*	intronic	4 tissues	17 tissues	9 tissues	5 altered motifs
rs2526633	7	19030751	42,427	40,498	G>C	0.33	1.00	0.98	1.02	9.6E-01	*HDAC9, NPM1P13*	intronic			PANC	Cdx,Zfp691
rs3754014	1	92171420	42,362	40,428	T>A	0.23	1.00	0.98	1.02	9.7E-01	*TGFBR3*	intronic		5 tissues	HRT	CEBPB,Foxa,Irf
rs9554236	13	28641775	42,498	40,551	A>G	0.19	1.00	0.98	1.03	9.7E-01	*FLT3, CHCHD2P8, LOC100420919*	intronic		MUS	MUS,MUS	HNF4,PLZF
rs4845622	1	154411419	42,502	40,568	A>C	0.40	1.00	0.98	1.02	9.7E-01	*IL6R, SHE, PSMD8P1*	intronic		4 tissues		Foxm1
rs6602437	10	6130077	42,498	40,559	A>G	0.45	1.00	0.98	1.02	9.7E-01	*IL2RA, RBM17, RPL32P23*			BLD		CEBPB,Foxp1,Irf
rs6740847	2	182308352	42,458	40,508	G>A	0.43	1.00	0.98	1.02	9.7E-01	*ITGA4*			BLD		10 altered motifs
rs6073991	20	44656112	42,337	40,436	A>G	0.22	1.00	0.98	1.03	9.7E-01	*MMP9, SLC12A5, NCOA5, LOC100128028*	intronic			49 tissues	SZF1-1,T3R
rs1041429	21	34699909	42,510	40,577	G>A	0.06	1.00	0.96	1.04	9.7E-01	*IFNAR1, IL10RB, USF1P1*	intronic			4 tissues	4 altered motifs
rs677986	10	6477267	42,505	40,574	A>G	0.26	1.00	0.98	1.02	9.7E-01	*PRKCQ*	intronic		FAT	PANC	NF-AT1
rs17884213	1	12263545	42,503	40,575	G>A	0.26	1.00	0.98	1.02	9.7E-01	*TNFRSF1B, VPS13D, LOC390998, MIR4632*	intronic		8 tissues	BLD	9 altered motifs
rs10179642	2	9683696	42,508	40,576	A>G	0.16	1.00	0.97	1.03	9.7E-01	*ADAM17, YWHAQ*	intronic				
rs7088910	10	6030194	42,509	40,573	G>A	0.33	1.00	0.98	1.02	9.7E-01	*IL2RA, IL15RA*		BRN, BLD	7 tissues	BLD,BLD	4 altered motifs
rs944724	17	26109417	42,508	40,575	G>A	0.22	1.00	0.98	1.02	9.7E-01	*NOS2, LOC645754*	intronic		4 tissues		Bcl6b,GATA,STAT
rs624968	19	6623888	42,094	40,141	G>C	0.45	1.00	0.98	1.02	9.7E-01	*CD70, TNFSF14, RPL7P50*					NF-I
rs3794763	17	26111226	42,501	40,572	G>A	0.21	1.00	0.98	1.02	9.7E-01	*NOS2, LOC645754*	intronic		ESDR, ESC, LNG	ADRL,OVRY,LNG	4 altered motifs
rs7538756	1	92186241	42,507	40,571	G>A	0.38	1.00	0.98	1.02	9.7E-01	*TGFBR3*	intronic		LIV		GR
rs10208649	2	54161363	42,345	40,357	A>G	0.07	1.00	0.96	1.04	9.7E-01	*PSME4*	intronic				
rs3021094	1	206944952	42,509	40,576	A>C	0.09	1.00	0.97	1.04	9.7E-01	*IL10, MAPKAPK2, IL19*	intronic	BLD, LIV, GI	4 tissues	BLD	4 altered motifs
rs602128	11	102713465	40,819	38,872	G>A	0.50	1.00	0.98	1.02	9.7E-01	*MMP1, MMP3, MMP12, CSNK1A1P2, WTAPP1, LOC100288111*	synonymous		BRST, SKIN		ATF3,PU.1
rs11264798	1	157661848	42,503	40,575	C>G	0.50	1.00	0.98	1.02	9.7E-01	*FCRL3, SONP1, VDAC1P9*	intronic		BLD, BRN		Hoxb9,Mef2,NRSF
rs6503692	17	40421513	42,498	40,573	G>A	0.29	1.00	0.98	1.02	9.7E-01	*STAT3, STAT5A, STAT5B*	intronic	BLD, LIV	8 tissues	4 tissues	5 altered motifs
rs17676026	1	157666336	42,497	40,568	C>G	0.20	1.00	0.97	1.02	9.7E-01	*FCRL2, FCRL3, SONP1, VDAC1P9*	intronic	BLD	BLD		5 altered motifs
rs3742257	13	43173198	42,499	40,574	A>G	0.46	1.00	0.98	1.02	9.7E-01	*TNFSF11*	intronic		IPSC		LBP-1
rs6979890	7	18693393	42,460	40,548	A>G	0.22	1.00	0.98	1.02	9.7E-01	*HDAC9*	intronic				NF-kappaB
rs12032588	1	92355156	42,497	40,560	C>A	0.40	1.00	0.98	1.02	9.7E-01	*TGFBR3*	intronic		7 tissues		Pax-5
rs12722574	10	6066462	42,494	40,563	G>A	0.21	1.00	0.98	1.03	9.7E-01	*IL2RA, IL15RA, RPL32P23*	intronic		SKIN, BRN	OVRY,SKIN	Irf,TEF
rs9554229	13	28639249	42,506	40,574	A>G	0.19	1.00	0.98	1.03	9.7E-01	*FLT3, CHCHD2P8, LOC100420919*	intronic				HDAC2
rs12430855	13	28662007	42,505	40,563	G>A	0.21	1.00	0.98	1.03	9.7E-01	*FLT3, CHCHD2P8, PAN3-AS1, LOC100420919*	intronic			PANC	6 altered motifs
rs10911901	1	186624006	42,505	40,575	A>C	0.14	1.00	0.97	1.03	9.7E-01	*PTGS2*				BLD,BLD	
rs2799090	1	218568829	42,498	40,546	A>G	0.27	1.00	0.98	1.02	9.7E-01	*TGFB2, LOC728463*	intronic		5 tissues	LNG	
rs73174426	13	43144867	42,509	40,576	G>A	0.08	1.00	0.96	1.04	9.7E-01	*TNFSF11*	intronic				
rs1148476	1	12186797	42,498	40,574	G>A	0.23	1.00	0.98	1.02	9.7E-01	*TNFRSF8, TNFRSF1B*	intronic	BLD, FAT, GI	5 tissues		5 altered motifs
rs2057482	14	62213848	42,510	40,576	G>A	0.13	1.00	0.97	1.03	9.7E-01	*HIF1A, SNAPC1, HIF1A-AS2*	3'-UTR	HRT	6 tissues	SKIN,BLD,MUS	Nanog,p300
rs2957125	18	60058342	42,500	40,569	T>A	0.44	1.00	0.98	1.02	9.7E-01	*TNFRSF11A, RPL17P44*			BLD, GI	IPSC,GI	Nrf-2
rs5746078	1	12270222	42,499	40,567	G>A	0.08	1.00	0.96	1.04	9.7E-01	*TNFRSF1B, VPS13D, LOC390998, MIR4632*			4 tissues	5 tissues	BDP1,EWSR1-FLI1,GR
rs9529	6	41903007	42,508	40,573	G>A	0.27	1.00	0.98	1.02	9.7E-01	*BYSL, CCND3, MED20, USP49*	3'-UTR		14 tissues	MUS,BLD	4 altered motifs
rs1977389	10	90773494	42,499	40,572	A>C	0.39	1.00	0.98	1.02	9.7E-01	*ACTA2, FAS, FAS-AS1, MIR4679-1, MIR4679-2*	intronic		FAT		6 altered motifs
rs6491247	13	28606521	42,507	40,576	G>A	0.22	1.00	0.98	1.02	9.7E-01	*FLT3, PRHOXNB, LOC100420919*	intronic	BLD	BLD		
rs16967611	17	40401567	42,474	40,551	A>G	0.29	1.00	0.98	1.02	9.8E-01	*STAT5A, STAT5B*	intronic	BLD	13 tissues	5 tissues	RXRA,STAT
rs1148459	1	12217852	42,484	40,561	C>A	0.47	1.00	0.98	1.02	9.8E-01	*TNFRSF8, TNFRSF1B, MIR4632*		BLD	15 tissues	9 tissues	Pax-4
rs9856760	3	45906356	42,507	40,575	A>G	0.22	1.00	0.98	1.02	9.8E-01	*CCR9, SDHDP4, LZTFL1*		THYM	BLD	THYM,BLD,BLD	HNF1,Hoxa4
rs2295359	1	67635950	42,504	40,574	G>A	0.34	1.00	0.98	1.02	9.8E-01	*IL23R, C1orf141*	intronic	BLD, LIV	BLD, ADRL, LIV		GATA,Hoxd10,TAL1
rs12001295	9	5518260	42,507	40,576	G>A	0.06	1.00	0.96	1.04	9.8E-01	*CD274, PDCD1LG2*	intronic	FAT, SKIN, BLD	12 tissues	12 tissues	EBF
rs3856848	3	3147676	42,496	40,574	G>A	0.20	1.00	0.98	1.03	9.8E-01	*IL5RA, TRNT1, CRBN, CNTN4*	intronic		BLD		Arnt,Mxi1,Myc
rs4453032	1	154414086	42,503	40,575	A>G	0.40	1.00	0.98	1.02	9.8E-01	*IL6R, SHE, PSMD8P1*	intronic	BLD	12 tissues		Maf,PPAR,PU.1
rs6973063	7	18697589	42,497	40,571	A>G	0.22	1.00	0.98	1.02	9.8E-01	*HDAC9*	intronic		4 tissues		5 altered motifs
rs3132940	6	32161396	42,508	40,565	C>A	0.14	1.00	0.97	1.03	9.8E-01	*AGER, NOTCH4, PBX2, RNF5, PPT2, AGPAT1, GPSM3, PRRT1, EGFL8, LOC100507547, PPT2-EGFL8*	intronic	4 tissues	13 tissues		8 altered motifs

SNP	Chr	Position[a]	N Cases	N Controls	Allele	MAF	OR[b]	95%CI		*p* -value	Gene annotation	dbSNP functional annotation	Promoter histone marks	Enhancer histone marks	DNAse	Motifs changed
rs4620621	10	6616336	42,373	40,427	A>G	0.40	1.00	0.98	1.02	9.8E-01	*PRKCQ, PRKCQ-AS1*	intronic		4 tissues	THYM	
rs10889664	1	67635475	42,508	40,574	G>A	0.34	1.00	0.98	1.02	9.8E-01	*IL23R, C1orf141*	intronic			GI	
rs17375018	1	67655147	42,500	40,572	G>A	0.31	1.00	0.98	1.02	9.8E-01	*IL23R*	intronic		BLD		GATA,Hand1,Pbx-1
rs10506955	12	88929026	41,321	39,470	A>G	0.10	1.00	0.97	1.03	9.8E-01	*KITLG*	intronic			BRN	AP-3,SIX5
rs7599335	2	54141637	41,954	40,006	G>C	0.07	1.00	0.96	1.04	9.8E-01	*PSME4*	intronic		BLD, VAS		Foxp1
rs8064638	17	40424255	42,503	40,575	G>A	0.29	1.00	0.98	1.02	9.8E-01	*STAT3, STAT5A, STAT5B*	intronic	BLD, GI	11 tissues	BLD,CRVX,BLD	GR
rs9472131	6	43777500	42,502	40,574	G>A	0.29	1.00	0.98	1.02	9.8E-01	*VEGFA*			14 tissues		HEY1,Pou2f2,Znf143
rs10889668	1	67661244	42,501	40,566	G>A	0.13	1.00	0.97	1.03	9.8E-01	*IL23R*	intronic			BLD	
rs4683148	3	45956060	42,485	40,558	G>A	0.39	1.00	0.98	1.02	9.8E-01	*CXCR6, CCR9, SDHDP4, FYCO1*		GI, THYM	BLD	THYM	5 altered motifs
rs2785665	1	157623017	42,489	40,568	A>C	0.45	1.00	0.98	1.02	9.8E-01	*FCRL3*		BLD	BLD, THYM	7 tissues	
rs2491230	13	28609997	42,450	40,535	G>A	0.22	1.00	0.98	1.02	9.8E-01	*FLT3, PRHOXNB, LOC100420919*	intronic		BLD		
rs17573483	1	92218245	42,507	40,573	G>A	0.18	1.00	0.97	1.03	9.8E-01	*TGFBR3*	intronic		6 tissues	BLD	Maf
rs801523	7	18703723	42,322	40,430	C>A	0.08	1.00	0.96	1.04	9.8E-01	*HDAC9*	intronic				Foxa,GATA
rs2506146	10	33474952	42,505	40,569	G>C	0.10	1.00	0.97	1.03	9.8E-01	*NRP1*	intronic		FAT, SKIN	IPSC	DMRT2,Maf
rs2064689	1	67653010	42,502	40,576	G>A	0.31	1.00	0.98	1.02	9.8E-01	*IL23R*	intronic				4 altered motifs
rs6491248	13	28612457	42,504	40,574	G>A	0.18	1.00	0.97	1.03	9.8E-01	*FLT3, PRHOXNB, LOC100420919*	intronic		IPSC		Pou2f2
rs9906933	17	40410045	42,487	40,555	G>A	0.29	1.00	0.98	1.02	9.8E-01	*STAT5A, STAT5B*	intronic		BLD, LNG		
rs3856845	3	3133006	42,505	40,576	G>A	0.12	1.00	0.97	1.03	9.8E-01	*IL5RA, TRNT1, CNTN4*	intronic				Foxi1,TATA
rs3758483	10	90748736	42,506	40,576	A>G	0.14	1.00	0.97	1.03	9.8E-01	*ACTA2, FAS, ACTA2-AS1, FAS-AS1*	intronic	FAT	11 tissues	LNG	5 altered motifs
rs7831912	8	128703679	42,505	40,571	A>G	0.07	1.00	0.96	1.04	9.8E-01	*MYC*					
rs9513000	13	28621937	42,506	40,569	G>C	0.20	1.00	0.97	1.03	9.8E-01	*FLT3, LOC100420919*	intronic		BLD	4 tissues	NF-AT,SIX5,Znf143
rs61773703	1	11281952	42,122	40,102	G>A	0.12	1.00	0.97	1.03	9.8E-01	*MTOR, ANGPTL7, RPL39P6*	intronic				Mef2,Pax-8
rs386574	19	6649564	42,501	40,569	A>G	0.29	1.00	0.98	1.02	9.8E-01	*C3, TNFSF14*			BLD	BLD	AP-1,Bach1
rs876685	1	23072420	42,508	40,574	A>G	0.11	1.00	0.97	1.03	9.8E-01	*EPHB2, MIR4684*	intronic	BLD	13 tissues	4 tissues	6 altered motifs
rs10503068	18	59951133	42,505	40,573	A>G	0.23	1.00	0.98	1.02	9.8E-01	*TNFRSF11A, KIAA1468*	intronic				Barhl1,Pbx-1
rs4648276	1	186645488	42,508	40,577	A>G	0.12	1.00	0.97	1.03	9.8E-01	*PTGS2*	intronic		BRST, BLD, VAS		TBX5
rs2799098	1	218521609	42,502	40,573	A>G	0.18	1.00	0.97	1.03	9.8E-01	*TGFB2, RRP15, RPS26P17, LOC728463*	intronic	17 tissues	4 tissues	25 tissues	
rs4571935	1	36963878	42,503	40,573	T>A	0.45	1.00	0.98	1.02	9.9E-01	*CSF3R, MRPS15, OSCP1*					Hbp1,Irf
rs9579149	13	28630540	42,500	40,570	A>C	0.20	1.00	0.98	1.03	9.9E-01	*FLT3, LOC100420919*	intronic		5 tissues		
rs12129467	1	11212697	42,508	40,576	G>C	0.09	1.00	0.97	1.04	9.9E-01	*MTOR, ANGPTL7, MTOR-AS1*	intronic		16 tissues	MUS	RFX5,RXRA,TFII-I
rs11216855	11	118208750	42,442	40,512	A>G	0.44	1.00	0.98	1.02	9.9E-01	*CD3D, CD3E, CD3G, UBE4A, LOC100131626*			BLD, THYM		AP-1,HES1
rs4256810	1	206890449	42,498	40,568	A>G	0.32	1.00	0.98	1.02	9.9E-01	*MAPKAPK2, RPS14P1*	intronic	BLD	19 tissues	LNG,HRT,CRVX	5 altered motifs
rs7096751	10	6438493	42,508	40,576	A>G	0.11	1.00	0.97	1.03	9.9E-01	*PRKCQ, DKFZp667F0711*		BLD	5 tissues	5 tissues	
rs8001973	13	28650549	42,507	40,575	A>C	0.21	1.00	0.98	1.03	9.9E-01	*FLT3, CHCHD2P8, LOC100420919*	intronic				
rs2240281	7	19005937	42,509	40,574	C>A	0.18	1.00	0.97	1.03	9.9E-01	*HDAC9, NPM1P13*	intronic		ESDR		Elf3,Ik-1,PRDM1
rs9525641	13	43148024	42,488	40,551	A>G	0.47	1.00	0.98	1.02	9.9E-01	*TNFSF11*	intronic	5 tissues	6 tissues	ESC,ESDR,ESC	FXR
rs9513027	13	28648669	42,502	40,572	G>A	0.21	1.00	0.98	1.02	9.9E-01	*FLT3, CHCHD2P8, LOC100420919*	intronic				
rs3177980	1	169676486	42,506	40,574	A>G	0.27	1.00	0.98	1.02	9.9E-01	*SELE, SELL*					
rs10827246	10	33642629	42,510	40,575	G>A	0.08	1.00	0.96	1.04	9.9E-01	*NRP1*					E2F
rs627715	10	6555831	42,489	40,563	G>A	0.13	1.00	0.97	1.03	9.9E-01	*PRKCQ*	intronic	MUS	5 tissues	6 tissues	AP-1
rs12405141	1	186623470	42,506	40,576	A>G	0.12	1.00	0.97	1.03	9.9E-01	*PTGS2*			BLD		HDAC2,Myb
rs2541322	7	18750718	42,509	40,577	C>A	0.10	1.00	0.97	1.03	9.9E-01	*HDAC9*	intronic				Nkx2,Nkx3
rs2191032	3	45906572	42,503	40,570	A>G	0.22	1.00	0.98	1.02	9.9E-01	*CCR9, SDHDP4, LZTFL1*		THYM, BLD	BLD, GI	4 tissues	GR
rs2069837	7	22768027	42,509	40,577	A>G	0.07	1.00	0.96	1.04	9.9E-01	*IL6, RPS26P32, LOC541472*	intronic	11 tissues	9 tissues	5 tissues	15 altered motifs
rs684014	1	12224303	42,506	40,574	C>A	0.10	1.00	0.97	1.03	9.9E-01	*TNFRSF8, TNFRSF1B, MIR4632*		BLD	7 tissues	PLCNT	18 altered motifs
rs17351243	1	206959527	42,500	40,574	G>A	0.46	1.00	0.98	1.02	9.9E-01	*IL10, IL19*			BLD, THYM, SPLN	BLD	6 altered motifs
rs4072391	1	154438880	42,508	40,576	A>G	0.20	1.00	0.98	1.03	9.9E-01	*IL6R, TDRD10, SHE*	3'-UTR	BLD	11 tissues	5 tissues	TEF-1
rs5746065	1	12267464	41,938	40,104	C>A	0.10	1.00	0.97	1.03	9.9E-01	*TNFRSF1B, VPS13D, LOC390998, MIR4632*	3'-UTR		ESDR, IPSC, SPLN		6 altered motifs
rs10489678	1	157669668	42,497	40,569	G>A	0.19	1.00	0.97	1.03	9.9E-01	*FCRL2, FCRL3, SONP1, VDAC1P9*	intronic			BLD,MUS,MUS	5 altered motifs
rs7226991	18	59988691	42,506	40,575	G>A	0.30	1.00	0.98	1.02	9.9E-01	*TNFRSF11A, KIAA1468*			6 tissues	5 tissues	PRDM1
rs12770958	10	30706590	42,492	40,577	C>A	0.16	1.00	0.97	1.03	9.9E-01	*CCND3P, MAP3K8, MKI67IPP1, LOC729668*			11 tissues	PANC	NF-kappaB,STAT
rs10003233	4	142679432	42,500	40,574	G>A	0.39	1.00	0.98	1.02	9.9E-01	*IL15*					11 altered motifs
rs2296622	1	92161578	42,508	40,576	A>G	0.23	1.00	0.98	1.02	9.9E-01	*TGFBR3*	intronic		ESDR, FAT		AP-1,CEBPG,Foxj1
rs12531908	7	18838616	42,495	40,571	A>G	0.28	1.00	0.98	1.02	9.9E-01	*HDAC9*	intronic		LNG, BRN, BONE	4 tissues	HDAC2,HNF4
rs983751	10	90746693	42,505	40,569	C>A	0.14	1.00	0.97	1.03	9.9E-01	*ACTA2, FAS, ACTA2-AS1, FAS-AS1*	intronic	VAS	7 tissues	6 tissues	6 altered motifs
rs17817076	2	191851997	42,510	40,576	G>A	0.47	1.00	0.98	1.02	9.9E-01	*GLS, STAT1, STAT4, LOC100420571*	intronic		BLD, LIV		5 altered motifs
rs4750439	10	6469324	42,509	40,574	G>A	0.25	1.00	0.98	1.02	9.9E-01	*PRKCQ*	3'-UTR		MUS		
rs10956387	8	128775144	42,504	40,574	G>A	0.42	1.00	0.98	1.02	9.9E-01	*MYC, MIR1204*					STAT
rs3780869	10	33547465	42,506	40,576	G>A	0.15	1.00	0.97	1.03	9.9E-01	*NRP1*	intronic		10 tissues	ADRL,BRN	AIRE,Mrg1::Hoxa9,Pax-3
rs2270502	16	85933038	42,498	40,572	G>A	0.06	1.00	0.96	1.04	1.0E+00	*IRF8*	intronic	14 tissues	4 tissues	13 tissues	DBP,Foxp1
rs1468488	22	17590744	42,502	40,568	A>G	0.28	1.00	0.98	1.02	1.0E+00	*IL17RA, CECR6, CECR5, CECR5-AS1, RPL31P62, LOC100996342*	3'-UTR		ESC, BLD, MUS		CAC-binding-protein

SNP	Chr	Position[a]	N Cases	N Controls	Allele	MAF	OR[b]	95%CI		*p* -value	Gene annotation	dbSNP functional annotation	Promoter histone marks	Enhancer histone marks	DNAse	Motifs changed
rs2307104	15	40282701	42,506	40,576	A>G	0.49	1.00	0.98	1.02	1.0E+00	*SRP14, EIF2AK4, H3F3AP1, SRP14-AS1*	intronic				CEBPA,STAT
rs3025035	6	43751359	42,507	40,577	G>A	0.07	1.00	0.96	1.04	1.0E+00	*VEGFA*	intronic		5 tissues		Foxj2
rs801524	7	18706918	42,464	40,532	G>A	0.19	1.00	0.97	1.03	1.0E+00	*HDAC9*	3'-UTR		11 tissues		4 altered motifs
rs9581967	13	28629327	42,510	40,575	C>G	0.20	1.00	0.98	1.03	1.0E+00	*FLT3, LOC100420919*	intronic		ESC, IPSC, BLD		TCF4
rs11648716	16	85906616	42,510	40,576	A>G	0.08	1.00	0.96	1.04	1.0E+00	*IRF8*		BLD	6 tissues	4 tissues	5 altered motifs
rs305094	16	85978105	42,501	40,575	A>G	0.33	1.00	0.98	1.02	1.0E+00	*IRF8*		BLD	BLD, LIV		Mtf1,Sox
rs2494740	14	105247881	42,403	40,482	T>A	0.30	1.00	0.98	1.02	1.0E+00	*AKT1, SIVA1, ADSSL1, LINC00638, ZBTB42, RPS26P49*	intronic		5 tissues		
rs8106574	19	6671142	42,394	40,486	G>A	0.24	1.00	0.98	1.02	1.0E+00	*C3, TNFSF14*		BLD	5 tissues	5 tissues	5 altered motifs
rs5275	1	186643058	42,098	40,100	A>G	0.34	1.00	0.98	1.02	1.0E+00	*PTGS2*	3'-UTR				HMG-IY,Mrg1::Hoxa9,Pax-4
rs3852251	7	18858570	42,487	40,555	C>A	0.07	1.00	0.96	1.04	1.0E+00	*HDAC9*	intronic				Mxi1,SREBP
rs284152	1	92274817	42,504	40,573	A>G	0.32	1.00	0.98	1.02	1.0E+00	*TGFBR3*	intronic		17 tissues		4 altered motifs
rs6669994	1	198638257	42,508	40,576	C>G	0.16	1.00	0.97	1.03	1.0E+00	*PTPRC, PEBP1P3*	intronic	BLD	14 tissues	25 tissues	4 altered motifs
rs2250232	4	15879076	42,393	40,450	A>C	0.23	1.00	0.98	1.02	1.0E+00	*CD38, LOC100130067*				BRN	6 altered motifs

[a] Build 37

[b] OR adjusted for age, study and nine European principal components.

[c] The association for rs284147 is likely to be an artifact. The cluster plot of rs284147 shows that most genotypes that could not be called are homozygous wild type.

SNP Single nucleotide polymorphism, Chr. chromosome, MAF minor allele frequency, OR odds ratio, CI confidence interval, UTR untranslated region
