# Supplementary material for: Genetic variation in the immunosuppression pathway genes and breast cancer susceptibility: a pooled analysis of 42,510 cases and 40,577 controls from the Breast Cancer Association Consortium
Source: Hum Genet. 2015 Nov 30;135:137–54. doi: 10.1007/s00439-015-1616-8 (PMC4698282; doi:10.1007/s00439-015-1616-8)
Supplement: Supplementary file 5 — ESM_5_TopSNPs_ERstatus.pdf Associations of TGFBR2, CCND1 and STAT3 SNPs with overall breast cancer risk as well as stratified by ER status [file 439_2015_1616_MOESM5_ESM.pdf]

Table S4 *TGFBR2* , *CCND1* and *STAT3* SNPs associated with overall breast cancer risk stratified by ER status.

SNP	Chr.	Position	Gene	Minor allele	MAF	ER-postive breast cancer		ER-negative breast cancer		Case-only analysis (ER+ vs ER-)
						OR[a] (95%CI)	*p* -value	OR[a] (95%CI)	*p* -value	*p* -value
rs1431131	3	30675880	*TGFBR2*	A	0.36	1.06 (1.04-1.09)	2.7×10^{-7}	1.01 (0.97-1.05)	0.61	0.01
rs1192442[illegible]	3	30677484	*TGFBR2*	C	0.41	0.94 (0.92-0.97)	1.2×10^{-6}	0.99 (0.95-1.03)	0.69	0.02
rs7177	11	69466115	*CCND1*	C	0.47	0.95 (0.93-0.97)	8.2×10^{-6}	0.99 (0.95-1.03)	0.61	0.04
rs1905339	17	40582296	*STAT3*	G	0.33	1.05 (1.03-1.08)	5.3×10^{-5}	1.04 (1.00-1.08)	0.07	0.62

[a] OR adjusted for age, study and nine European principal components.
ER, estrogen receptor; SNP, single nucleotide polymorphism; Chr., chromosome; MAF, minor allele frequency; OR, odds ratio; CI, confidence interval; TGFBR2, transforming growth factor beta receptor II; CCND1, cyclin D1; STAT3, signal transducer and activator of transcription 3.
