# Supplementary material for: Genetic variation in the immunosuppression pathway genes and breast cancer susceptibility: a pooled analysis of 42,510 cases and 40,577 controls from the Breast Cancer Association Consortium
Source: Hum Genet. 2015 Nov 30;135:137–54. doi: 10.1007/s00439-015-1616-8 (PMC4698282; doi:10.1007/s00439-015-1616-8)
Supplement: Supplementary file 17 — ESM_17_Gene_level_associations.pdf Gene-level associations with breast cancer risk for 133 candidate genes in the immunosuppression pathway [file 439_2015_1616_MOESM17_ESM.pdf]

Table S5 Gene-level associations of 133 immunosuppression pathway candidate genes for overall breast cancer, ER-positive and ER-negative disease.

Gene name	Entrez Id	Chr.	Number of SNPs	Overall	ER-positive	ER-negative
				p -value	*p* -value	*p* -value
TGFBR2	7048	3	128	$<10^{-6}$	<0.001	0.22
CCND1	595	11	83	3.0×10^{-4}	<0.001	0.25
IL5	3567	5	7	0.001	0.08	0.12
GM-CSF	1437	5	9	0.007	0.002	0.35
IL8RA	3579	2	14	0.01	0.01	0.66
MMP3	4314	11	27	0.02	0.09	0.22
EPHB2	2048	1	22	0.02	0.02	0.27
AKT	207	14	38	0.03	0.13	0.02
CD304	8829	10	42	0.03	0.29	0.003
STAT3	6774	17	48	0.03	0.03	0.68
MYD88	4615	3	2	0.04	0.18	0.10
CD38	952	4	9	0.05	0.05	0.16
KITLG	4254	12	75	0.05	0.05	0.16
CSF3	1440	17	12	0.05	0.15	0.09
IL18R1	8809	2	6	0.06	0.01	0.56
CCR9	10803	3	19	0.07	0.28	0.12
STAT1	6772	2	37	0.08	0.15	0.01
IL12A	3592	3	11	0.08	0.47	0.19
TGFB2	7042	1	58	0.08	0.31	0.32
CD45R	5788	1	14	0.10	0.13	0.57
GAL10	1178	19	8	0.12	0.13	0.78
IL6	3569	7	41	0.13	0.15	0.39
mTOR	2475	1	18	0.14	0.14	0.95
IL4	3565	5	22	0.15	0.48	0.57
VEGF	7422	6	44	0.15	0.42	0.13
IL1B	3553	2	17	0.15	0.07	0.83
IL23R	149233	1	60	0.15	0.38	0.24
HuR	1994	19	9	0.16	0.27	0.93
HLA-DRA	3122	6	23	0.16	0.09	0.34
CXCR1	3577	2	1	0.16	0.05	0.27
IL7R	3575	5	51	0.17	0.15	0.38
IL15	3600	4	13	0.17	0.08	0.22
CD274	29126	9	47	0.17	0.70	0.29
PRKCQ	5588	10	170	0.17	0.12	0.96
CD69	969	12	5	0.18	0.11	0.56
TGFBR3	7049	1	301	0.18	0.33	0.45
LGALS9	3965	17	15	0.18	0.36	0.94
CD73	4907	6	5	0.18	0.09	0.61
CD62L	6402	1	10	0.18	0.20	0.91
FOXP3	50943	X	4	0.19	0.33	0.52
IL23A	51561	12	1	0.20	0.05	0.43
RAGE	177	6	16	0.20	0.19	0.03
MMP12	4321	11	29	0.21	0.22	0.35
IL10RA	3587	11	20	0.22	0.31	0.11
GZMB	3002	14	12	0.22	0.03	0.86

Gene name	Entrez Id	Chr.	Number of SNPs	Overall	ER-positive	ER-negative
				p -value	*p* -value	*p* -value
TNFRSF11A	8792	18	57	0.22	0.31	0.47
CD95	355	10	35	0.23	0.10	0.22
CD40L	959	X	5	0.24	0.52	0.22
TNFRSF1B	7133	1	26	0.25	0.33	0.35
CCR4	1233	3	1	0.25	0.38	0.20
CD4	920	12	1	0.26	0.39	0.95
IL2RA	3559	10	172	0.26	0.07	0.64
BIRC5	332	17	22	0.27	0.24	0.65
HIF1a	3091	14	14	0.27	0.11	0.84
IL13	3596	5	1	0.28	0.18	0.98
CTLA4	1493	2	12	0.28	0.26	0.63
CD86	942	3	14	0.28	0.09	0.33
IL4R	3566	16	46	0.28	0.08	0.79
CCR6	1235	6	10	0.29	0.12	0.28
ERK2	5594	22	30	0.32	0.59	0.45
CXCR4	7852	2	2	0.33	0.65	0.29
CD49d	3676	2	13	0.35	0.38	0.38
LAG3	3902	12	6	0.35	0.26	0.48
IRAK1	3654	X	4	0.36	0.44	0.60
IL5RA	3568	3	62	0.38	0.15	0.69
COX2	5743	1	15	0.38	0.38	0.33
IL19	29949	1	26	0.39	0.21	0.50
CD3G	917	11	16	0.39	0.48	0.26
MAPKAPK2	9261	1	4	0.39	0.73	0.78
CXCL12	6387	10	19	0.39	0.18	0.20
TGFBR1	7046	9	42	0.39	0.25	0.74
IL12RB2	3595	1	14	0.42	0.23	0.32
ADAM17	6868	2	10	0.42	0.64	0.27
CXCL10	3627	4	1	0.43	0.19	0.75
BCL-XL	598	20	12	0.44	0.60	0.59
B7-H4	79679	1	2	0.46	0.97	0.03
IL7	3574	8	45	0.47	0.17	0.95
IL10RB	3588	21	14	0.48	0.78	0.81
GZMA	3001	5	4	0.48	0.44	0.62
S100A8	6279	1	8	0.50	0.48	0.50
IFNA1	3439	9	4	0.52	0.54	0.16
CD40	958	20	15	0.54	0.45	0.87
CD28	940	2	8	0.56	0.62	0.64
GARP	2615	11	9	0.56	0.90	0.43
IRF8	3394	16	56	0.57	0.39	0.17
MMP1	4312	11	4	0.58	0.22	0.35
STAT6	6778	12	1	0.58	0.41	0.99
IL13RA2	3598	X	7	0.61	0.37	0.86
CD31	5175	17	7	0.62	0.36	0.31
CSF3R	1441	1	10	0.64	0.76	0.05
PRF1	5551	10	3	0.65	0.92	0.39
CD2	914	1	3	0.65	0.42	0.39
PD1	5133	2	4	0.65	0.86	0.10

Gene name	Entrez Id	Chr.	Number of SNPs	Overall	ER-positive	ER-negative
				p -value	*p* -value	*p* -value
MYC	4609	8	49	0.67	0.21	0.85
CD80	941	3	11	0.67	0.40	0.87
iNOS	4843	17	32	0.67	0.56	0.74
LGALS1	3956	22	5	0.67	0.57	0.65
CCR7	1236	17	1	0.68	0.83	0.46
HDAC9	9734	7	416	0.69	0.90	0.51
TNFSF4	7292	1	14	0.70	0.75	0.18
EIF2A	83939	3	11	0.72	0.79	0.70
STAT5B	6777	17	7	0.73	0.93	0.13
ICOS	29851	2	5	0.75	0.58	0.61
B9D2	80776	19	8	0.75	0.39	0.91
TGFB3	7043	14	22	0.75	0.96	0.82
GITR	8784	1	9	0.75	0.42	0.07
IL17RA	23765	22	26	0.75	0.84	0.33
CCND3	896	6	13	0.77	0.92	0.42
IL2	3558	4	13	0.77	0.91	0.28
CD27	939	12	13	0.79	0.49	0.31
EIF2AK4	440275	15	72	0.81	0.65	0.36
CD103	3682	17	7	0.83	0.77	0.50
MMP9	4318	20	24	0.83	0.75	0.33
MAP3K8	1326	10	11	0.83	0.78	0.78
NOX2	1536	X	3	0.86	0.79	0.92
ARG1	383	6	9	0.86	0.74	0.94
CD3E	916	11	4	0.88	0.25	0.71
IL8	3576	4	4	0.88	0.96	0.23
PSME4	23198	2	71	0.89	0.99	0.22
IL12B	3593	5	28	0.90	0.78	0.77
TNFSF14	8740	19	14	0.91	0.55	0.35
FASLG	356	1	18	0.93	0.94	0.77
TRANCE	8600	13	22	0.93	0.87	0.95
CD39	953	10	4	0.94	0.24	0.11
FLT3	2322	13	83	0.96	0.94	0.82
FCRL3	115352	1	17	0.96	0.92	0.77
INDO	3620	8	10	0.99	0.73	0.56
IL6R	3570	1	35	0.99	0.88	0.77
HSP70	3308	5	4	1.00	0.64	0.35
CD3D	915	11	0	-	-	-
IL10	3586	1	0	-	-	-
STAT5A	6776	17	0	-	-	-
TGFB1	7040	19	0	-	-	-
